# Social differences in avoidable mortality between small areas of 15 European cities: an ecological study

**DOI:** 10.1186/1476-072X-13-8

**Published:** 2014-03-12

**Authors:** Rasmus Hoffmann, Gerard Borsboom, Marc Saez, Marc Mari Dell’Olmo, Bo Burström, Diana Corman, Claudia Costa, Patrick Deboosere, M Felicitas Domínguez-Berjón, Dagmar Dzúrová, Ana Gandarillas, Mercè Gotsens, Katalin Kovács, Johan Mackenbach, Pekka Martikainen, Laia Maynou, Joana Morrison, Laia Palència, Gloria Pérez, Hynek Pikhart, Maica Rodríguez-Sanz, Paula Santana, Carme Saurina, Lasse Tarkiainen, Carme Borrell

**Affiliations:** 1 Department of Public Health, Erasmus Medical Center, P.O. Box 2040, Rotterdam, CA 3000, The Netherlands; 2 Research Group on Statistics, Econometrics and Health (GRECS), University of Girona, Campus de Montilivi, Girona 17071, Spain; 3 CIBER of Epidemiology and Public Health (CIBERESP), Madrid, Spain; 4 Department of Public Health Sciences, Division of Social Medicine, Karolinska Institutet, Stockholm 17177, Sweden; 5 Centro de Estudos de Geografia e de Ordenamento do Territorio (CEGOT), Departamento de Geografia, Colégio de S. Jerónimo, Universidade de Coimbra, Coimbra 3000-043, Portugal; 6 Department of Social research, Vrije Universiteit Brussel, Pleinlaan 2, Elsene, Brussels 1050, Belgium; 7 Subdirección de Promoción de la Salud y Prevención. Consejería de Sanidad, San Martín de Porres, 6, 28035, Madrid 28037, Spain; 8 Department of Social Geography and Regional Development, Faculty of Science, Charles University in Prague, Albertov 6, Prague 2 12843, Czech Republic; 9 Demographic Research Institute, HCSO, 1024, 1/3 Buday L. u., Budapest, Hungary; 10 Department of Social Research, University of Helsinki, P.O. Box 18, Helsinki 00014, Finland; 11 London School of Hygiene and Tropical Medicine, London, UK; 12 Department of Epidemiology and Public Health, University College London, 1-19 Torrington Place, London WC1E 6BT, United Kingdom; 13 Agència de Salut Pública de Barcelona, Plaça Lesseps, 1, Barcelona 08023, Spain

**Keywords:** Avoidable mortality, Health inequality, Small area, Urban health, Spatial analysis, Bayesian methods

## Abstract

**Background:**

Health and inequalities in health among inhabitants of European cities are of major importance for European public health and there is great interest in how different health care systems in Europe perform in the reduction of health inequalities. However, evidence on the spatial distribution of cause-specific mortality across neighbourhoods of European cities is scarce. This study presents maps of avoidable mortality in European cities and analyses differences in avoidable mortality between neighbourhoods with different levels of deprivation.

**Methods:**

We determined the level of mortality from 14 avoidable causes of death for each neighbourhood of 15 large cities in different European regions. To address the problems associated with Standardised Mortality Ratios for small areas we smooth them using the Bayesian model proposed by Besag, York and Mollié. Ecological regression analysis was used to assess the association between social deprivation and mortality.

**Results:**

Mortality from avoidable causes of death is higher in deprived neighbourhoods and mortality rate ratios between areas with different levels of deprivation differ between gender and cities. In most cases rate ratios are lower among women. While Eastern and Southern European cities show higher levels of avoidable mortality, the association of mortality with social deprivation tends to be higher in Northern and lower in Southern Europe.

**Conclusions:**

There are marked differences in the level of avoidable mortality between neighbourhoods of European cities and the level of avoidable mortality is associated with social deprivation. There is no systematic difference in the magnitude of this association between European cities or regions. Spatial patterns of avoidable mortality across small city areas can point to possible local problems and specific strategies to reduce health inequality which is important for the development of urban areas and the well-being of their inhabitants.

## Background

The concept of mortality amenable to medical care was introduced in the early 1970s by Rutstein. His working group selected over 90 conditions as “sentinel health events” from which disease, disability or death “should not occur in the presence of timely and effective care” [1]. Revisions of the aforementioned list undertaken in 1977 and 1980 [2,3] have formed the basis for practically all subsequent studies on avoidable mortality. Charlton was the first to apply the concept at the population level in England and Wales in 1974–78, also introducing the terms “avoidable deaths” and “conditions amenable to medical intervention” [4]. He narrowed the concept by excluding deaths that were not directly linked to medical care, e.g. deaths avoided by policies on tobacco control, and the concept was developed further within the Health Services Research Program of the European Community in the 1980s. This collaborative action resulted in a European Community atlas of avoidable mortality in which the work of Charlton and colleagues was extended and the boundaries of health services were interpreted as encompassing primary care, hospital care and collective health services [5]. In 2001 Tobias and Jackson produced an updated list of conditions derived from an expert consensus exercise in which the relative avoidability of death was distributed according to primary, secondary and tertiary actions [6].

The usefulness of the concept of avoidable mortality is based on the assumption that such causes of death are related to the functioning of medical care. This association has been studied in the past [7-9]. Avoidable causes of death can point at possible deficiencies in the delivery of medical care. Although their direct and simple use as indicators of quality of medical care in international comparisons is questionable [10], avoidable mortality represents the fraction of overall mortality that is more responsive to medical interventions and therefore offers insights into the scope for improvement of medical care. The link between the concept of avoidable mortality and research on health inequalities is based on the fact that medical care plays a role for the origin and reduction of socioeconomic inequalities in health and mortality [11,12]. The latter can be explained by differences in access and use of medical care by socioeconomic group [13]. Therefore the analysis of socioeconomic differences in avoidable causes of death can offer important lessons for tackling health inequalities. Following this reasoning, studies have looked at social differences in avoidable mortality [14] and whether access to medical care explains socioeconomic differences in avoidable mortality [15].

We combine the avoidable mortality approach with an analysis of mortality on the small area level because the socio-spatial context of the small area has been shown to be an important determinant of health and health inequality that goes beyond the effect of individual characteristics on health. This socio-spatial epidemiological framework has been proposed on the general conceptional level of “place” as a determinant of health [16,17], on the more specific level of the city [18,19], but also on the small area level as unit of analysis [20]. Studying health inequalities in small city areas is useful because, first, the percentage of urban population is increasing [19], second, health inequalities tend to be larger in cities than in rural areas because city areas tend to contain a concentration of deprivation, poverty or affluence, and third, some policies and interventions aiming at the reduction of health inequalities are approved and implemented at the city level. Therefore the monitoring of and intervening on health inequalities and its determinants at the city and small area level are especially appropriate [21]. For these reasons the use of spatial analysis of health outcomes and their predictors have been increasing in the past years. Likewise, the development of spatial methods for epidemiological analysis has rapidly improved [22,23].

In principal, the link between the socio-spatial concept of health determinants and avoidable mortality has been already established by studies observing geographical variations of avoidable mortality [4,24] but very few studies have applied this concept to the level of small areas [25,26]. While mortality differences in small areas of Spanish cities are relatively well studied [25,27], studies showing geographical patterns of socioeconomic indicators and cause-specific mortality by small area in a large number of European cities are scarce and no study has used avoidable mortality for such a comparison on an international scale. This is the first study presenting spatial patterns of avoidable mortality in small areas of several cities of different European countries and its dependence on area-level social deprivation.

We have studied 14 avoidable causes of death, first, to estimate smoothed Standardised Mortality Ratios (SMR) for the small areas of 15 European cities in the early 21th century by gender, and to represent them on maps and, second, to estimate inequalities in the level of avoidable mortality between small areas with different level of deprivation.

In this paper we analyse (1) whether levels of mortality from avoidable causes of death are higher in deprived small areas and (2) whether the magnitude of these social inequalities in mortality differs between European cities, regions and gender.

## Results

Table 1 gives an overview of the cities in our study, their number of small areas with at least one inhabitant, and illustrates our data on the study population, on mortality and on the distribution of social deprivation in percentiles. The first column shows that the number of areas differs greatly between cities, between 17 relatively large areas in Bratislava and 2666 very small areas in Turin. The distribution of social deprivation in the last three columns is also different across cities, e.g. Brussels, London and Rotterdam show higher proportions of deprived areas than Bratislava, Prague or Stockholm. Cause-specific numbers of death are in the appendix [see additional file 1].

**Table 1:** 15 European cities, number and size of their areas, period of mortality, number of deaths, and distribution of social deprivation

**City**		**Population**									**Mortality**				**Deprivation index**		
	**N. of areas**	**Year**	**Men**				**Women**				**Period**	**Number of avoidable deaths**^**a**^			**Both gender**		
			**Total**	**P25**	**P50**	**P75**	**Total**	**P25**	**P50**	**P75**		**Men**	**Women**	**Total**	**P25**	**P50**	**P75**
Amsterdam	94	2001	363,877	1630	3768	5,551	374,448	1674	3826	5766	1996-2008	6224	9044	15268	4.26	6.45	8.79
Barcelona	1491	2004	750,998	364	457	578	837,406	421	517	648	1996-2008	21,875	30,947	52,822	5.61	6.99	8.75
Bratislava	17	2004	198,778	1138	8927	16,230	226,378	1216	9795	18,360	1996-2008	4329	4764	9093	3.94	4.52	4.85
Brussels^b^	118	2001	464,364	2604	3763	5089	505,673	2958	4020	5742	2001-2004	2022	3400	5422	5.51	7.13	9.29
Budapest	23	2004	776,834	26,010	35,590	41,690	928,475	32,380	41,140	49,410	2001-2008	17,187	24,981	42,168	5.48	6.41	6.97
Helsinki	94	2004	250,567	1410	2351	3642	292,134	1524	2681	4368	2000-2009	2571	4335	6906	3.58	4.55	5.31
Košice	22	2004	112,275	598	1632	10,370	122,966	647	1741	11,820	1996-2008	2216	2492	4708	5.71	6.51	7.68
Lisbon^c^	207	2001	1,275,659	1959	4558	8278	1,386,191	2135	5065	9428	1995-2008	46,337	55,548	101,885	4.89	5.76	6.41
London^d^	633	2001	3,468,738	4835	5460	6194	3,703,293	5177	5827	6582	1995-2008	57,685	81,638	139,323	6.26	7.80	9.68
Madrid	2358	2005	1,481,721	459	576	724	1,667,894	531	663	807	1995-2007	32,979	45,665	78,644	5.56	7.83	9.75
Prague	57	2004	559,108	912	1875	13,320	611,463	906	1575	13,890	2003-2007	5617	7950	13,567	4.25	4.37	4.54
Rotterdam	83	2001	294,398	417	3276	5466	305,624	414	3260	5273	1996-2008	6047	9347	15,394	4.90	6.71	9.24
Stockholm	1171	2004	914,257	249	596	1070	950,102	257	628	1132	2000-2007	9784	13,876	23,660	2.59	3.20	4.09
Turin A	2666	2004	424,872	45	96	165	467,276	50	107	182	1995-2008	14,079	21,133	35,212	5.02	6.58	7.84
Turin B^e^	94	2004	424,872	166	5,411	9,981	467,276	211	4,895	9,238	1995-2008	14,079	21,133	35,212	4.96	6.26	8.00
Zurich	212	2004	177,970	497	801	1119	187,007	489	842	1214	1995-2008	3701	5992	9693	4.49	6.16	7.52

^a^Causes of death included and their ICD-10 codes are: AIDS/HIV disease (B20-B24, R75), MN colon (C18), MN rectum, anus, anal canal (C19-C21), MN cervix uteri (C53), MN testes (C62), Hodgkin’s disease (C81), Rheumatic heart disease (I00-I09), Hypertension (I10-I13), Heart failure (I50-I51), Cerebrovascular diseases (I60-I69), Peptic ulcer (K25-K27), Renal failure (N17-N19), Conditions originating in the perinatal period (P00-P96), Congenital heart disease (Q20-Q24).

^b^For Brussels we analyzed “Brussels Region”.

^c^For Lisbon we analyzed “Lisbon Metropolitan Area”.

^d^The analysis for London does not include mortality from Conditions originating in the perinatal period and Congenital heart disease because these causes of death were not reported in the mortality data.

^e^This second setup for Turin is used in a sensitivity analysis, see discussion.

**Figure 1:**
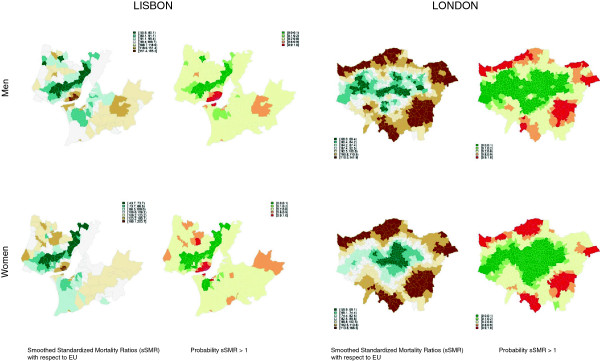
**Smoothed standardized mortality ratios for Lisbon and London and the credibility of their difference from 1.** Figure 1 shows mortality maps for avoidable mortality in the Lisbon Metropolitan Area and in London for men and women separately. The sSMR ratios in Lisbon vary from 55.8 (dark green) to 165.2 (dark brown) for men and from 43.7 (dark green) to 203.7 (dark brown) for women. The corresponding intervals for London are from 68.0 (dark green) to 141.9 (dark brown) for men and from 59.9 (dark green) to 166.5 (dark brown) for women. The colours represent smoothed Standardized Mortality Ratios (sSMR) with respect to the EU. This means, for example, that the lowest mortality level for men in Lisbon (dark green) is between 55.8 and 80.1 percent of the EU-average. Next to each mortality map showing the level of mortality for each small area, there is a map with the probability that the shown sSMRs are above 1. This is the credibility level and represents the Bayesian correspondent to confidence intervals. On this credibility map, red colour indicates a probability of 90-100% that an sSMR is higher than 1 and green colour indicates with the same probability that it is lower than 1.

Due to space limitations we can only show mortality maps for two out of 15 cities in Figure 1. The remaining maps for avoidable mortality are in the appendix [see additional file 2] and also the cause-specific maps [see additional files 3, additional file 4, additional file 5, additional file 6, additional file 7, additional file 8, additional file 9, additional file 10, additional file 11, additional file 12, additional file 13, additional file 14, additional file 15, additional file 16 and additional file 17]. Figure 1 shows that mortality from avoidable causes shows a clear spatial pattern in both cities. In Lisbon small areas with significantly higher mortality (dark brown) are in the very center of the city, while there is lower mortality mainly in the north-west but also in the south (green). In London however, the city center is characterized by lower mortality, and the areas with lower mortality extend more to the West, East and South for men than for women. All areas at the edge of London show higher mortality except for the West. In summary, Lisbon has a city center with relatively high mortality and the center of London has relatively low mortality. In both cities disadvantaged areas are larger for men than for women. The relative mortality level with respect to the EU average can be better displayed in the box-plots presented in the next section.

**Figure 2:**
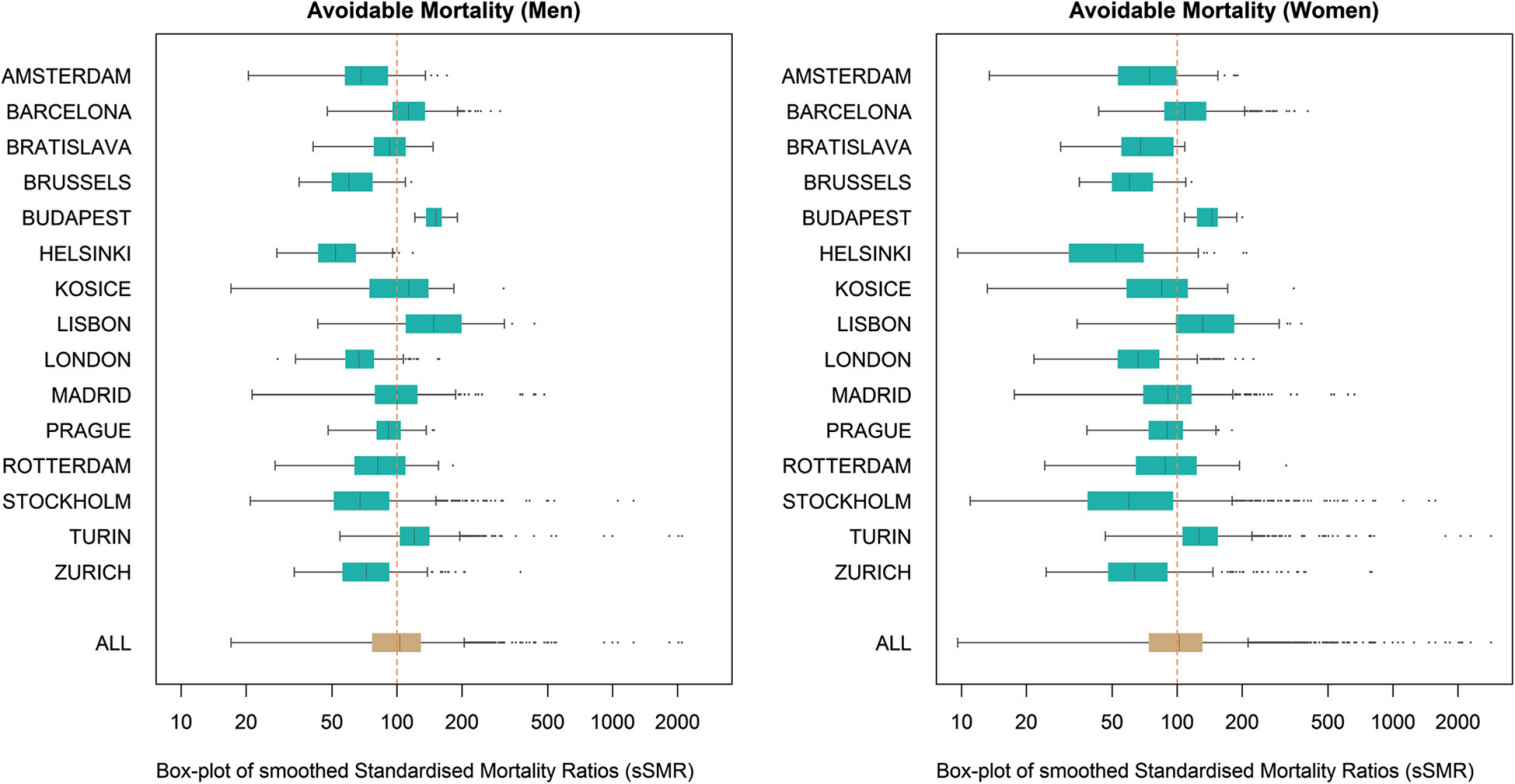
**Box-plots for avoidable mortality in small areas of 15 European cities.** The box-plots show the range of mortality between the areas with the lowest and highest mortality in each city. The rectangles are the range between the 25th and 75th percentile and single dots represent single areas that are considered as outliers with very high mortality. The box-plot for “ALL” at the bottom shows the simple aggregation of all areas of all cities and is therefore dominated by cities with many areas. With these graphs it is possible to compare the level of mortality of a city relative to the EU-average, and to see the range of mortality across areas of one city.

Figure 2 provides box-plots for all 15 cities for men and women respectively. Cities with a mortality level clearly below the EU average tend to be in Central and Northern Europe (mainly Amsterdam, Brussels, Helsinki, London, Stockholm and Zurich) while cities with high mortality are in the East and in the South (mainly Budapest, Lisbon, Turin). This pattern is generally true for both men and women with the exception of Bratislava where female avoidable mortality is well below the EU average. Women show wider ranges of mortality across areas in almost all cities. The cause-specific box-plots are shown in the appendix [see additional file 18].

**Figure 3:**
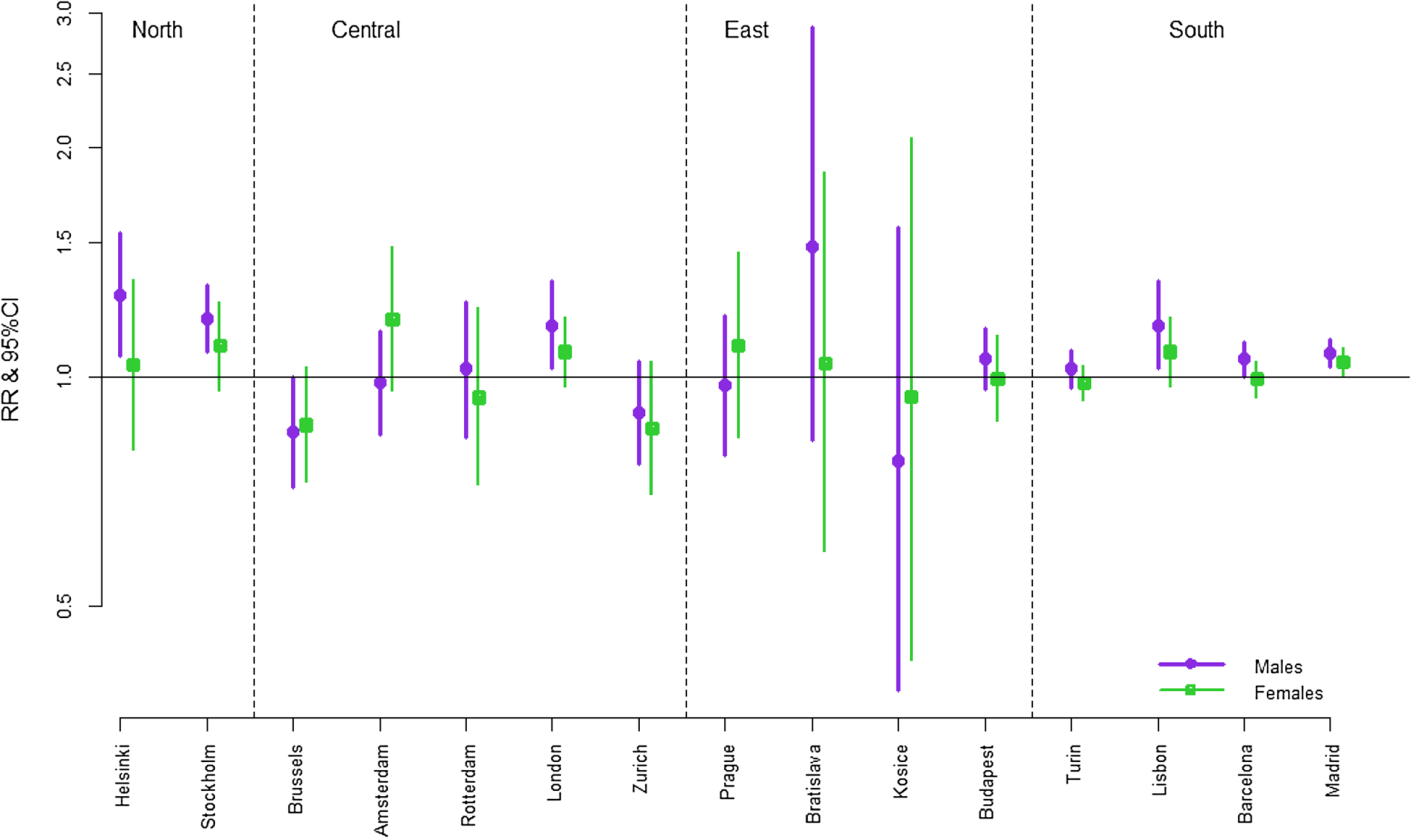
**Avoidable mortality rate ratios between 1st and 4th quartile of social deprivation.** The graph shows the excess mortality of the quartile with most deprivation of all small areas in a city relative to the quartile with least deprivation of all small areas, complemented by the 95% credibility interval. For example, among men in Helsinki, more deprived areas have higher mortality from avoidable causes than less deprived areas (RR: 1.28; 95% CI: 1.07-1.54).

Figure 3 presents the results of the ecological regression of mortality on social deprivation exploring the association between the index of social deprivation and avoidable mortality. Most rate ratios in Figure 3 and all statistically significant rate ratios indicate a positive association between area deprivation and avoidable mortality. However there is also a considerable number of rate ratios below 1, albeit not statistically significant. The excess risk of mortality is not significantly different between men and women, although in most cities there is a higher association for men than for women. The international comparison across European regions shows a slight tendency towards higher rate ratios in Northern Europe and low rate ratios in Southern Europe. But overall, we do not find a systematic pattern in the magnitude of health inequality between European regions or cities. The cause-specific rate ratios are presented in the appendix as a table [see additional file 19] and as graphs [see additional file 20].

## Discussion

This study is the first that offers an international comparison of avoidable mortality at the small area level and its association with social deprivation of the area. Our results show that there are significant differences in the level of avoidable mortality between neighbourhoods. We showed that higher and lower levels of mortality cluster into a geographical pattern across the city area, such as center versus periphery, or north, south, east or west. Mortality from avoidable causes of death is often but not always higher in deprived areas with higher social differences among men than among women. We could not identify cities, countries or European regions that consistently show higher or lower health inequality.

We also cannot confirm findings from previous studies that larger cities are more unequal [27].

Our findings can extend the current knowledge by showing spatial patterns of avoidable mortality in many cities of several European countries, identifying small areas in each city with an excess risk of avoidable mortality and thereby pointing at problematic areas that could potentially benefit from urban policies addressing avoidable causes of death and social inequalities in these deaths. Because the use of the concept of avoidable mortality is always dependent on how avoidability is defined it is important to note that our selection of causes of death includes deaths that can be avoided by medical care and not by health care or social policy in a wider sense.

The results from our international comparison across Europe cannot be directly compared to previous findings because our study is the first to provide such results. However, the lack of a strong pattern of different magnitudes of health inequalities between different regions of Europe is surprising and requires some tentative explanations because at the individual level the magnitude of health inequality differs across countries and European regions. One previous study compared the effect of small area unemployment on all-cause mortality between cities from six different countries [28]. The authors concluded that this effect is not substantially modified by the country, although one could have expected such international differences based on differences in absolute levels (and ranges) of deprivation or based on different national policies to address health inequalities. We tend to agree to their interpretation that there seems to be a general mechanism that links area level deprivation to mortality across many European cities, either because these cities are not different enough, or because they are just similar social worlds of relative social deprivation and its effect on health.

### Limitations

One major limitation is the low statistical power of our analysis that focuses on numbers of deaths in relatively small areas. Credibility intervals are large and in the cause-specific analysis presented in the appendix, several cases had to be excluded due to too many areas with zero deaths [29]. This makes it difficult to systematically compare cause-specific results between cities and it was the reason to let the main part of the study focus on the 14 aggregated causes of death in order to draw general conclusions on avoidable mortality. The problem of low numbers of death can hardly be avoided if one wants to look at mortality in small areas, because, first, larger areas would increase the heterogeneity within each area in terms of deprivation and mortality and, second, a longer period of observation over which rare deaths could be aggregated would necessarily introduce more bias from migration, changing borders between small areas and other changes over time. To obtain acceptable numbers of death we had to aggregate mortality data from several years which assumes that other circumstances such as migration and consequent change of the exposed population are negligible and do not bias the results substantially [30,31].

Second, the number of areas differs greatly between cities. The median population size per area varies between about 100 persons in Turin and about 40,000 in Budapest (Table 1). Smaller areas tend to be more homogeneous and the result is potentially higher variability between the areas. This influences the variance in the deprivation index between areas. Differences in observed SMRs could also be dampened as higher concentrations of mortality in smaller areas will dissolve in larger areas. Inversely, more extreme variance in SMRs could appear by using smaller areas. As a consequence, the comparison between cities could be difficult, because the observed variance is a result both of bigger disparities in social status and of the area size. However, our method takes into account this effect by controlling for spatial dependence of small areas, that is to say for similarities of neighbouring areas. This results in clustering of similar small areas. In fact, previous studies have shown that the bias due to different level of units within a city is relatively small [32]. We also examined this possible bias by a sensitivity analysis in which we used an alternative division into fewer areas for Turin, the city with the smallest areas in our study. In the original analysis Turin had 2666 small areas and the second available official division from the city of Turin has 94 areas. These two alternative setups are presented in Table 1. The result of this sensitivity analysis is that the rate ratios for the effect of deprivation on avoidable mortality changed from 1.02 (CI: 0.97-1.09) and 0.98 (CI: 0.93-1.04) to 0.99 (CI: 0.77-1.05) and 1.08 (CI: 0.90-1.30) for men and women respectively. While the point estimates hardly change, the confidence intervals become wider due to fewer areas. More importantly, we do not see lower rate ratios as could be expected with fewer areas, but rather the opposite. Turin was the only city where an alternative division into areas was available and we conclude that our overall results and conclusions are not sensitive to the number of areas. Maps for avoidable mortality for Turin with 2666 and 94 areas can be found in the appendix [see additional file 2].

Next to these limitations we can point at exceptional strengths. First, we created a unique data collection on cause-specific mortality data on the small area level of 15 European cities and on several indicators of social deprivation of these areas. Second, these indicators were the same across all cities of all countries, and by that we solved a common problem in international comparative studies of health inequalities. Third, we used a powerful and established analytic method for the ecological analysis of the association between deprivation and mortality level. This method allows producing smoothed estimates based on relatively low number of cause-specific deaths in small areas, minimizing potential bias, and still presenting a valid spatial pattern in each city. The appropriateness of Bayesian hierarchical modelling for this purpose is widely recognized [33].

## Conclusions

Our study shows clear differences in the level of avoidable mortality between neighbourhoods of European cities and the level of avoidable mortality is in general positively associated with social deprivation. There is no systematic difference in the magnitude of this association between European cities or regions. It is important to monitor avoidable mortality on the level of small areas because they have the potential to point to specific areas with need for specific medical care and they also reflect inequalities with regard to medical care at the individual level. But without sufficient data on medical care services on the city and on the small area level, it is very difficult to conclude from the mortality level in a small area to specific problems. Therefore, cause-specific mortality maps can only be used to point at potential problems in deprived small areas, which then have to be studied with more specific information and better local data on the relation between medical care and health outcome.

## Methods

Based on previous work on avoidable causes of death, we selected 14 causes of death amenable to medical intervention (see footnote in Table 1) [34]. This selection covers causes of death amenable to primary and secondary prevention (e.g. vaccination, cancer screening) as well as causes of death amenable to treatment (e.g. surgery, chemotherapy), but not deaths avoided by health policy in general, e.g. tobacco control. Our choice is restricted to causes of death for which numbers can be expected to be large enough to allow small area analysis.

The data we use for this analysis are, first, mortality data by cause of death aggregated during a period of several years around the year 2001 (see Table 1), by age, gender and small area for 15 European cities from the respective national statistical offices, and second, information on several social indicators from censuses around the year 2001, except for the two Dutch cities where a labour force survey was used. The 15 cities represent four main European regions (north, central, east, south). The expected numbers of deaths in each area of the cities were calculated with the population in the whole period (although for some cities the population in one year was multiplied by the years in the study period) and taking as reference mortality rates by gender, age (5 year age-specific mortality rates) and cause of death in 25 countries that were part of the 27 countries of the European Union in 2004 [35].

### Index of deprivation

When constructing the index of deprivation, we used five socioeconomic indicators: percentage of unemployed persons, percentage of manual workers, percentage of population aged 25–64 with primary education or lower, percentage of population aged 25–34 with a university degree, and percentage foreigners from low income countries. These indicators were selected out of a set of 13 indicators for social status available in our dataset. Of the eight indicators discarded, two indicators related to lower education were removed for conceptual reasons. In the case of the percentage of people with primary education or lower, we preferred to use the 25–64 age range (instead of the 25–34 age range) because six cities did not have data for the 25–34 age group. Two age ranges for percentage of people with a university degree were available in most of the cities. In this case, we chose the indicator measuring the percentage in the 25–34 age range, because access to university was very low in the age range 25–64. Two indicators were discarded because they barely contributed anything to the percentage of variance explained by the five indicators finally chosen: activity rate of the population and non-home ownership. The indicators that reflected the percentage of temporary workers or the percentage of part-time workers were excluded due to lack of information in some cities (nine cities in the case of temporary contracts and five in the case of part time work) and also because in most cities such working conditions were not directly related to deprivation but rather depended on the country’s labour market conditions. In fact, the correlations between unemployment (which is commonly regarded as the most important indicator for social deprivation) and these two variables were relatively small (0.328 for temporary workers and 0.121 for part-time workers), especially for women working part-time (0.081). The reasons for the exclusion of the variables indicating single parent households and overcrowding were similar: neither seems to be clearly related to deprivation. The correlation with unemployment was either very small (0.061 for overcrowding) or even negative (−0.106 for single parent household).

The deprivation index was constructed by aggregating the above-mentioned variables using the distance indicator, DP_2_[36]. This indicator permits to obtain an index that is comparable across cities. Albeit not carrying out the widely used principal component analysis (PCA) [37,38], the original variables we used are very similar to other small area studies [25,27]. Let x_i_ be the vector of the state of the components (indicators) in the situation i, and x_ij_ be the state of the component j in situation i. Let x_i*_ be the reference vector. This vector can represent an ideal situation where x_i*j_ is the state of component j in the reference situation. In order to compare x_i_,x_i*_ the DP_2_ index is defined in the following manner:

$$DP_{2}=\sum_{j=1}^{p}\frac{\left|x_{\mathrm{ij}}-x_{i^{*}j}\right|}{\sigma_{j}}\left(1-R_{j,j-1,j-2,\ldots ,1}^{2}\right)$$

Where $R_{j,j-1,j-2,\ldots ,1}^{2}$ is the coefficient of determination in the regression of x_j_ over *x*_*j*−1_, *x*_*j*−2_ ,…, *x*_1_ . This coefficient is independent of the unit of measure of the variables. $R_{1}^{2}$ =0, given that the first variable contributes all its information as there is no previous variable, and the weight assigned to it is 1. The standard deviation σ_*j*_ corresponds to the component j. Dividing the distance for component *j* by σ_*j*_ the indicator is dimensionless. Moreover, this distance is weighted by the inverse of σ_*j*_, so that its contribution to the index is inversely proportional to its dispersion. The main weights are given by $\left(1-R_{j,j-1,j-2,\ldots ,1}^{2}\right)$. These factors eliminate the redundant information of the indicators, separating these from the variability already explained by other preceding indicators. The DP_2_ is constructed following an iterative procedure. The order in which the indicators are introduced alters the final result. In order to control this, the iterative process of Ivanovic is applied [39]. Each indicator is introduced according to its linear correlation previously calculated. Iteration continues until the order of the indicators is stabilized. The final DP_2_ distance for case *i* with respect to reference i* (which is the most favourable value for each indicator) shows the correct order of inclusion for the indicators [36]:

$$\begin{array}{ll} \;DP_{2}= & \frac{\left|x_{i1}-x_{i^{*}1}\right|}{\sigma_{1}}+\frac{\left|x_{i2}-x_{i^{*}2}\right|}{\sigma_{2}}\left(1-R_{2,1}^{2}\right)\\ & +\frac{\left|x_{i3}-x_{i^{*}3}\right|}{\sigma_{3}}\left(1-R_{3,2,1}^{2}\right)+\ldots \\ & +\frac{\left|x_{\mathrm{ip}}-x_{i^{*}p}\right|}{\sigma_{p}}\left(1-R_{p,p-1,\ldots ,1}^{2}\right) \end{array}$$

### Standardised mortality ratios

Our mortality maps show Standardized Mortality Ratios (SMR) which are expressed as [observed cases/expected cases]*100. When analysing aggregated data from small areas of a city it is important to account for two sources of variability: first, the spatial dependence between geographical areas, which means that neighbouring areas are more likely to have a similar deprivation and mortality level than distant areas; second, the non-spatial variability (random variation). To solve the problems associated with SMRs for small areas with low number of deaths we smooth them using the Bayesian model proposed by Besag, York and Mollié (BYM-model) [40] obtaining smoothed SMR (sSMR). The geographical distributions of sSMR values derived from the BYM models are displayed using maps of the septiles of the sSMR. Areas with dark green colours have the lowest sSMR and those with dark brown tones have the highest. The model used was:

$$O_{i}\sim \mathrm{Poisson}\left(E_{i}\theta_{i}\right)$$

$$\log\left(\theta_{i}\right)=\alpha +S_{i}+H_{i}$$

*O*_*i*_ denoted the observed cases of deaths for a particular cause and gender in the small area i of a city; *E*_*i*_ was the expected number of deaths (of such cause and gender) in the small area i, and *θ*_*i*_ the relative risk for each specific area. *α* represents the intercept, S_i_ spatial random effects and H_i_ denotes heterogenous (non-spatial) effects.

In the mortality maps, for each city, the sSMR for each area were estimated as follows:

$$\mathrm{sSM}R_{\_i\left(c\right)}=\exp\left(S_{i}+H_{i}\right)$$

In the box-plots showing the range of mortality across small areas for each city, the sSMR for each area were estimated as follows:

$$\mathrm{sSM}R_{\_i\left(\mathrm{eu}\right)}=\exp\left(\alpha +S_{i}+H_{i}\right)$$

Note that the sSMRs for the mortality maps exclude the intercept of the Poisson model *α* in order to be comparable to the average mortality level of the city (indicated by the subscript c), while the sSMRs for the box-plots include the intercept and thereby refer to the European mortality level (indicated by the subscript eu).

### Ecological regression

We analysed how the cause-specific level of mortality in a small area is associated with the degree of social deprivation. In each of the cities, for each of the causes of death and for each gender, the observed cases of death were assumed to follow a Poisson distribution,

$$O_{i}\sim \mathrm{Poisson}\left(\mu_{i}E_{i}\right)$$

where μ_i_ was the relative risk in the small area i. In turn, the relative risk could be associated with the explanatory variables by means of an ecological regression. In our case, this regression was formulated as follows:

$$\log\left(\mu_{i}\right)=\alpha +\sum_{j=2}^{4}\beta_{j}XQ_{j,i}+S_{i}+{Η}_{i}$$

where XQ_j,i_ denoted the j-th quartile of the socioeconomic indicator X (the quartiles were constructed within each city) – the first quartile (corresponding to the lowest deprivation) was taken as a reference value - and α and the βs were unknown parameters.

We were interested in the relative risk associated with each quartile of the socioeconomic indicator, i.e. $e^{\beta_{2}}$ for the second quartile; $e^{\beta_{3}}$ for the third quartile and $e^{\beta_{4}}$ for the fourth quartile (always with respect to the first quartile). However, in the result section we only present rate ratios for the fourth quartile (most deprived) relative to the first (least deprived). Using quartiles of the city specific social distribution of small areas implies that our measure of deprivation is a relative measure. No absolute categories of social deprivation were applicable across 15 cities of different European countries and regions.

The deprivation index was included in quartiles in the ecological regressions for two reasons: first, to capture a possible nonlinear relationship between the indicators (including the deprivation index) and the response variable, second, to avoid the effects of concurvity, the non-linear analogue of multi-collinearity, as a consequence of a high correlation between the covariates and the clustering term. The ecological associations we present are likely to represent both the effect of individual level socioeconomic status on health and the effect of the area level social deprivation, which again can consist of social and physical pathways. We used the software WinBUGS and the R statistical package. All maps are plotted using R and we have used a diverging color scheme brown/blue-green which has been shown to be an effective choice of pair hues for the representation of mortality ratios on choropleth maps [41].

## Competing interests

The authors declare that they have no competing interests.

## Authors’ contributions

RH drafted the paper, made the main conception and design of the paper and interpretation of data, and contributed to the analysis. GB, MS, MMDO made or contributed to data analysis and interpretation and revised the paper. CB, JMo, JMa have contributed to conception and design and interpretation of data and revised the paper. BB, DC, CC, PD, FD, DD, AG, MG, KK, PM, LP, GP, HP, MRS, PS, LT prepared data for the analysis and revised the paper. LMP, CS contributed to data analysis and revised the paper. All authors read and approved the final manuscript.

## Supplementary Material

Additional file 1 Cause-specific numbers of death. Click here for file

Additional file 2 Avoidable mortality maps for remaining 13 cities. Click here for file

Additional file 3 Cause-specific mortality maps for Amsterdam. Click here for file

Additional file 4 Cause-specific mortality maps for Barcelona. Click here for file

Additional file 5 Cause-specific mortality maps for Bratislava. Click here for file

Additional file 6 Cause-specific mortality maps for Brussels. Click here for file

Additional file 7 Cause-specific mortality maps for Budapest. Click here for file

Additional file 8 Cause-specific mortality maps for Helsinki. Click here for file

Additional file 9 Cause-specific mortality maps for Kosice. Click here for file

Additional file 10 Cause-specific mortality maps for Lisbon. Click here for file

Additional file 11 Cause-specific mortality maps for London. Click here for file

Additional file 12 Cause-specific mortality maps for Madrid. Click here for file

Additional file 13 Cause-specific mortality maps for Prague. Click here for file

Additional file 14 Cause-specific mortality maps for Rotterdam. Click here for file

Additional file 15 Cause-specific mortality maps for Stockholm. Click here for file

Additional file 16 Cause-specific mortality maps for Turin. Click here for file

Additional file 17 Cause-specific mortality maps for Zurich. Click here for file

Additional file 18 Cause-specific box-plots graphs. Click here for file

Additional file 19 Cause-specific mortality rate ratios (Table). Click here for file

Additional file 20 Cause-specific mortality rate ratios (Graphs). Click here for file
