# Supplementary material for: Social differences in avoidable mortality between small areas of 15 European cities: an ecological study
Source: Int J Health Geogr. 2014 Mar 12;13:8. doi: 10.1186/1476-072X-13-8 (PMC4007807; doi:10.1186/1476-072X-13-8)
Supplement: Additional file 1 — Cause-specific numbers of death. [file 1476-072X-13-8-S1.pdf]

Appendix Table 1: Numbers of death included in the analysis, by cause and gender

Cause of Death	AIDS (HIV disease)		MN colon		MN rectum, anus, anal canal		MN cervix uteri	MN testes	Hodgkin's disease		Rheumatic heart disease		Hypertension		Heart failure		Cerebro-vascular diseases		Peptic ulcer		Renal failure		Perinatal Deaths[a]		Congeni-tal heart disease	
**ICD-9**	042-044		153		154		180	186	201		390-398		401-404		428-429		430-434,436-438		531-533		584-586		760-779		745-746	
**ICD-10**	B20-B24, R75		C18		C19-C21		C53	C62	C81		I00-I09		I10-I13		I50-I51		I60-I69		K25-K27		N17-N19		P00-P96		Q20-Q24	
**Gender** / **City**	m	f	m	f	m	f	f	m	m	f	m	f	m	f	m	f	m	f	m	f	m	f	m	f	m	f
Amsterdam	462	76	805	962	297	255	210	12	17	8	6	9	211	371	1256	2196	2498	4199	100	171	334	393	180	162	46	32
Barcelona	1366	350	3507	3127	1094	804	417	15	92	85	299	768	1370	3042	4099	8327	7943	12064	226	211	1612	1557	176	133	76	62
Bratislava	5	1	630	491	577	450	307	27	14	17	43	70	670	734	373	445	1422	1669	225	177	256	341	73	52	14	10
Brussels	29	18	260	363	80	109	55	4	8	5	15	51	87	259	486	858	866	1472	59	61	125	146	0	0	3	3
Budapest	27	6	2520	2703	1220	1137	743	56	39	39	146	317	2250	4416	1768	2277	7778	12055	814	777	239	276	255	198	75	37
Helsinki	23	4	277	366	186	185	65	2	6	7	15	48	112	235	262	303	1512	2920	111	116	48	63	4	1	13	22
Košice	1	0	234	232	243	157	144	8	21	7	37	30	472	629	102	115	812	937	98	59	102	106	69	62	17	14
Lisbon	6003	1490	4977	4060	2031	1462	932	76	131	104	288	686	1291	2463	4125	6798	23473	33910	741	617	2552	2466	504	398	145	162
London	1013	348	6448	6994	3738	3053	1662	83	259	177	653	1681	3725	4152	8031	14258	28551	43877	2953	3144	2231	2292	NA[b]	NA[b]	NA[b]	NA[b]
Madrid	2942	641	5092	4406	1769	1333	572	28	143	111	454	1345	1343	2987	7026	14736	10202	15603	323	316	2987	3102	444	334	226	179
Prague	1	0	801	696	560	453	212	16	21	21	39	72	229	420	711	779	2804	4755	136	187	240	323	48	28	11	4
Rotterdam	105	30	843	1012	272	259	191	8	23	19	2	11	175	356	1585	2393	2471	4355	103	165	287	432	134	97	39	27
Stockholm	92	22	1077	1234	580	482	281	17	21	19	48	95	364	672	2163	3653	4499	6592	334	315	437	377	115	91	37	43
Turin	372	92	1769	1731	677	619	140	18	49	36	155	334	1580	2911	2142	3862	6333	10465	234	229	569	566	136	104	45	44
Zurich	285	114	433	461	191	197	70	8	13	11	53	106	501	985	594	1257	1363	2445	77	138	82	106	89	78	12	24

[a]Perinatal Deaths = Conditions originating in the perinatal period

[b]NA=These causes of death were not reported in the mortality data
