# Supplementary material for: Social differences in avoidable mortality between small areas of 15 European cities: an ecological study
Source: Int J Health Geogr. 2014 Mar 12;13:8. doi: 10.1186/1476-072X-13-8 (PMC4007807; doi:10.1186/1476-072X-13-8)

Avoidable mortality, Amsterdam , Males, 1996−2008
Smoothed Standardised Mortality Ratios (sSMR)

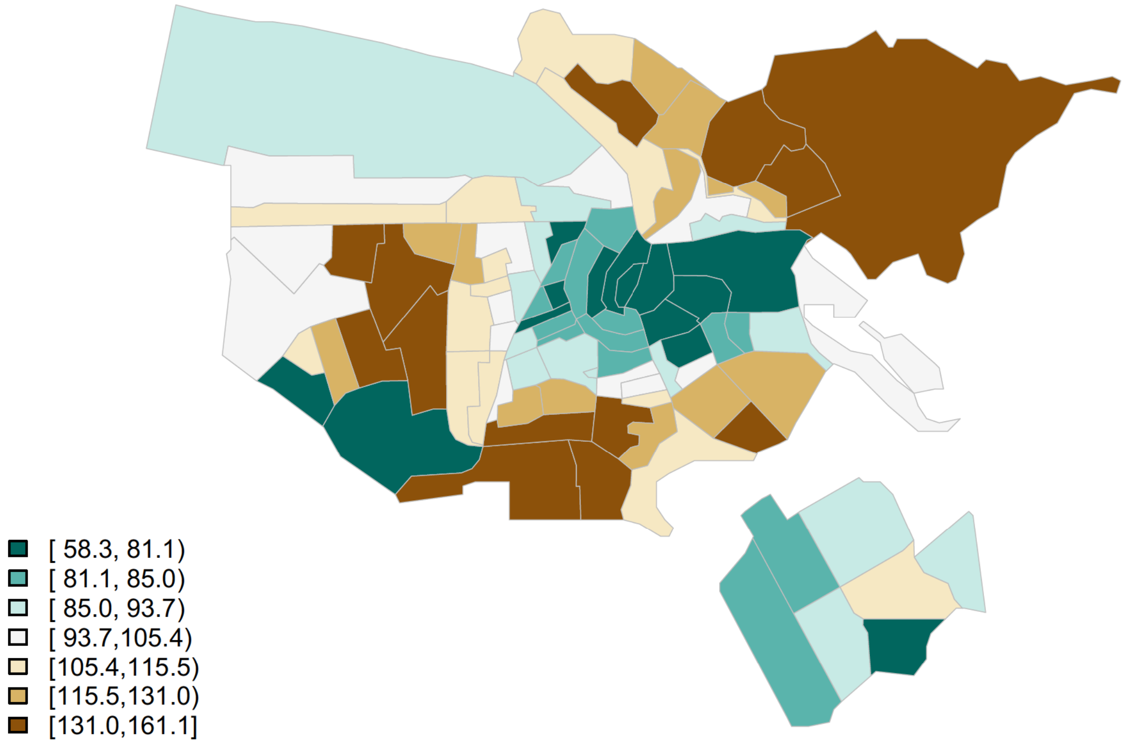

Avoidable mortality, Amsterdam , Males, 1996−2008
Probability that the sSMR is higher than 100

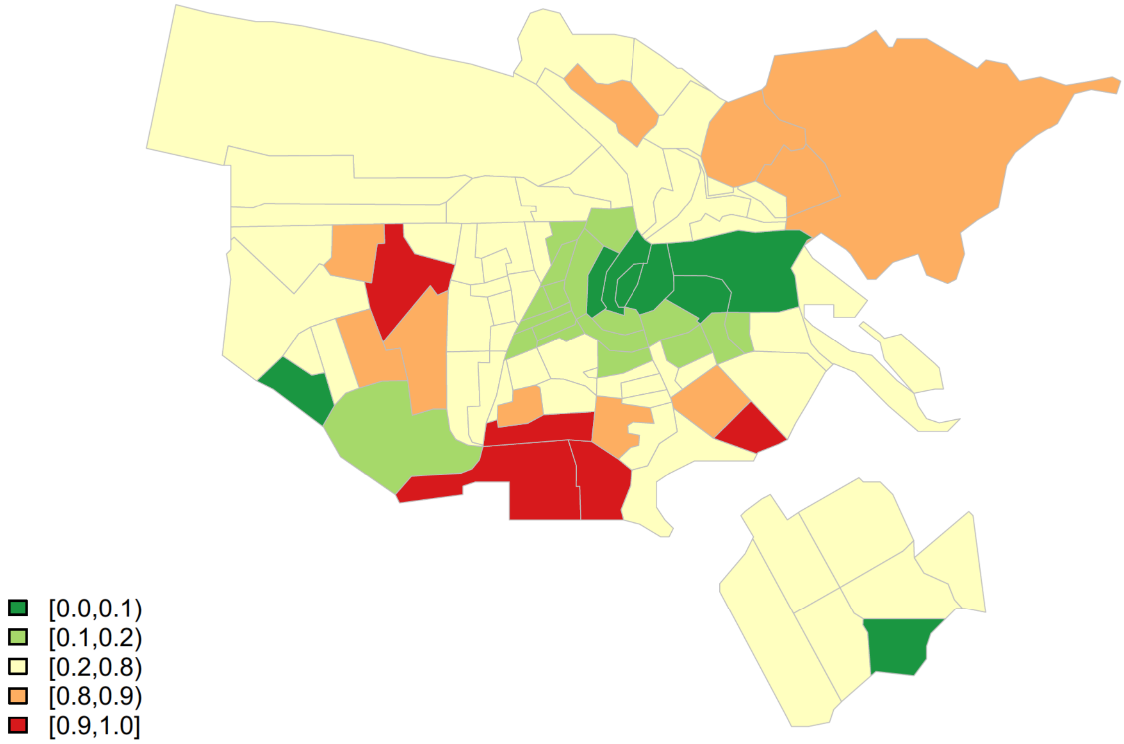

Avoidable mortality, Amsterdam , Females, 1996−2008
Smoothed Standardised Mortality Ratios (sSMR)

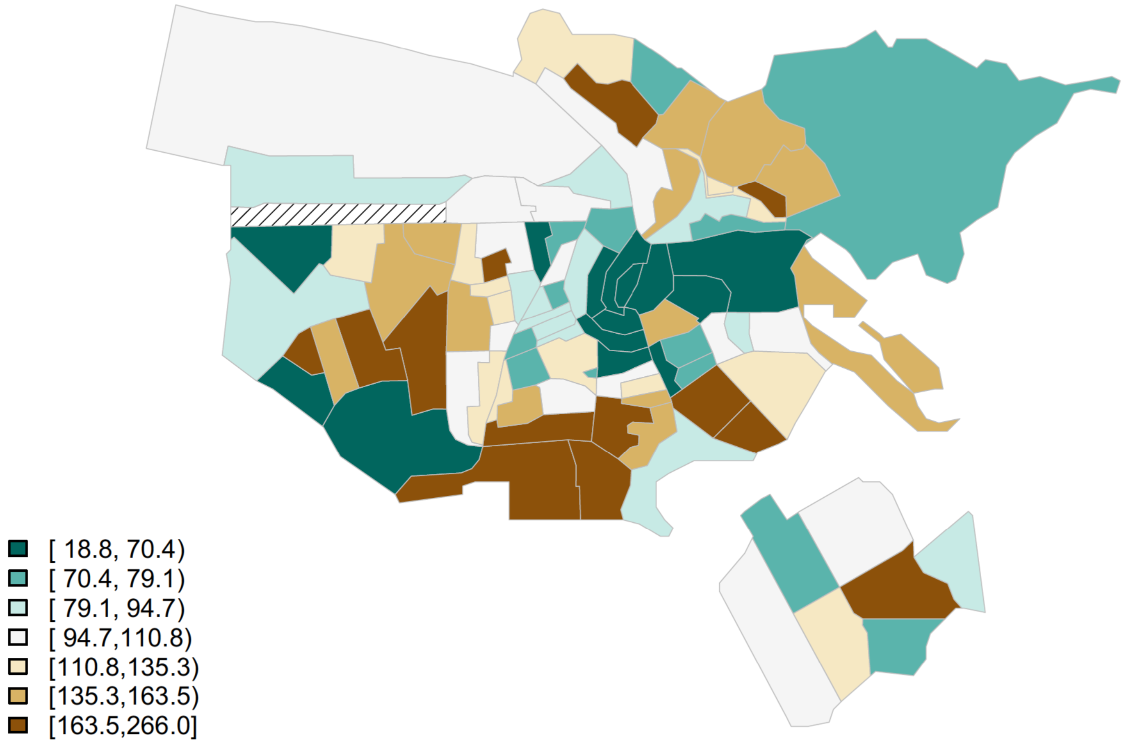

Avoidable mortality, Amsterdam , Females, 1996−2008
Probability that the sSMR is higher than 100

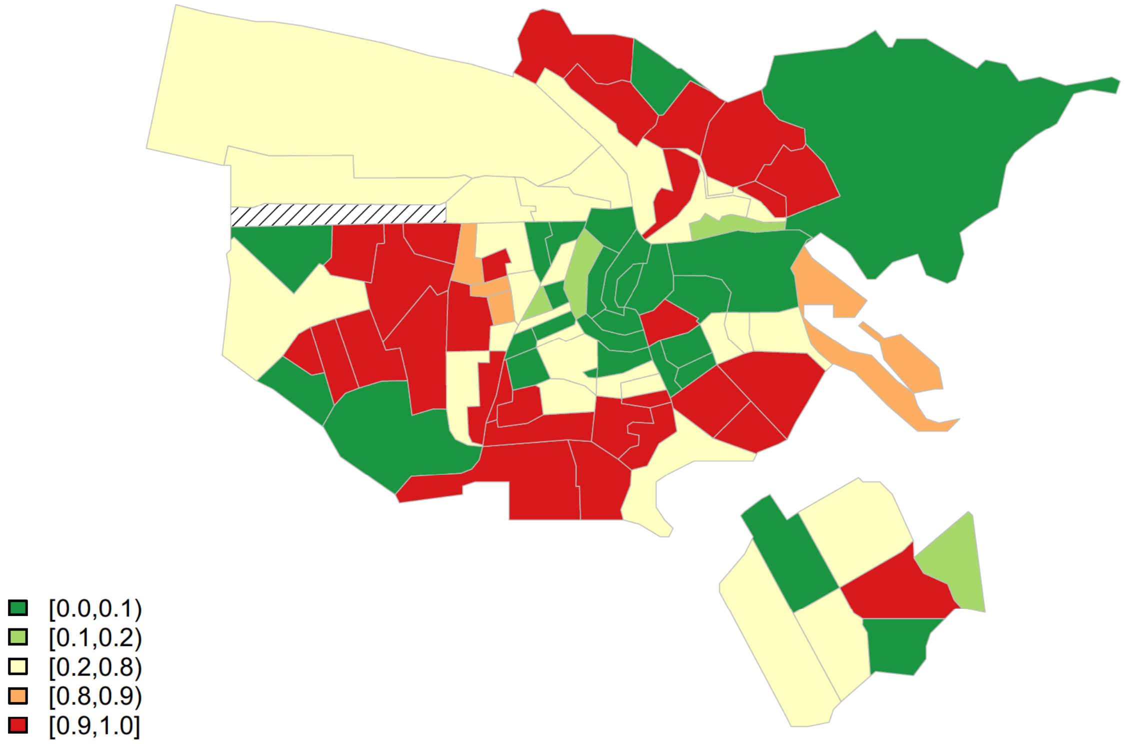

Avoidable mortality, Barcelona , Males, 2000−2008
Smoothed Standardised Mortality Ratios (sSMR)

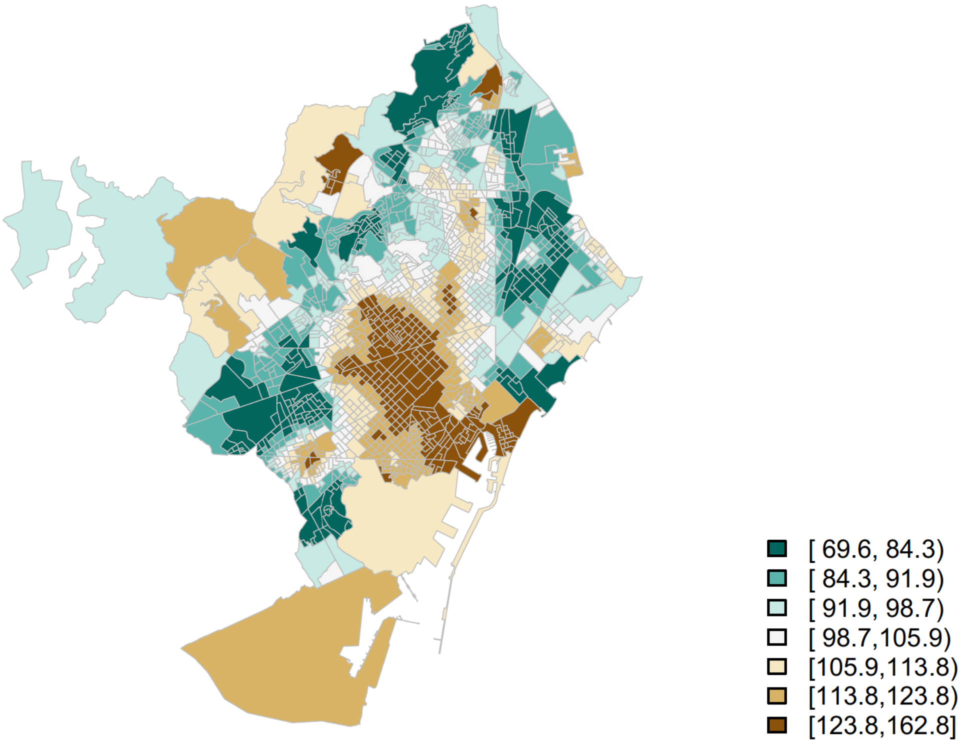

Avoidable mortality, Barcelona , Males, 2000−2008
Probability that the sSMR is higher than 100

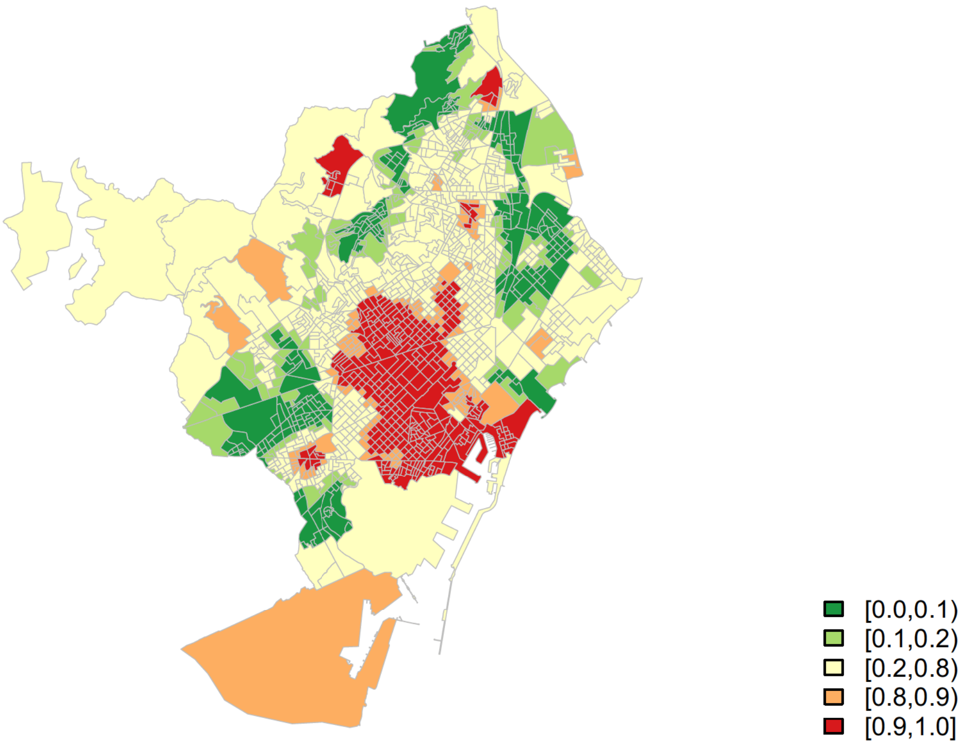

Avoidable mortality, Barcelona , Females, 2000−2008
Smoothed Standardised Mortality Ratios (sSMR)

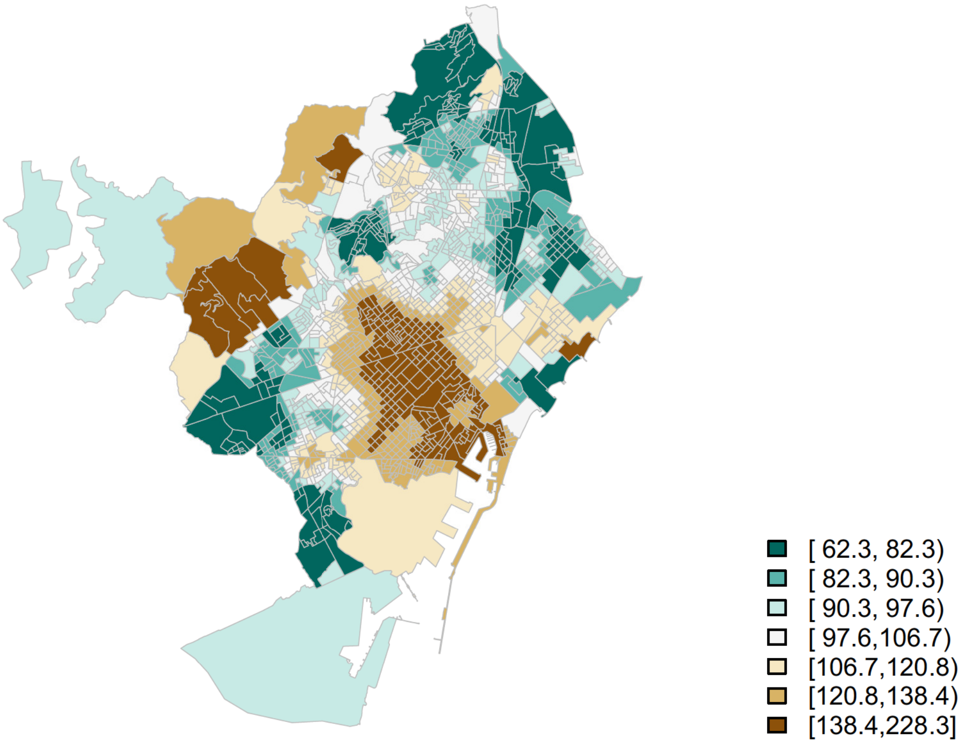

Avoidable mortality, Barcelona , Females, 2000−2008
Probability that the sSMR is higher than 100

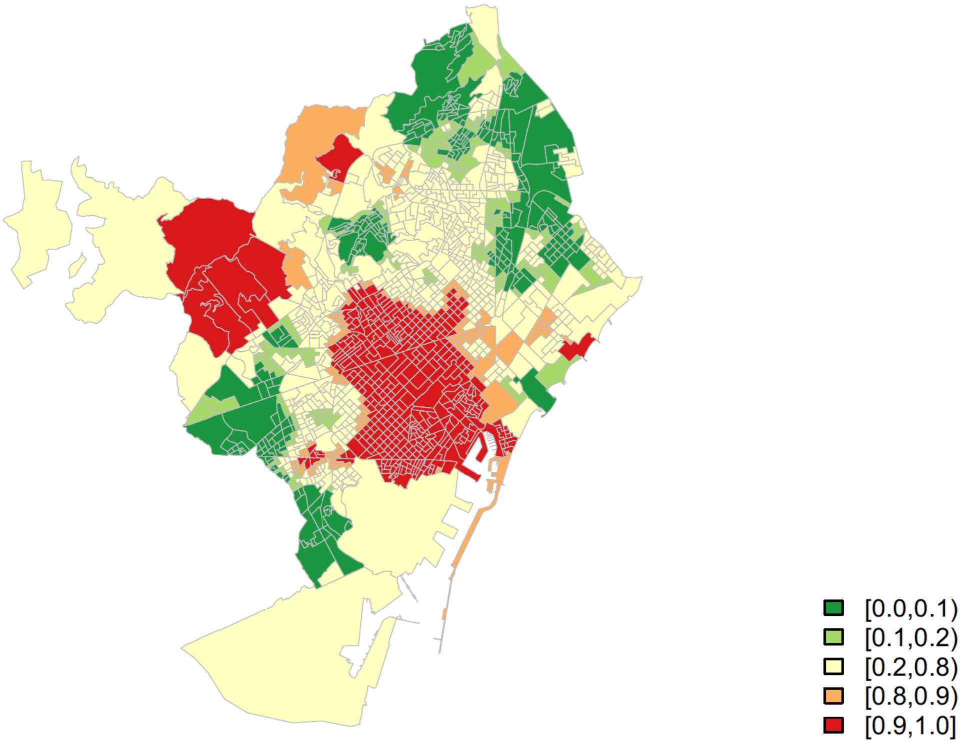

Avoidable mortality, Bratislava , Males, 1996−2008
Smoothed Standardised Mortality Ratios (sSMR)

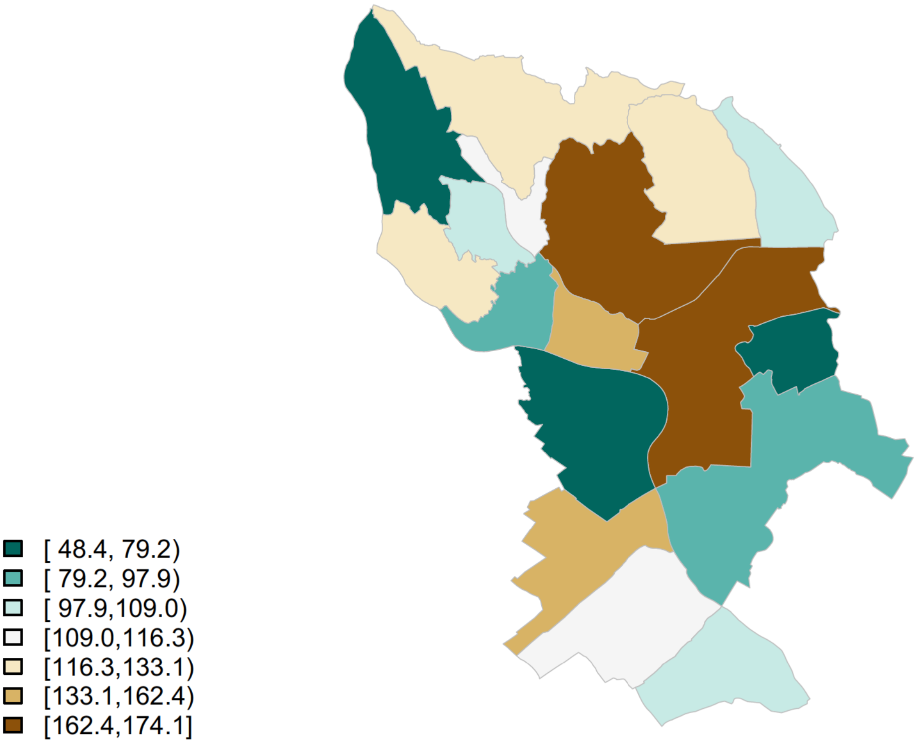

Avoidable mortality, Bratislava , Males, 1996−2008
Probability that the sSMR is higher than 100

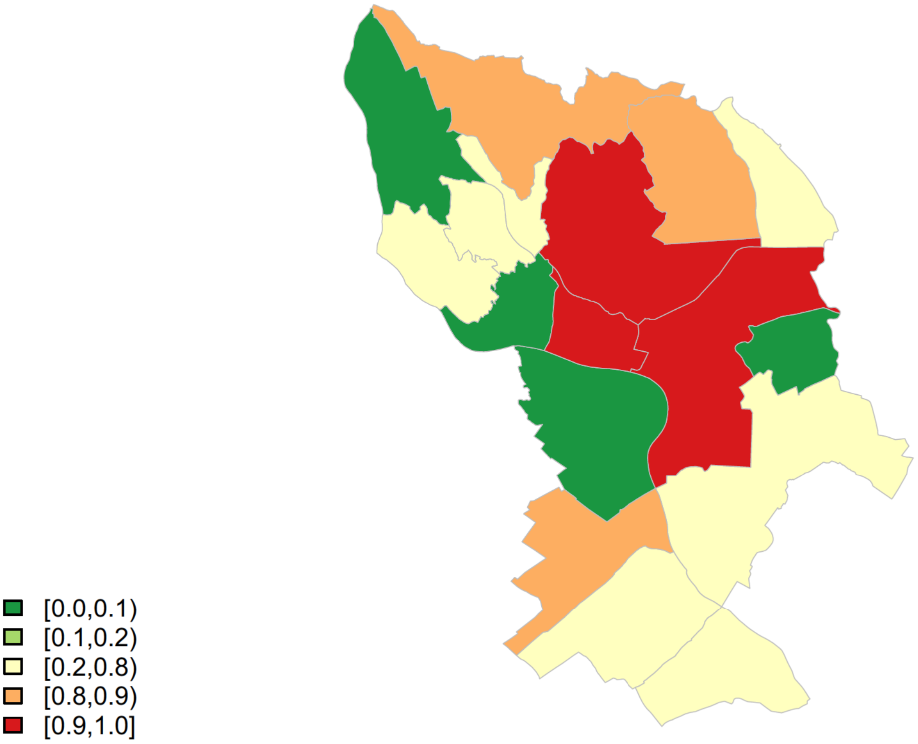

Avoidable mortality, Bratislava , Females, 1996−2008
Smoothed Standardised Mortality Ratios (sSMR)

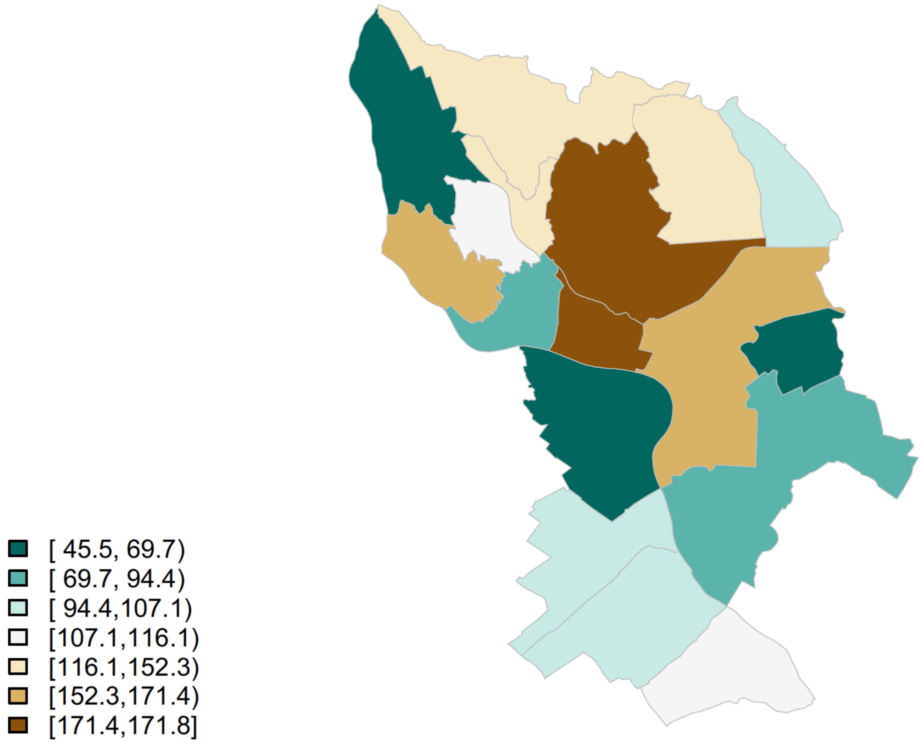

Avoidable mortality, Bratislava , Females, 1996−2008
Probability that the sSMR is higher than 100

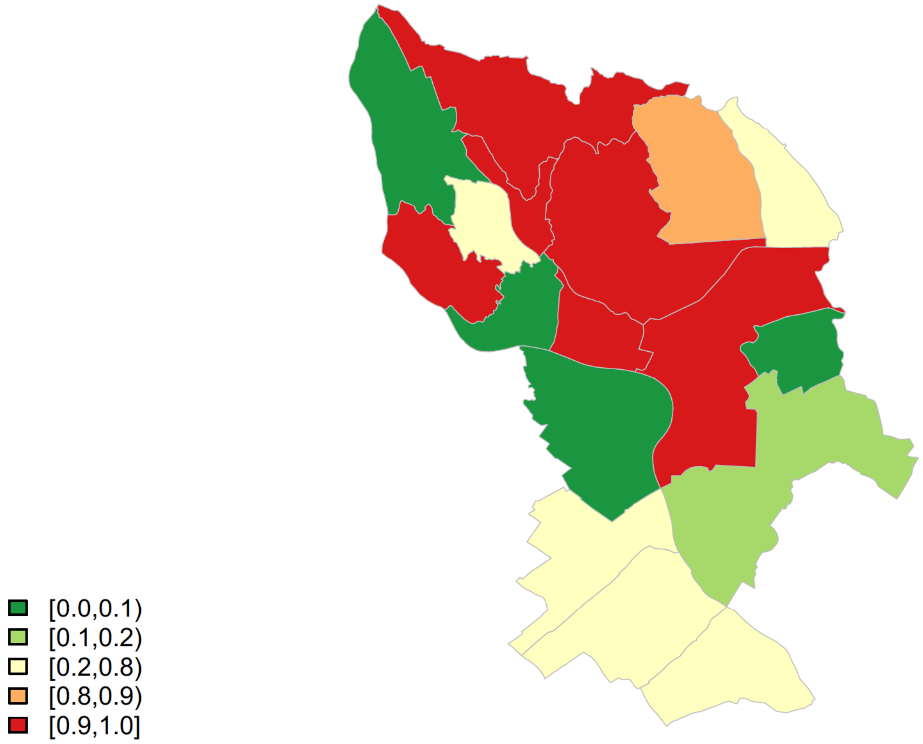

Avoidable mortality, Brussels , Males, 2001−2004
Smoothed Standardised Mortality Ratios (sSMR)

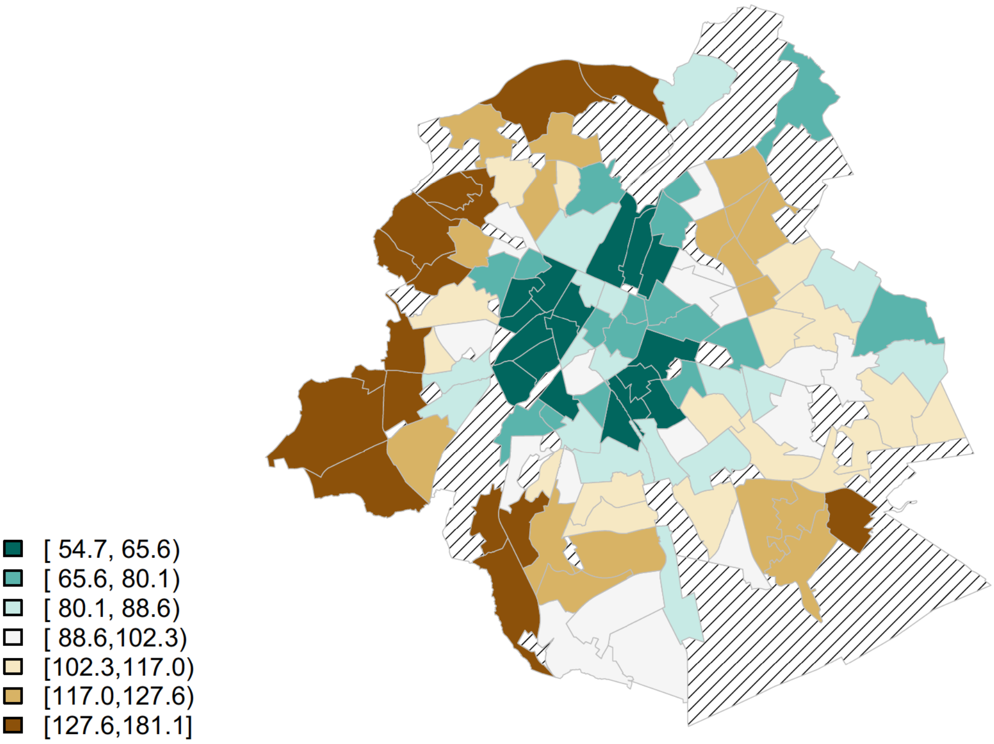

Avoidable mortality, Brussels , Males, 2001−2004
Probability that the sSMR is higher than 100

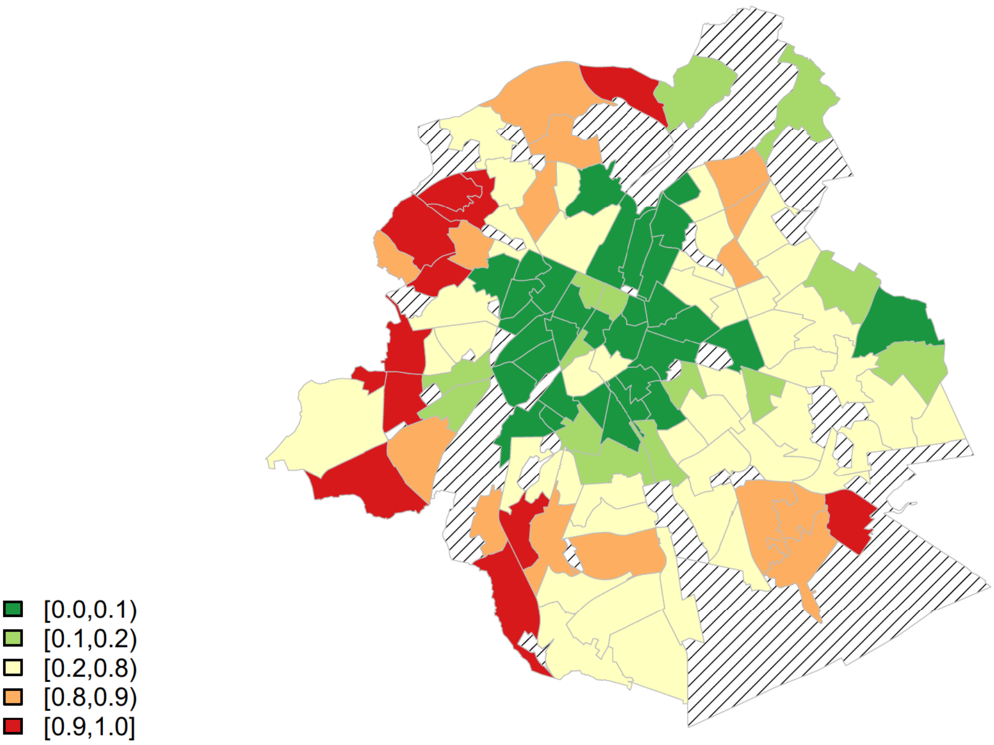

Avoidable mortality, Brussels , Females, 2001−2004
Smoothed Standardised Mortality Ratios (sSMR)

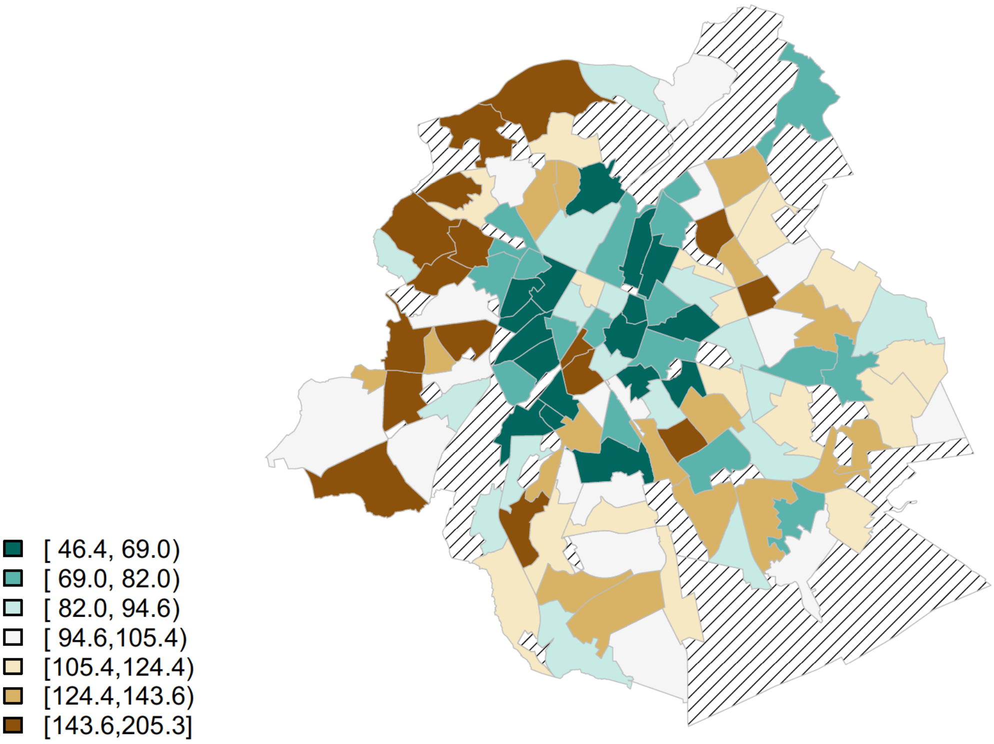

Avoidable mortality, Brussels , Females, 2001−2004
Probability that the sSMR is higher than 100

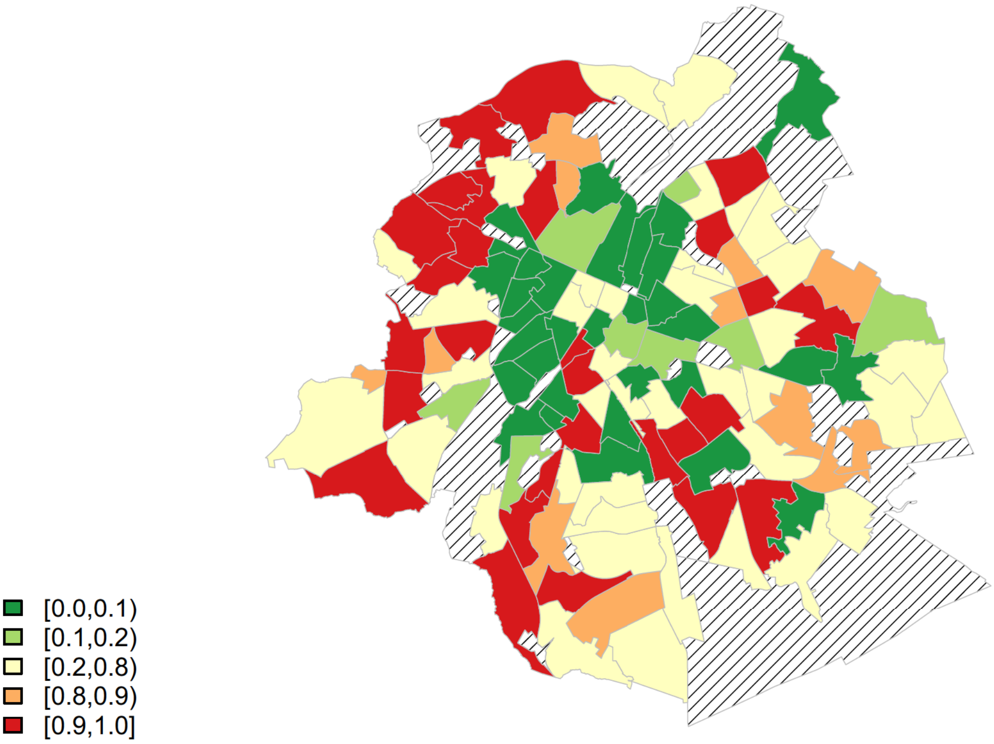

Avoidable mortality, Budapest , Males, 2001−2008
Smoothed Standardised Mortality Ratios (sSMR)

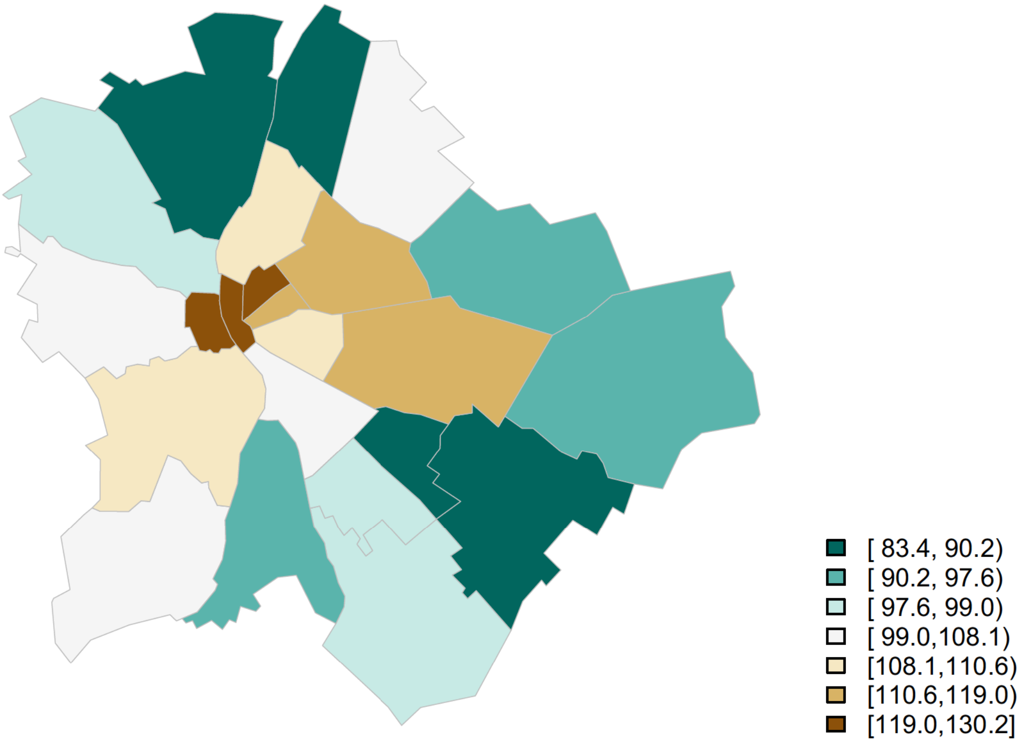

Avoidable mortality, Budapest , Males, 2001−2008
Probability that the sSMR is higher than 100

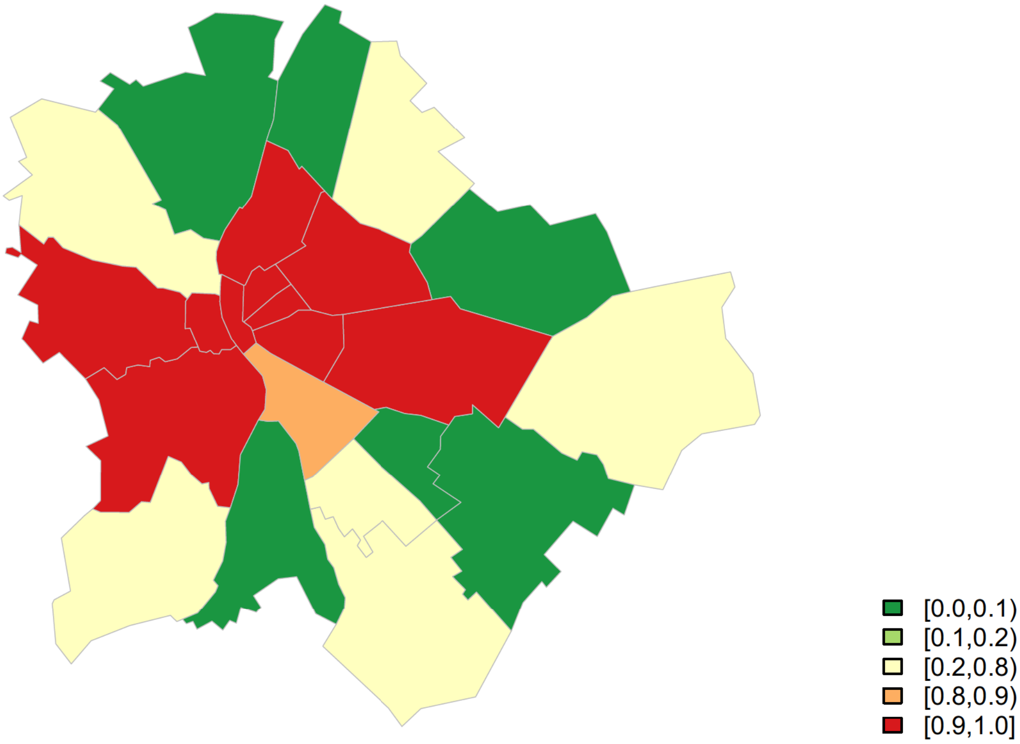

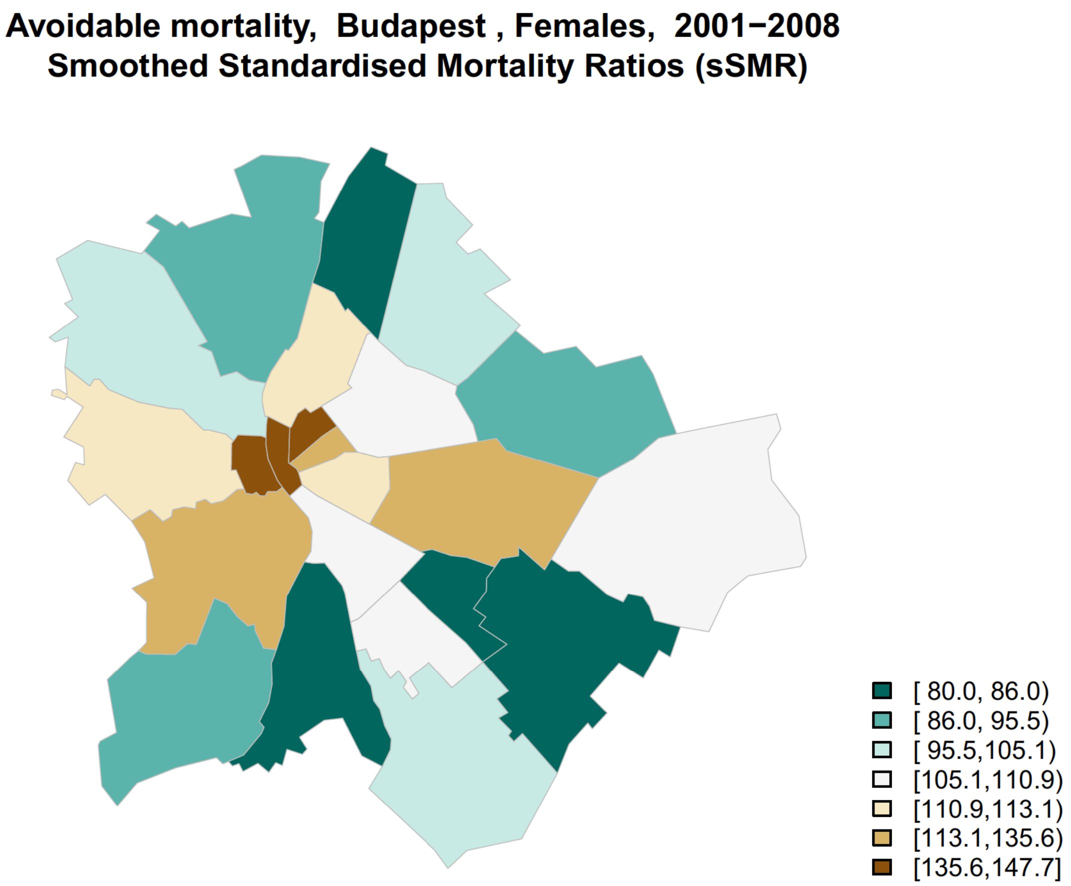
Avoidable mortality, Budapest , Females, 2001−2008
Smoothed Standardised Mortality Ratios (sSMR)
[80.0, 86.0)
[86.0, 95.5)
[95.5,105.1)
[105.1,110.9)
[110.9,113.1)
[113.1,135.6)
[135.6,147.7]

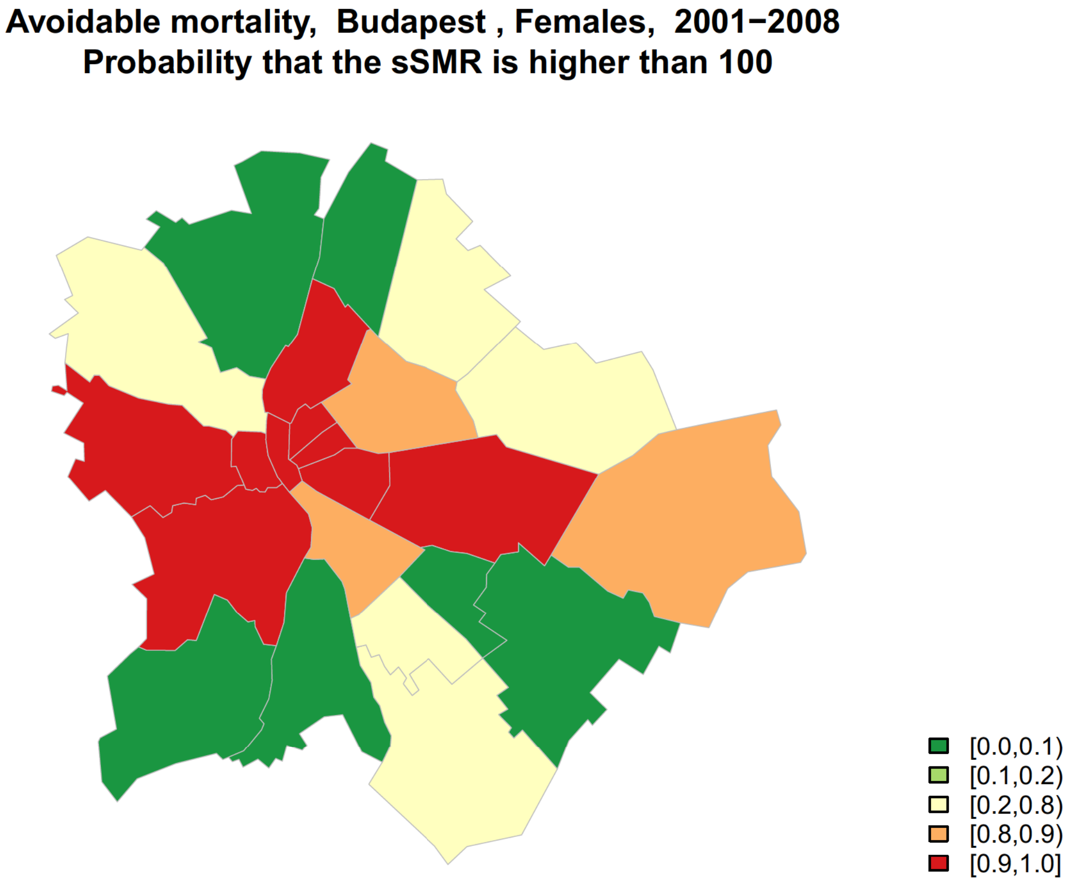
Avoidable mortality, Budapest , Females, 2001−2008
Probability that the sSMR is higher than 100
[0.0,0.1)
[0.1,0.2)
[0.2,0.8)
[0.8,0.9)
[0.9,1.0]

Avoidable mortality, Helsinki , Males, 2000−2009
Smoothed Standardised Mortality Ratios (sSMR)

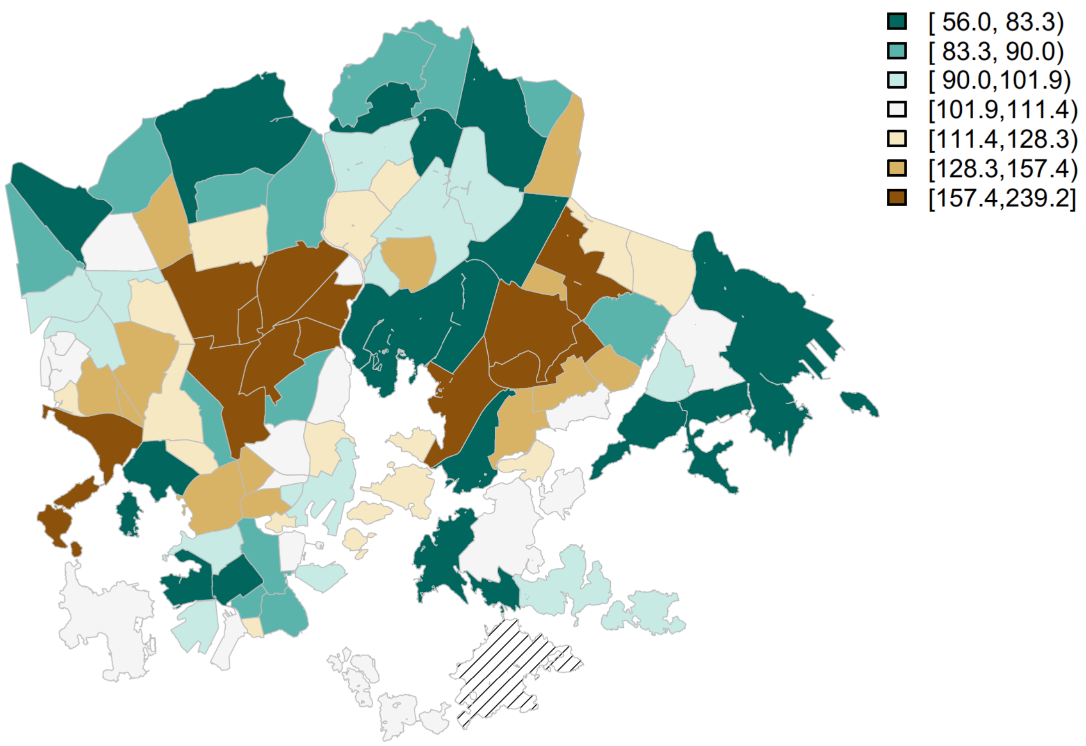

Avoidable mortality, Helsinki , Males, 2000−2009
Probability that the sSMR is higher than 100

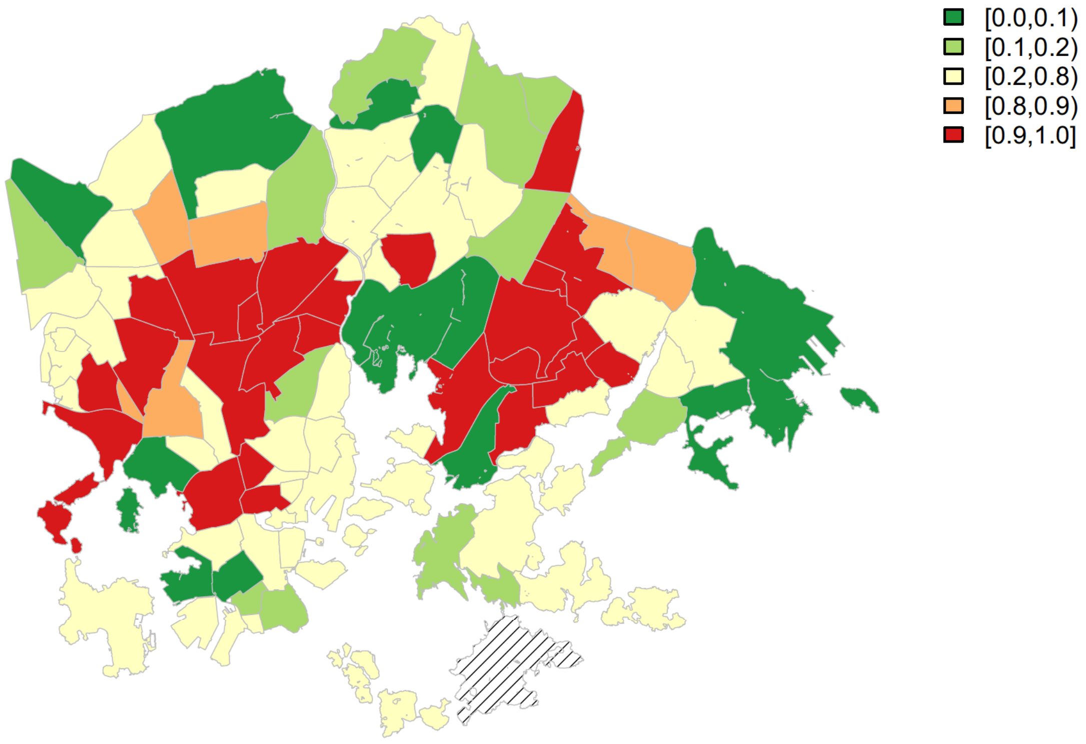

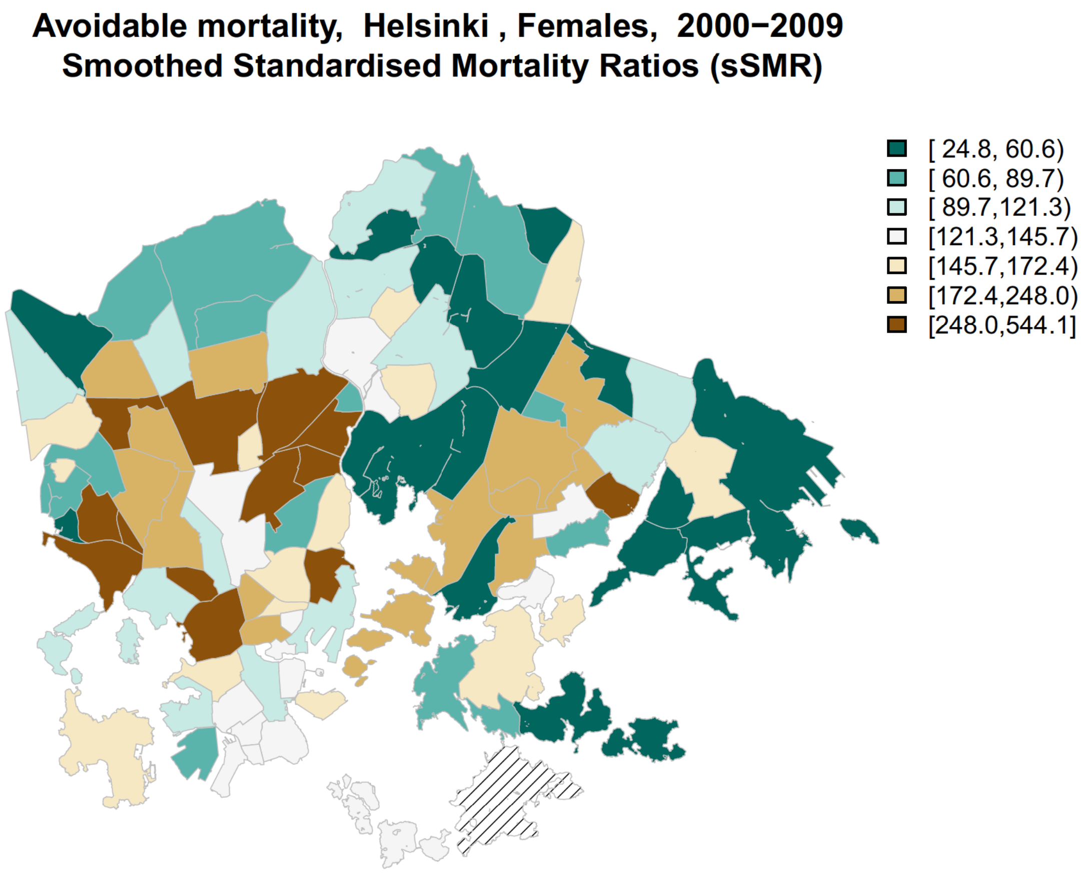
Avoidable mortality, Helsinki , Females, 2000−2009
Smoothed Standardised Mortality Ratios (sSMR)
[24.8, 60.6)
[60.6, 89.7)
[89.7,121.3)
[121.3,145.7)
[145.7,172.4)
[172.4,248.0)
[248.0,544.1]

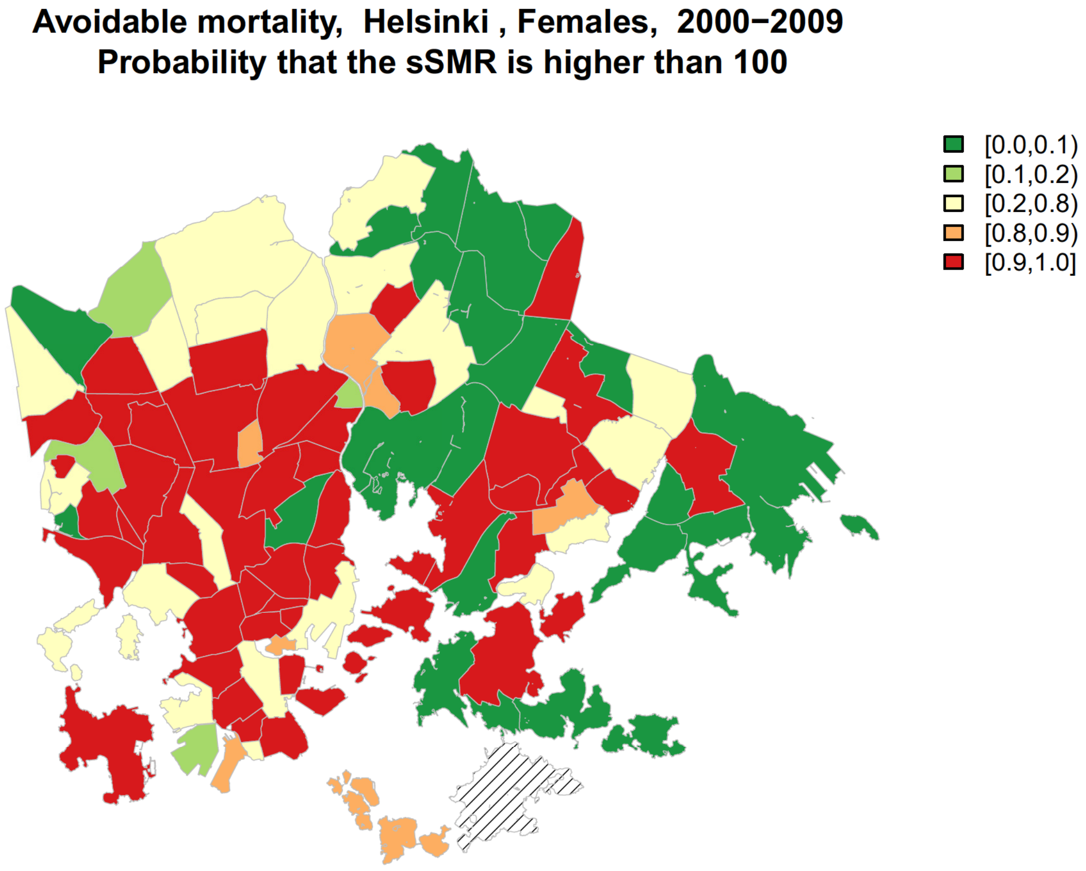
Avoidable mortality, Helsinki , Females, 2000−2009
Probability that the sSMR is higher than 100
[0.0,0.1)
[0.1,0.2)
[0.2,0.8)
[0.8,0.9)
[0.9,1.0]

Avoidable mortality, Kosice , Males, 1996−2008
Smoothed Standardised Mortality Ratios (sSMR)

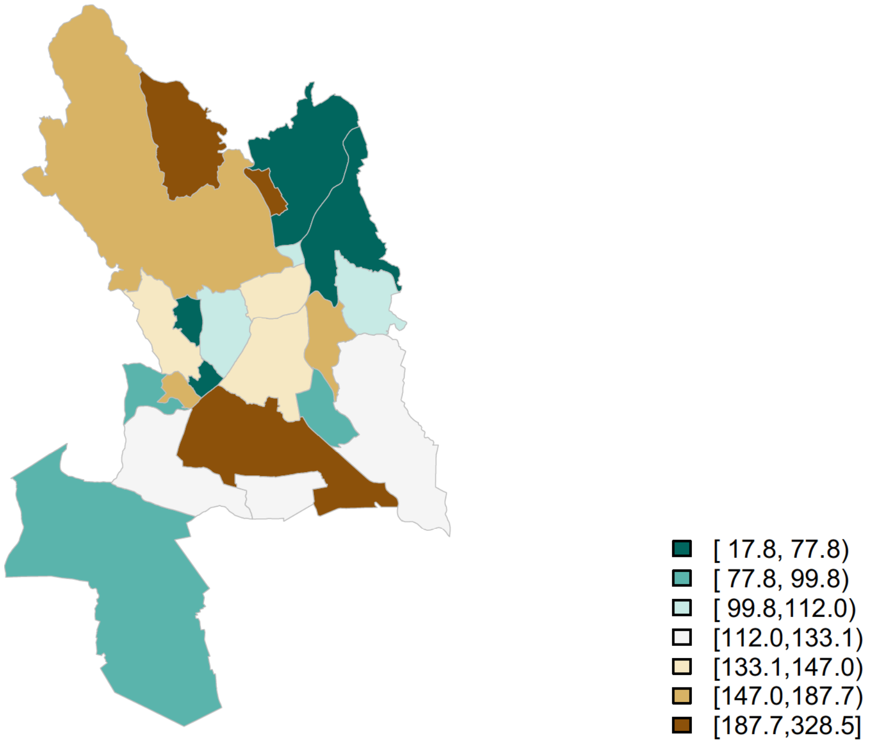

Avoidable mortality, Kosice , Males, 1996−2008
Probability that the sSMR is higher than 100

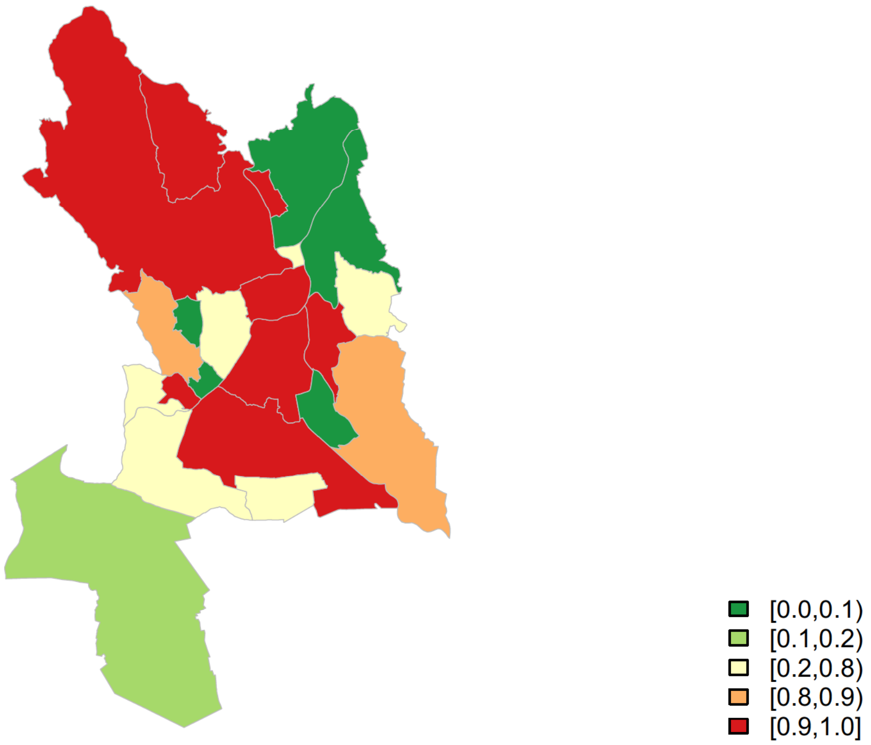

Avoidable mortality, Kosice , Females, 1996−2008
Smoothed Standardised Mortality Ratios (sSMR)

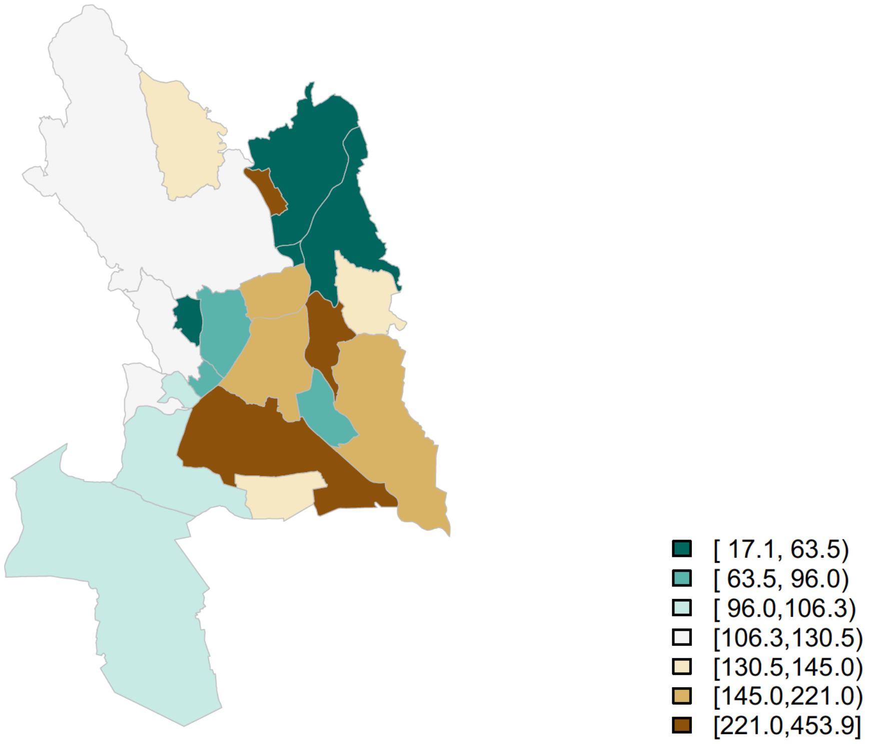

Avoidable mortality, Kosice , Females, 1996−2008
Probability that the sSMR is higher than 100

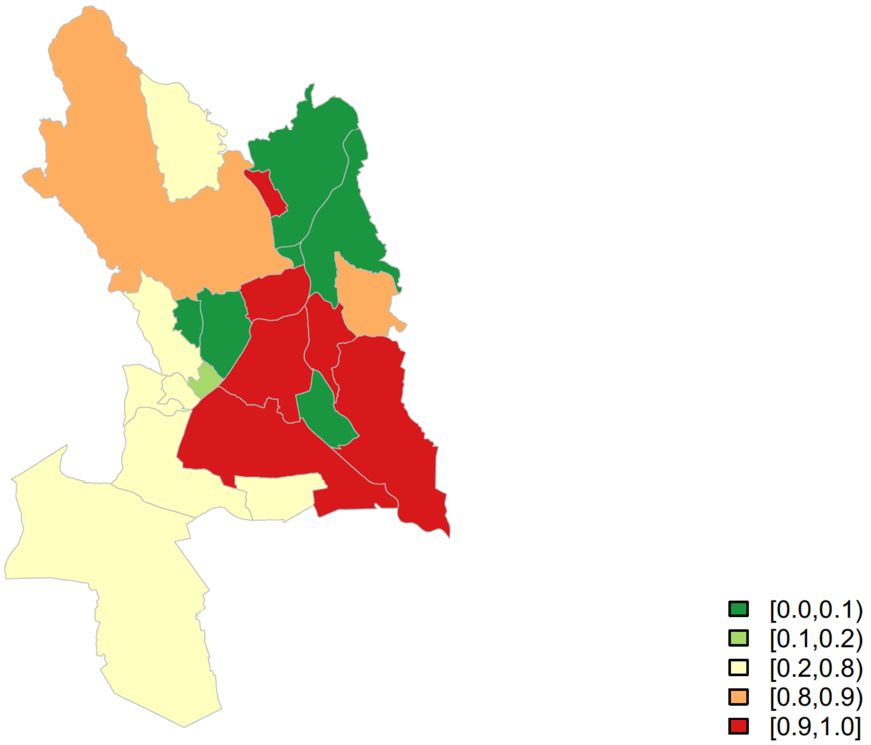

Avoidable mortality, Lisbon , Males, 1995−2008
Smoothed Standardised Mortality Ratios (sSMR)

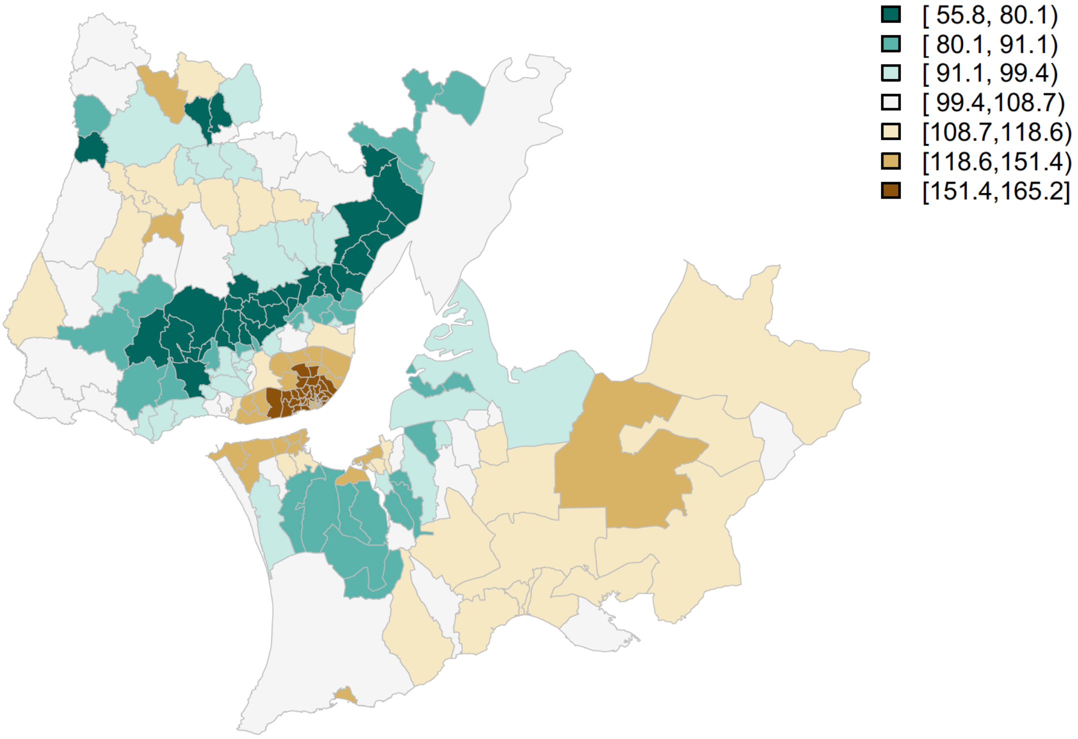

Avoidable mortality, Lisbon , Males, 1995−2008
Probability that the sSMR is higher than 100

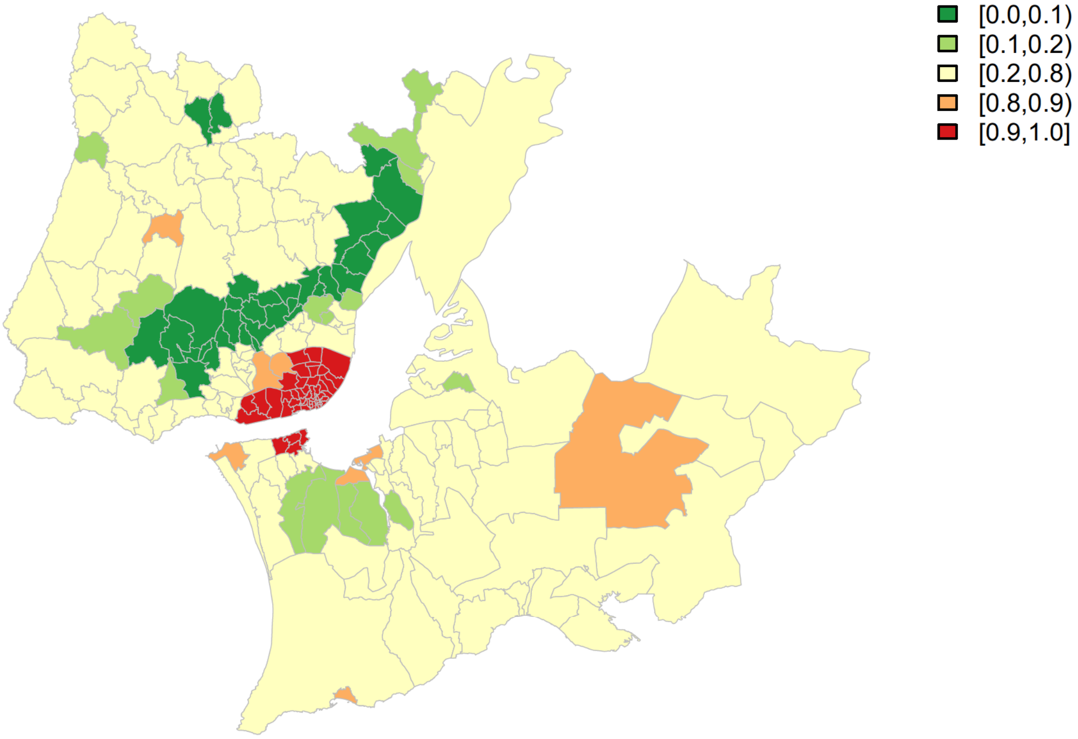

Avoidable mortality, Lisbon , Females, 1995−2008
Smoothed Standardised Mortality Ratios (sSMR)

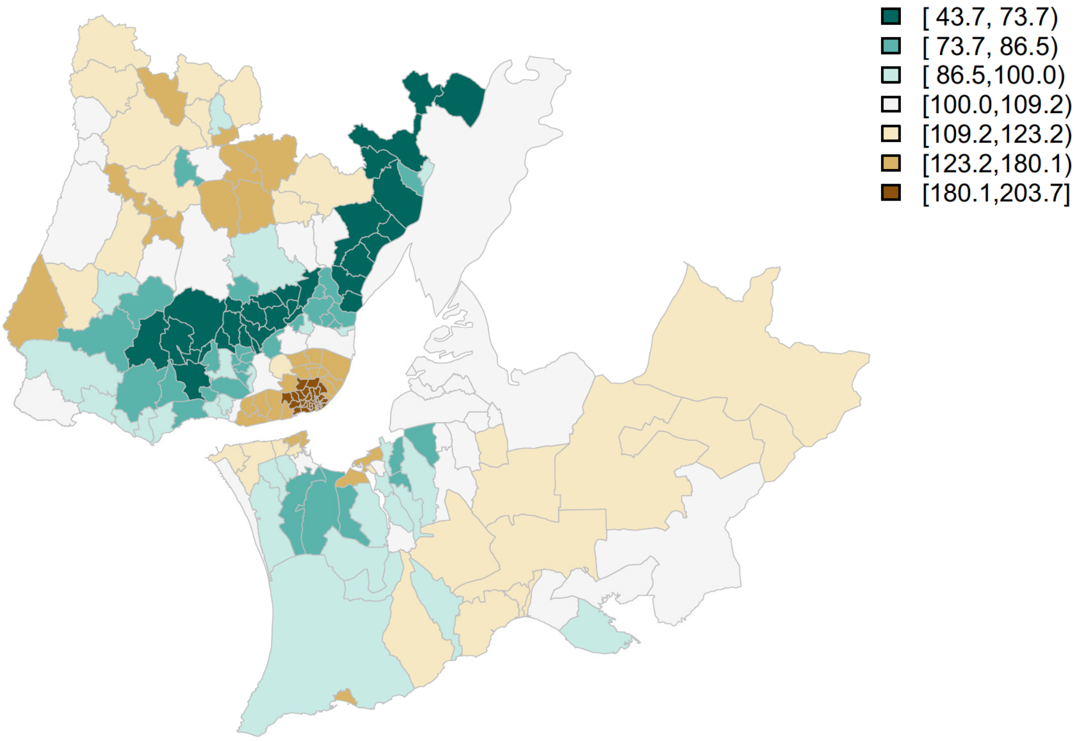

Avoidable mortality, Lisbon , Females, 1995−2008
Probability that the sSMR is higher than 100

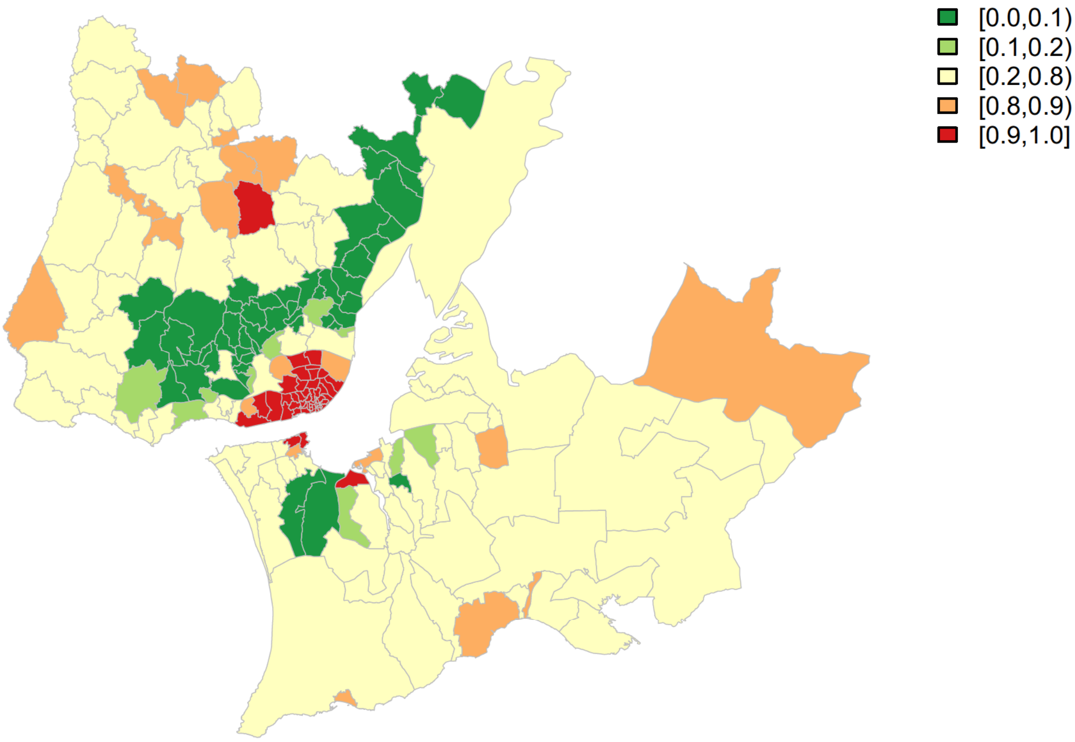

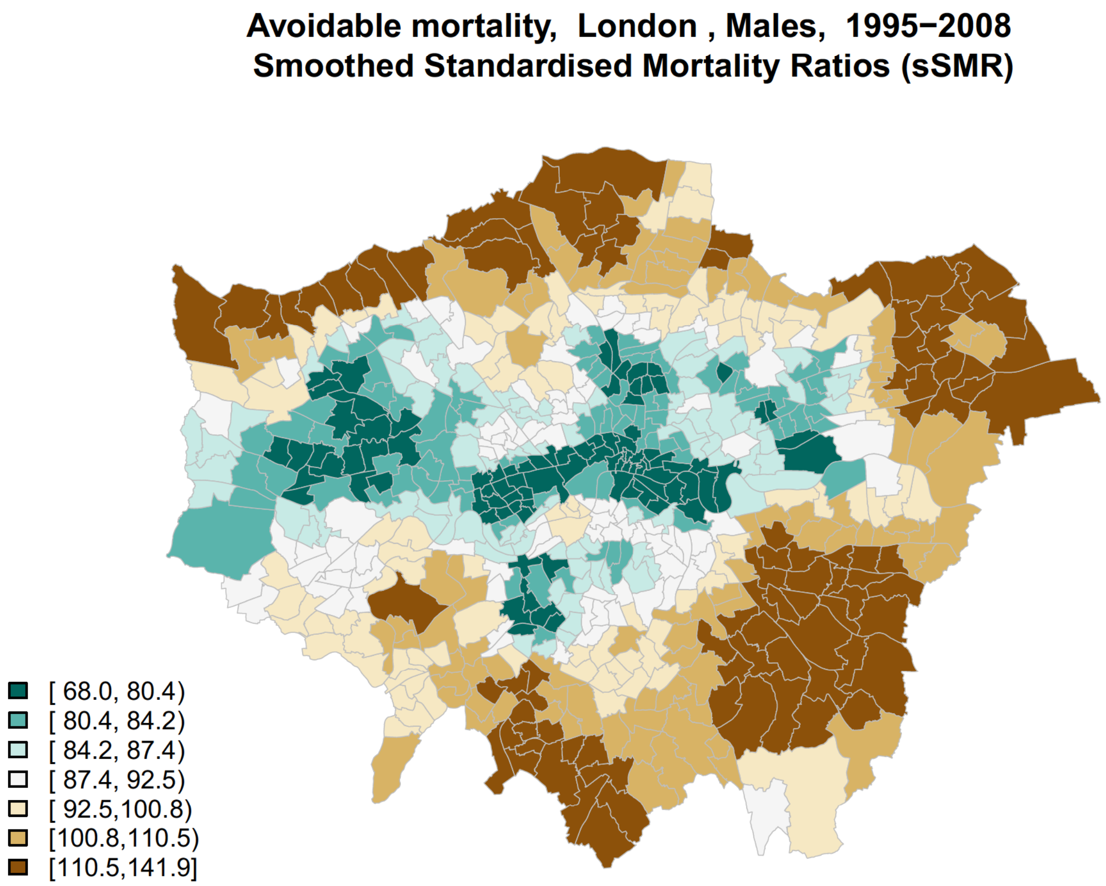
Avoidable mortality, London , Males, 1995−2008
Smoothed Standardised Mortality Ratios (sSMR)
[68.0, 80.4)
[80.4, 84.2)
[84.2, 87.4)
[87.4, 92.5)
[92.5,100.8)
[100.8,110.5)
[110.5,141.9]

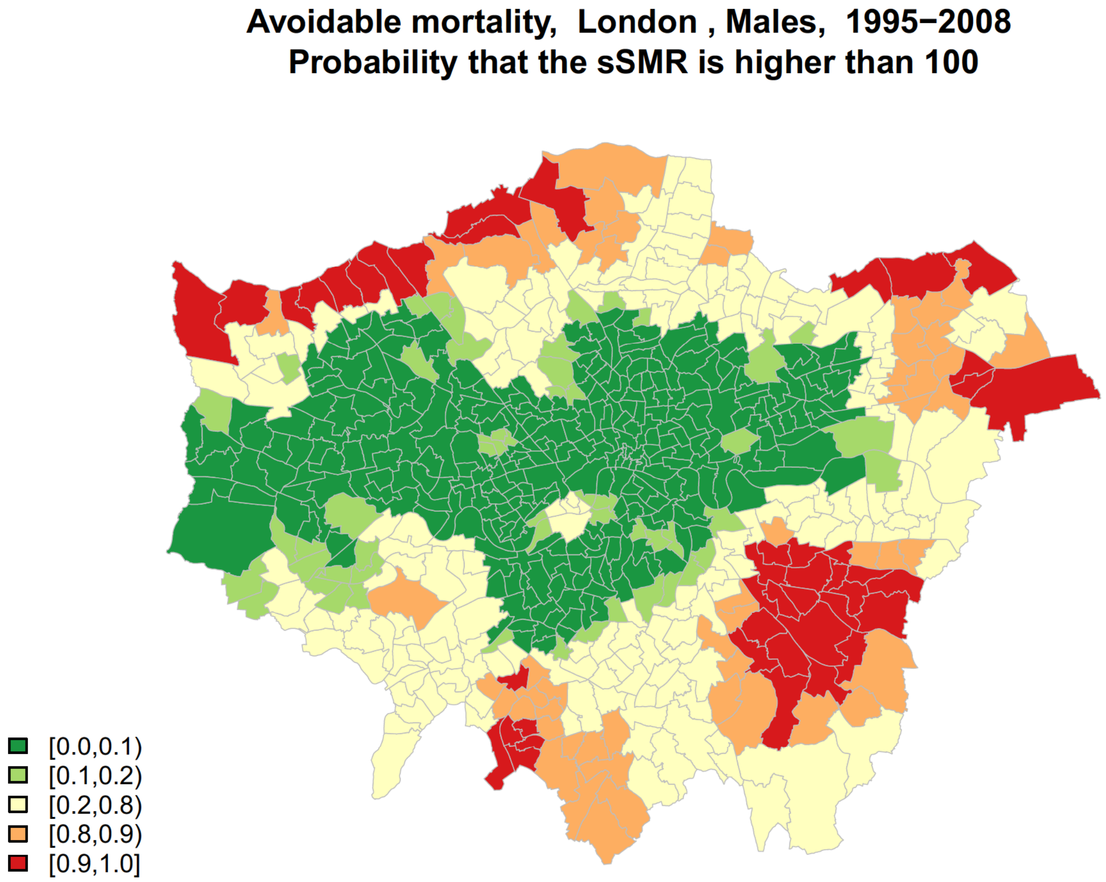
Avoidable mortality, London , Males, 1995−2008
Probability that the sSMR is higher than 100
[0.0,0.1)
[0.1,0.2)
[0.2,0.8)
[0.8,0.9)
[0.9,1.0]

Avoidable mortality, London , Females, 1995−2008
Smoothed Standardised Mortality Ratios (sSMR)

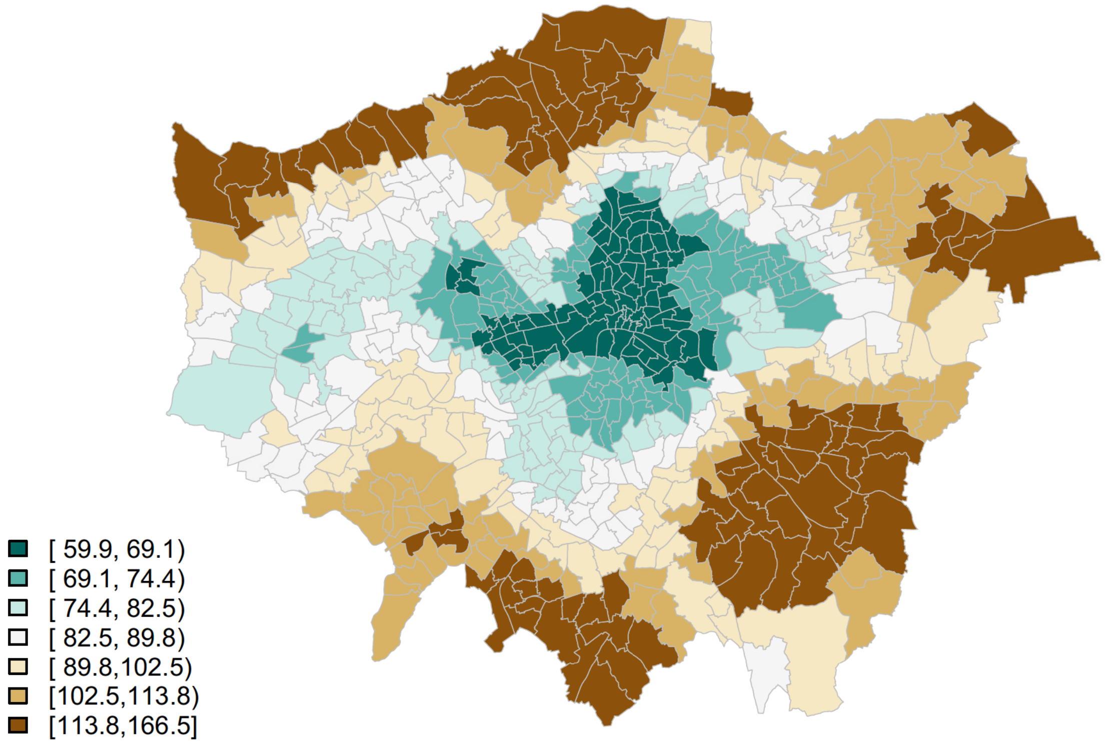

Avoidable mortality, London , Females, 1995−2008
Probability that the sSMR is higher than 100

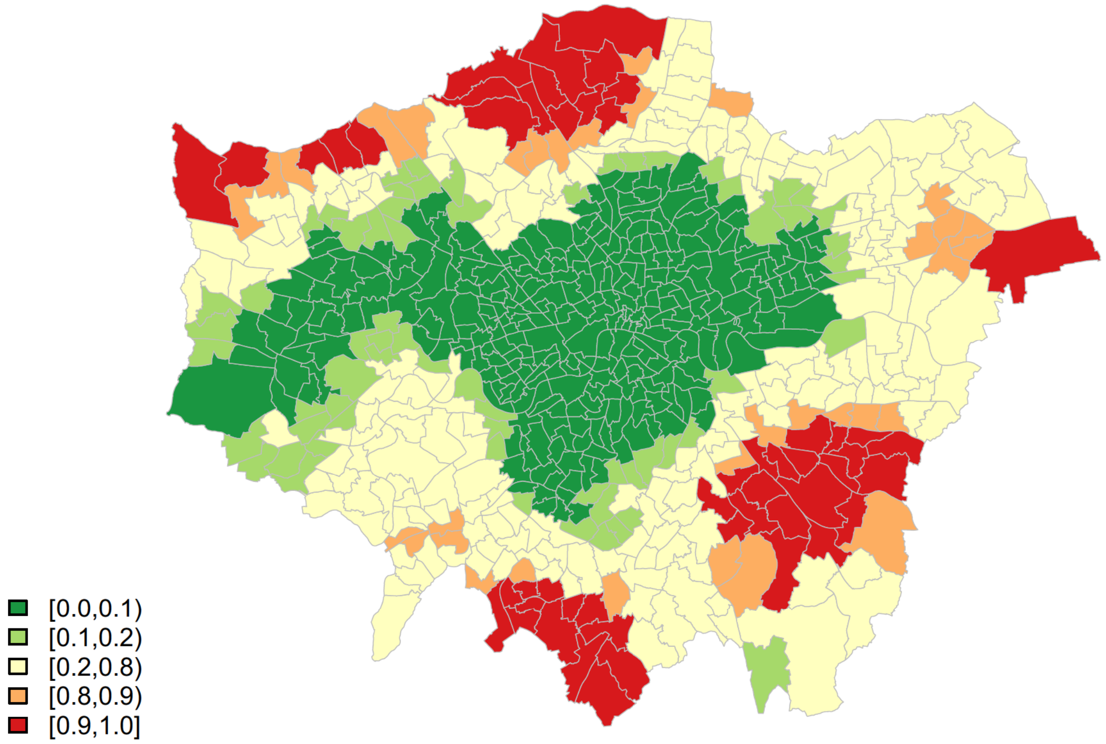

Avoidable mortality, Madrid , Males, 1995−2007
Smoothed Standardised Mortality Ratios (sSMR)

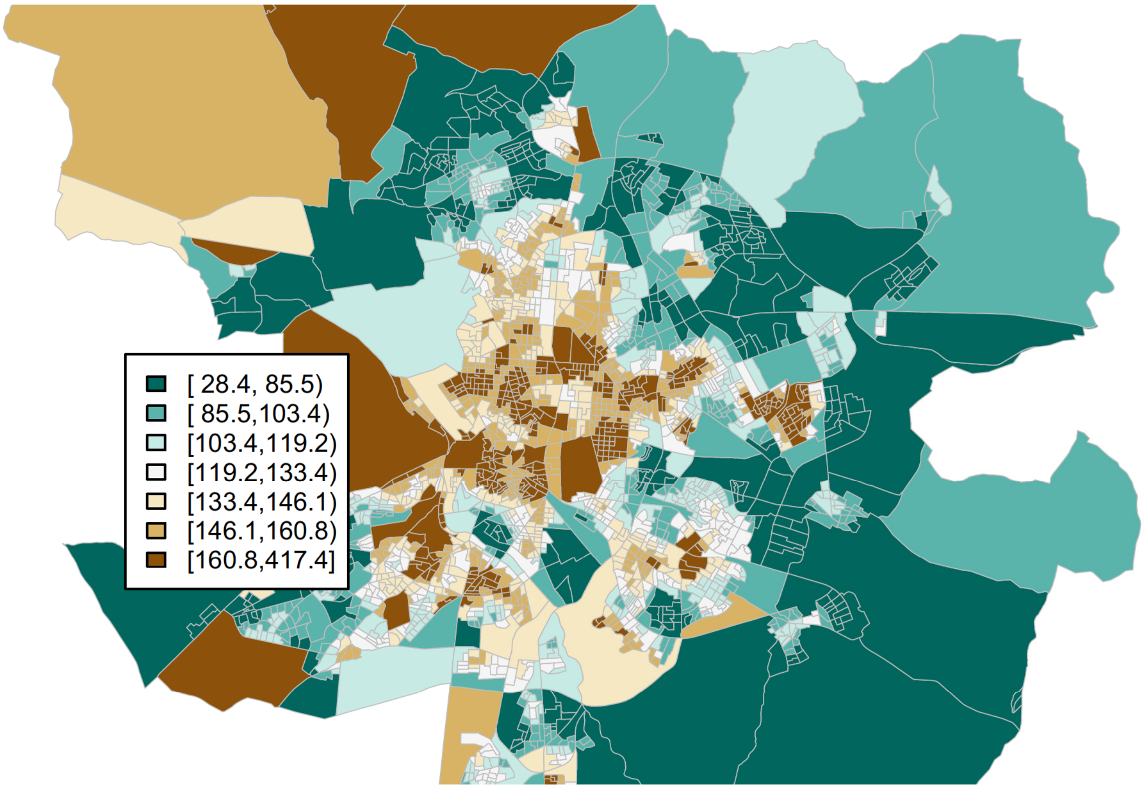

Avoidable mortality, Madrid , Males, 1995−2007
Probability that the sSMR is higher than 100

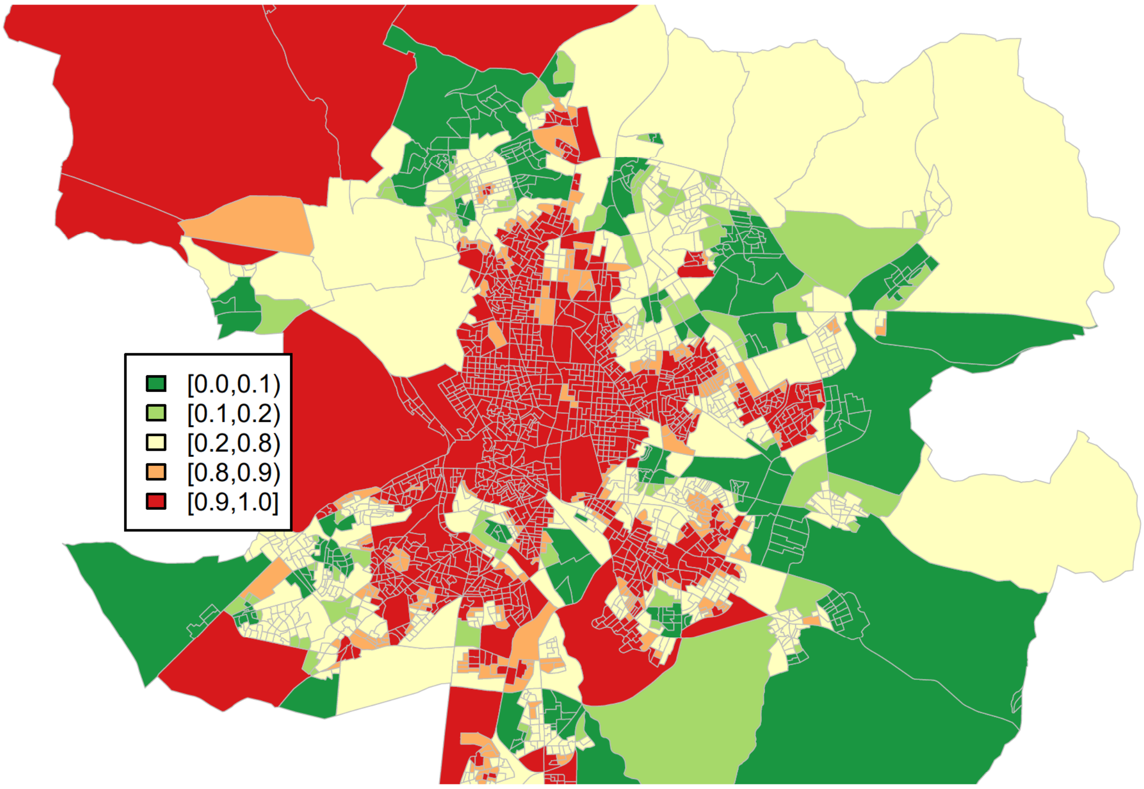

Avoidable mortality, Madrid , Females, 1995−2007
Smoothed Standardised Mortality Ratios (sSMR)

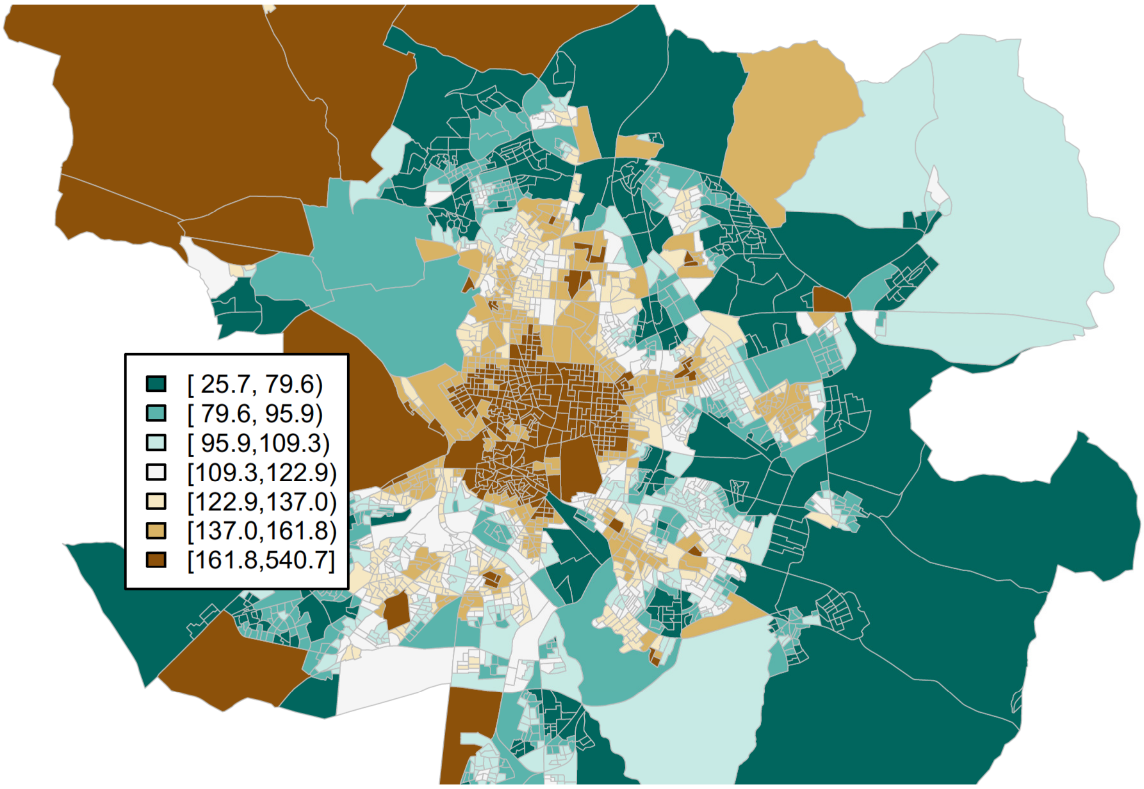

Avoidable mortality, Madrid , Females, 1995−2007
Probability that the sSMR is higher than 100

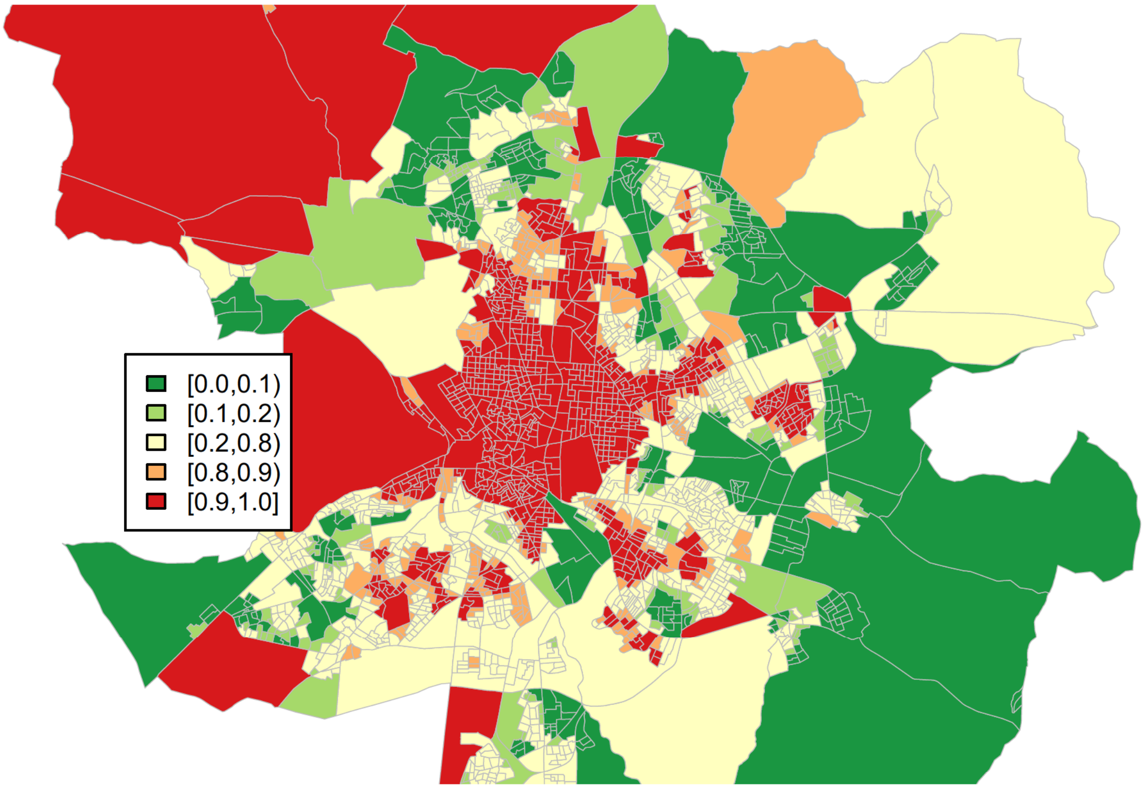

Avoidable mortality, Prague , Males, 2003−2007
Smoothed Standardised Mortality Ratios (sSMR)

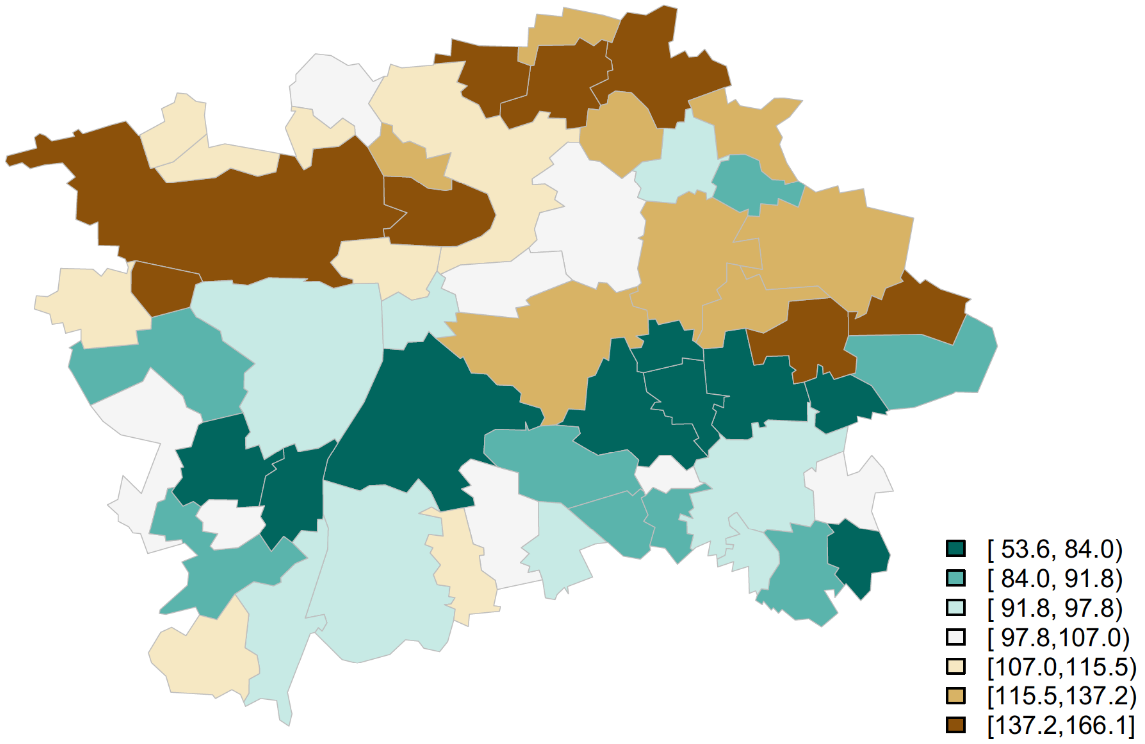

Avoidable mortality, Prague , Males, 2003−2007
Probability that the sSMR is higher than 100

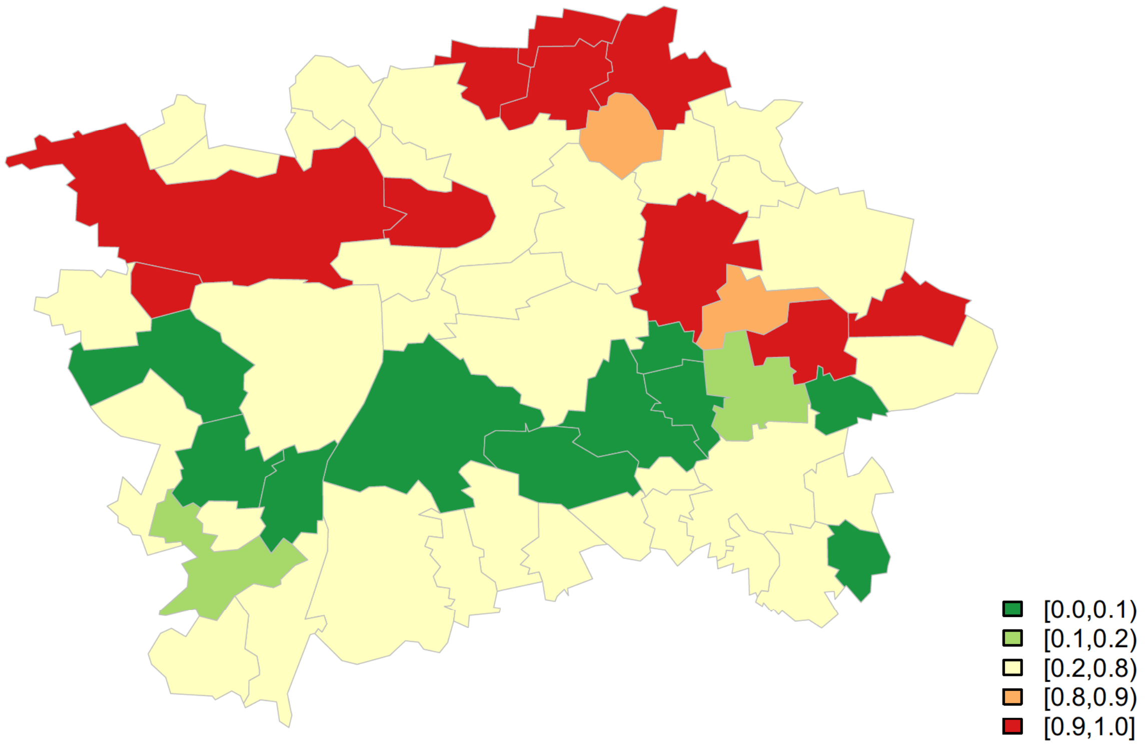

Avoidable mortality, Prague , Females, 2003−2007
Smoothed Standardised Mortality Ratios (sSMR)

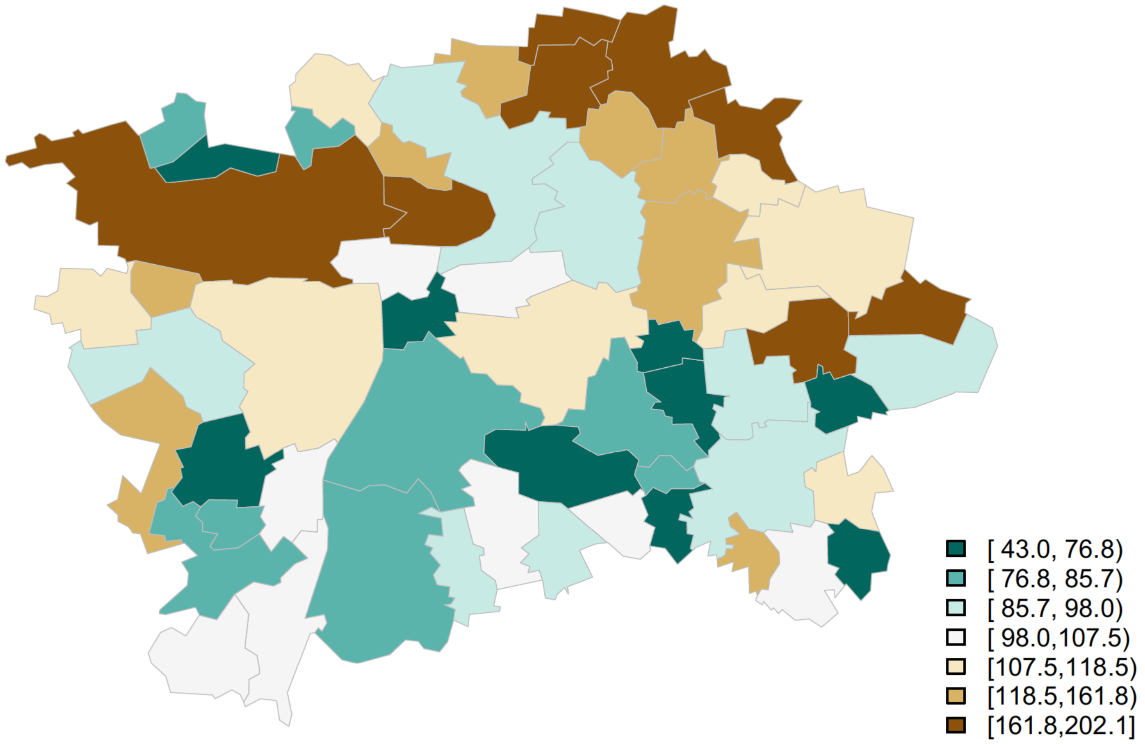

Avoidable mortality, Prague , Females, 2003−2007
Probability that the sSMR is higher than 100

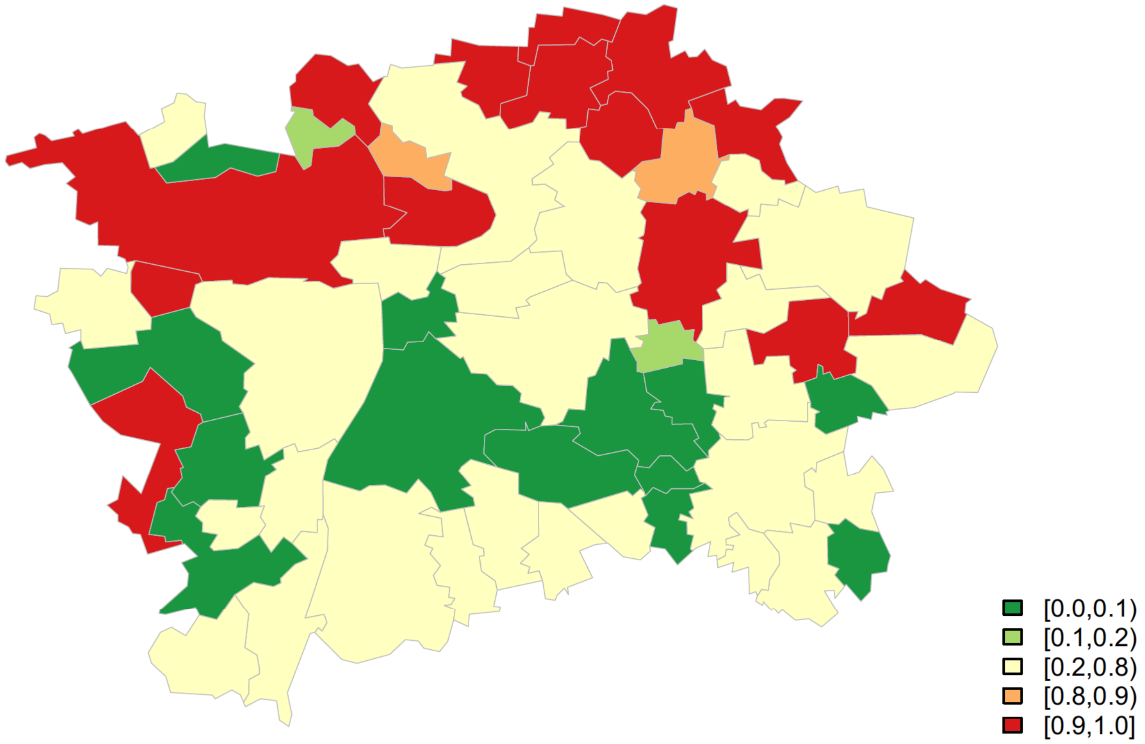

Avoidable mortality, Rotterdam , Males, 1996−2008
Smoothed Standardised Mortality Ratios (sSMR)

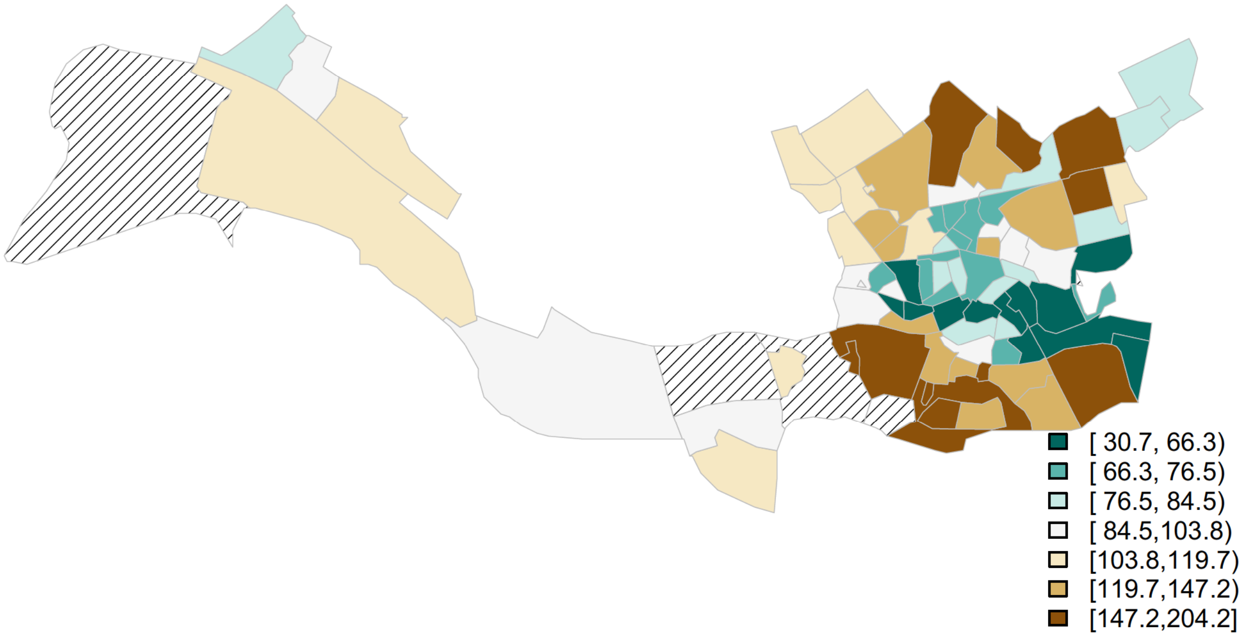

Avoidable mortality, Rotterdam , Males, 1996−2008
Probability that the sSMR is higher than 100

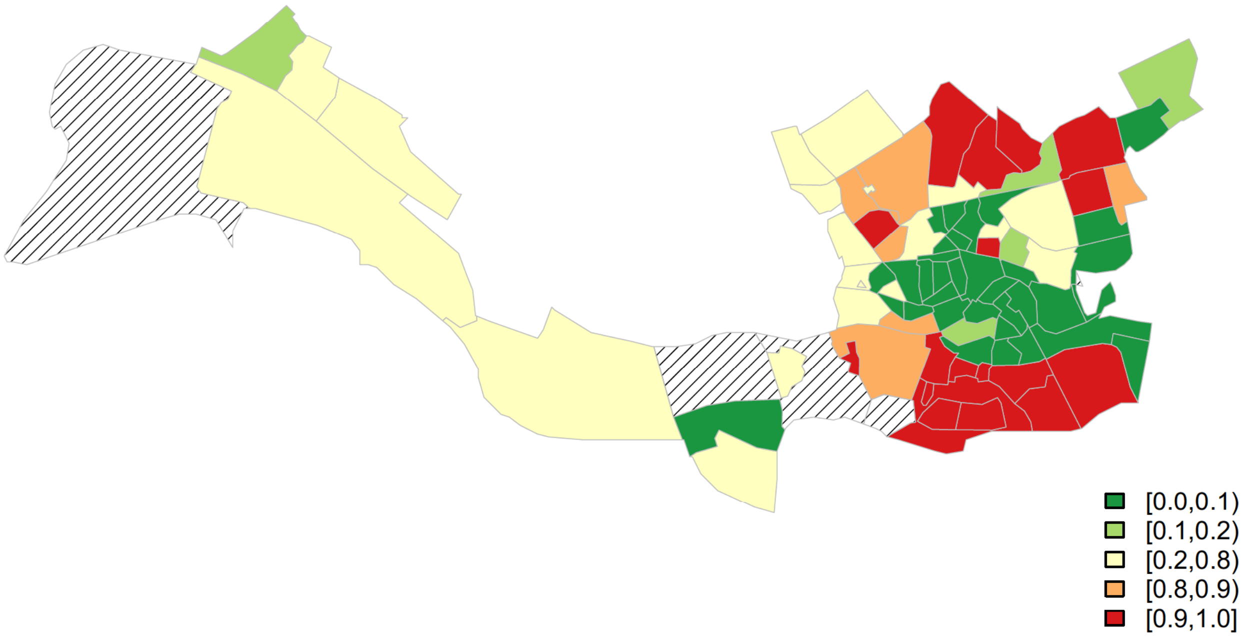

Avoidable mortality, Rotterdam , Females, 1996−2008
Smoothed Standardised Mortality Ratios (sSMR)

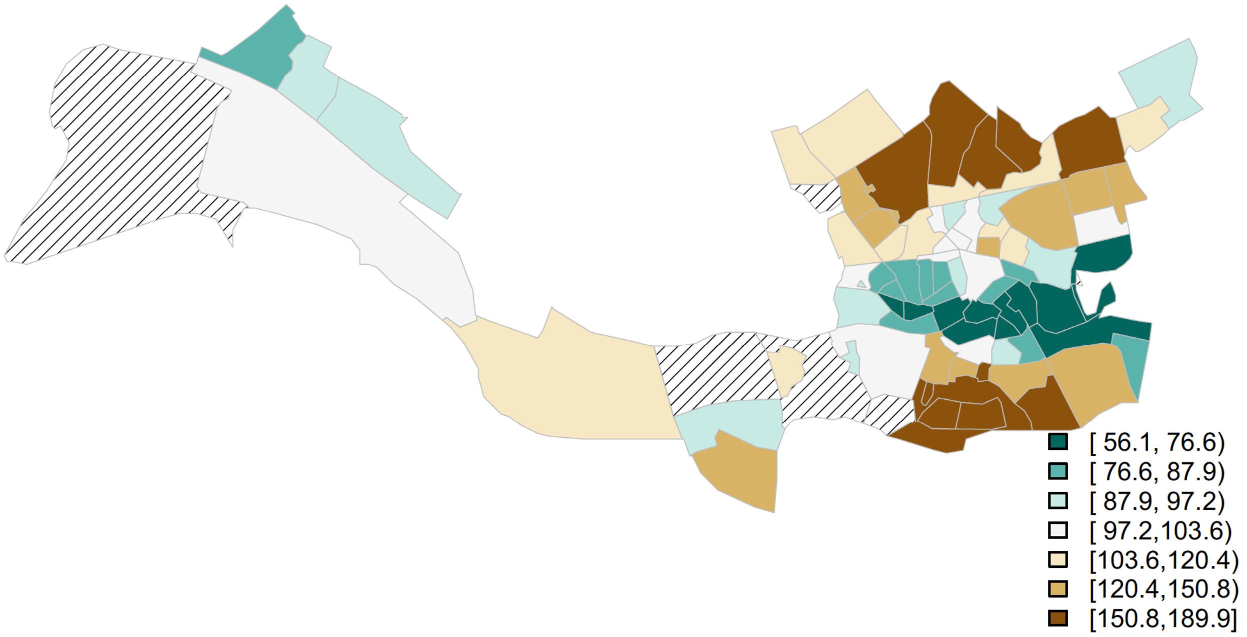

Avoidable mortality, Rotterdam , Females, 1996−2008
Probability that the sSMR is higher than 100

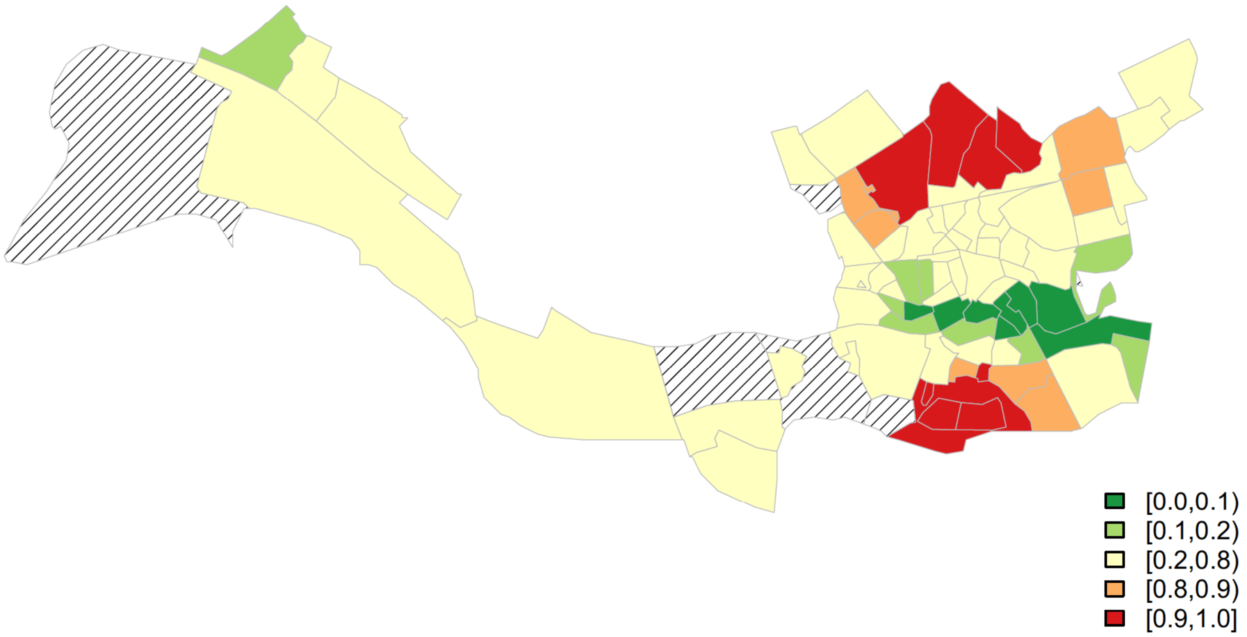

Avoidable mortality, Stockholm , Males, 2000−2007
Smoothed Standardised Mortality Ratios (sSMR)

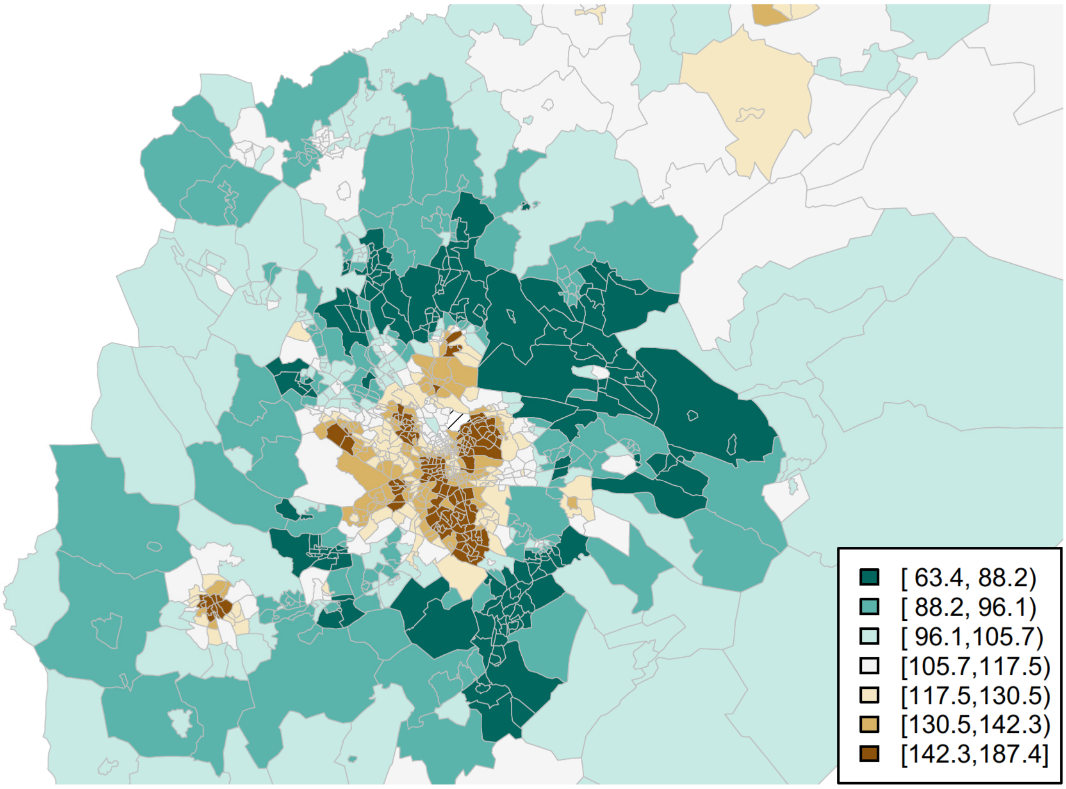

Avoidable mortality, Stockholm , Males, 2000−2007
Probability that the sSMR is higher than 100

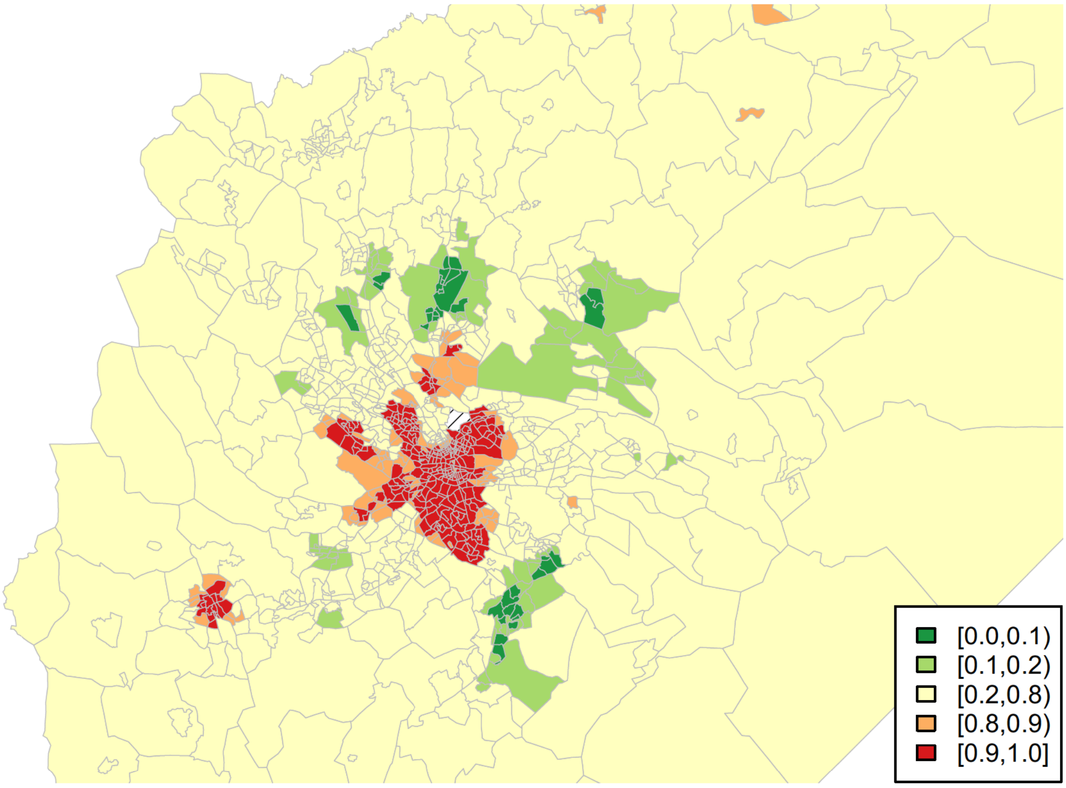

Avoidable mortality, Stockholm , Females, 2000−2007
Smoothed Standardised Mortality Ratios (sSMR)

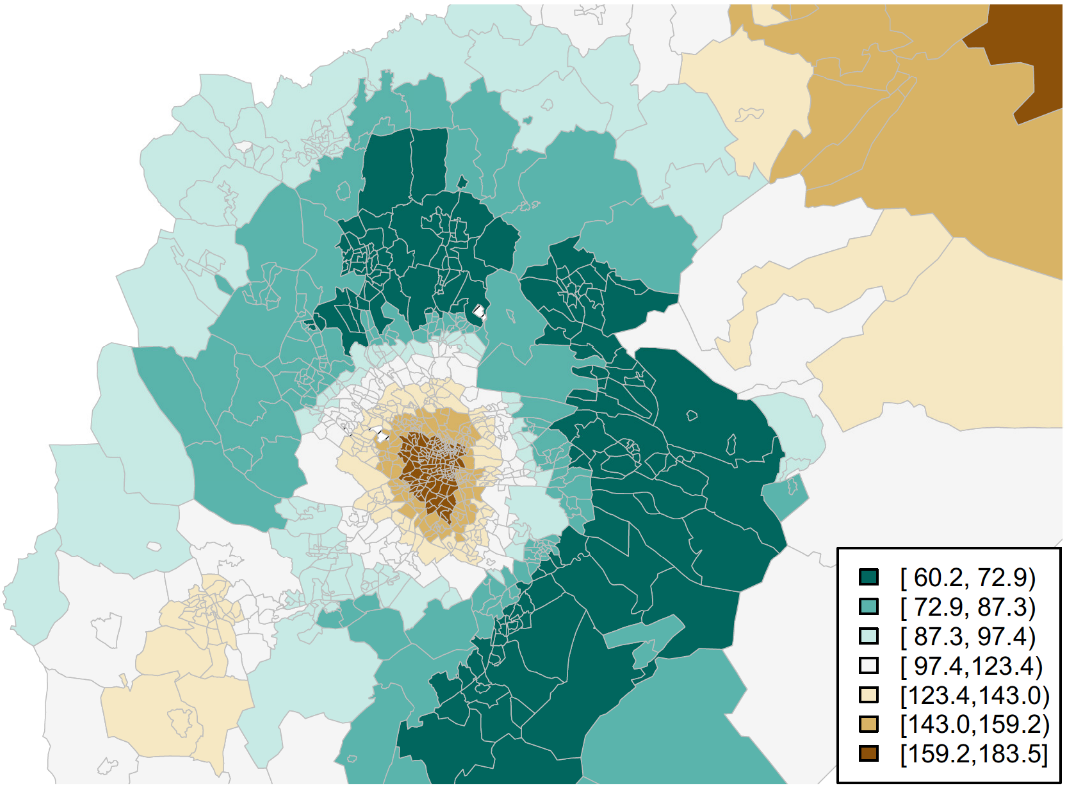

Avoidable mortality, Stockholm , Females, 2000−2007
Probability that the sSMR is higher than 100

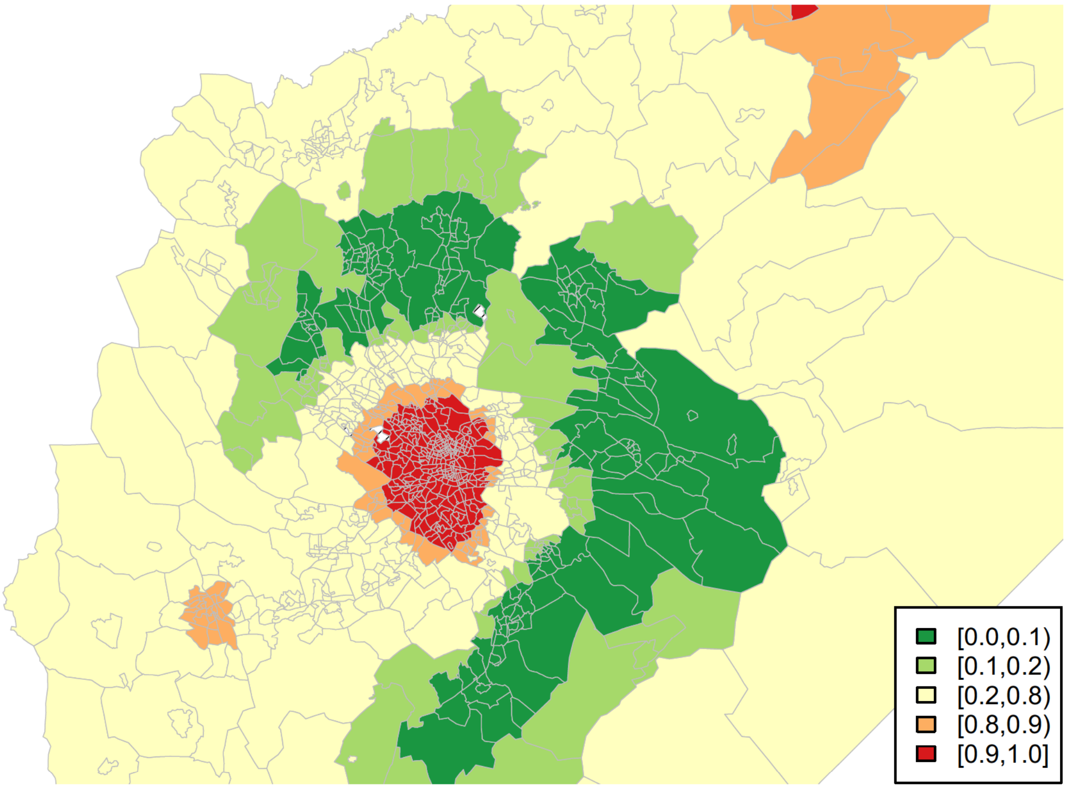

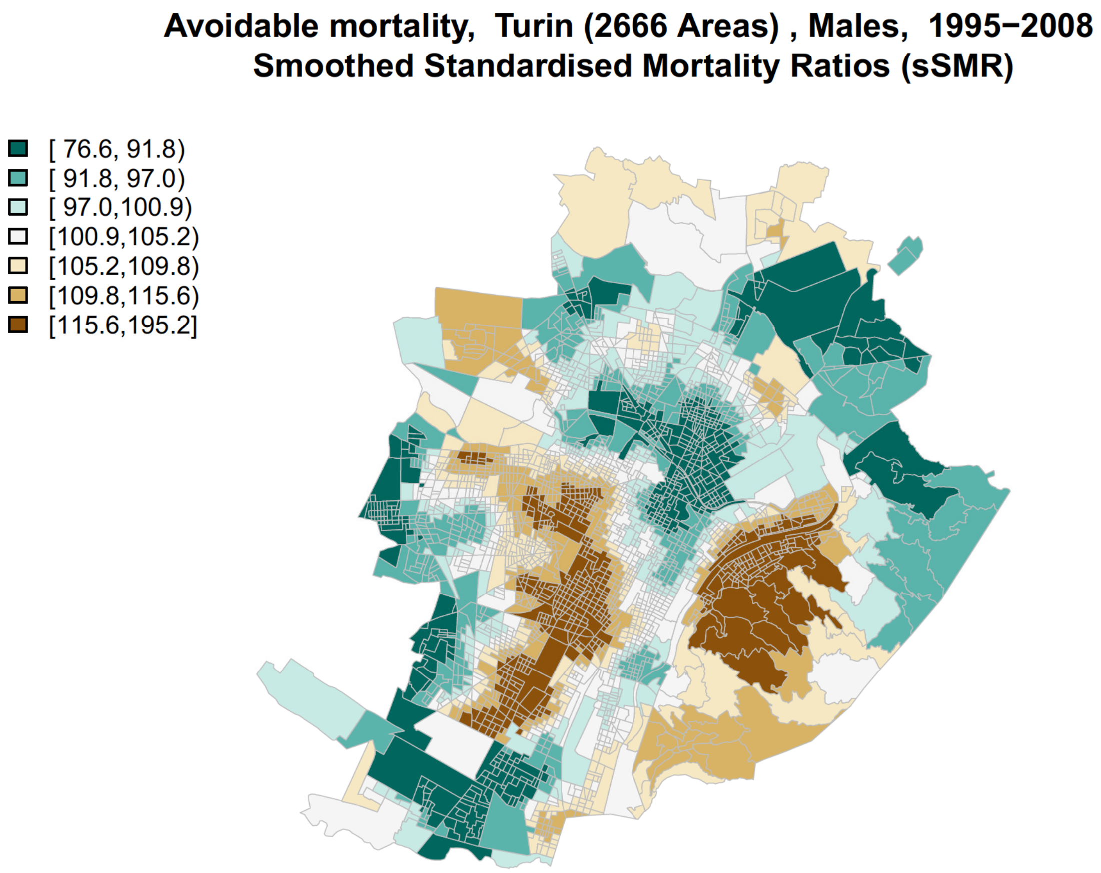
Avoidable mortality, Turin (2666 Areas) , Males, 1995−2008
Smoothed Standardised Mortality Ratios (sSMR)
[76.6, 91.8)
[91.8, 97.0)
[97.0,100.9)
[100.9,105.2)
[105.2,109.8)
[109.8,115.6)
[115.6,195.2]

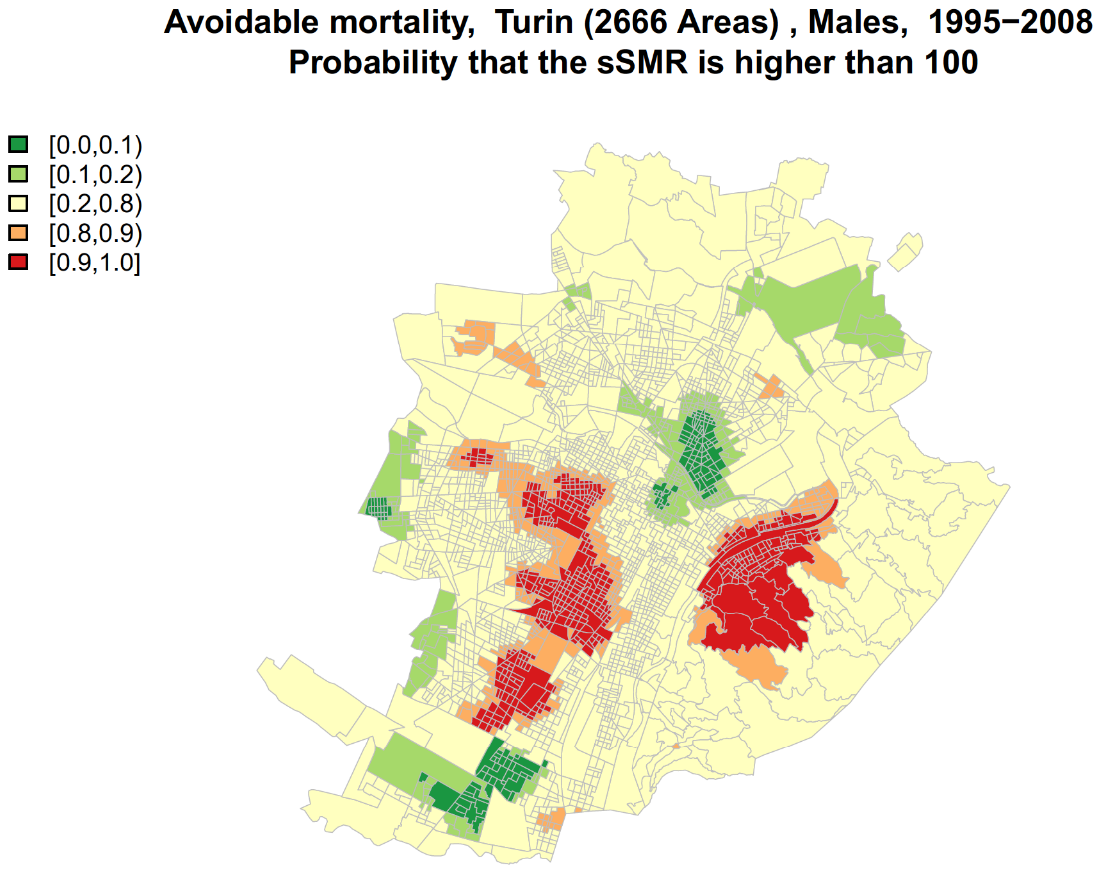
Avoidable mortality, Turin (2666 Areas) , Males, 1995−2008
Probability that the sSMR is higher than 100
[0.0,0.1)
[0.1,0.2)
[0.2,0.8)
[0.8,0.9)
[0.9,1.0]

Avoidable mortality, Turin (2666 Areas) , Females, 1995−2008
Smoothed Standardised Mortality Ratios (sSMR)

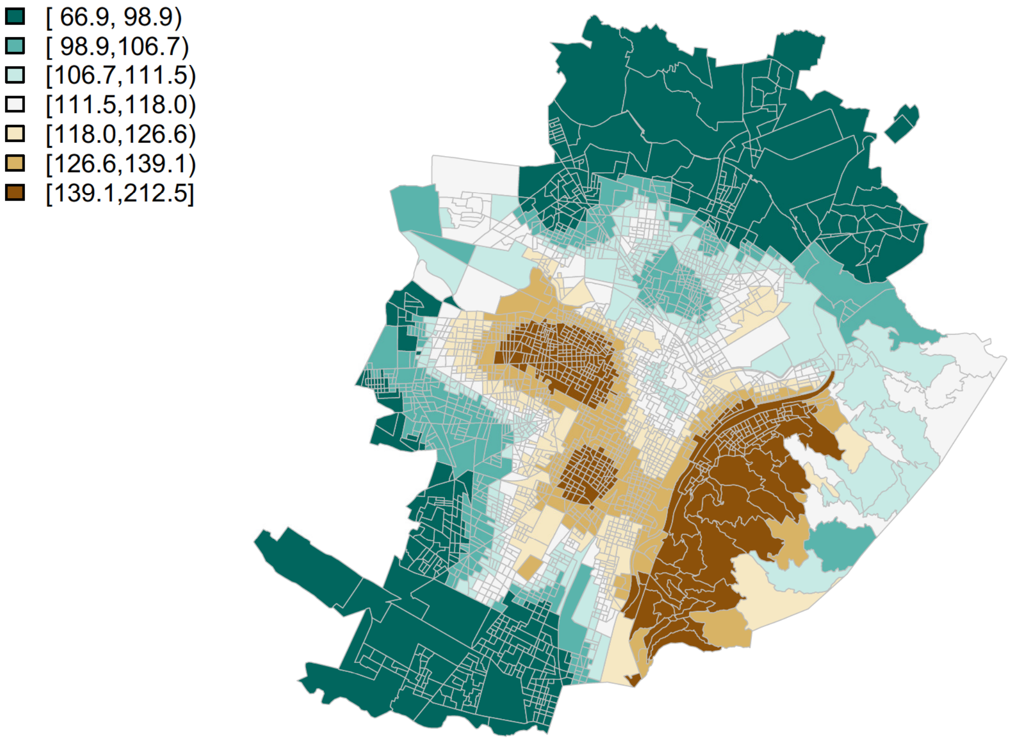

Avoidable mortality, Turin (2666 Areas) , Females, 1995−2008
Probability that the sSMR is higher than 100

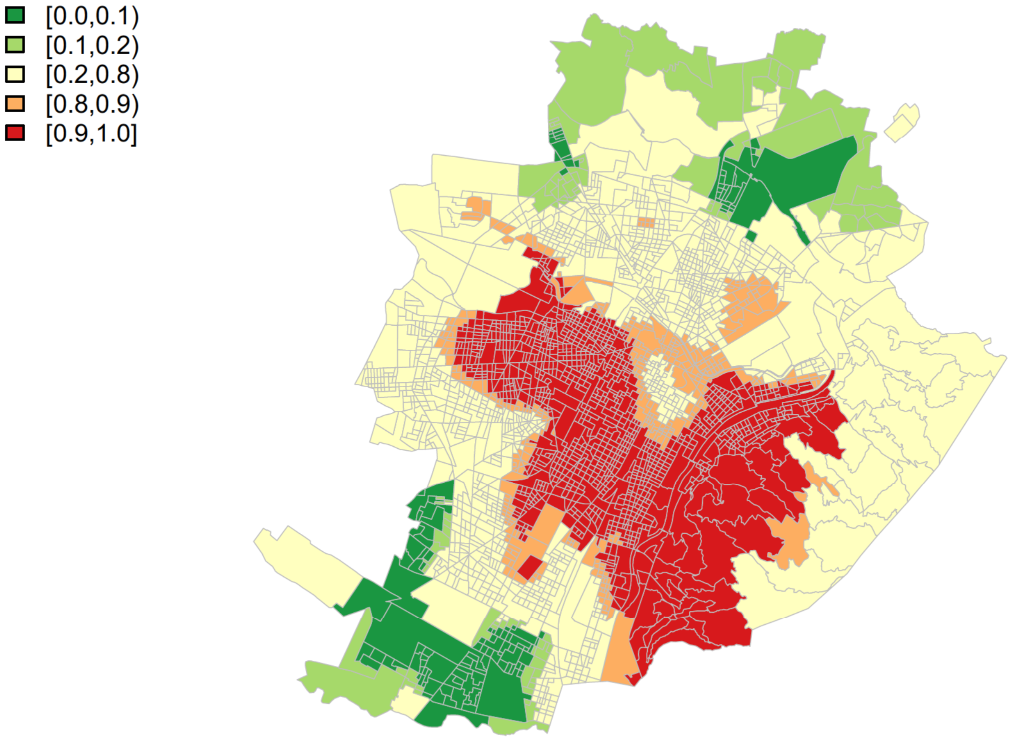

Avoidable mortality, Turin (94 Areas) , Males, 1995−2008
Smoothed Standardised Mortality Ratios (sSMR)

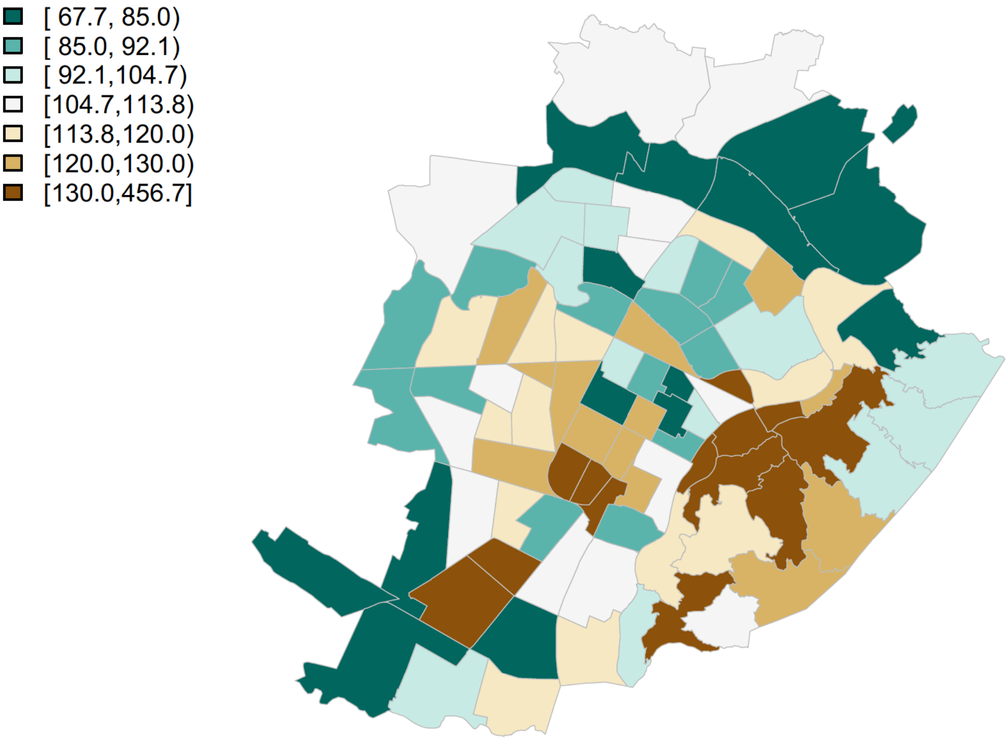

Avoidable mortality, Turin (94 Areas) , Males, 1995−2008
Probability that the sSMR is higher than 100

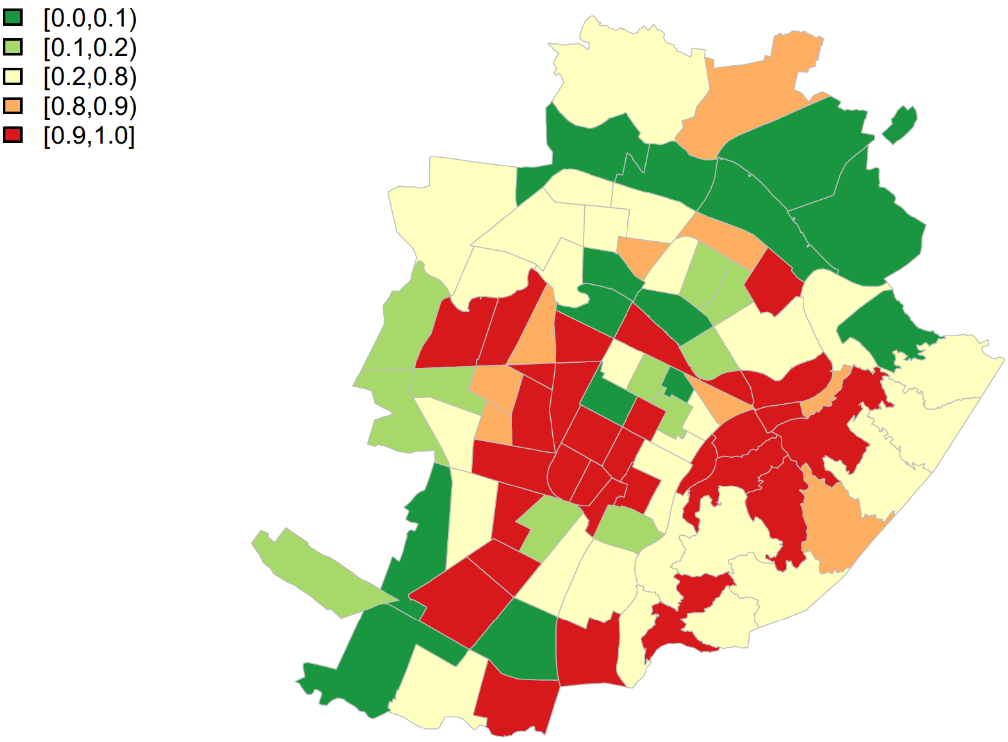

Avoidable mortality, Turin (94 Areas) , Females, 1995−2008
Smoothed Standardised Mortality Ratios (sSMR)

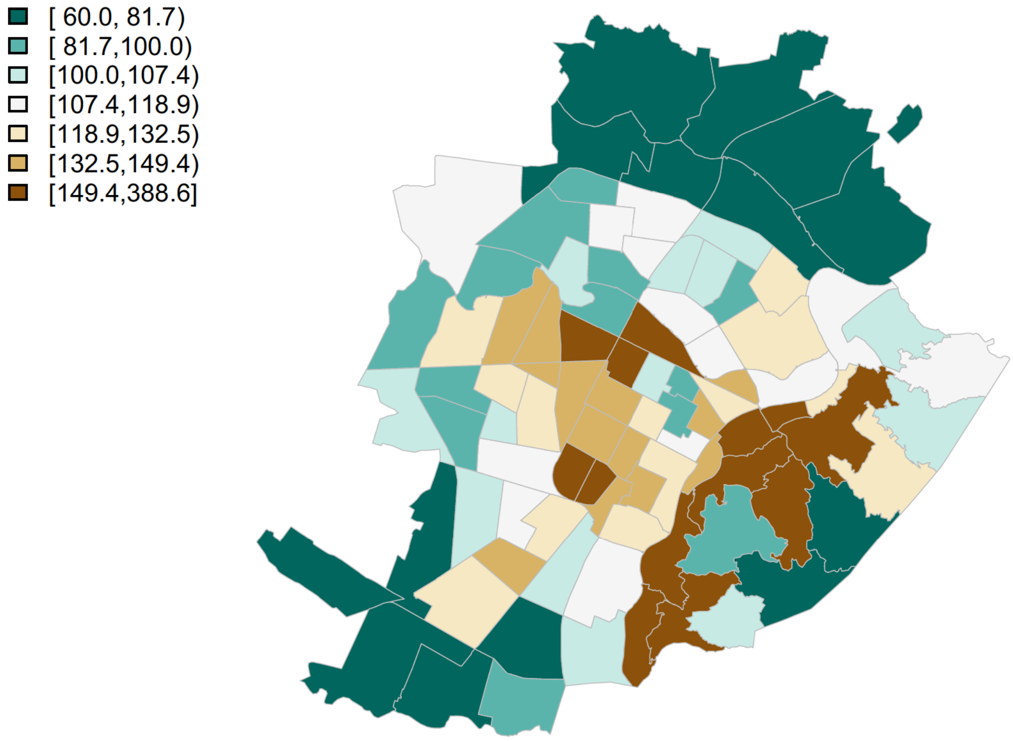

Avoidable mortality, Turin (94 Areas) , Females, 1995−2008
Probability that the sSMR is higher than 100

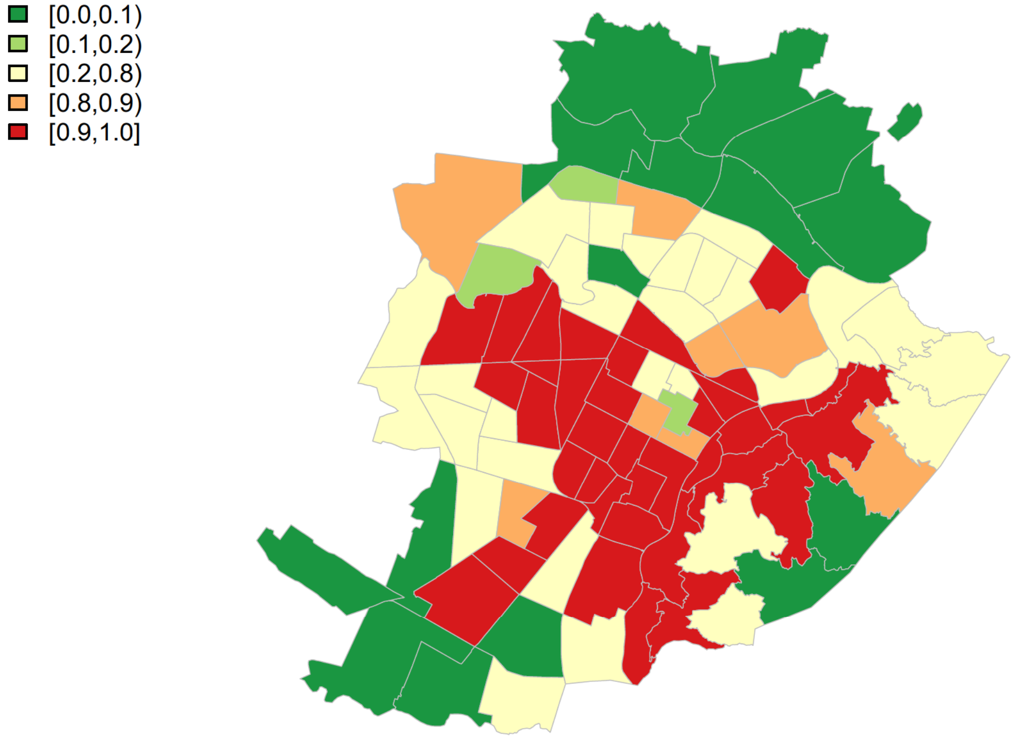

Avoidable mortality, Zurich , Males, 1995−2008
Smoothed Standardised Mortality Ratios (sSMR)

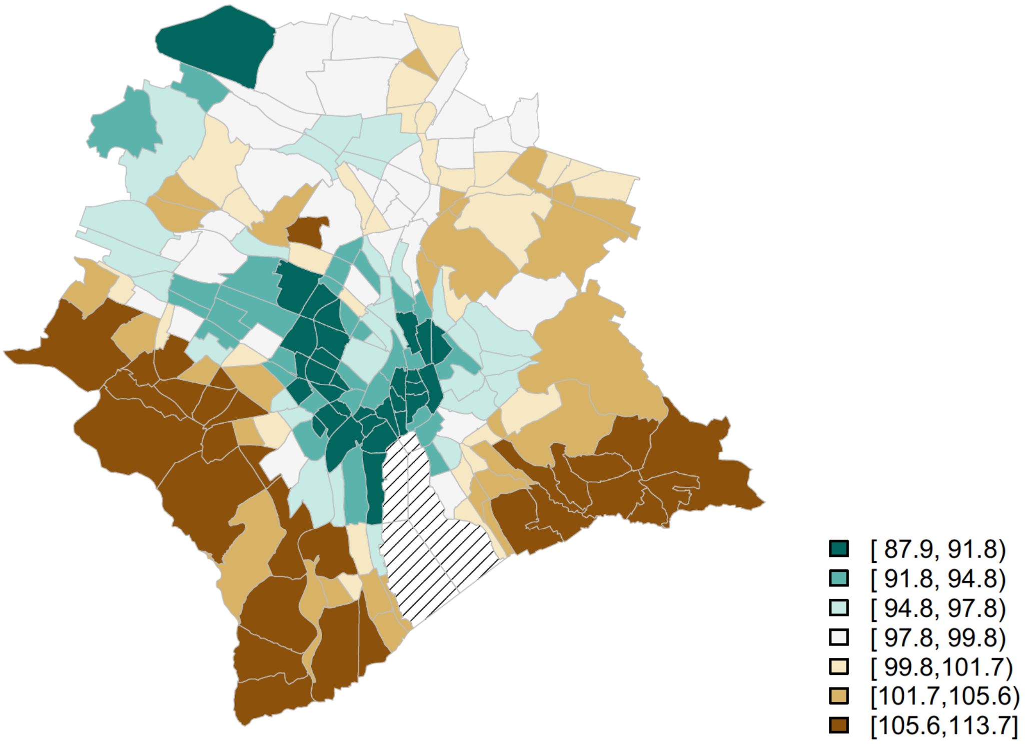

Avoidable mortality, Zurich , Males, 1995−2008
Probability that the sSMR is higher than 100

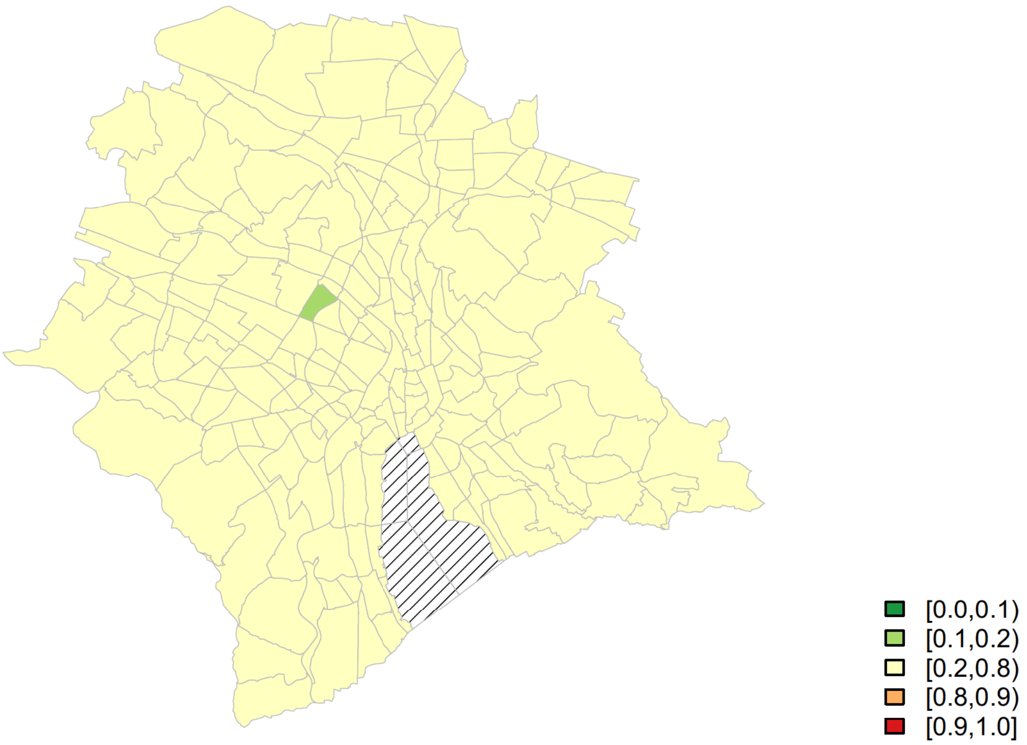

Avoidable mortality, Zurich , Females, 1995−2008
Smoothed Standardised Mortality Ratios (sSMR)

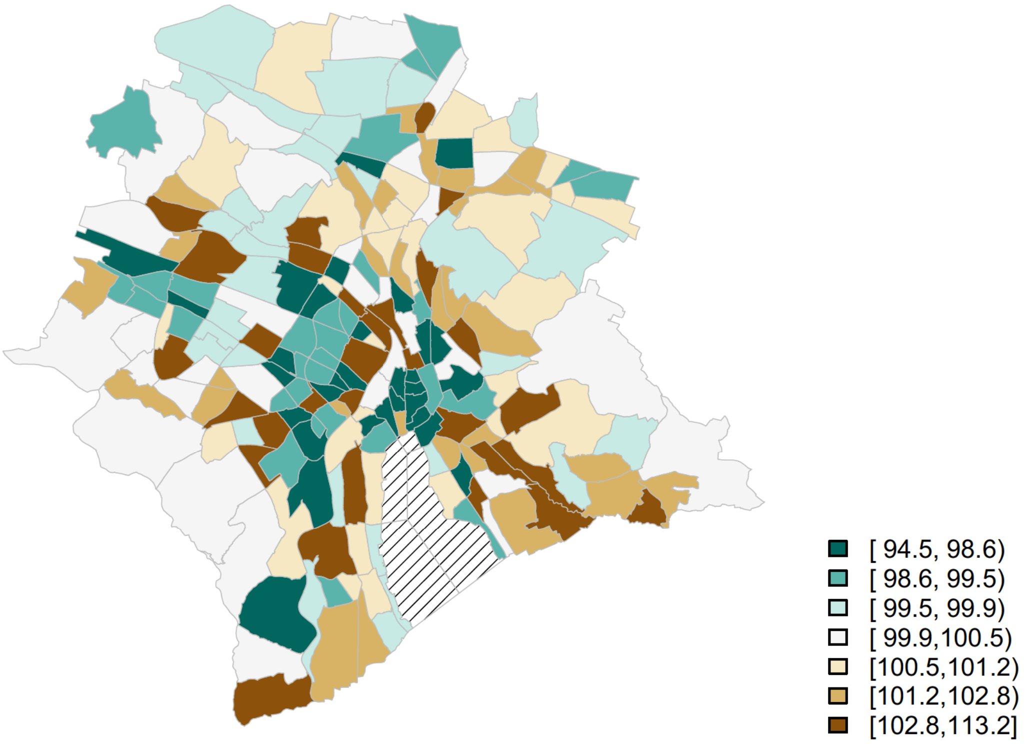

Avoidable mortality, Zurich , Females, 1995−2008
Probability that the sSMR is higher than 100

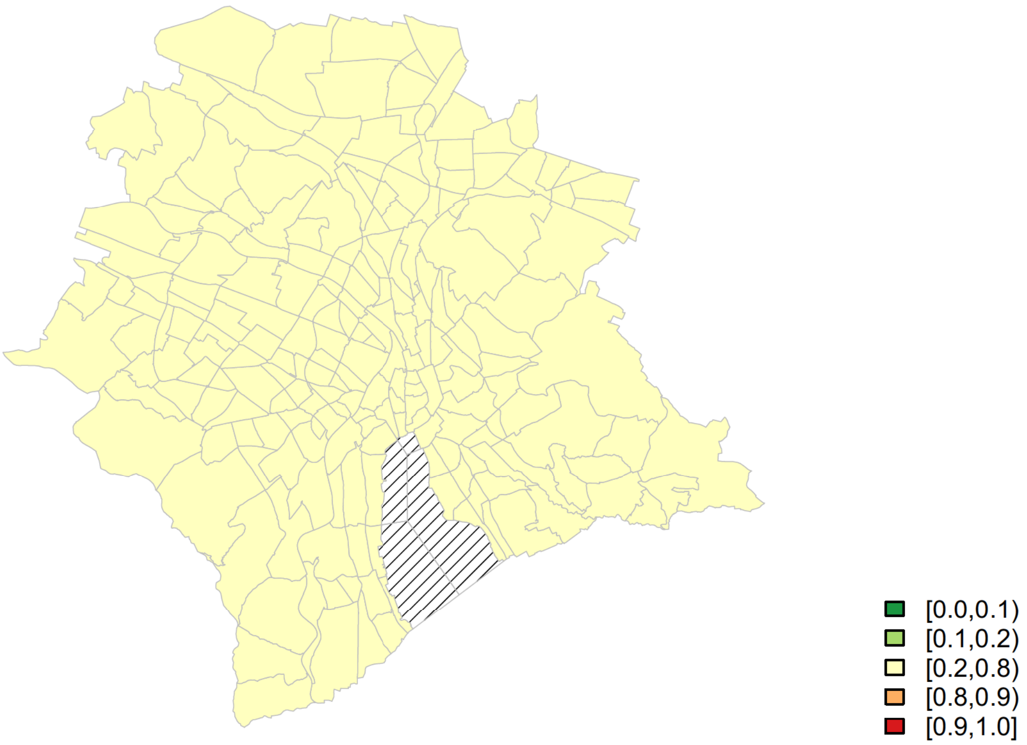

Supplement: Additional file 2 — Avoidable mortality maps for remaining 13 cities. [file 1476-072X-13-8-S2.pdf]
