# Supplementary material for: Social differences in avoidable mortality between small areas of 15 European cities: an ecological study
Source: Int J Health Geogr. 2014 Mar 12;13:8. doi: 10.1186/1476-072X-13-8 (PMC4007807; doi:10.1186/1476-072X-13-8)
Supplement: Additional file 3 — Cause-specific mortality maps for Amsterdam. [file 1476-072X-13-8-S3.pdf]

Amsterdam, Males, 1996 - 2008
AIDS (HIV disease)

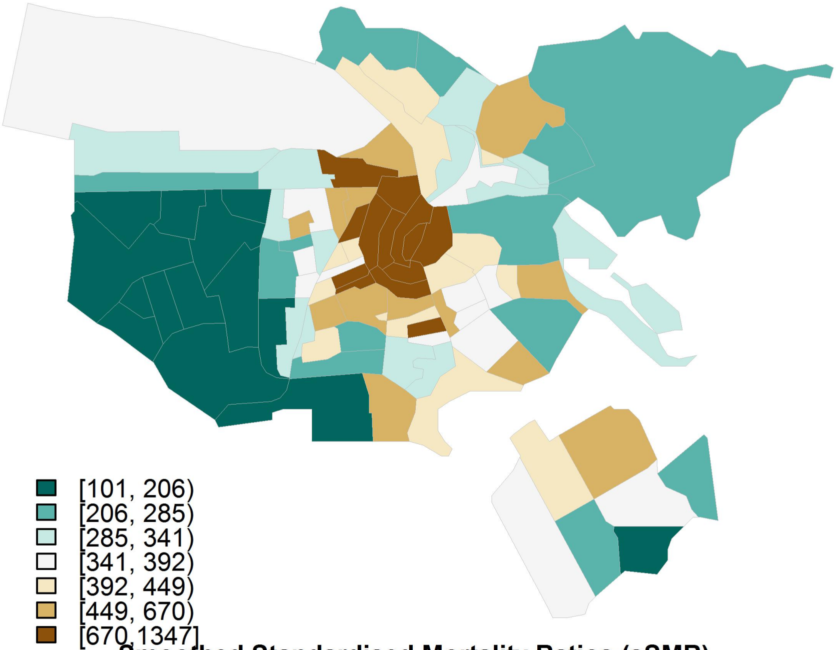

Smoothed Standardised Mortality Ratios (sSMR)
with respect to EU

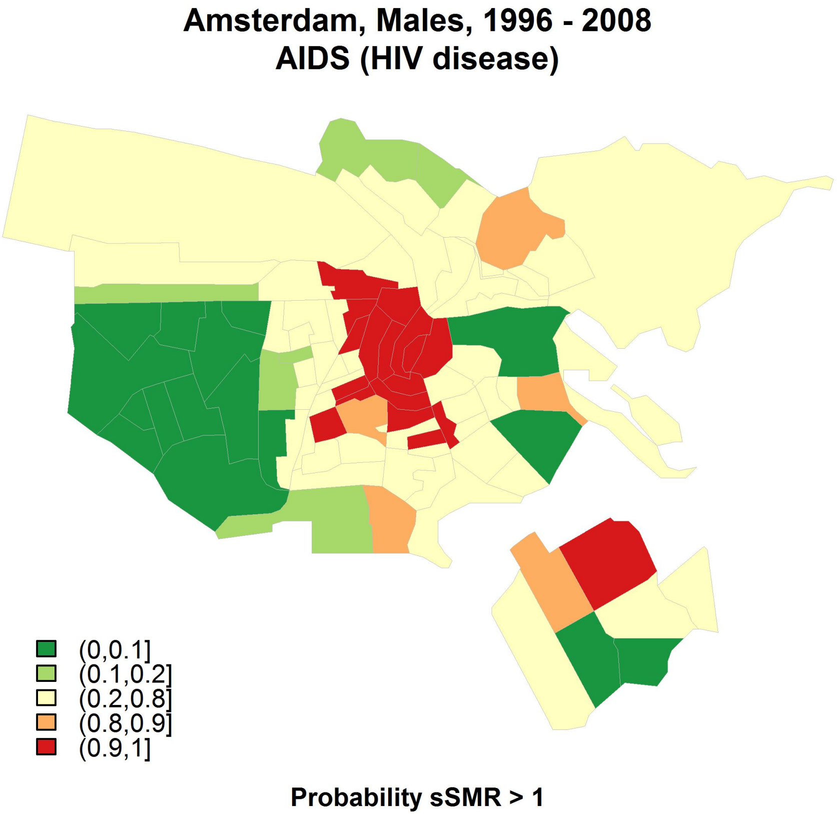
Amsterdam, Males, 1996 - 2008
AIDS (HIV disease)
(0,0.1]
(0.1,0.2]
(0.2,0.8]
(0.8,0.9]
(0.9,1]
Probability sSMR > 1

Amsterdam, Males, 1996 - 2008
MN colon

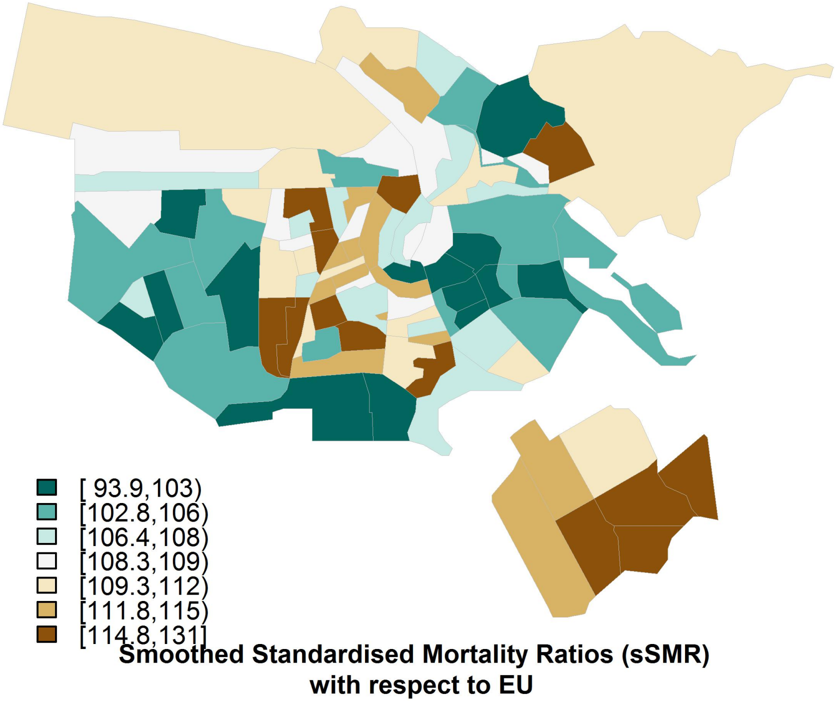

Smoothed Standardised Mortality Ratios (sSMR)
with respect to EU

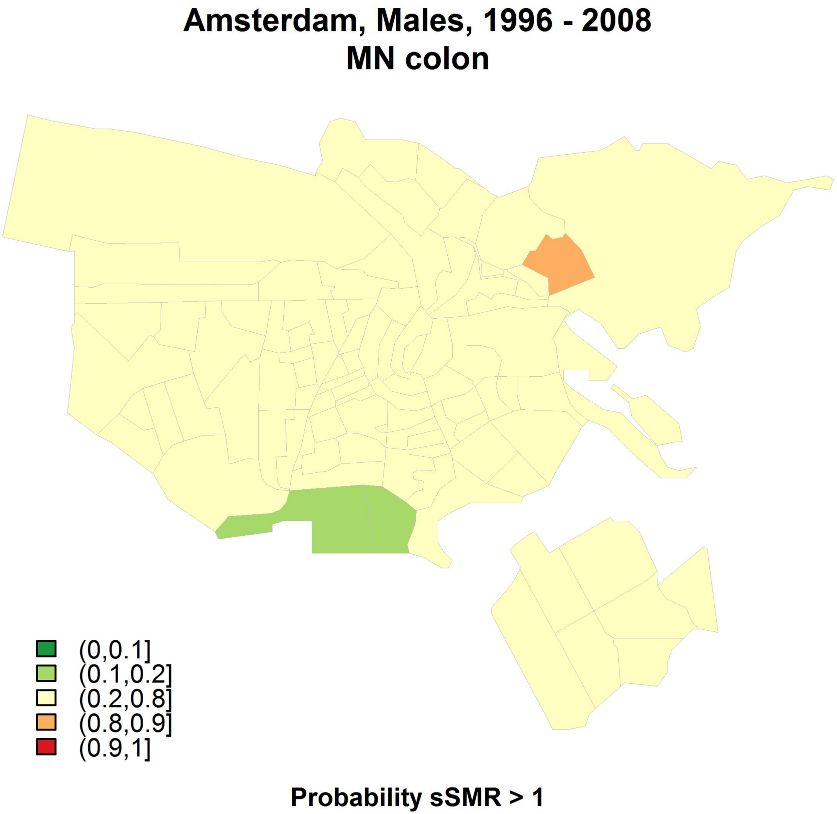
Amsterdam, Males, 1996 - 2008
MN colon
(0,0.1]
(0.1,0.2]
(0.2,0.8]
(0.8,0.9]
(0.9,1]
Probability sSMR > 1

Amsterdam, Males, 1996 - 2008
MN rectum, anus and anal canal

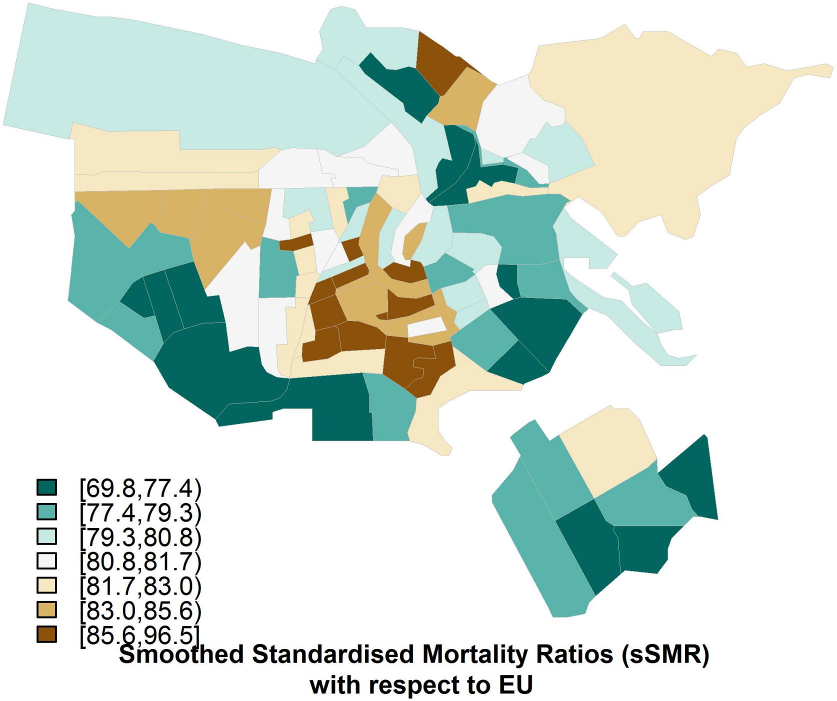

Smoothed Standardised Mortality Ratios (sSMR)
with respect to EU

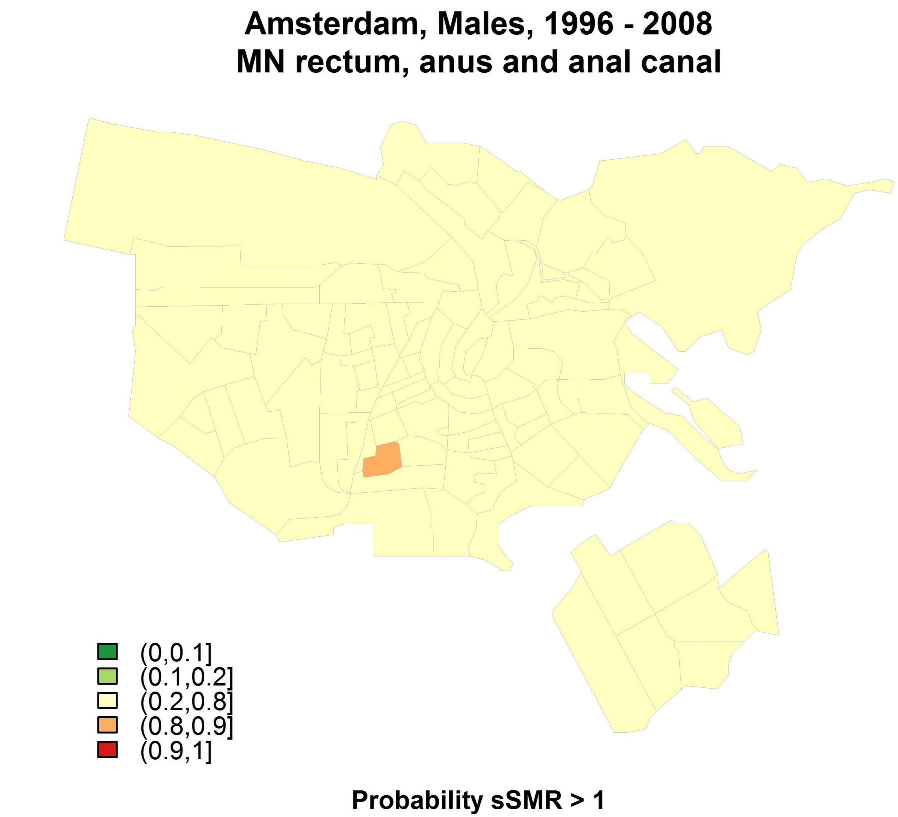

Amsterdam, Males, 1996 - 2008
MN rectum, anus and anal canal
(0,0.1]
(0.1,0.2]
(0.2,0.8]
(0.8,0.9]
(0.9,1]
Probability sSMR > 1

Amsterdam, Males, 1996 - 2008
Hypertension

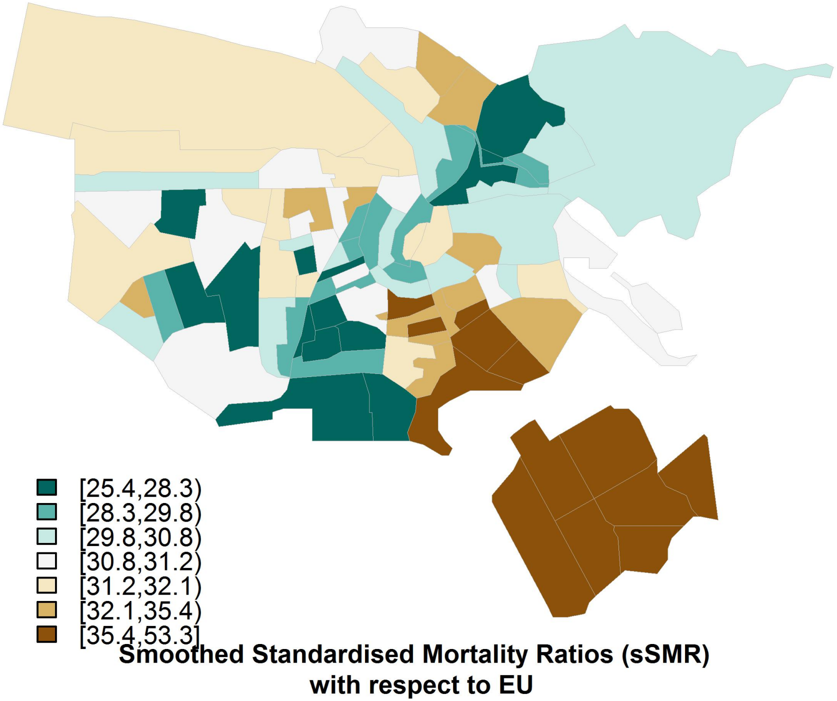

Smoothed Standardised Mortality Ratios (sSMR)
with respect to EU

Amsterdam, Males, 1996 - 2008
Hypertension

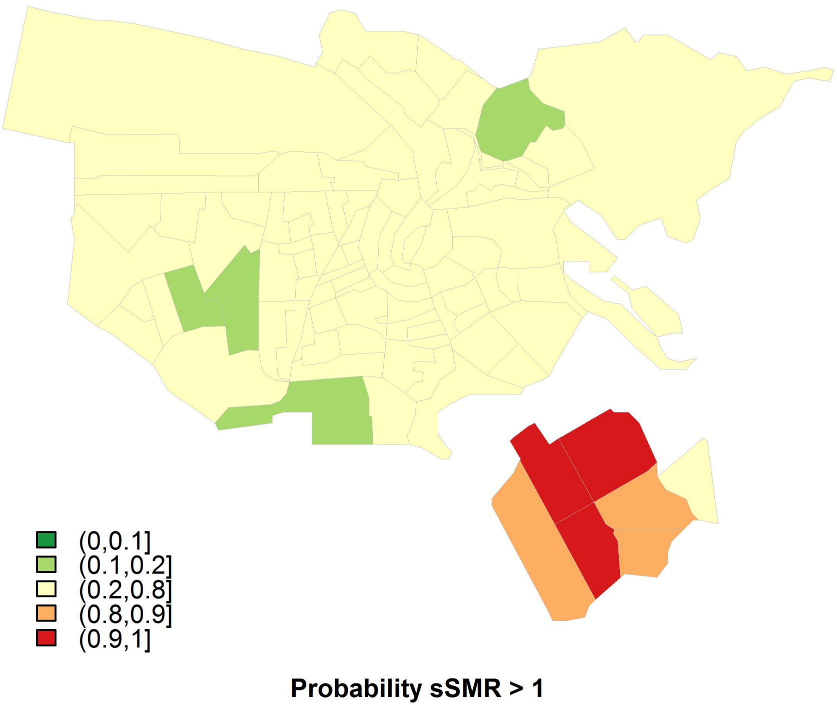

Amsterdam, Males, 1996 - 2008
Heart failure

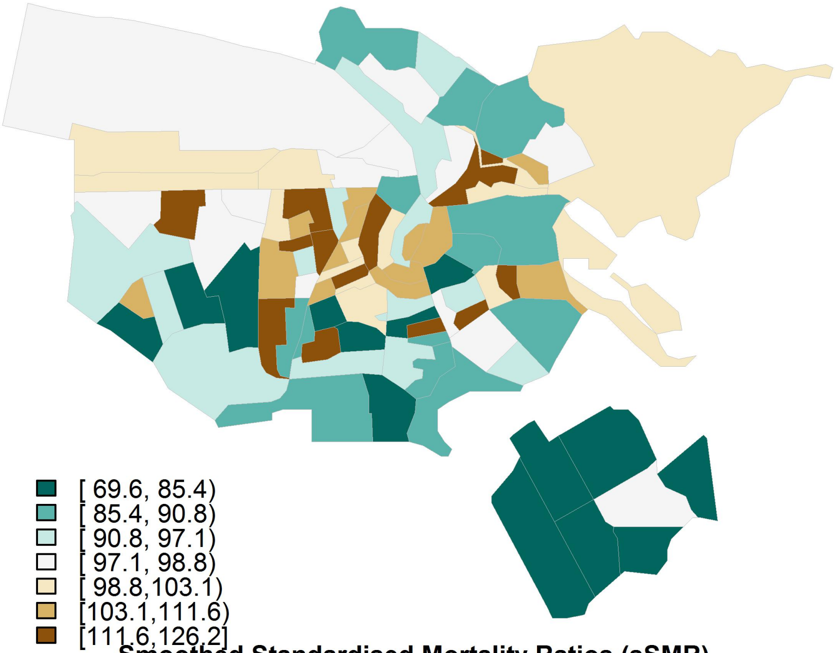

**Smoothed Standardised Mortality Ratios (sSMR)
with respect to EU**

Amsterdam, Males, 1996 - 2008
Heart failure

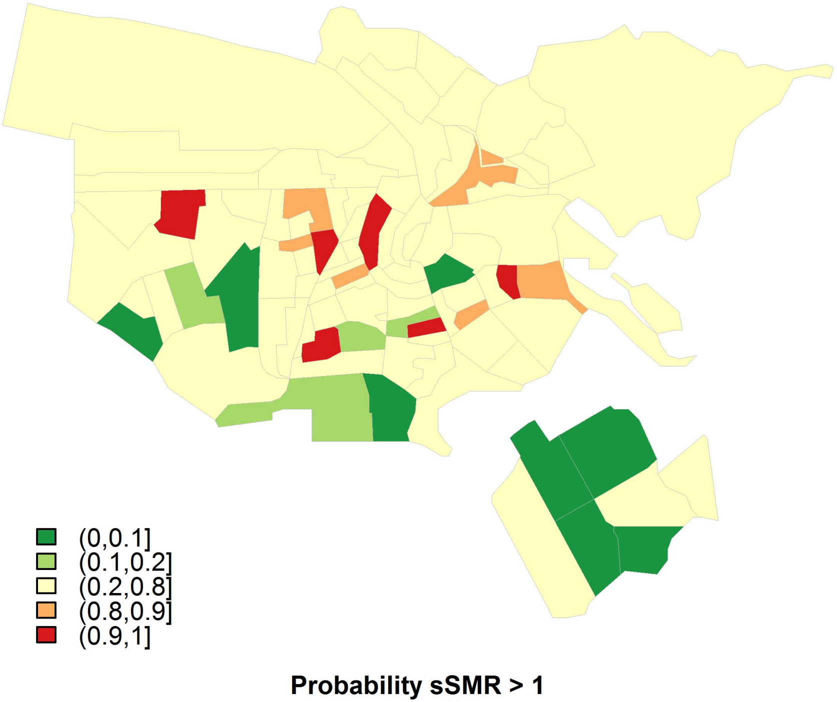

Amsterdam, Males, 1996 - 2008
Cerebrovascular diseases

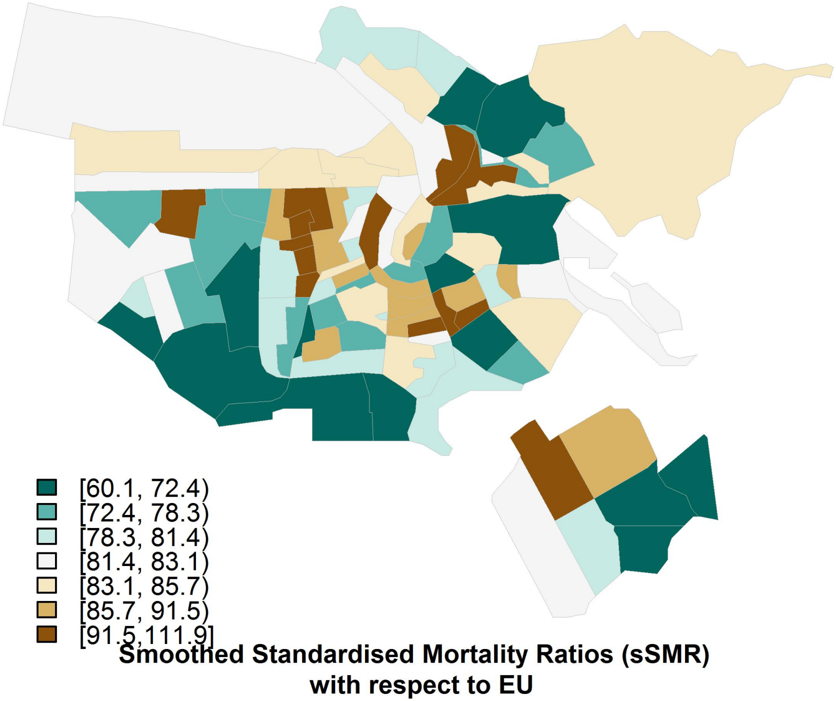

Smoothed Standardised Mortality Ratios (sSMR)
with respect to EU

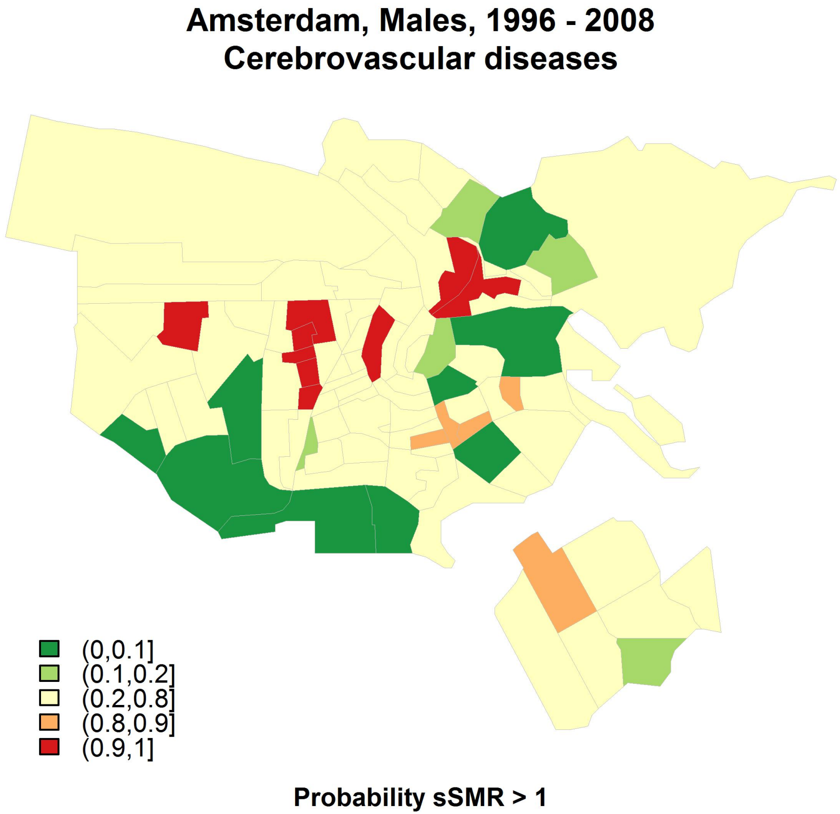
Amsterdam, Males, 1996 - 2008
Cerebrovascular diseases
(0,0.1]
(0.1,0.2]
(0.2,0.8]
(0.8,0.9]
(0.9,1]
Probability sSMR > 1

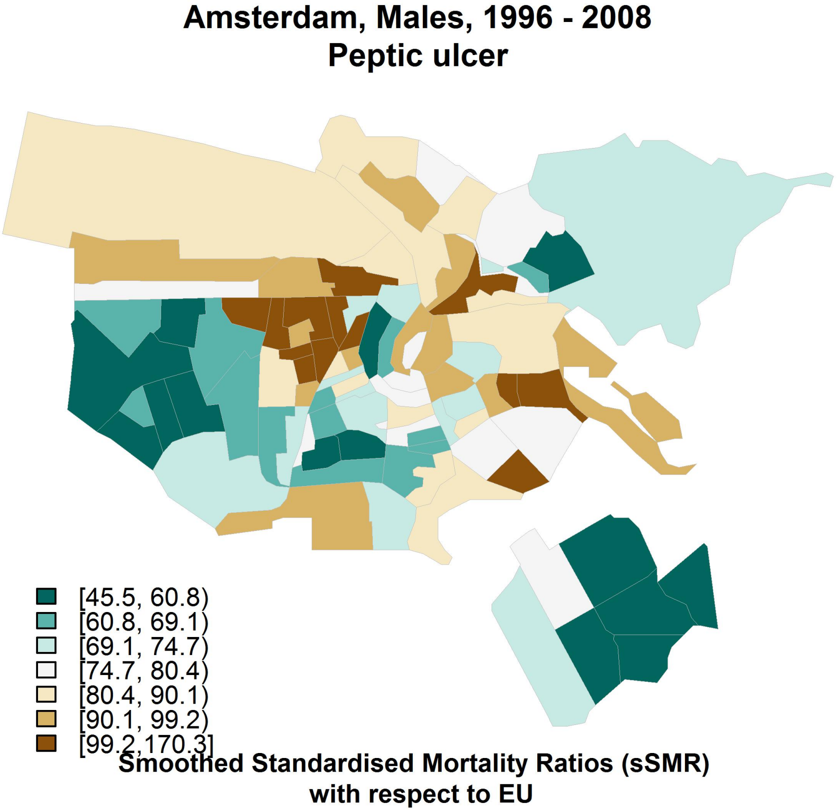
Amsterdam, Males, 1996 - 2008
Peptic ulcer
[45.5, 60.8)
[60.8, 69.1)
[69.1, 74.7)
[74.7, 80.4)
[80.4, 90.1)
[90.1, 99.2)
[99.2,170.3]
Smoothed Standardised Mortality Ratios (sSMR)
with respect to EU

Amsterdam, Males, 1996 - 2008
Peptic ulcer

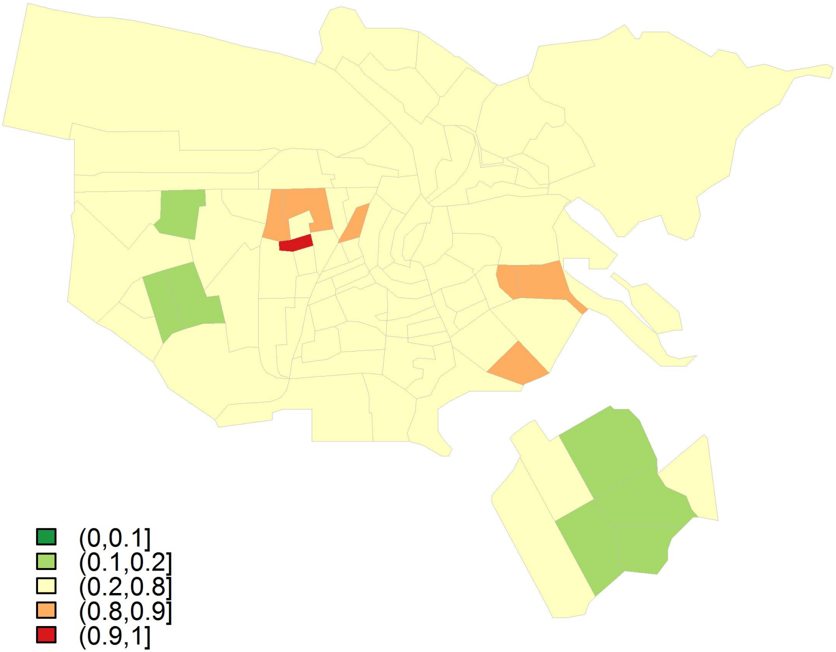

Probability sSMR > 1

Amsterdam, Males, 1996 - 2008
Renal failure

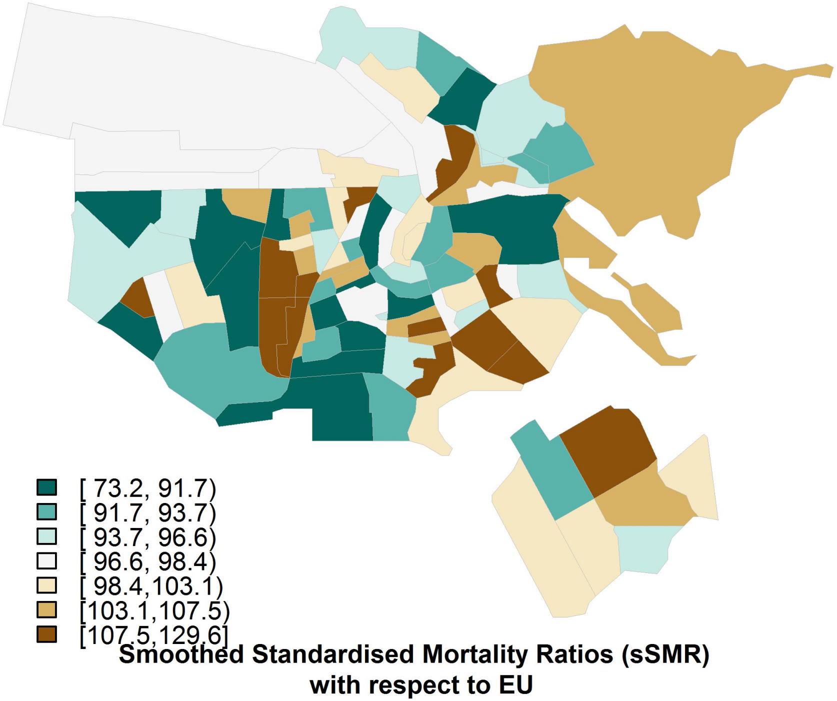

Smoothed Standardised Mortality Ratios (sSMR)
with respect to EU

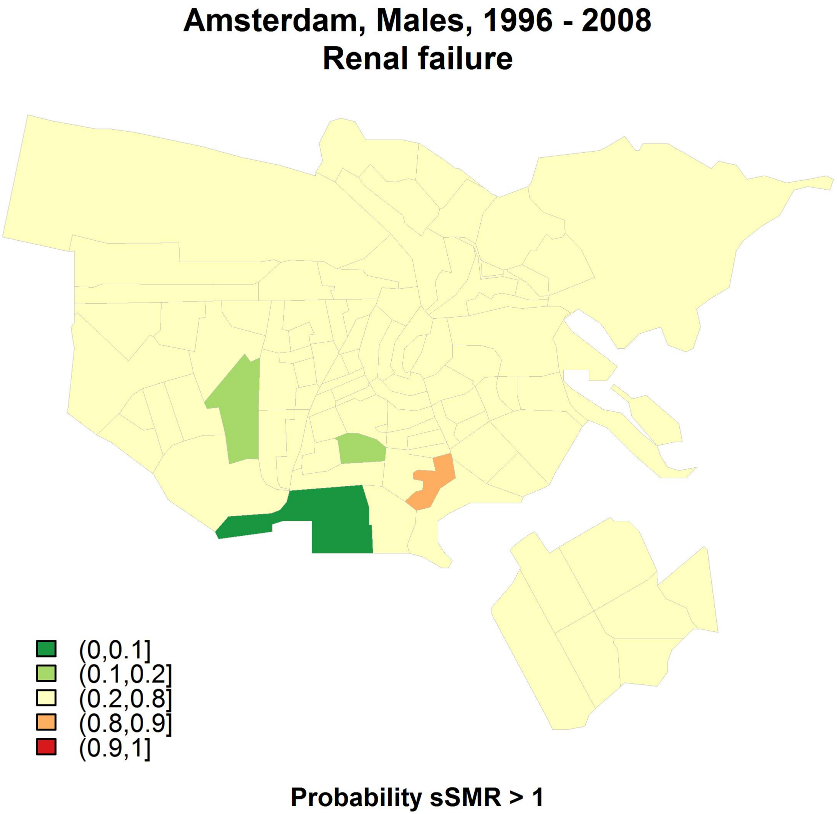
Amsterdam, Males, 1996 - 2008
Renal failure
(0,0.1]
(0.1,0.2]
(0.2,0.8]
(0.8,0.9]
(0.9,1]
Probability sSMR > 1

Amsterdam, Males, 1996 - 2008
Conditions originating in the perinatal period

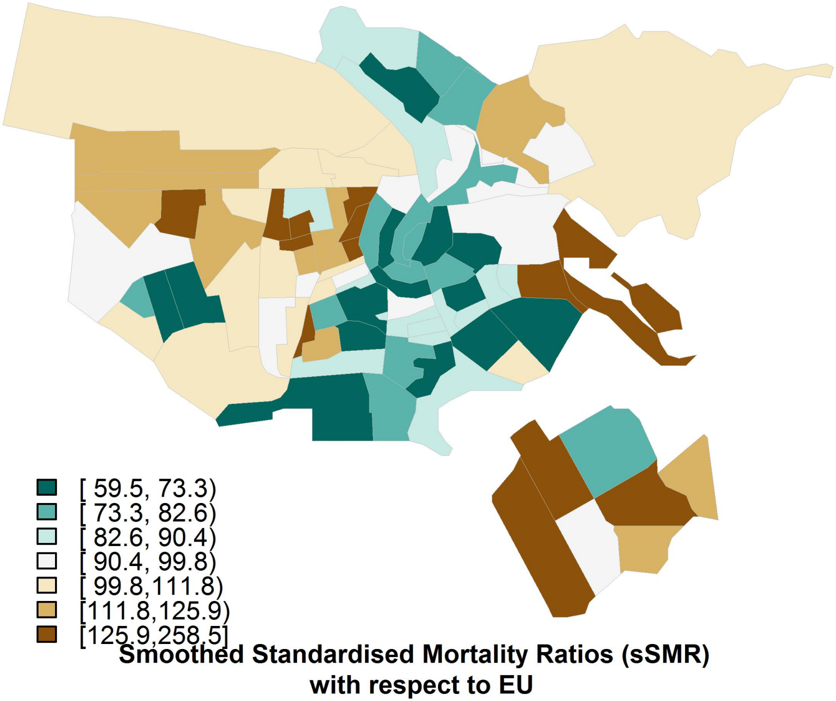

Smoothed Standardised Mortality Ratios (sSMR)
with respect to EU

Amsterdam, Males, 1996 - 2008
Conditions originating in the perinatal period

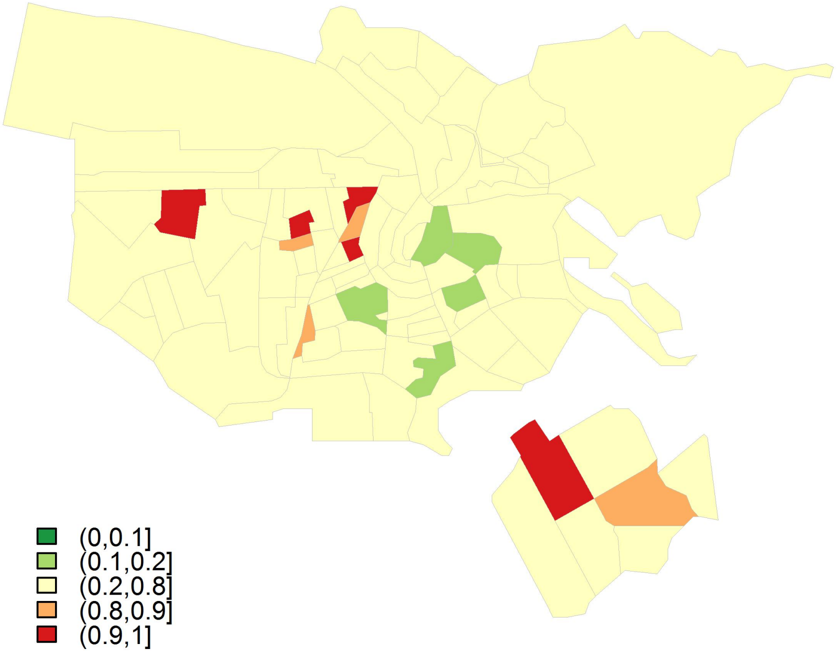

Probability sSMR > 1

Amsterdam, Males, 1996 - 2008
Congenital heart disease

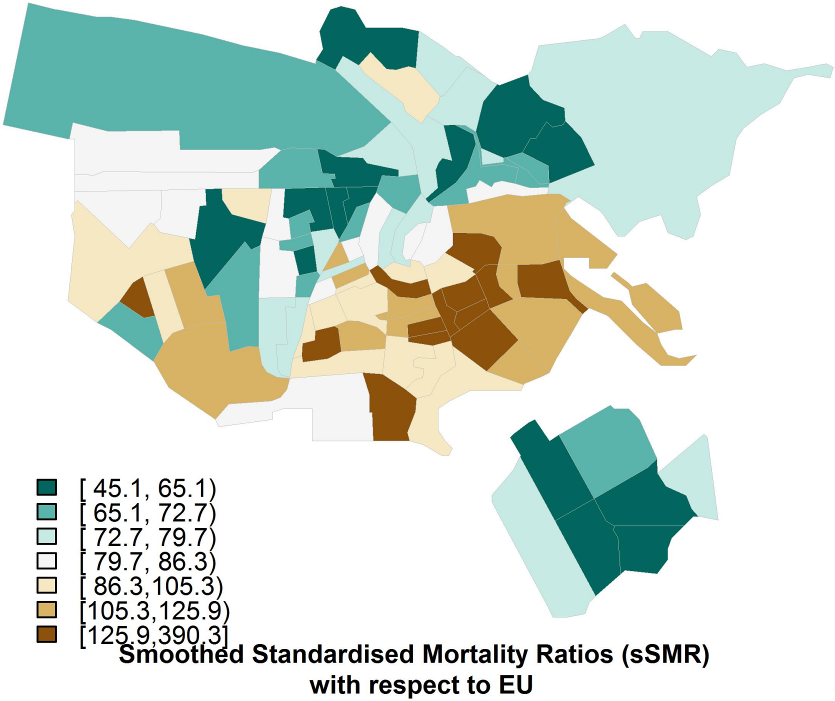

Smoothed Standardised Mortality Ratios (sSMR)
with respect to EU

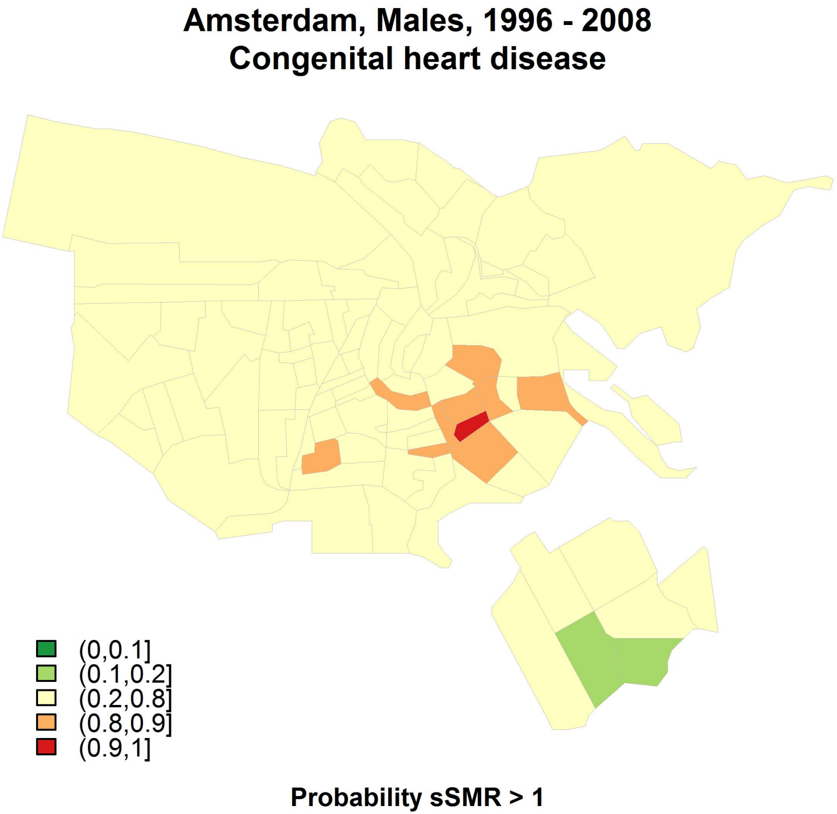
Amsterdam, Males, 1996 - 2008
Congenital heart disease
(0,0.1]
(0.1,0.2]
(0.2,0.8]
(0.8,0.9]
(0.9,1]
Probability sSMR > 1

Amsterdam, Females, 1996 - 2008
AIDS (HIV disease)

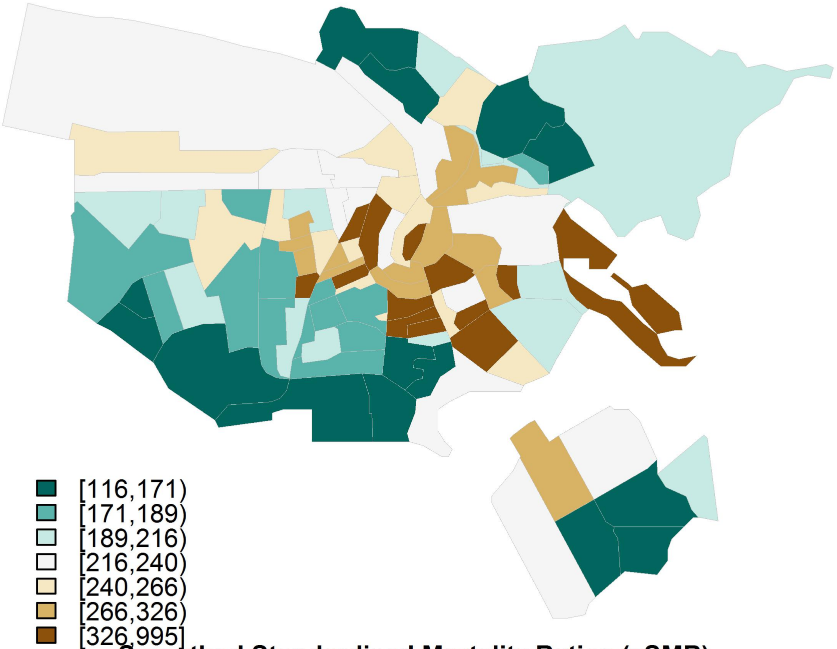

**Smoothed Standardised Mortality Ratios (sSMR)
with respect to EU**

Amsterdam, Females, 1996 - 2008
AIDS (HIV disease)

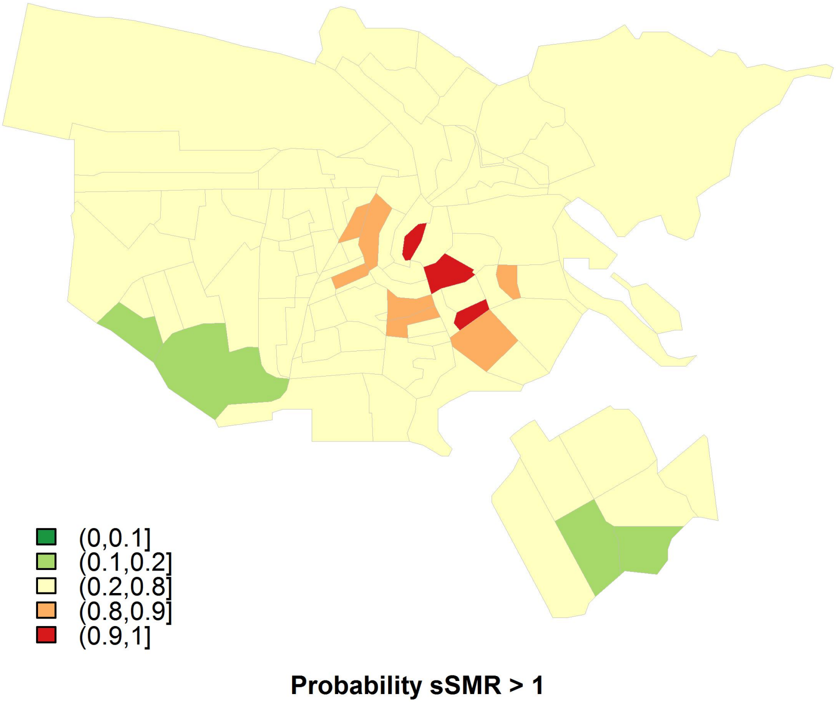

Amsterdam, Females, 1996 - 2008
MN colon

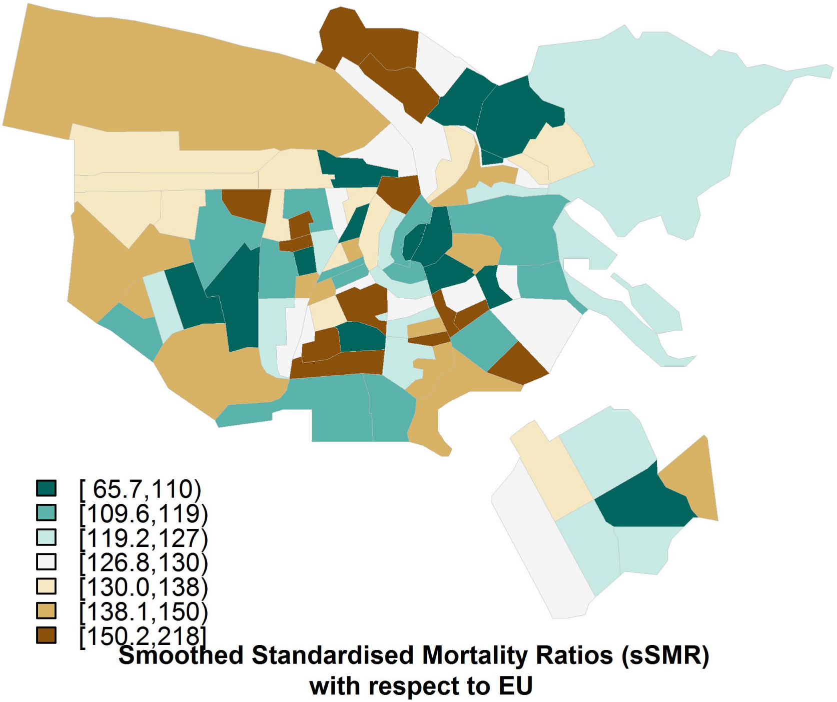

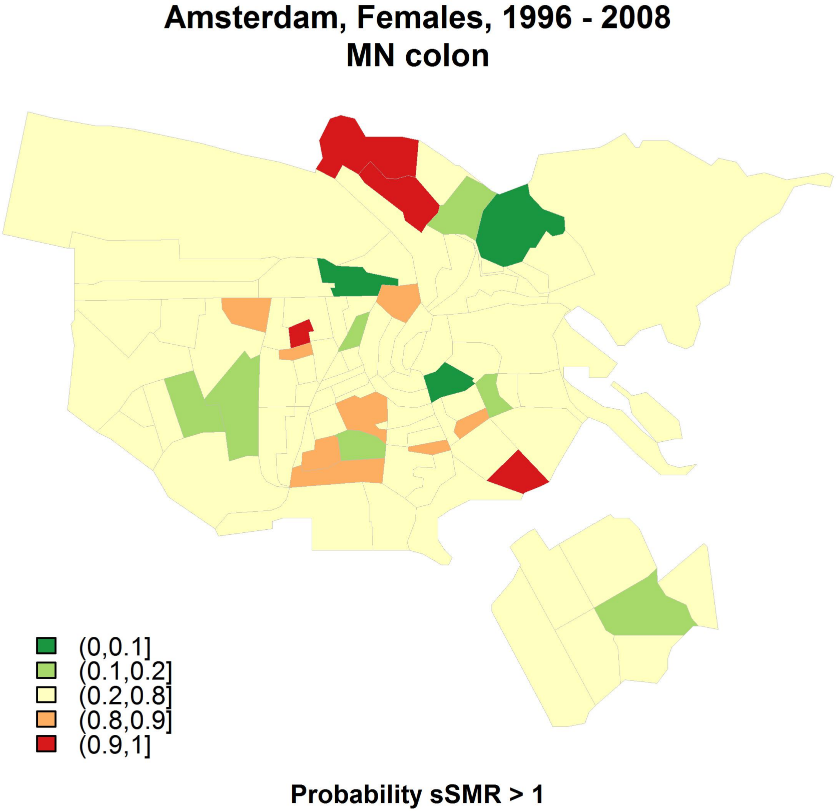
Amsterdam, Females, 1996 - 2008
MN colon
(0,0.1]
(0.1,0.2]
(0.2,0.8]
(0.8,0.9]
(0.9,1]
Probability sSMR > 1

Amsterdam, Females, 1996 - 2008
MN rectum, anus and anal canal

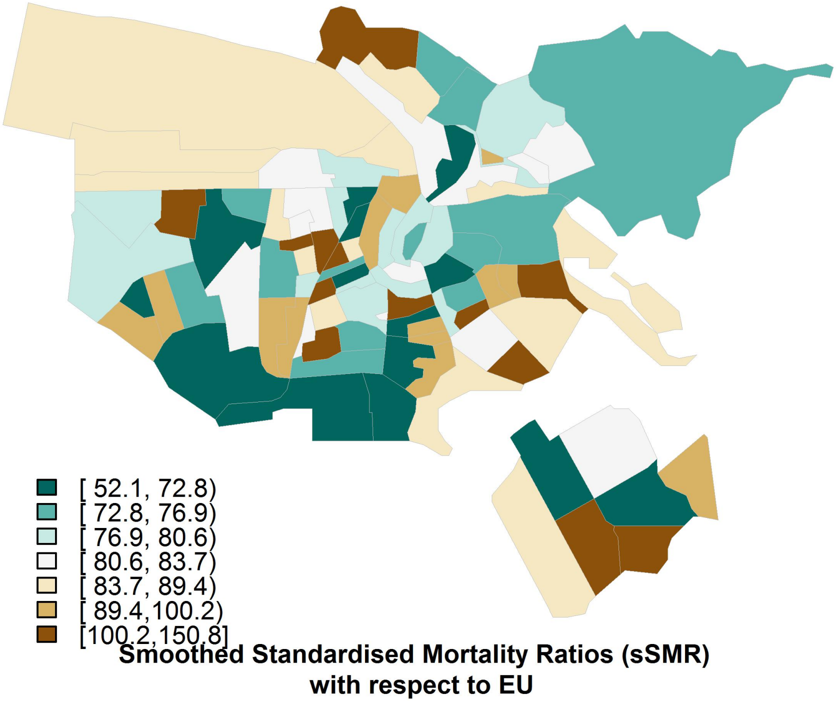

Smoothed Standardised Mortality Ratios (sSMR)
with respect to EU

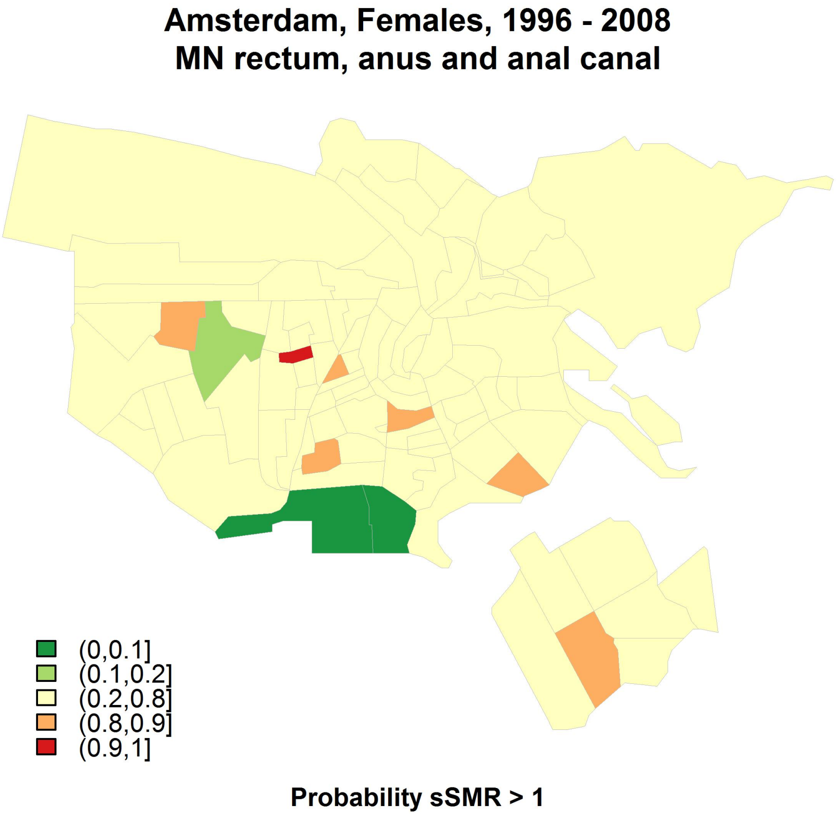
Amsterdam, Females, 1996 - 2008
MN rectum, anus and anal canal
(0,0.1]
(0.1,0.2]
(0.2,0.8]
(0.8,0.9]
(0.9,1]
Probability sSMR > 1

Amsterdam, Females, 1996 - 2008
MN cervix uteri

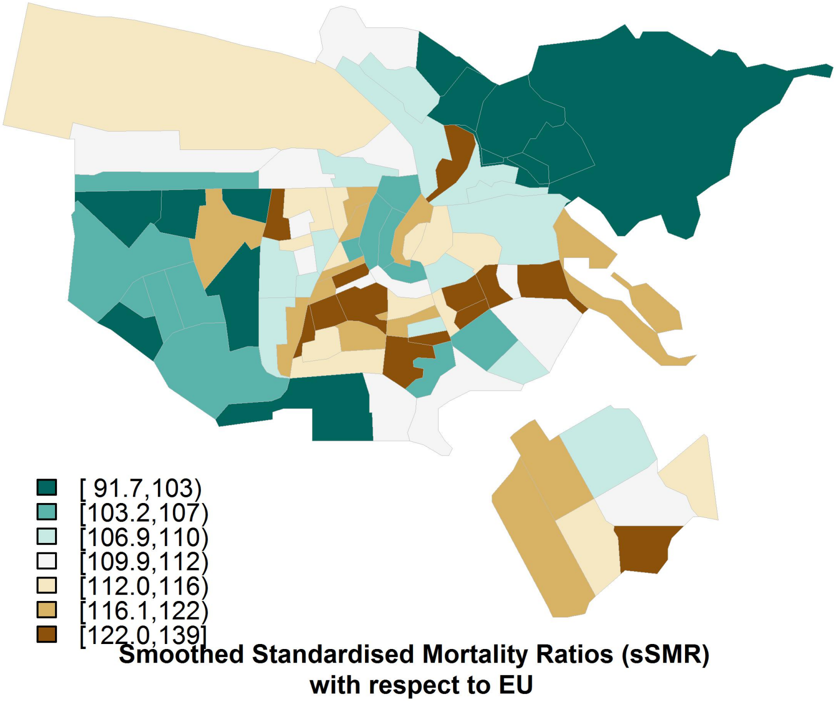

Smoothed Standardised Mortality Ratios (sSMR)
with respect to EU

Amsterdam, Females, 1996 - 2008
MN cervix uteri

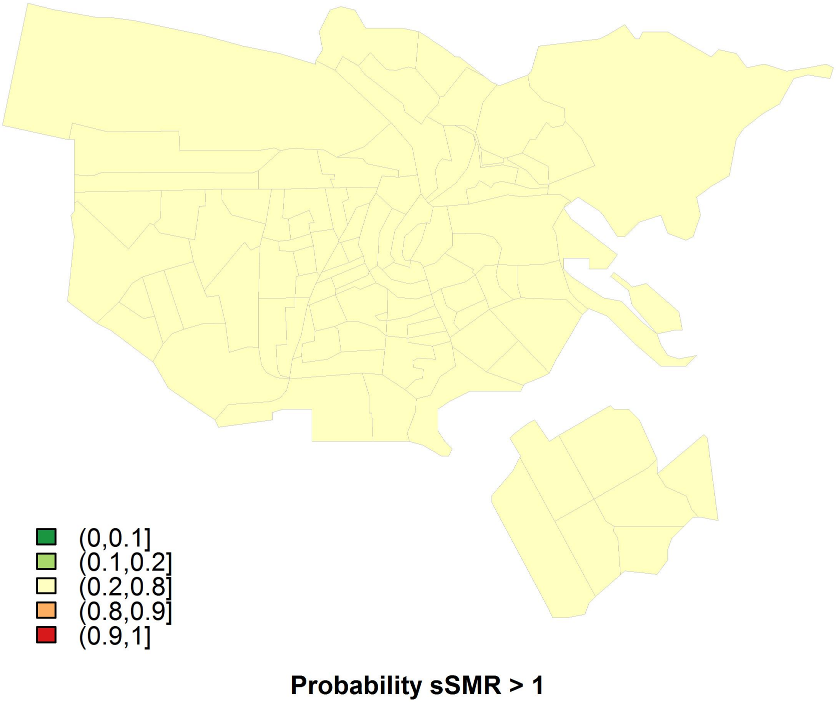

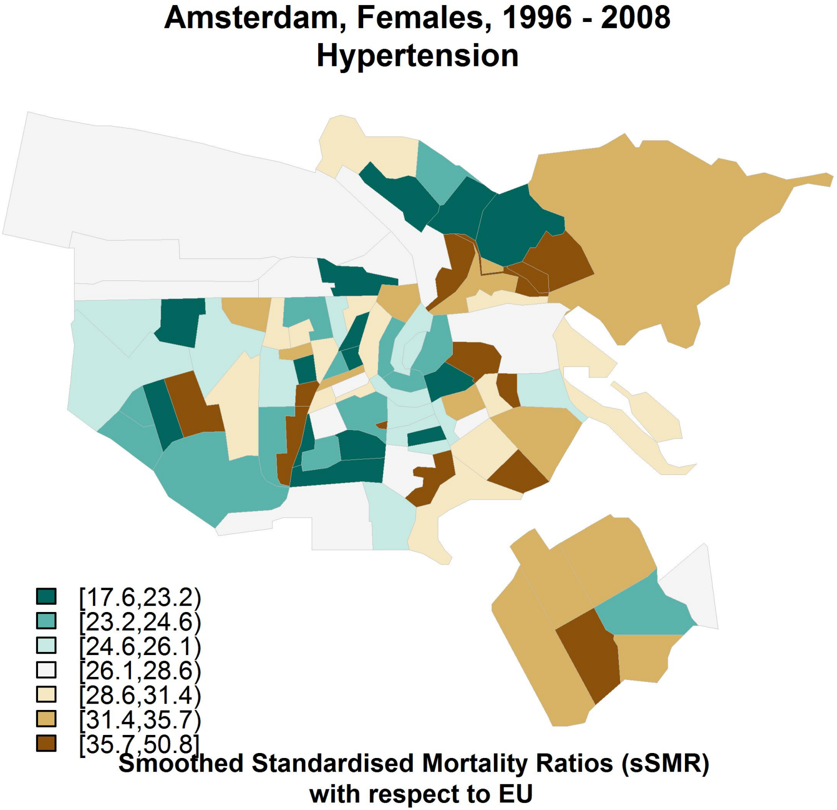
Amsterdam, Females, 1996 - 2008
Hypertension
[17.6,23.2)
[23.2,24.6)
[24.6,26.1)
[26.1,28.6)
[28.6,31.4)
[31.4,35.7)
[35.7,50.8]
Smoothed Standardised Mortality Ratios (sSMR)
with respect to EU

Amsterdam, Females, 1996 - 2008
Hypertension

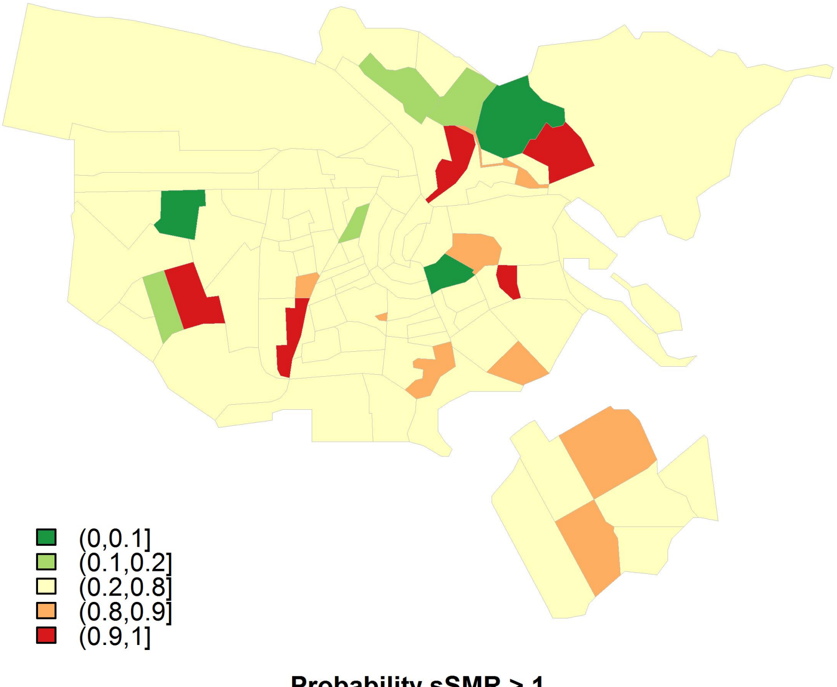

Amsterdam, Females, 1996 - 2008
Heart failure

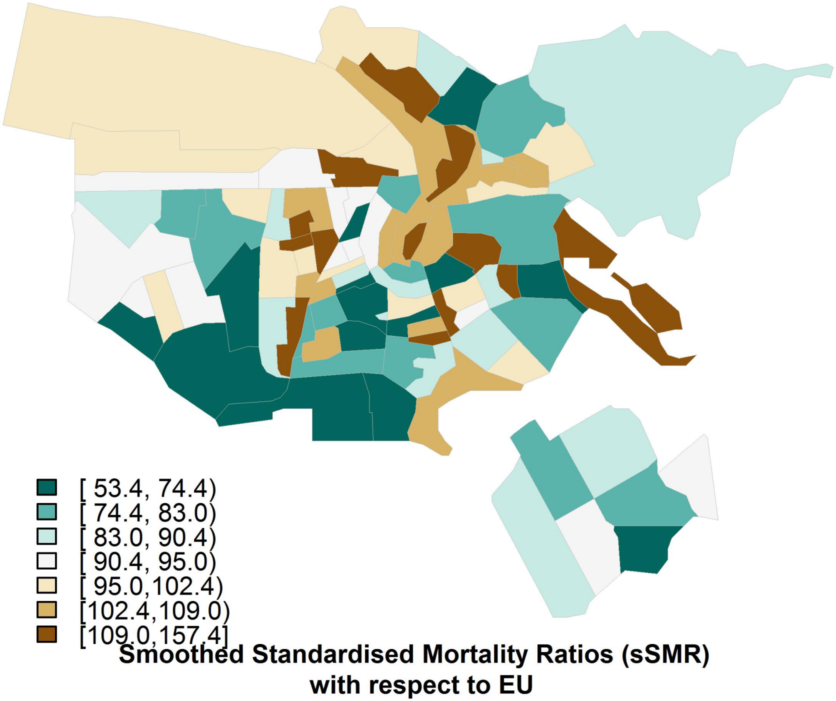

Smoothed Standardised Mortality Ratios (sSMR) with respect to EU

Amsterdam, Females, 1996 - 2008
Heart failure

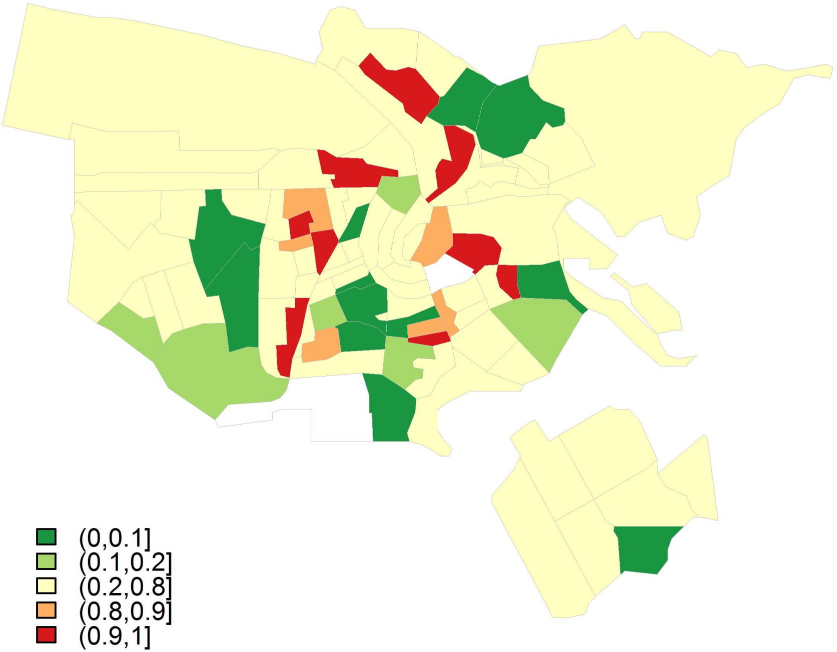

Probability sSMR > 1

Amsterdam, Females, 1996 - 2008
Cerebrovascular diseases

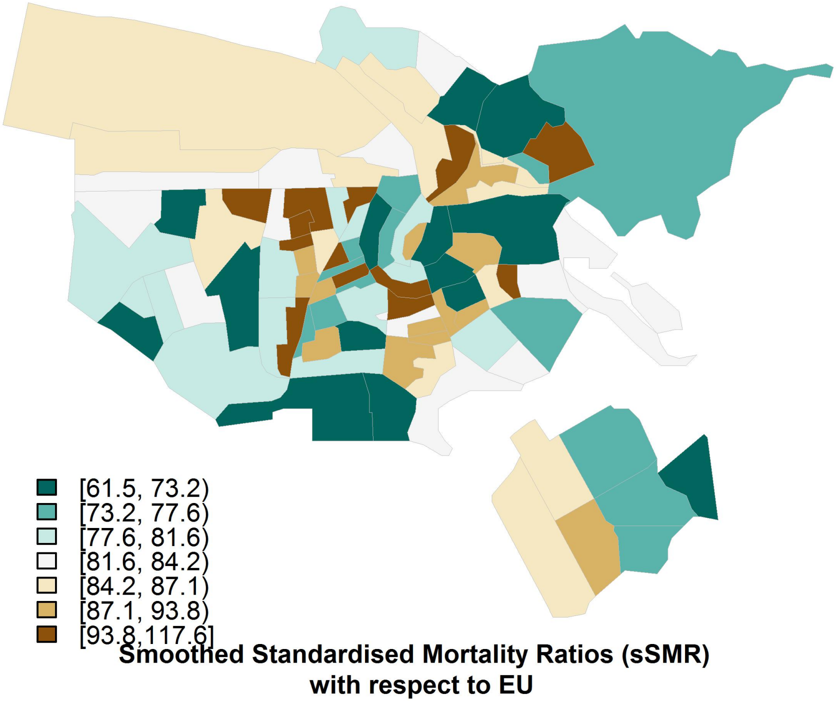

Smoothed Standardised Mortality Ratios (sSMR)
with respect to EU

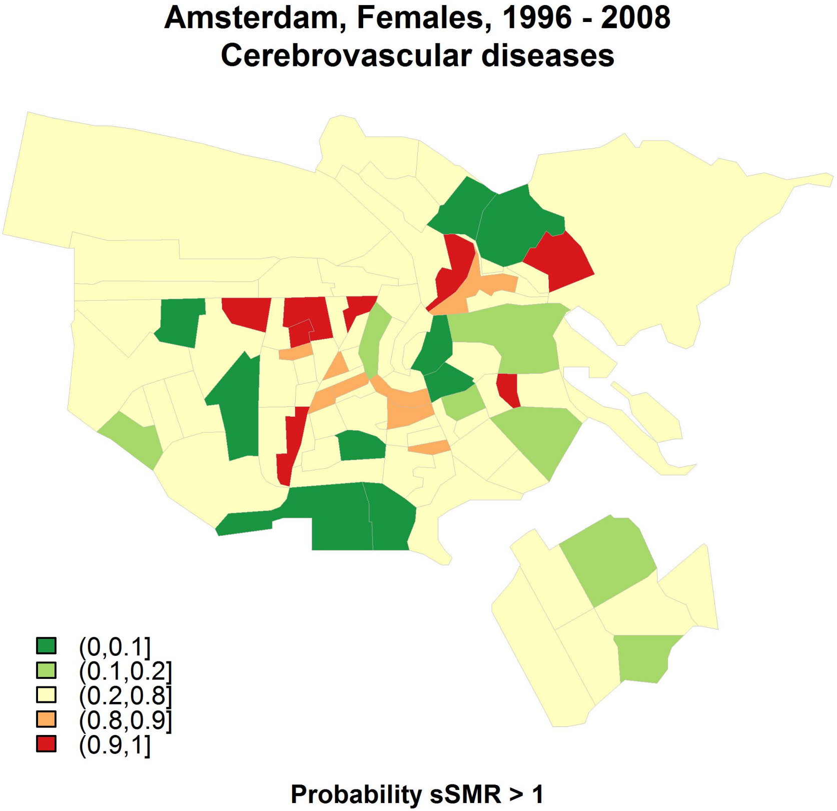
Amsterdam, Females, 1996 - 2008
Cerebrovascular diseases
(0,0.1]
(0.1,0.2]
(0.2,0.8]
(0.8,0.9]
(0.9,1]
Probability sSMR > 1

Amsterdam, Females, 1996 - 2008
Peptic ulcer

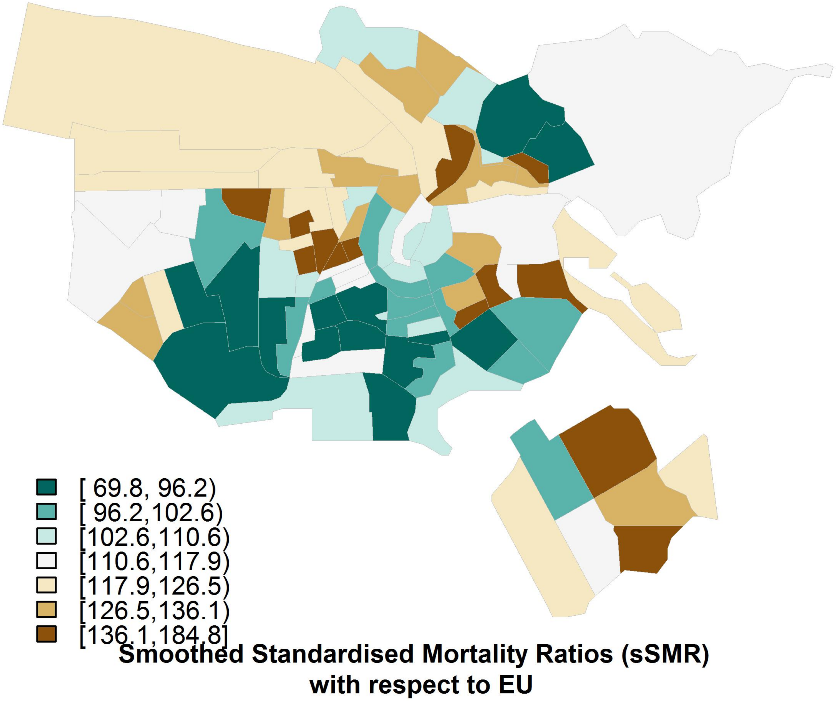

Smoothed Standardised Mortality Ratios (sSMR)
with respect to EU

Amsterdam, Females, 1996 - 2008
Peptic ulcer

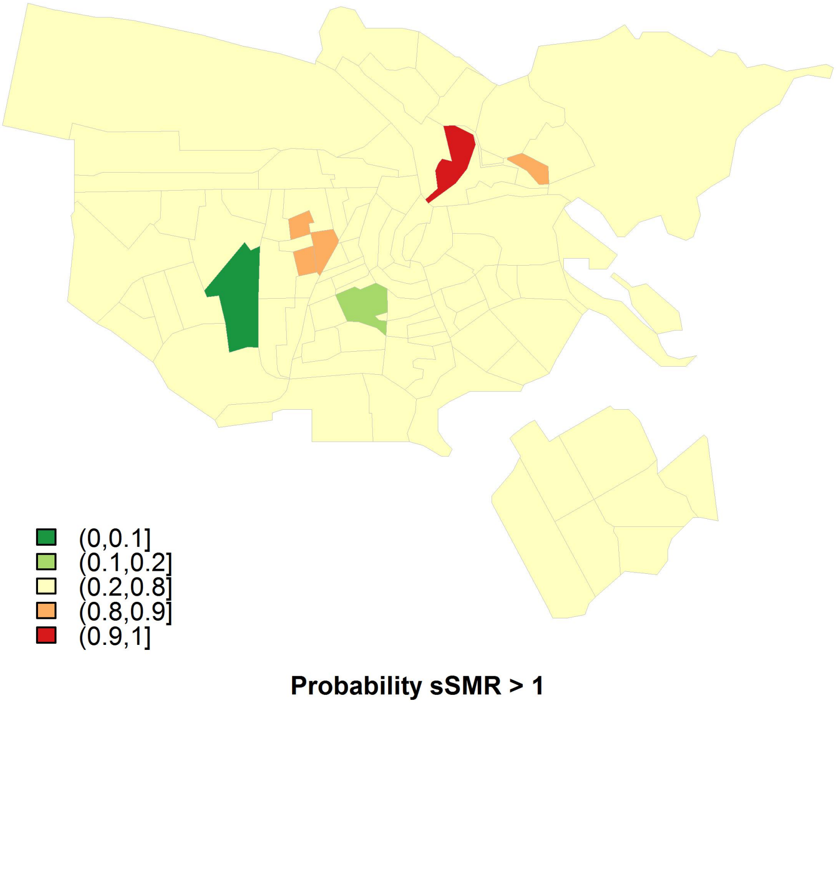

Amsterdam, Females, 1996 - 2008
Renal failure

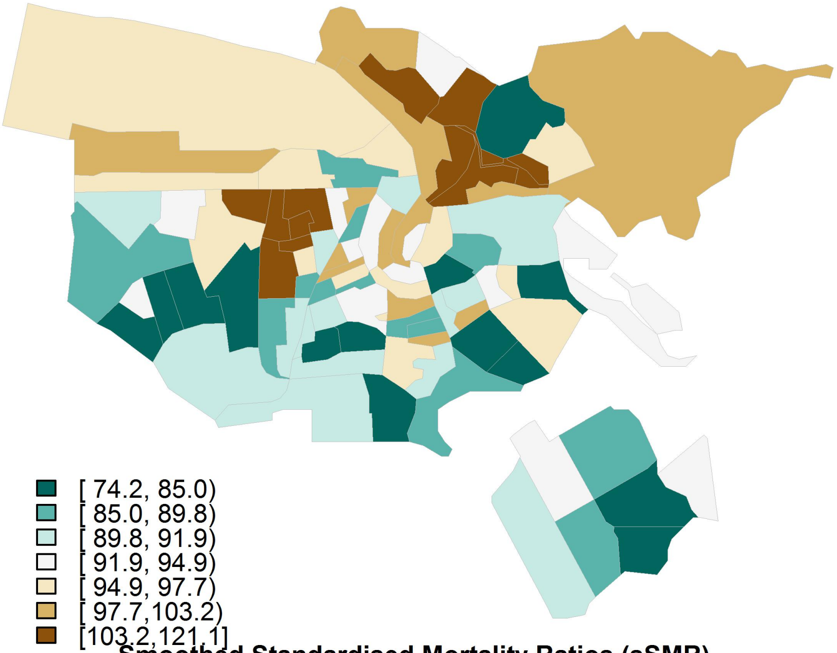

Smoothed Standardised Mortality Ratios (sSMR)
with respect to EU

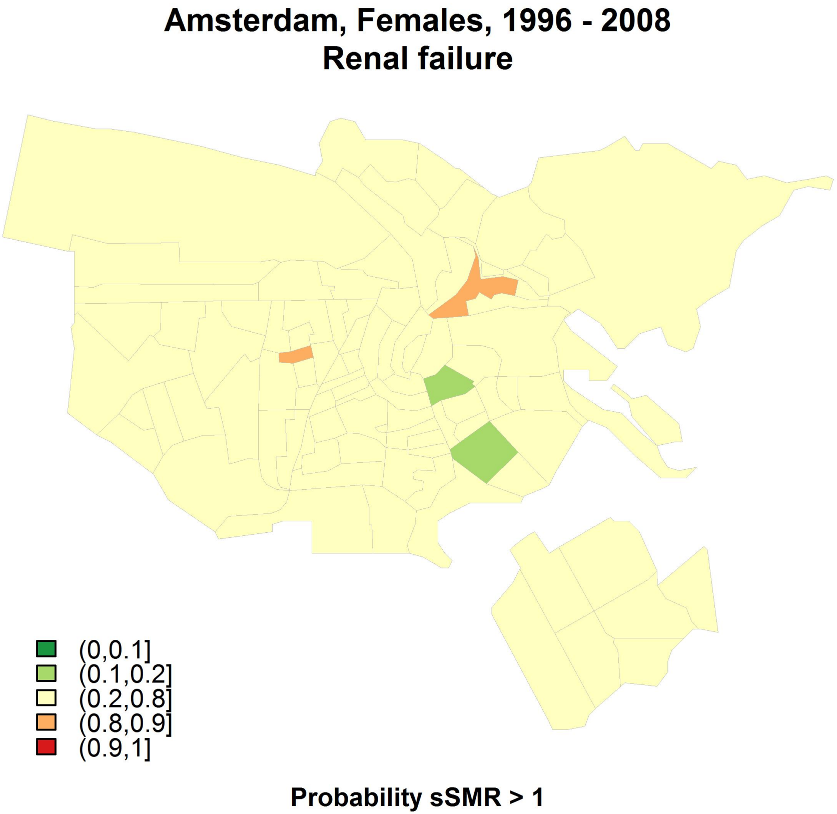
Amsterdam, Females, 1996 - 2008
Renal failure
(0,0.1]
(0.1,0.2]
(0.2,0.8]
(0.8,0.9]
(0.9,1]
Probability sSMR > 1

Amsterdam, Females, 1996 - 2008
Conditions originating in the perinatal period

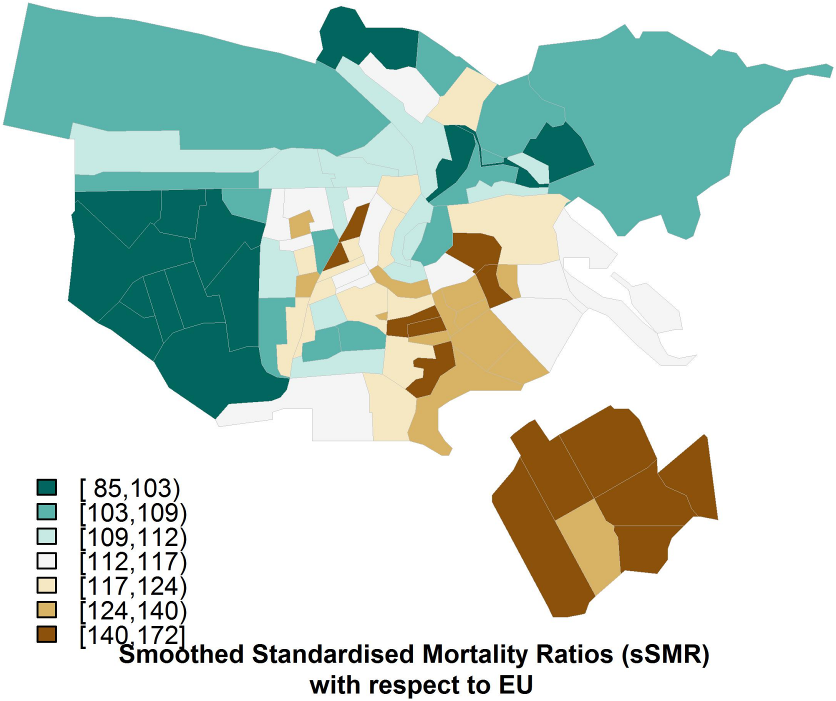

Smoothed Standardised Mortality Ratios (sSMR)
with respect to EU

Amsterdam, Females, 1996 - 2008
Conditions originating in the perinatal period

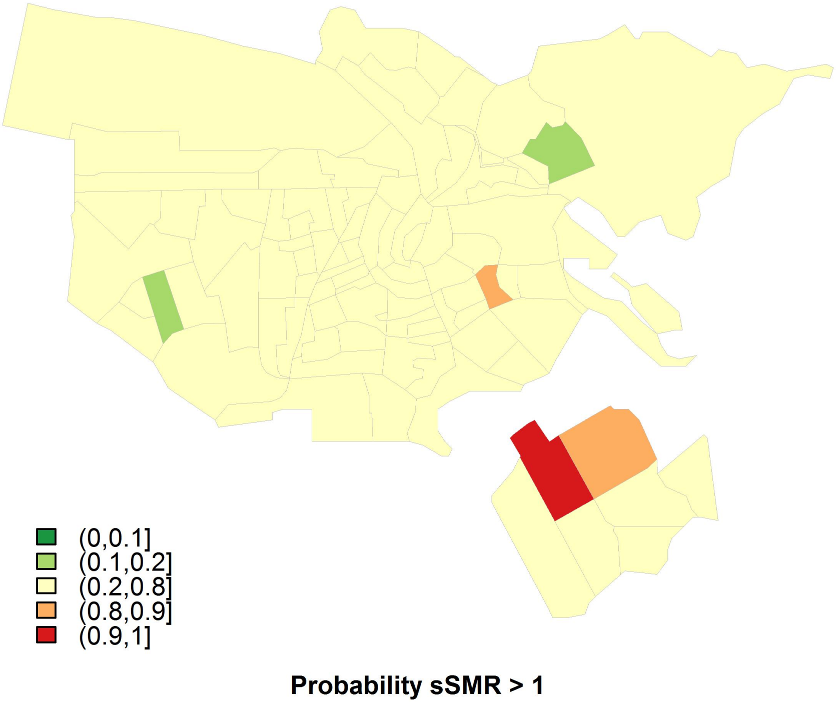

Amsterdam, Females, 1996 - 2008
Congenital heart disease

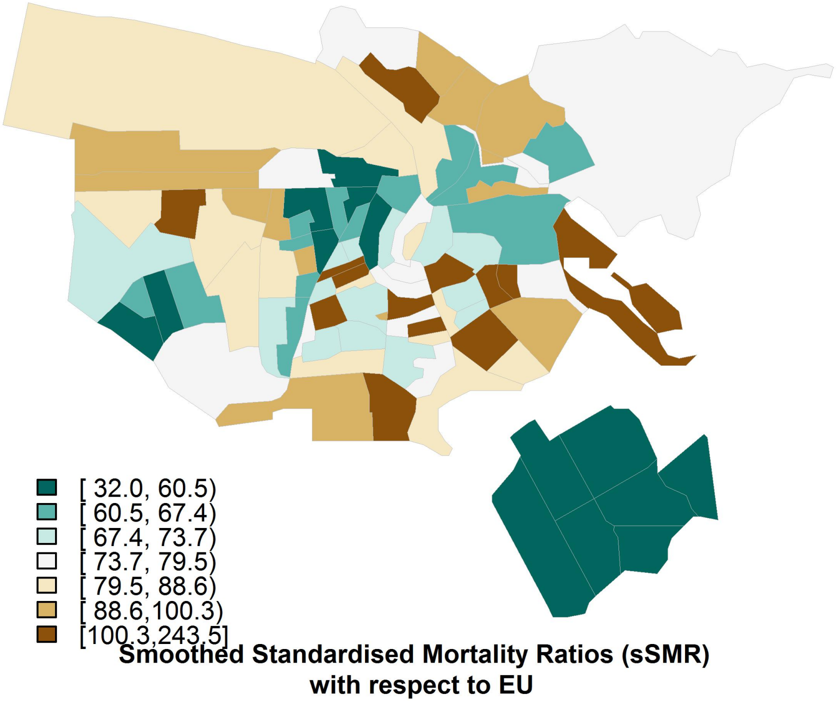

Smoothed Standardised Mortality Ratios (sSMR)
with respect to EU

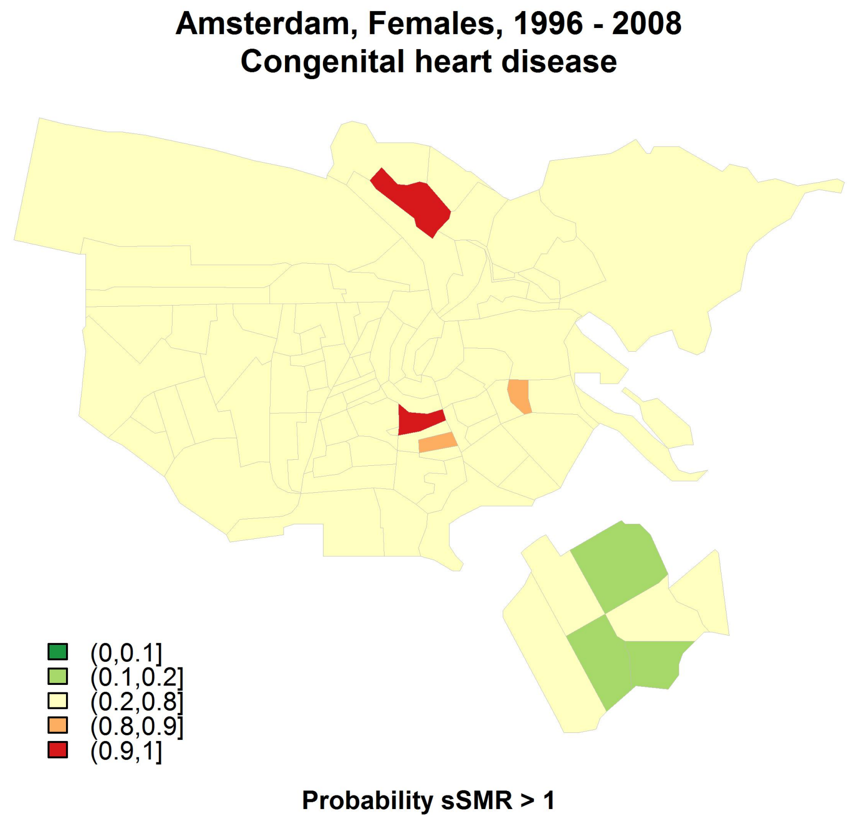
Amsterdam, Females, 1996 - 2008
Congenital heart disease
(0,0.1]
(0.1,0.2]
(0.2,0.8]
(0.8,0.9]
(0.9,1]
Probability sSMR > 1
