# Supplementary material for: Social differences in avoidable mortality between small areas of 15 European cities: an ecological study
Source: Int J Health Geogr. 2014 Mar 12;13:8. doi: 10.1186/1476-072X-13-8 (PMC4007807; doi:10.1186/1476-072X-13-8)
Supplement: Additional file 4 — Cause-specific mortality maps for Barcelona. [file 1476-072X-13-8-S4.pdf]

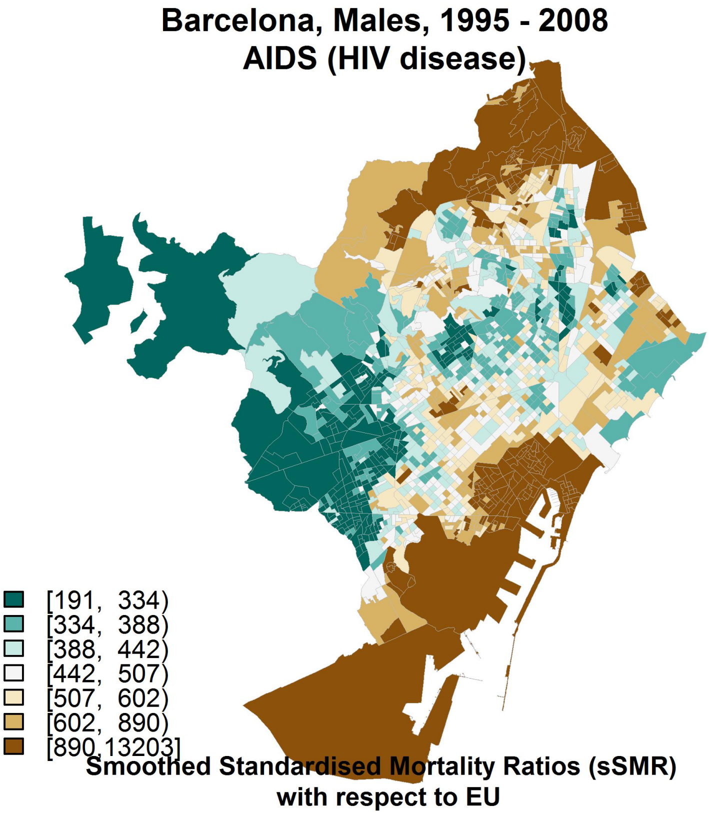
Barcelona, Males, 1995 - 2008
AIDS (HIV disease)
[191, 334)
[334, 388)
[388, 442)
[442, 507)
[507, 602)
[602, 890)
[890,13203]
Smoothed Standardised Mortality Ratios (sSMR)
with respect to EU

Barcelona, Males, 1995 - 2008
AIDS (HIV disease)

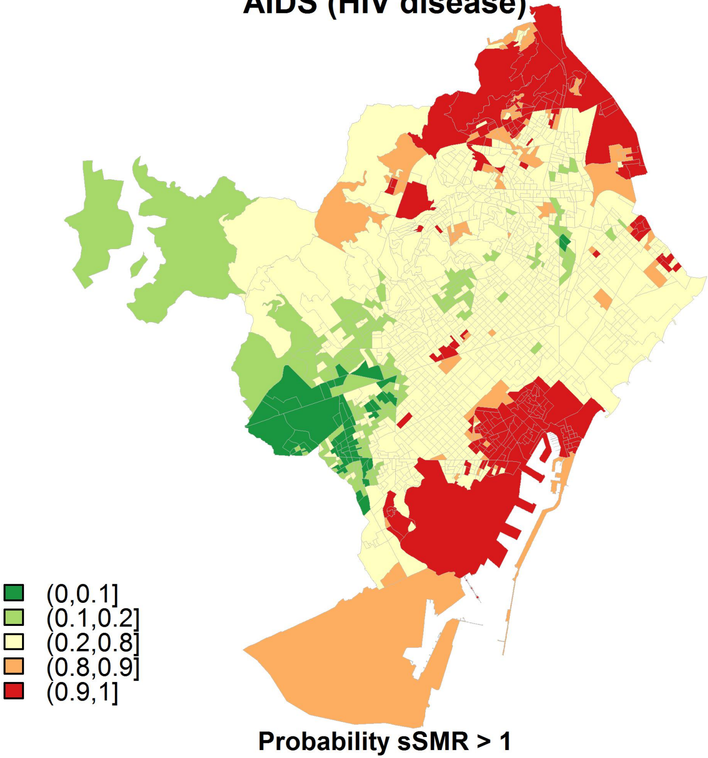

Barcelona, Males, 1995 - 2008
MN colon

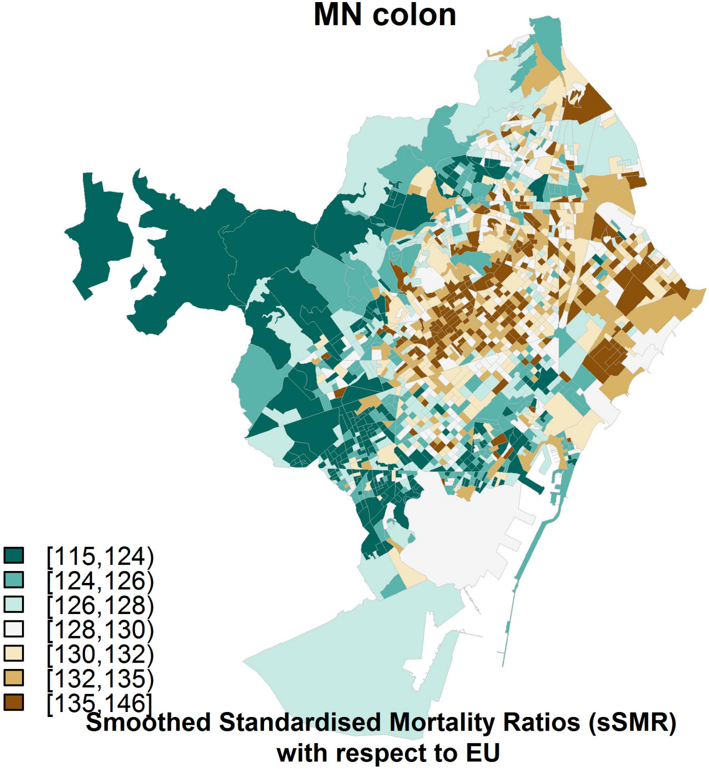

Smoothed Standardised Mortality Ratios (sSMR)
with respect to EU

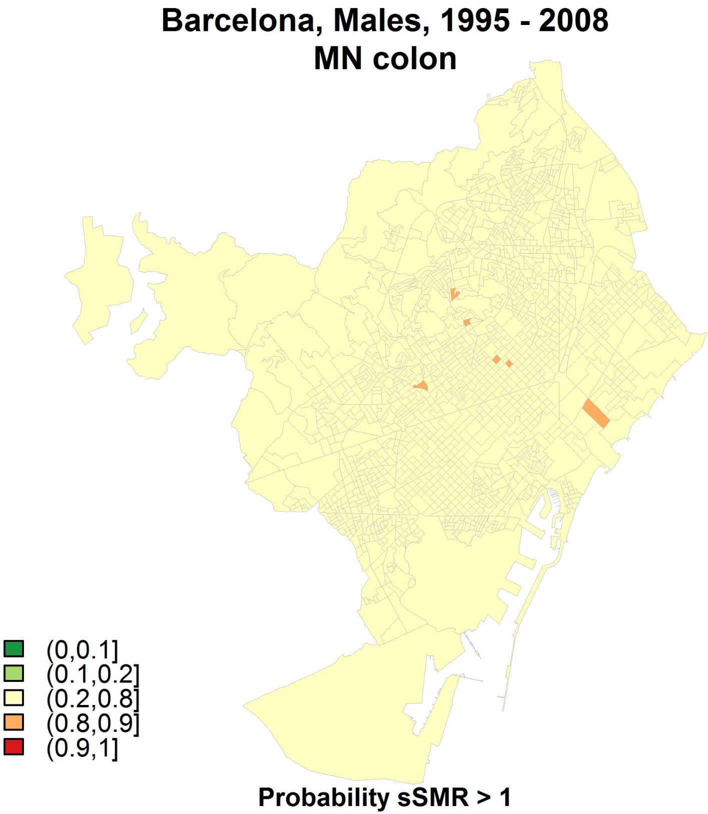

Barcelona, Males, 1995 - 2008
MN colon
(0,0.1]
(0.1,0.2]
(0.2,0.8]
(0.8,0.9]
(0.9,1]
Probability sSMR > 1

Barcelona, Males, 1995 - 2008
MN rectum, anus and anal canal

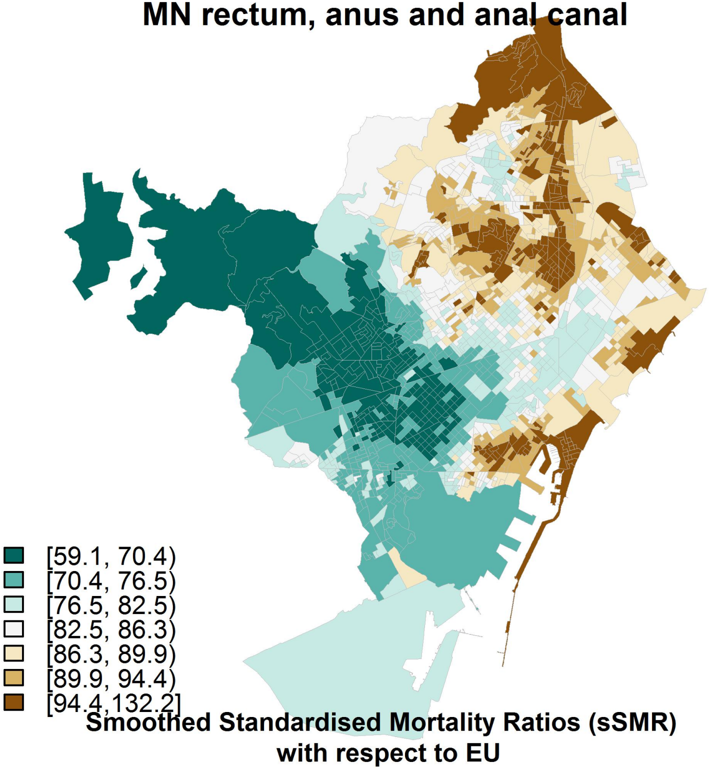

Smoothed Standardised Mortality Ratios (sSMR) with respect to EU

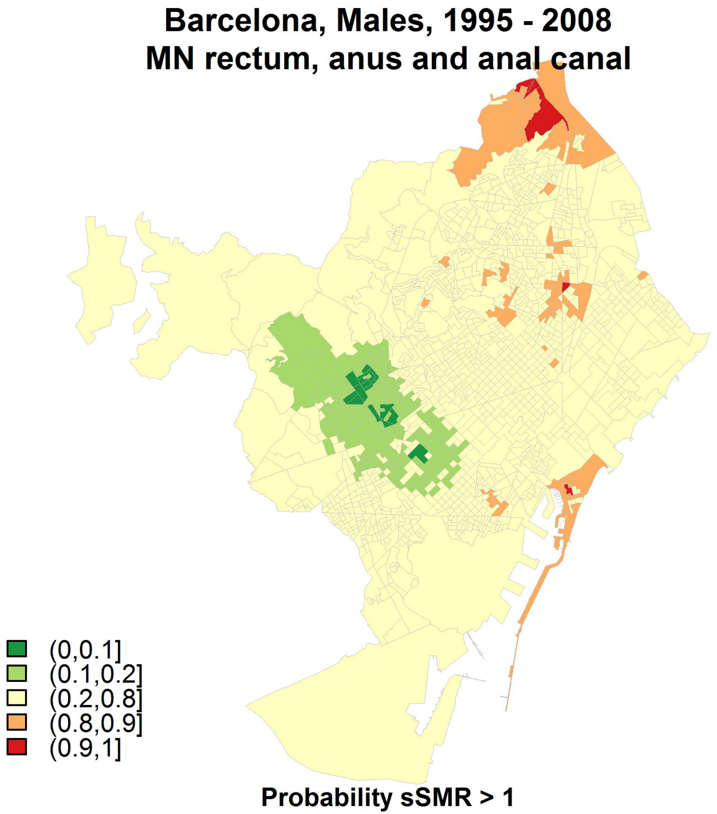
Barcelona, Males, 1995 - 2008
MN rectum, anus and anal canal
(0,0.1]
(0.1,0.2]
(0.2,0.8]
(0.8,0.9]
(0.9,1]
Probability sSMR > 1

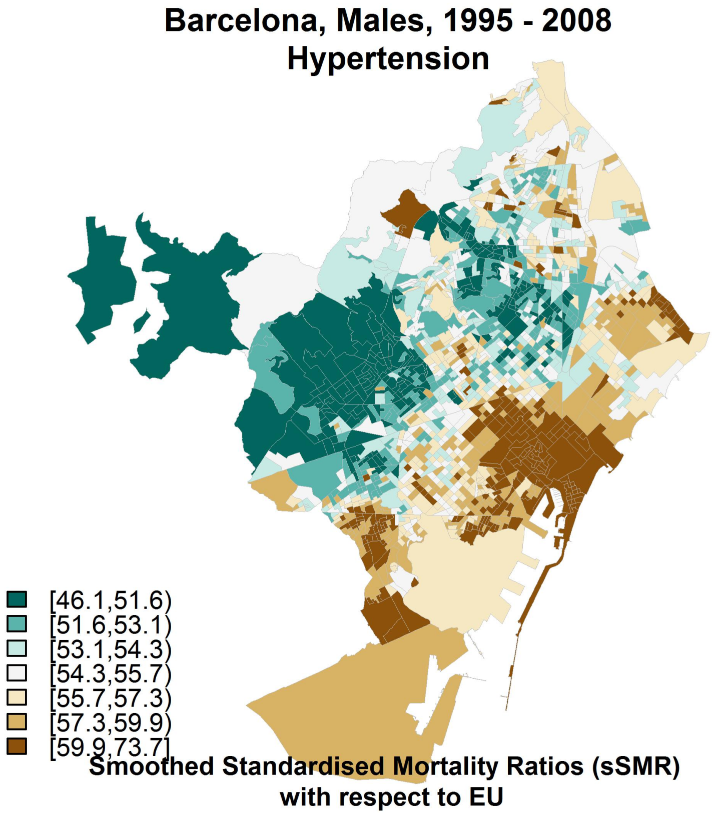
Barcelona, Males, 1995 - 2008
Hypertension
[46.1,51.6)
[51.6,53.1)
[53.1,54.3)
[54.3,55.7)
[55.7,57.3)
[57.3,59.9)
[59.9,73.7]
Smoothed Standardised Mortality Ratios (sSMR)
with respect to EU

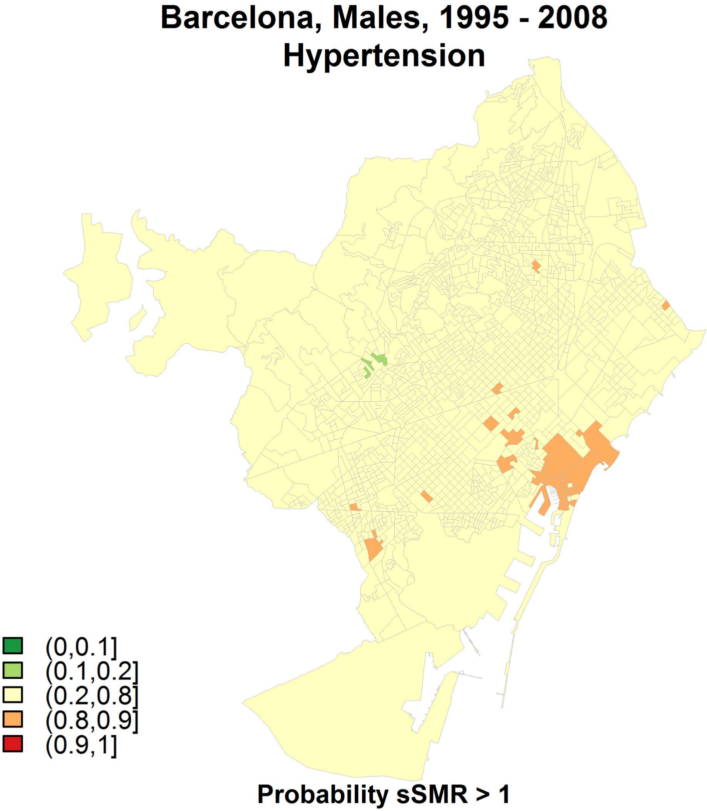

Barcelona, Males, 1995 - 2008
Hypertension
(0,0.1]
(0.1,0.2]
(0.2,0.8]
(0.8,0.9]
(0.9,1]
Probability sSMR > 1

Barcelona, Males, 1995 - 2008
Heart failure

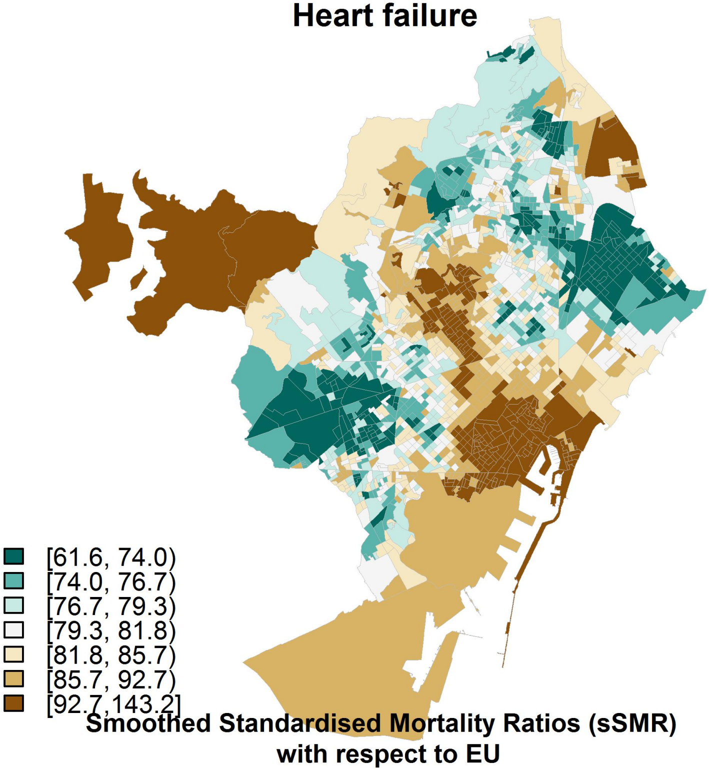

Smoothed Standardised Mortality Ratios (sSMR)
with respect to EU

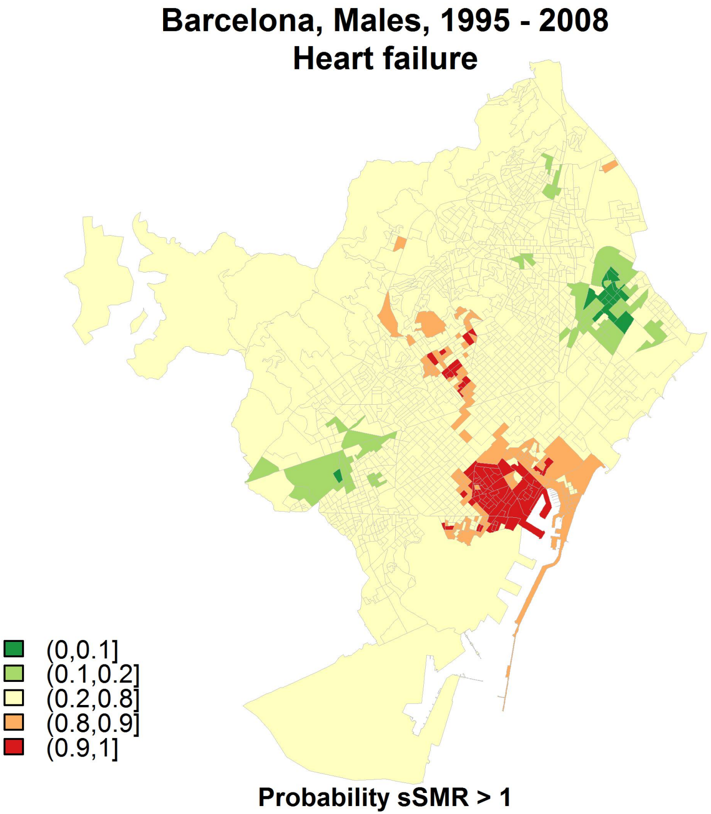
Barcelona, Males, 1995 - 2008
Heart failure
(0,0.1]
(0.1,0.2]
(0.2,0.8]
(0.8,0.9]
(0.9,1]
Probability sSMR > 1

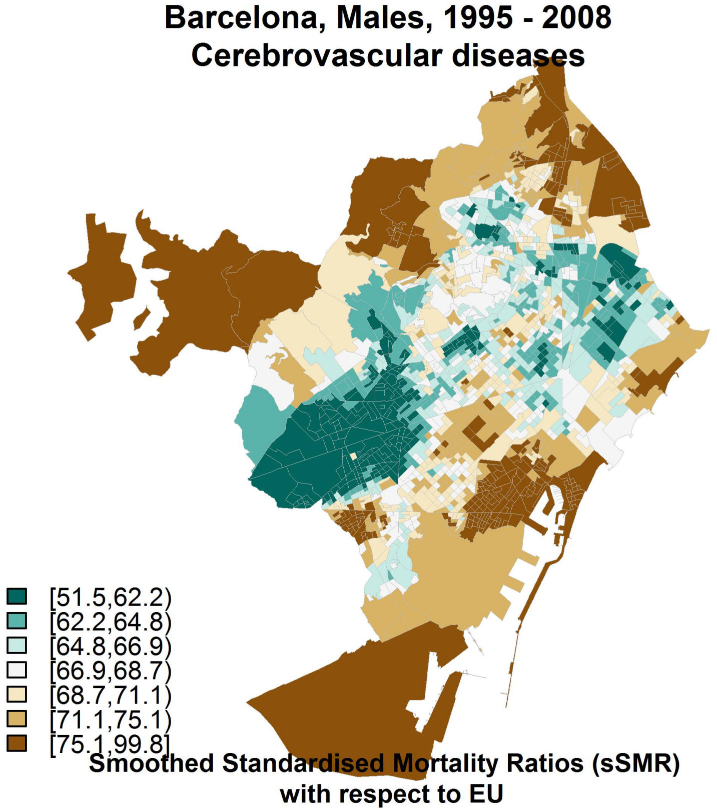

Barcelona, Males, 1995 - 2008
Cerebrovascular diseases
[51.5,62.2)
[62.2,64.8)
[64.8,66.9)
[66.9,68.7)
[68.7,71.1)
[71.1,75.1)
[75.1,99.8]
Smoothed Standardised Mortality Ratios (sSMR)
with respect to EU

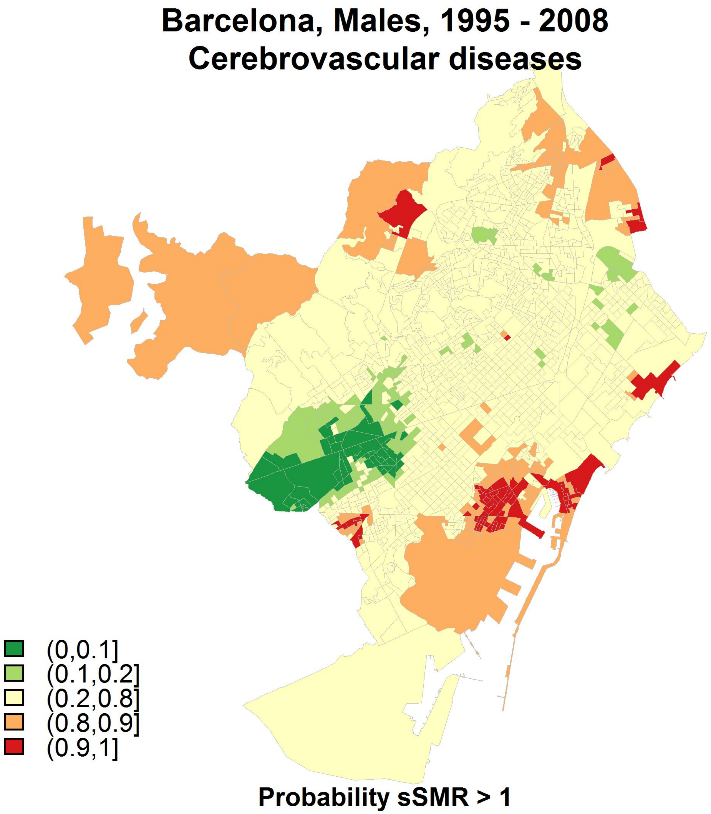
Barcelona, Males, 1995 - 2008
Cerebrovascular diseases
(0,0.1]
(0.1,0.2]
(0.2,0.8]
(0.8,0.9]
(0.9,1]
Probability sSMR > 1

Barcelona, Males, 1995 - 2008
Renal failure

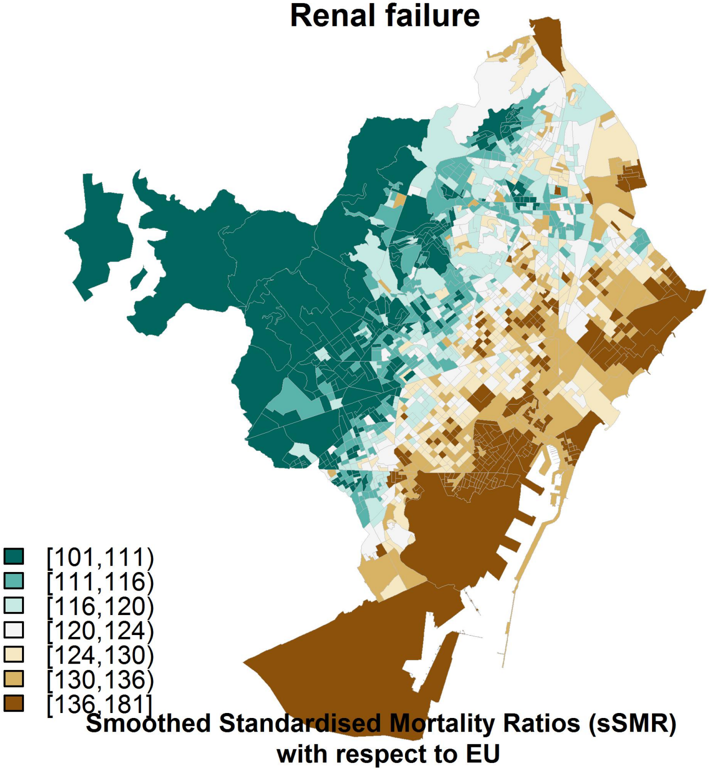

**Smoothed Standardised Mortality Ratios (sSMR)
with respect to EU**

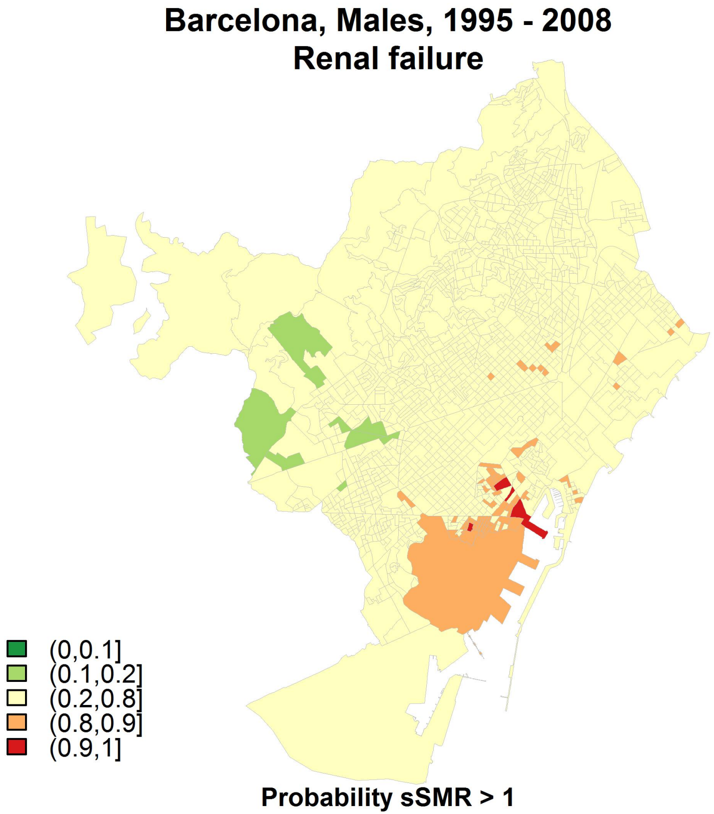
Barcelona, Males, 1995 - 2008
Renal failure
(0,0.1]
(0.1,0.2]
(0.2,0.8]
(0.8,0.9]
(0.9,1]
Probability sSMR > 1

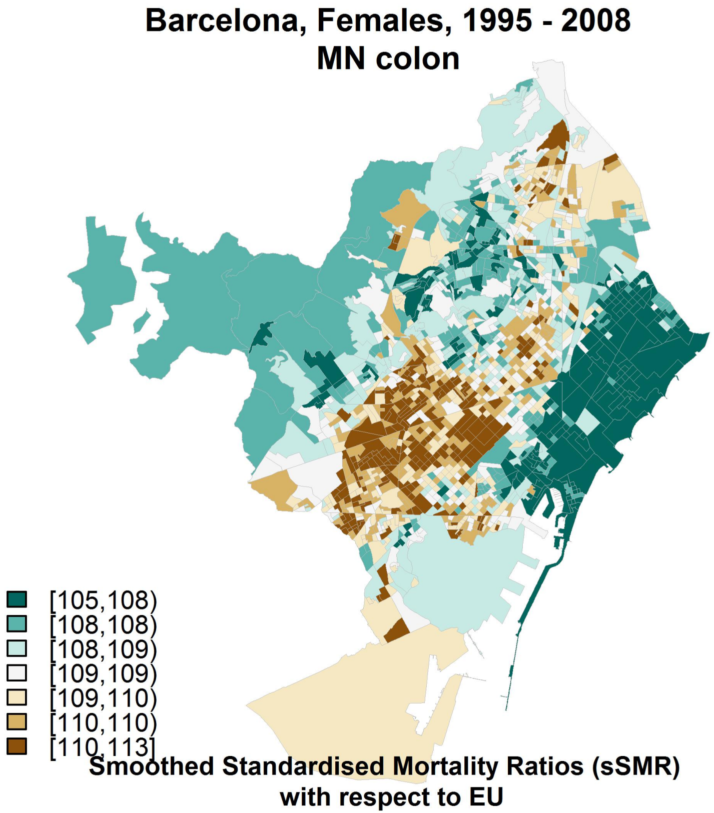
Barcelona, Females, 1995 - 2008
MN colon
[105,108)
[108,108)
[108,109)
[109,109)
[109,110)
[110,110)
[110,113]
Smoothed Standardised Mortality Ratios (sSMR)
with respect to EU

Barcelona, Females, 1995 - 2008
MN colon

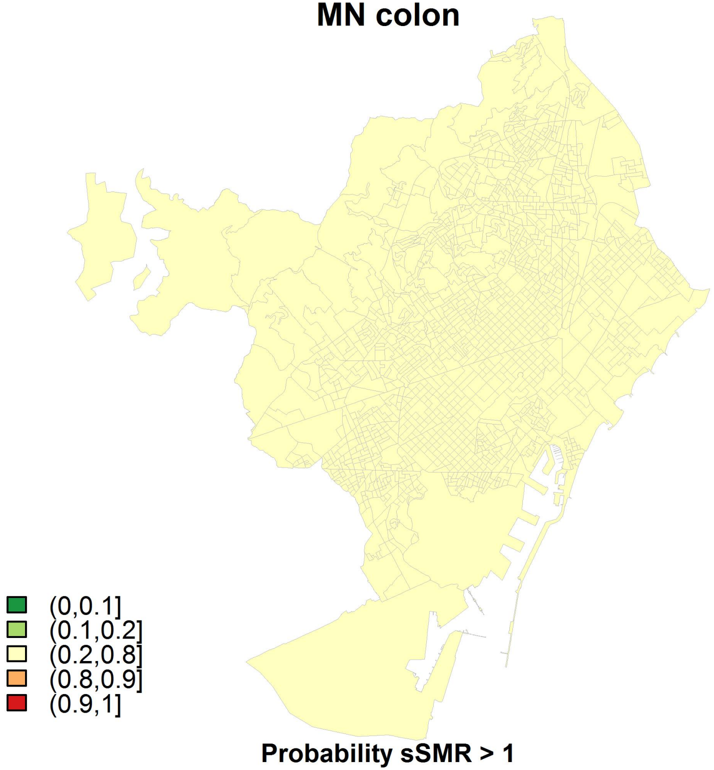

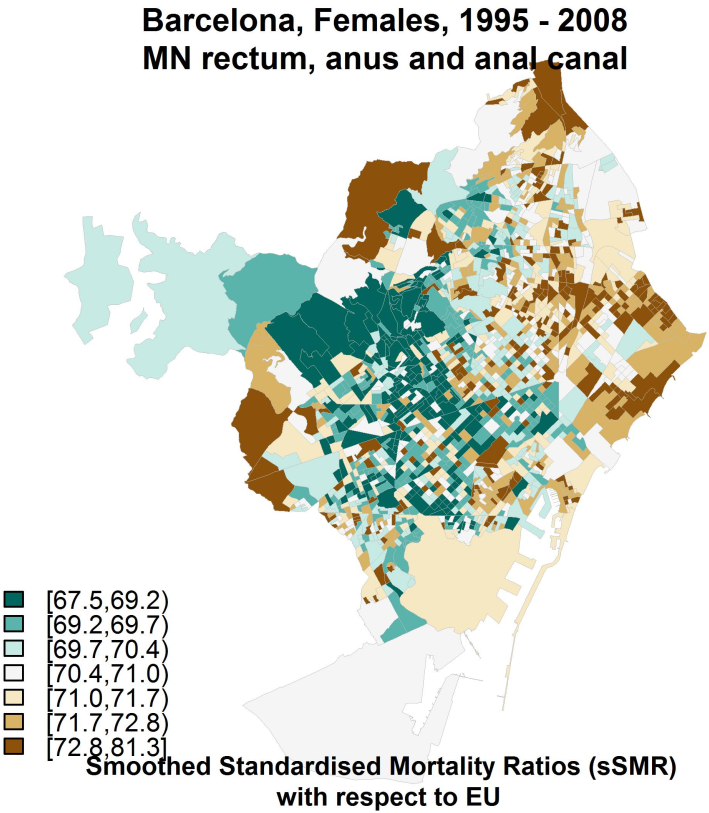
Barcelona, Females, 1995 - 2008
MN rectum, anus and anal canal
[67.5,69.2)
[69.2,69.7)
[69.7,70.4)
[70.4,71.0)
[71.0,71.7)
[71.7,72.8)
[72.8,81.3]
Smoothed Standardised Mortality Ratios (sSMR)
with respect to EU

Barcelona, Females, 1995 - 2008
MN rectum, anus and anal canal

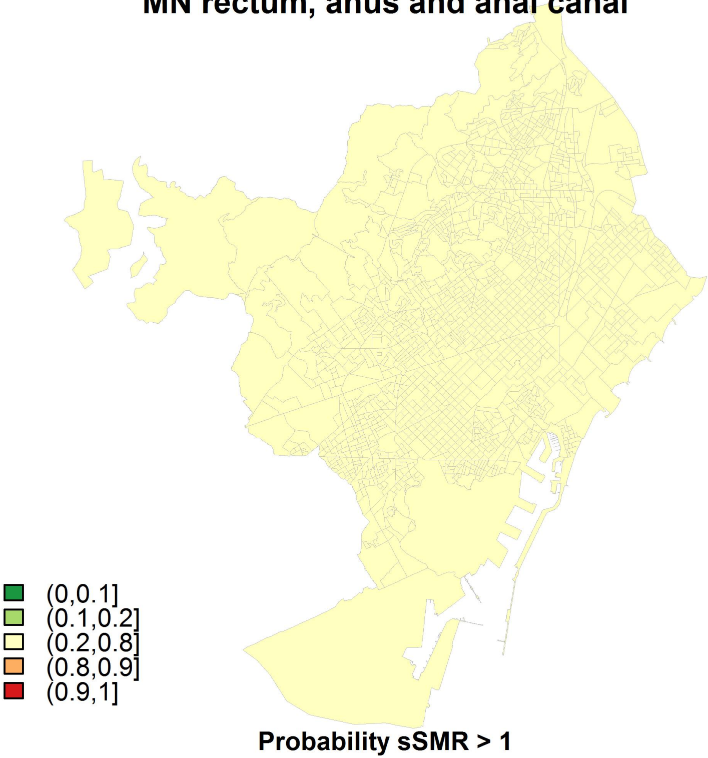

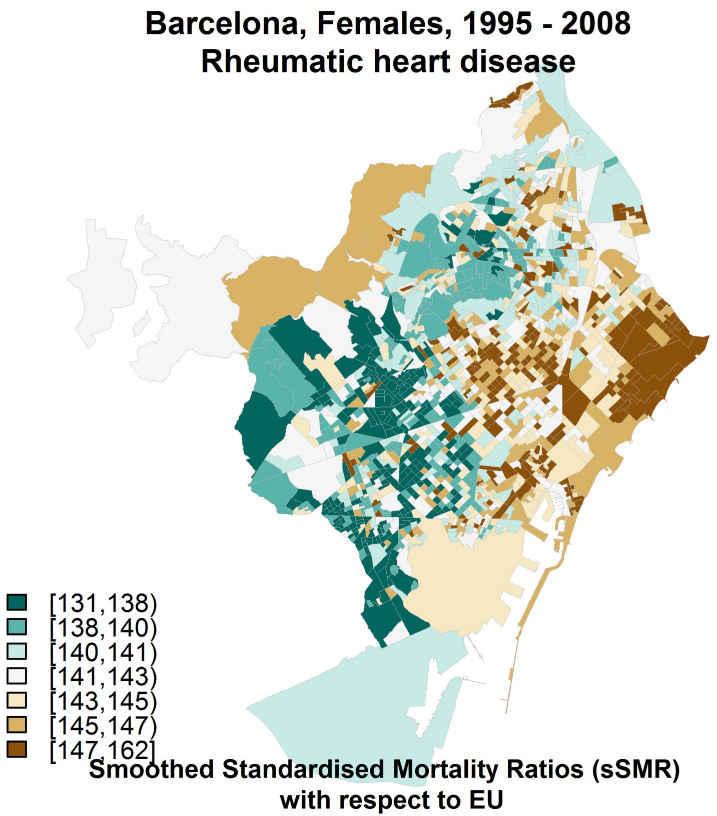
Barcelona, Females, 1995 - 2008
Rheumatic heart disease
[131,138)
[138,140)
[140,141)
[141,143)
[143,145)
[145,147)
[147,162]
Smoothed Standardised Mortality Ratios (sSMR)
with respect to EU

Barcelona, Females, 1995 - 2008
Rheumatic heart disease

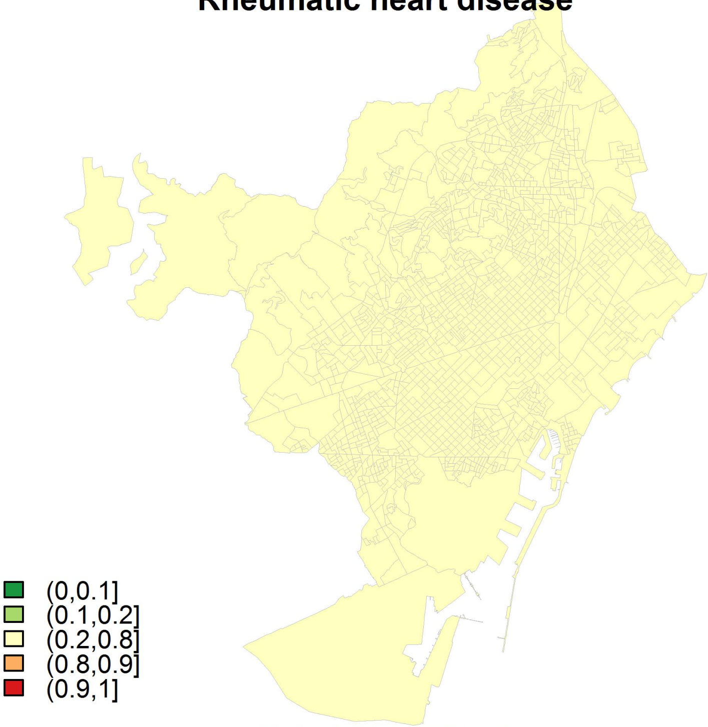

Probability sSMR > 1

Barcelona, Females, 1995 - 2008
Hypertension

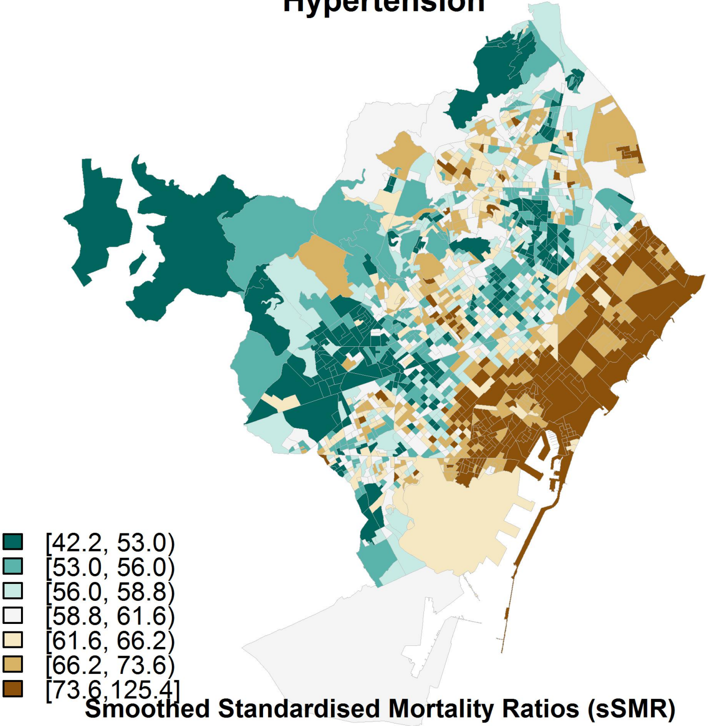

Smoothed Standardised Mortality Ratios (sSMR)
with respect to EU

Barcelona, Females, 1995 - 2008
Hypertension

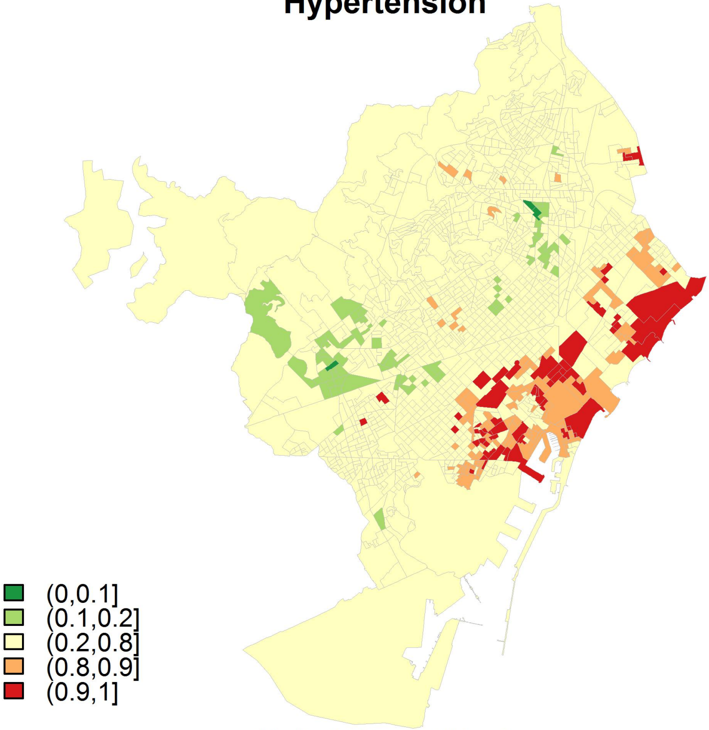

Probability sSMR > 1

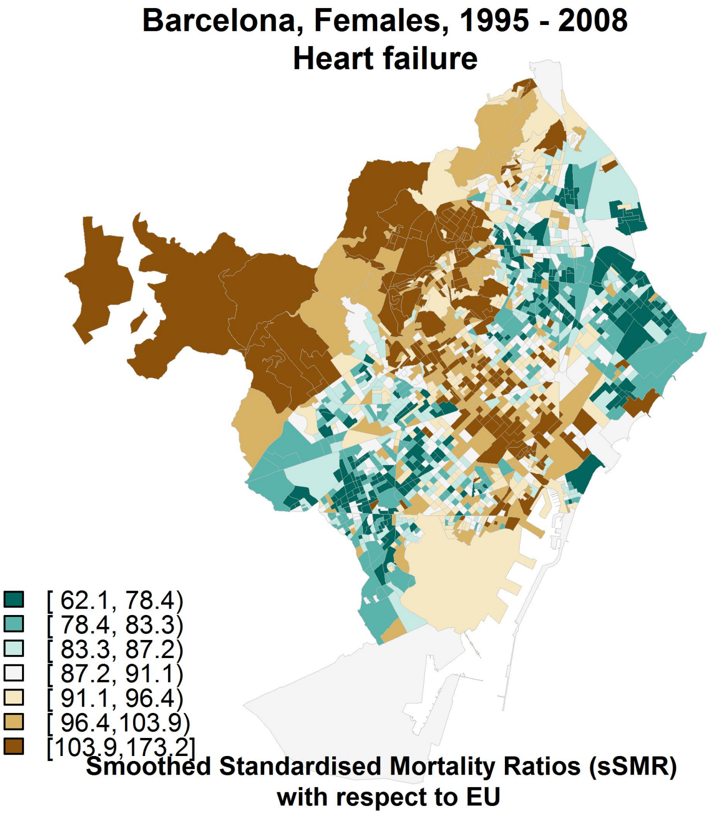
Barcelona, Females, 1995 - 2008
Heart failure
[62.1, 78.4)
[78.4, 83.3)
[83.3, 87.2)
[87.2, 91.1)
[91.1, 96.4)
[96.4,103.9)
[103.9,173.2]
Smoothed Standardised Mortality Ratios (sSMR)
with respect to EU

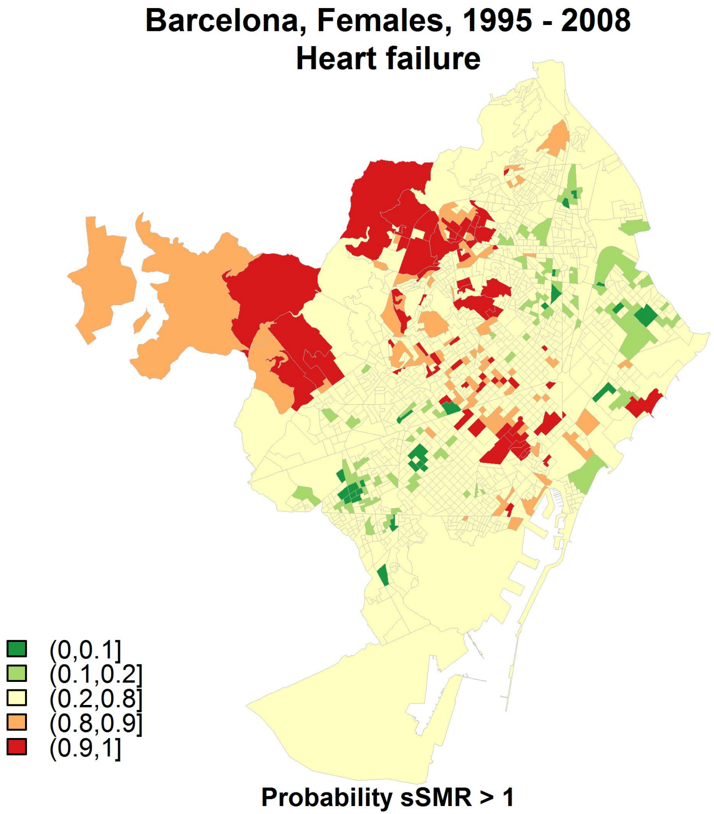
Barcelona, Females, 1995 - 2008
Heart failure
(0,0.1]
(0.1,0.2]
(0.2,0.8]
(0.8,0.9]
(0.9,1]
Probability sSMR > 1

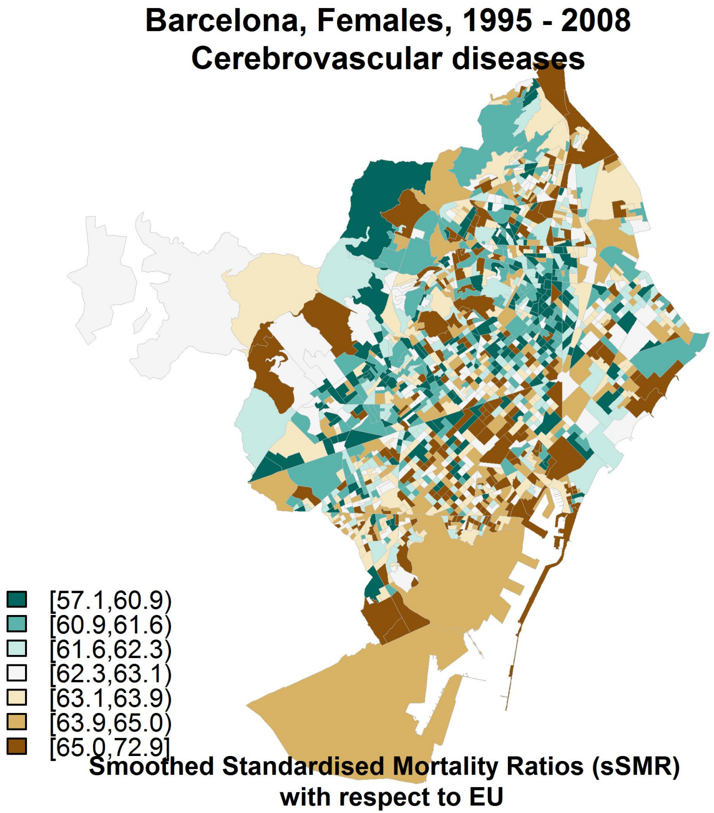
Barcelona, Females, 1995 - 2008
Cerebrovascular diseases
[57.1,60.9)
[60.9,61.6)
[61.6,62.3)
[62.3,63.1)
[63.1,63.9)
[63.9,65.0)
[65.0,72.9]
Smoothed Standardised Mortality Ratios (sSMR)
with respect to EU

Barcelona, Females, 1995 - 2008
Cerebrovascular diseases

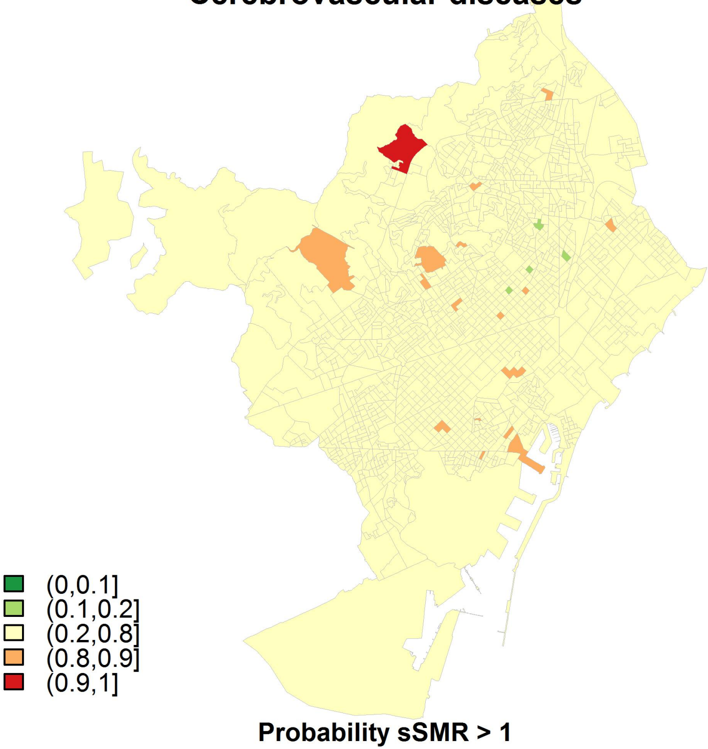

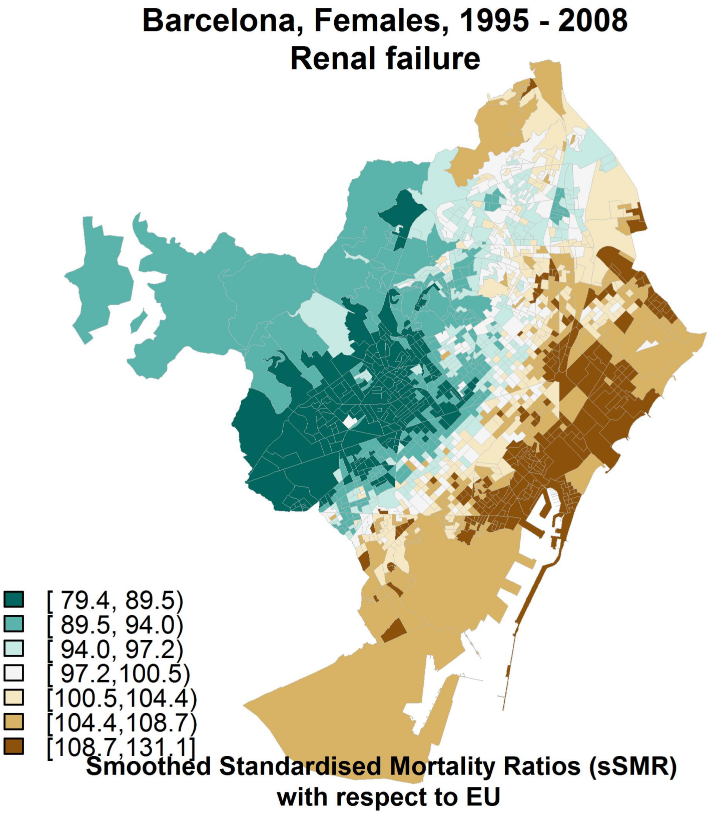
Barcelona, Females, 1995 - 2008
Renal failure
[79.4, 89.5)
[89.5, 94.0)
[94.0, 97.2)
[97.2,100.5)
[100.5,104.4)
[104.4,108.7)
[108.7,131.1]
Smoothed Standardised Mortality Ratios (sSMR)
with respect to EU

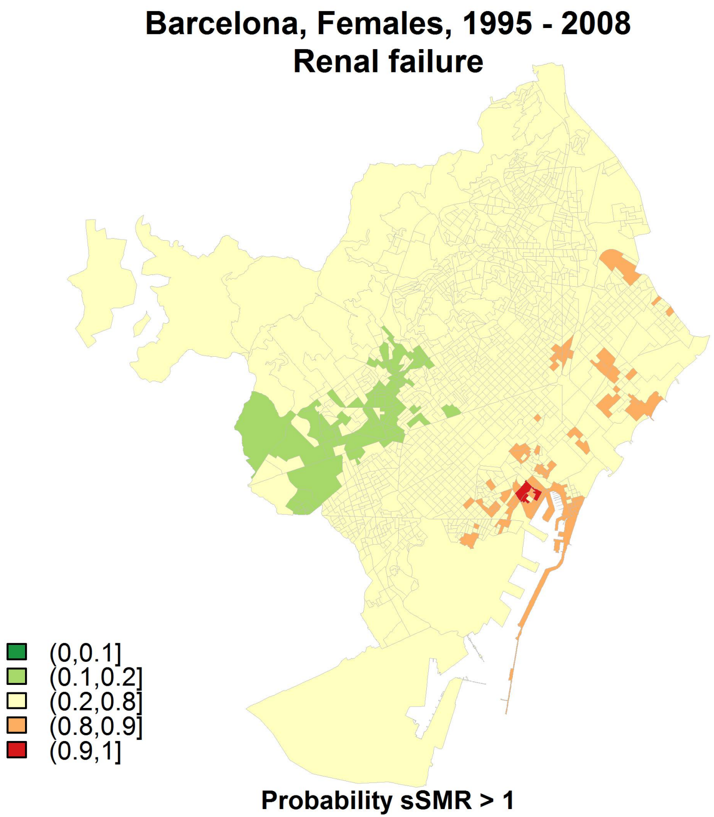
Barcelona, Females, 1995 - 2008
Renal failure
(0,0.1]
(0.1,0.2]
(0.2,0.8]
(0.8,0.9]
(0.9,1]
Probability sSMR > 1
