# Supplementary material for: Social differences in avoidable mortality between small areas of 15 European cities: an ecological study
Source: Int J Health Geogr. 2014 Mar 12;13:8. doi: 10.1186/1476-072X-13-8 (PMC4007807; doi:10.1186/1476-072X-13-8)

Bratislava, Males, 1996 - 2008
MN colon

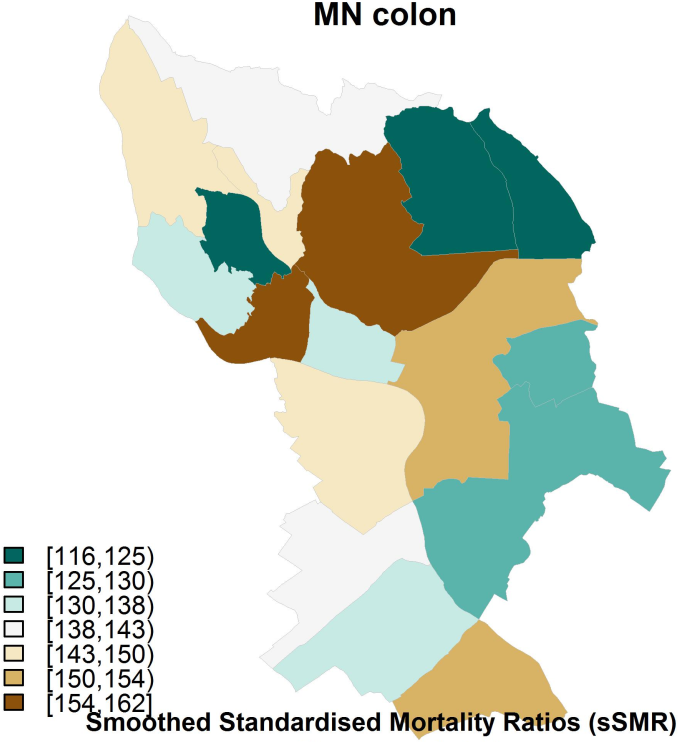

Smoothed Standardised Mortality Ratios (sSMR) with respect to EU

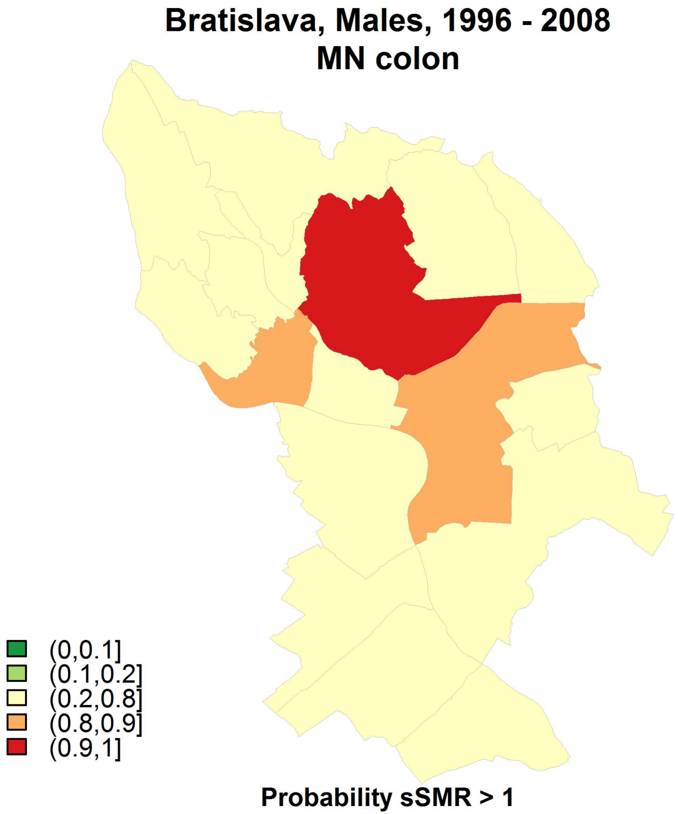
Bratislava, Males, 1996 - 2008
MN colon
(0,0.1]
(0.1,0.2]
(0.2,0.8]
(0.8,0.9]
(0.9,1]
Probability sSMR > 1

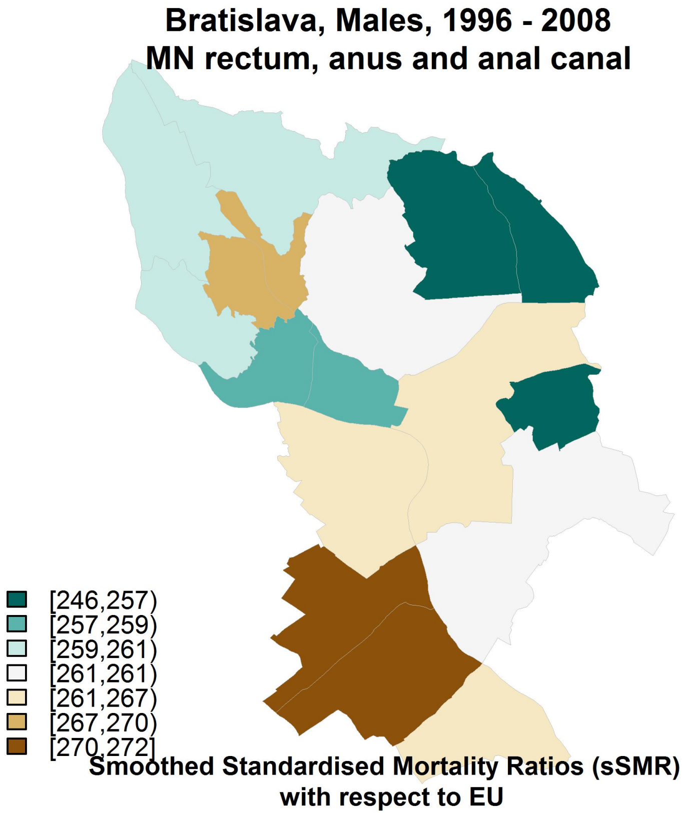
Bratislava, Males, 1996 - 2008
MN rectum, anus and anal canal
[246,257)
[257,259)
[259,261)
[261,261)
[261,267)
[267,270)
[270,272]
Smoothed Standardised Mortality Ratios (sSMR)
with respect to EU

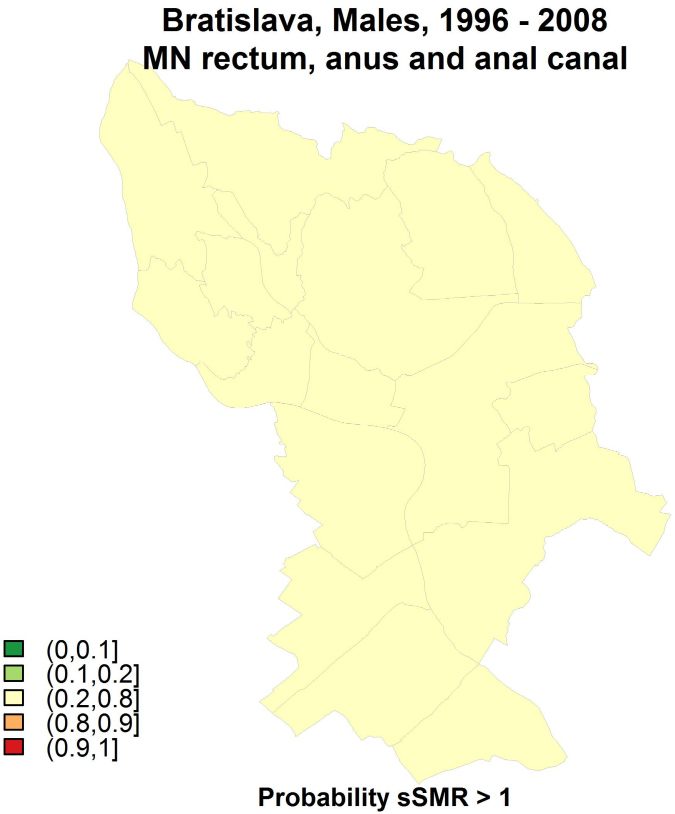
Bratislava, Males, 1996 - 2008
MN rectum, anus and anal canal
(0,0.1]
(0.1,0.2]
(0.2,0.8]
(0.8,0.9]
(0.9,1]
Probability sSMR > 1

Bratislava, Males, 1996 - 2008
MN testes

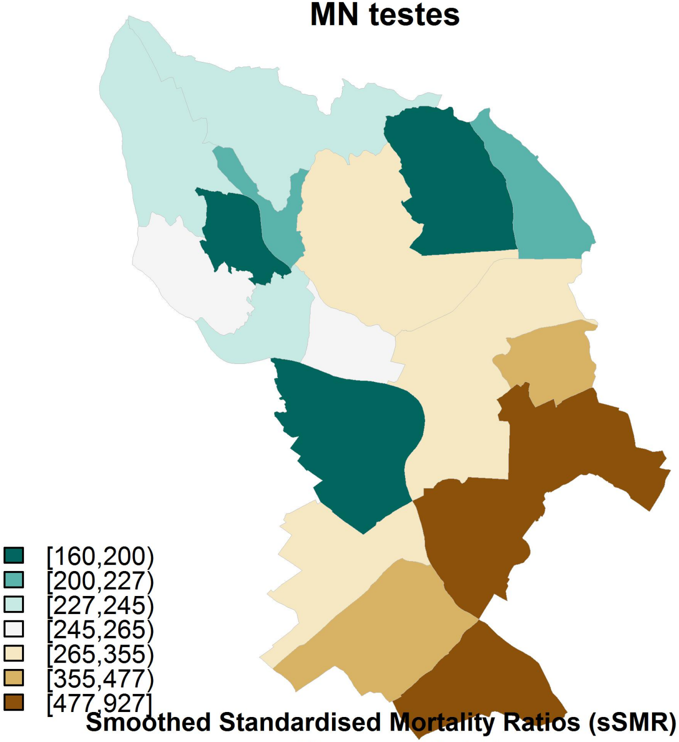

Smoothed Standardised Mortality Ratios (sSMR) with respect to EU

Bratislava, Males, 1996 - 2008
MN testes

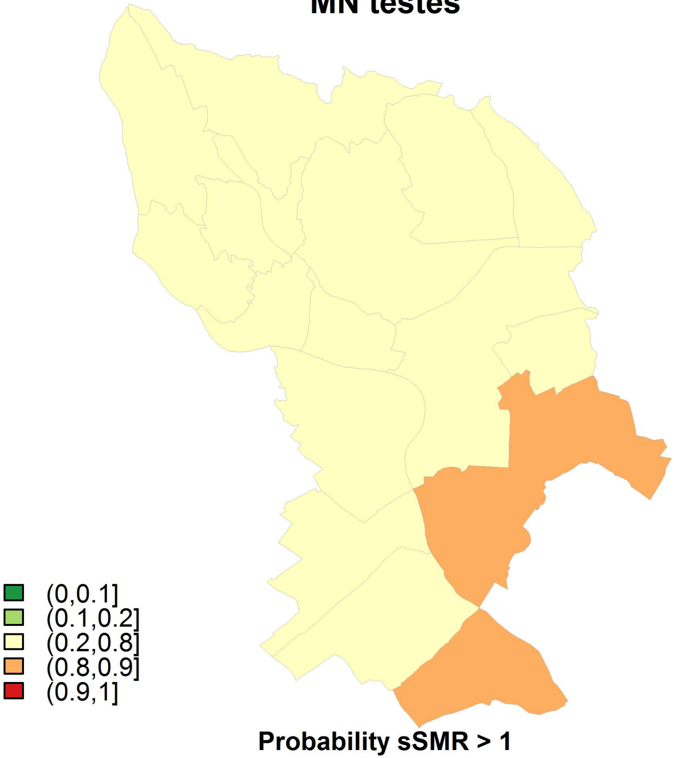

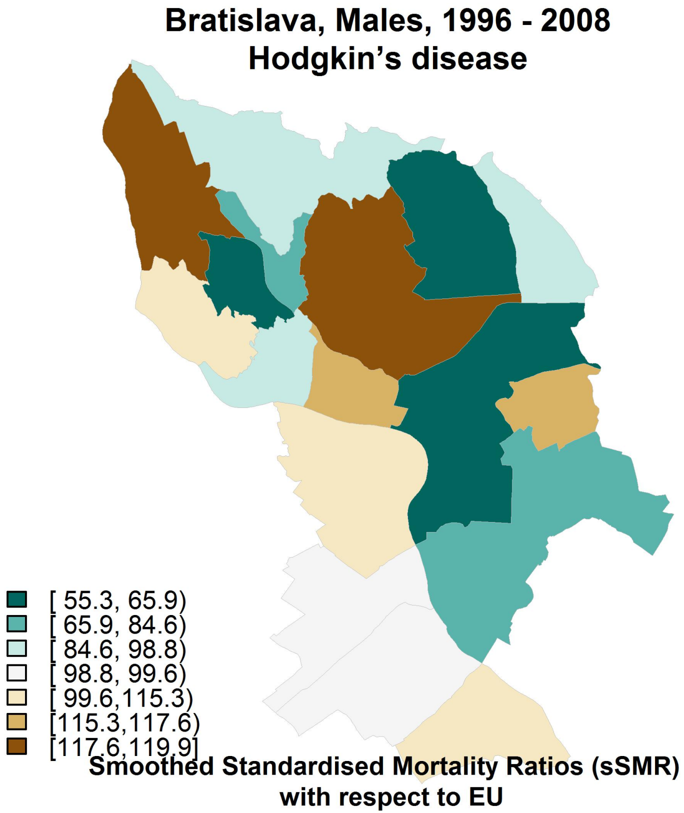
Bratislava, Males, 1996 - 2008
Hodgkin's disease
[55.3, 65.9)
[65.9, 84.6)
[84.6, 98.8)
[98.8, 99.6)
[99.6,115.3)
[115.3,117.6)
[117.6,119.9]
Smoothed Standardised Mortality Ratios (sSMR)
with respect to EU

Bratislava, Males, 1996 - 2008
Hodgkin’s disease

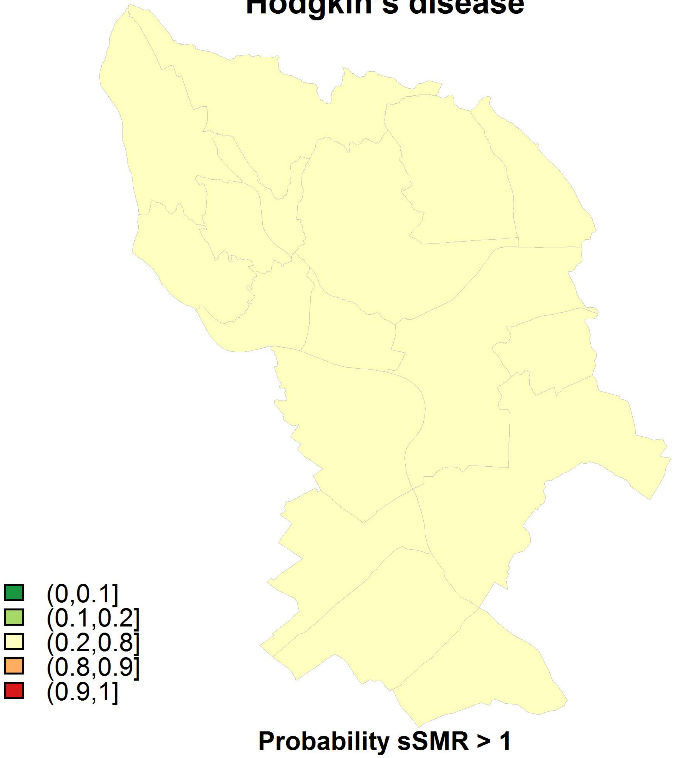

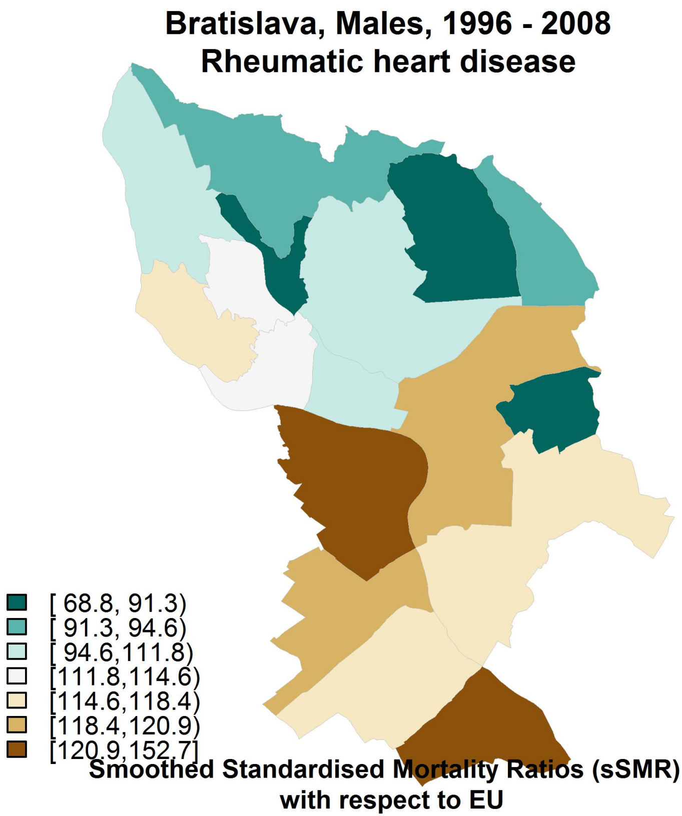
Bratislava, Males, 1996 - 2008
Rheumatic heart disease
[68.8, 91.3)
[91.3, 94.6)
[94.6,111.8)
[111.8,114.6)
[114.6,118.4)
[118.4,120.9)
[120.9,152.7]
Smoothed Standardised Mortality Ratios (sSMR)
with respect to EU

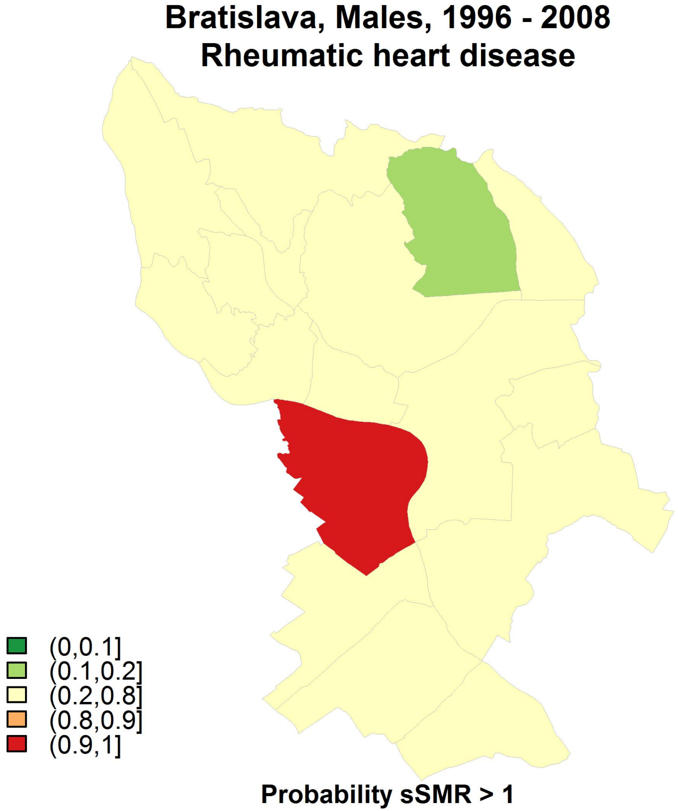
Bratislava, Males, 1996 - 2008
Rheumatic heart disease
(0,0.1]
(0.1,0.2]
(0.2,0.8]
(0.8,0.9]
(0.9,1]
Probability sSMR > 1

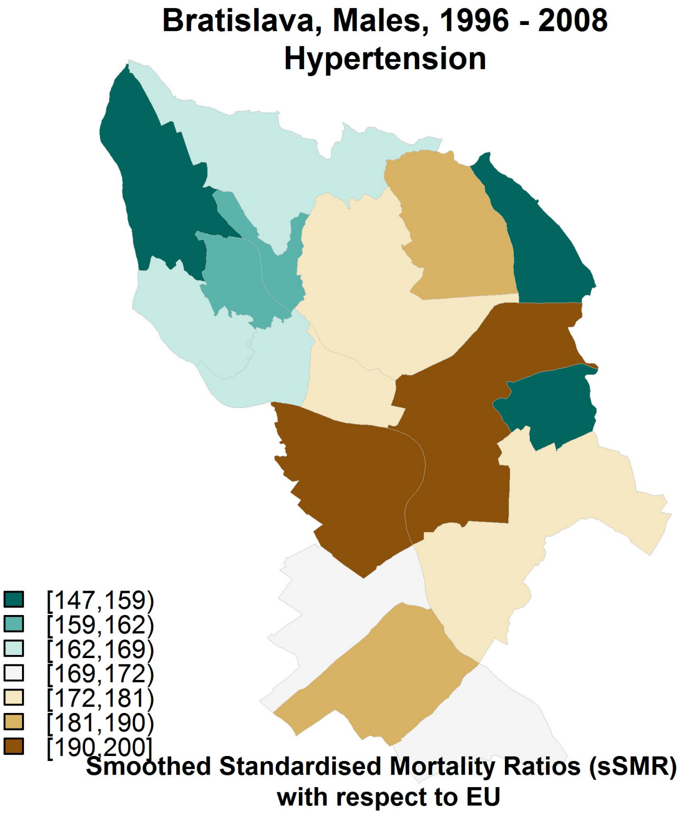
Bratislava, Males, 1996 - 2008
Hypertension
[147,159)
[159,162)
[162,169)
[169,172)
[172,181)
[181,190)
[190,200]
Smoothed Standardised Mortality Ratios (sSMR)
with respect to EU

Bratislava, Males, 1996 - 2008
Hypertension

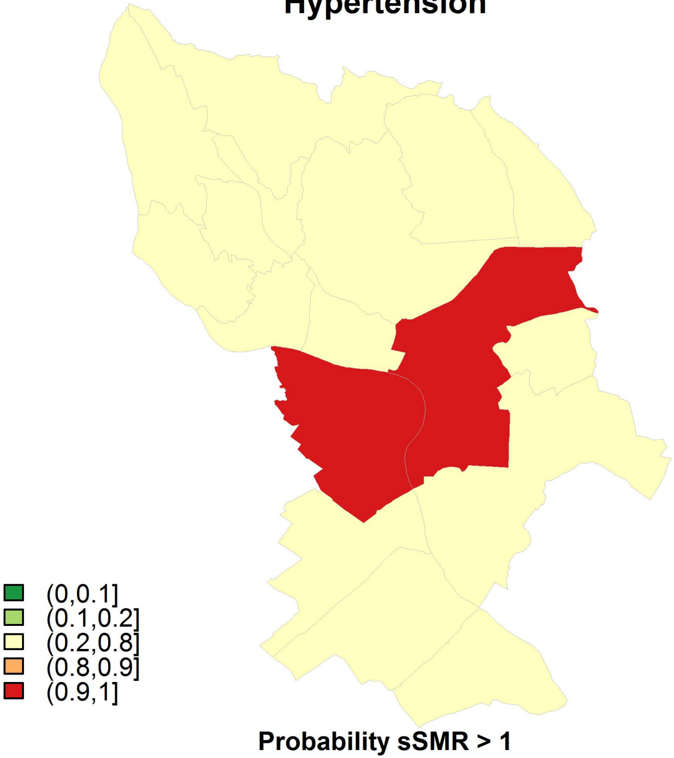

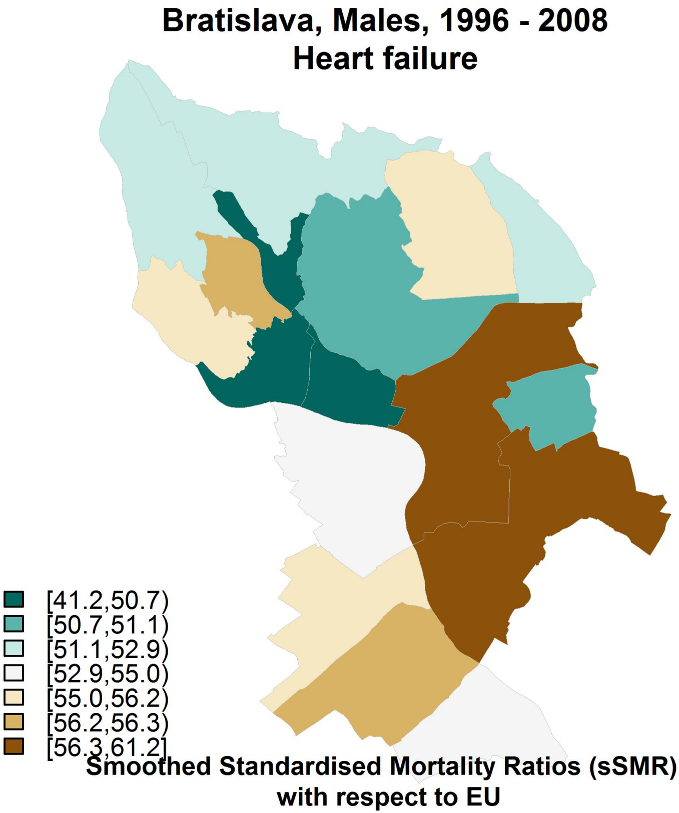
Bratislava, Males, 1996 - 2008
Heart failure
[41.2,50.7)
[50.7,51.1)
[51.1,52.9)
[52.9,55.0)
[55.0,56.2)
[56.2,56.3)
[56.3,61.2]
Smoothed Standardised Mortality Ratios (sSMR)
with respect to EU

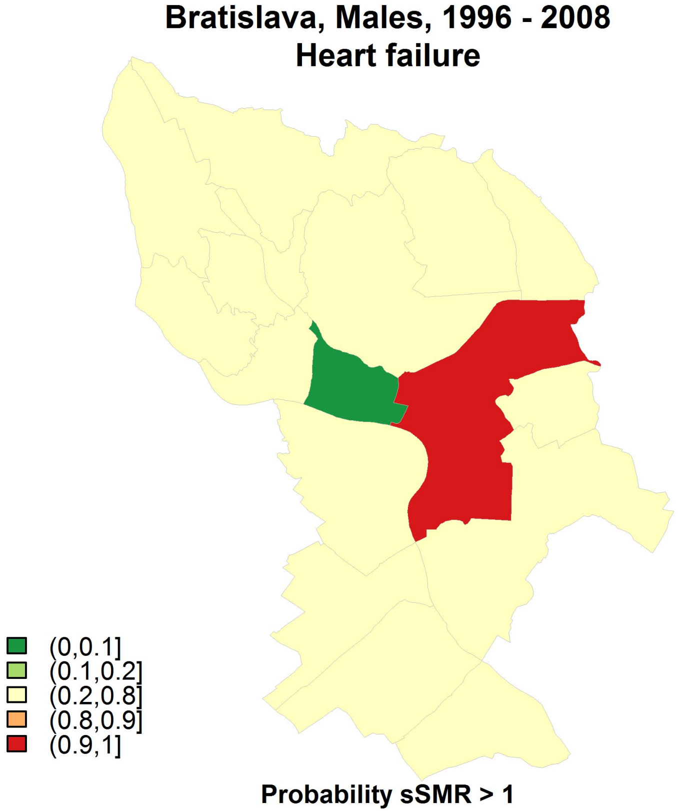

Bratislava, Males, 1996 - 2008
Heart failure
(0,0.1]
(0.1,0.2]
(0.2,0.8]
(0.8,0.9]
(0.9,1]
Probability sSMR > 1

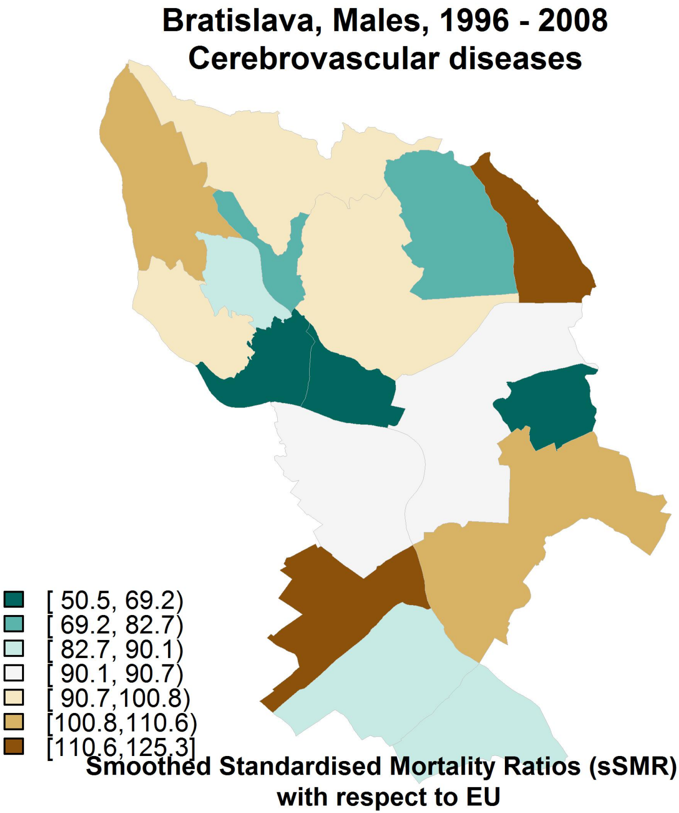
Bratislava, Males, 1996 - 2008
Cerebrovascular diseases
[50.5, 69.2)
[69.2, 82.7)
[82.7, 90.1)
[90.1, 90.7)
[90.7,100.8)
[100.8,110.6)
[110.6,125.3]
Smoothed Standardised Mortality Ratios (sSMR)
with respect to EU

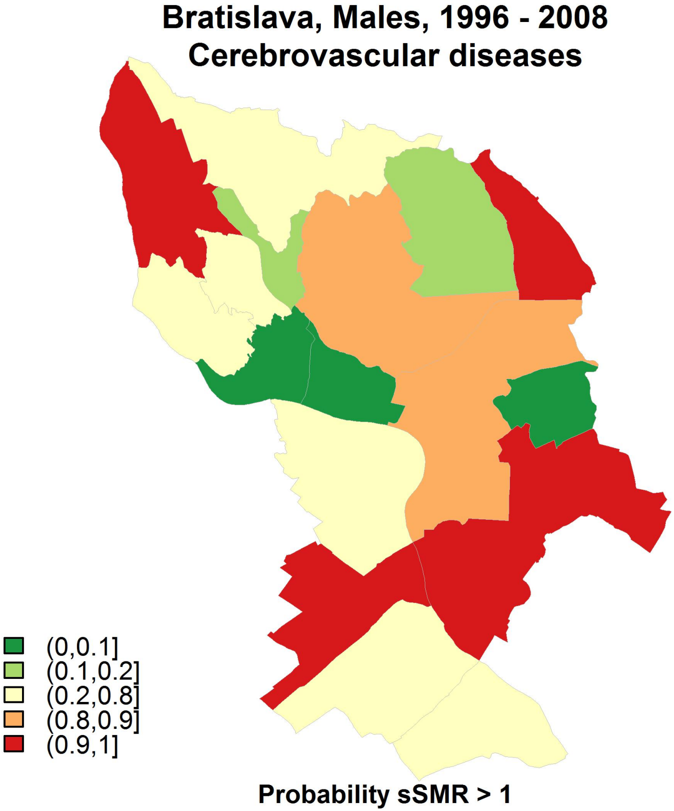
Bratislava, Males, 1996 - 2008
Cerebrovascular diseases
(0,0.1]
(0.1,0.2]
(0.2,0.8]
(0.8,0.9]
(0.9,1]
Probability sSMR > 1

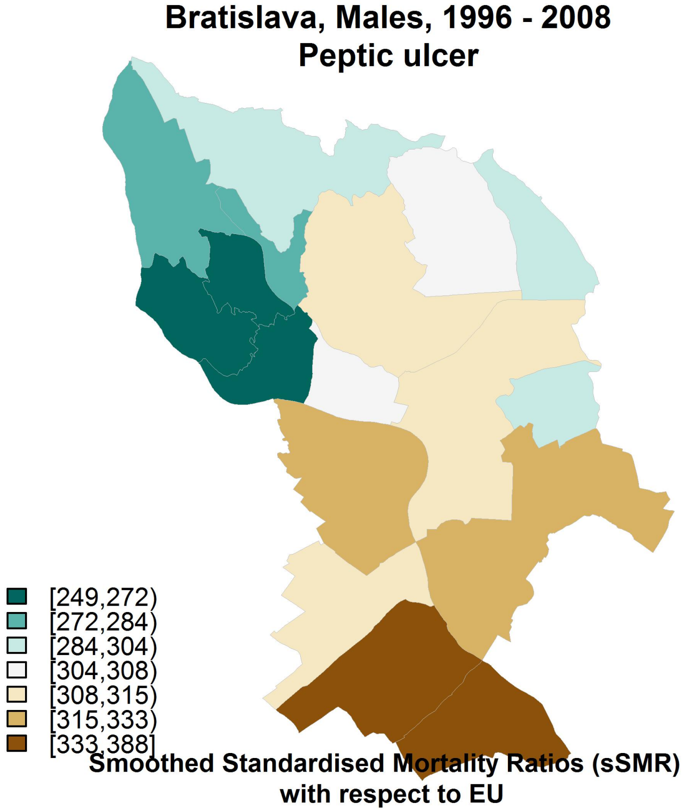
Bratislava, Males, 1996 - 2008
Peptic ulcer
[249,272)
[272,284)
[284,304)
[304,308)
[308,315)
[315,333)
[333,388]
Smoothed Standardised Mortality Ratios (sSMR)
with respect to EU

Bratislava, Males, 1996 - 2008
Peptic ulcer

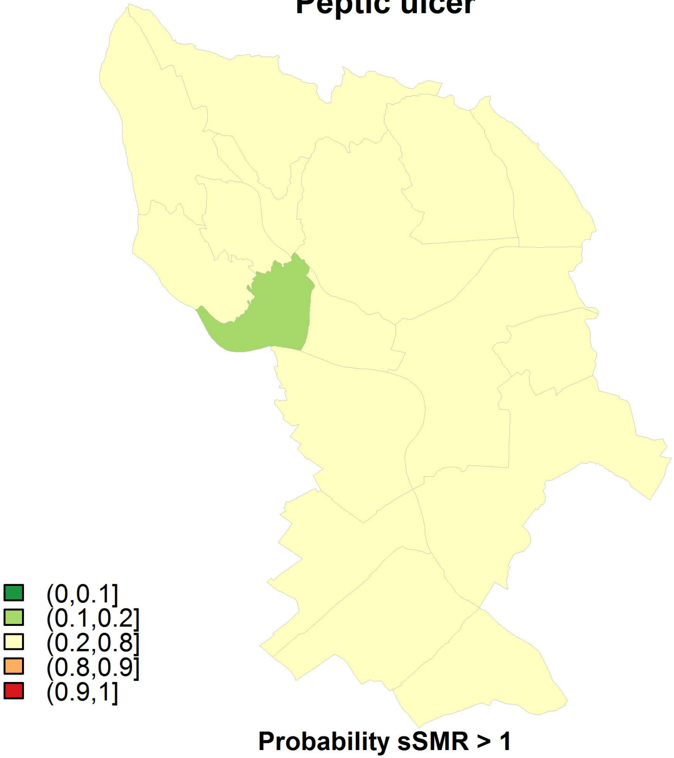

Bratislava, Males, 1996 - 2008
Renal failure

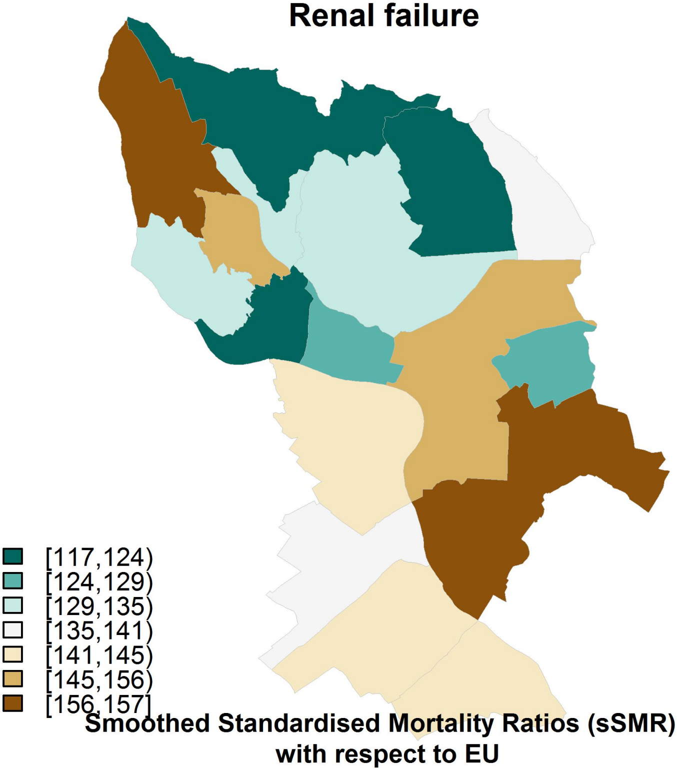

Smoothed Standardised Mortality Ratios (sSMR)
with respect to EU

Bratislava, Males, 1996 - 2008
Renal failure

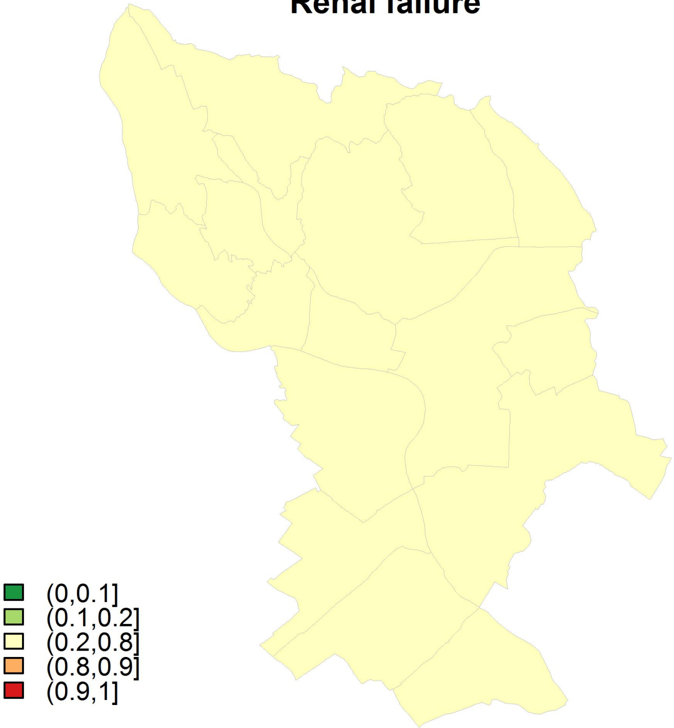

Probability sSMR > 1

Bratislava, Males, 1996 - 2008
Conditions originating in the perinatal period

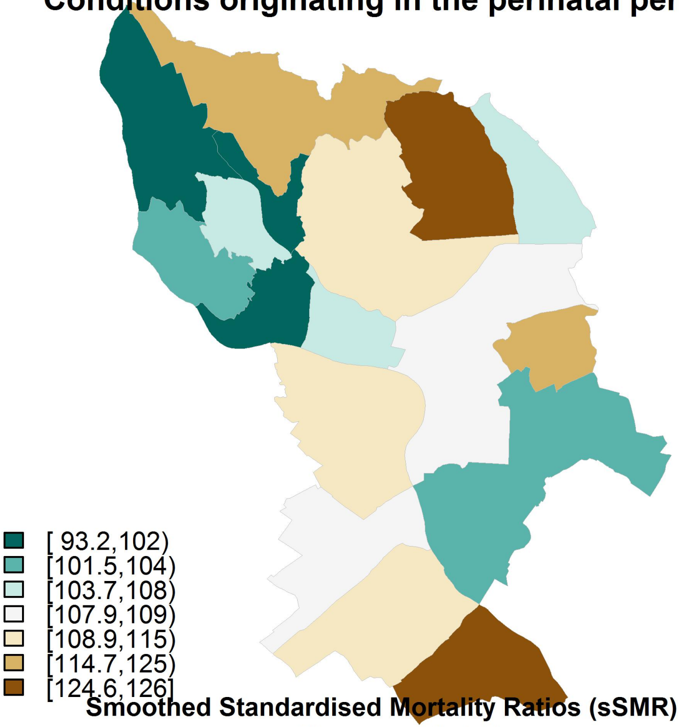

Smoothed Standardised Mortality Ratios (sSMR)
with respect to EU

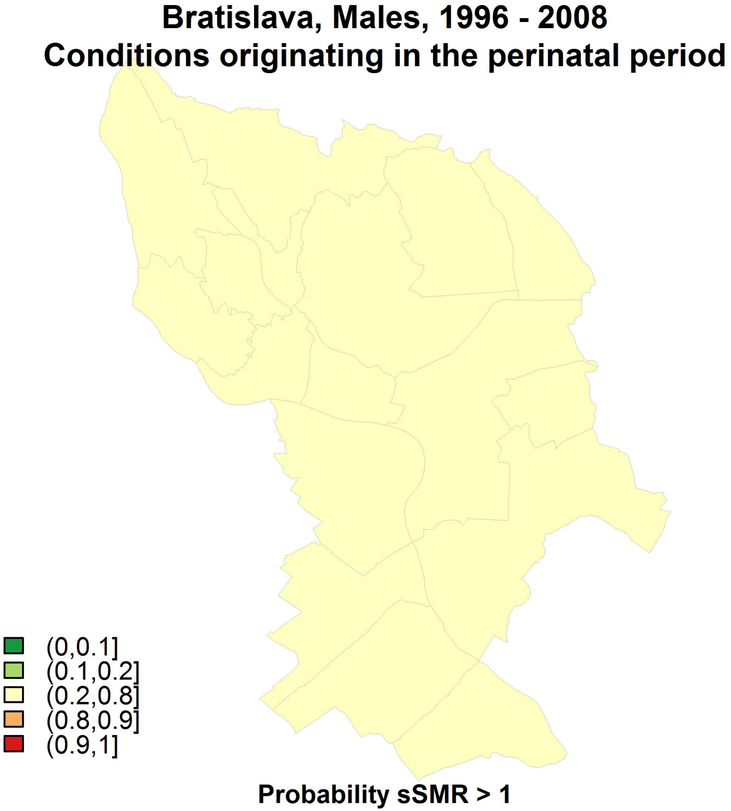
Bratislava, Males, 1996 - 2008
Conditions originating in the perinatal period
(0,0.1]
(0.1,0.2]
(0.2,0.8]
(0.8,0.9]
(0.9,1]
Probability sSMR > 1

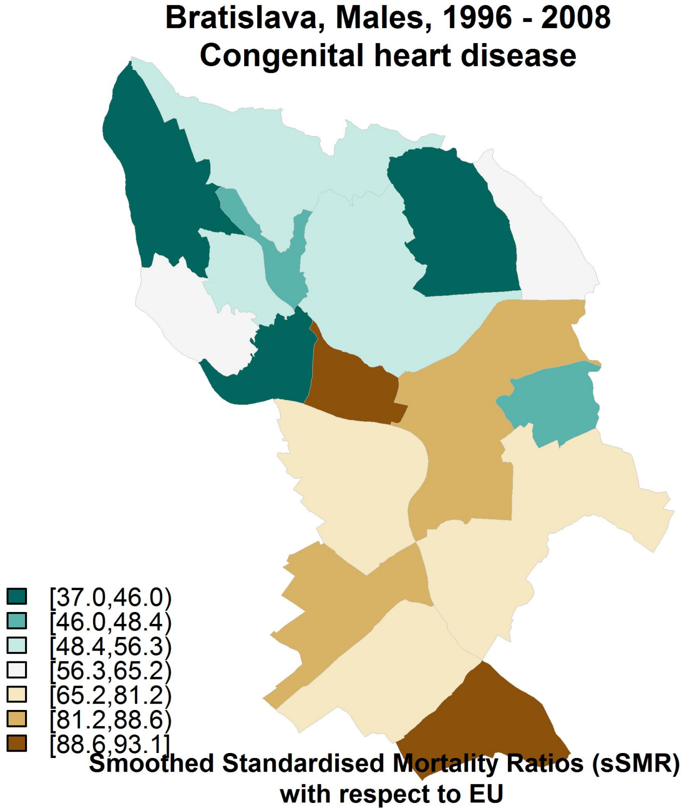
Bratislava, Males, 1996 - 2008
Congenital heart disease
[37.0,46.0)
[46.0,48.4)
[48.4,56.3)
[56.3,65.2)
[65.2,81.2)
[81.2,88.6)
[88.6,93.1]
Smoothed Standardised Mortality Ratios (sSMR)
with respect to EU

Bratislava, Males, 1996 - 2008
Congenital heart disease

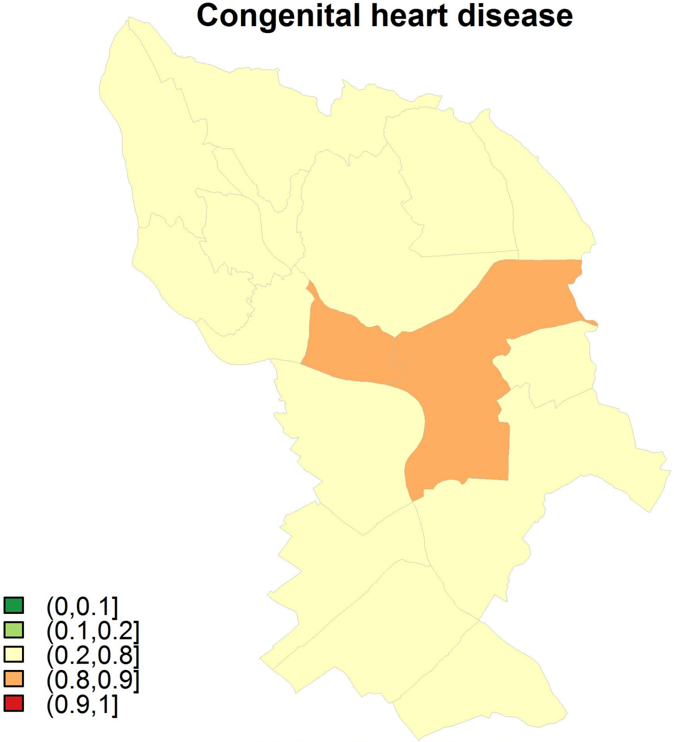

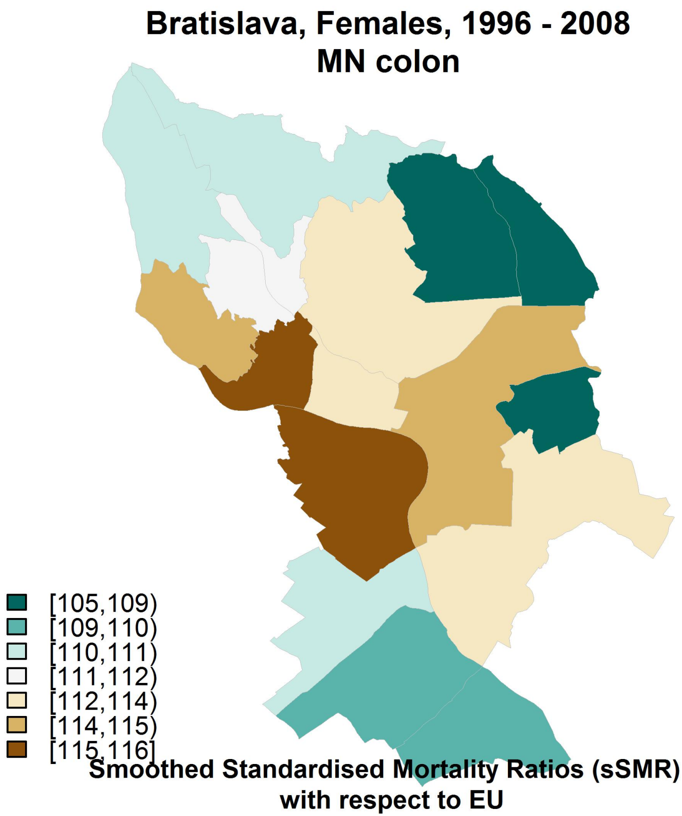
Bratislava, Females, 1996 - 2008
MN colon
[105,109)
[109,110)
[110,111)
[111,112)
[112,114)
[114,115)
[115,116]
Smoothed Standardised Mortality Ratios (sSMR)
with respect to EU

Bratislava, Females, 1996 - 2008
MN colon

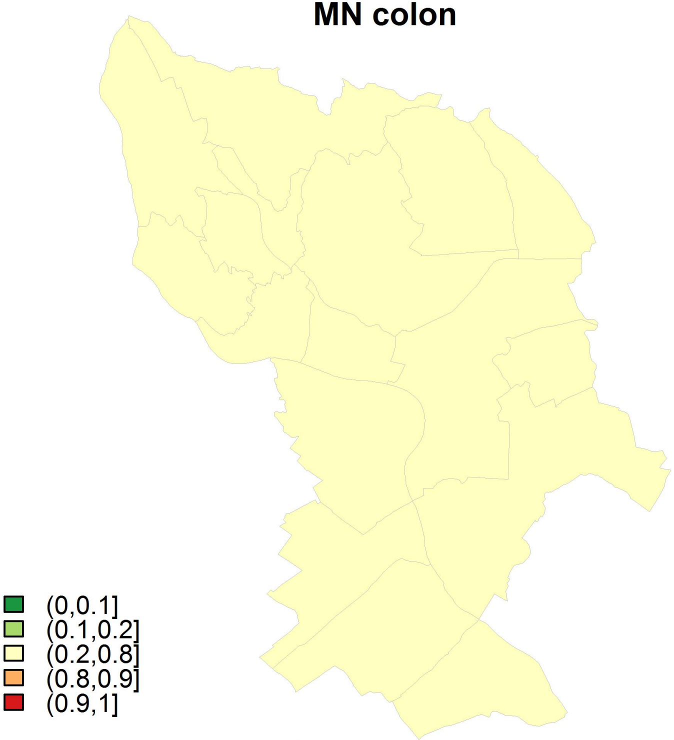

Probability sSMR > 1

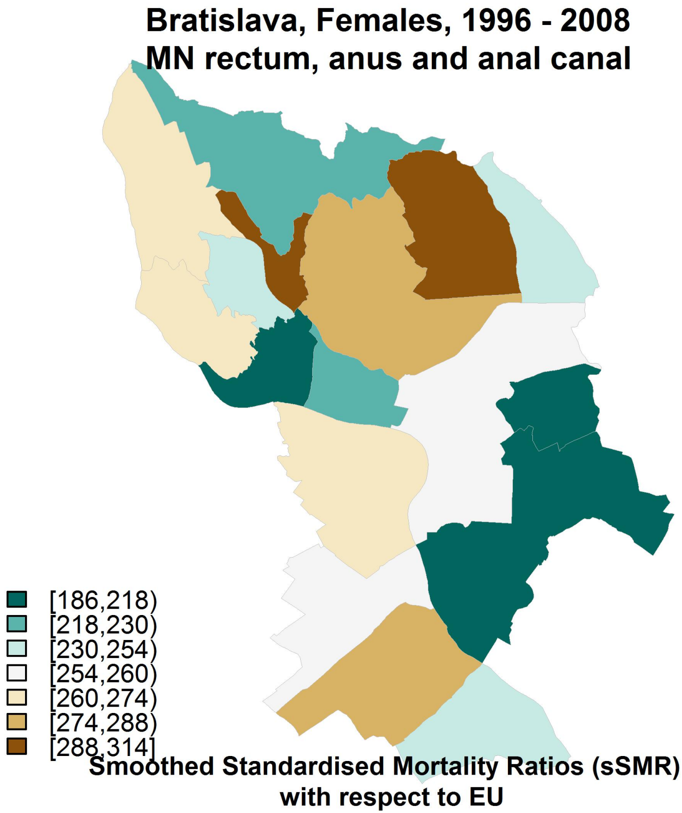
Bratislava, Females, 1996 - 2008
MN rectum, anus and anal canal
[186,218)
[218,230)
[230,254)
[254,260)
[260,274)
[274,288)
[288,314]
Smoothed Standardised Mortality Ratios (sSMR)
with respect to EU

Bratislava, Females, 1996 - 2008
MN rectum, anus and anal canal

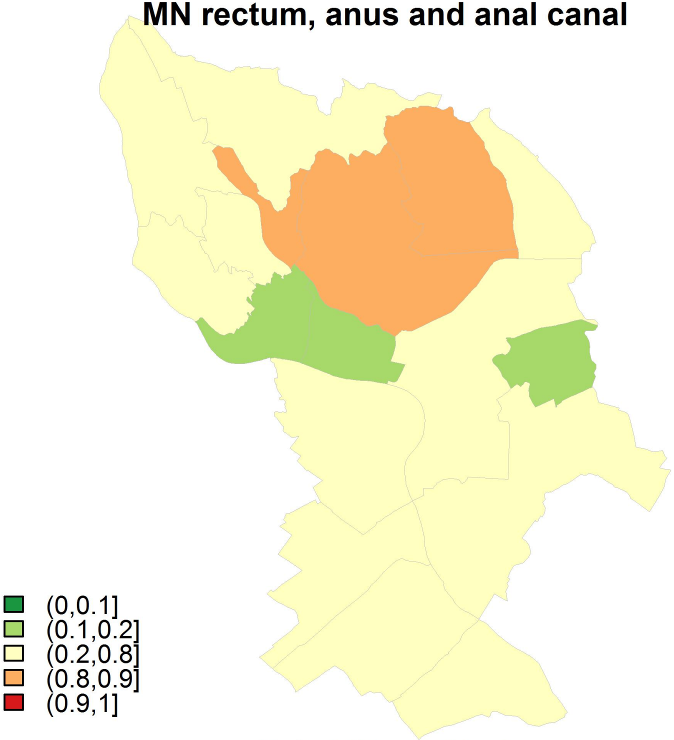

Probability sSMR > 1

Bratislava, Females, 1996 - 2008
MN cervix uteri

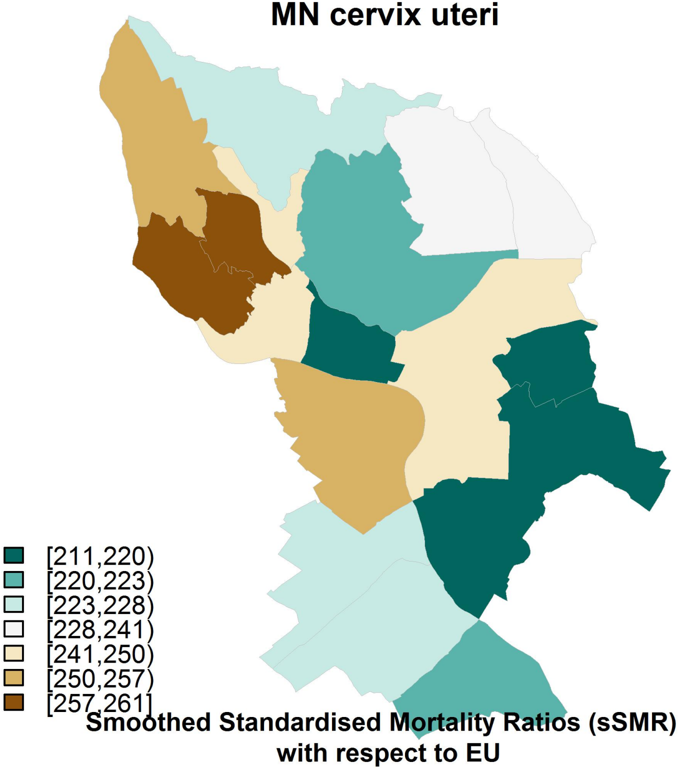

Smoothed Standardised Mortality Ratios (sSMR)
with respect to EU

Bratislava, Females, 1996 - 2008
MN cervix uteri

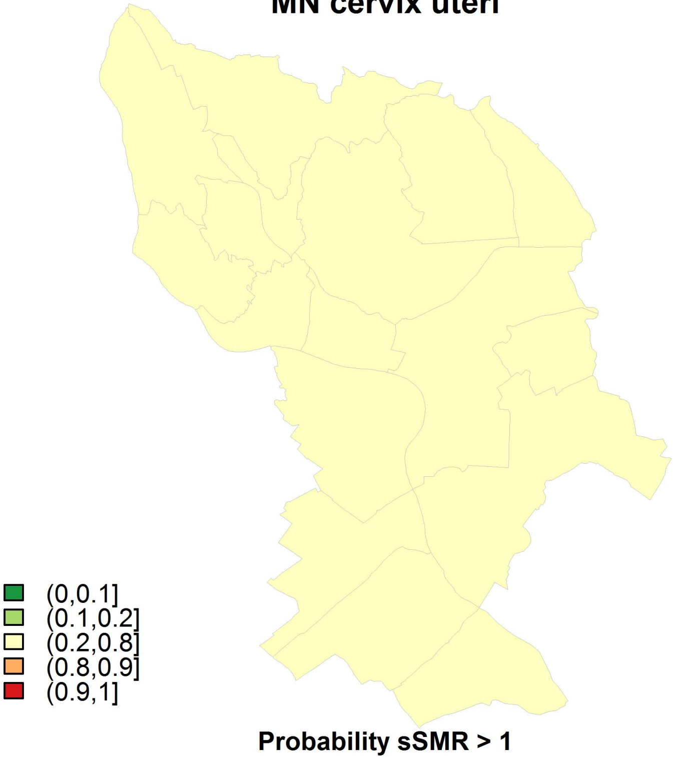

Bratislava, Females, 1996 - 2008
Hodgkin's disease

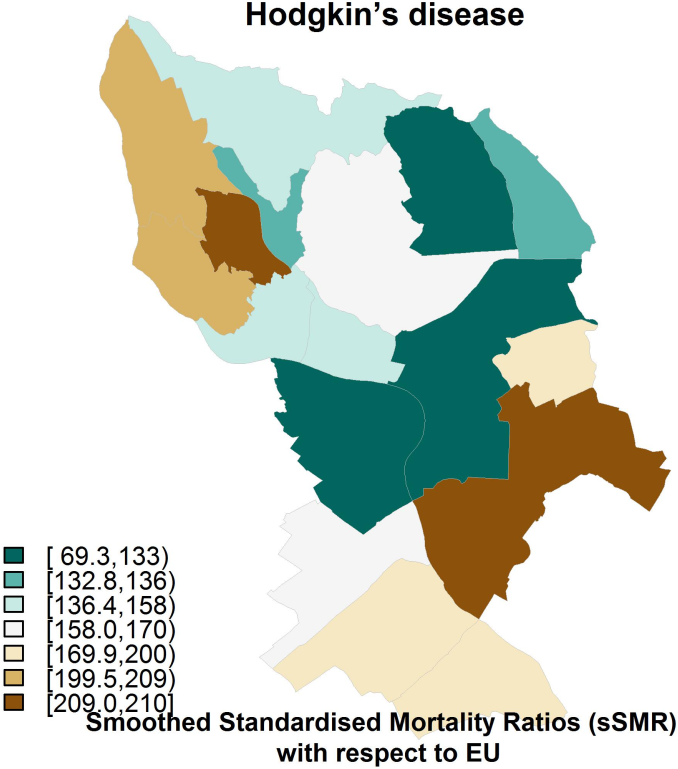

Smoothed Standardised Mortality Ratios (sSMR)
with respect to EU

Bratislava, Females, 1996 - 2008
Hodgkin's disease

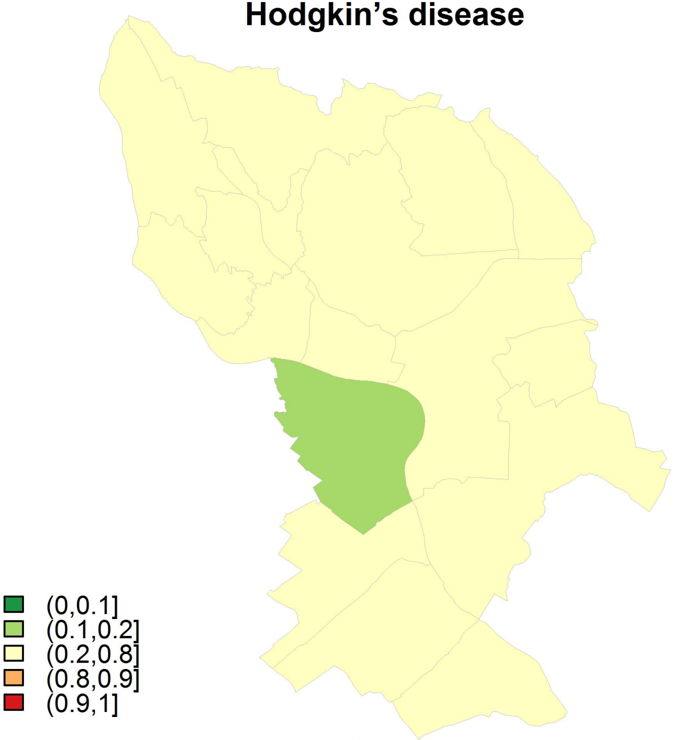

Probability sSMR > 1

Bratislava, Females, 1996 - 2008
Rheumatic heart disease

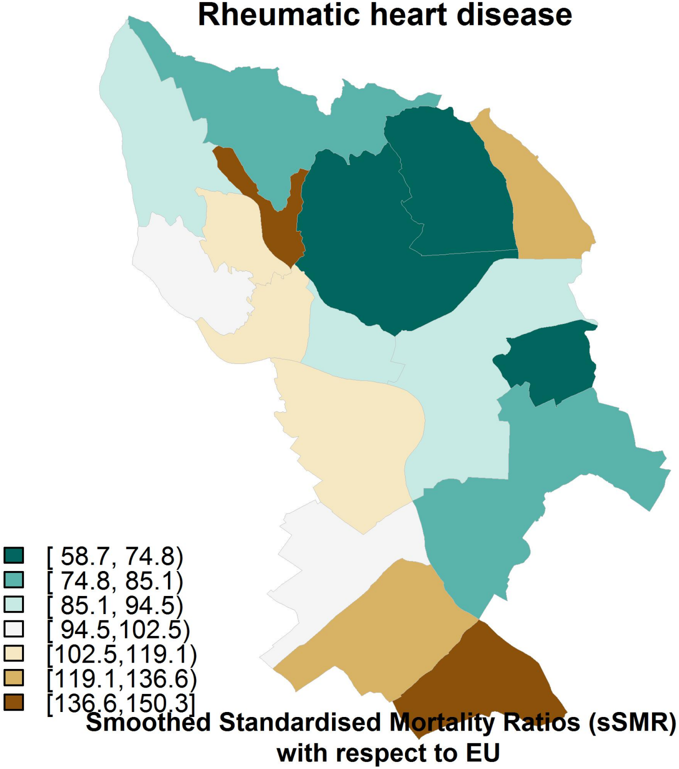

Smoothed Standardised Mortality Ratios (sSMR)
with respect to EU

Bratislava, Females, 1996 - 2008
Rheumatic heart disease

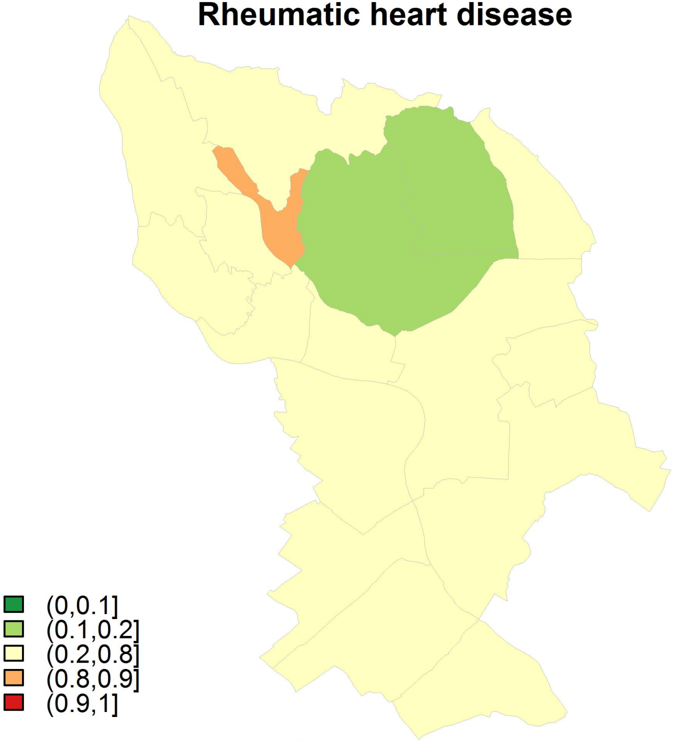

Bratislava, Females, 1996 - 2008
Hypertension

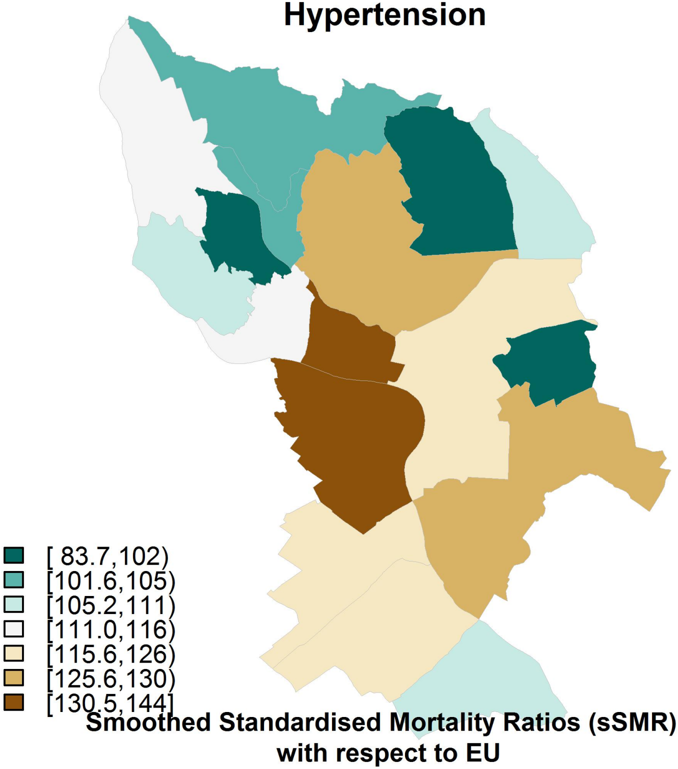

Smoothed Standardised Mortality Ratios (sSMR)
with respect to EU

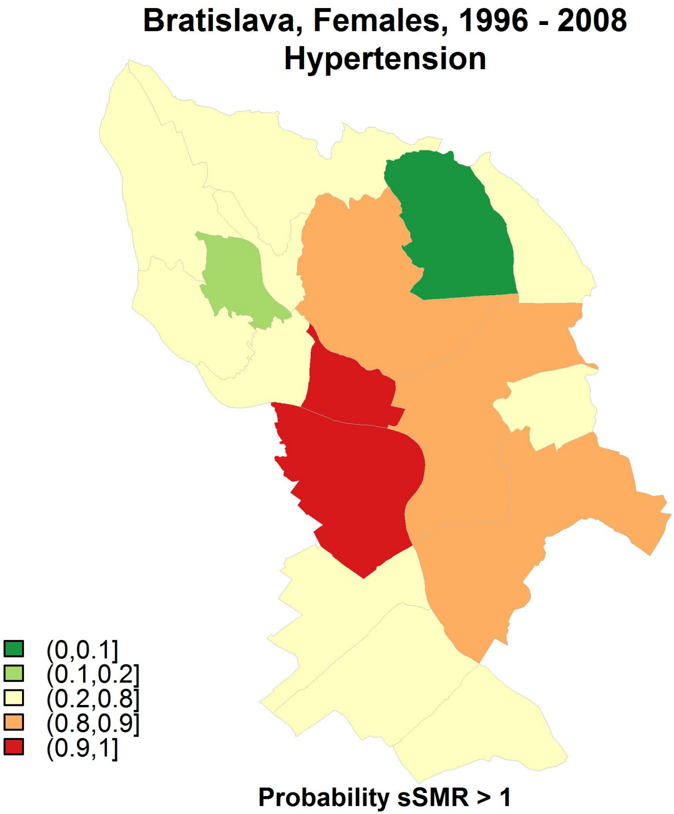
Bratislava, Females, 1996 - 2008
Hypertension
(0,0.1]
(0.1,0.2]
(0.2,0.8]
(0.8,0.9]
(0.9,1]
Probability sSMR > 1

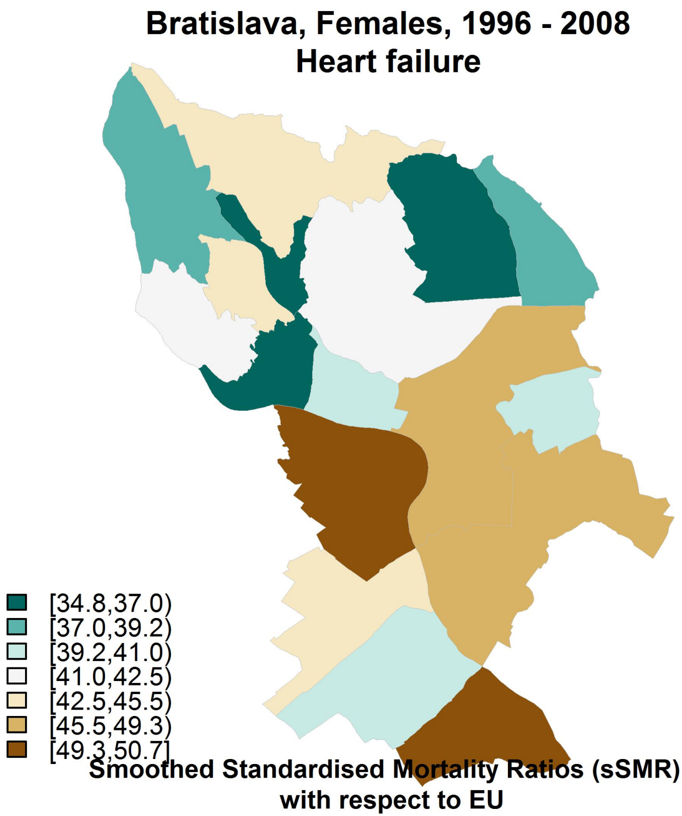
Bratislava, Females, 1996 - 2008
Heart failure
[34.8,37.0)
[37.0,39.2)
[39.2,41.0)
[41.0,42.5)
[42.5,45.5)
[45.5,49.3)
[49.3,50.7]
Smoothed Standardised Mortality Ratios (sSMR)
with respect to EU

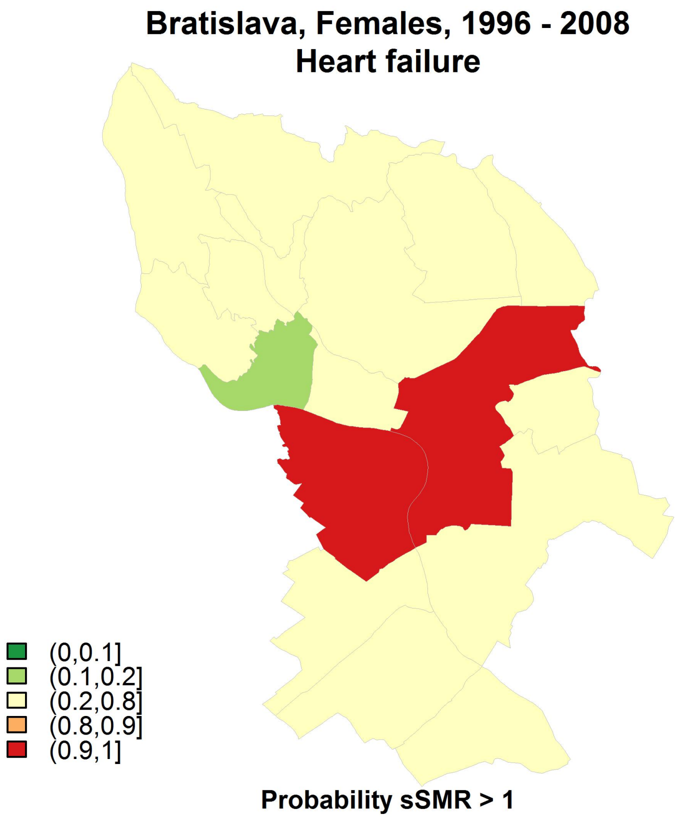
Bratislava, Females, 1996 - 2008
Heart failure
(0,0.1]
(0.1,0.2]
(0.2,0.8]
(0.8,0.9]
(0.9,1]
Probability sSMR > 1

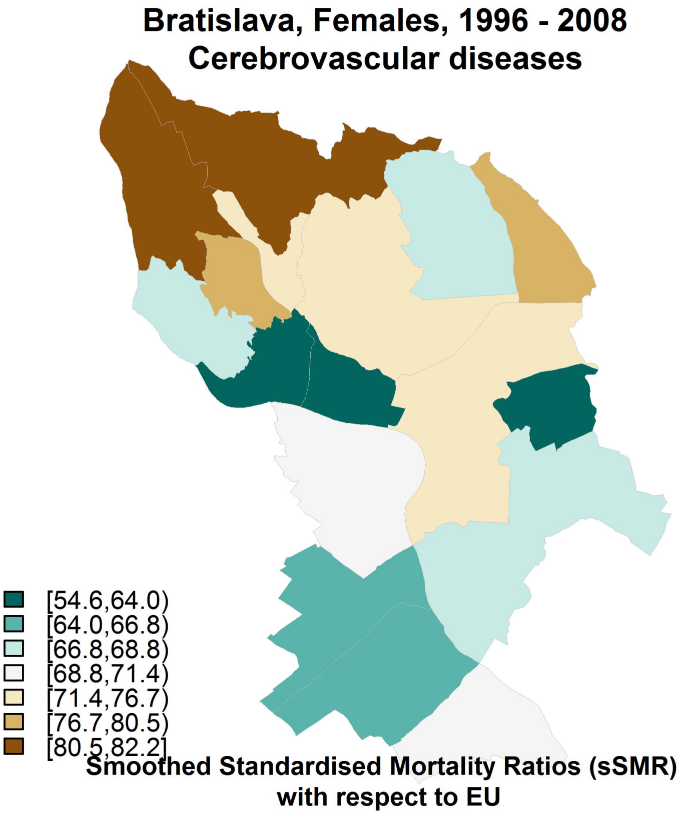
Bratislava, Females, 1996 - 2008
Cerebrovascular diseases
[54.6,64.0)
[64.0,66.8)
[66.8,68.8)
[68.8,71.4)
[71.4,76.7)
[76.7,80.5)
[80.5,82.2]
Smoothed Standardised Mortality Ratios (sSMR)
with respect to EU

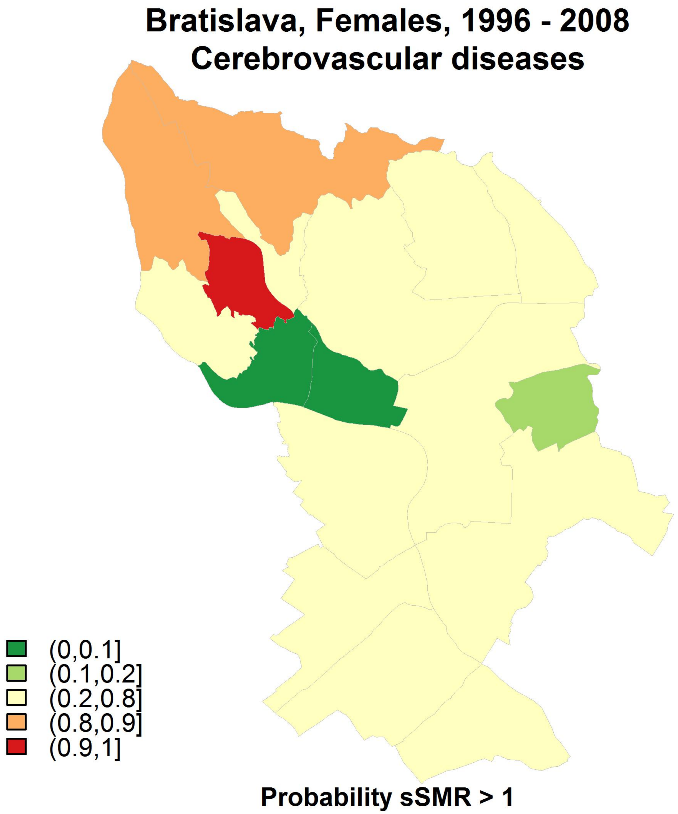
Bratislava, Females, 1996 - 2008
Cerebrovascular diseases
(0,0.1]
(0.1,0.2]
(0.2,0.8]
(0.8,0.9]
(0.9,1]
Probability sSMR > 1

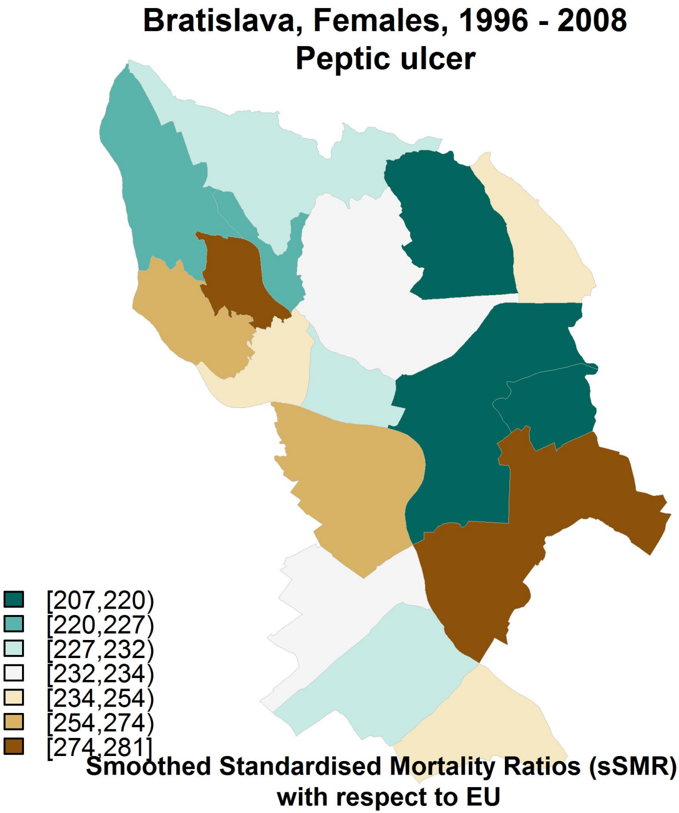
Bratislava, Females, 1996 - 2008
Peptic ulcer
[207,220)
[220,227)
[227,232)
[232,234)
[234,254)
[254,274)
[274,281]
Smoothed Standardised Mortality Ratios (sSMR)
with respect to EU

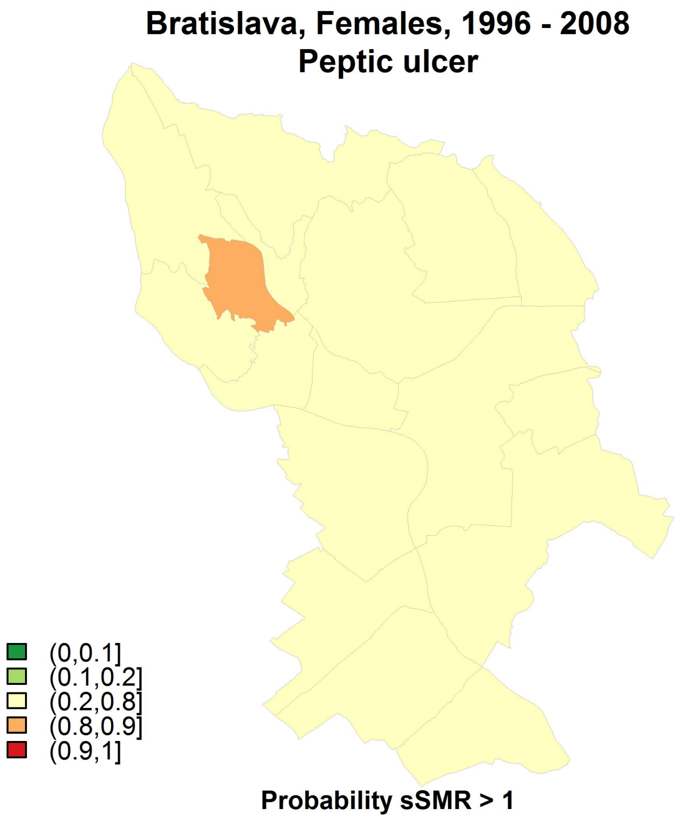
Bratislava, Females, 1996 - 2008
Peptic ulcer
(0,0.1]
(0.1,0.2]
(0.2,0.8]
(0.8,0.9]
(0.9,1]
Probability sSMR > 1

Bratislava, Females, 1996 - 2008
Renal failure

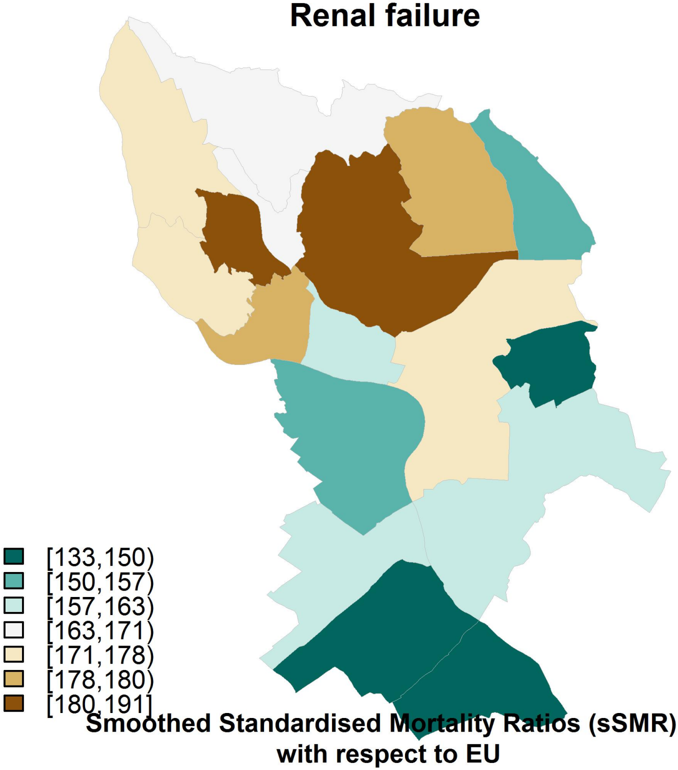

Smoothed Standardised Mortality Ratios (sSMR)
with respect to EU

Bratislava, Females, 1996 - 2008
Renal failure

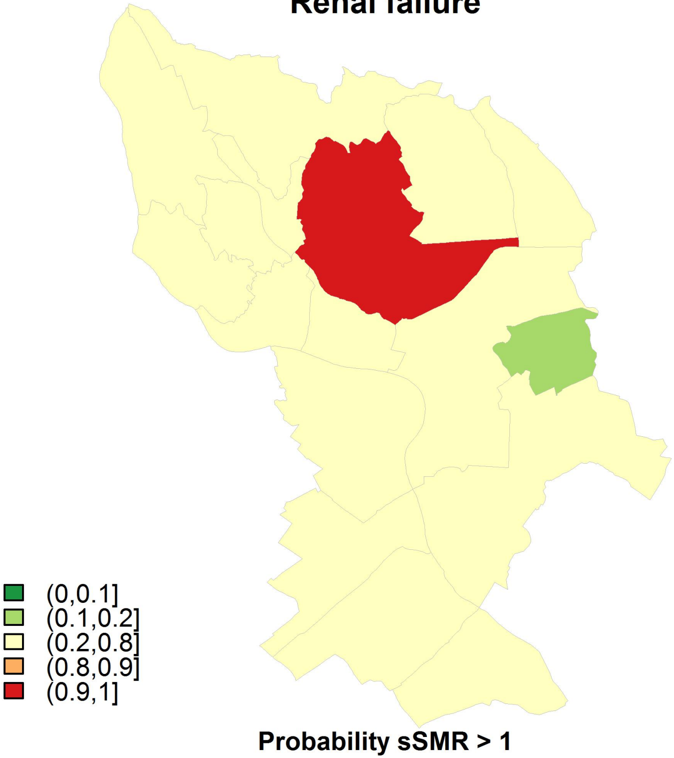

Bratislava, Females, 1996 - 2008
Conditions originating in the perinatal period

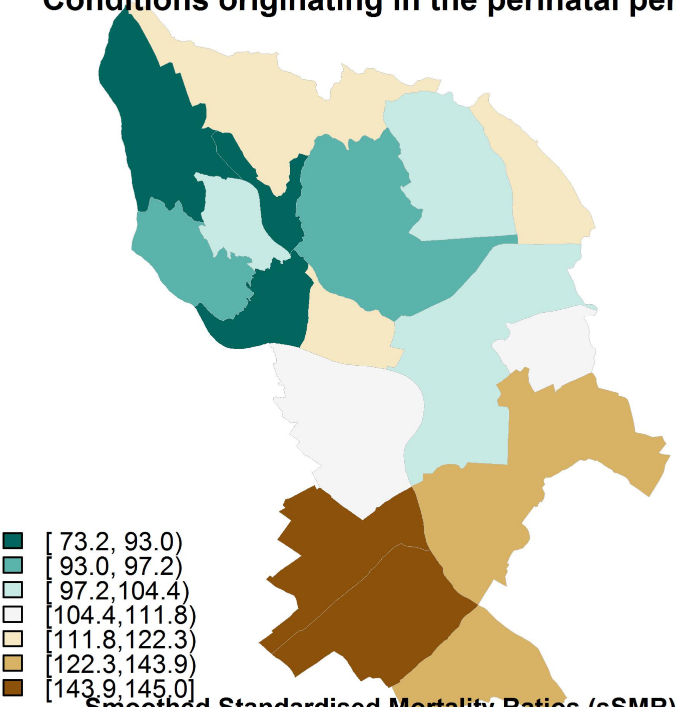

Smoothed Standardised Mortality Ratios (sSMR)
with respect to EU

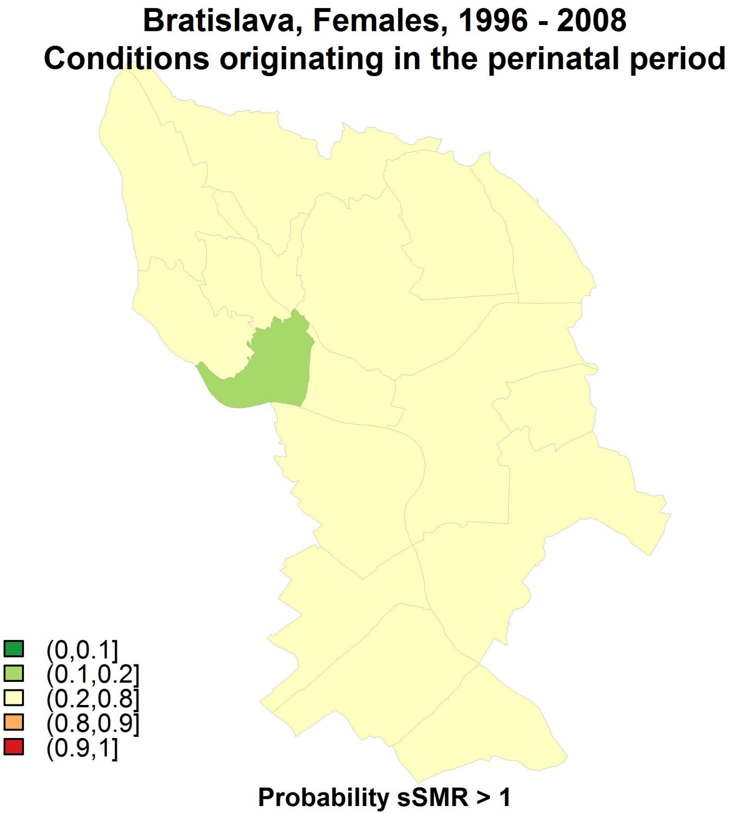
Bratislava, Females, 1996 - 2008
Conditions originating in the perinatal period
(0,0.1]
(0.1,0.2]
(0.2,0.8]
(0.8,0.9]
(0.9,1]
Probability sSMR > 1

Bratislava, Females, 1996 - 2008
Congenital heart disease

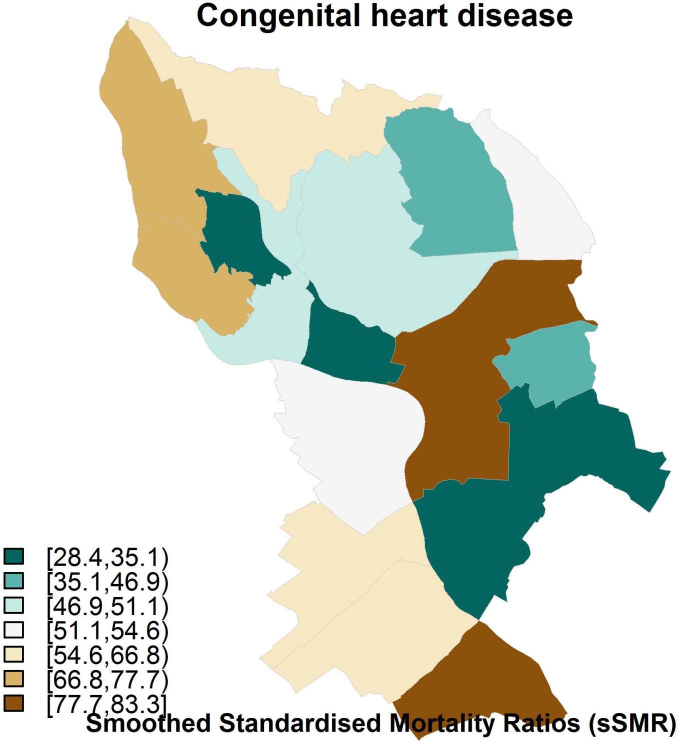

Smoothed Standardised Mortality Ratios (sSMR) with respect to EU

Bratislava, Females, 1996 - 2008
Congenital heart disease

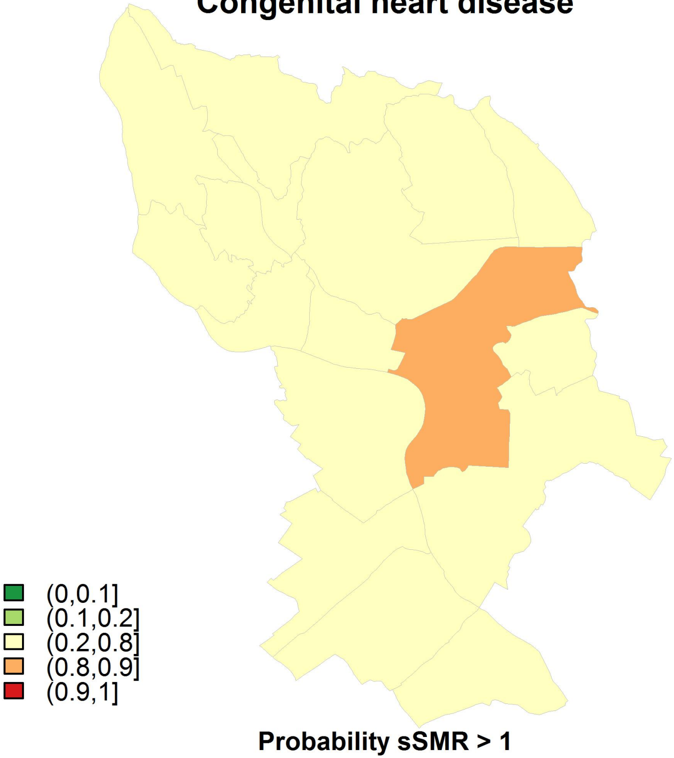

Supplement: Additional file 5 — Cause-specific mortality maps for Bratislava. [file 1476-072X-13-8-S5.pdf]
