# Supplementary material for: Social differences in avoidable mortality between small areas of 15 European cities: an ecological study
Source: Int J Health Geogr. 2014 Mar 12;13:8. doi: 10.1186/1476-072X-13-8 (PMC4007807; doi:10.1186/1476-072X-13-8)
Supplement: Additional file 6 — Cause-specific mortality maps for Brussels. [file 1476-072X-13-8-S6.pdf]

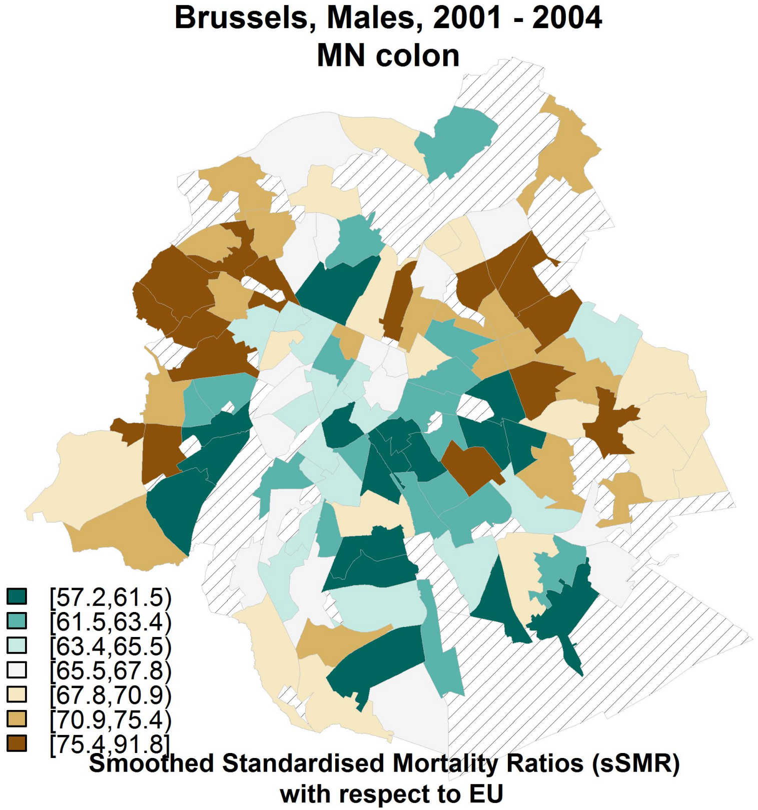
Brussels, Males, 2001 - 2004
MN colon
[57.2,61.5)
[61.5,63.4)
[63.4,65.5)
[65.5,67.8)
[67.8,70.9)
[70.9,75.4)
[75.4,91.8]
Smoothed Standardised Mortality Ratios (sSMR)
with respect to EU

Brussels, Males, 2001 - 2004
MN colon

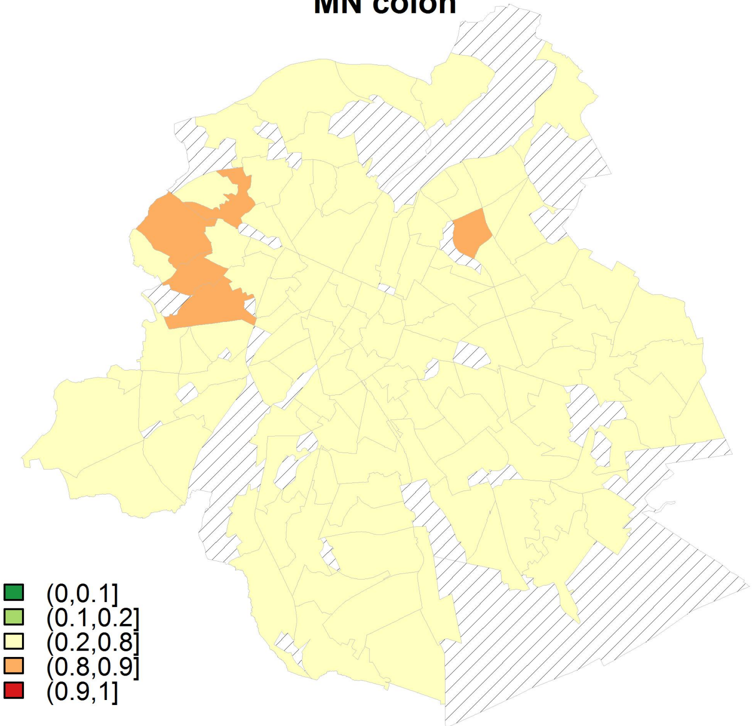

Probability sSMR > 1

Brussels, Males, 2001 - 2004
MN rectum, anus and anal canal

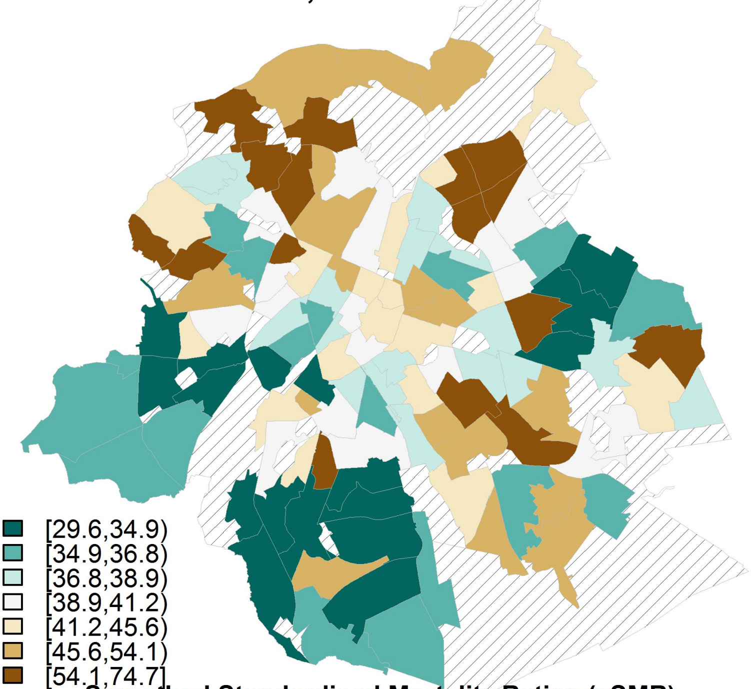

Smoothed Standardised Mortality Ratios (sSMR)
with respect to EU

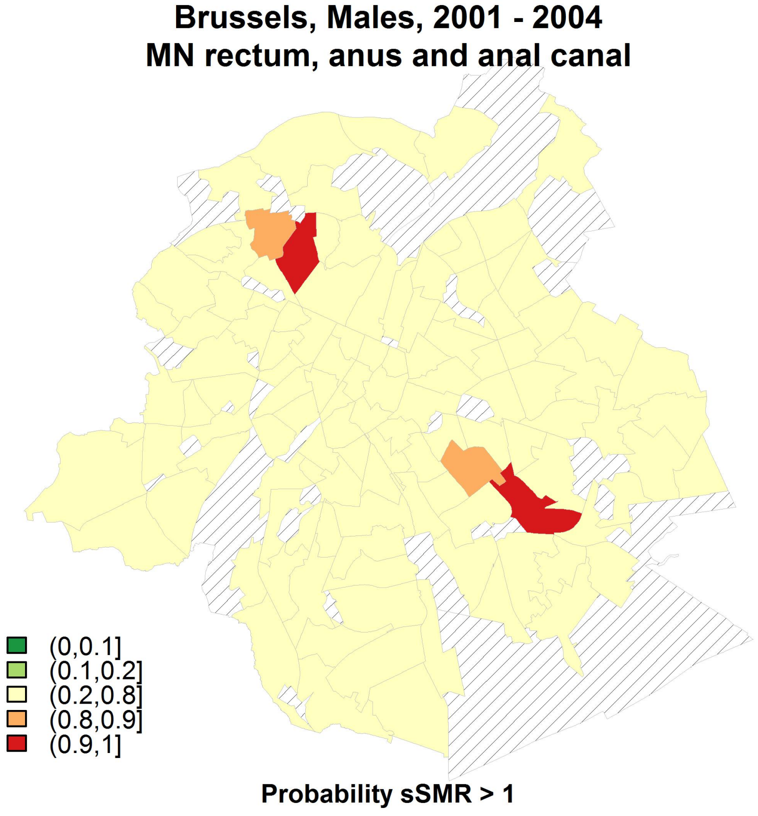
Brussels, Males, 2001 - 2004
MN rectum, anus and anal canal
(0,0.1]
(0.1,0.2]
(0.2,0.8]
(0.8,0.9]
(0.9,1]
Probability sSMR > 1

Brussels, Males, 2001 - 2004
Hypertension

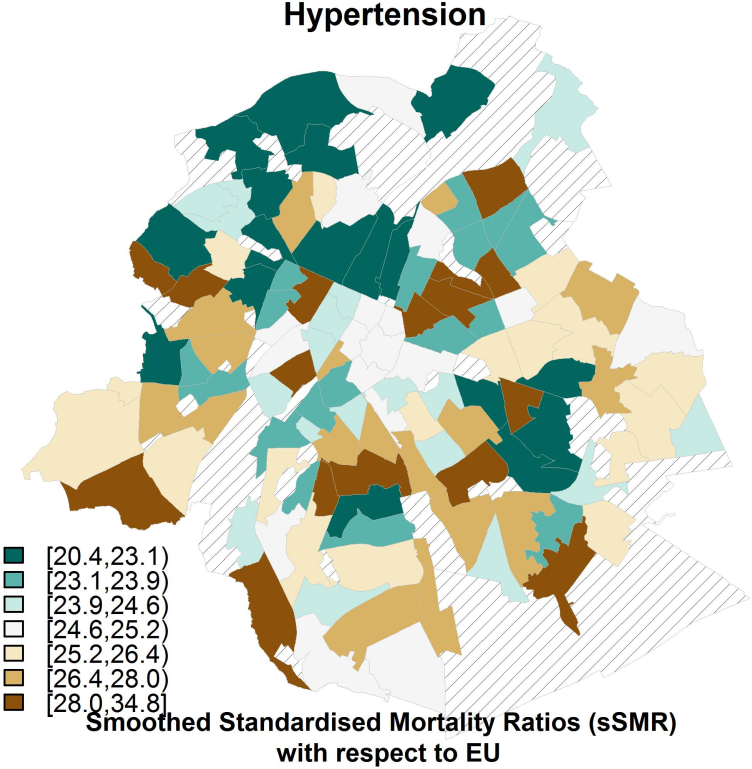

Smoothed Standardised Mortality Ratios (sSMR)
with respect to EU

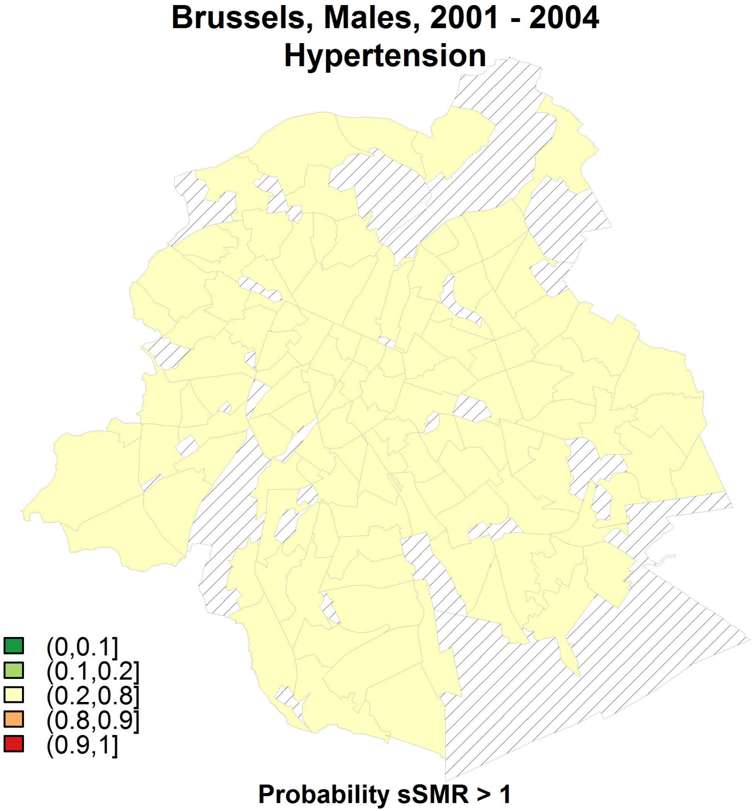

Brussels, Males, 2001 - 2004
Hypertension
(0,0.1]
(0.1,0.2]
(0.2,0.8]
(0.8,0.9]
(0.9,1]
Probability sSMR > 1

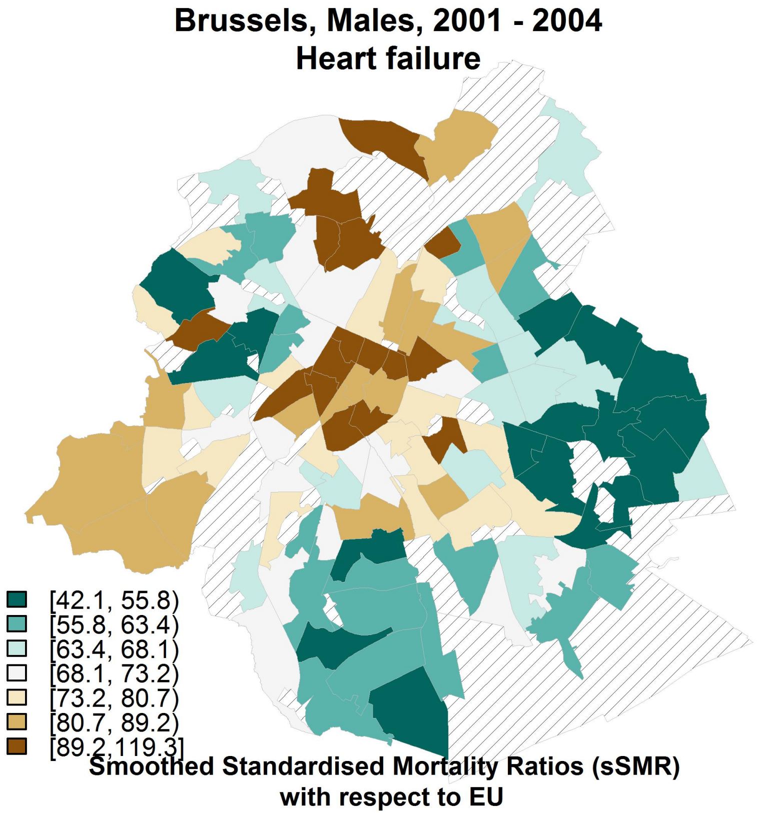
Brussels, Males, 2001 - 2004
Heart failure
[42.1, 55.8)
[55.8, 63.4)
[63.4, 68.1)
[68.1, 73.2)
[73.2, 80.7)
[80.7, 89.2)
[89.2,119.3]
Smoothed Standardised Mortality Ratios (sSMR)
with respect to EU

Brussels, Males, 2001 - 2004
Heart failure

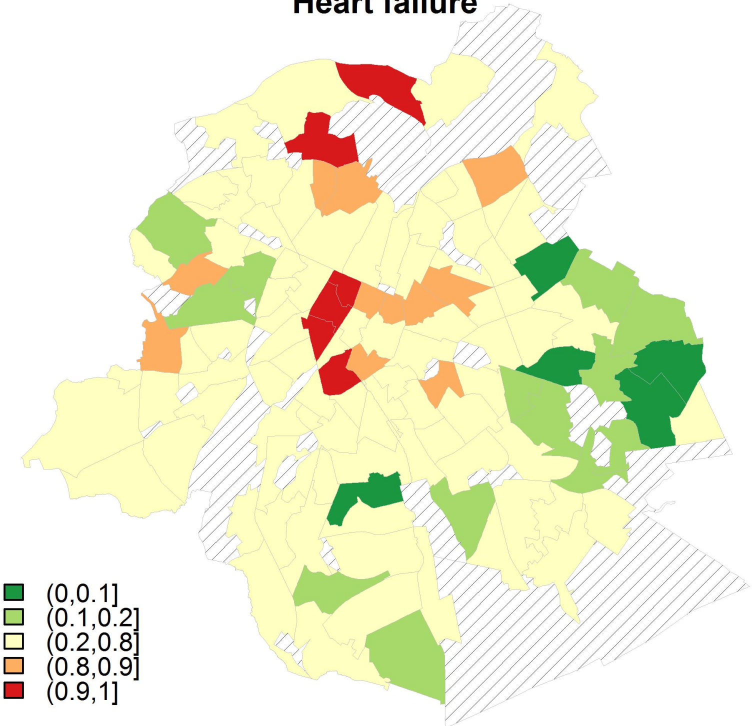

Probability sSMR > 1

Brussels, Males, 2001 - 2004
Cerebrovascular diseases

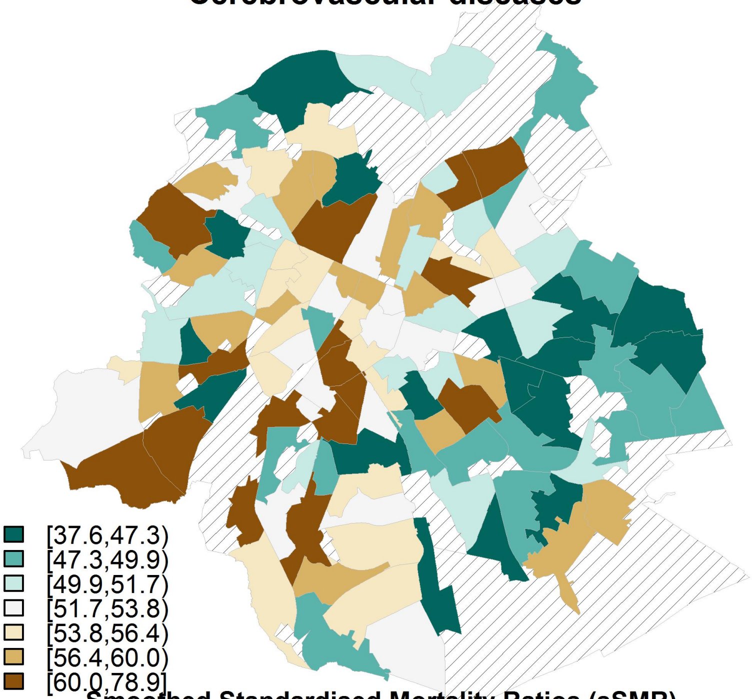

Smoothed Standardised Mortality Ratios (sSMR)
with respect to EU

Brussels, Males, 2001 - 2004
Cerebrovascular diseases

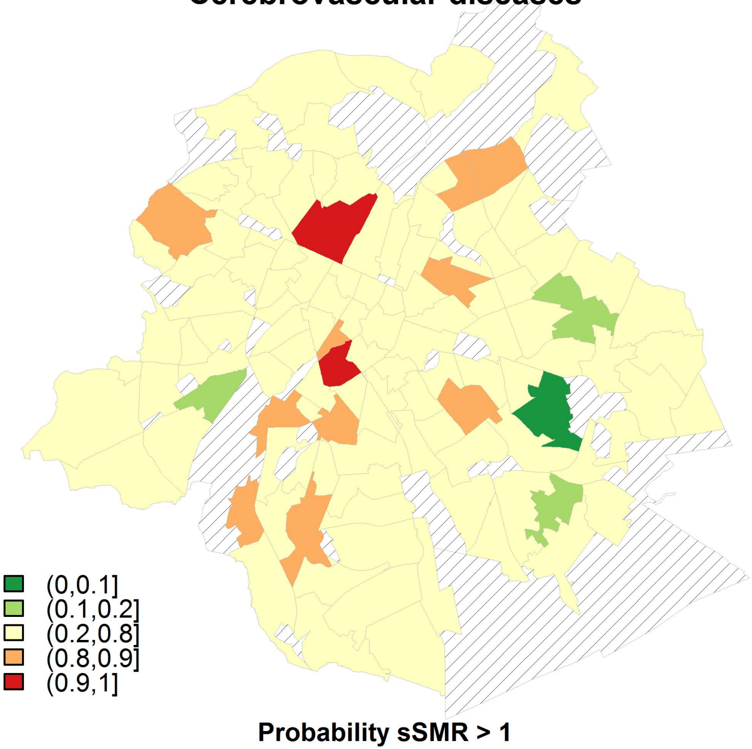

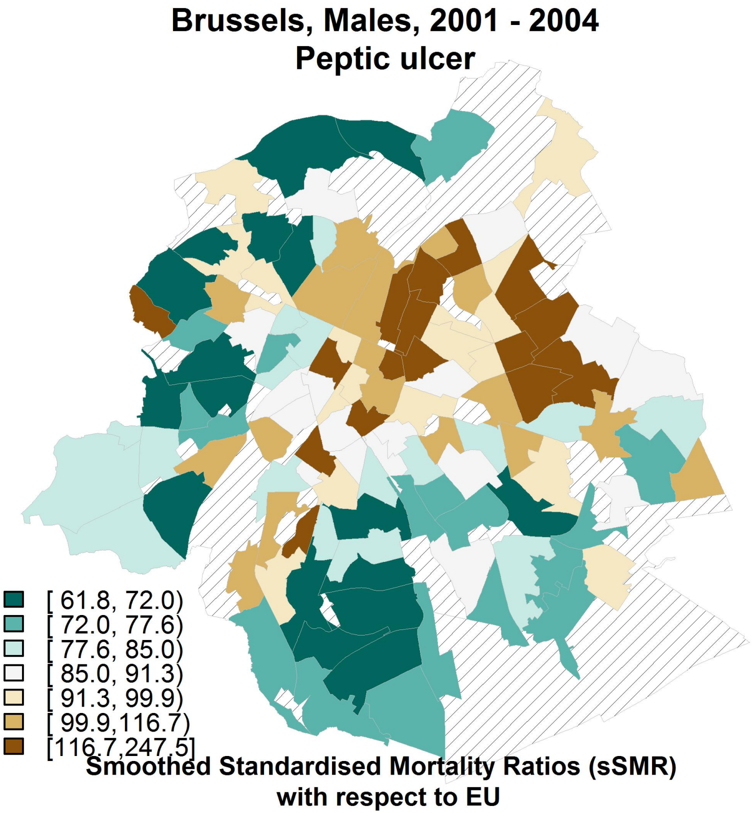
Brussels, Males, 2001 - 2004
Peptic ulcer
[61.8, 72.0)
[72.0, 77.6)
[77.6, 85.0)
[85.0, 91.3)
[91.3, 99.9)
[99.9,116.7)
[116.7,247.5]
Smoothed Standardised Mortality Ratios (sSMR)
with respect to EU

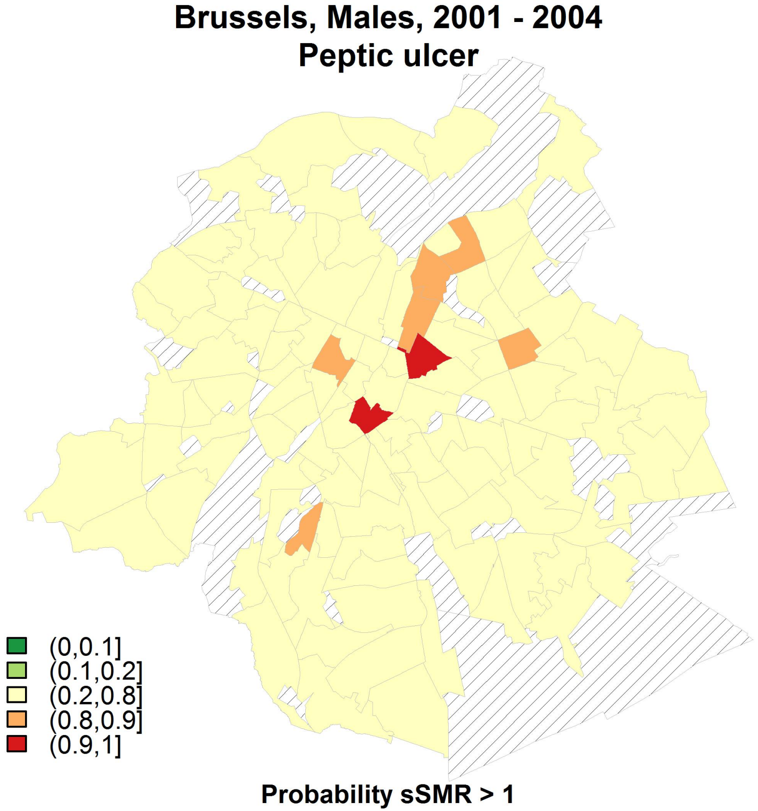
Brussels, Males, 2001 - 2004
Peptic ulcer
(0,0.1]
(0.1,0.2]
(0.2,0.8]
(0.8,0.9]
(0.9,1]
Probability sSMR > 1

Brussels, Males, 2001 - 2004
Renal failure

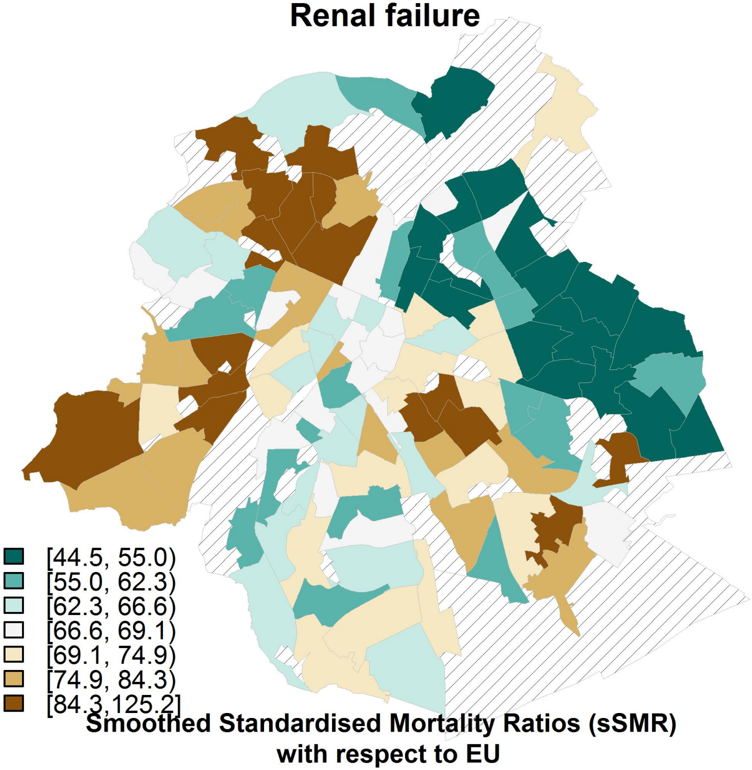

Smoothed Standardised Mortality Ratios (sSMR)
with respect to EU

Brussels, Males, 2001 - 2004
Renal failure

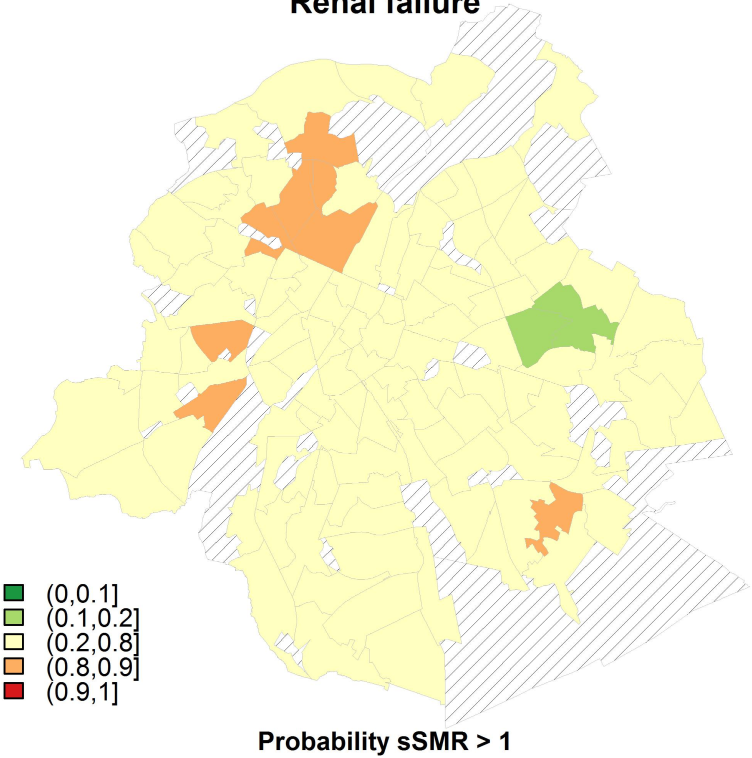

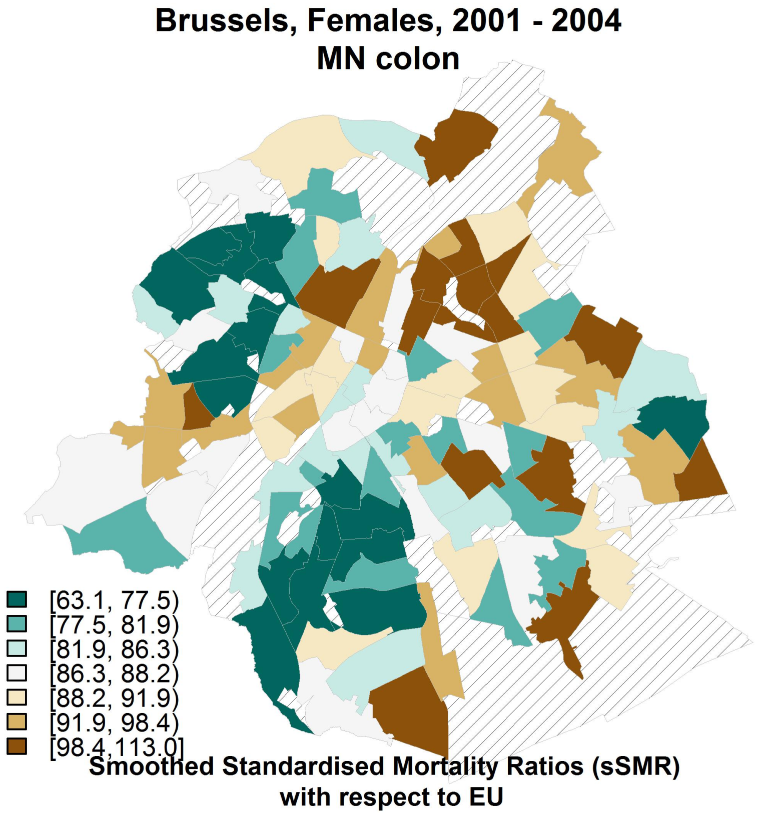
Brussels, Females, 2001 - 2004
MN colon
[63.1, 77.5)
[77.5, 81.9)
[81.9, 86.3)
[86.3, 88.2)
[88.2, 91.9)
[91.9, 98.4)
[98.4,113.0]
Smoothed Standardised Mortality Ratios (sSMR)
with respect to EU

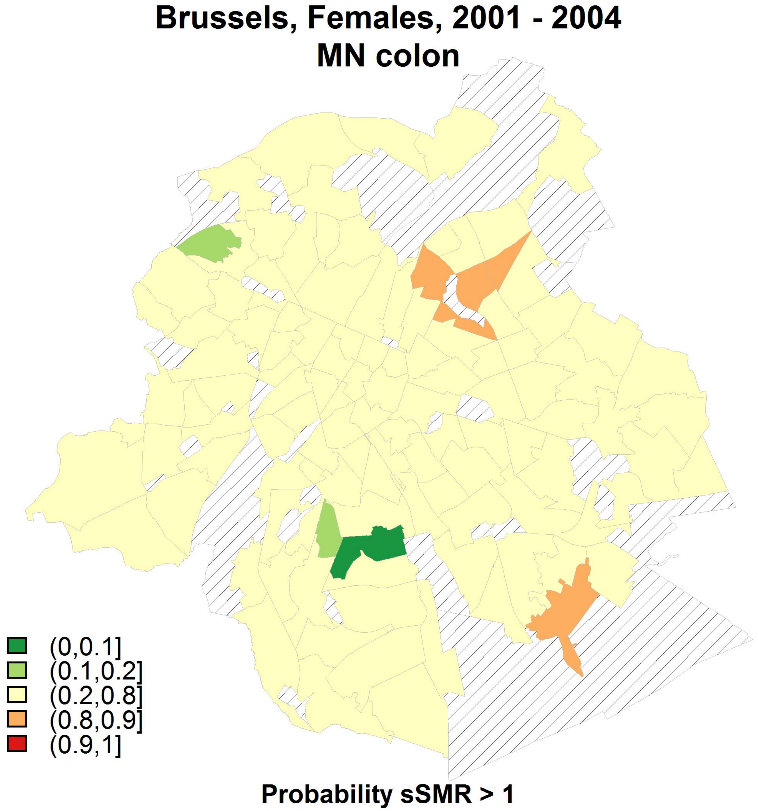

Brussels, Females, 2001 - 2004
MN colon
(0,0.1]
(0.1,0.2]
(0.2,0.8]
(0.8,0.9]
(0.9,1]
Probability sSMR > 1

Brussels, Females, 2001 - 2004
MN rectum, anus and anal canal

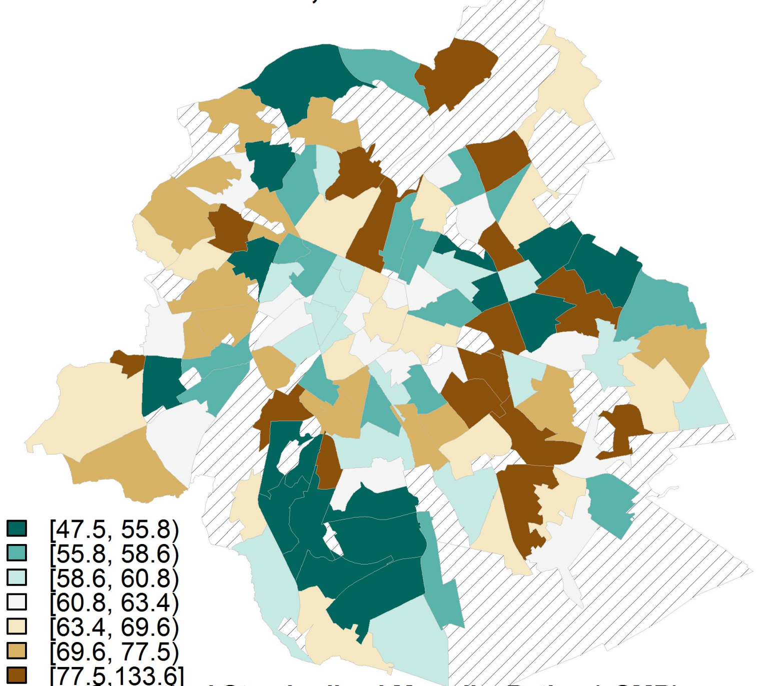

Smoothed Standardised Mortality Ratios (sSMR)
with respect to EU

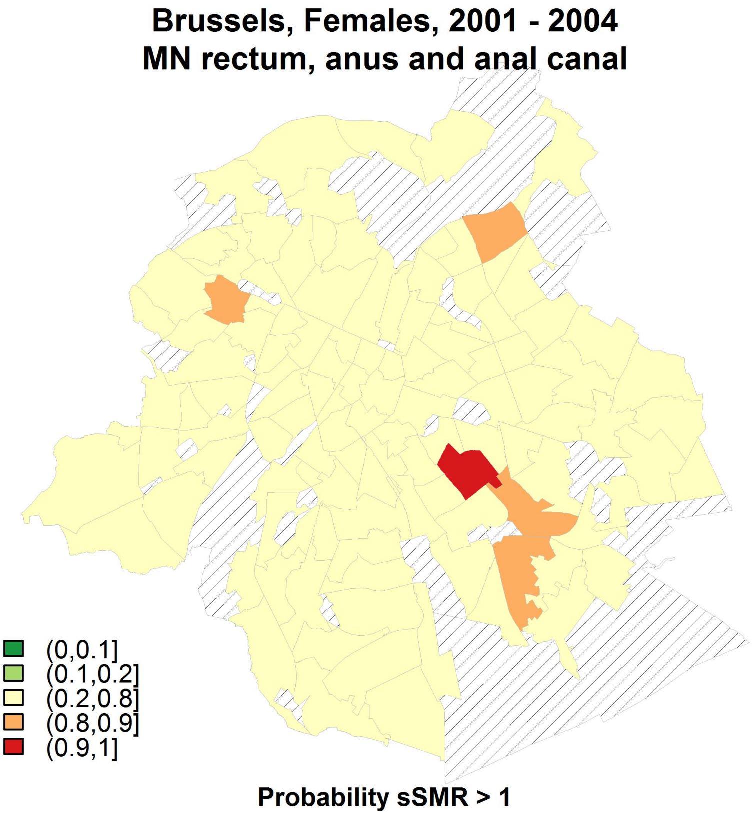
Brussels, Females, 2001 - 2004
MN rectum, anus and anal canal
(0,0.1]
(0.1,0.2]
(0.2,0.8]
(0.8,0.9]
(0.9,1]
Probability sSMR > 1

Brussels, Females, 2001 - 2004
MN cervix uteri

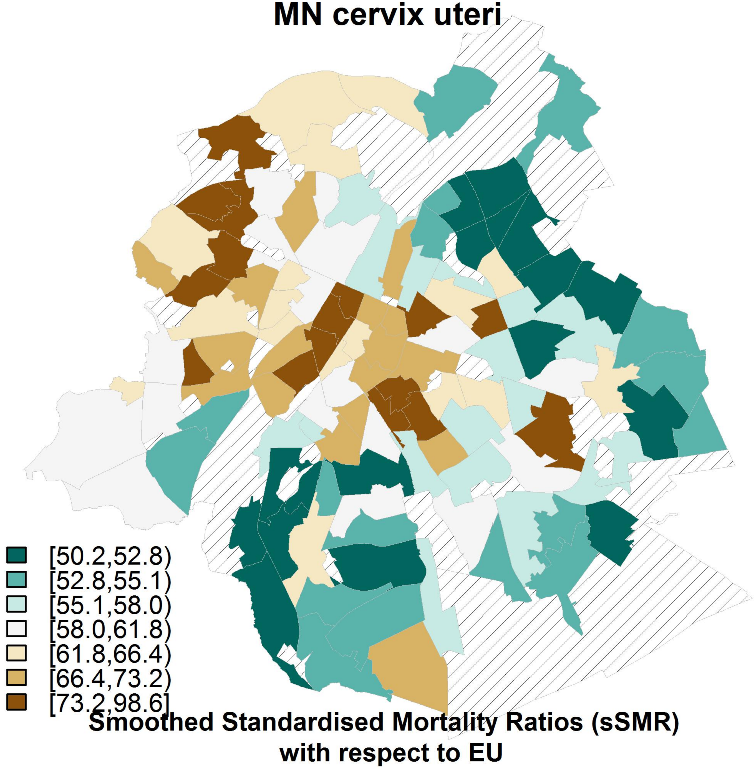

Smoothed Standardised Mortality Ratios (sSMR) with respect to EU

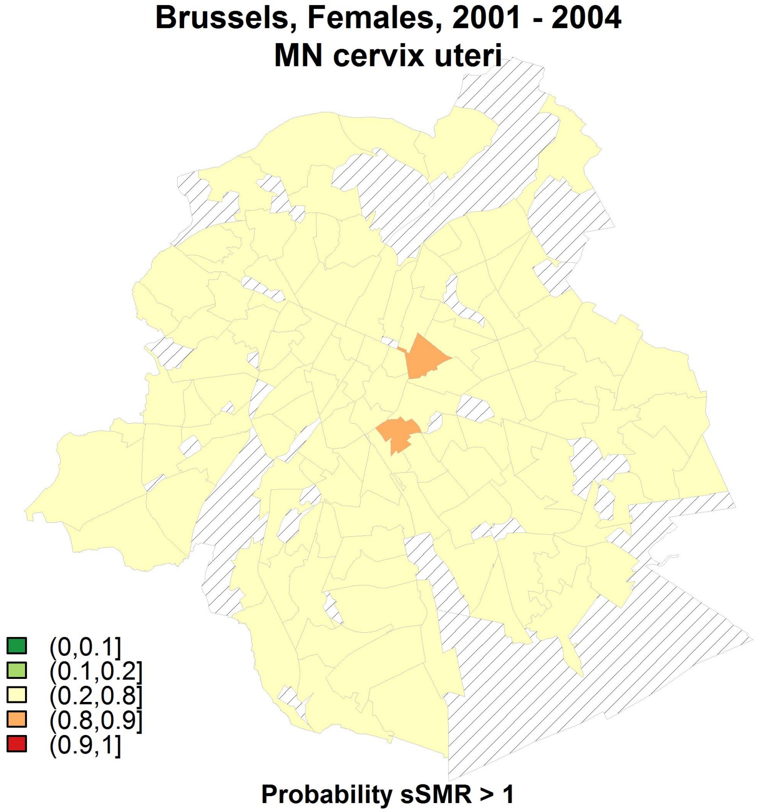
Brussels, Females, 2001 - 2004
MN cervix uteri
(0,0.1]
(0.1,0.2]
(0.2,0.8]
(0.8,0.9]
(0.9,1]
Probability sSMR > 1

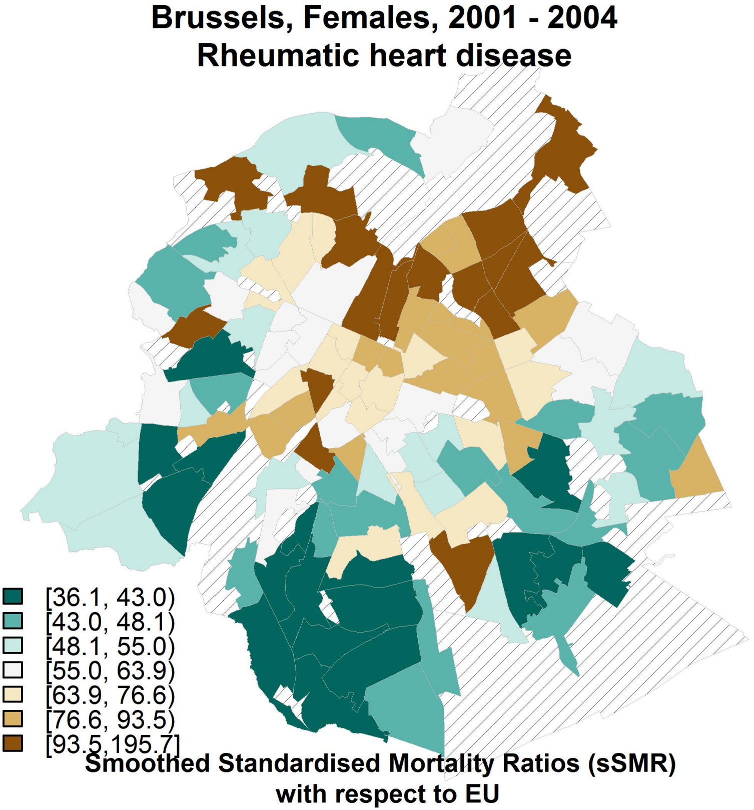
Brussels, Females, 2001 - 2004
Rheumatic heart disease
[36.1, 43.0)
[43.0, 48.1)
[48.1, 55.0)
[55.0, 63.9)
[63.9, 76.6)
[76.6, 93.5)
[93.5,195.7]
Smoothed Standardised Mortality Ratios (sSMR)
with respect to EU

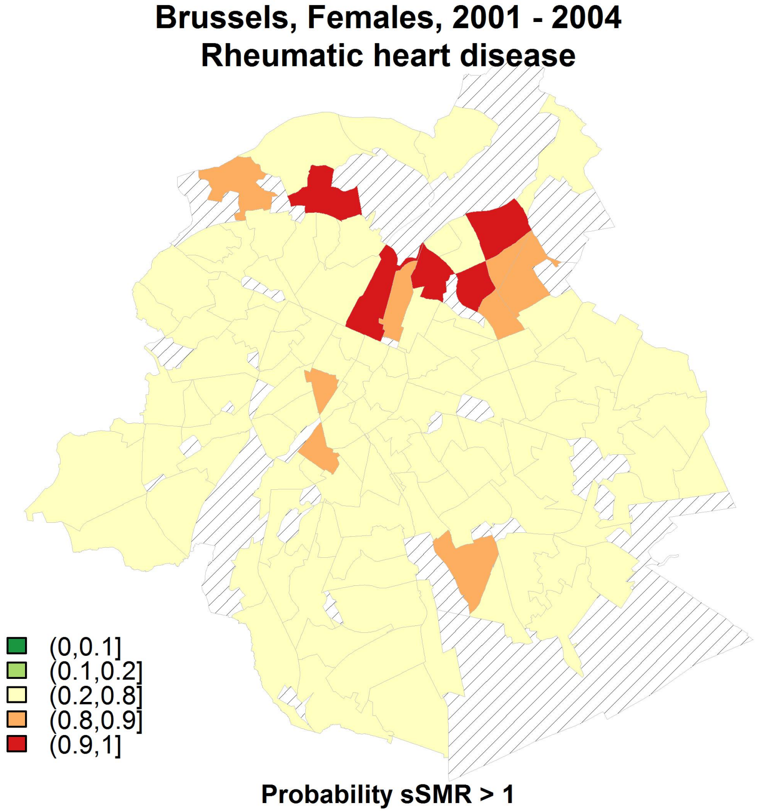
Brussels, Females, 2001 - 2004
Rheumatic heart disease
(0,0.1]
(0.1,0.2]
(0.2,0.8]
(0.8,0.9]
(0.9,1]
Probability sSMR > 1

Brussels, Females, 2001 - 2004
Hypertension

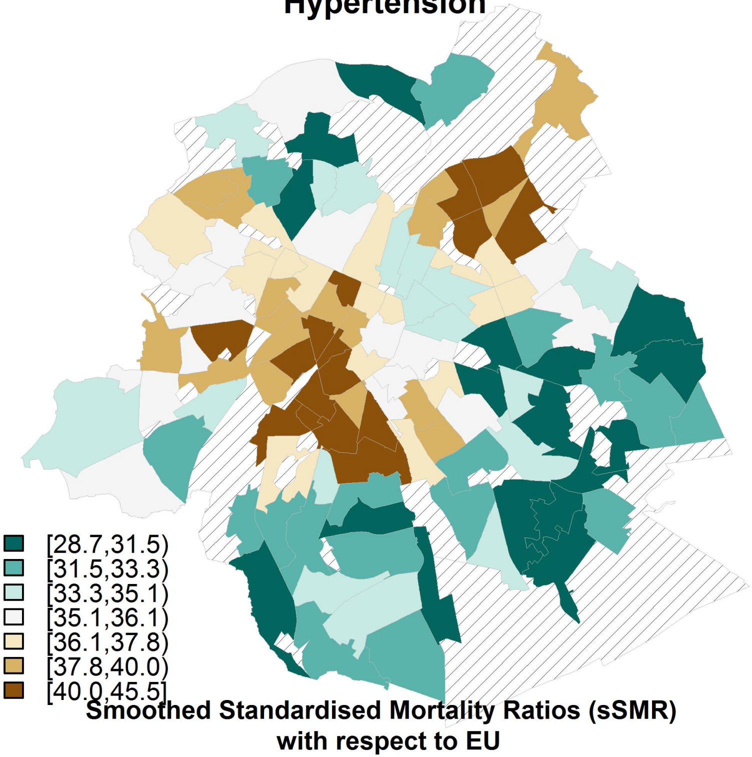

Smoothed Standardised Mortality Ratios (sSMR) with respect to EU

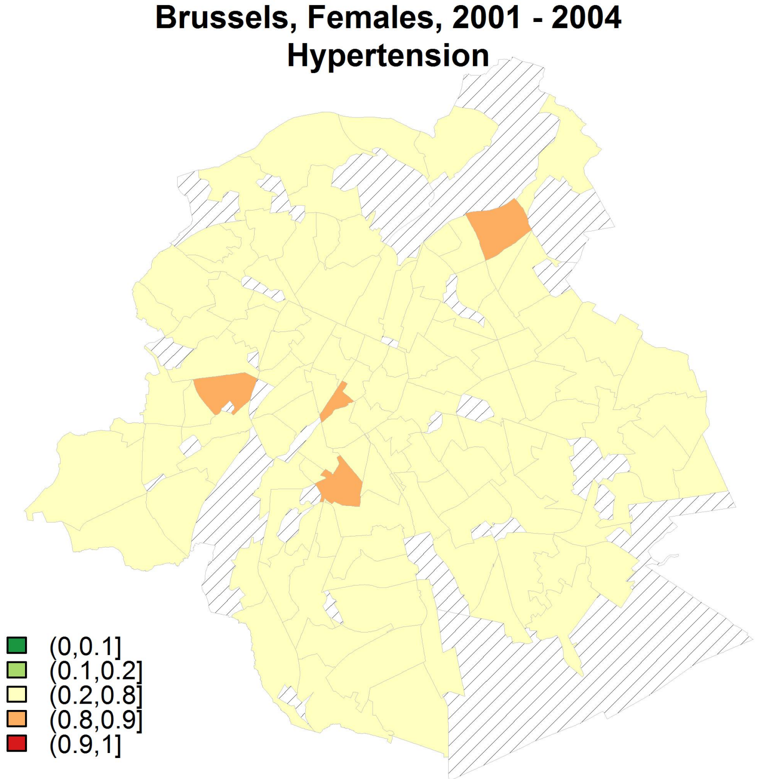

Probability sSMR > 1

Brussels, Females, 2001 - 2004
Heart failure

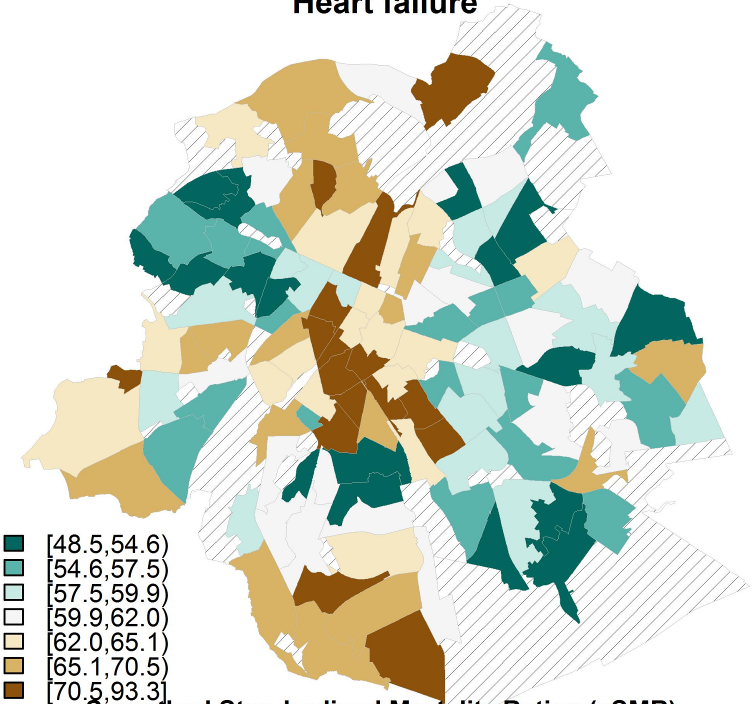

Smoothed Standardised Mortality Ratios (sSMR) with respect to EU

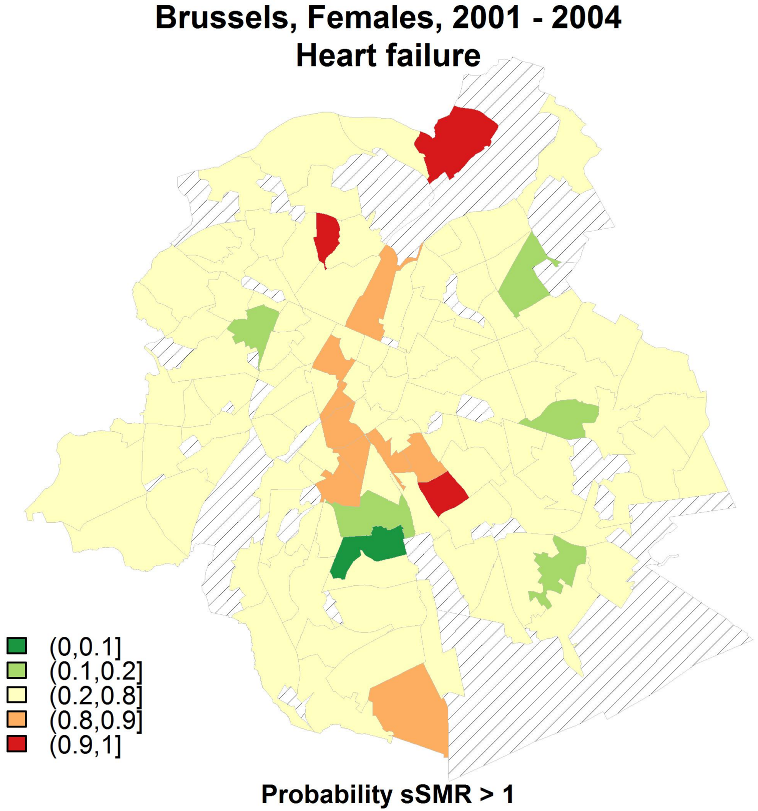
Brussels, Females, 2001 - 2004
Heart failure
(0,0.1]
(0.1,0.2]
(0.2,0.8]
(0.8,0.9]
(0.9,1]
Probability sSMR > 1

Brussels, Females, 2001 - 2004
Cerebrovascular diseases

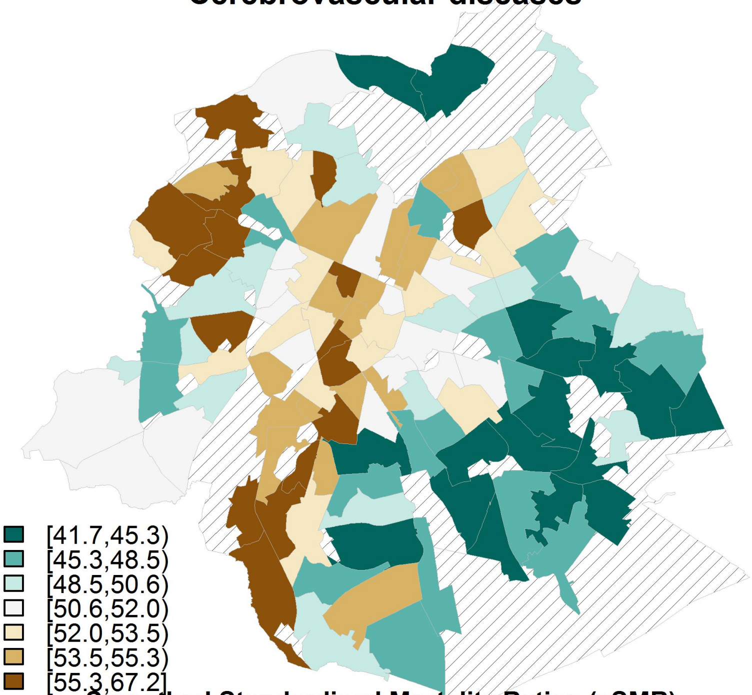

Smoothed Standardised Mortality Ratios (sSMR)
with respect to EU

Brussels, Females, 2001 - 2004
Cerebrovascular diseases

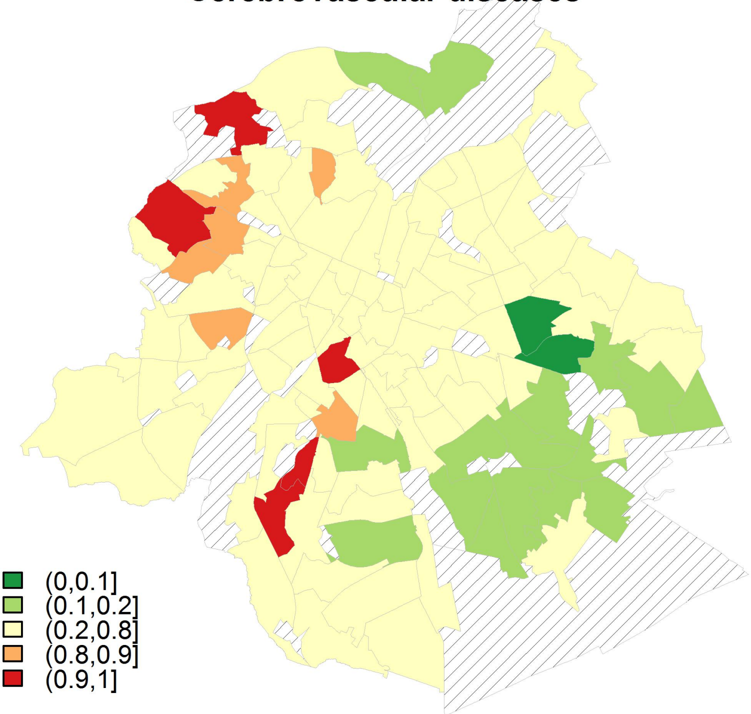

Probability sSMR > 1

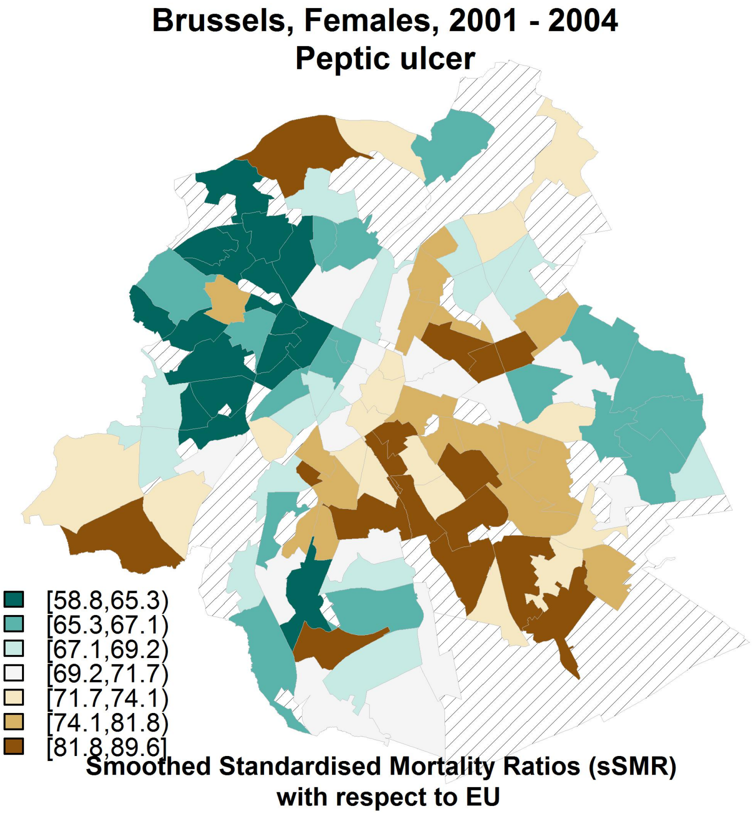
Brussels, Females, 2001 - 2004
Peptic ulcer
[58.8,65.3)
[65.3,67.1)
[67.1,69.2)
[69.2,71.7)
[71.7,74.1)
[74.1,81.8)
[81.8,89.6]
Smoothed Standardised Mortality Ratios (sSMR)
with respect to EU

Brussels, Females, 2001 - 2004
Peptic ulcer

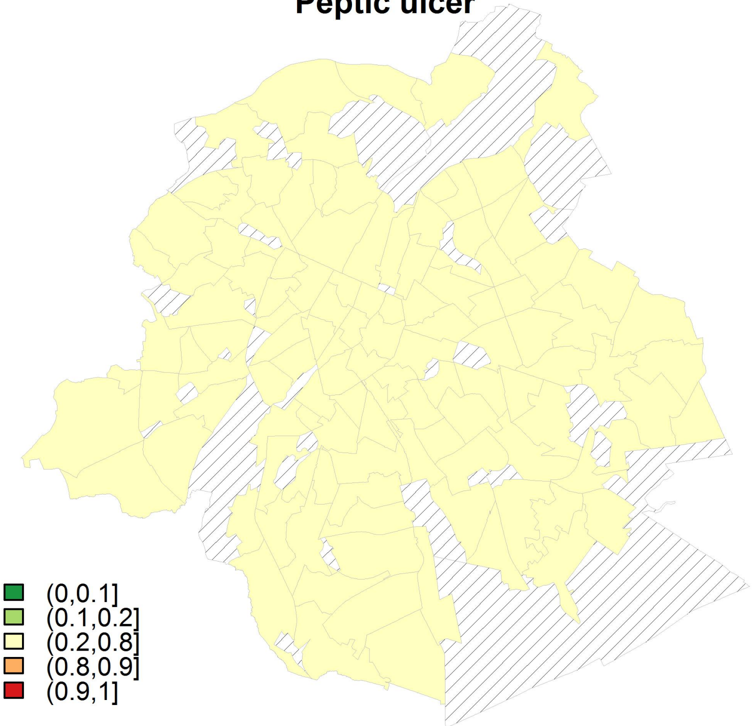

Brussels, Females, 2001 - 2004
Renal failure

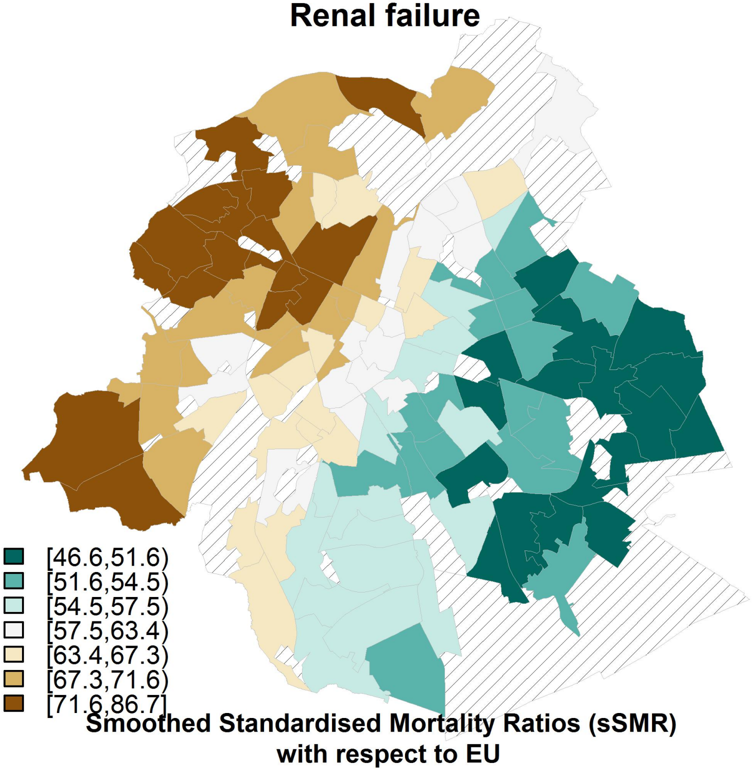

Smoothed Standardised Mortality Ratios (sSMR)
with respect to EU

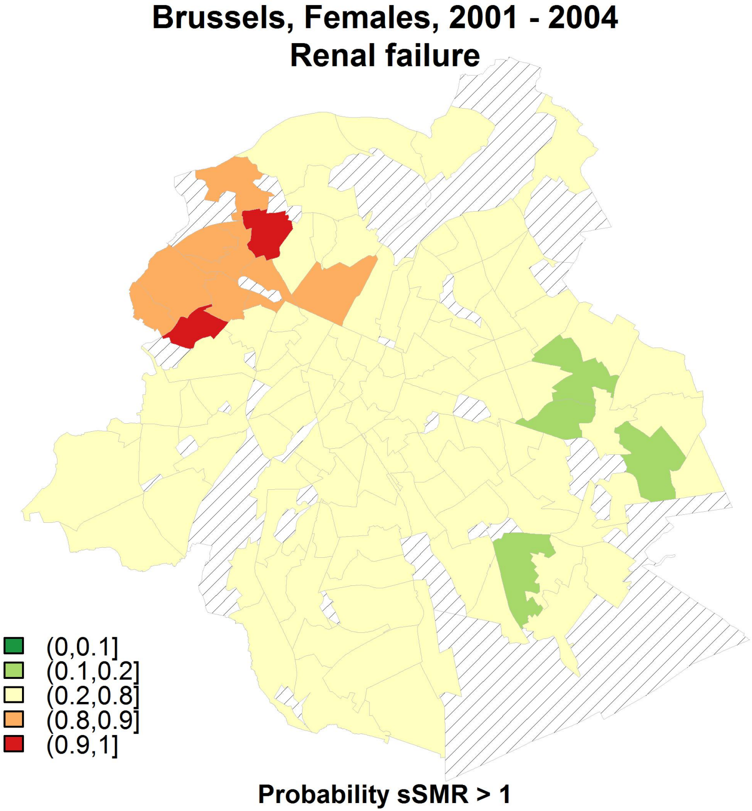
Brussels, Females, 2001 - 2004
Renal failure
(0,0.1]
(0.1,0.2]
(0.2,0.8]
(0.8,0.9]
(0.9,1]
Probability sSMR > 1
