# Supplementary material for: Social differences in avoidable mortality between small areas of 15 European cities: an ecological study
Source: Int J Health Geogr. 2014 Mar 12;13:8. doi: 10.1186/1476-072X-13-8 (PMC4007807; doi:10.1186/1476-072X-13-8)
Supplement: Additional file 7 — Cause-specific mortality maps for Budapest. [file 1476-072X-13-8-S7.pdf]

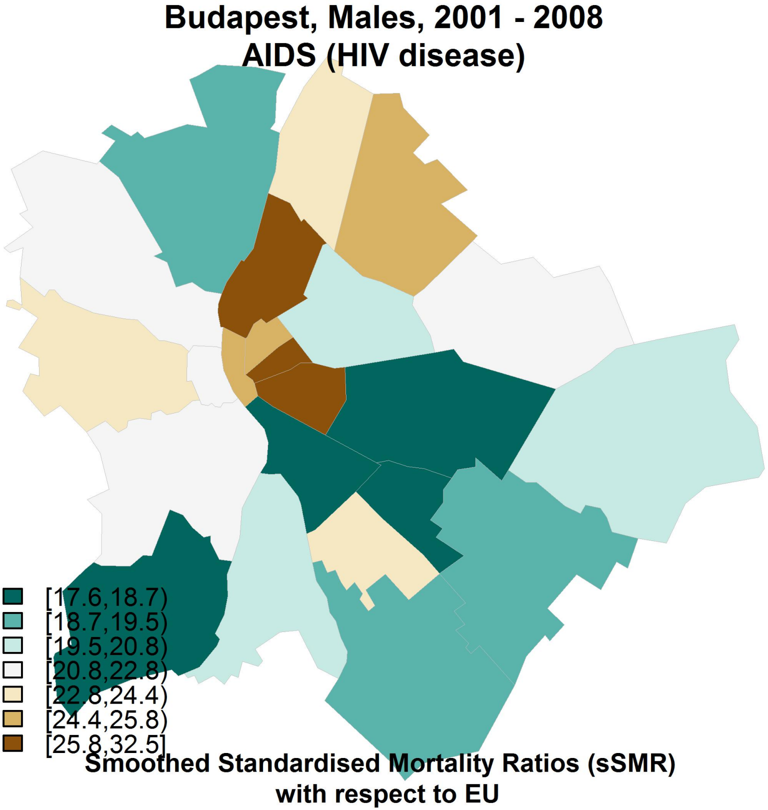
Budapest, Males, 2001 - 2008
AIDS (HIV disease)
[17.6,18.7)
[18.7,19.5)
[19.5,20.8)
[20.8,22.8)
[22.8,24.4)
[24.4,25.8)
[25.8,32.5]
Smoothed Standardised Mortality Ratios (sSMR)
with respect to EU

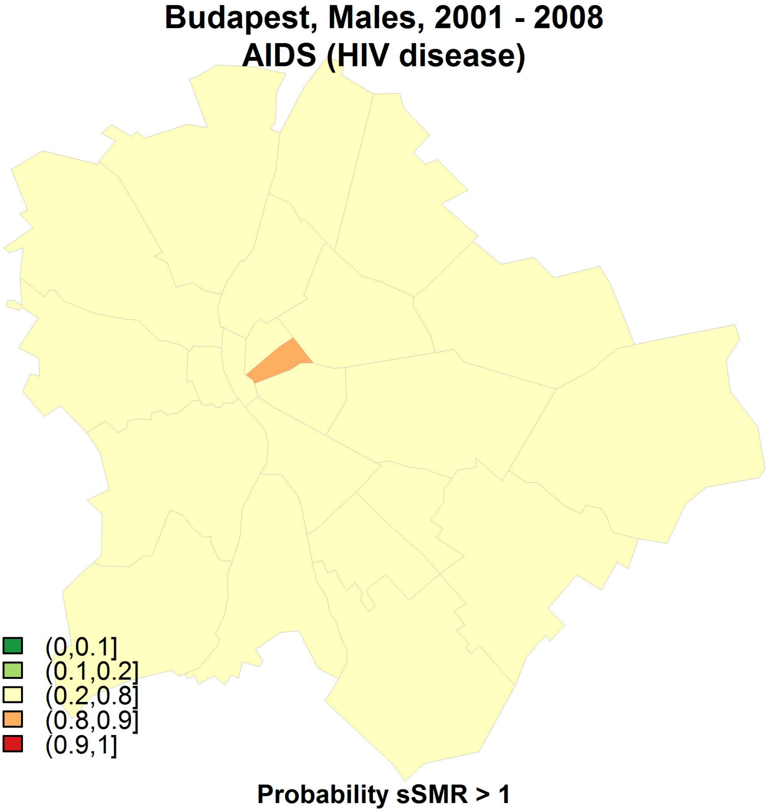

Budapest, Males, 2001 - 2008
AIDS (HIV disease)
(0,0.1]
(0.1,0.2]
(0.2,0.8]
(0.8,0.9]
(0.9,1]
Probability sSMR > 1

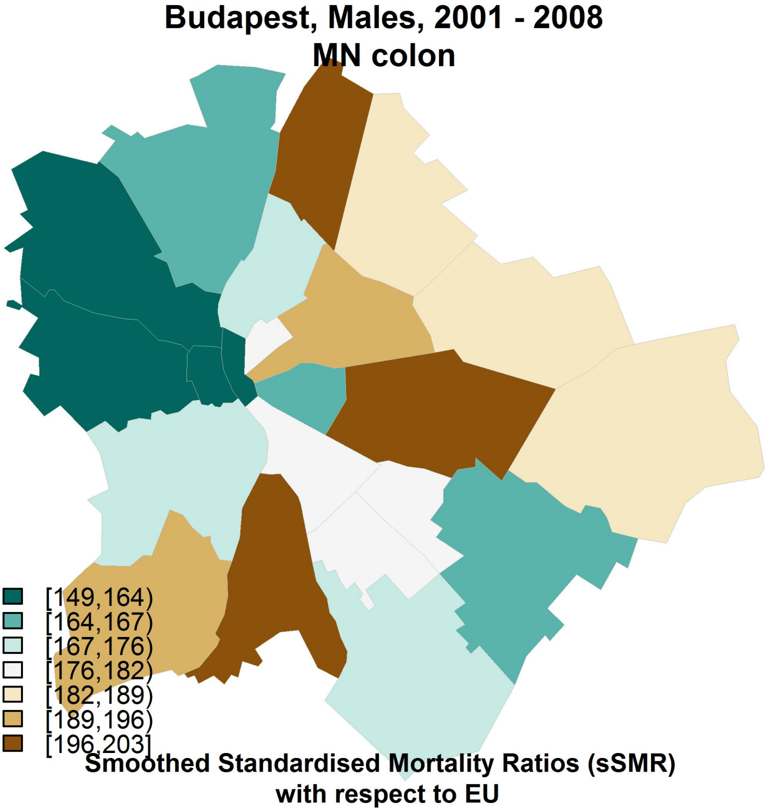
Budapest, Males, 2001 - 2008
MN colon
[149,164)
[164,167)
[167,176)
[176,182)
[182,189)
[189,196)
[196,203]
Smoothed Standardised Mortality Ratios (sSMR)
with respect to EU

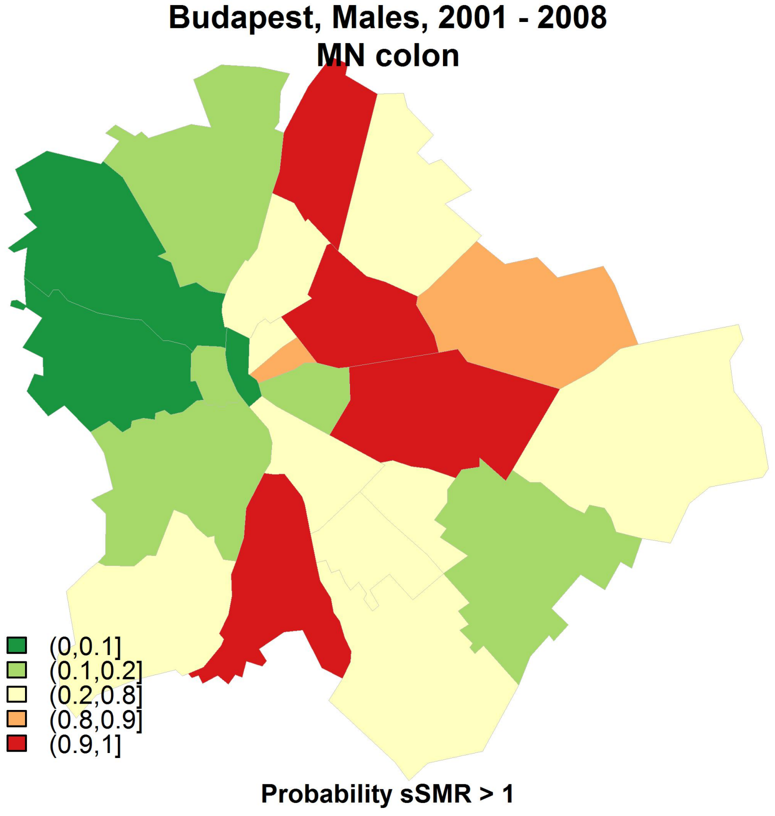
Budapest, Males, 2001 - 2008
MN colon
(0,0.1]
(0.1,0.2]
(0.2,0.8]
(0.8,0.9]
(0.9,1]
Probability sSMR > 1

Budapest, Males, 2001 - 2008
MN rectum, anus and anal canal

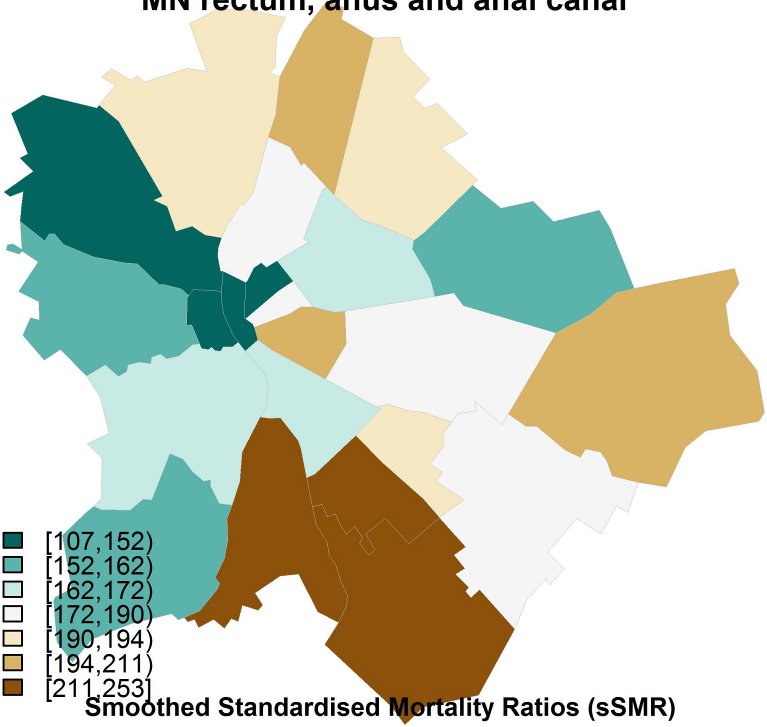

Smoothed Standardised Mortality Ratios (sSMR) with respect to EU

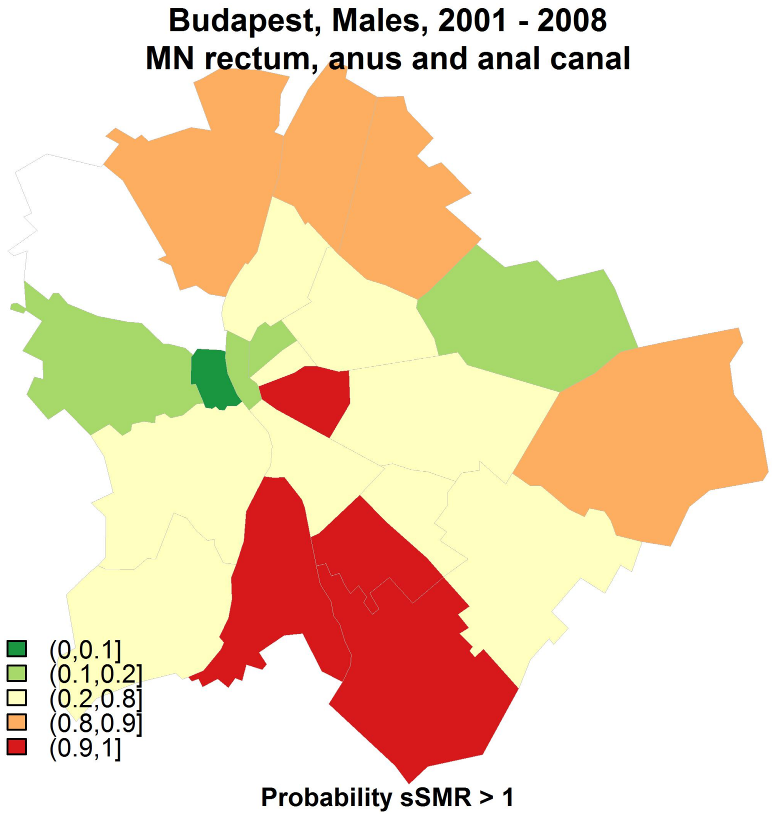
Budapest, Males, 2001 - 2008
MN rectum, anus and anal canal
(0,0.1]
(0.1,0.2]
(0.2,0.8]
(0.8,0.9]
(0.9,1]
Probability sSMR > 1

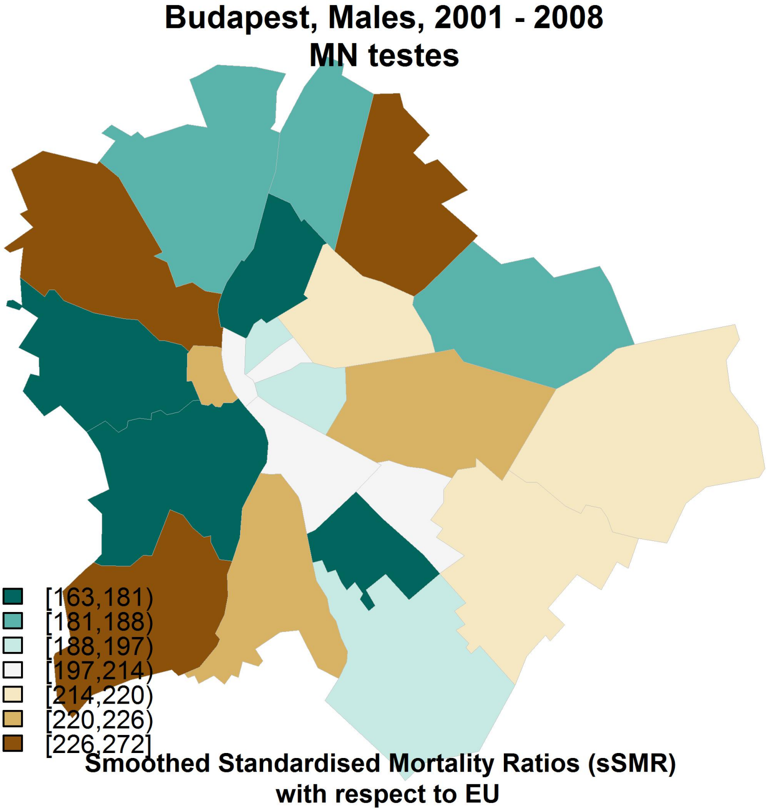

Budapest, Males, 2001 - 2008
MN testes
[163,181)
[181,188)
[188,197)
[197,214)
[214,220)
[220,226)
[226,272]
Smoothed Standardised Mortality Ratios (sSMR)
with respect to EU

Budapest, Males, 2001 - 2008
MN testes

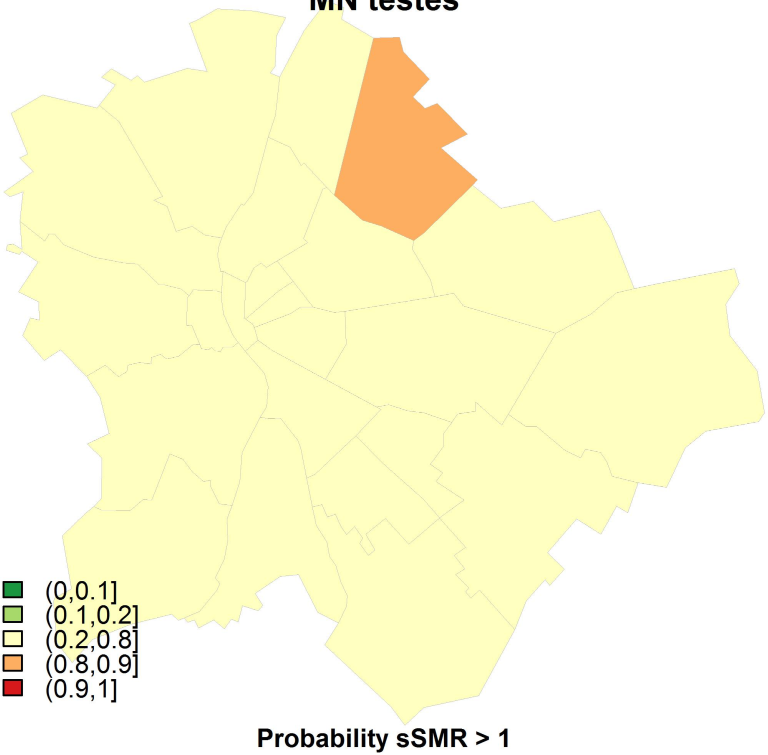

Budapest, Males, 2001 - 2008
Hodgkin's disease

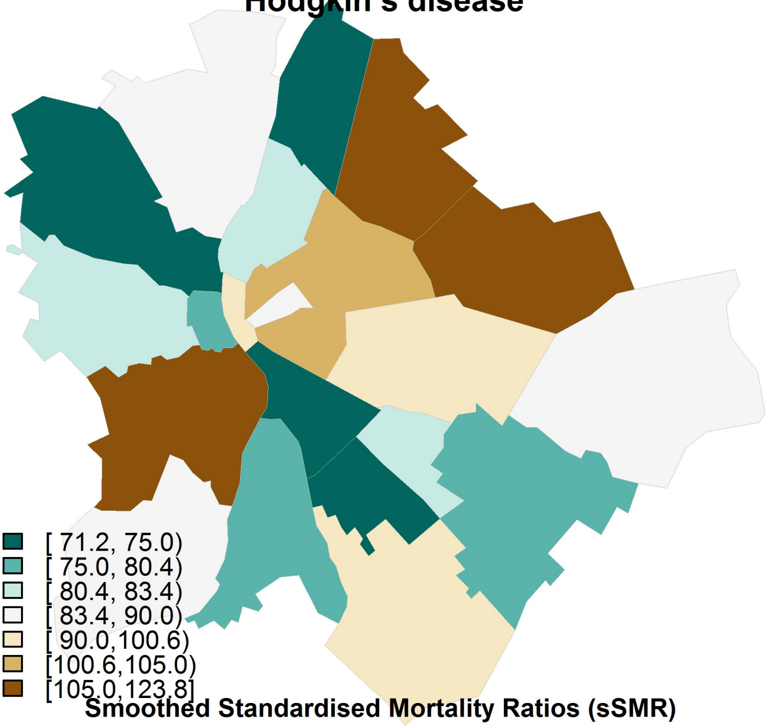

Smoothed Standardised Mortality Ratios (sSMR)
with respect to EU

Budapest, Males, 2001 - 2008
Hodgkin's disease

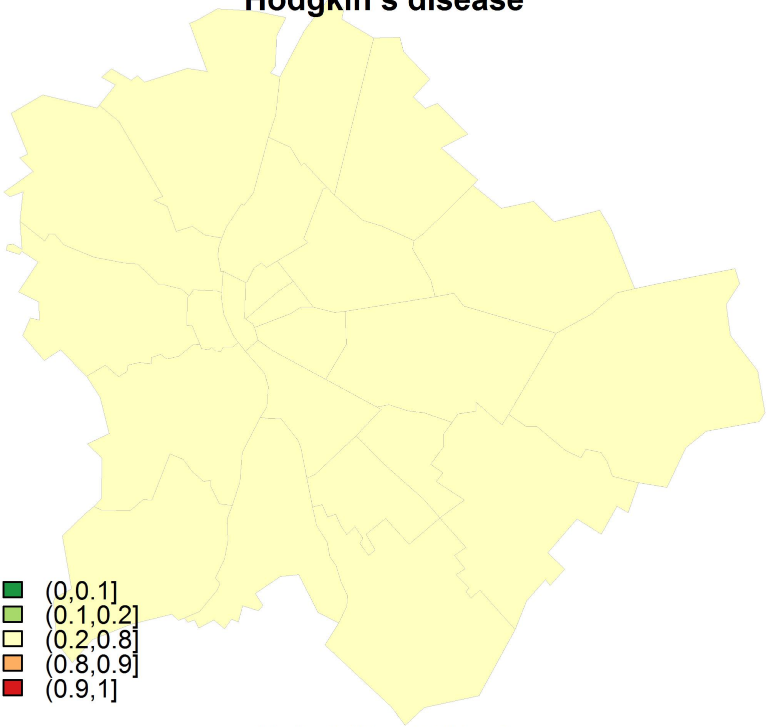

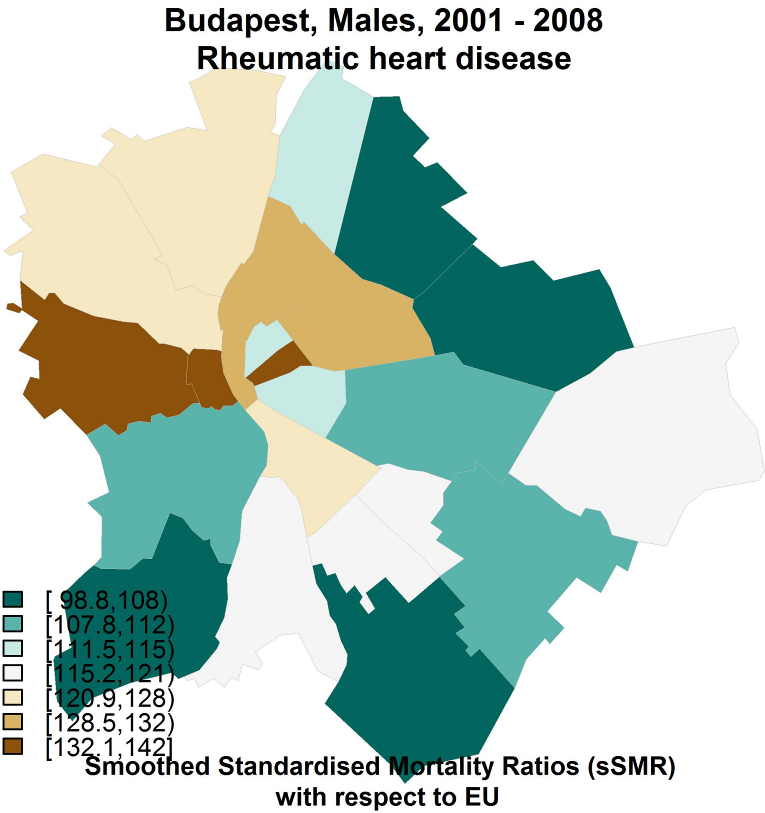
Budapest, Males, 2001 - 2008
Rheumatic heart disease
[98.8,108)
[107.8,112)
[111.5,115)
[115.2,121)
[120.9,128)
[128.5,132)
[132.1,142]
Smoothed Standardised Mortality Ratios (sSMR)
with respect to EU

Budapest, Males, 2001 - 2008
Rheumatic heart disease

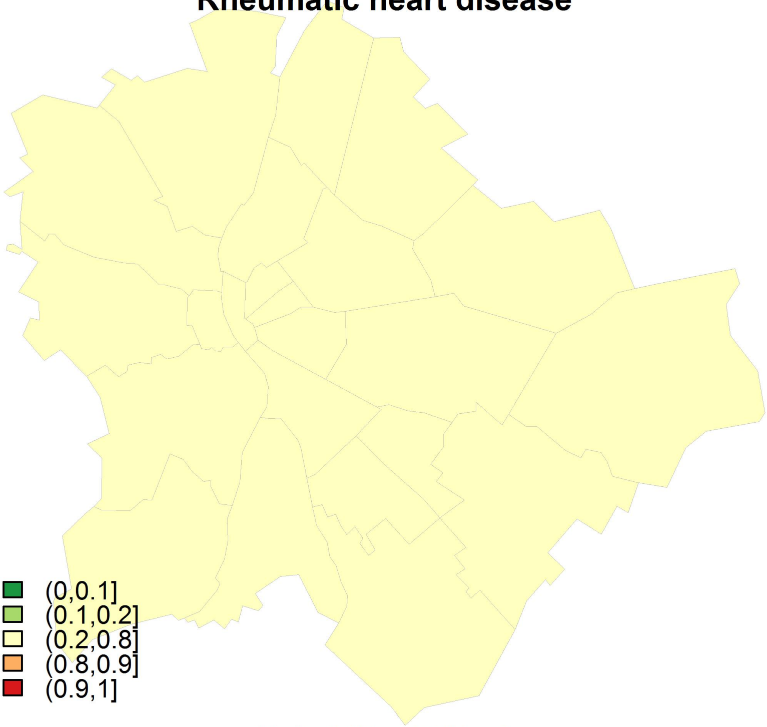

Budapest, Males, 2001 - 2008
Hypertension

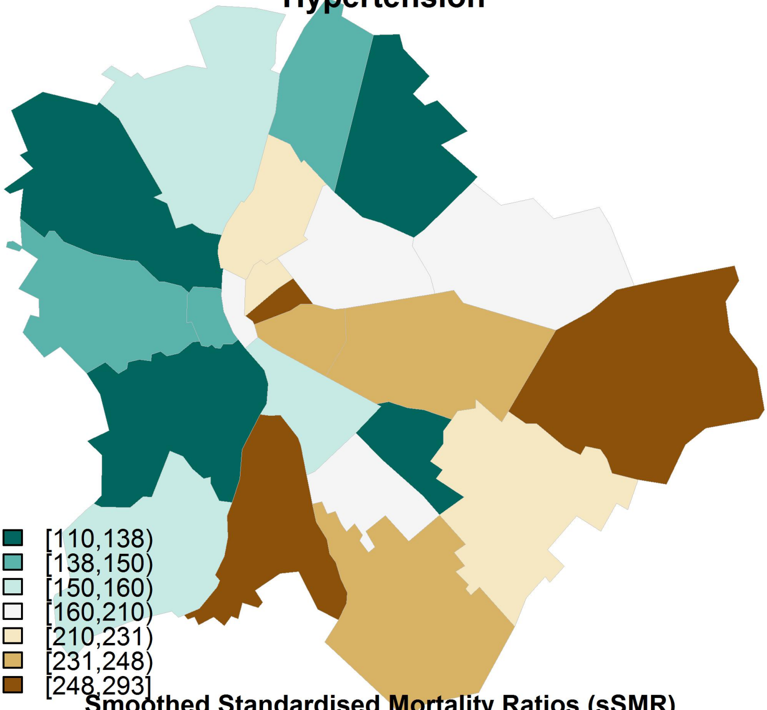

Smoothed Standardised Mortality Ratios (sSMR)
with respect to EU

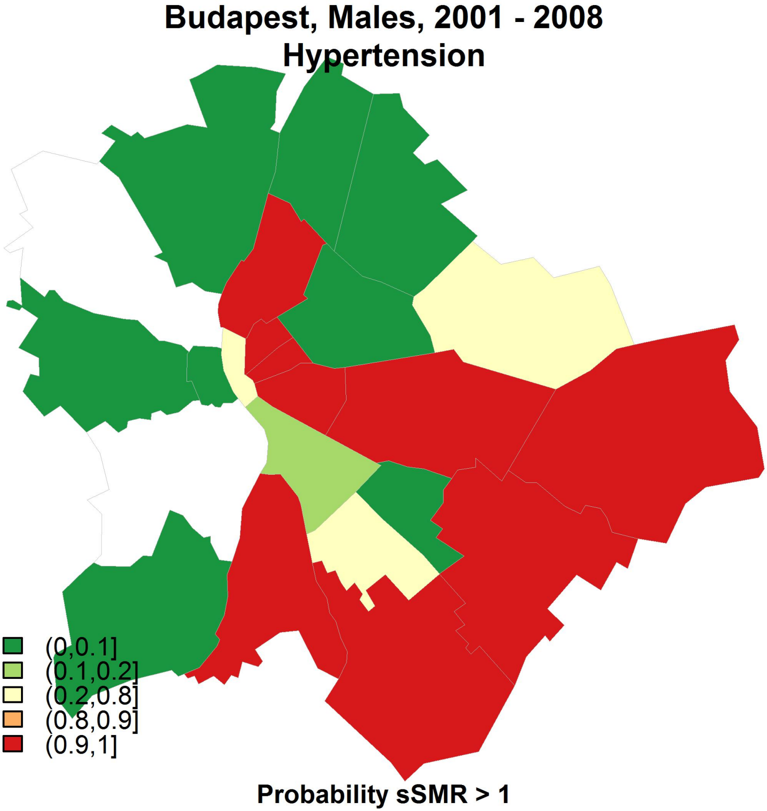

Budapest, Males, 2001 - 2008
Hypertension
(0,0.1]
(0.1,0.2]
(0.2,0.8]
(0.8,0.9]
(0.9,1]
Probability sSMR > 1

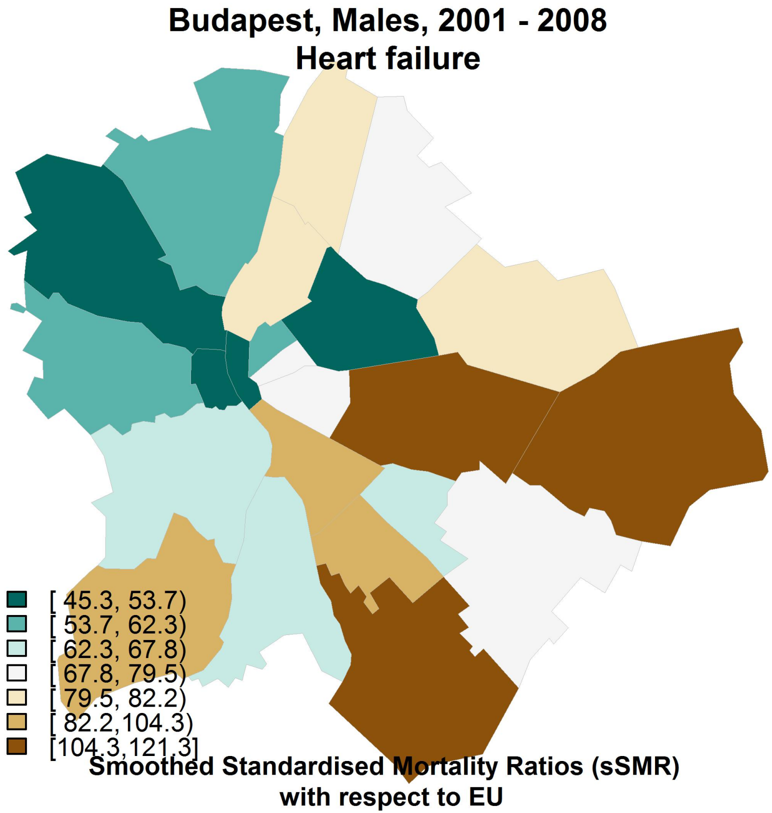
Budapest, Males, 2001 - 2008
Heart failure
[45.3, 53.7)
[53.7, 62.3)
[62.3, 67.8)
[67.8, 79.5)
[79.5, 82.2)
[82.2,104.3)
[104.3,121.3]
Smoothed Standardised Mortality Ratios (sSMR)
with respect to EU

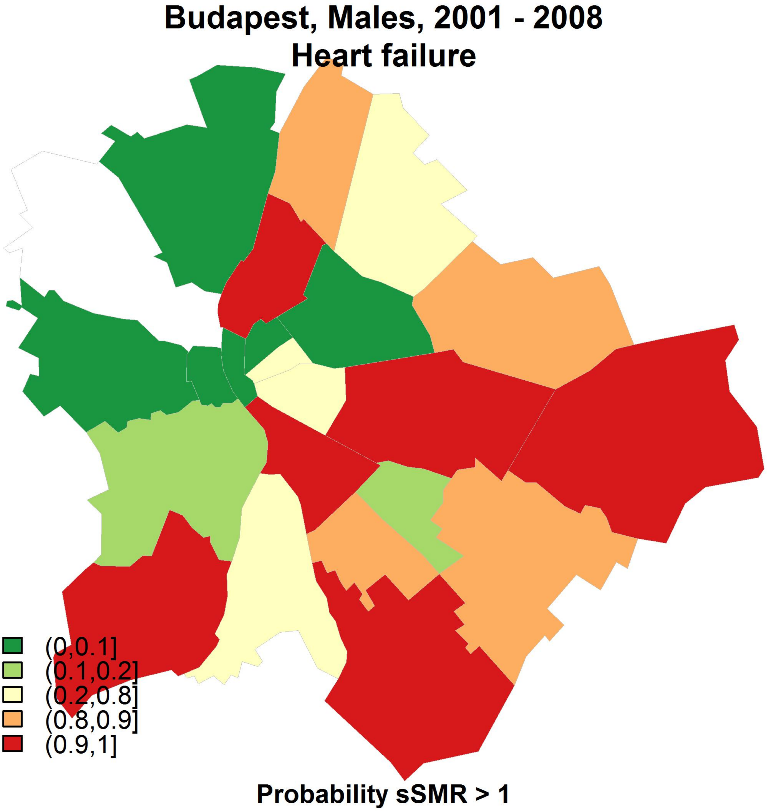
Budapest, Males, 2001 - 2008
Heart failure
(0,0.1]
(0.1,0.2]
(0.2,0.8]
(0.8,0.9]
(0.9,1]
Probability sSMR > 1

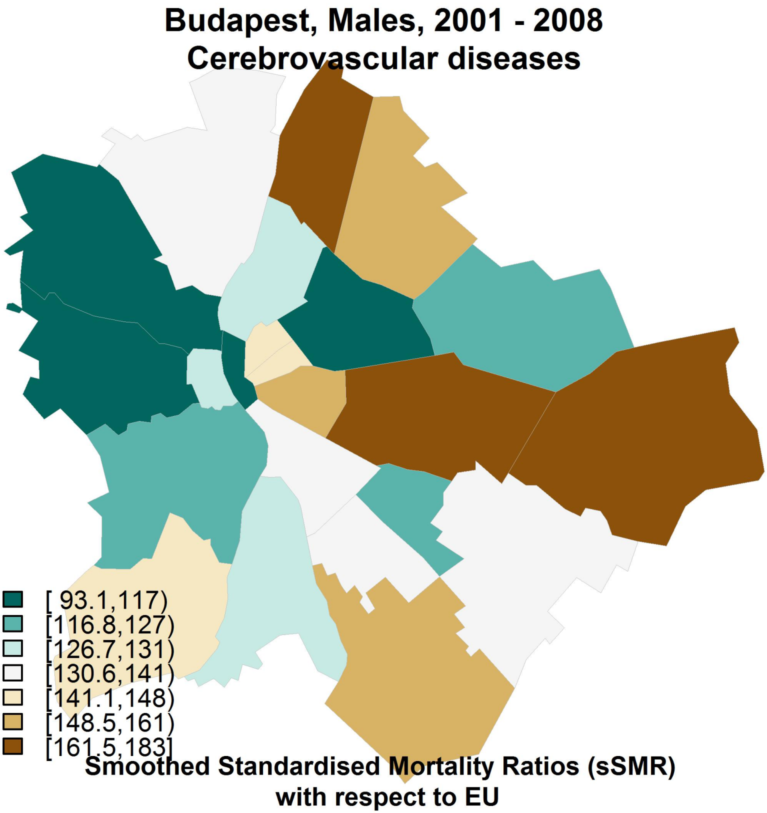
Budapest, Males, 2001 - 2008
Cerebrovascular diseases
[93.1,117)
[116.8,127)
[126.7,131)
[130.6,141)
[141.1,148)
[148.5,161)
[161.5,183]
Smoothed Standardised Mortality Ratios (sSMR)
with respect to EU

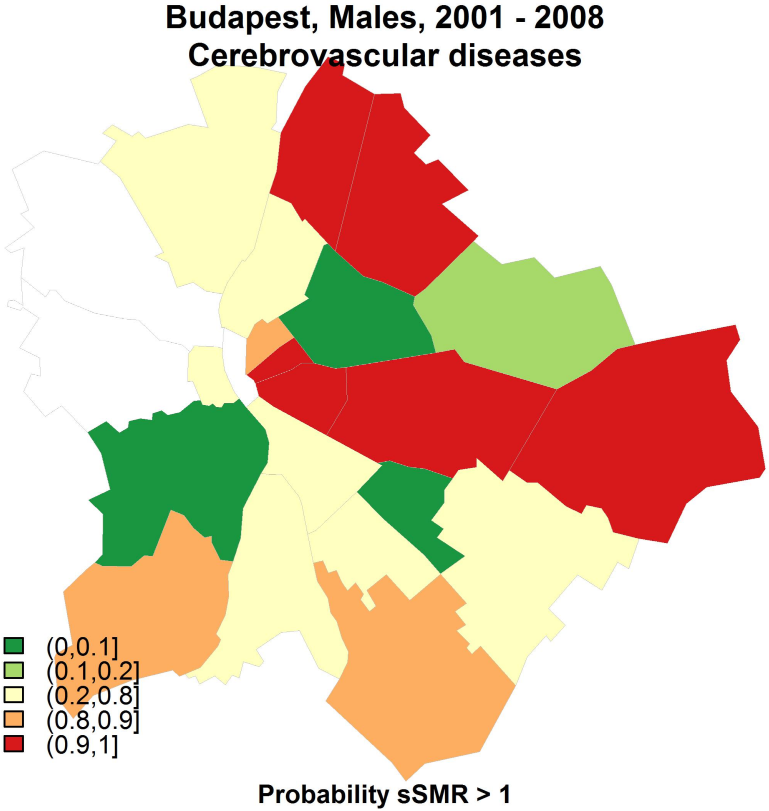

Budapest, Males, 2001 - 2008
Cerebrovascular diseases
(0,0.1]
(0.1,0.2]
(0.2,0.8]
(0.8,0.9]
(0.9,1]
Probability sSMR > 1

Budapest, Males, 2001 - 2008
Peptic ulcer

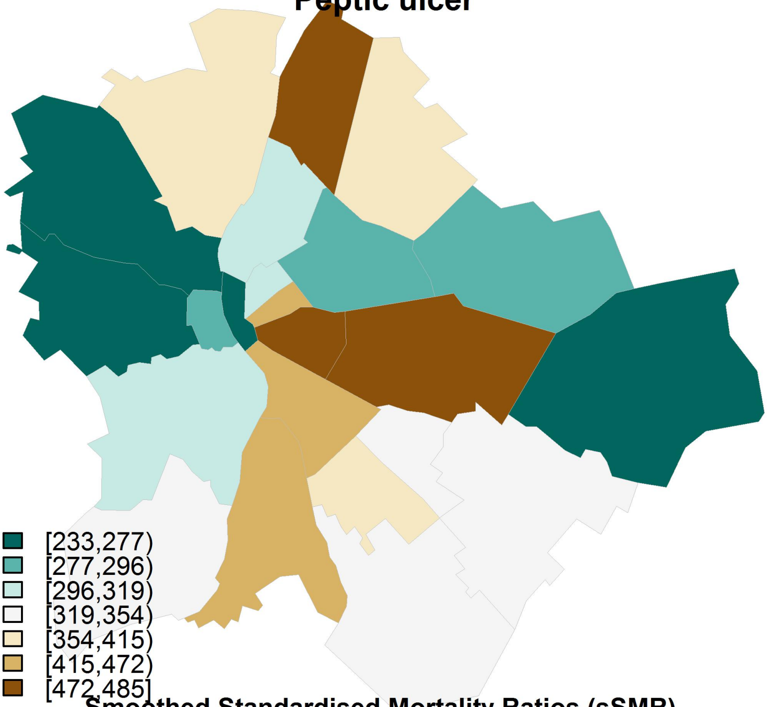

Smoothed Standardised Mortality Ratios (sSMR)
with respect to EU

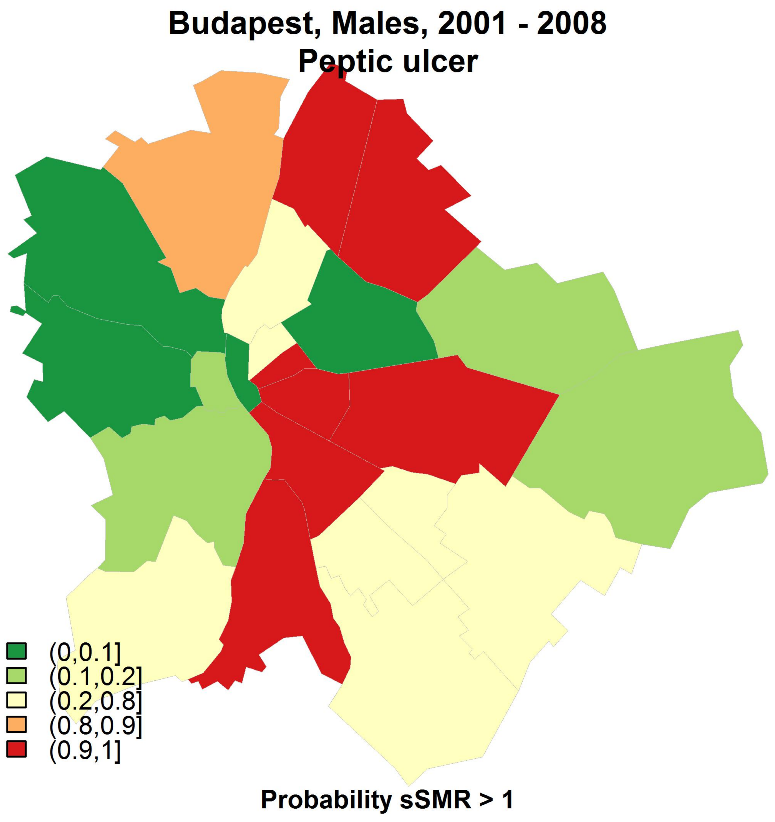
Budapest, Males, 2001 - 2008
Peptic ulcer
(0,0.1]
(0.1,0.2]
(0.2,0.8]
(0.8,0.9]
(0.9,1]
Probability sSMR > 1

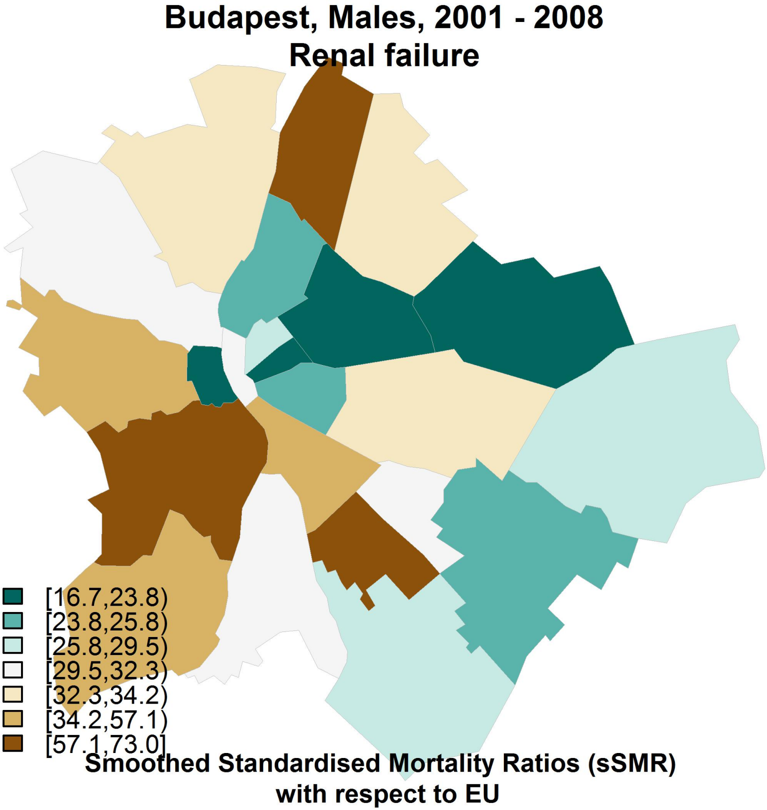
Budapest, Males, 2001 - 2008
Renal failure
[16.7,23.8)
[23.8,25.8)
[25.8,29.5)
[29.5,32.3)
[32.3,34.2)
[34.2,57.1)
[57.1,73.0]
Smoothed Standardised Mortality Ratios (sSMR)
with respect to EU

Budapest, Males, 2001 - 2008
Renal failure

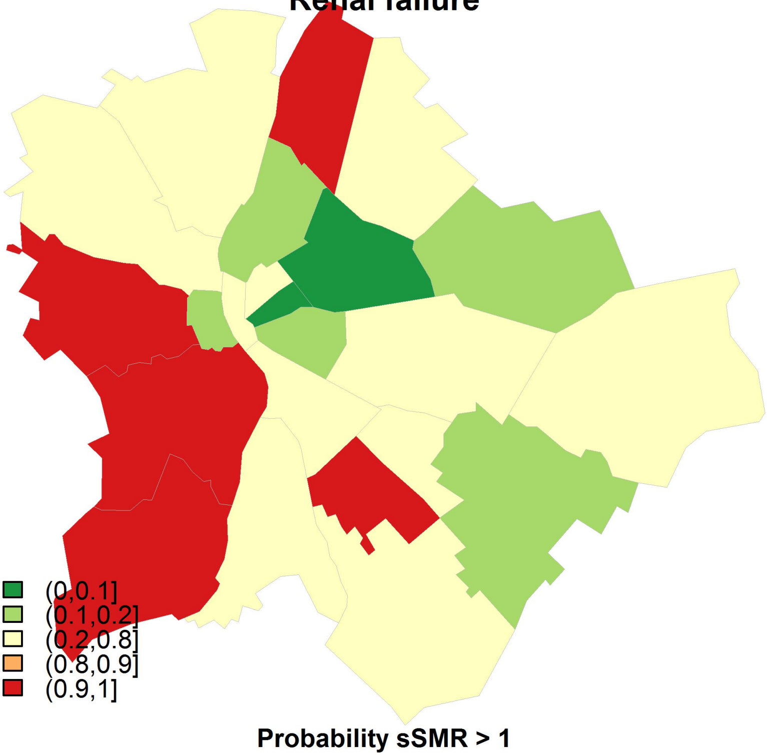

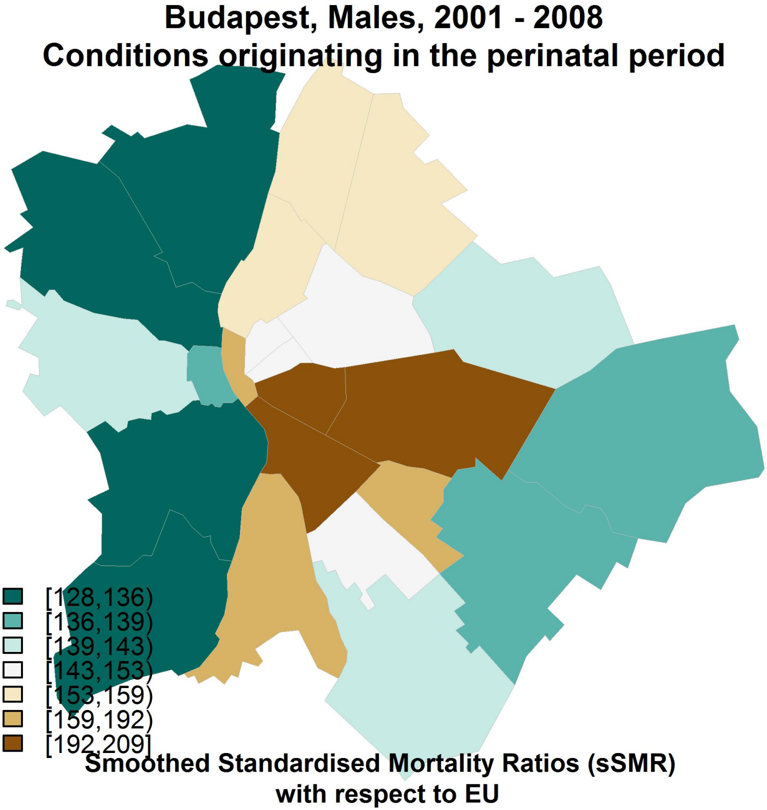
Budapest, Males, 2001 - 2008
Conditions originating in the perinatal period
[128,136)
[136,139)
[139,143)
[143,153)
[153,159)
[159,192)
[192,209]
Smoothed Standardised Mortality Ratios (sSMR)
with respect to EU

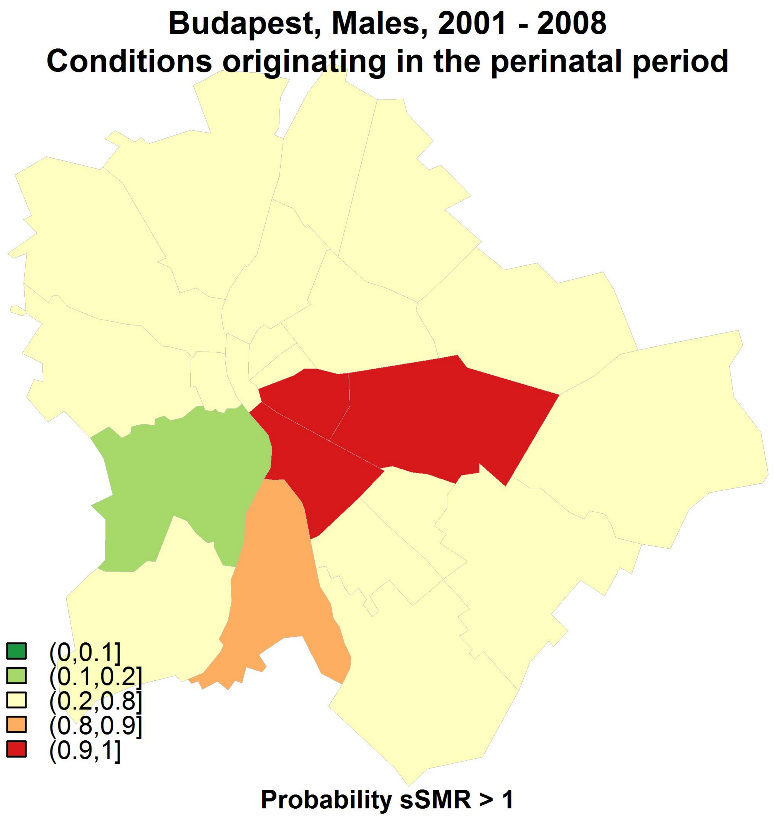
Budapest, Males, 2001 - 2008
Conditions originating in the perinatal period
(0,0.1]
(0.1,0.2]
(0.2,0.8]
(0.8,0.9]
(0.9,1]
Probability sSMR > 1

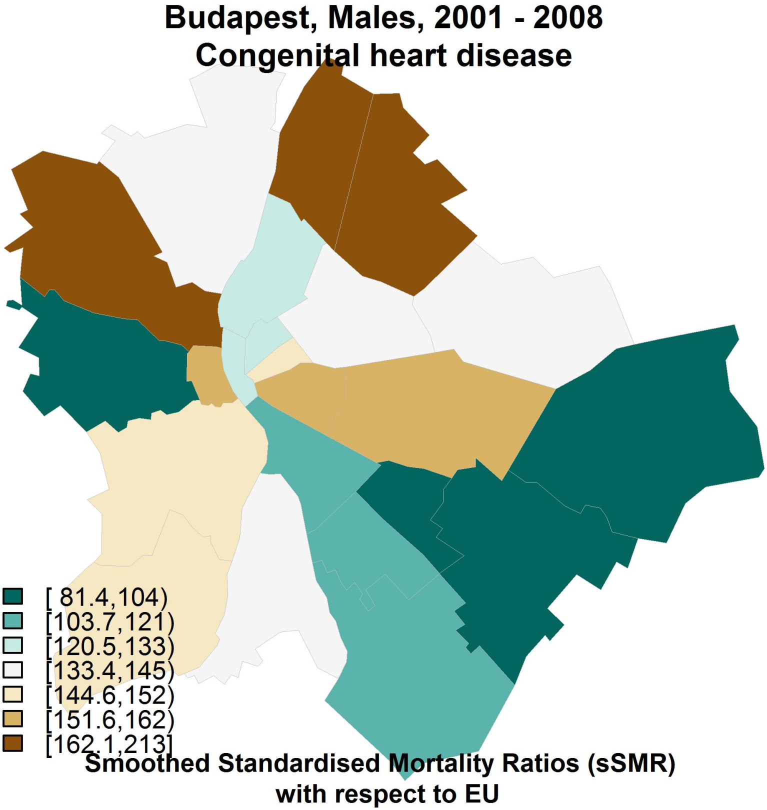
Budapest, Males, 2001 - 2008
Congenital heart disease
[81.4,104)
[103.7,121)
[120.5,133)
[133.4,145)
[144.6,152)
[151.6,162)
[162.1,213]
Smoothed Standardised Mortality Ratios (sSMR)
with respect to EU

Budapest, Males, 2001 - 2008
Congenital heart disease

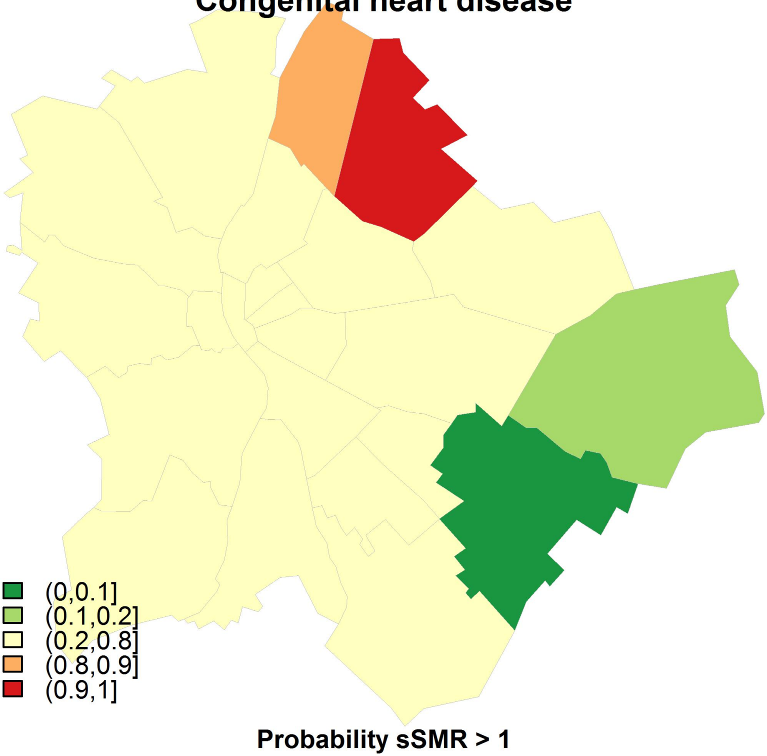

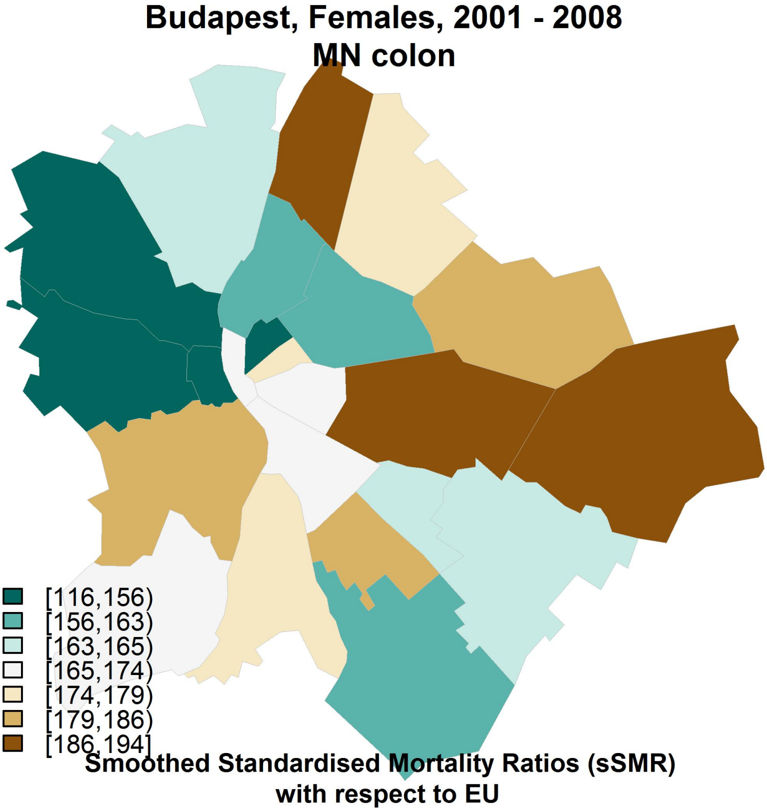
Budapest, Females, 2001 - 2008
MN colon
[116,156)
[156,163)
[163,165)
[165,174)
[174,179)
[179,186)
[186,194]
Smoothed Standardised Mortality Ratios (sSMR)
with respect to EU

Budapest, Females, 2001 - 2008
MN colon

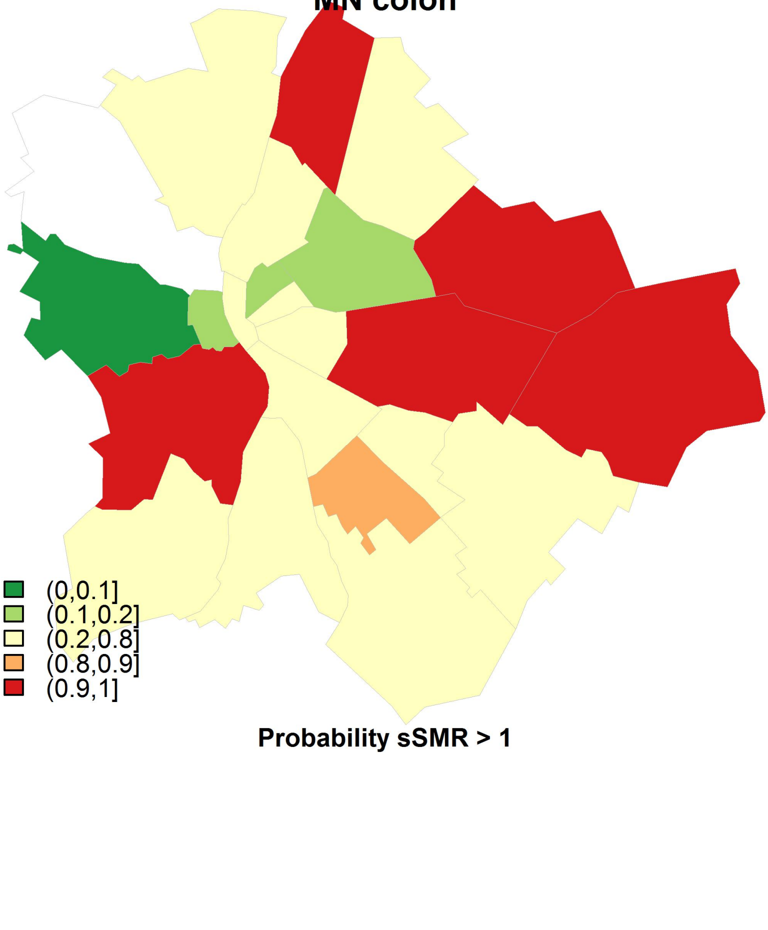

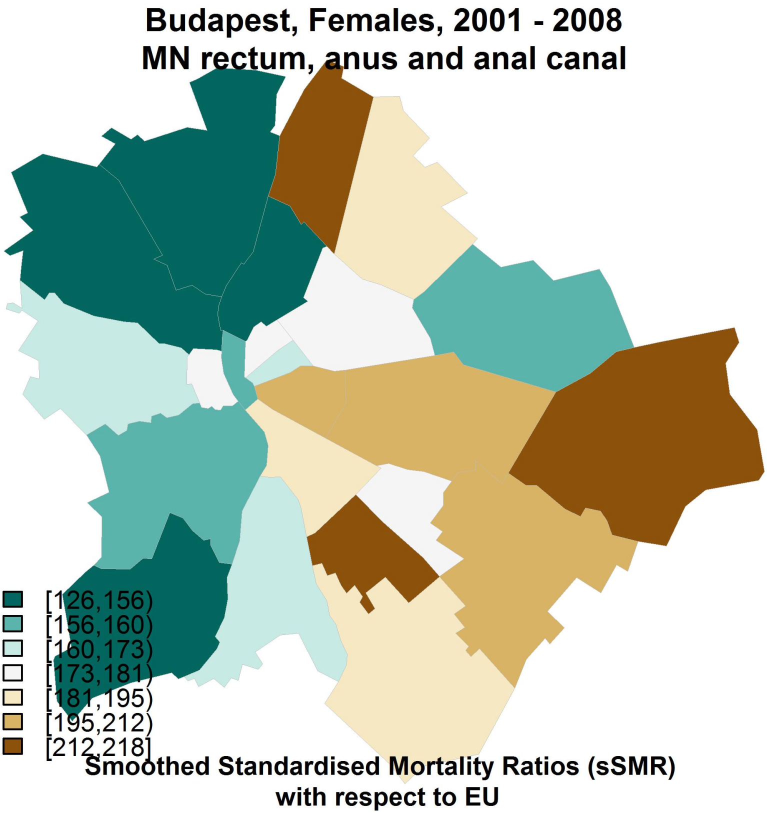
Budapest, Females, 2001 - 2008
MN rectum, anus and anal canal
[126,156)
[156,160)
[160,173)
[173,181)
[181,195)
[195,212)
[212,218]
Smoothed Standardised Mortality Ratios (sSMR)
with respect to EU

Budapest, Females, 2001 - 2008
MN rectum, anus and anal canal

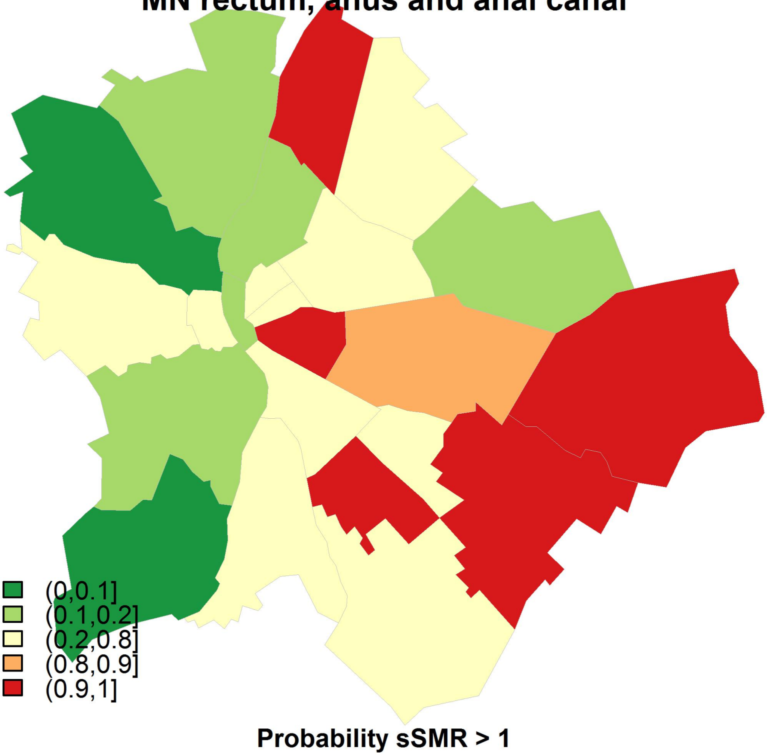

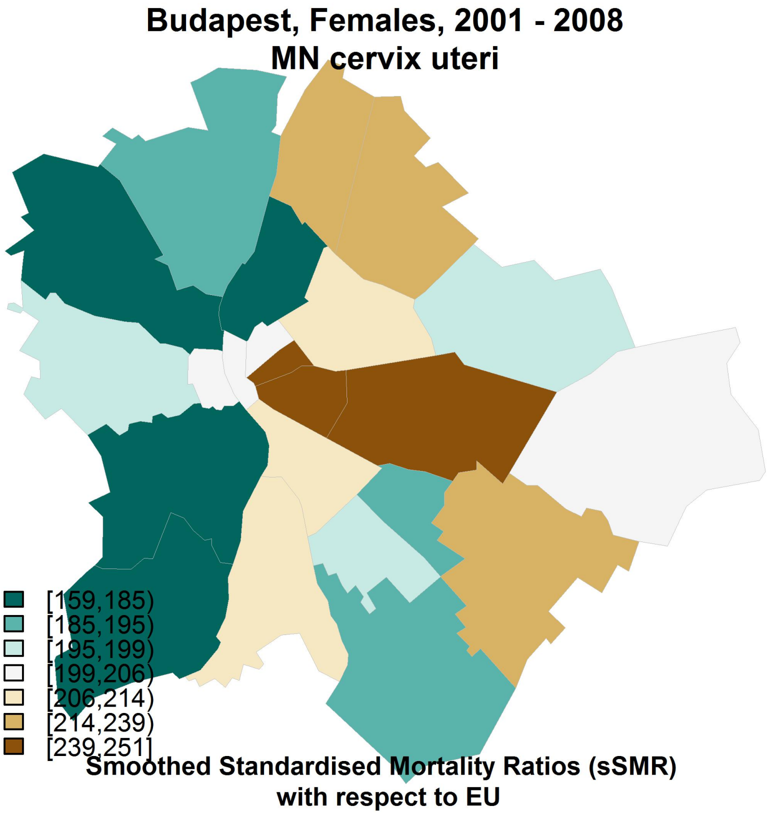
Budapest, Females, 2001 - 2008
MN cervix uteri
[159,185)
[185,195)
[195,199)
[199,206)
[206,214)
[214,239)
[239,251]
Smoothed Standardised Mortality Ratios (sSMR)
with respect to EU

Budapest, Females, 2001 - 2008
MN cervix uteri

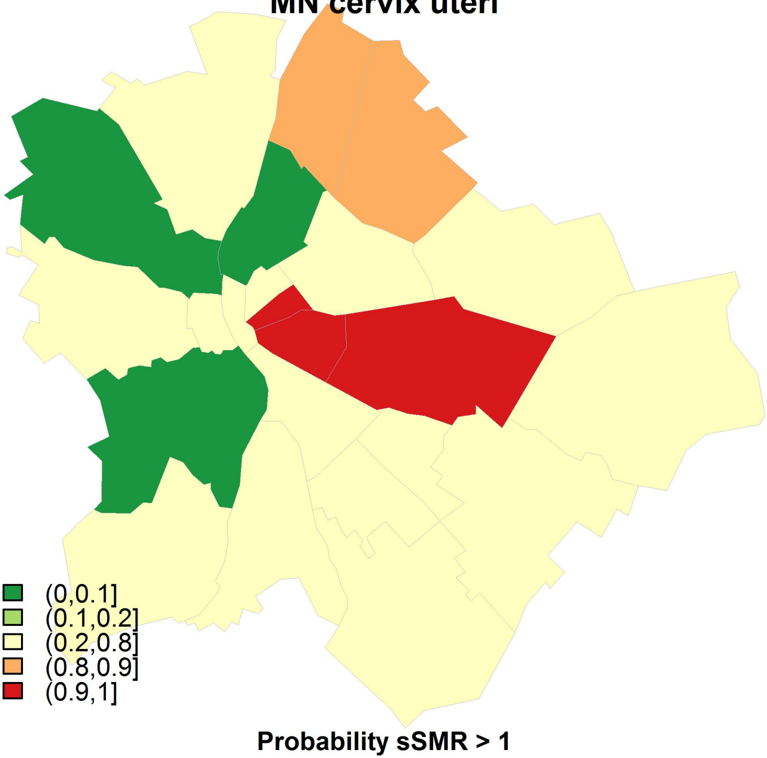

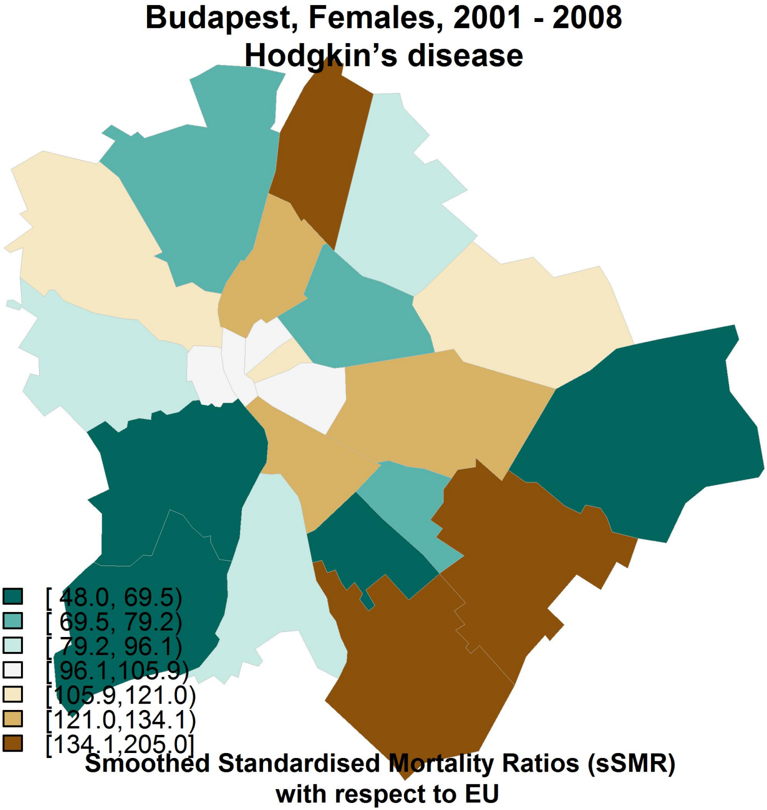
Budapest, Females, 2001 - 2008
Hodgkin's disease
[48.0, 69.5)
[69.5, 79.2)
[79.2, 96.1)
[96.1,105.9)
[105.9,121.0)
[121.0,134.1)
[134.1,205.0]
Smoothed Standardised Mortality Ratios (sSMR)
with respect to EU

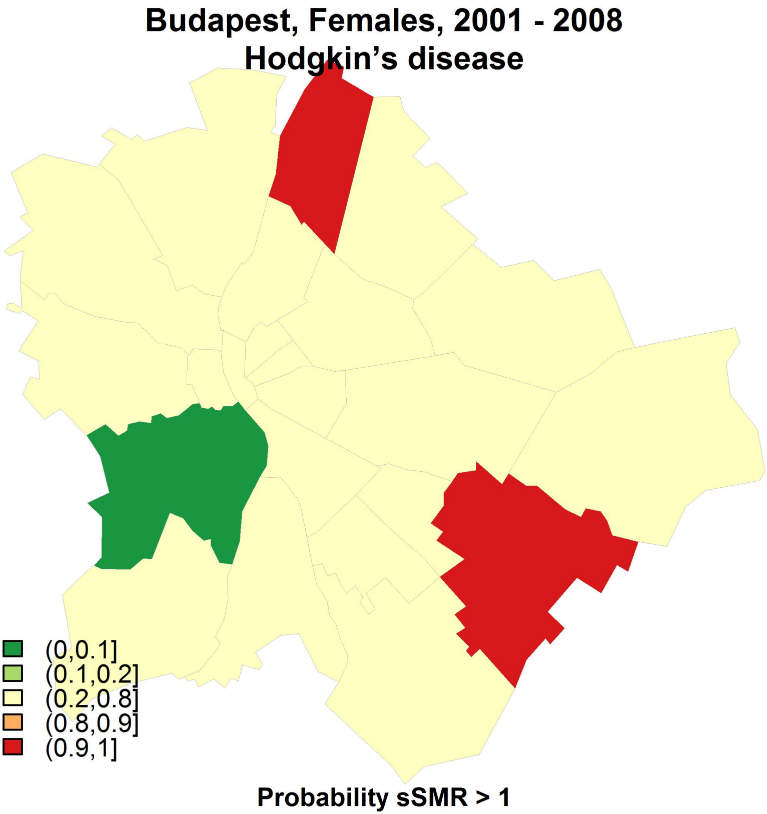
Budapest, Females, 2001 - 2008
Hodgkin's disease
(0,0.1]
(0.1,0.2]
(0.2,0.8]
(0.8,0.9]
(0.9,1]
Probability sSMR > 1

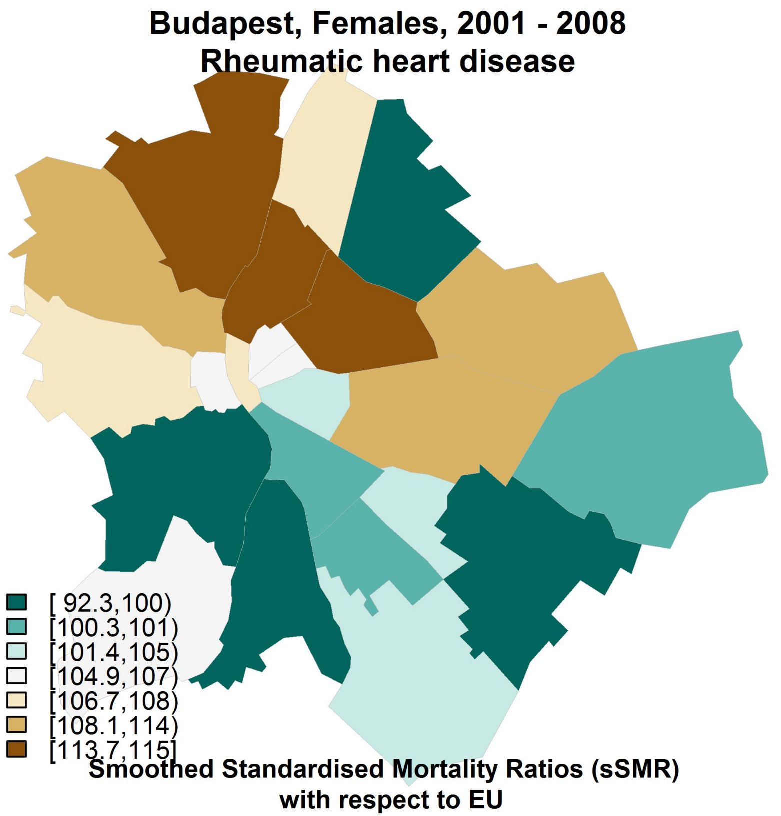
Budapest, Females, 2001 - 2008
Rheumatic heart disease
[92.3,100)
[100.3,101)
[101.4,105)
[104.9,107)
[106.7,108)
[108.1,114)
[113.7,115]
Smoothed Standardised Mortality Ratios (sSMR)
with respect to EU

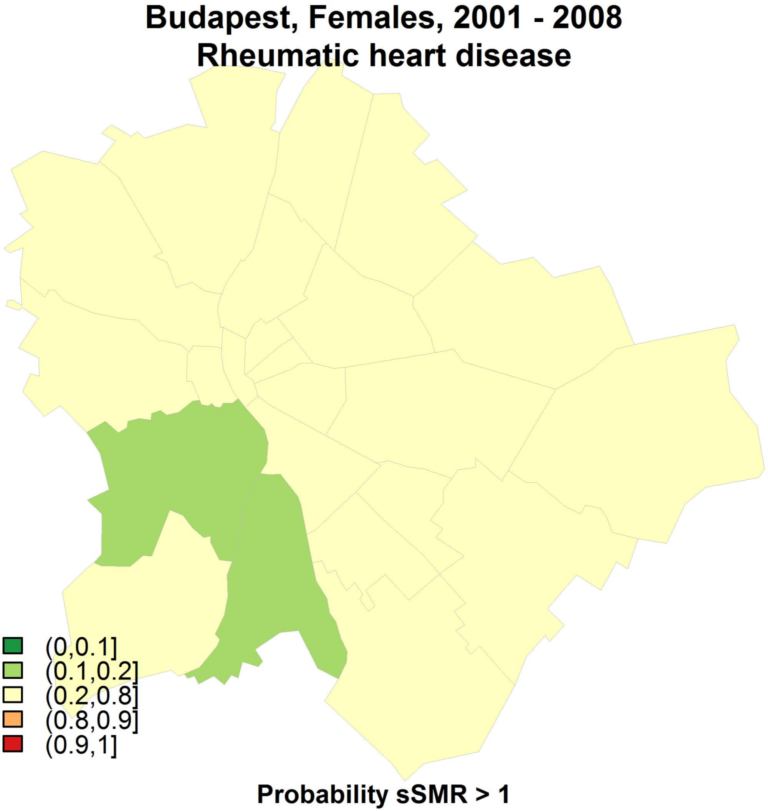
Budapest, Females, 2001 - 2008
Rheumatic heart disease
(0,0.1]
(0.1,0.2]
(0.2,0.8]
(0.8,0.9]
(0.9,1]
Probability sSMR > 1

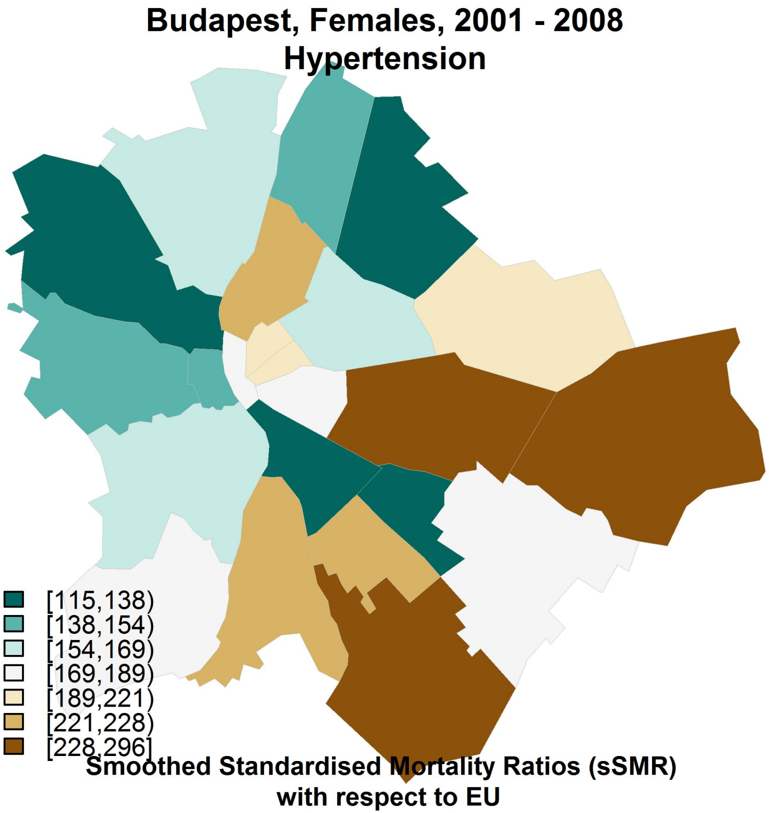

Budapest, Females, 2001 - 2008
Hypertension
[115,138)
[138,154)
[154,169)
[169,189)
[189,221)
[221,228)
[228,296]
Smoothed Standardised Mortality Ratios (sSMR)
with respect to EU

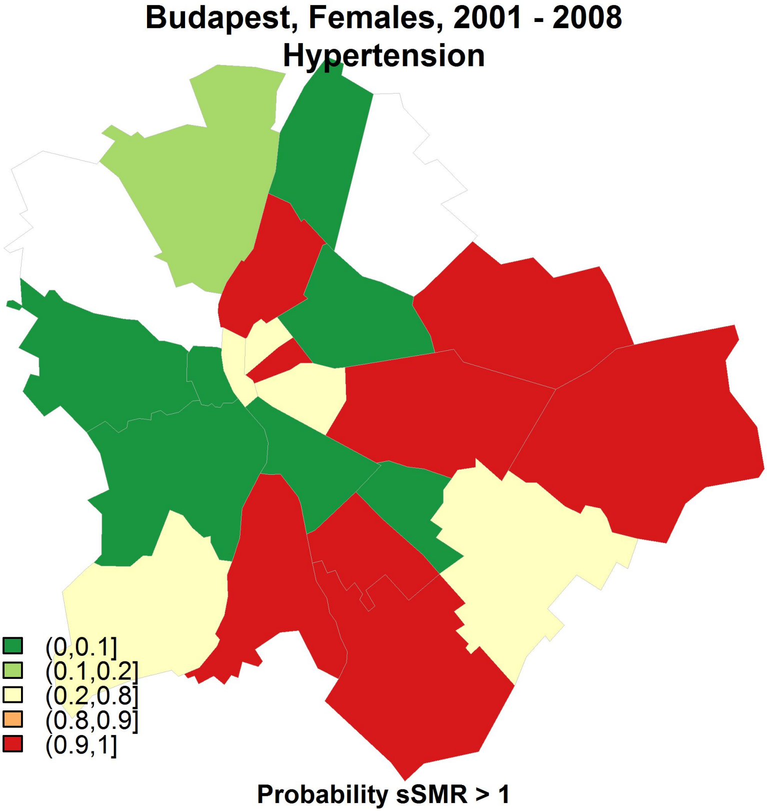
Budapest, Females, 2001 - 2008
Hypertension
(0,0.1]
(0.1,0.2]
(0.2,0.8]
(0.8,0.9]
(0.9,1]
Probability sSMR > 1

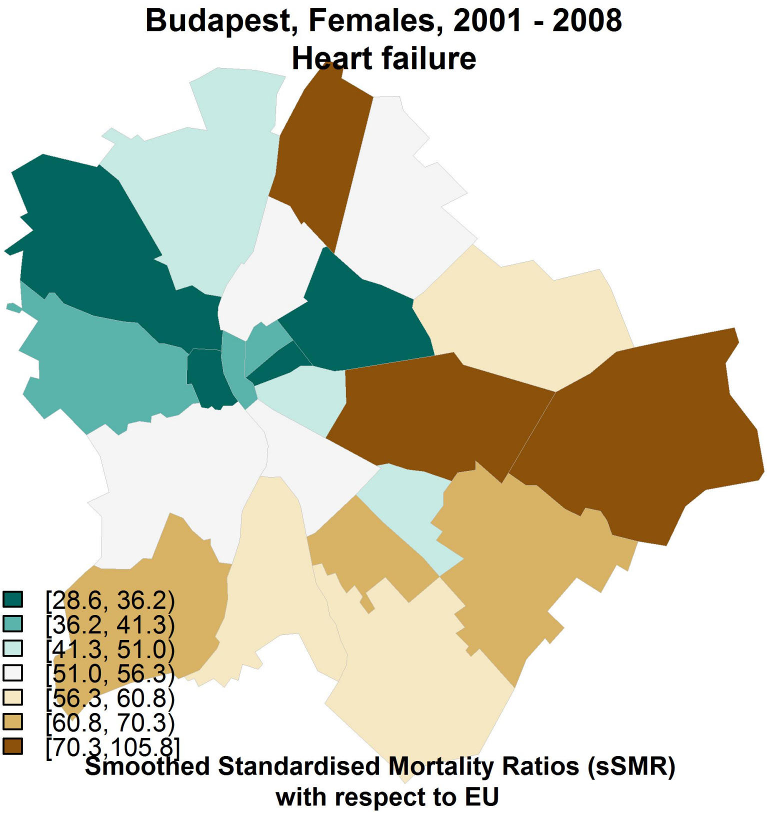
Budapest, Females, 2001 - 2008
Heart failure
[28.6, 36.2)
[36.2, 41.3)
[41.3, 51.0)
[51.0, 56.3)
[56.3, 60.8)
[60.8, 70.3)
[70.3,105.8]
Smoothed Standardised Mortality Ratios (sSMR)
with respect to EU

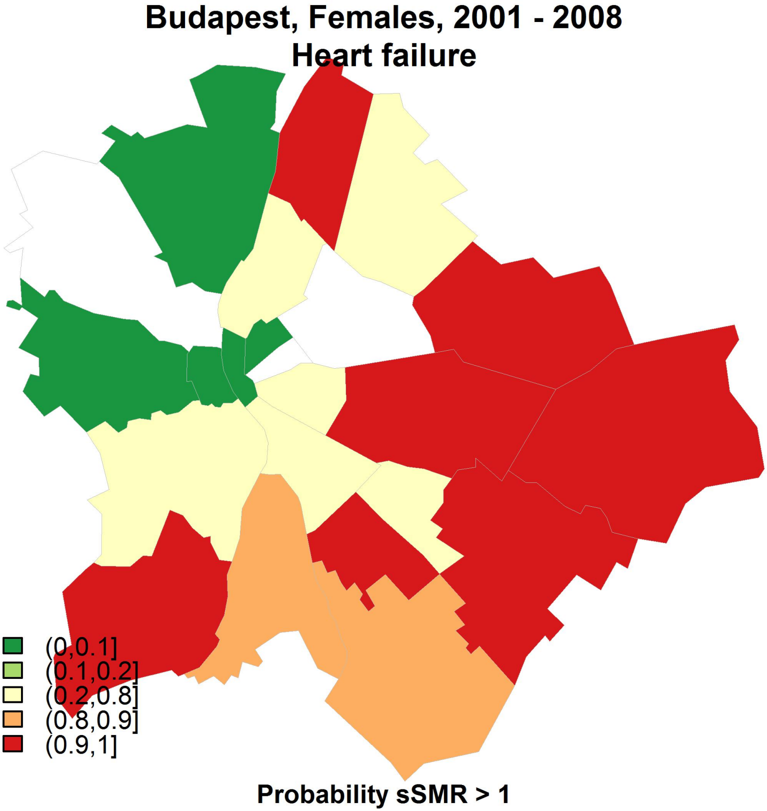
Budapest, Females, 2001 - 2008
Heart failure
(0,0.1]
(0.1,0.2]
(0.2,0.8]
(0.8,0.9]
(0.9,1]
Probability sSMR > 1

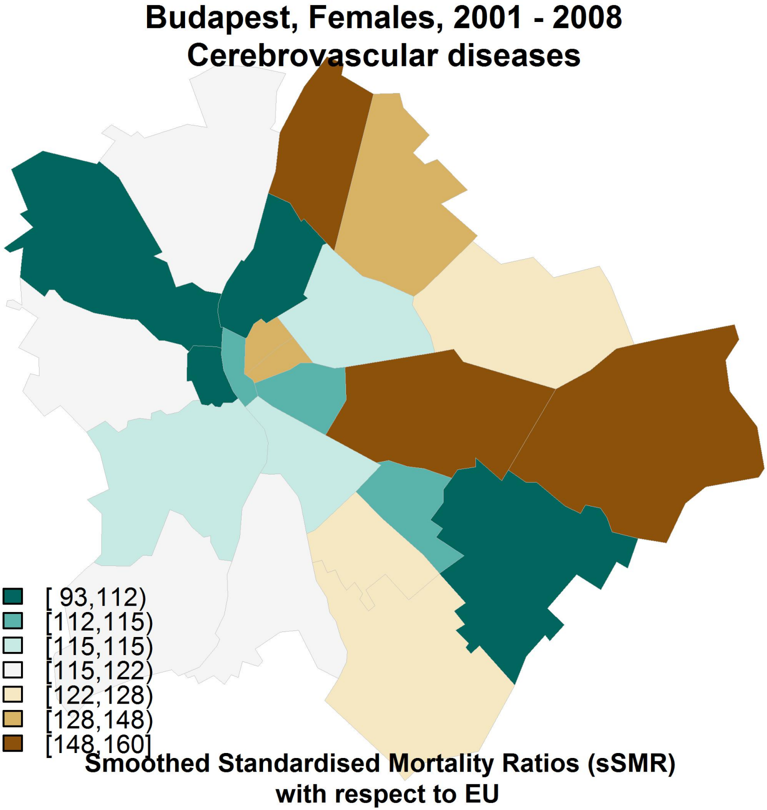

Budapest, Females, 2001 - 2008
Cerebrovascular diseases
[93,112)
[112,115)
[115,115)
[115,122)
[122,128)
[128,148)
[148,160]
Smoothed Standardised Mortality Ratios (sSMR)
with respect to EU

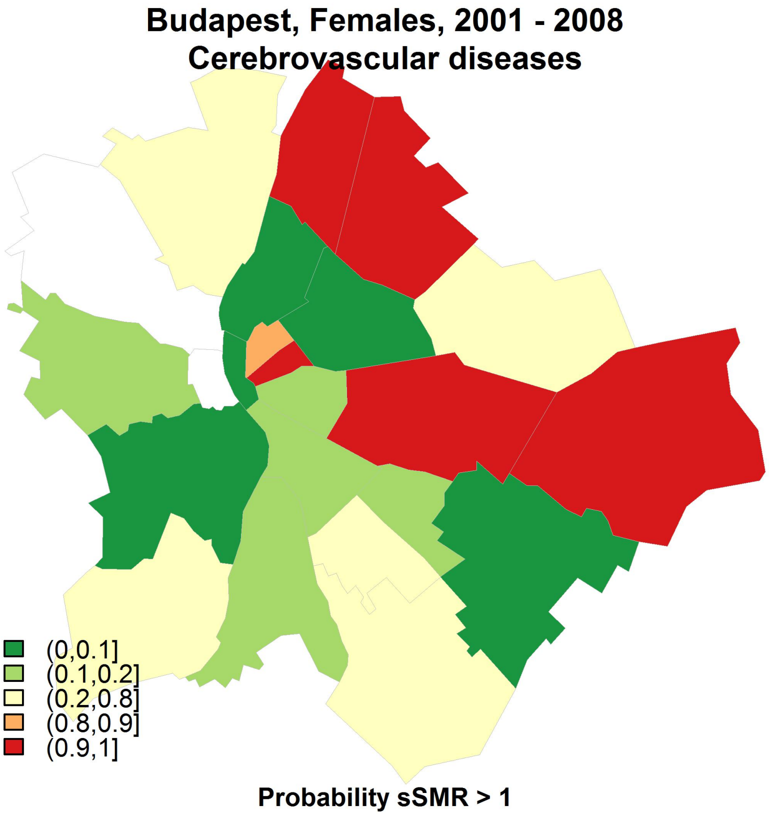
Budapest, Females, 2001 - 2008
Cerebrovascular diseases
(0,0.1]
(0.1,0.2]
(0.2,0.8]
(0.8,0.9]
(0.9,1]
Probability sSMR > 1

Budapest, Females, 2001 - 2008
Peptic ulcer

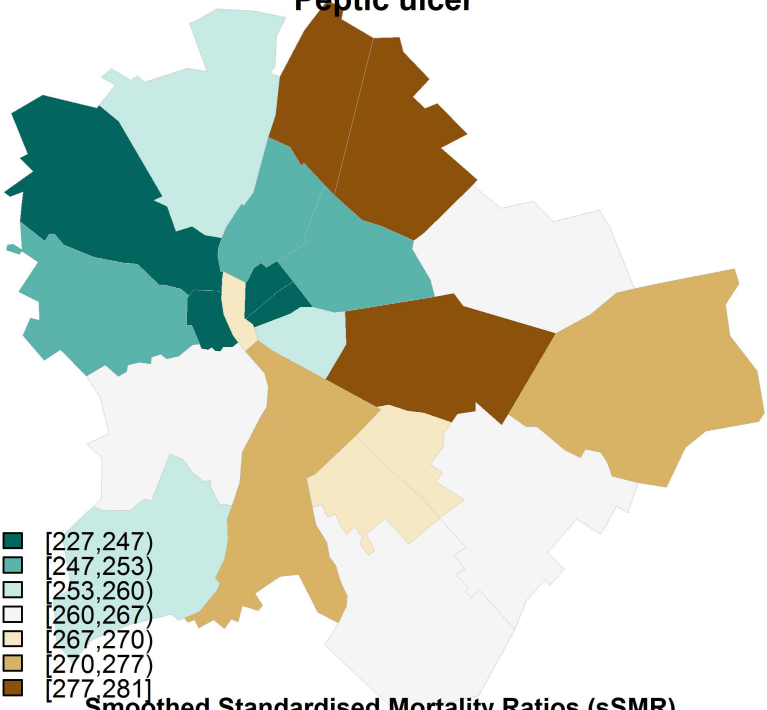

Smoothed Standardised Mortality Ratios (sSMR)
with respect to EU

Budapest, Females, 2001 - 2008
Peptic ulcer

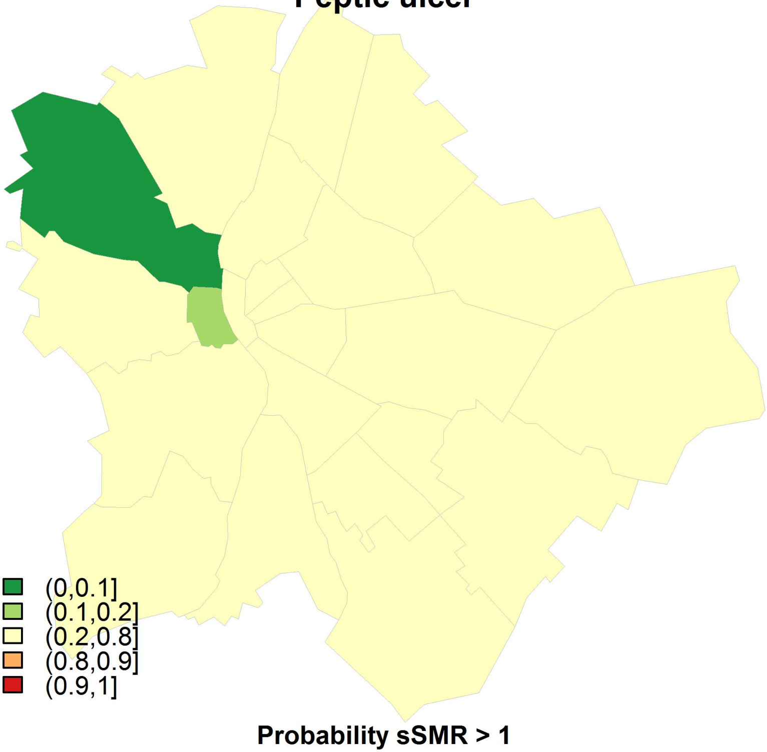

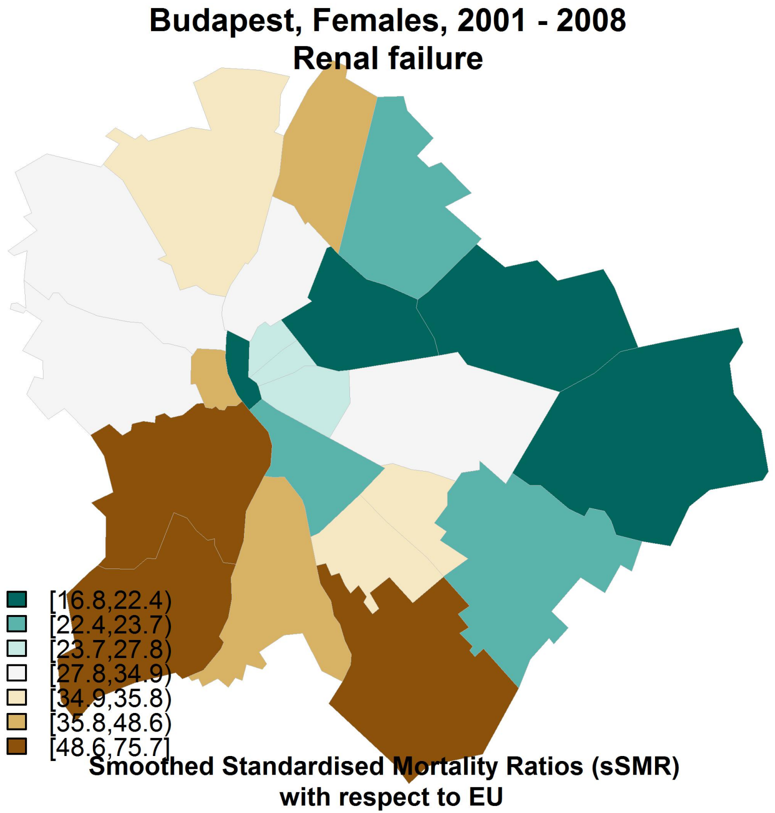
Budapest, Females, 2001 - 2008
Renal failure
[16.8,22.4)
[22.4,23.7)
[23.7,27.8)
[27.8,34.9)
[34.9,35.8)
[35.8,48.6)
[48.6,75.7]
Smoothed Standardised Mortality Ratios (sSMR)
with respect to EU

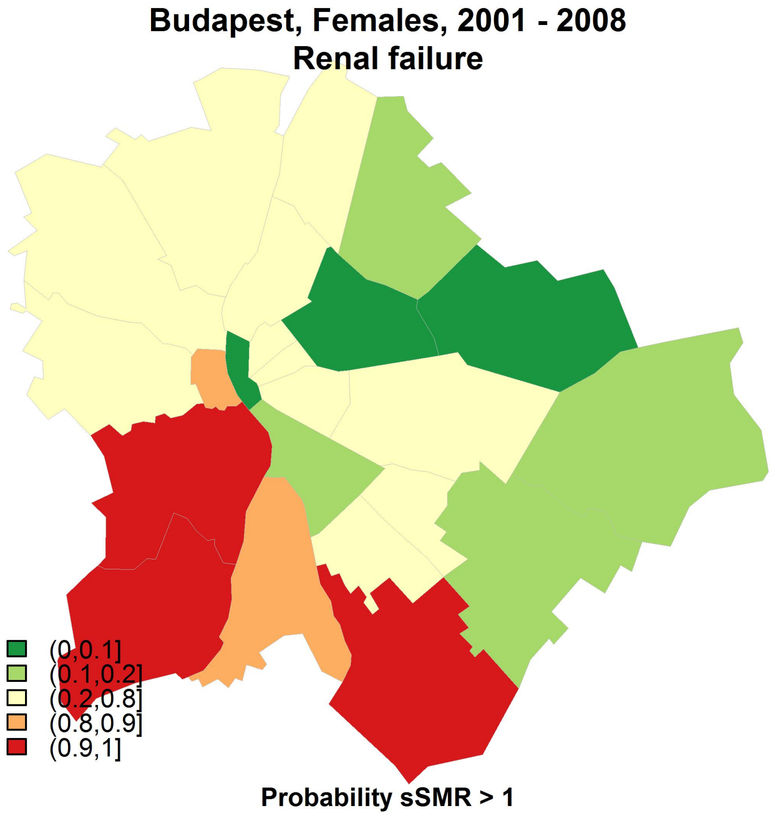
Budapest, Females, 2001 - 2008
Renal failure
(0,0.1]
(0.1,0.2]
(0.2,0.8]
(0.8,0.9]
(0.9,1]
Probability sSMR > 1

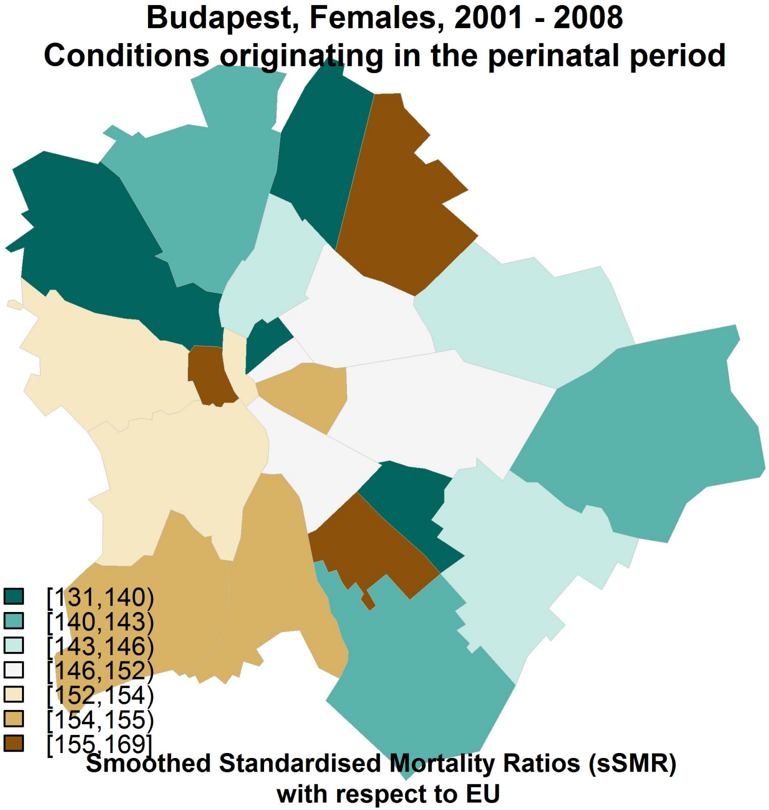
Budapest, Females, 2001 - 2008
Conditions originating in the perinatal period
[131,140)
[140,143)
[143,146)
[146,152)
[152,154)
[154,155)
[155,169]
Smoothed Standardised Mortality Ratios (sSMR)
with respect to EU

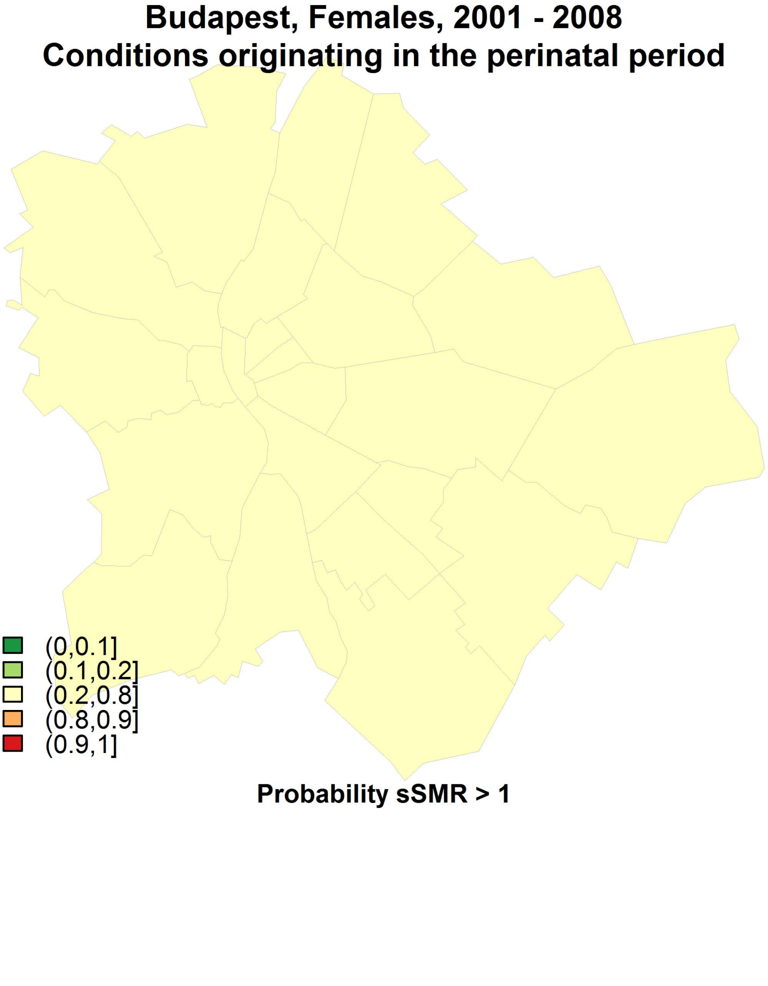
Budapest, Females, 2001 - 2008
Conditions originating in the perinatal period
(0,0.1]
(0.1,0.2]
(0.2,0.8]
(0.8,0.9]
(0.9,1]
Probability sSMR > 1

Budapest, Females, 2001 - 2008
Congenital heart disease

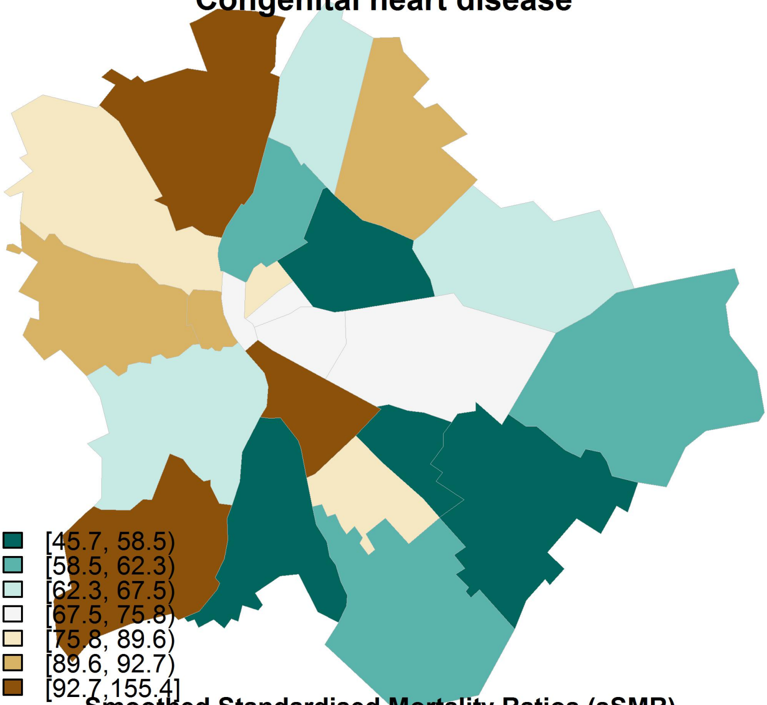

Smoothed Standardised Mortality Ratios (sSMR)
with respect to EU

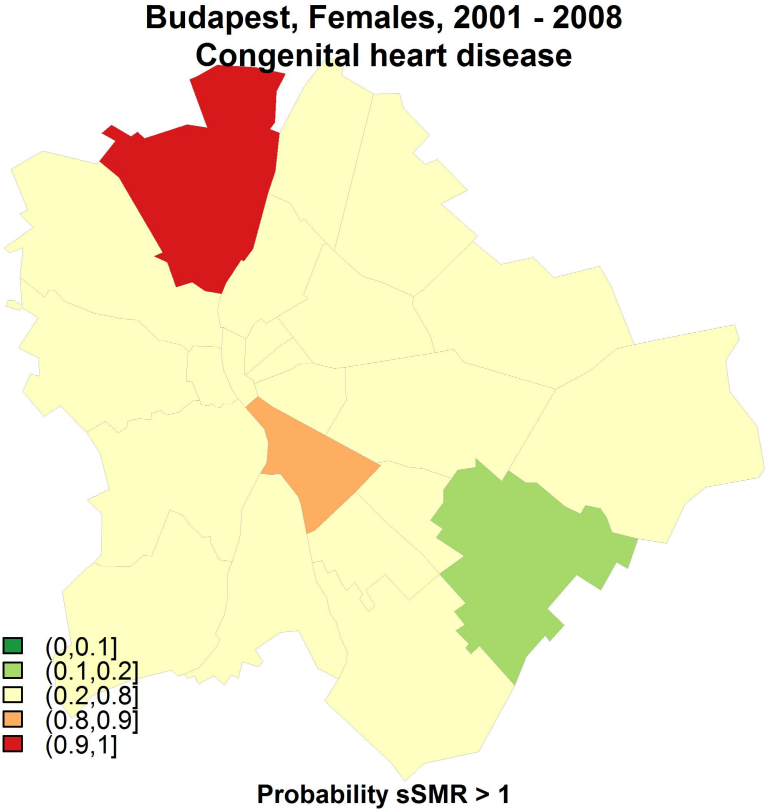
Budapest, Females, 2001 - 2008
Congenital heart disease
(0,0.1]
(0.1,0.2]
(0.2,0.8]
(0.8,0.9]
(0.9,1]
Probability sSMR > 1
