# Supplementary material for: Social differences in avoidable mortality between small areas of 15 European cities: an ecological study
Source: Int J Health Geogr. 2014 Mar 12;13:8. doi: 10.1186/1476-072X-13-8 (PMC4007807; doi:10.1186/1476-072X-13-8)
Supplement: Additional file 8 — Cause-specific mortality maps for Helsinki. [file 1476-072X-13-8-S8.pdf]

# Helsinki, Males, 2000 - 2009
# MN colon

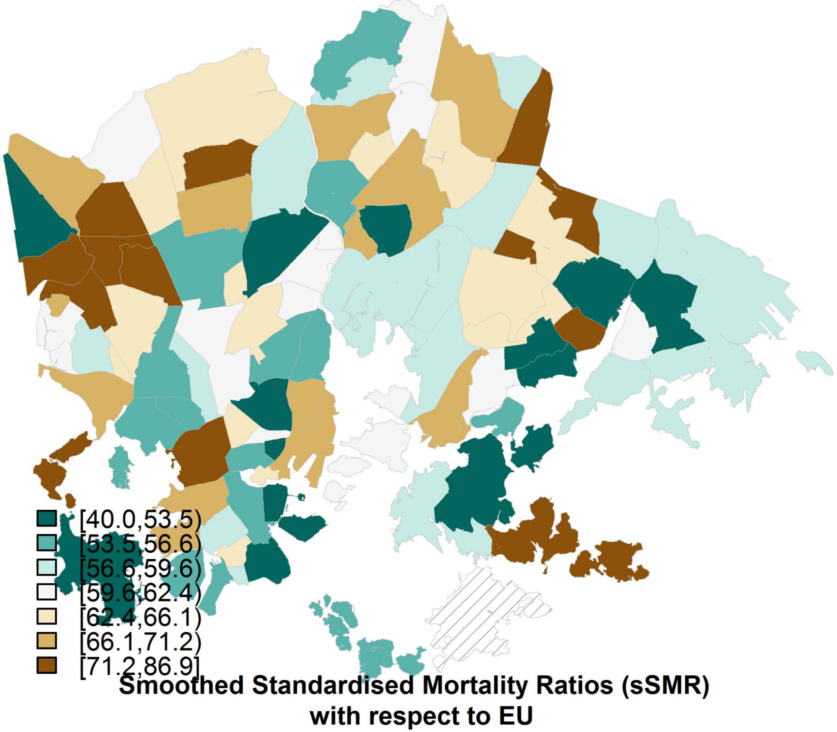


**Smoothed Standardised Mortality Ratios (sSMR)**
**with respect to EU**

# Helsinki, Males, 2000 - 2009
## MN colon

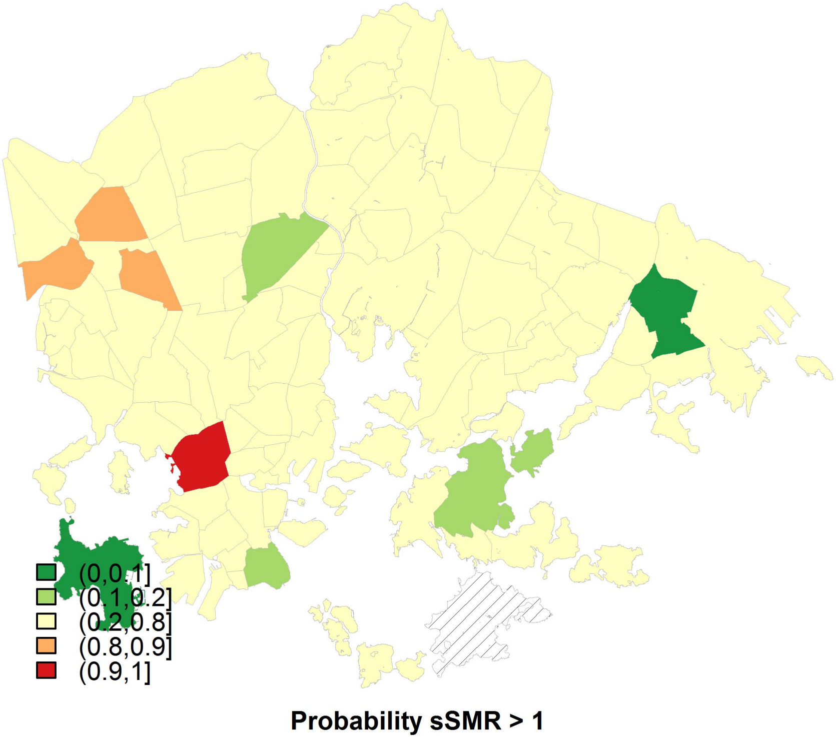

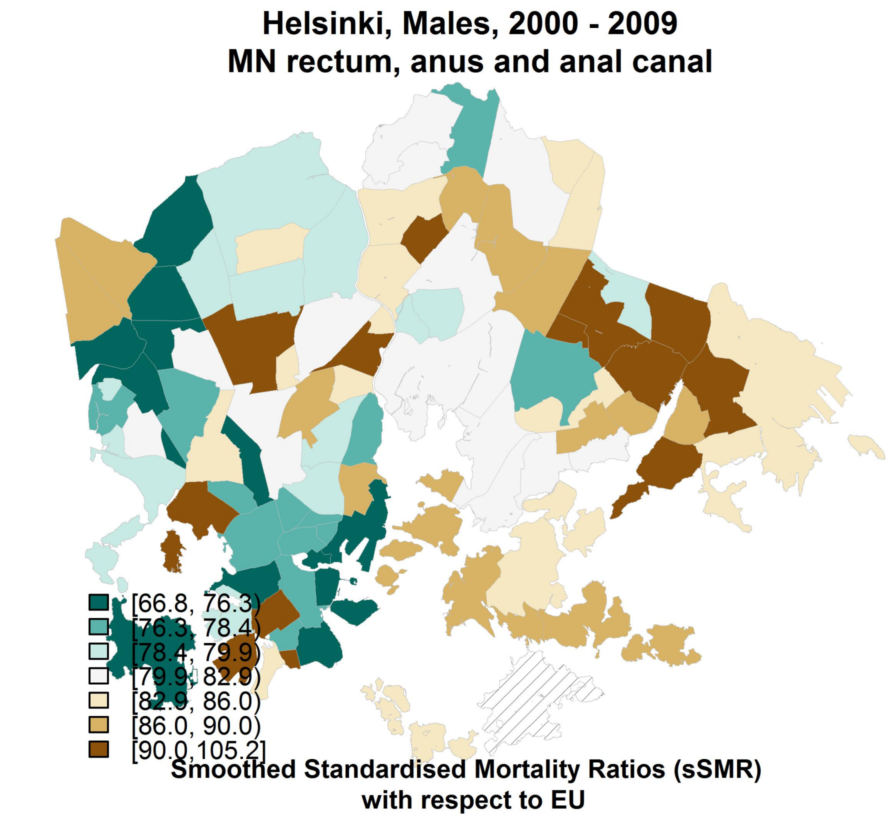
Helsinki, Males, 2000 - 2009
MN rectum, anus and anal canal
[66.8, 76.3)
[76.3, 78.4)
[78.4, 79.9)
[79.9, 82.9)
[82.9, 86.0)
[86.0, 90.0)
[90.0,105.2]
Smoothed Standardised Mortality Ratios (sSMR)
with respect to EU

## Helsinki, Males, 2000 - 2009
## MN rectum, anus and anal canal

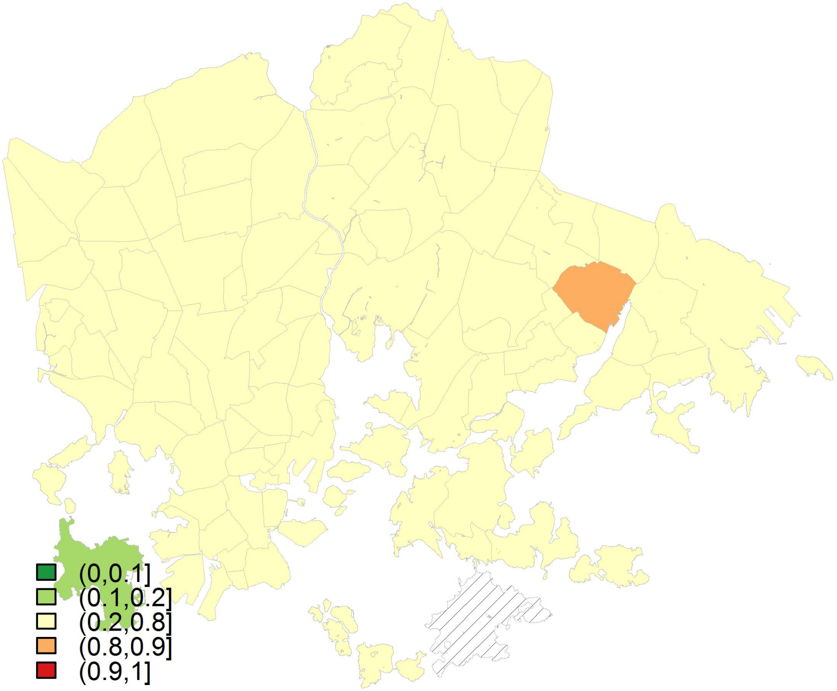


**Probability sSMR > 1**

# Helsinki, Males, 2000 - 2009
## Hypertension

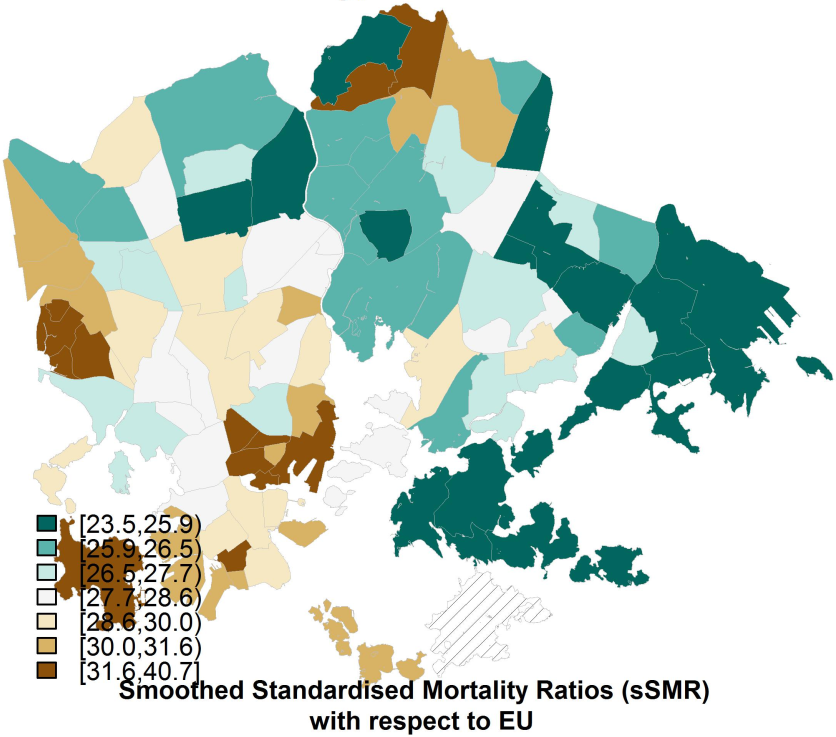


**Smoothed Standardised Mortality Ratios (sSMR)**
**with respect to EU**

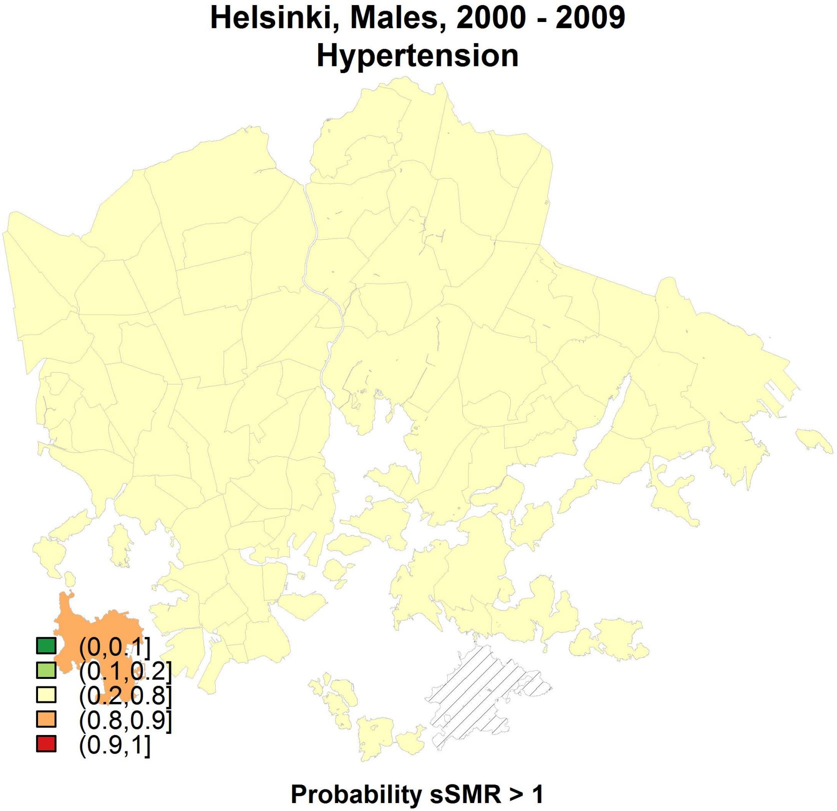
Helsinki, Males, 2000 - 2009
Hypertension
(0,0.1]
(0.1,0.2]
(0.2,0.8]
(0.8,0.9]
(0.9,1]
Probability sSMR > 1

# Helsinki, Males, 2000 - 2009
## Heart failure

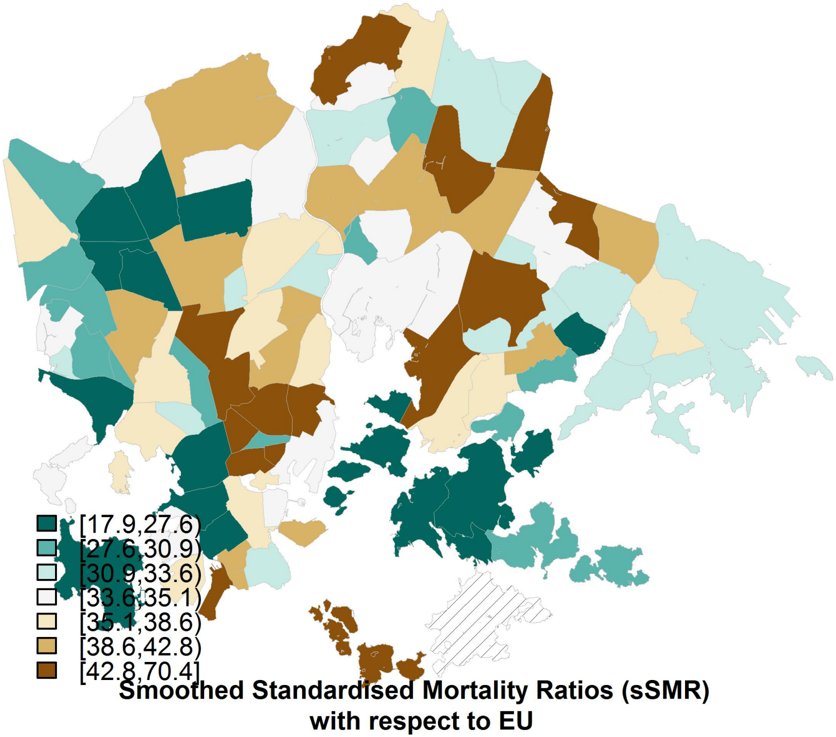


**Smoothed Standardised Mortality Ratios (sSMR)**
**with respect to EU**

## Helsinki, Males, 2000 - 2009
## Heart failure

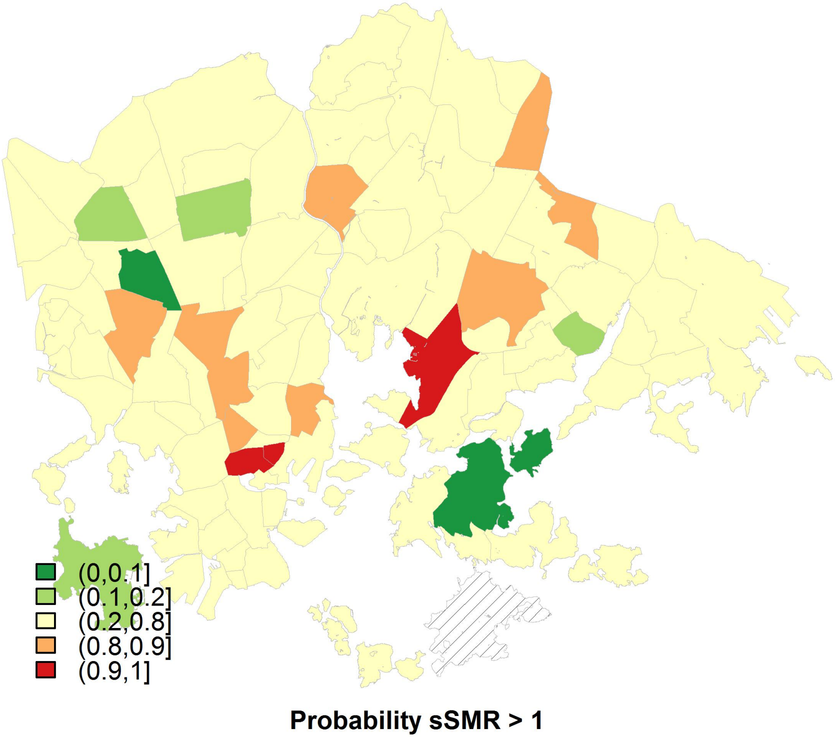

**Helsinki, Males, 2000 - 2009**
**Cerebrovascular diseases**

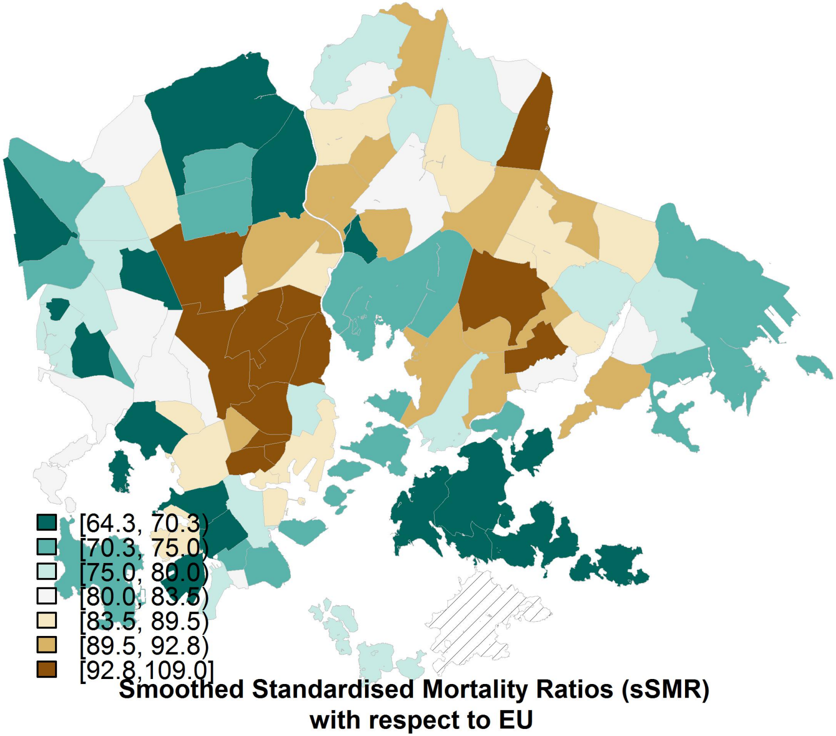


**Smoothed Standardised Mortality Ratios (sSMR)**
**with respect to EU**

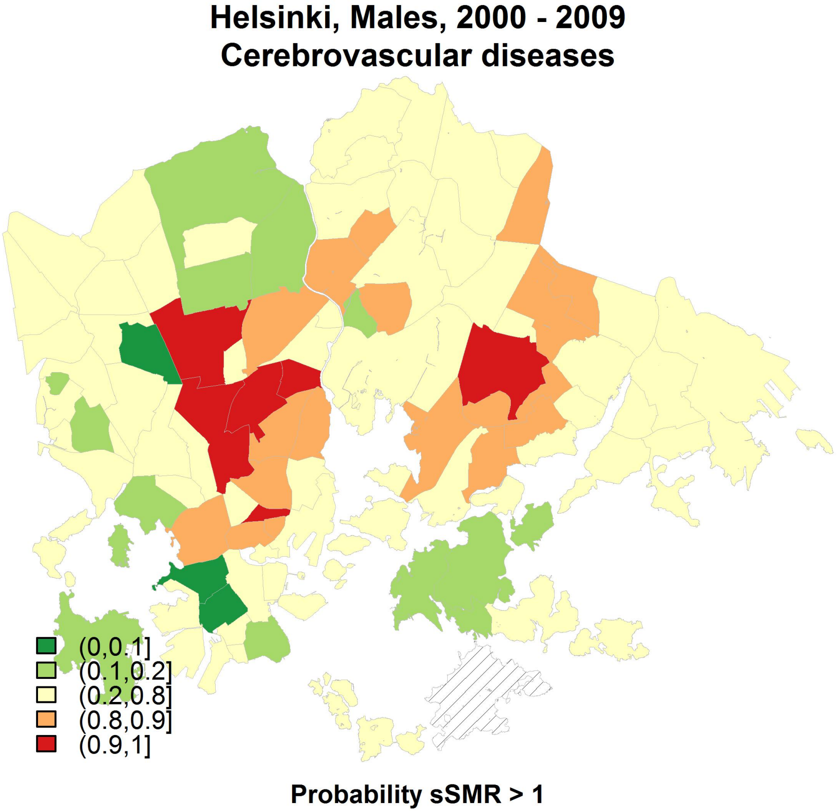
Helsinki, Males, 2000 - 2009
Cerebrovascular diseases
(0,0.1]
(0.1,0.2]
(0.2,0.8]
(0.8,0.9]
(0.9,1]
Probability sSMR > 1

## Helsinki, Males, 2000 - 2009
## Peptic ulcer

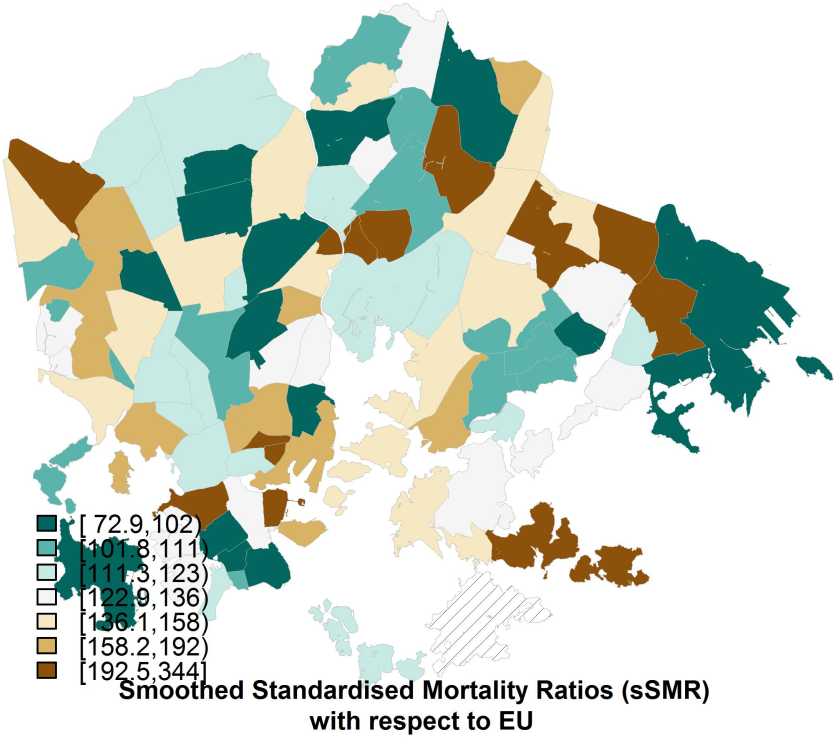


**Smoothed Standardised Mortality Ratios (sSMR)**
**with respect to EU**

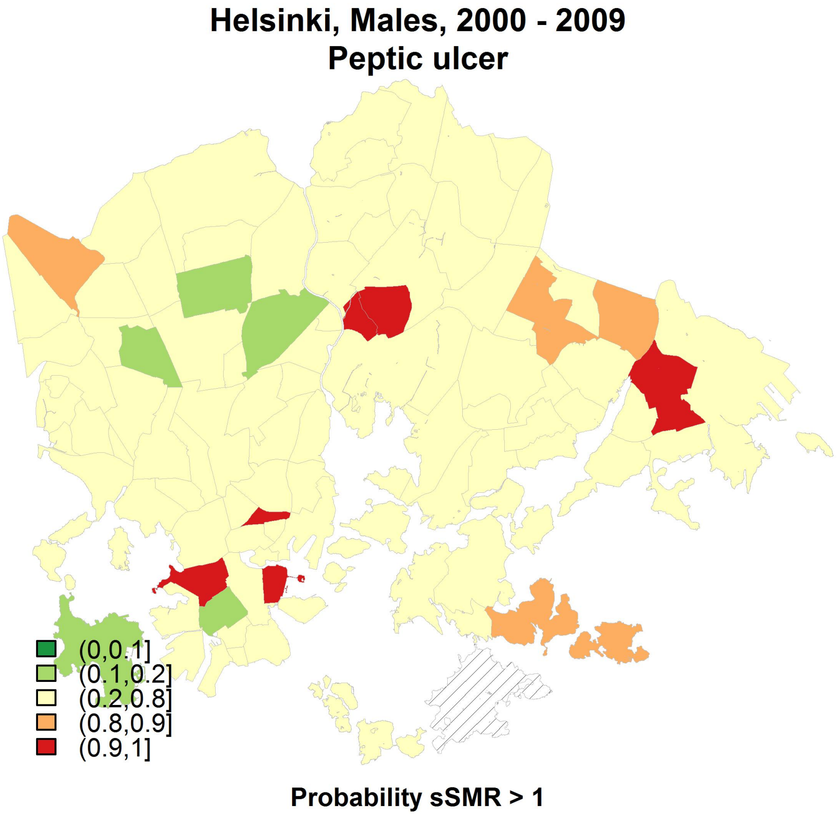

Helsinki, Males, 2000 - 2009
Peptic ulcer
(0,0.1]
(0.1,0.2]
(0.2,0.8]
(0.8,0.9]
(0.9,1]
Probability sSMR > 1

**Helsinki, Males, 2000 - 2009**
**Renal failure**

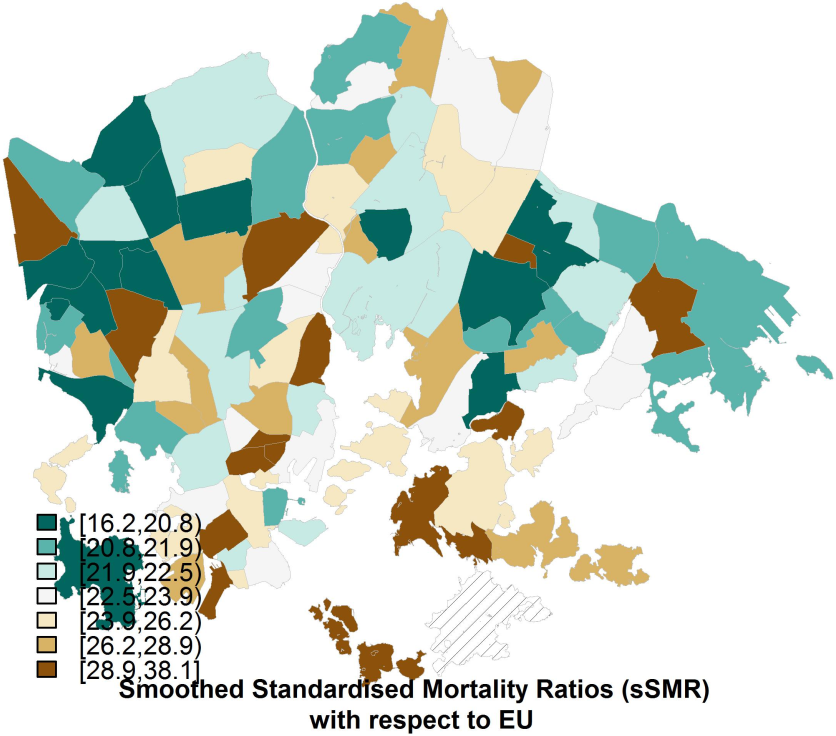


**Smoothed Standardised Mortality Ratios (sSMR)**
**with respect to EU**

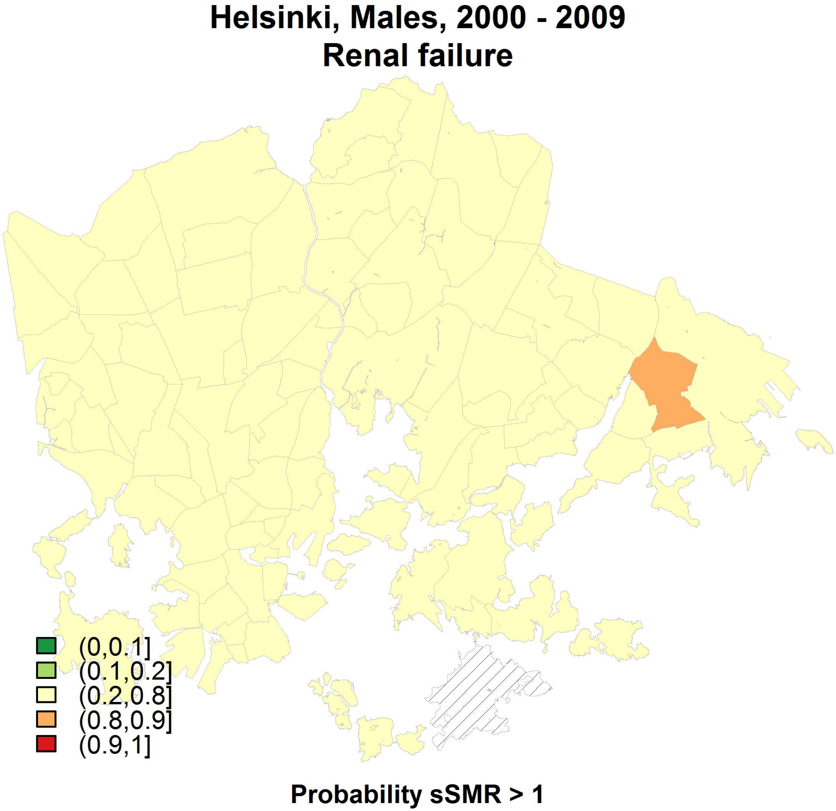
Helsinki, Males, 2000 - 2009
Renal failure
(0,0.1]
(0.1,0.2]
(0.2,0.8]
(0.8,0.9]
(0.9,1]
Probability sSMR > 1

# Helsinki, Females, 2000 - 2009
## MN colon

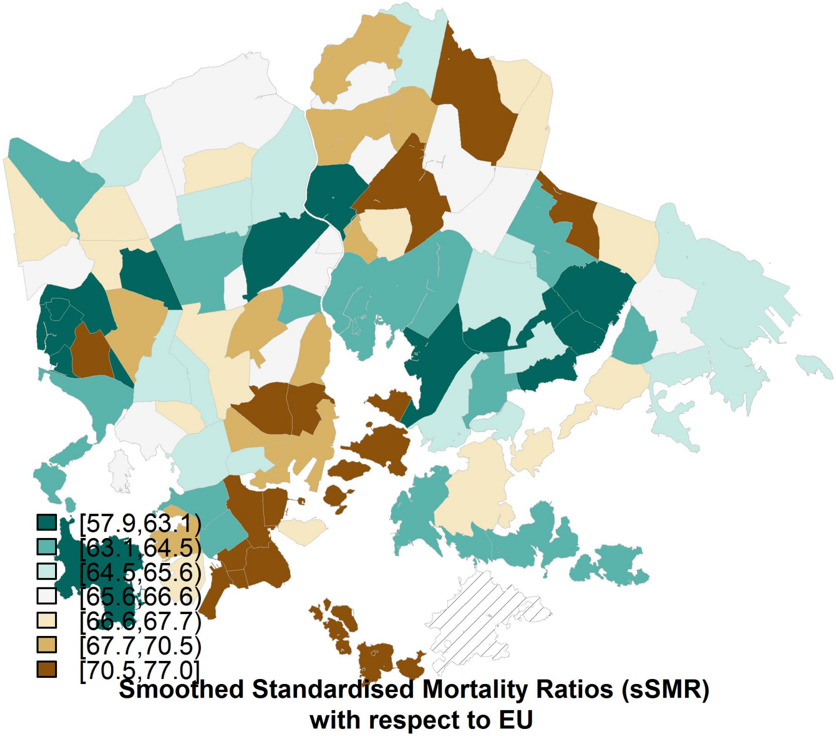


**Smoothed Standardised Mortality Ratios (sSMR)
with respect to EU**

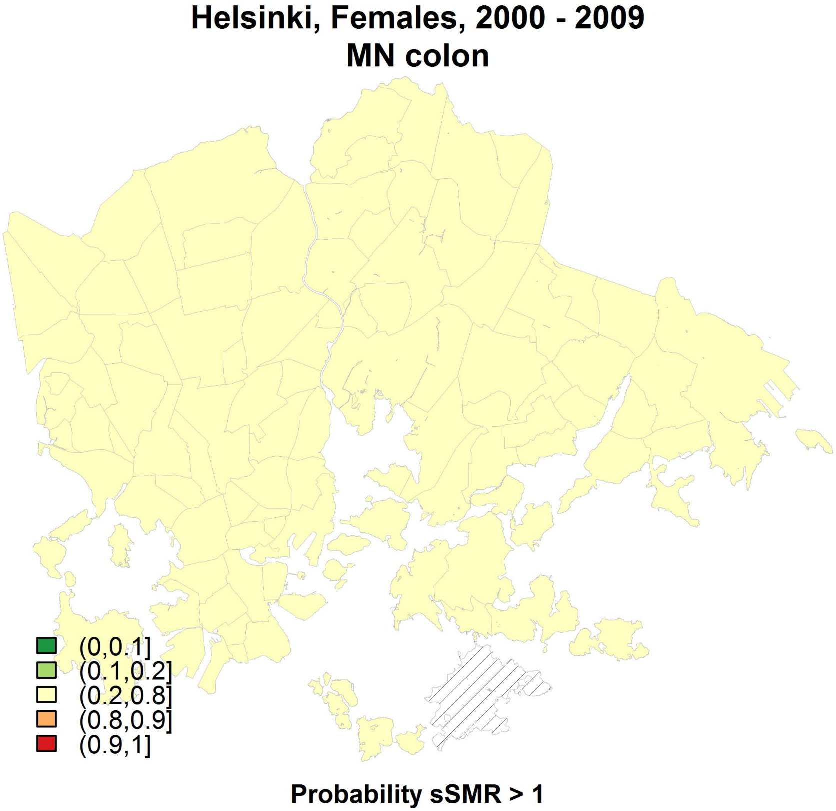
Helsinki, Females, 2000 - 2009
MN colon
(0,0.1]
(0.1,0.2]
(0.2,0.8]
(0.8,0.9]
(0.9,1]
Probability sSMR > 1

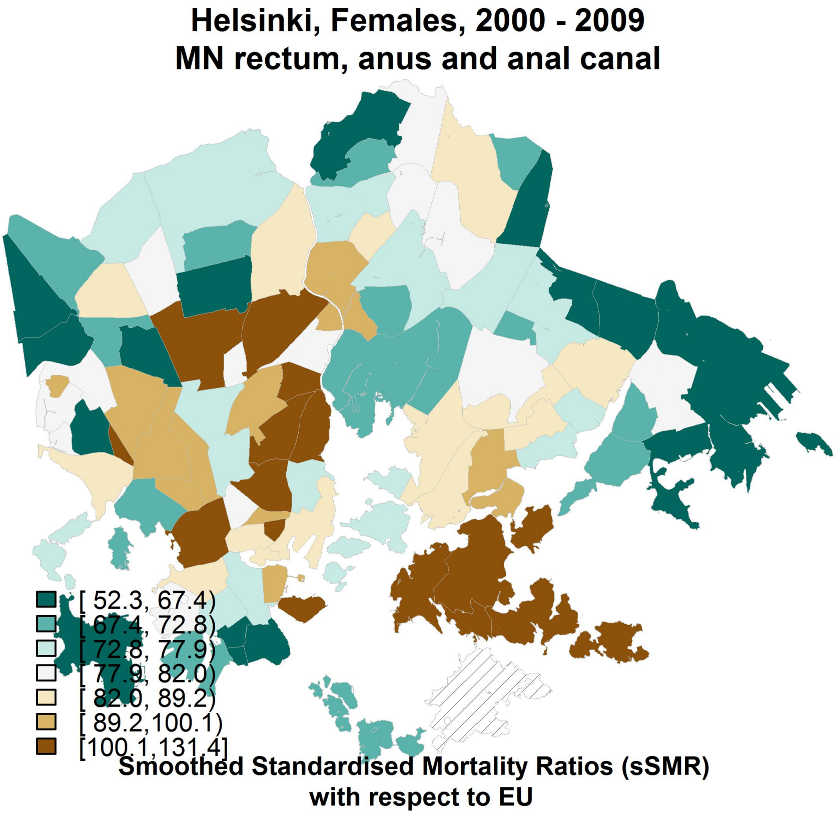
Helsinki, Females, 2000 - 2009
MN rectum, anus and anal canal
[ 52.3, 67.4)
[ 67.4, 72.8)
[ 72.8, 77.9)
[ 77.9, 82.0)
[ 82.0, 89.2)
[ 89.2,100.1)
[100.1,131.4]
Smoothed Standardised Mortality Ratios (sSMR)
with respect to EU

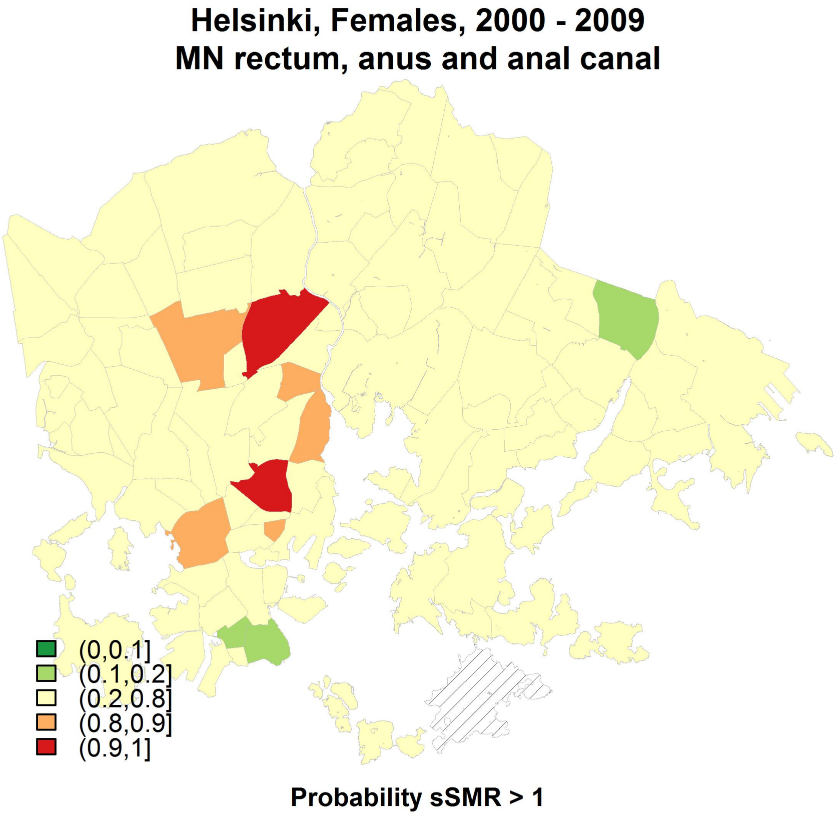
Helsinki, Females, 2000 - 2009
MN rectum, anus and anal canal
(0,0.1]
(0.1,0.2]
(0.2,0.8]
(0.8,0.9]
(0.9,1]
Probability sSMR > 1

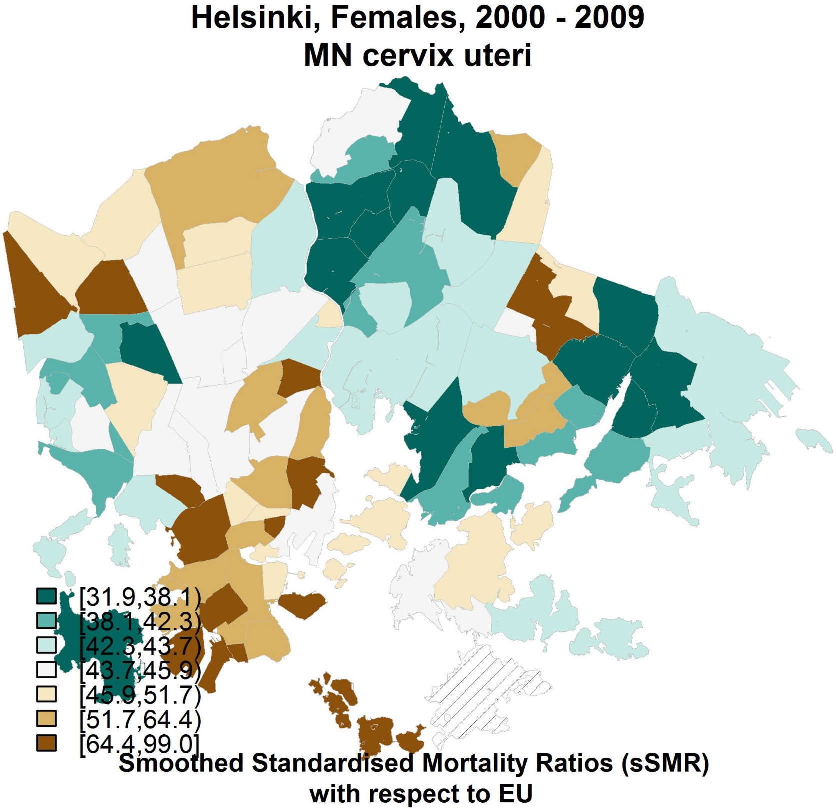

Helsinki, Females, 2000 - 2009
MN cervix uteri
[31.9,38.1)
[38.1,42.3)
[42.3,43.7)
[43.7,45.9)
[45.9,51.7)
[51.7,64.4)
[64.4,99.0]
Smoothed Standardised Mortality Ratios (sSMR)
with respect to EU

# Helsinki, Females, 2000 - 2009
# MN cervix uteri

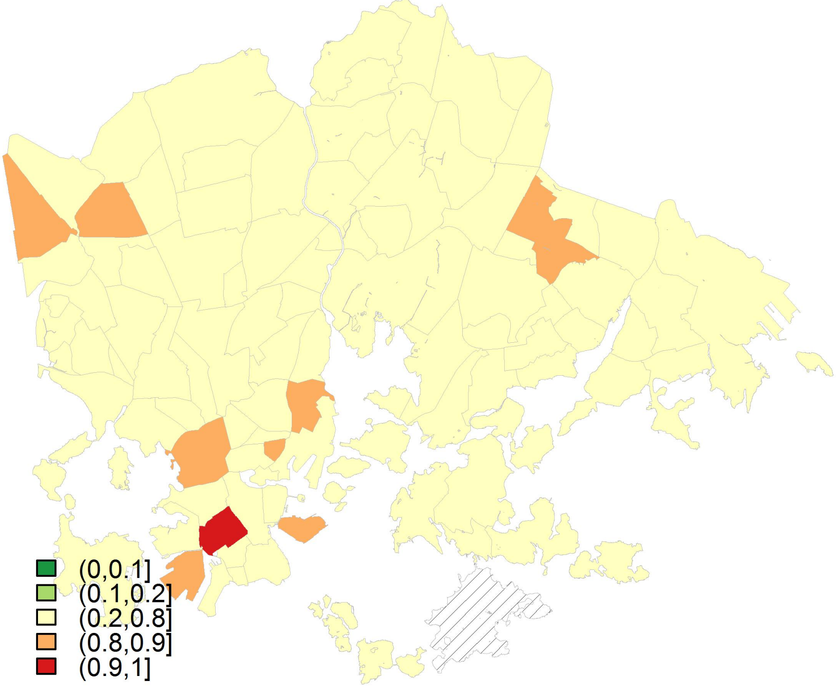


**Probability sSMR > 1**

## Helsinki, Females, 2000 - 2009
## Rheumatic heart disease

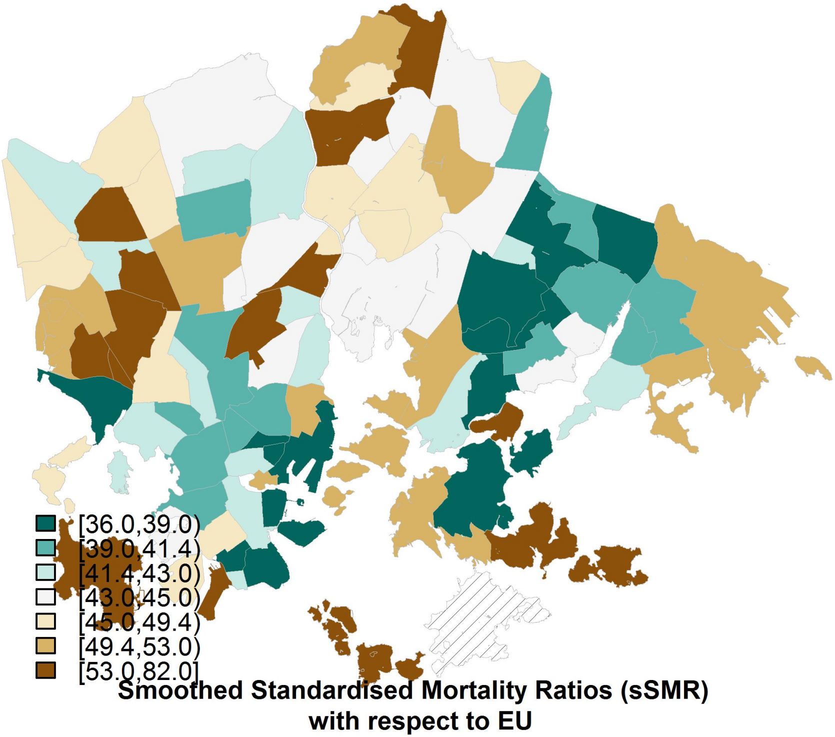


**Smoothed Standardised Mortality Ratios (sSMR)**
**with respect to EU**

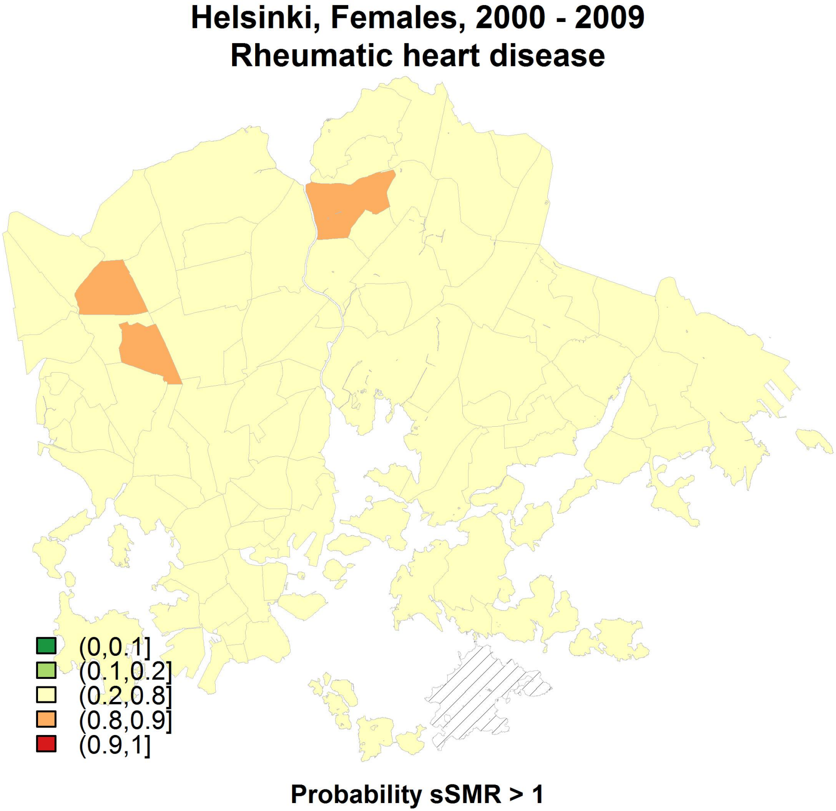
Helsinki, Females, 2000 - 2009
Rheumatic heart disease
(0,0.1]
(0.1,0.2]
(0.2,0.8]
(0.8,0.9]
(0.9,1]
Probability sSMR > 1

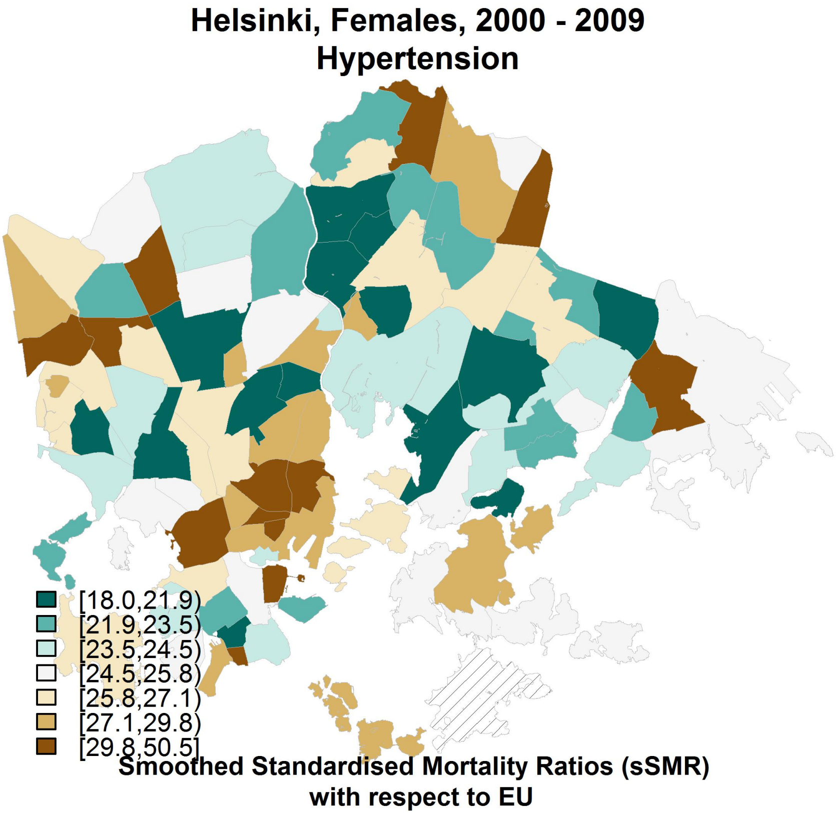

Helsinki, Females, 2000 - 2009
Hypertension
[18.0,21.9)
[21.9,23.5)
[23.5,24.5)
[24.5,25.8)
[25.8,27.1)
[27.1,29.8)
[29.8,50.5]
Smoothed Standardised Mortality Ratios (sSMR)
with respect to EU

# Helsinki, Females, 2000 - 2009
# Hypertension

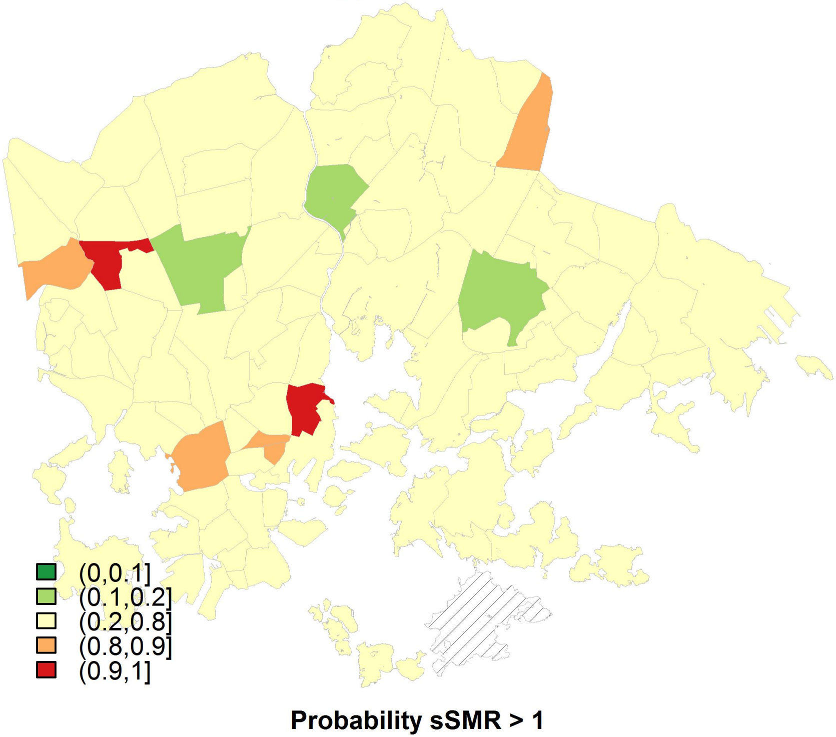

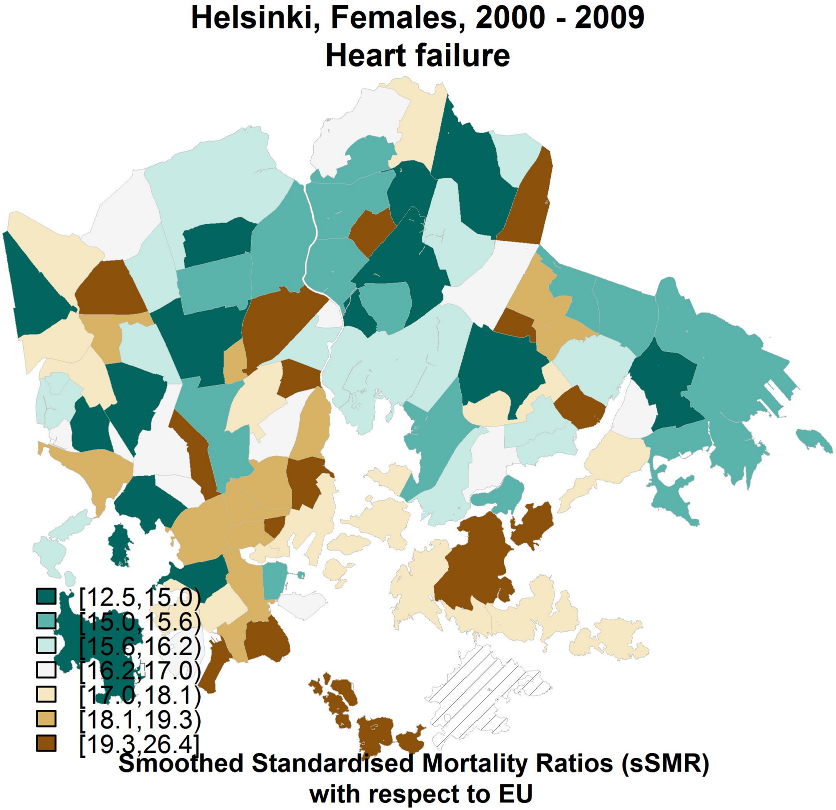
Helsinki, Females, 2000 - 2009
Heart failure
[12.5,15.0)
[15.0,15.6)
[15.6,16.2)
[16.2,17.0)
[17.0,18.1)
[18.1,19.3)
[19.3,26.4]
Smoothed Standardised Mortality Ratios (sSMR)
with respect to EU

## Helsinki, Females, 2000 - 2009
## Heart failure

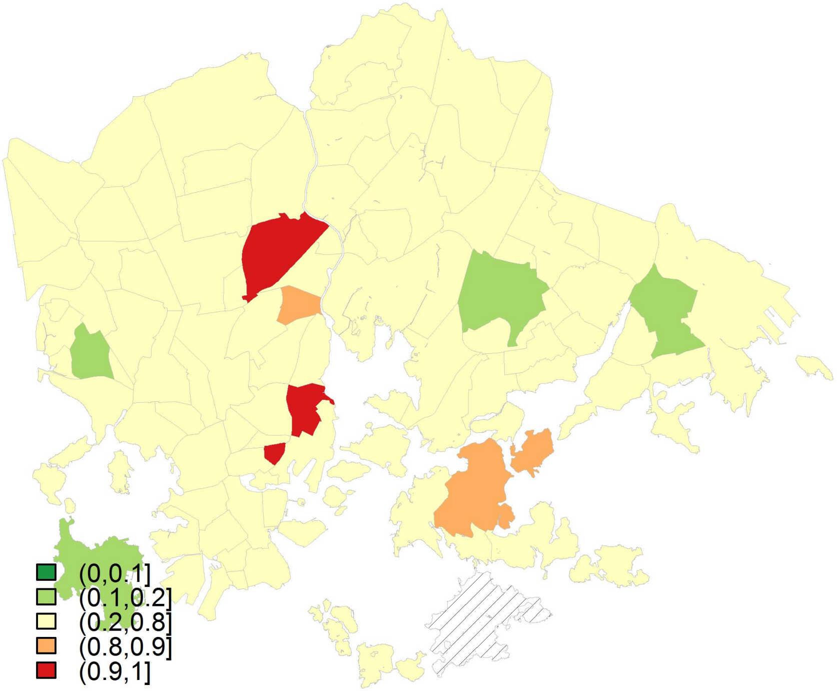

## Helsinki, Females, 2000 - 2009
## Cerebrovascular diseases

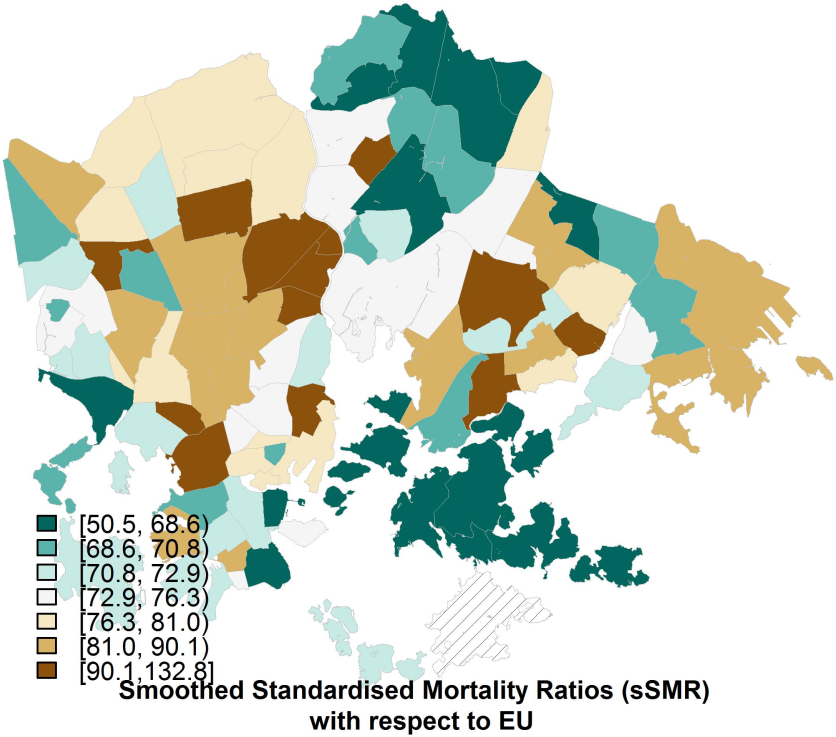


**Smoothed Standardised Mortality Ratios (sSMR) with respect to EU**

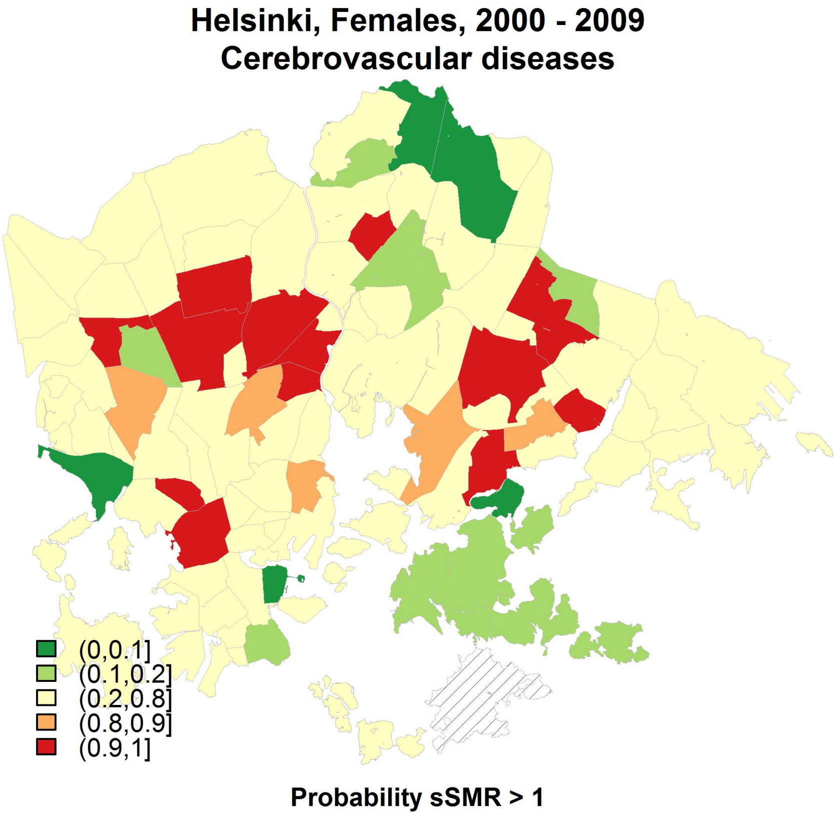

Helsinki, Females, 2000 - 2009
Cerebrovascular diseases
(0,0.1]
(0.1,0.2]
(0.2,0.8]
(0.8,0.9]
(0.9,1]
Probability sSMR > 1

## Helsinki, Females, 2000 - 2009
## Peptic ulcer

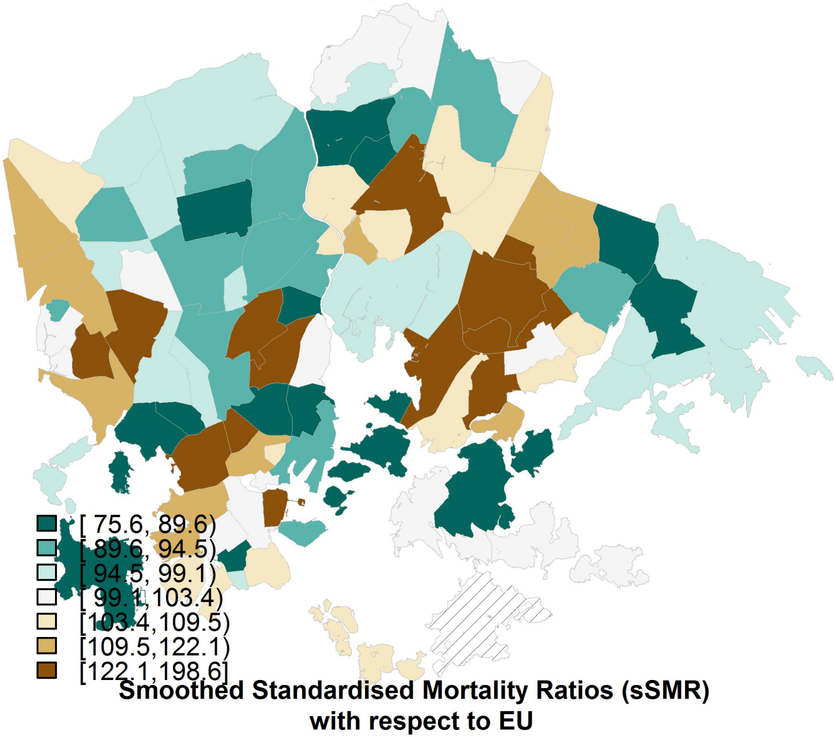


**Smoothed Standardised Mortality Ratios (sSMR)
with respect to EU**

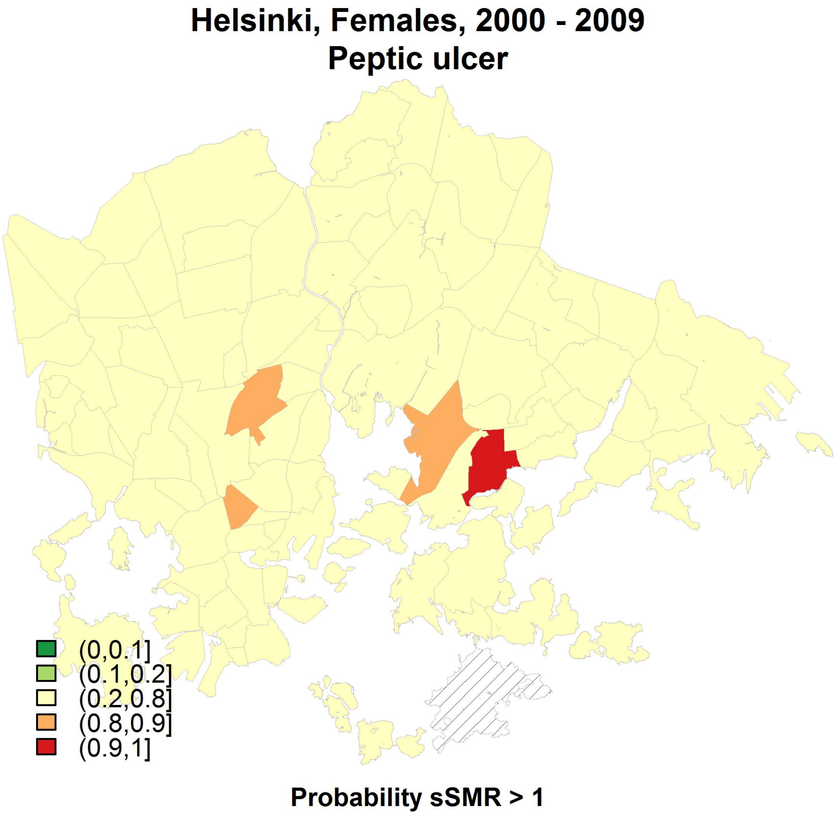
Helsinki, Females, 2000 - 2009
Peptic ulcer
(0,0.1]
(0.1,0.2]
(0.2,0.8]
(0.8,0.9]
(0.9,1]
Probability sSMR > 1

**Helsinki, Females, 2000 - 2009**
**Renal failure**

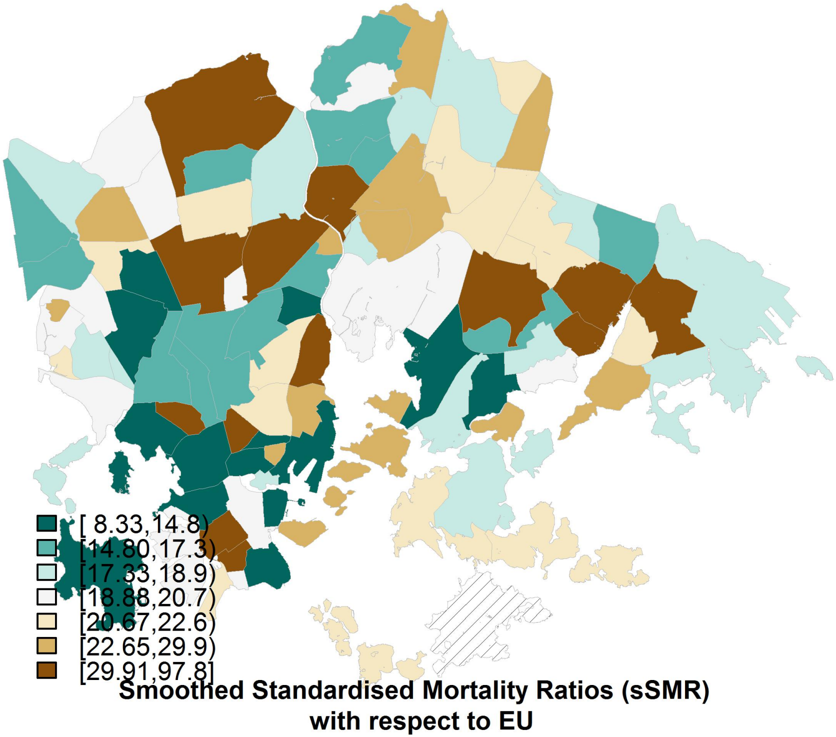


**Smoothed Standardised Mortality Ratios (sSMR)**
**with respect to EU**

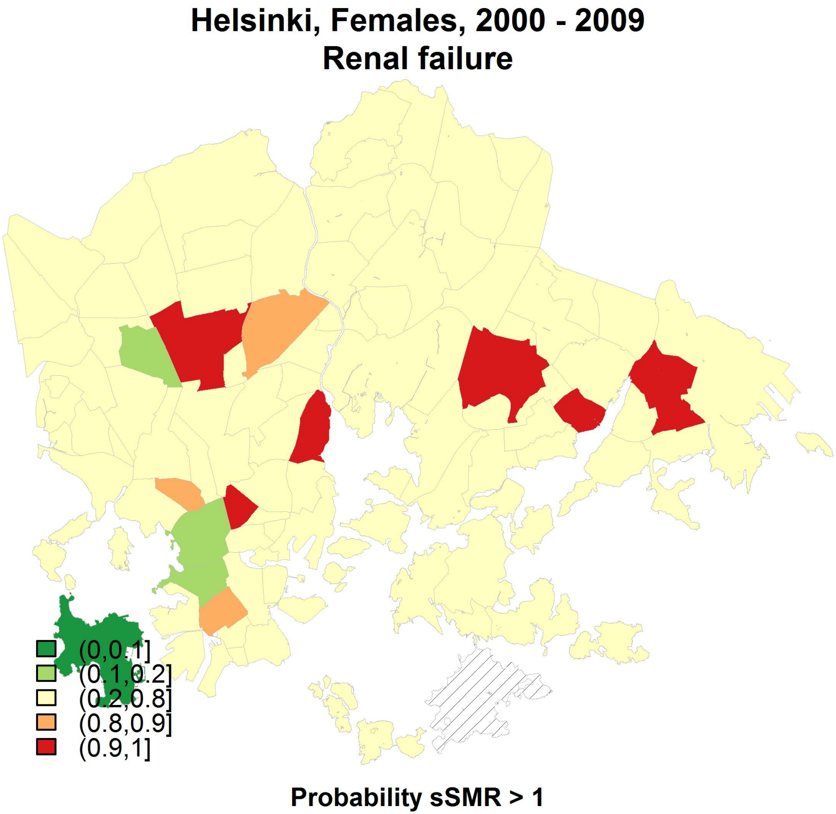
Helsinki, Females, 2000 - 2009
Renal failure
(0,0.1]
(0.1,0.2]
(0.2,0.8]
(0.8,0.9]
(0.9,1]
Probability sSMR > 1
