# Supplementary material for: Social differences in avoidable mortality between small areas of 15 European cities: an ecological study
Source: Int J Health Geogr. 2014 Mar 12;13:8. doi: 10.1186/1476-072X-13-8 (PMC4007807; doi:10.1186/1476-072X-13-8)

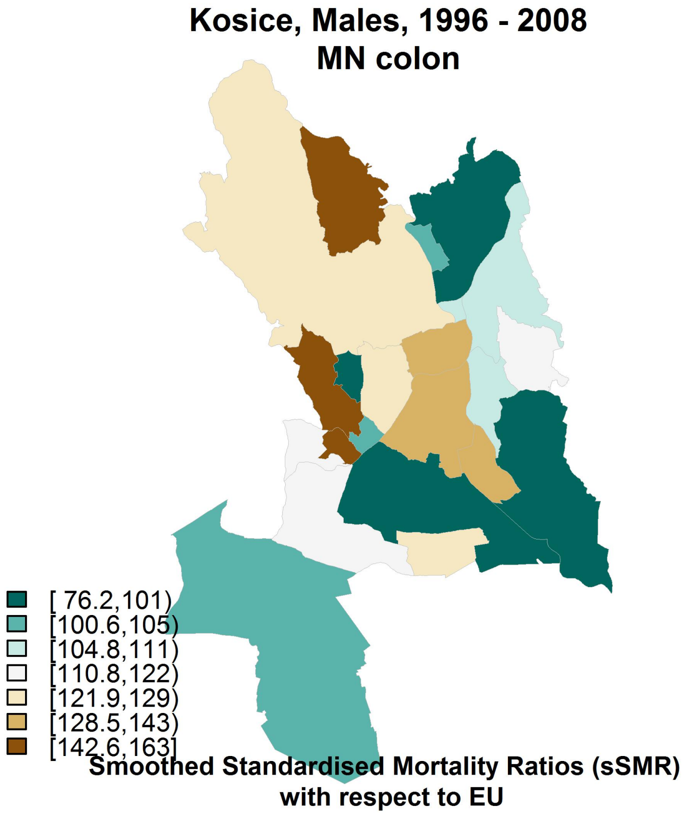
Kosice, Males, 1996 - 2008
MN colon
[76.2,101)
[100.6,105)
[104.8,111)
[110.8,122)
[121.9,129)
[128.5,143)
[142.6,163]
Smoothed Standardised Mortality Ratios (sSMR)
with respect to EU

Kosice, Males, 1996 - 2008
MN colon

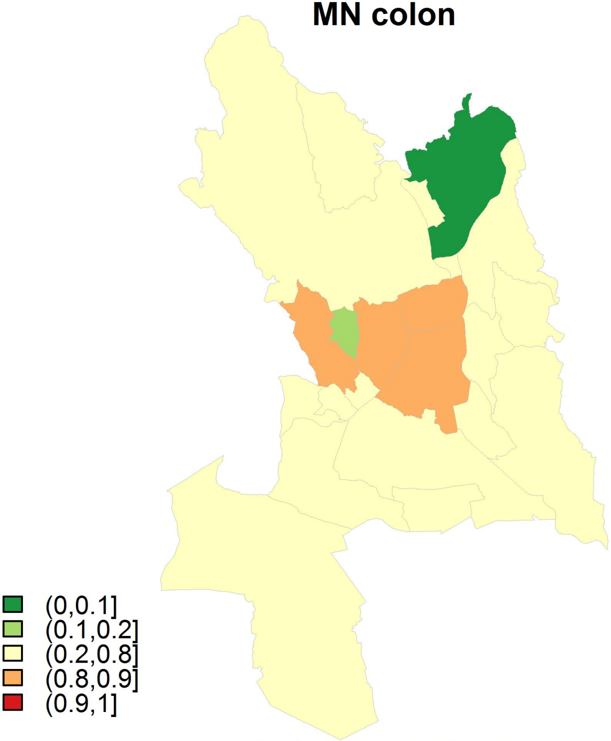

Probability sSMR > 1

Kosice, Males, 1996 - 2008
MN rectum, anus and anal canal

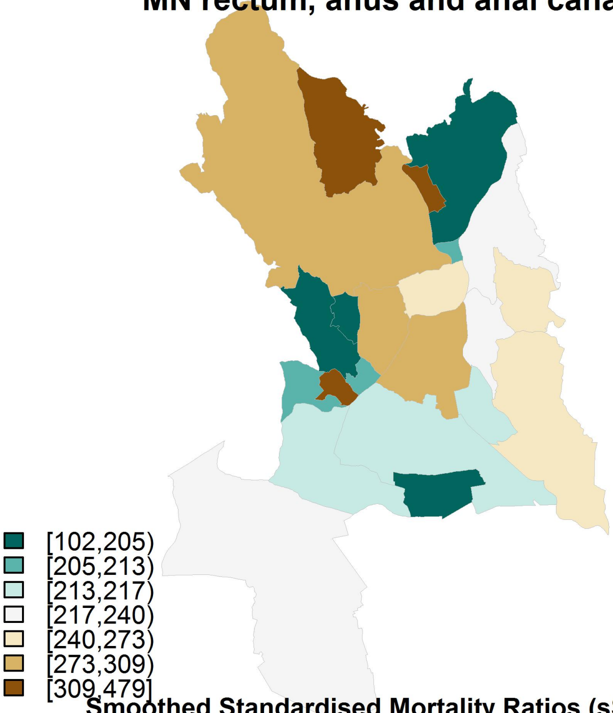

Smoothed Standardised Mortality Ratios (sSMR)
with respect to EU

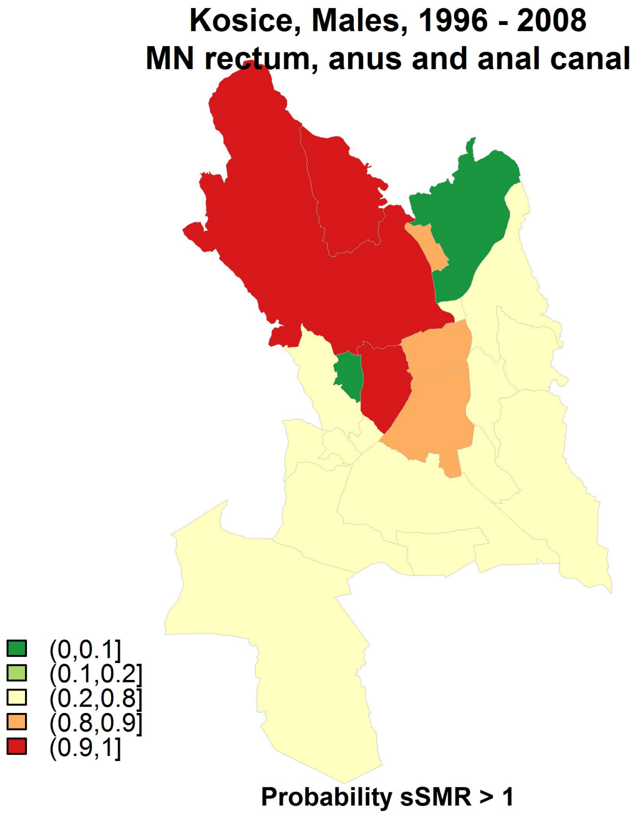
Kosice, Males, 1996 - 2008
MN rectum, anus and anal canal
(0,0.1]
(0.1,0.2]
(0.2,0.8]
(0.8,0.9]
(0.9,1]
Probability sSMR > 1

Kosice, Males, 1996 - 2008
MN testes

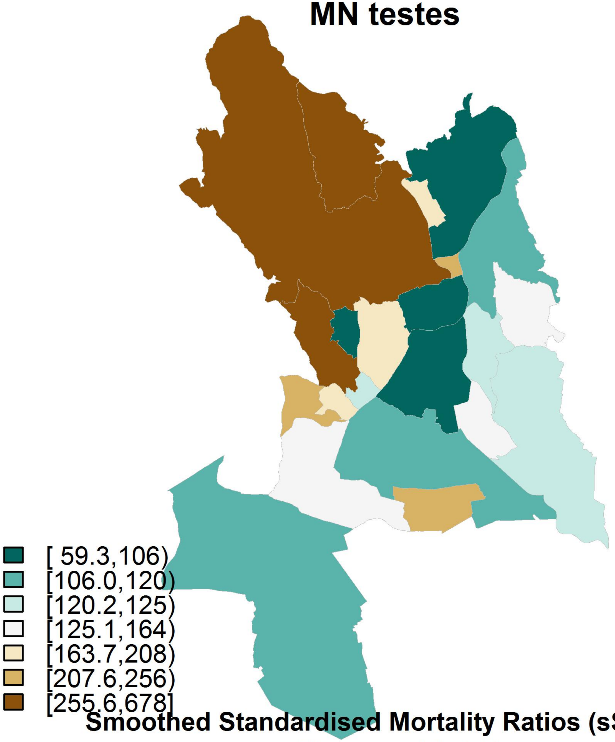

Smoothed Standardised Mortality Ratios (sSMR)
with respect to EU

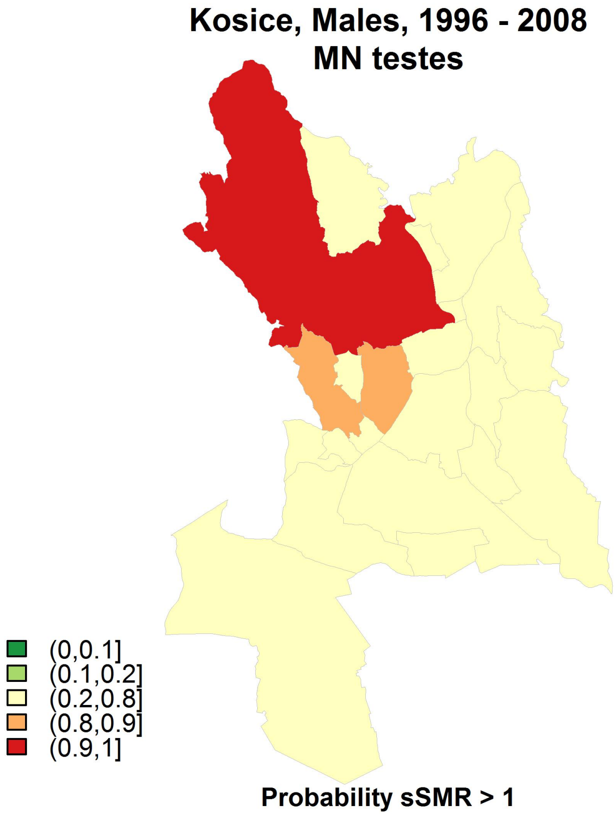
Kosice, Males, 1996 - 2008
MN testes
(0,0.1]
(0.1,0.2]
(0.2,0.8]
(0.8,0.9]
(0.9,1]
Probability sSMR > 1

Kosice, Males, 1996 - 2008
Hodgkin’s disease

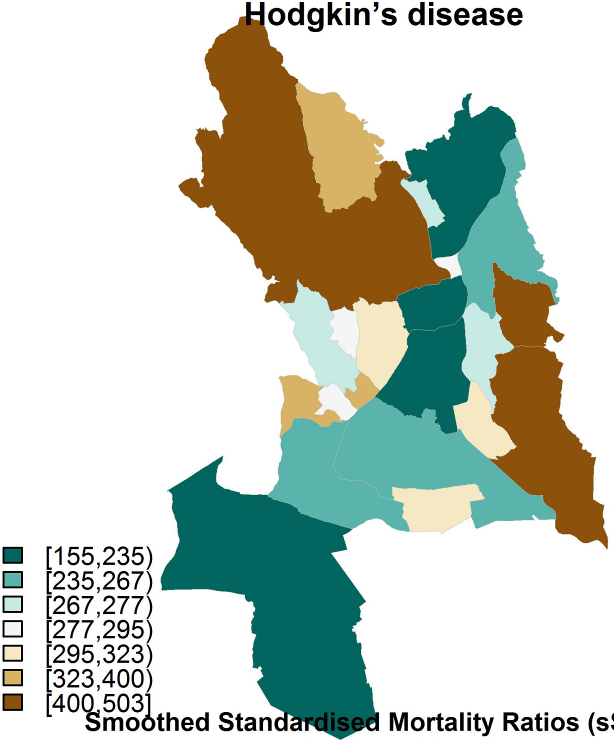

Smoothed Standardised Mortality Ratios (sSMR) with respect to EU

Kosice, Males, 1996 - 2008
Hodgkin's disease

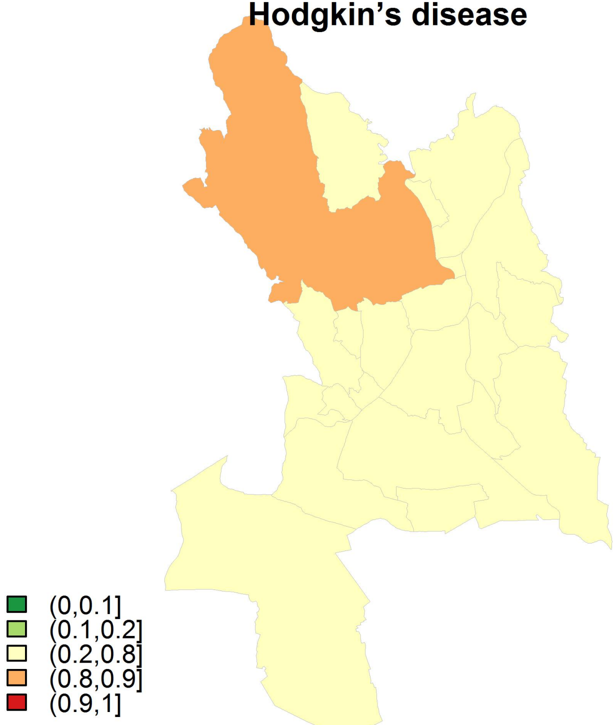

Probability sSMR > 1

Kosice, Males, 1996 - 2008
Rheumatic heart disease

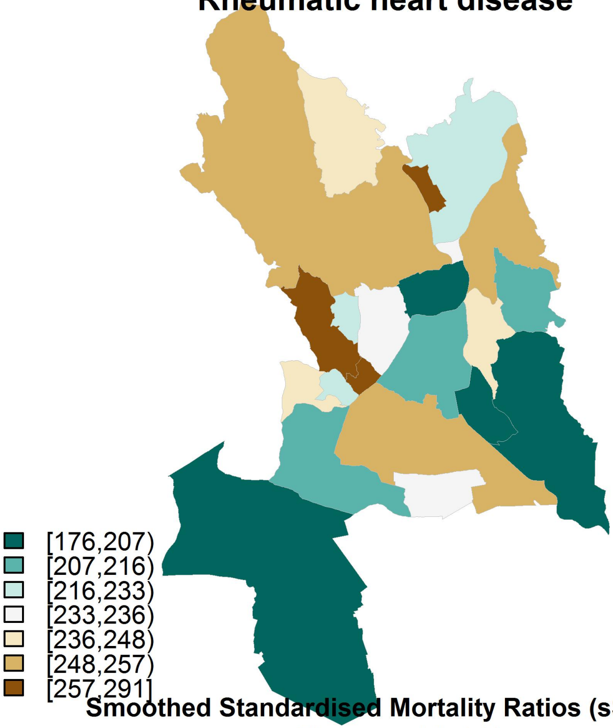

Smoothed Standardised Mortality Ratios (sSMR) with respect to EU

Kosice, Males, 1996 - 2008
Rheumatic heart disease

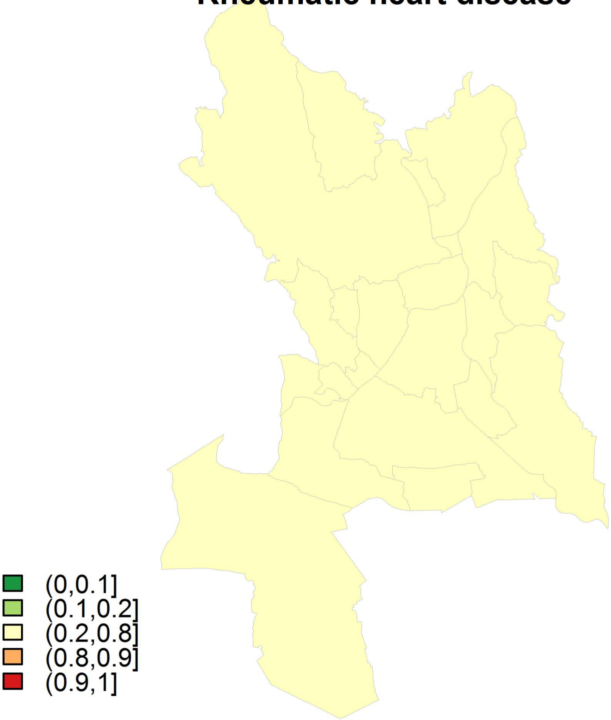

Probability sSMR > 1

Kosice, Males, 1996 - 2008
Hypertension

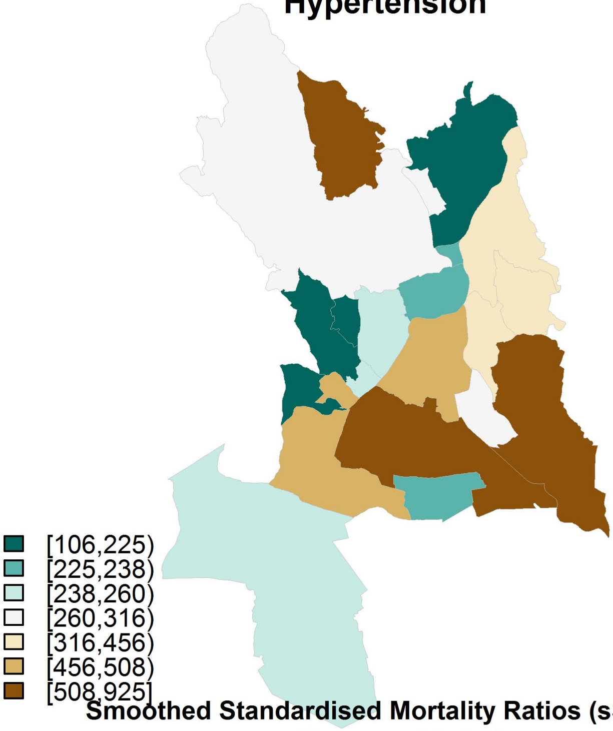

Smoothed Standardised Mortality Ratios (sSMR)
with respect to EU

Kosice, Males, 1996 - 2008
Hypertension

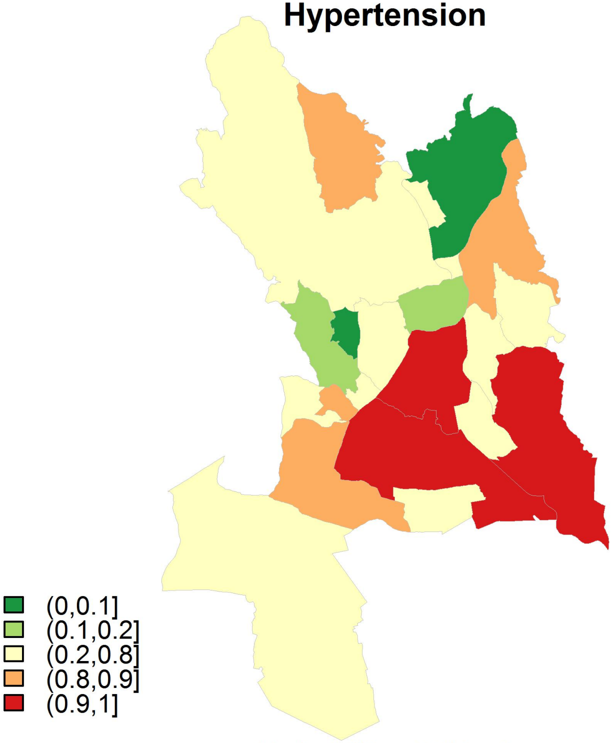

Probability sSMR > 1

Kosice, Males, 1996 - 2008
Heart failure

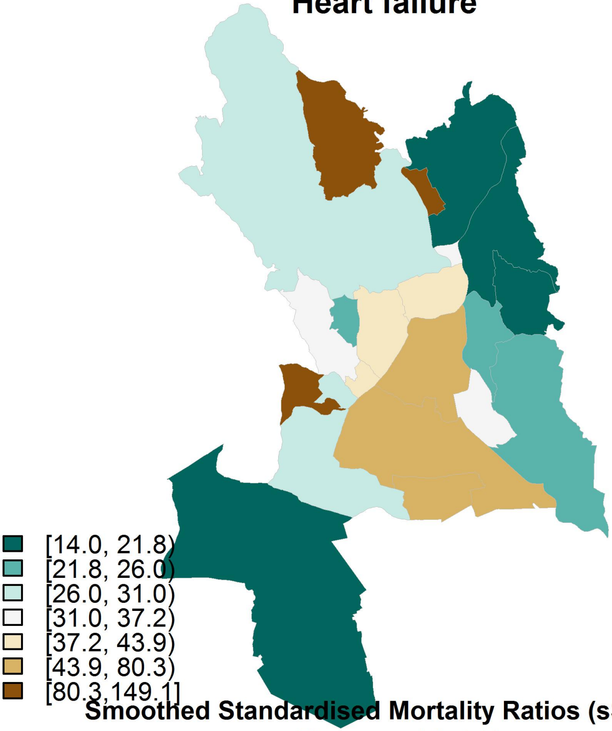

Smoothed Standardised Mortality Ratios (sSMR) with respect to EU

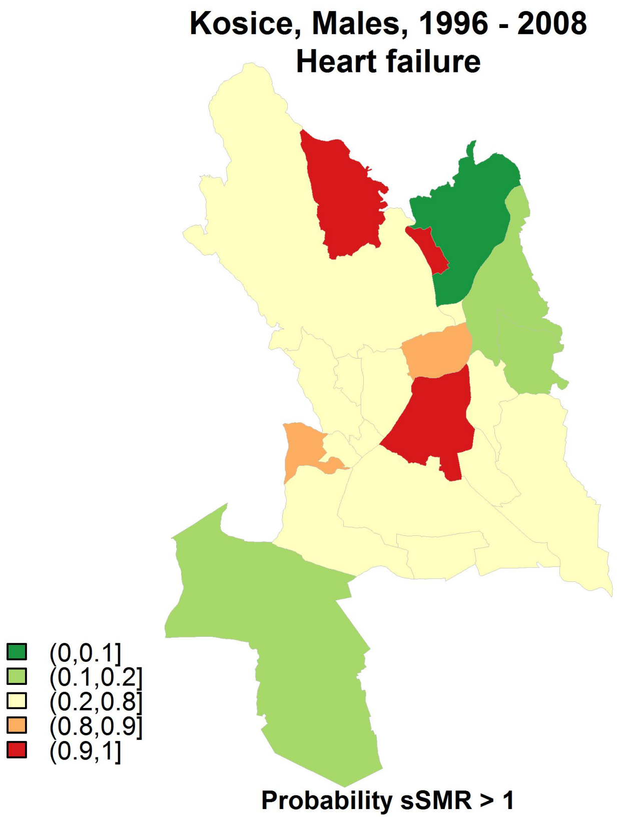

Kosice, Males, 1996 - 2008
Heart failure
(0,0.1]
(0.1,0.2]
(0.2,0.8]
(0.8,0.9]
(0.9,1]
Probability sSMR > 1

Kosice, Males, 1996 - 2008
Cerebrovascular diseases

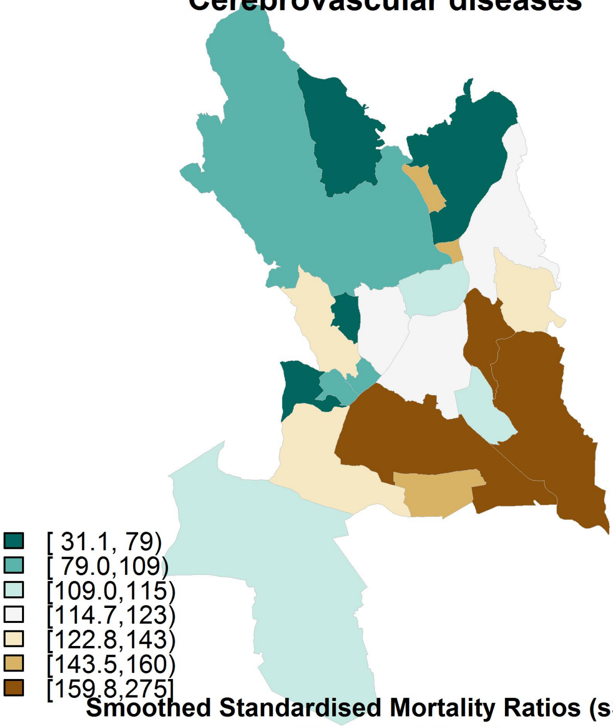

Smoothed Standardised Mortality Ratios (sSMR)
with respect to EU

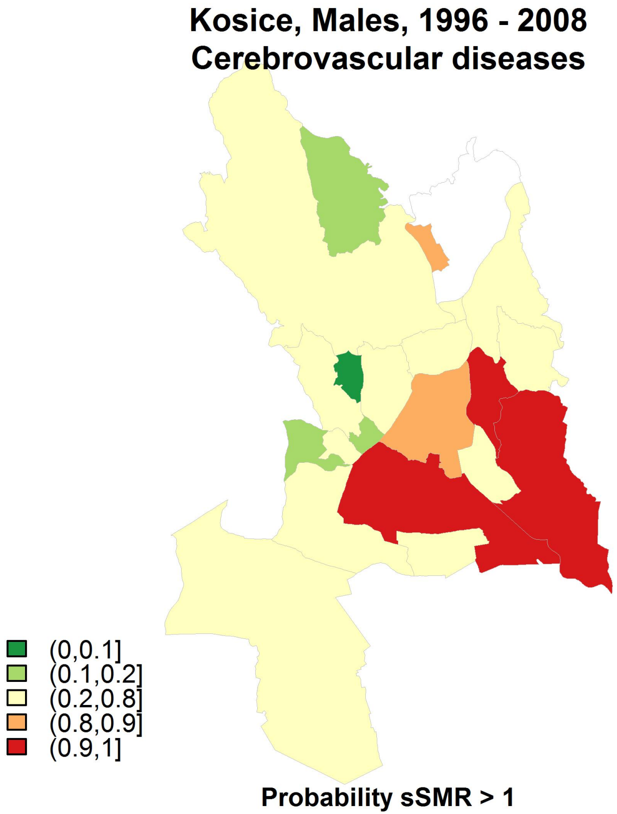
Kosice, Males, 1996 - 2008
Cerebrovascular diseases
(0,0.1]
(0.1,0.2]
(0.2,0.8]
(0.8,0.9]
(0.9,1]
Probability sSMR > 1

Kosice, Males, 1996 - 2008
Peptic ulcer

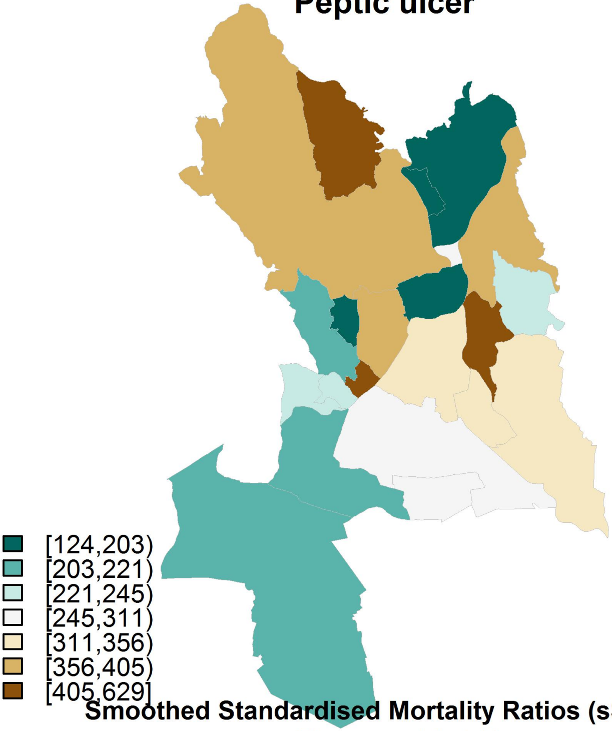

Smoothed Standardised Mortality Ratios (sSMR)
with respect to EU

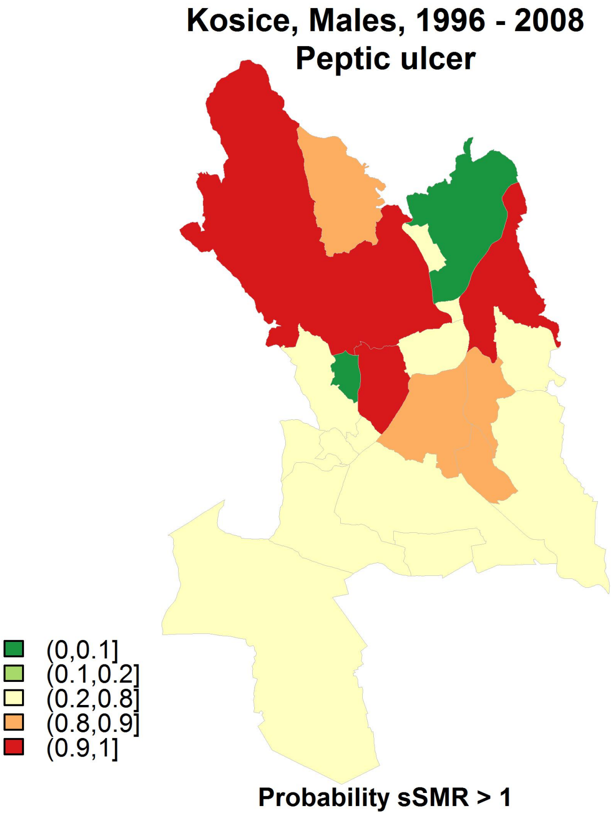
Kosice, Males, 1996 - 2008
Peptic ulcer
(0,0.1]
(0.1,0.2]
(0.2,0.8]
(0.8,0.9]
(0.9,1]
Probability sSMR > 1

Kosice, Males, 1996 - 2008
Renal failure

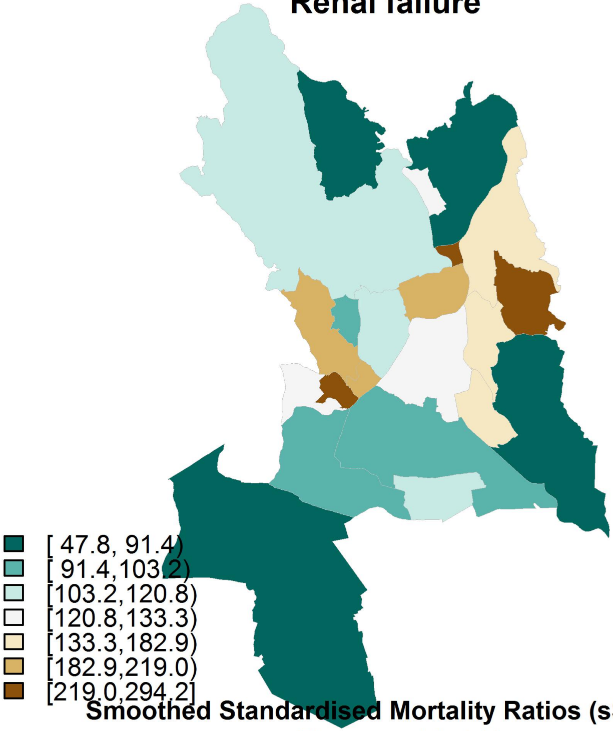

Smoothed Standardised Mortality Ratios (sSMR) with respect to EU

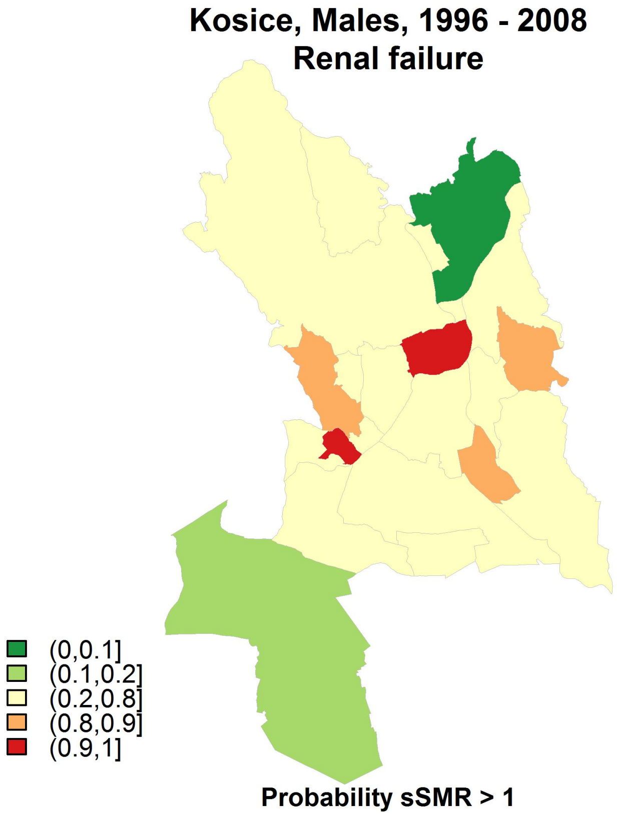
Kosice, Males, 1996 - 2008
Renal failure
(0,0.1]
(0.1,0.2]
(0.2,0.8]
(0.8,0.9]
(0.9,1]
Probability sSMR > 1

Kosice, Males, 1996 - 2008
Conditions originating in the perinatal period

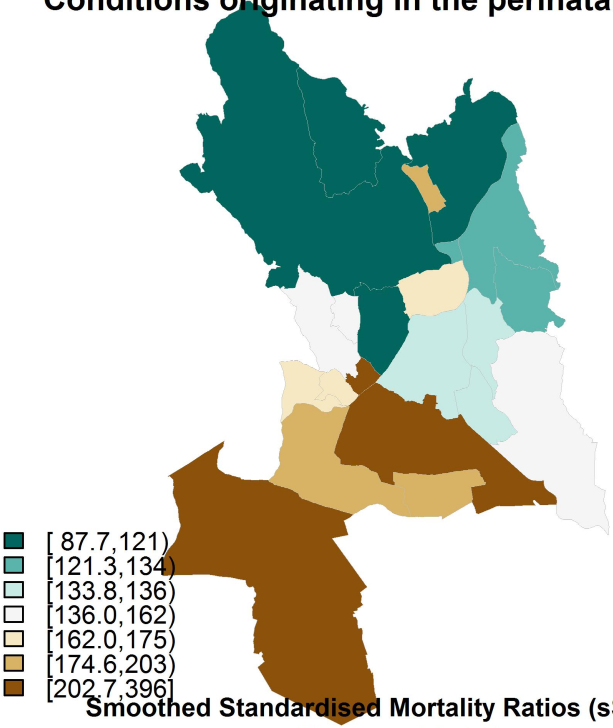

Smoothed Standardised Mortality Ratios (sSMR)
with respect to EU

Kosice, Males, 1996 - 2008
Conditions originating in the perinatal period

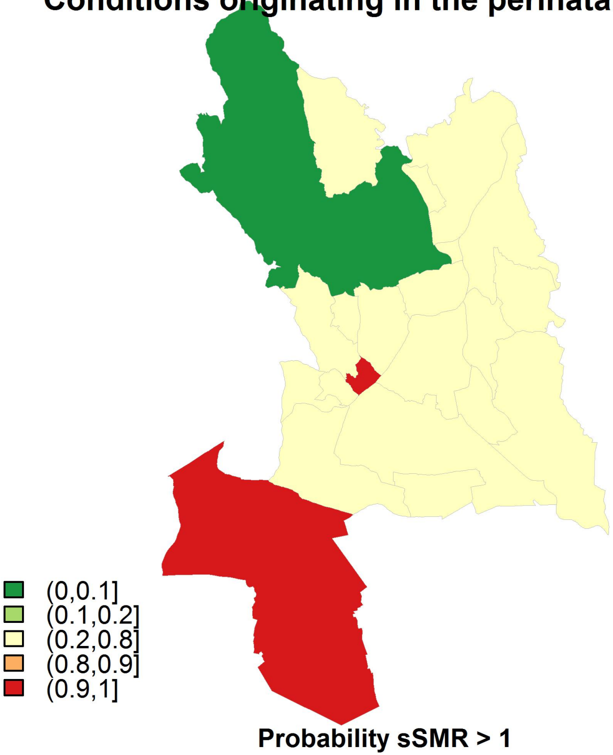

Kosice, Males, 1996 - 2008
Congenital heart disease

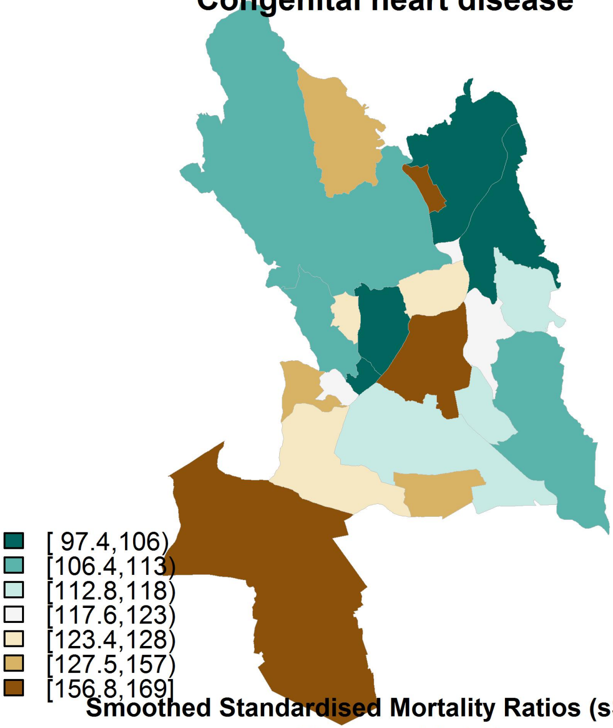

Smoothed Standardised Mortality Ratios (sSMR)
with respect to EU

Kosice, Males, 1996 - 2008
Congenital heart disease

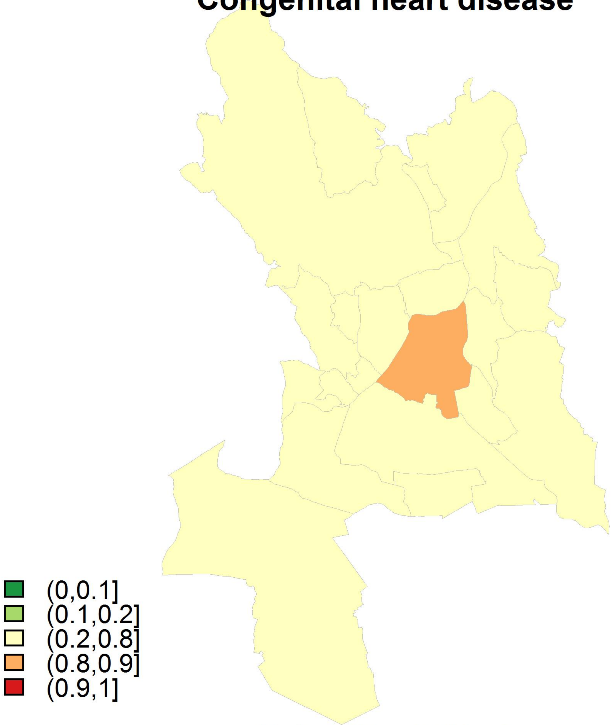

Probability sSMR > 1

Kosice, Females, 1996 - 2008
MN colon

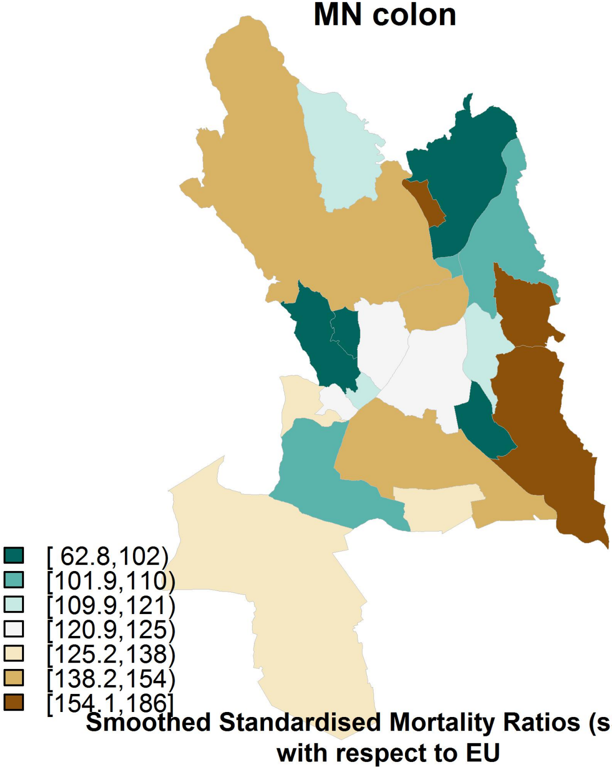

Smoothed Standardised Mortality Ratios (sSMR) with respect to EU

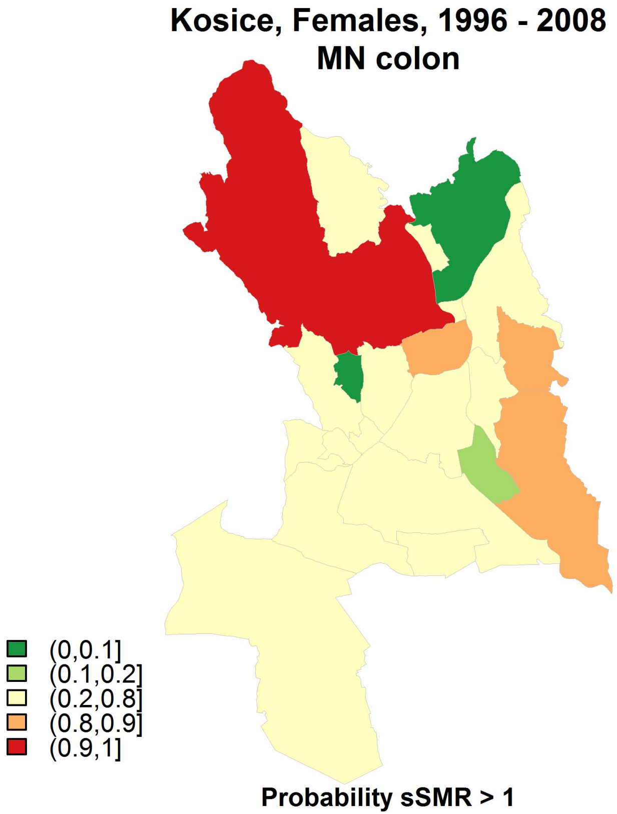

Kosice, Females, 1996 - 2008
MN colon
(0,0.1]
(0.1,0.2]
(0.2,0.8]
(0.8,0.9]
(0.9,1]
Probability sSMR > 1

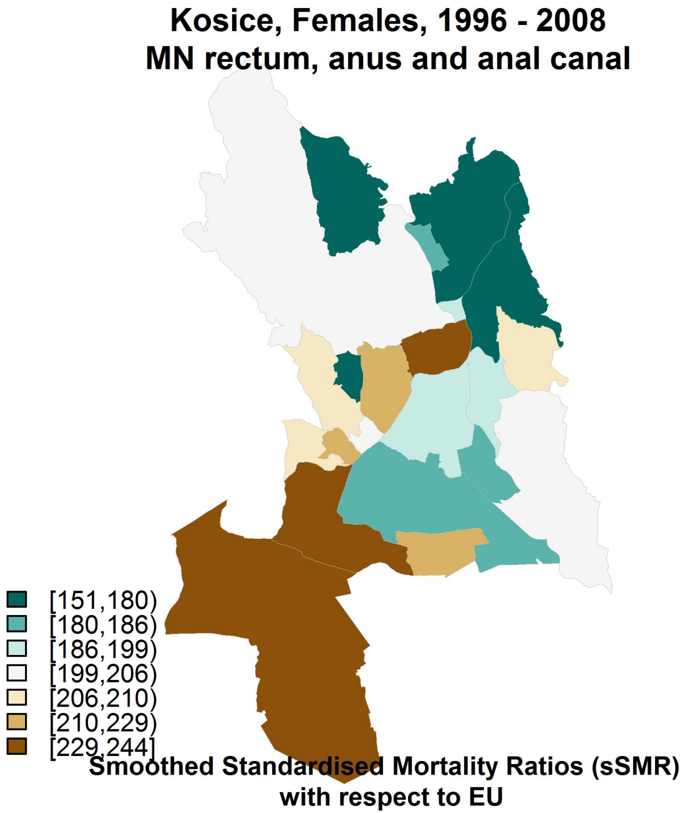
Kosice, Females, 1996 - 2008
MN rectum, anus and anal canal
[151,180)
[180,186)
[186,199)
[199,206)
[206,210)
[210,229)
[229,244]
Smoothed Standardised Mortality Ratios (sSMR)
with respect to EU

Kosice, Females, 1996 - 2008
MN rectum, anus and anal canal

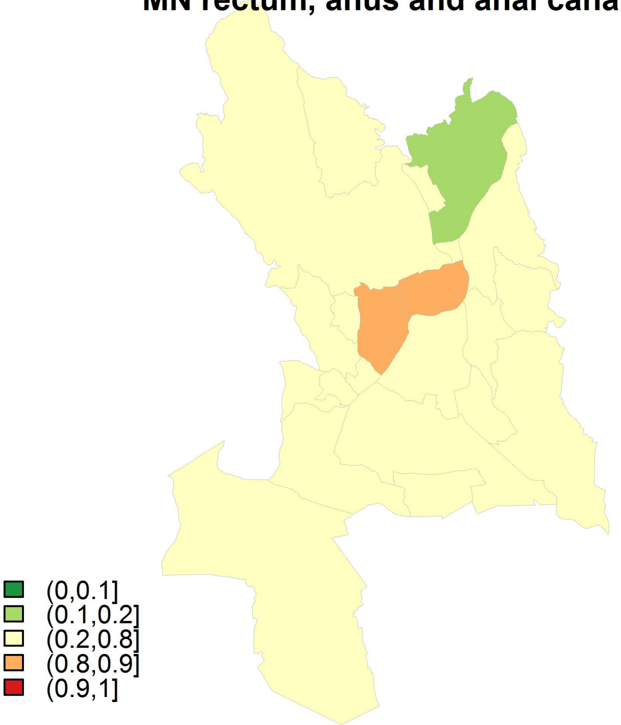

Probability sSMR > 1

Kosice, Females, 1996 - 2008
MN cervix uteri

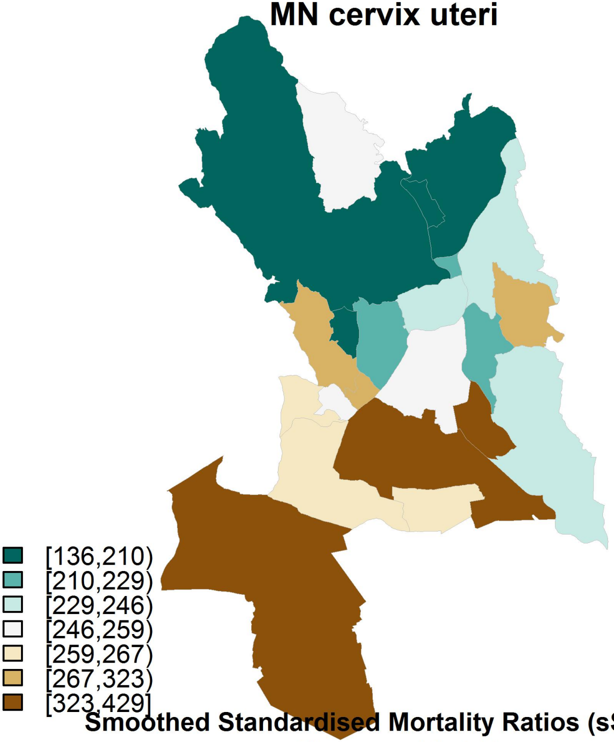

Smoothed Standardised Mortality Ratios (sSMR)
with respect to EU

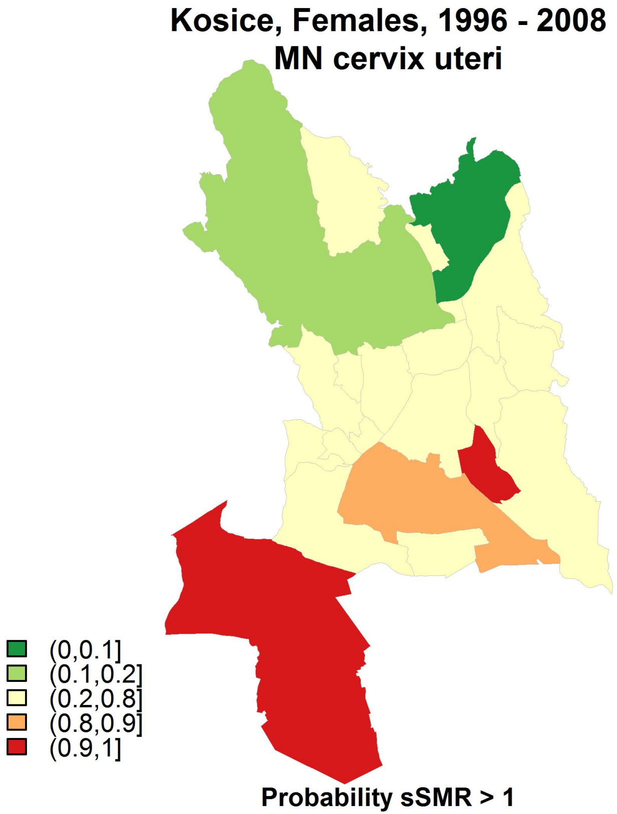
Kosice, Females, 1996 - 2008
MN cervix uteri
(0,0.1]
(0.1,0.2]
(0.2,0.8]
(0.8,0.9]
(0.9,1]
Probability sSMR > 1

Kosice, Females, 1996 - 2008
Rheumatic heart disease

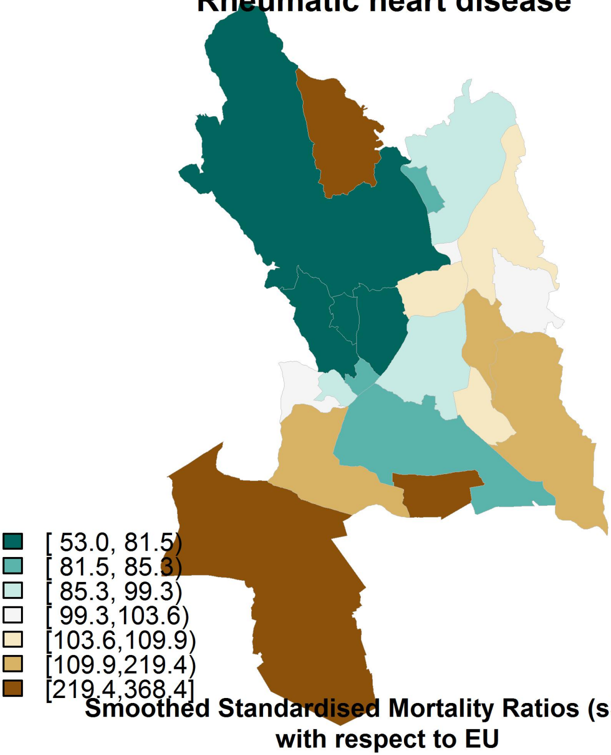

Smoothed Standardised Mortality Ratios (sSMR)
with respect to EU

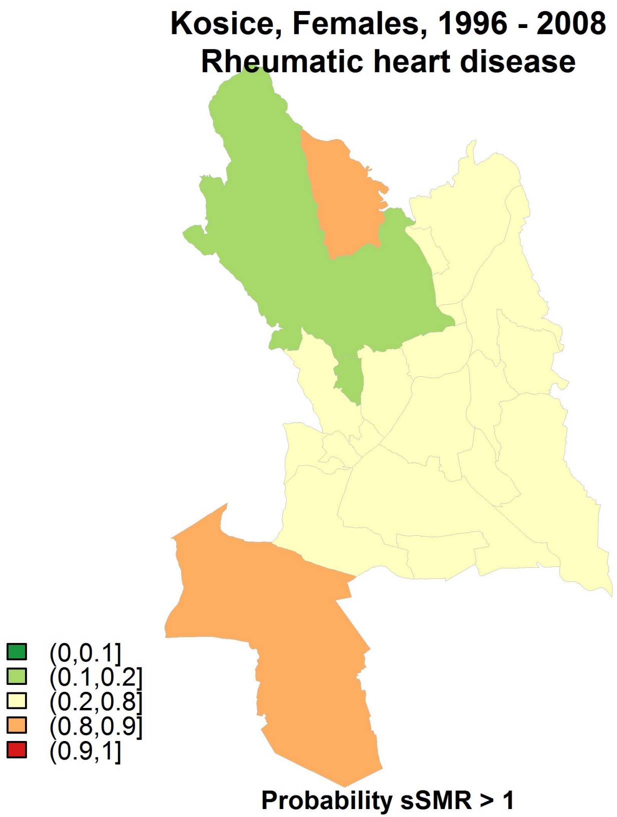
Kosice, Females, 1996 - 2008
Rheumatic heart disease
(0,0.1]
(0.1,0.2]
(0.2,0.8]
(0.8,0.9]
(0.9,1]
Probability sSMR > 1

Kosice, Females, 1996 - 2008
Hypertension

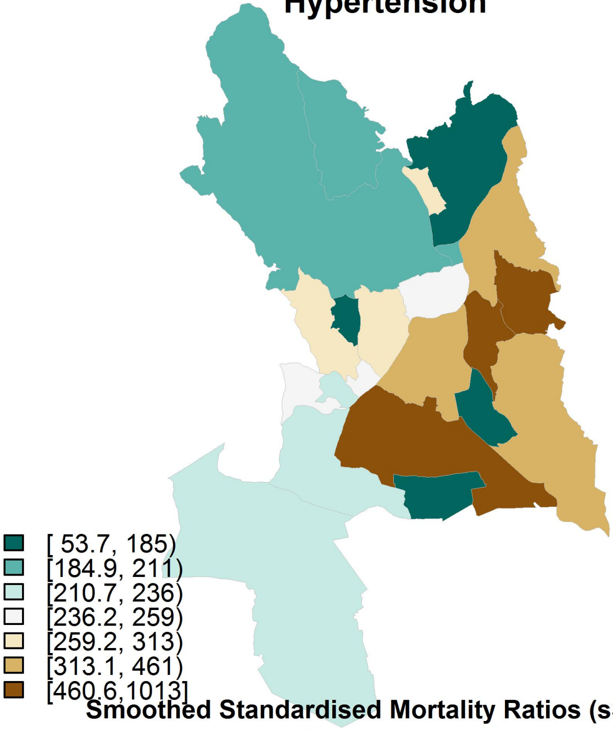

Smoothed Standardised Mortality Ratios (sSMR)
with respect to EU

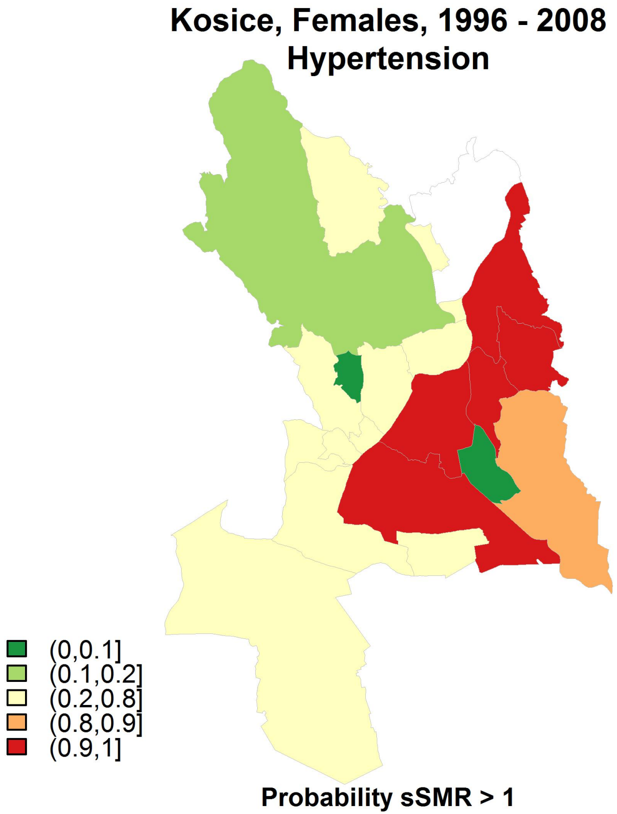
Kosice, Females, 1996 - 2008
Hypertension
(0,0.1]
(0.1,0.2]
(0.2,0.8]
(0.8,0.9]
(0.9,1]
Probability sSMR > 1

Kosice, Females, 1996 - 2008
Heart failure

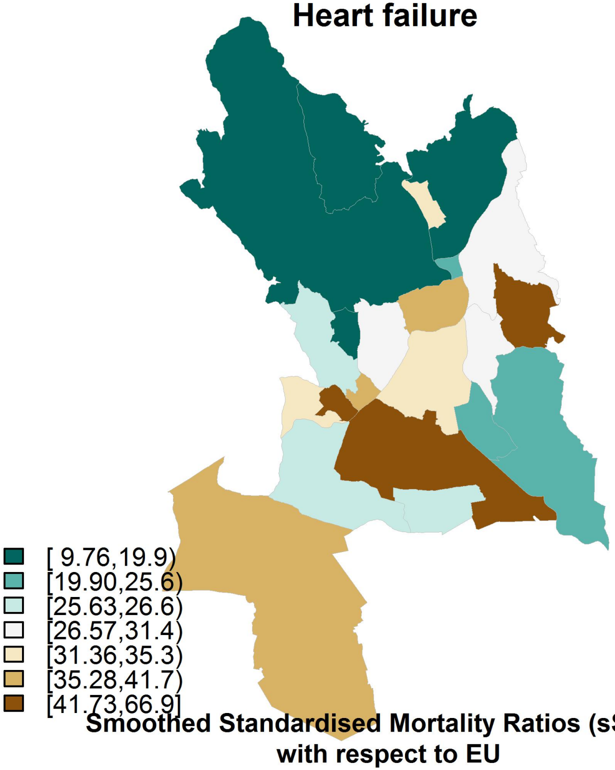

Smoothed Standardised Mortality Ratios (sSMR)
with respect to EU

Kosice, Females, 1996 - 2008
Heart failure

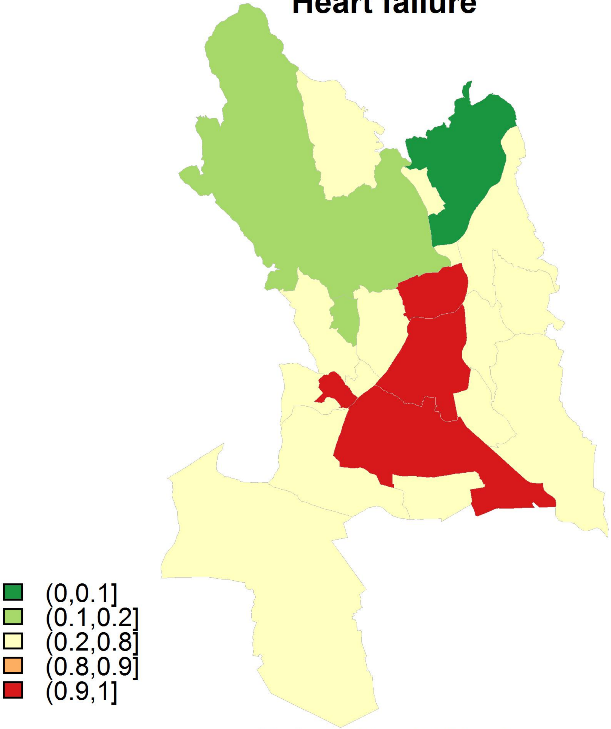

Probability sSMR > 1

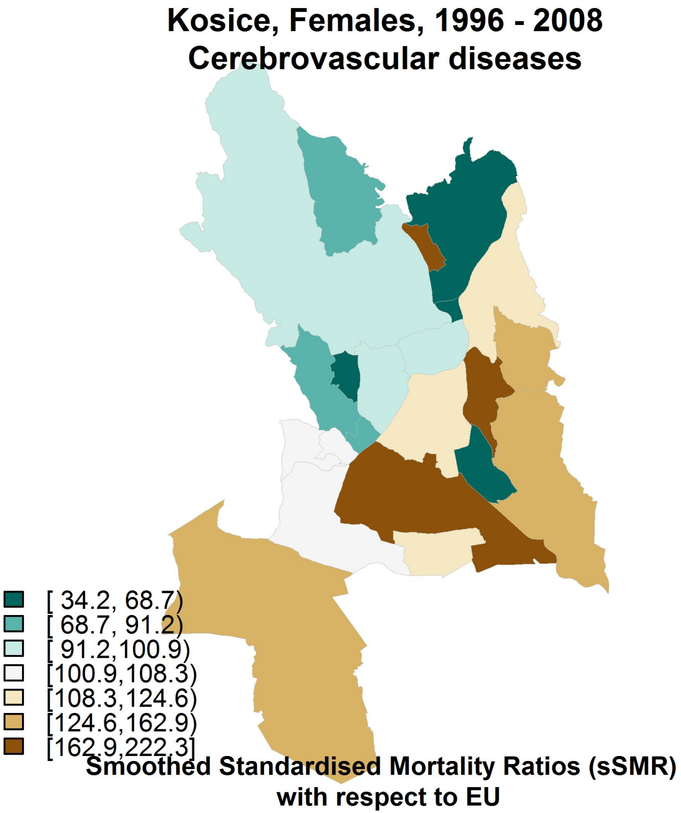
Kosice, Females, 1996 - 2008
Cerebrovascular diseases
[34.2, 68.7)
[68.7, 91.2)
[91.2,100.9)
[100.9,108.3)
[108.3,124.6)
[124.6,162.9)
[162.9,222.3]
Smoothed Standardised Mortality Ratios (sSMR)
with respect to EU

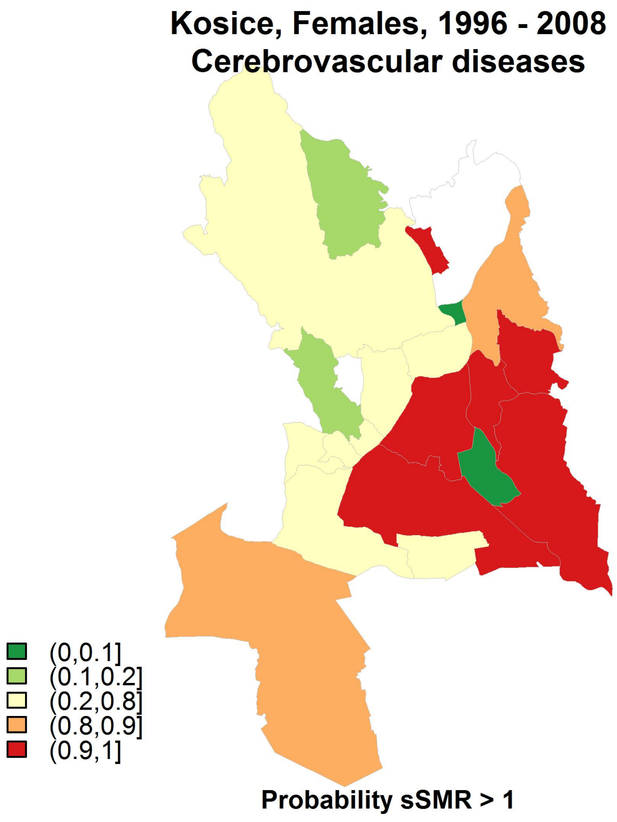
Kosice, Females, 1996 - 2008
Cerebrovascular diseases
(0,0.1]
(0.1,0.2]
(0.2,0.8]
(0.8,0.9]
(0.9,1]
Probability sSMR > 1

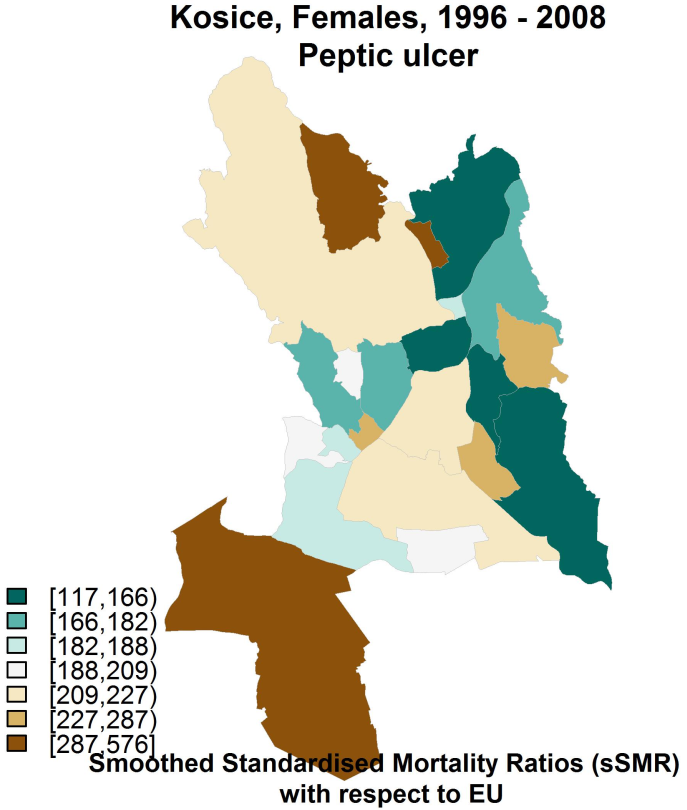
Kosice, Females, 1996 - 2008
Peptic ulcer
[117,166)
[166,182)
[182,188)
[188,209)
[209,227)
[227,287)
[287,576]
Smoothed Standardised Mortality Ratios (sSMR)
with respect to EU

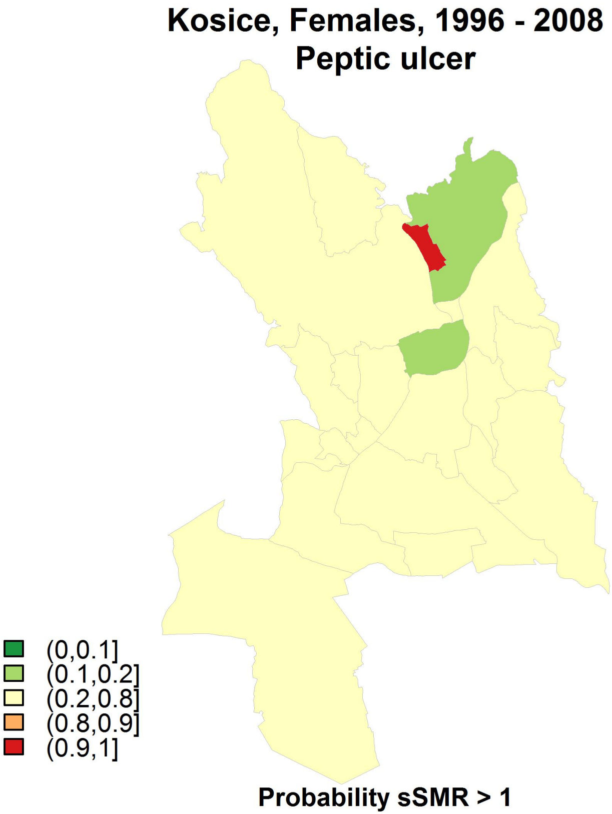
Kosice, Females, 1996 - 2008
Peptic ulcer
(0,0.1]
(0.1,0.2]
(0.2,0.8]
(0.8,0.9]
(0.9,1]
Probability sSMR > 1

Kosice, Females, 1996 - 2008
Renal failure

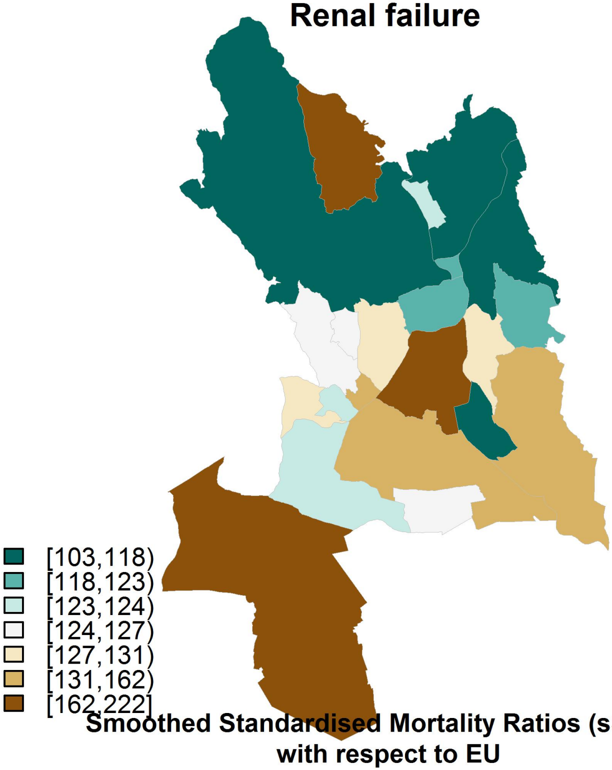

Smoothed Standardised Mortality Ratios (sSMR)
with respect to EU

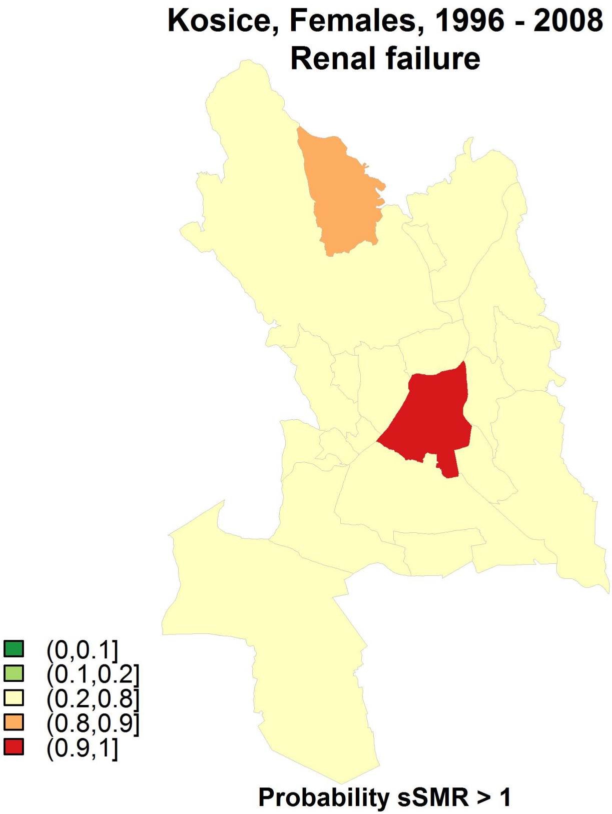

Kosice, Females, 1996 - 2008
Renal failure
(0,0.1]
(0.1,0.2]
(0.2,0.8]
(0.8,0.9]
(0.9,1]
Probability sSMR > 1

Kosice, Females, 1996 - 2008
Conditions originating in the perinatal period

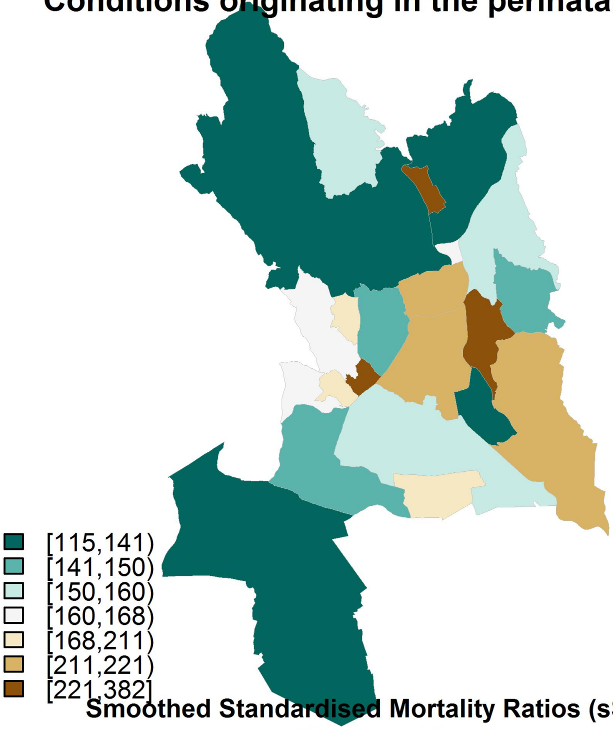

Smoothed Standardised Mortality Ratios (sSMR)
with respect to EU

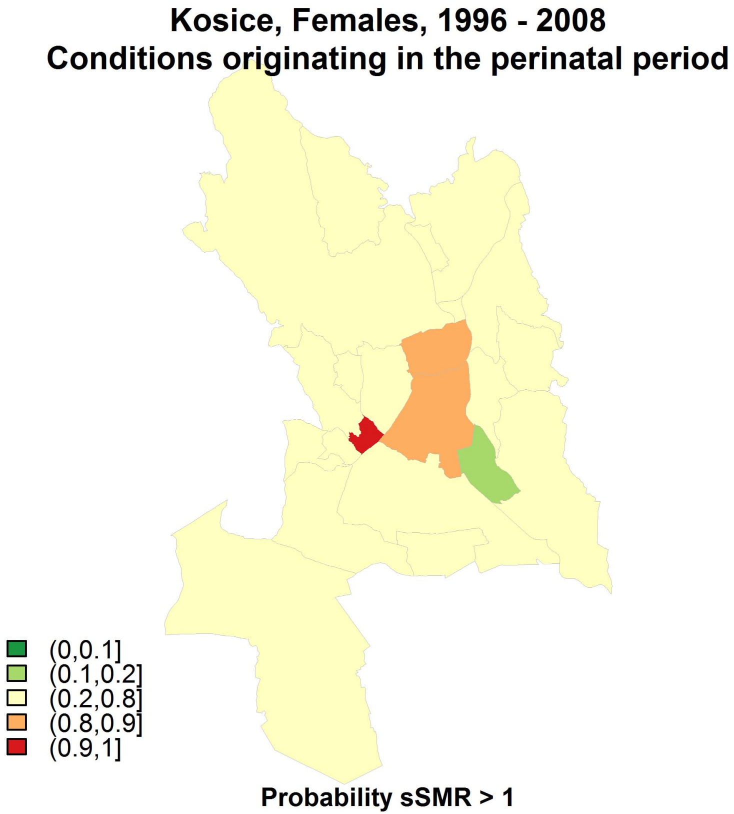
Kosice, Females, 1996 - 2008
Conditions originating in the perinatal period
(0,0.1]
(0.1,0.2]
(0.2,0.8]
(0.8,0.9]
(0.9,1]
Probability sSMR > 1

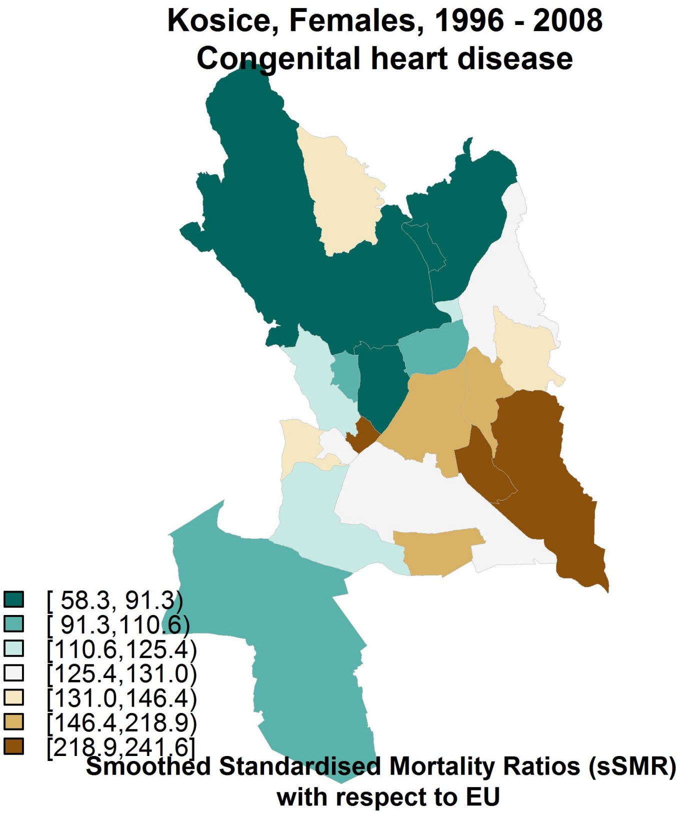
Kosice, Females, 1996 - 2008
Congenital heart disease
[58.3, 91.3)
[91.3,110.6)
[110.6,125.4)
[125.4,131.0)
[131.0,146.4)
[146.4,218.9)
[218.9,241.6]
Smoothed Standardised Mortality Ratios (sSMR)
with respect to EU

Kosice, Females, 1996 - 2008
Congenital heart disease

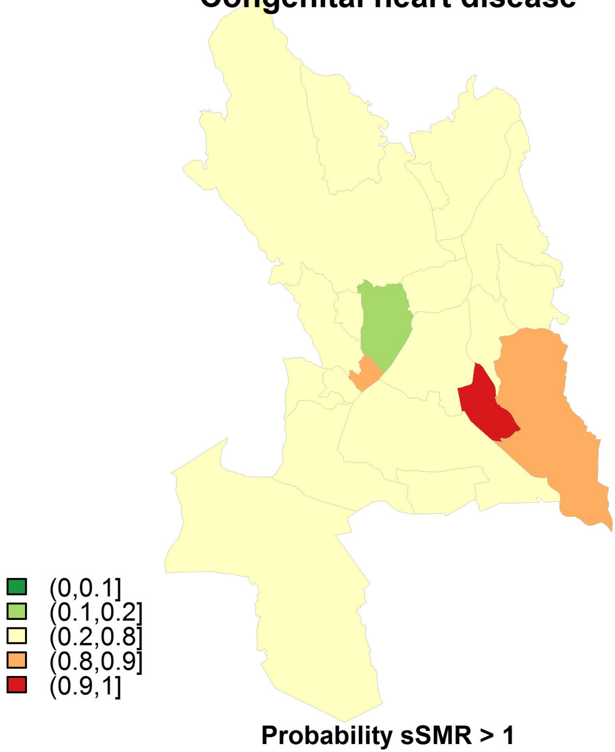

Supplement: Additional file 9 — Cause-specific mortality maps for Kosice. [file 1476-072X-13-8-S9.pdf]
