# Supplementary material for: Social differences in avoidable mortality between small areas of 15 European cities: an ecological study
Source: Int J Health Geogr. 2014 Mar 12;13:8. doi: 10.1186/1476-072X-13-8 (PMC4007807; doi:10.1186/1476-072X-13-8)
Supplement: Additional file 10 — Cause-specific mortality maps for Lisbon. [file 1476-072X-13-8-S10.pdf]

Lisbon, Males, 1995 - 2008
AIDS (HIV disease)

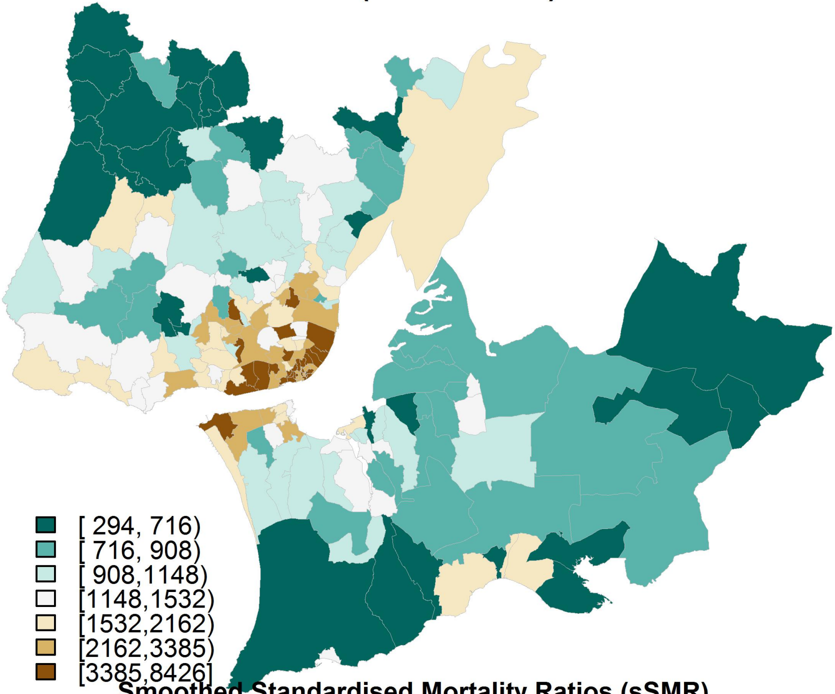

Smoothed Standardised Mortality Ratios (sSMR) with respect to EU

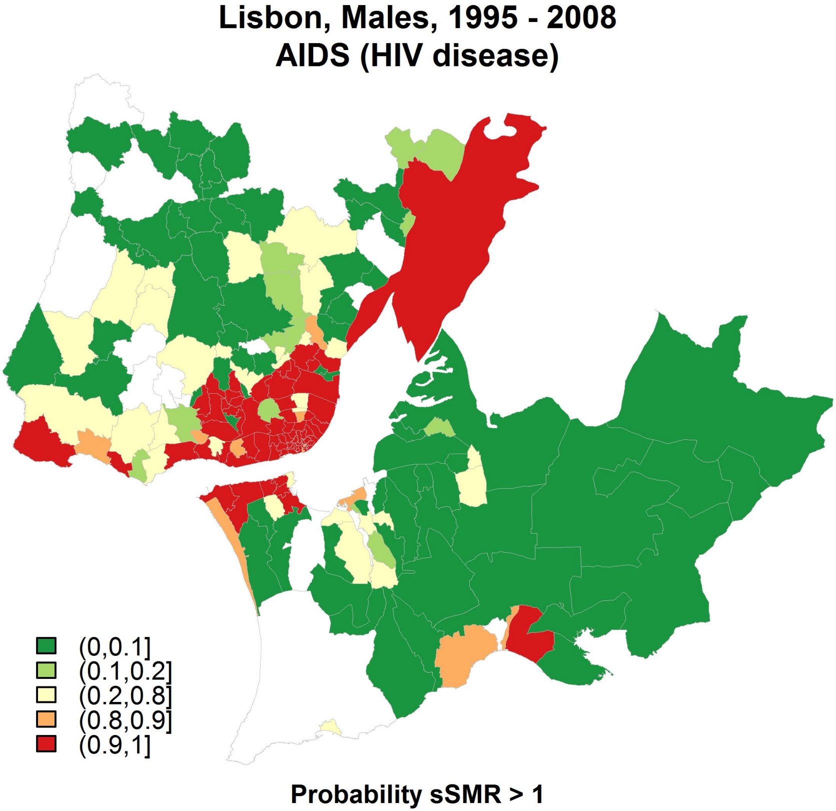
Lisbon, Males, 1995 - 2008
AIDS (HIV disease)
(0,0.1]
(0.1,0.2]
(0.2,0.8]
(0.8,0.9]
(0.9,1]
Probability sSMR > 1

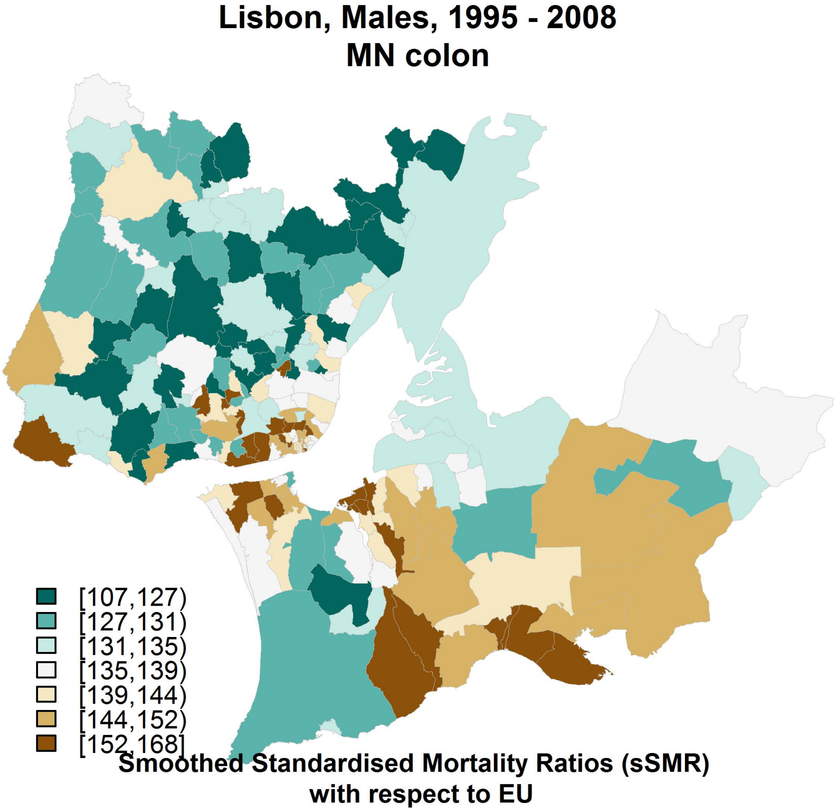

Lisbon, Males, 1995 - 2008
MN colon
[107,127)
[127,131)
[131,135)
[135,139)
[139,144)
[144,152)
[152,168]
Smoothed Standardised Mortality Ratios (sSMR)
with respect to EU

Lisbon, Males, 1995 - 2008
MN colon

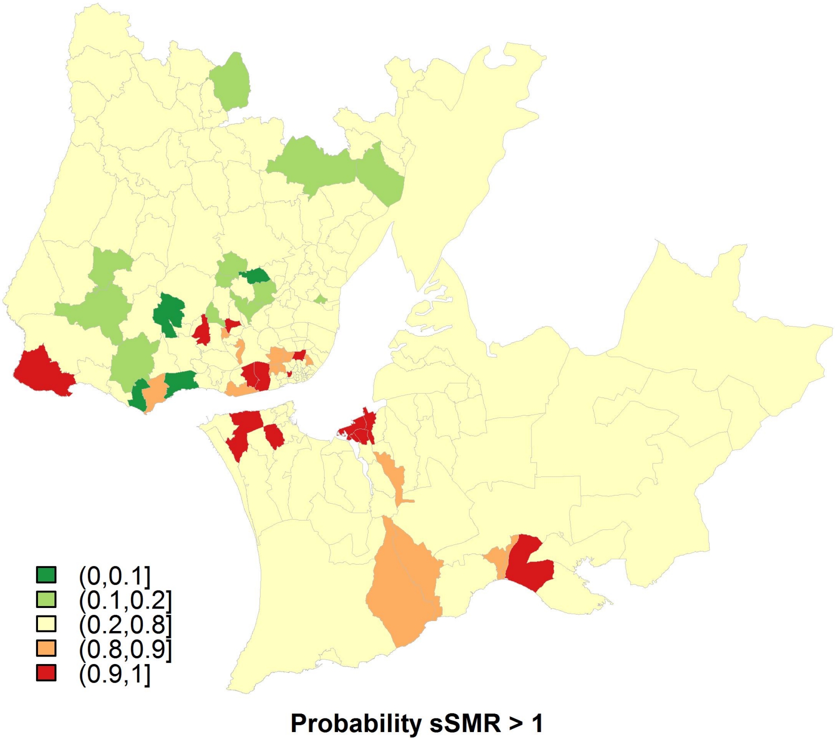

Lisbon, Males, 1995 - 2008
MN rectum, anus and anal canal

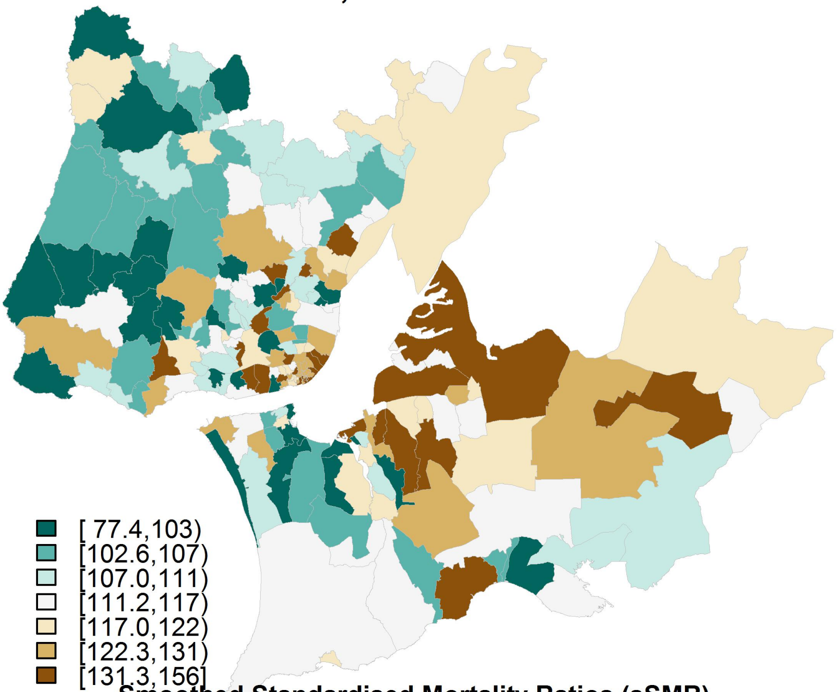

Smoothed Standardised Mortality Ratios (sSMR)
with respect to EU

Lisbon, Males, 1995 - 2008
MN rectum, anus and anal canal

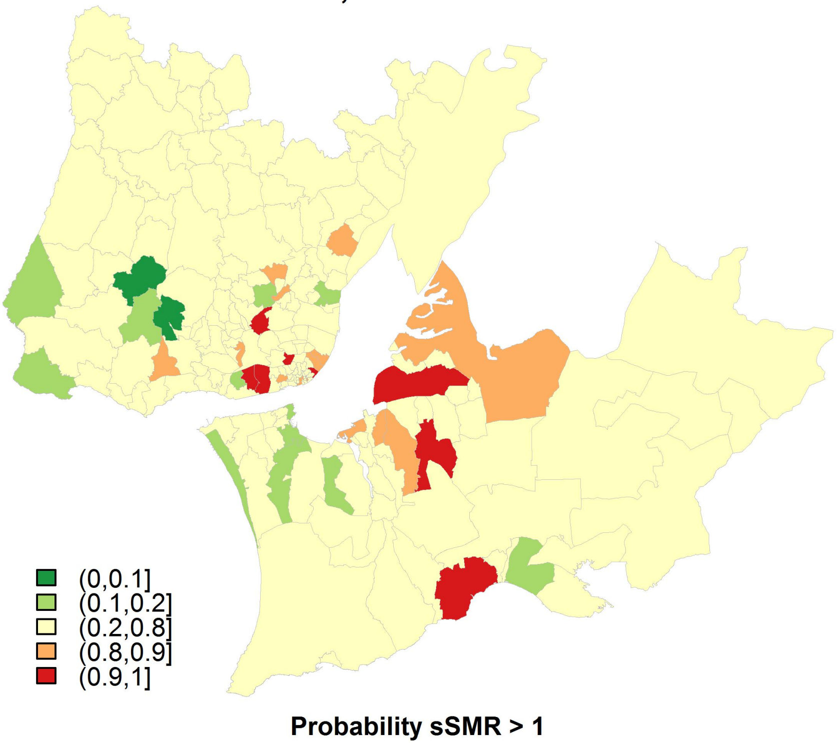

Lisbon, Males, 1995 - 2008
MN testes

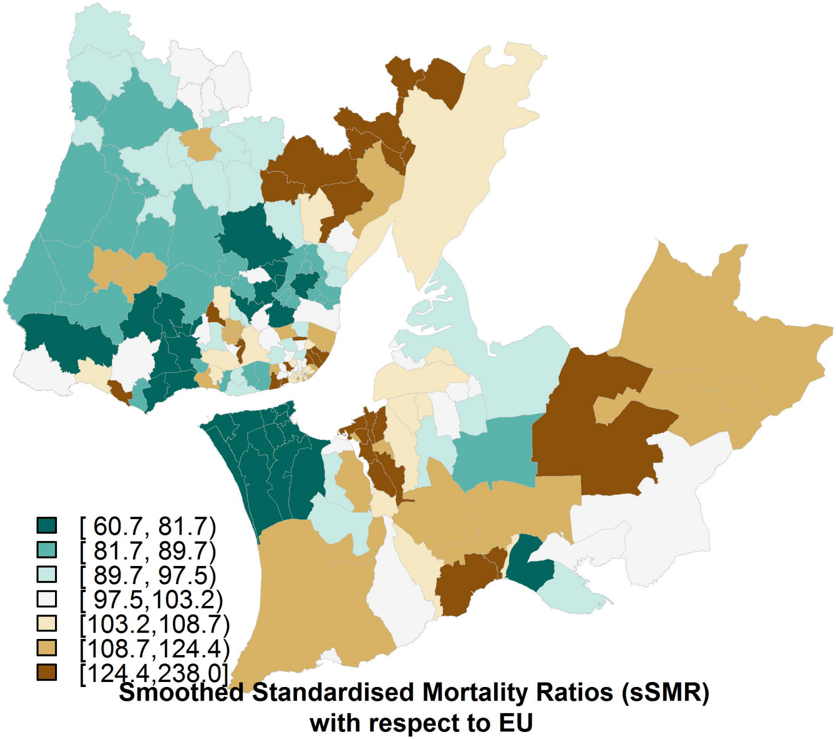

Smoothed Standardised Mortality Ratios (sSMR) with respect to EU

Lisbon, Males, 1995 - 2008
MN testes

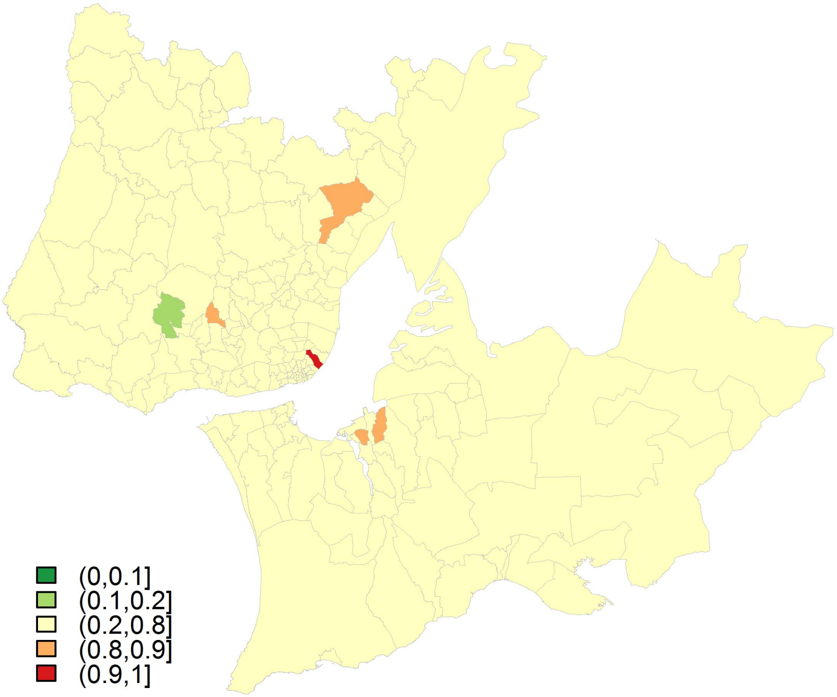

Probability sSMR > 1

Lisbon, Males, 1995 - 2008
Hodgkin's disease

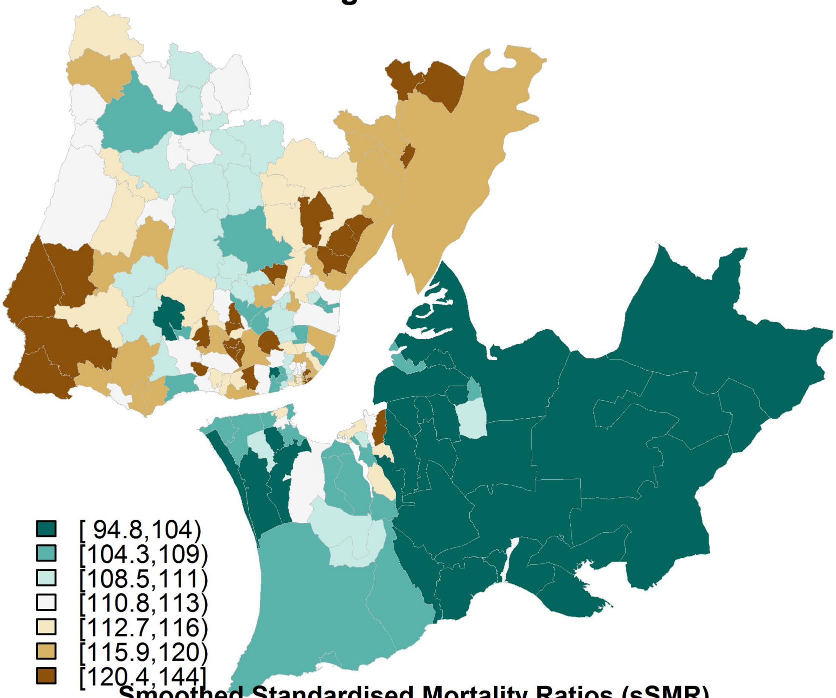

Smoothed Standardised Mortality Ratios (sSMR)
with respect to EU

Lisbon, Males, 1995 - 2008
Hodgkin's disease

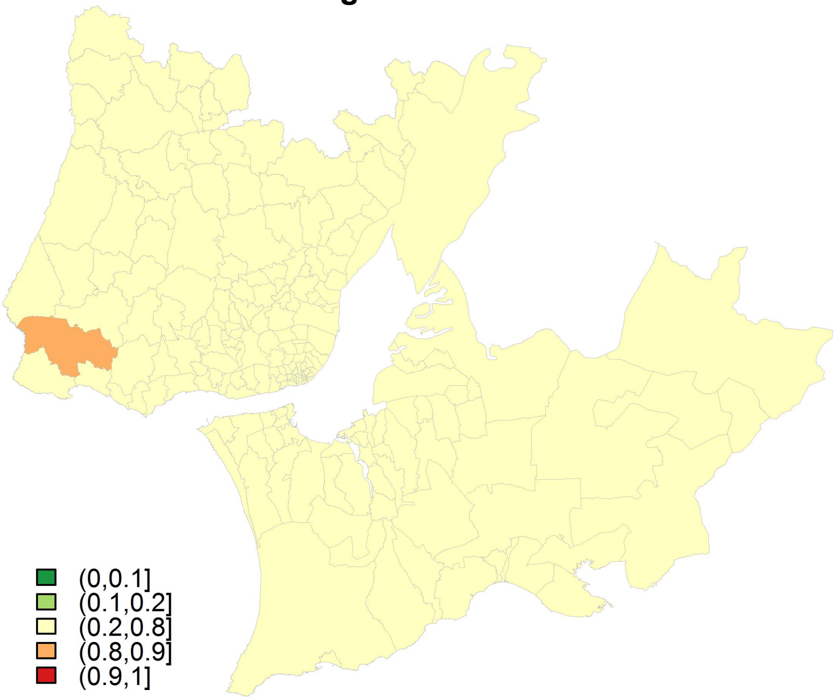

Probability sSMR > 1

Lisbon, Males, 1995 - 2008
Rheumatic heart disease

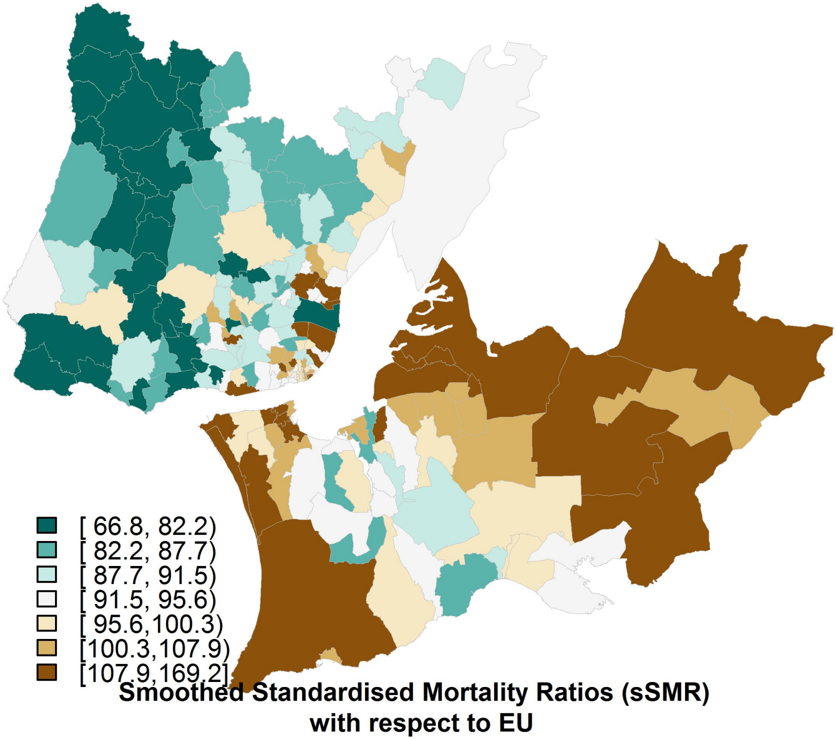

Smoothed Standardised Mortality Ratios (sSMR) with respect to EU

Lisbon, Males, 1995 - 2008
Rheumatic heart disease

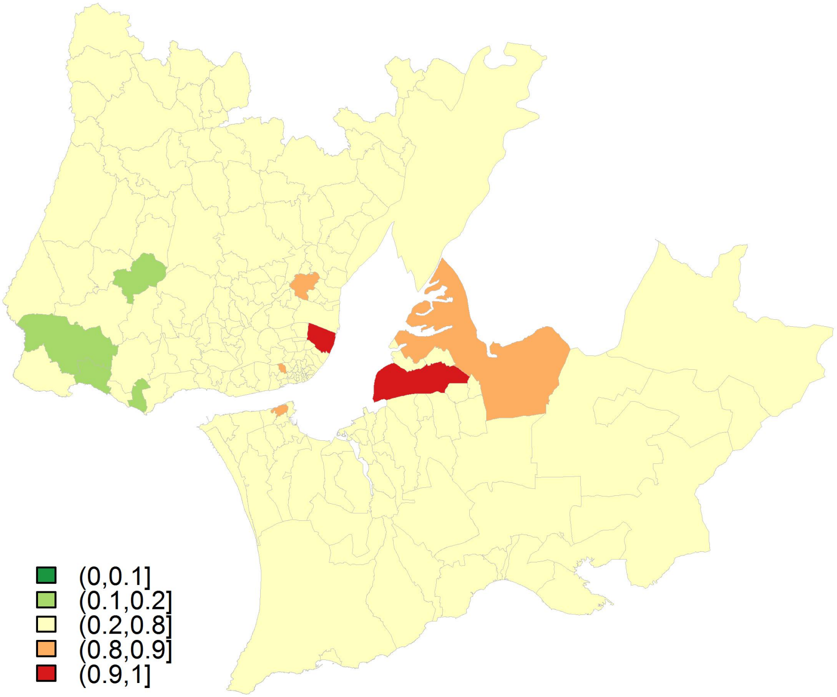

Probability sSMR > 1

Lisbon, Males, 1995 - 2008
Hypertension

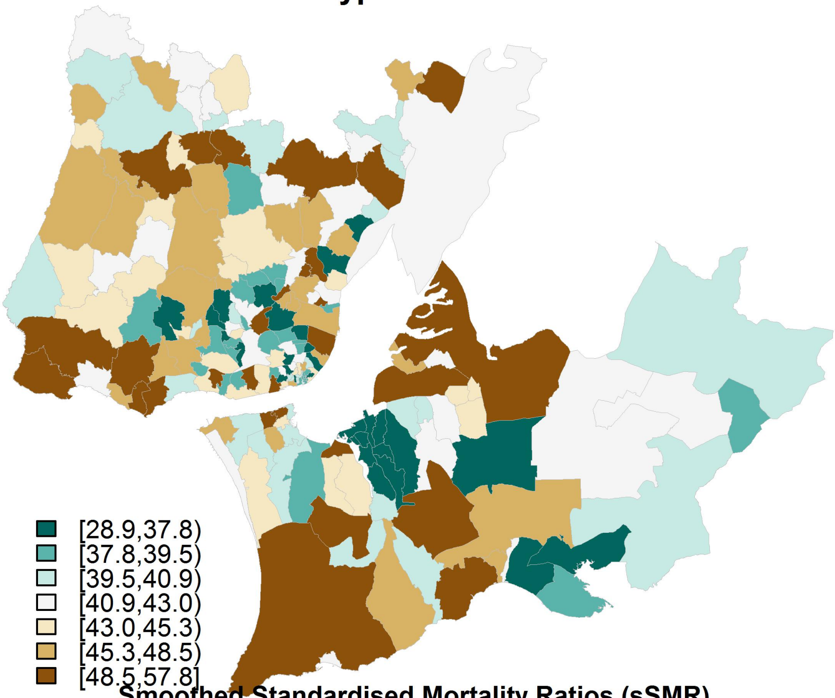

Smoothed Standardised Mortality Ratios (sSMR)
with respect to EU

Lisbon, Males, 1995 - 2008
Hypertension

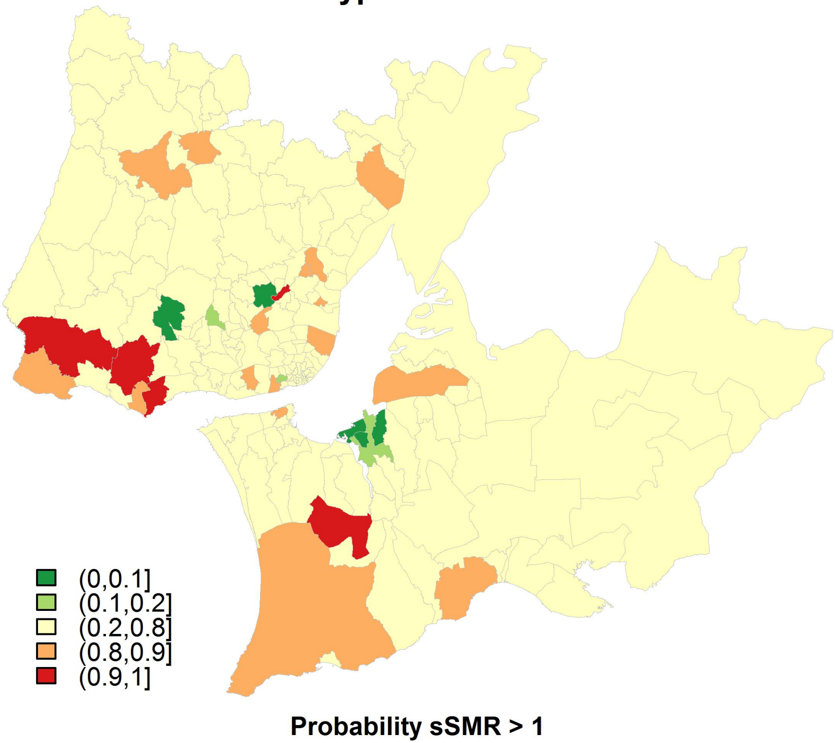

Lisbon, Males, 1995 - 2008
Heart failure

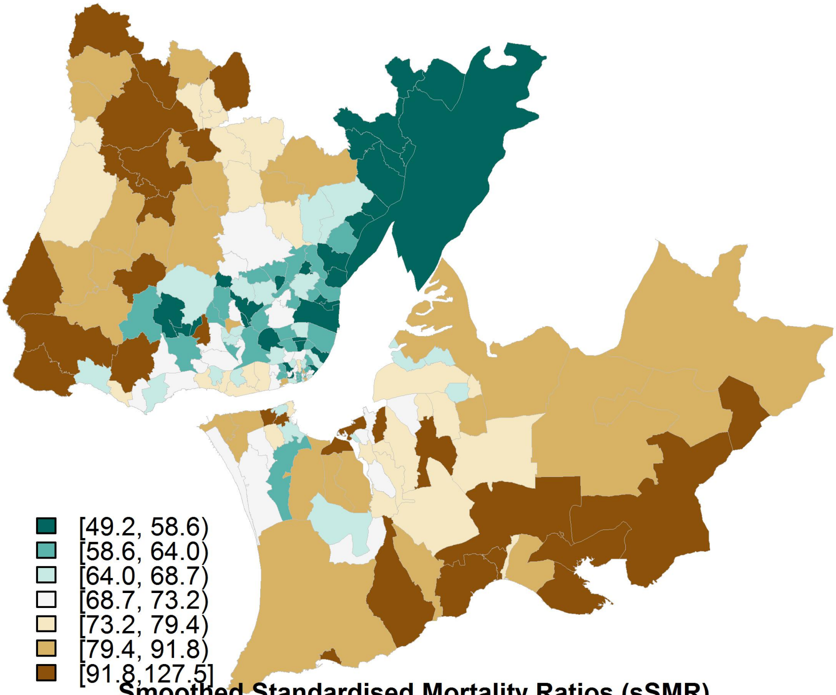

Smoothed Standardised Mortality Ratios (sSMR)
with respect to EU

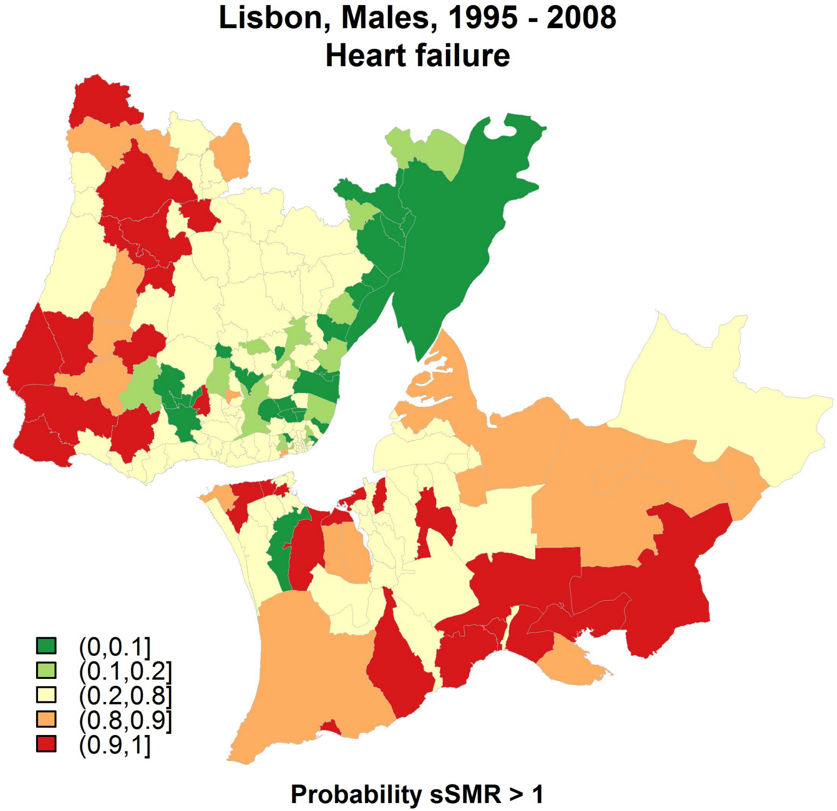
Lisbon, Males, 1995 - 2008
Heart failure
(0,0.1]
(0.1,0.2]
(0.2,0.8]
(0.8,0.9]
(0.9,1]
Probability sSMR > 1

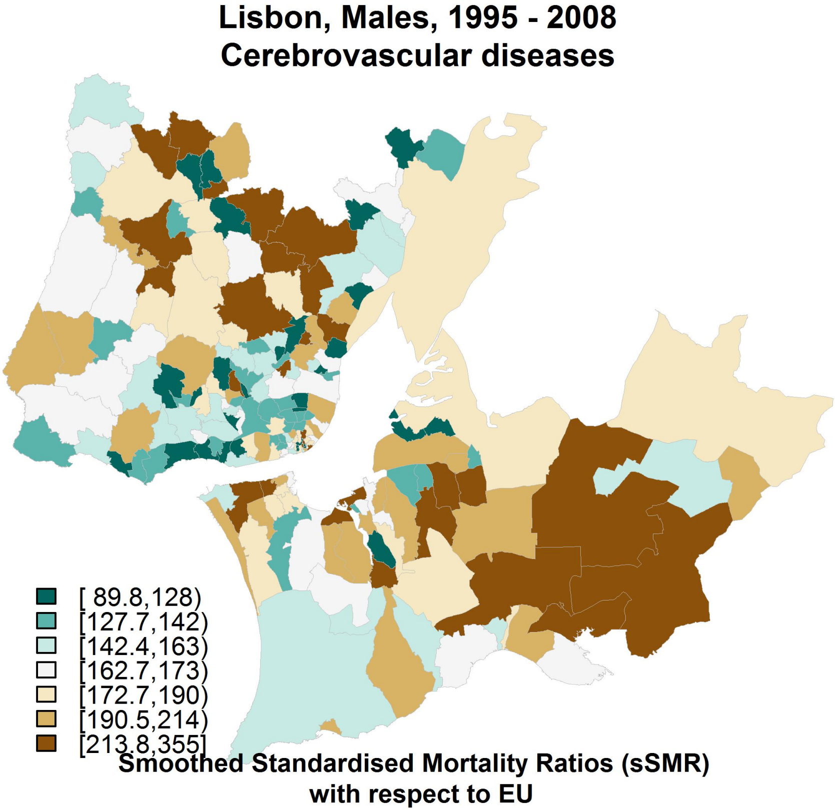
Lisbon, Males, 1995 - 2008
Cerebrovascular diseases
[89.8,128)
[127.7,142)
[142.4,163)
[162.7,173)
[172.7,190)
[190.5,214)
[213.8,355]
Smoothed Standardised Mortality Ratios (sSMR)
with respect to EU

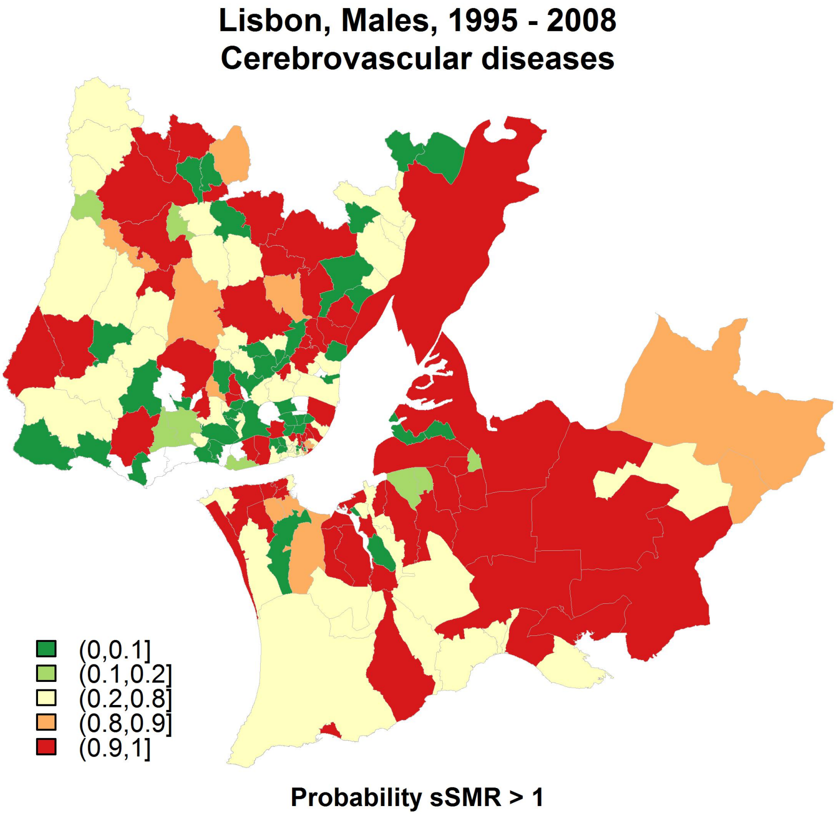

Lisbon, Males, 1995 - 2008
Cerebrovascular diseases
(0,0.1]
(0.1,0.2]
(0.2,0.8]
(0.8,0.9]
(0.9,1]
Probability sSMR > 1

Lisbon, Males, 1995 - 2008
Peptic ulcer

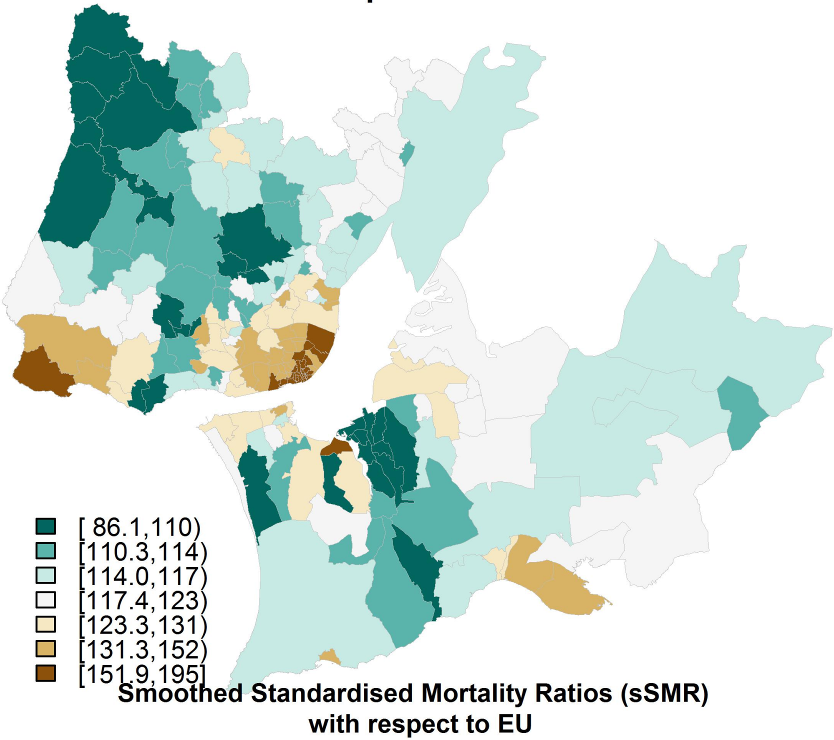

Smoothed Standardised Mortality Ratios (sSMR)
with respect to EU

Lisbon, Males, 1995 - 2008
Peptic ulcer

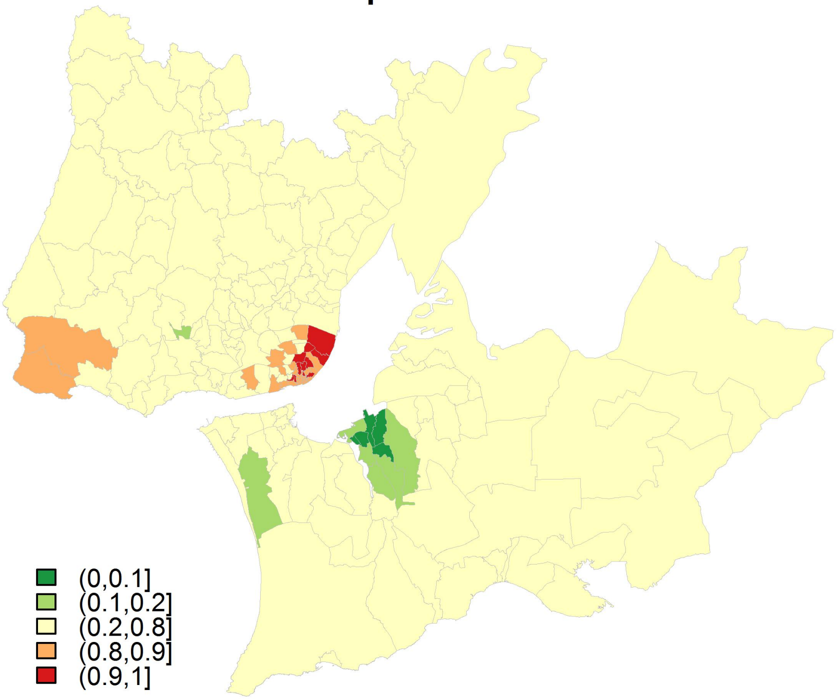

Probability sSMR > 1

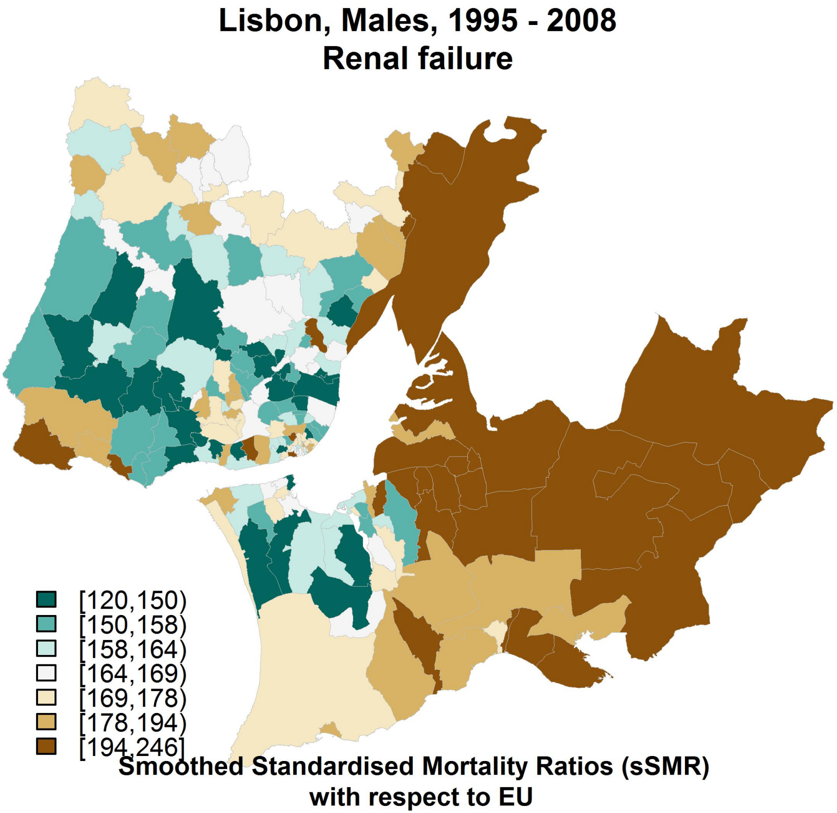
Lisbon, Males, 1995 - 2008
Renal failure
[120,150)
[150,158)
[158,164)
[164,169)
[169,178)
[178,194)
[194,246]
Smoothed Standardised Mortality Ratios (sSMR)
with respect to EU

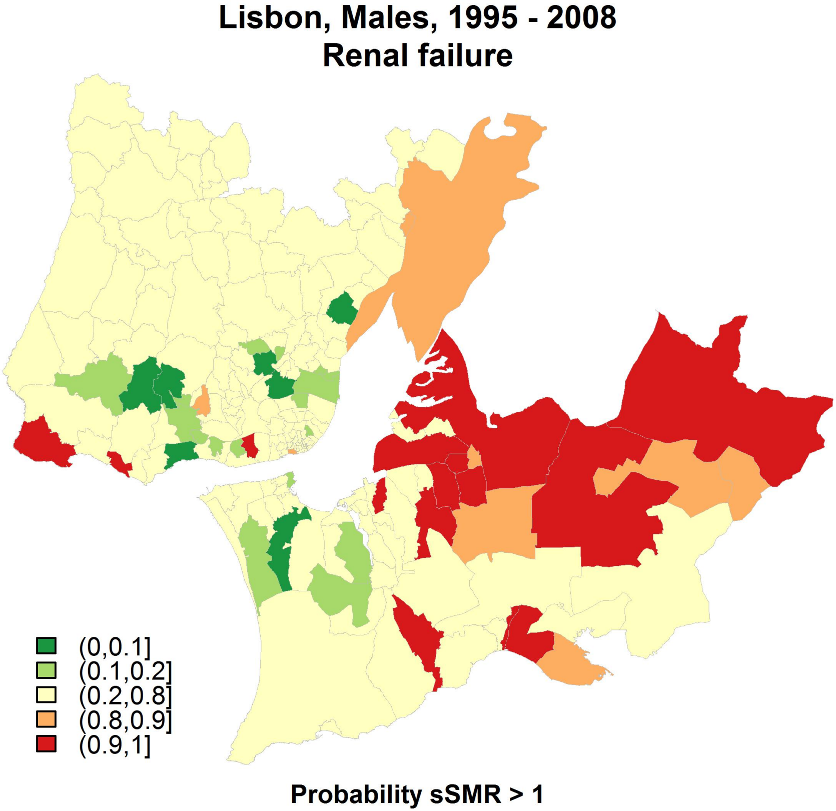
Lisbon, Males, 1995 - 2008
Renal failure
(0,0.1]
(0.1,0.2]
(0.2,0.8]
(0.8,0.9]
(0.9,1]
Probability sSMR > 1

Lisbon, Males, 1995 - 2008
Conditions originating in the perinatal period

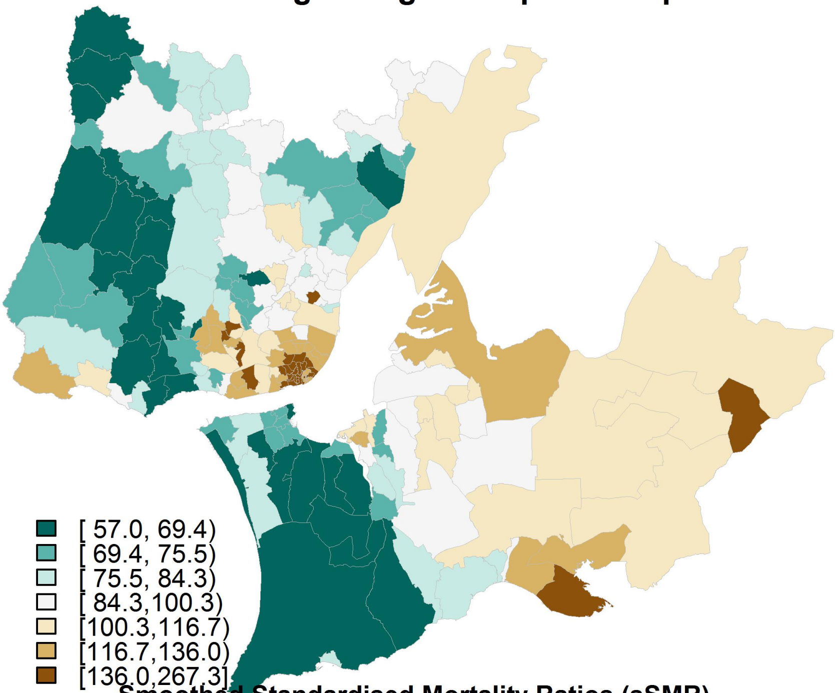

Smoothed Standardised Mortality Ratios (sSMR)
with respect to EU

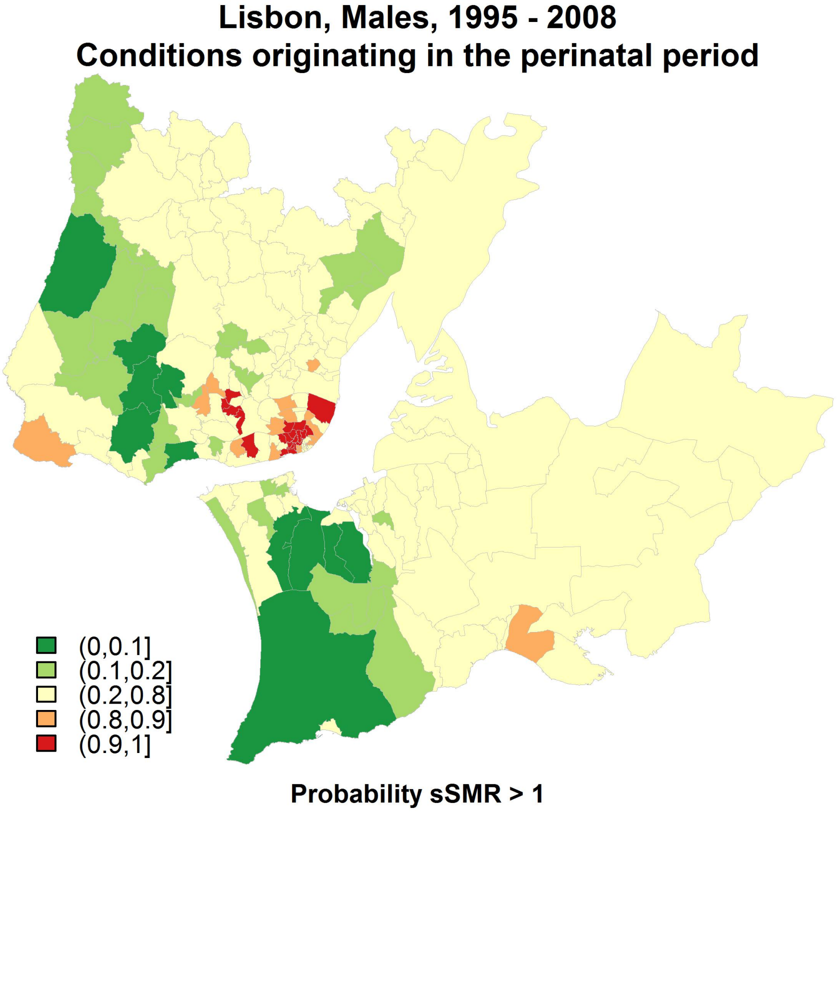
Lisbon, Males, 1995 - 2008
Conditions originating in the perinatal period
(0,0.1]
(0.1,0.2]
(0.2,0.8]
(0.8,0.9]
(0.9,1]
Probability sSMR > 1

Lisbon, Males, 1995 - 2008
Congenital heart disease

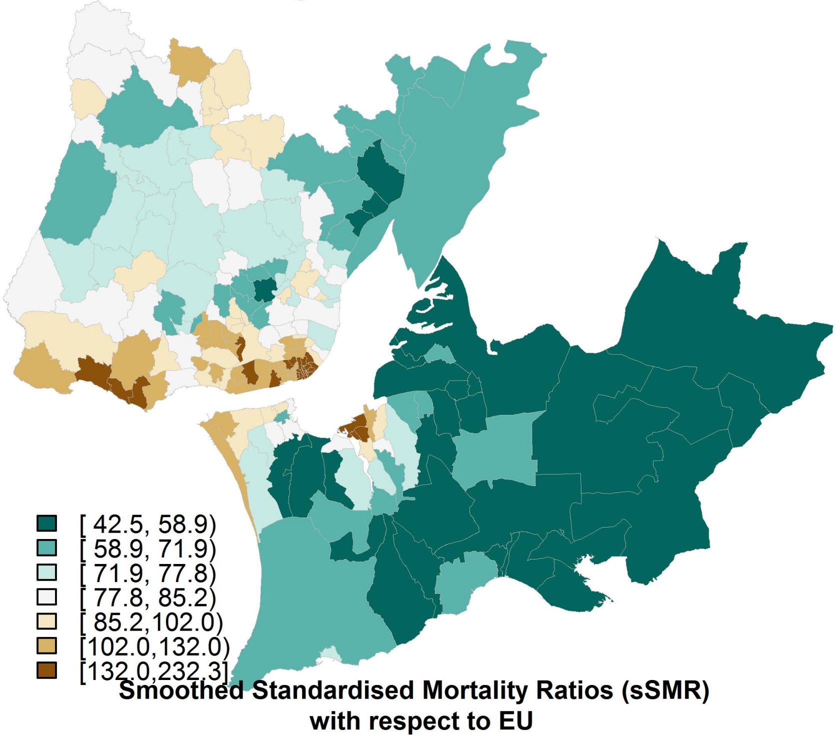

Smoothed Standardised Mortality Ratios (sSMR)
with respect to EU

Lisbon, Males, 1995 - 2008
Congenital heart disease

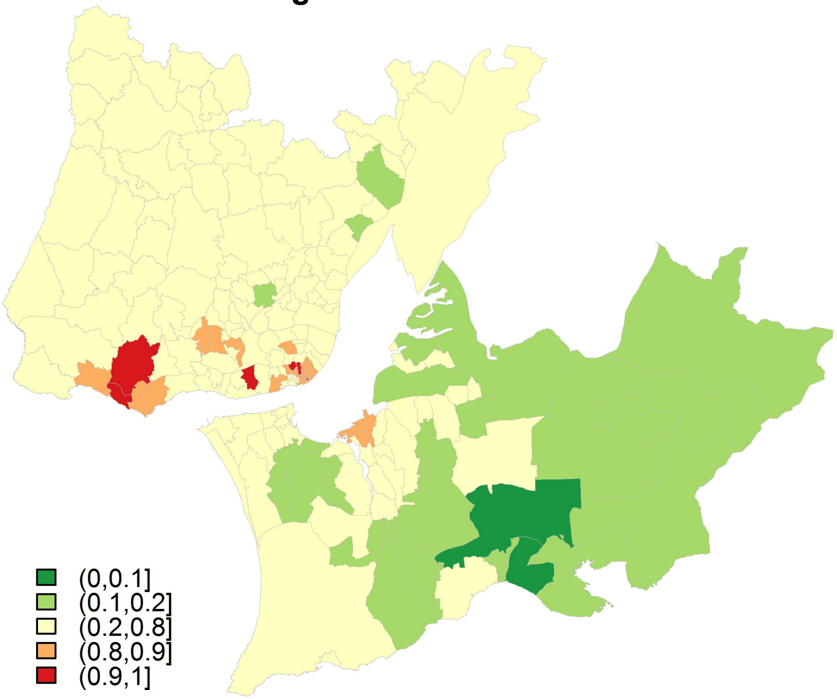

Probability sSMR > 1

Lisbon, Females, 1995 - 2008
AIDS (HIV disease)

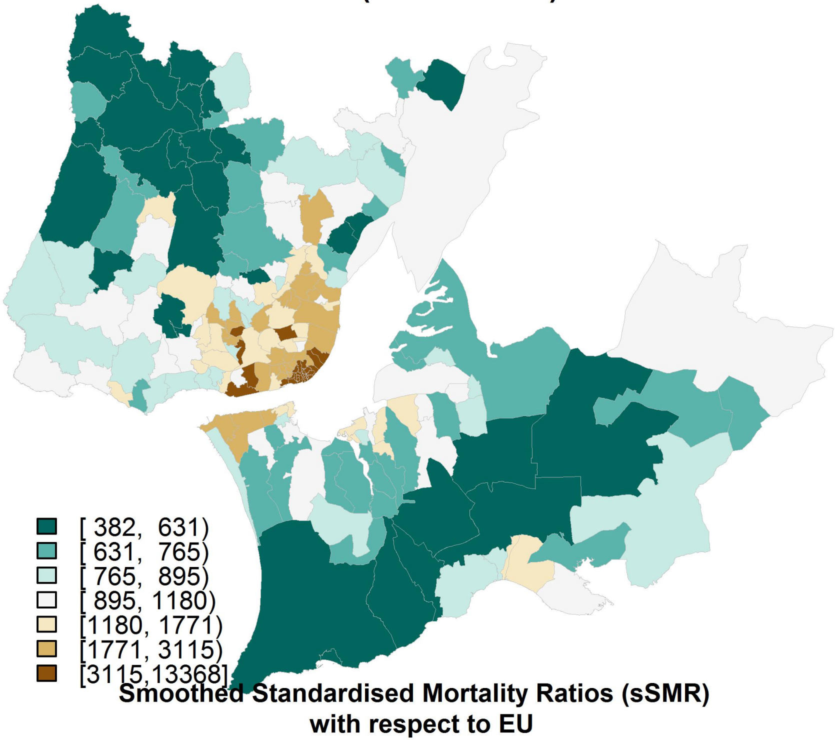

Smoothed Standardised Mortality Ratios (sSMR)
with respect to EU

Lisbon, Females, 1995 - 2008
AIDS (HIV disease)

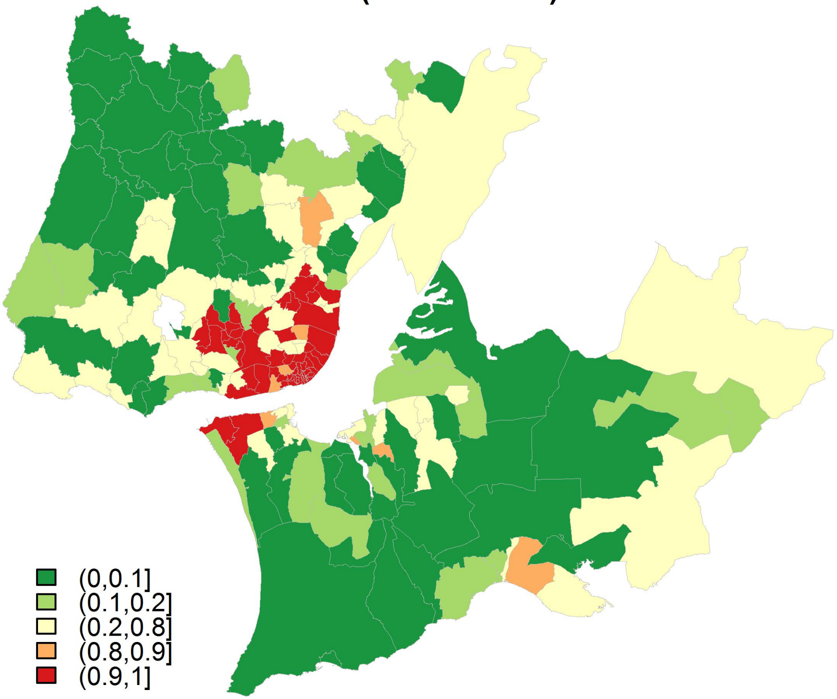

Probability sSMR > 1

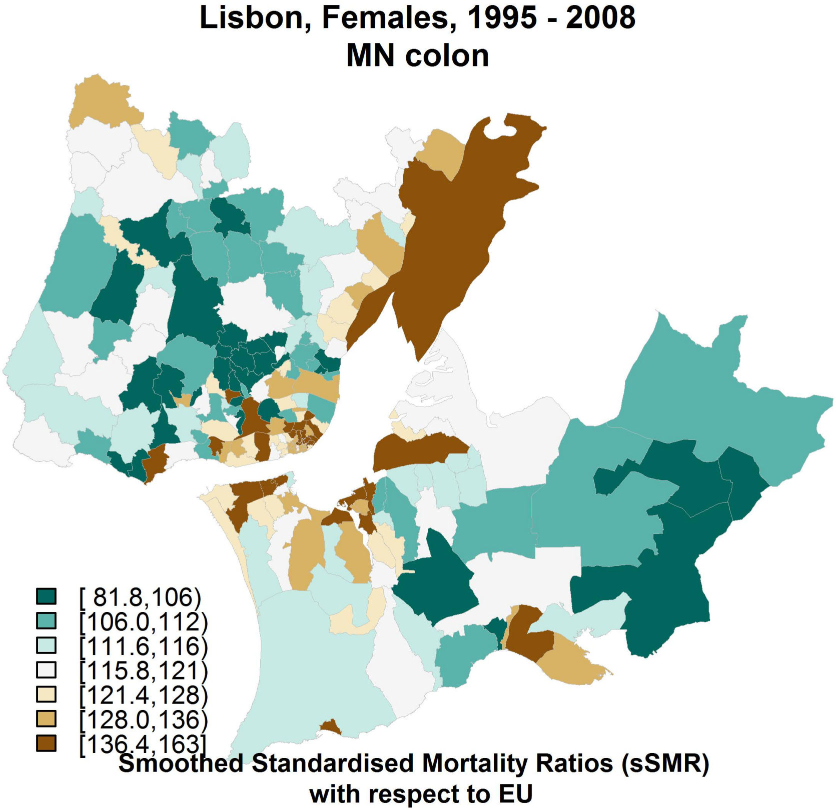
Lisbon, Females, 1995 - 2008
MN colon
[81.8,106)
[106.0,112)
[111.6,116)
[115.8,121)
[121.4,128)
[128.0,136)
[136.4,163]
Smoothed Standardised Mortality Ratios (sSMR)
with respect to EU

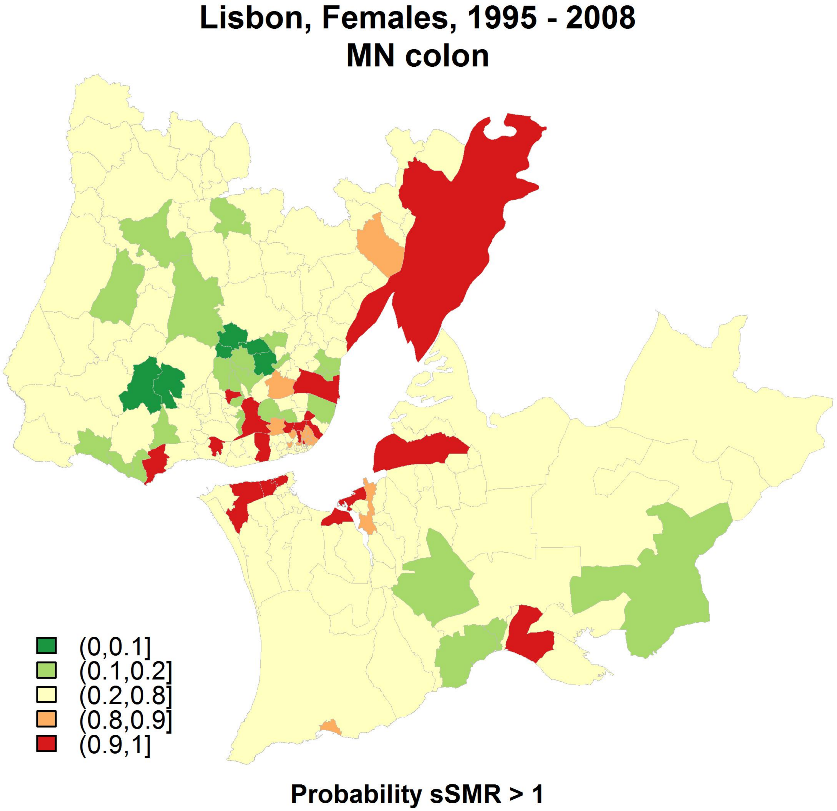
Lisbon, Females, 1995 - 2008
MN colon
(0,0.1]
(0.1,0.2]
(0.2,0.8]
(0.8,0.9]
(0.9,1]
Probability sSMR > 1

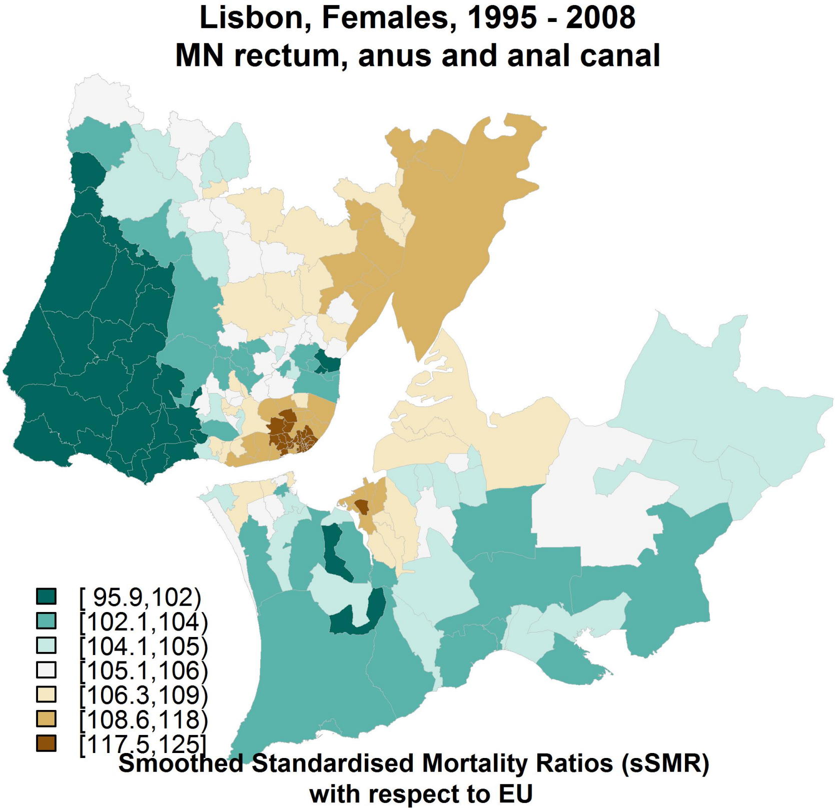
Lisbon, Females, 1995 - 2008
MN rectum, anus and anal canal
[95.9,102)
[102.1,104)
[104.1,105)
[105.1,106)
[106.3,109)
[108.6,118)
[117.5,125]
Smoothed Standardised Mortality Ratios (sSMR)
with respect to EU

Lisbon, Females, 1995 - 2008
MN rectum, anus and anal canal

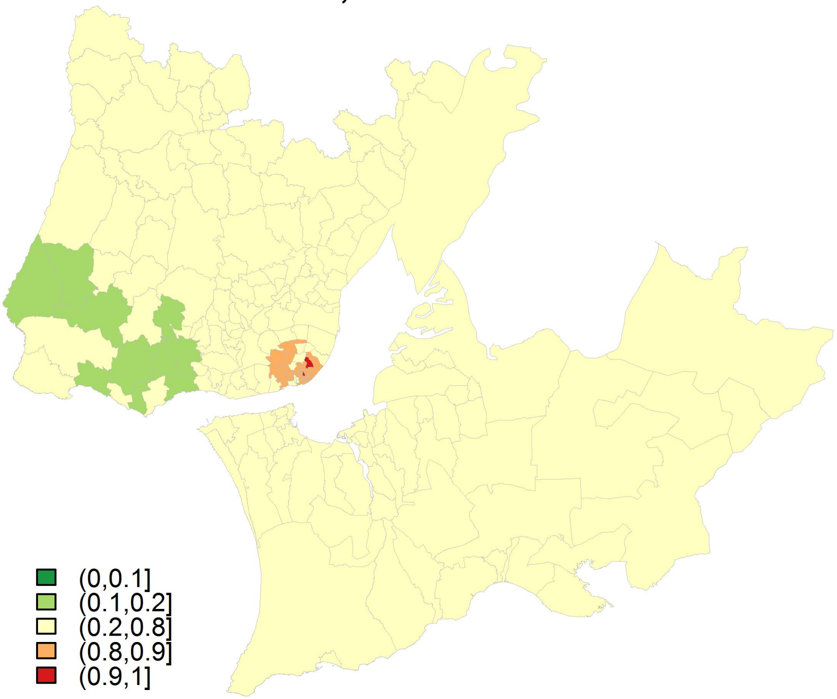

Probability sSMR > 1

Lisbon, Females, 1995 - 2008
MN cervix uteri

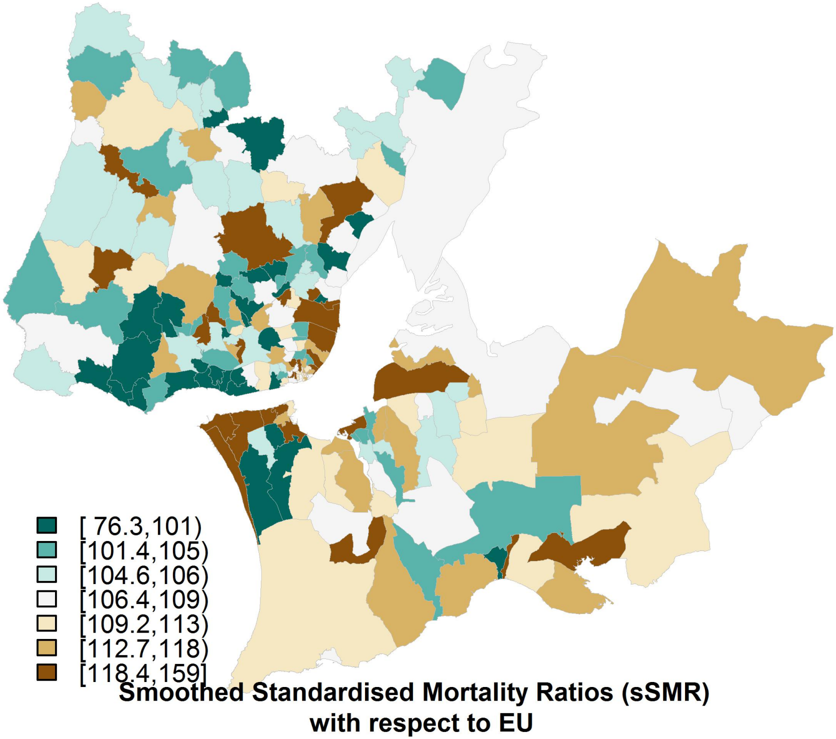

Smoothed Standardised Mortality Ratios (sSMR)
with respect to EU

Lisbon, Females, 1995 - 2008
MN cervix uteri

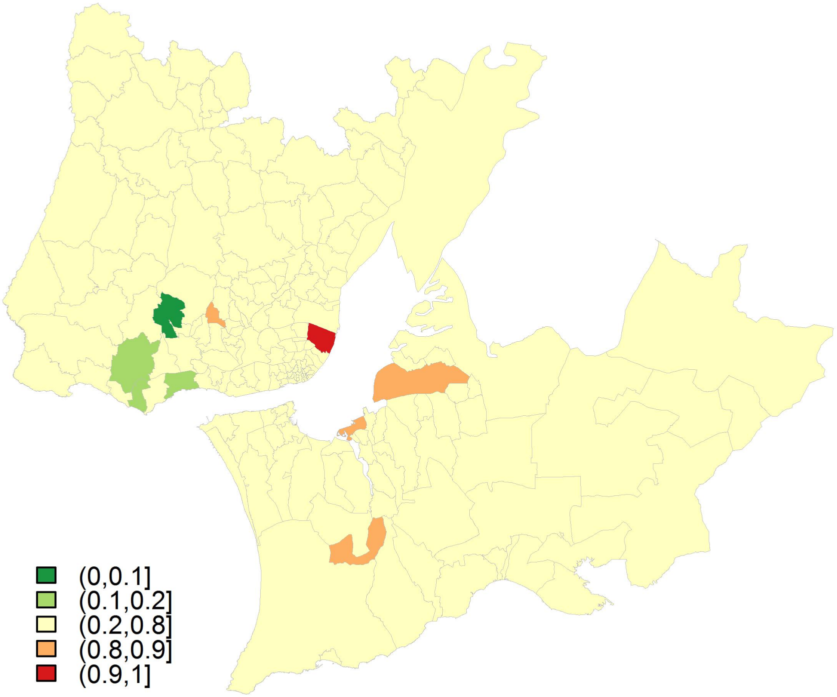

Probability sSMR > 1

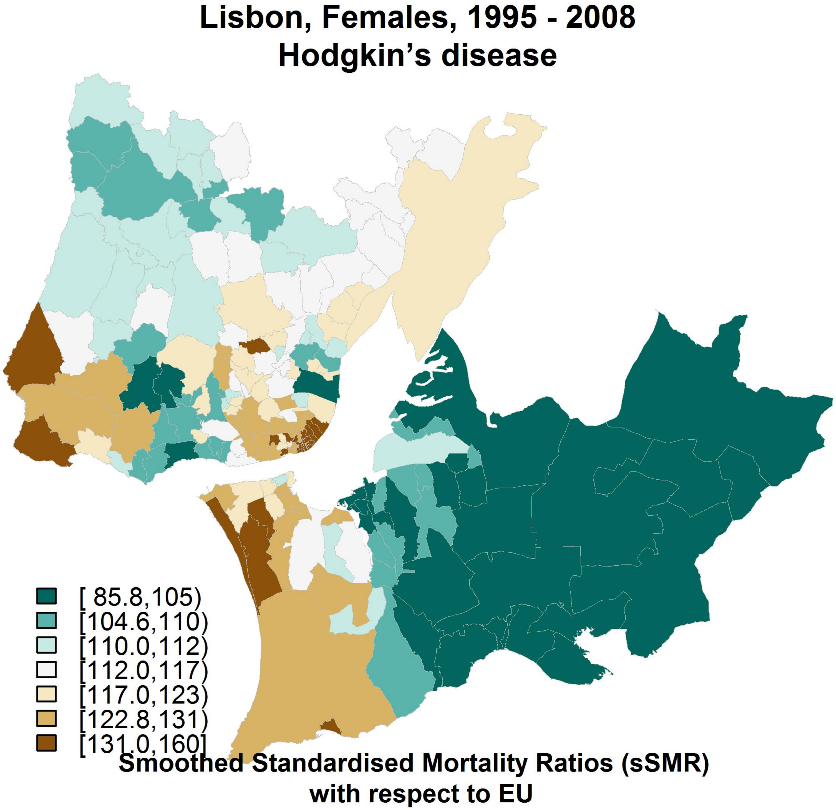
Lisbon, Females, 1995 - 2008
Hodgkin's disease
[85.8,105)
[104.6,110)
[110.0,112)
[112.0,117)
[117.0,123)
[122.8,131)
[131.0,160]
Smoothed Standardised Mortality Ratios (sSMR)
with respect to EU

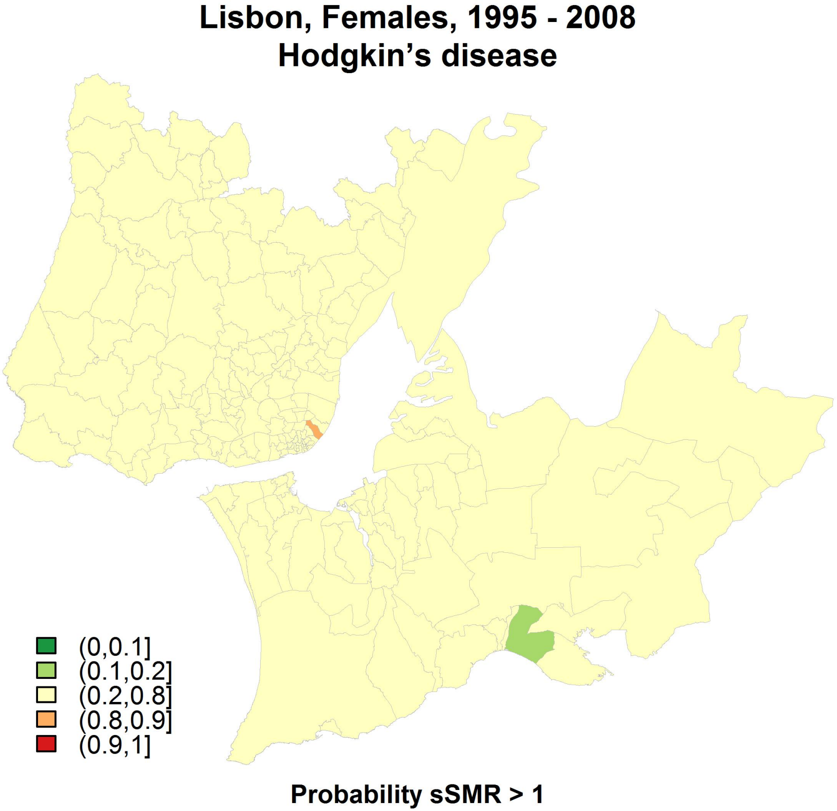
Lisbon, Females, 1995 - 2008
Hodgkin’s disease
(0,0.1]
(0.1,0.2]
(0.2,0.8]
(0.8,0.9]
(0.9,1]
Probability sSMR > 1

Lisbon, Females, 1995 - 2008
Rheumatic heart disease

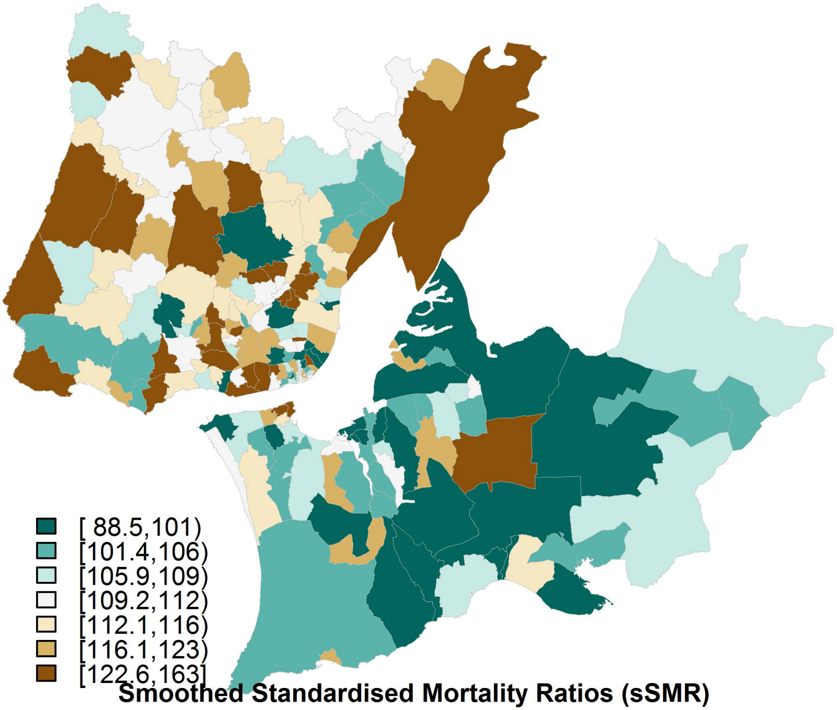

Smoothed Standardised Mortality Ratios (sSMR)
with respect to EU

Lisbon, Females, 1995 - 2008
Rheumatic heart disease

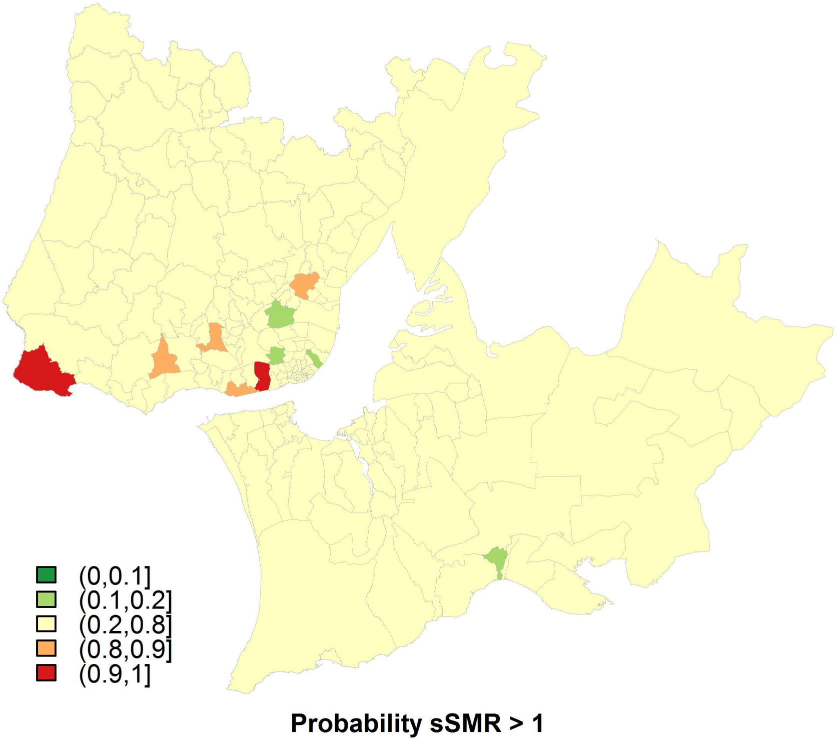

Lisbon, Females, 1995 - 2008
Hypertension

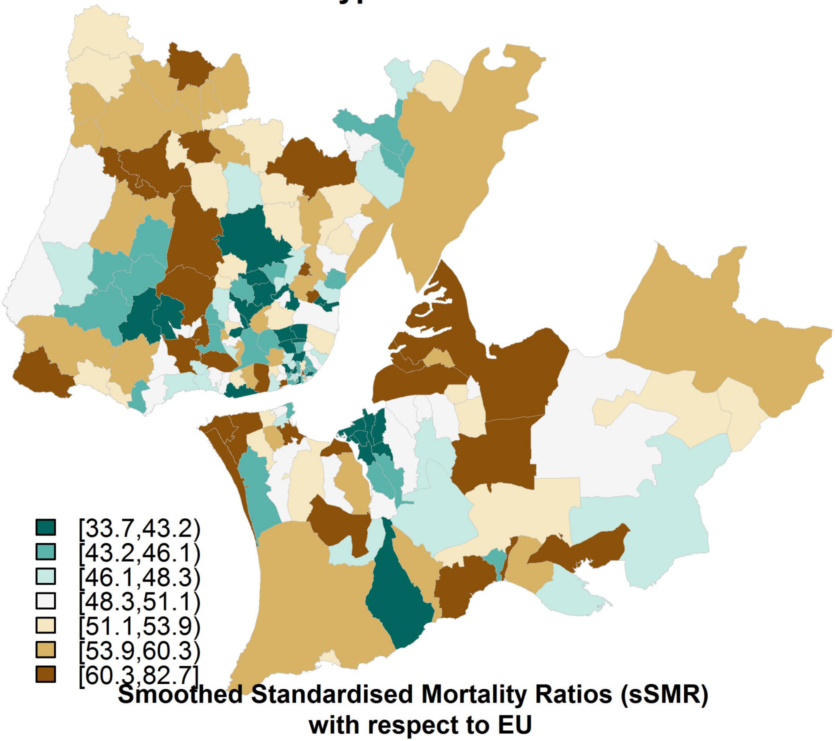

Smoothed Standardised Mortality Ratios (sSMR)
with respect to EU

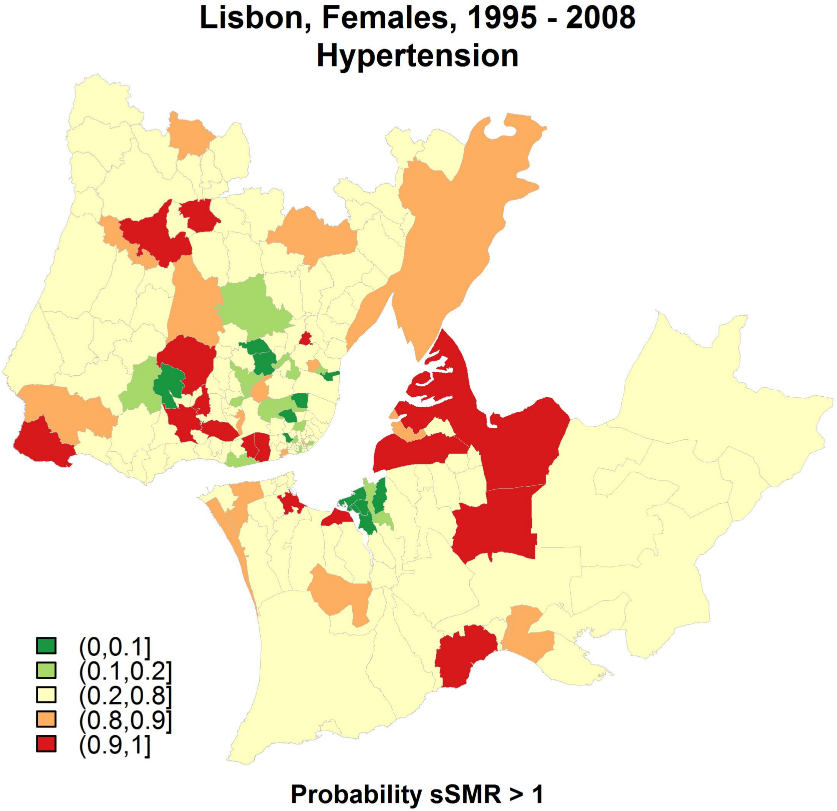
Lisbon, Females, 1995 - 2008
Hypertension
(0,0.1]
(0.1,0.2]
(0.2,0.8]
(0.8,0.9]
(0.9,1]
Probability sSMR > 1

Lisbon, Females, 1995 - 2008
Heart failure

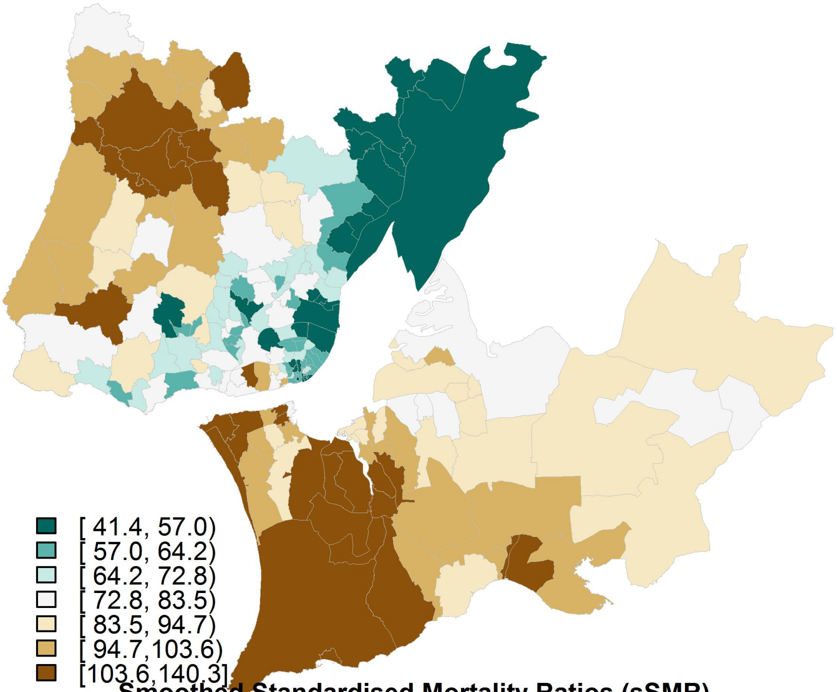

Smoothed Standardised Mortality Ratios (sSMR)
with respect to EU

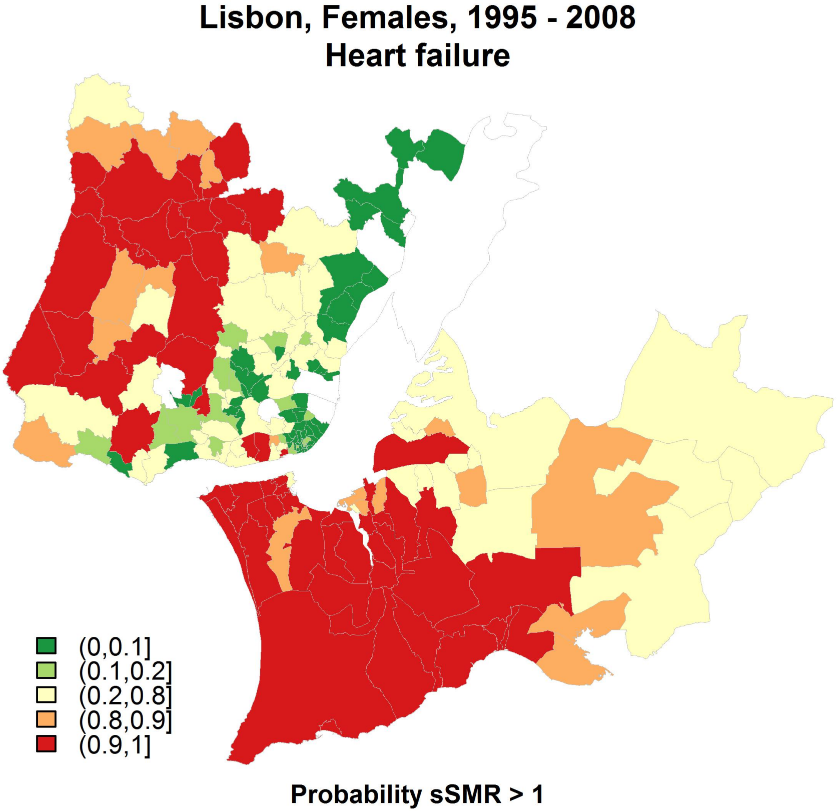
Lisbon, Females, 1995 - 2008
Heart failure
(0,0.1]
(0.1,0.2]
(0.2,0.8]
(0.8,0.9]
(0.9,1]
Probability sSMR > 1

Lisbon, Females, 1995 - 2008
Cerebrovascular diseases

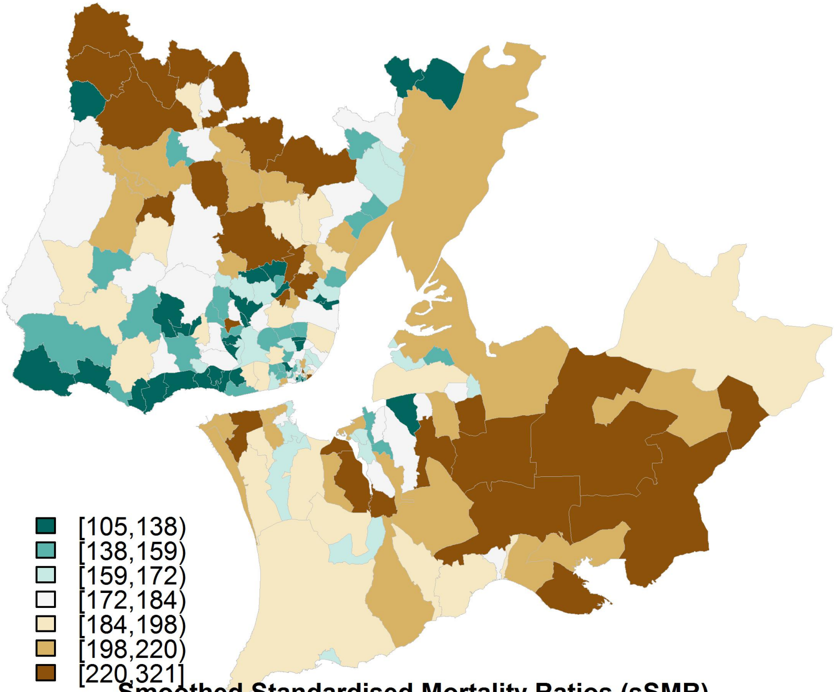

Smoothed Standardised Mortality Ratios (sSMR)
with respect to EU

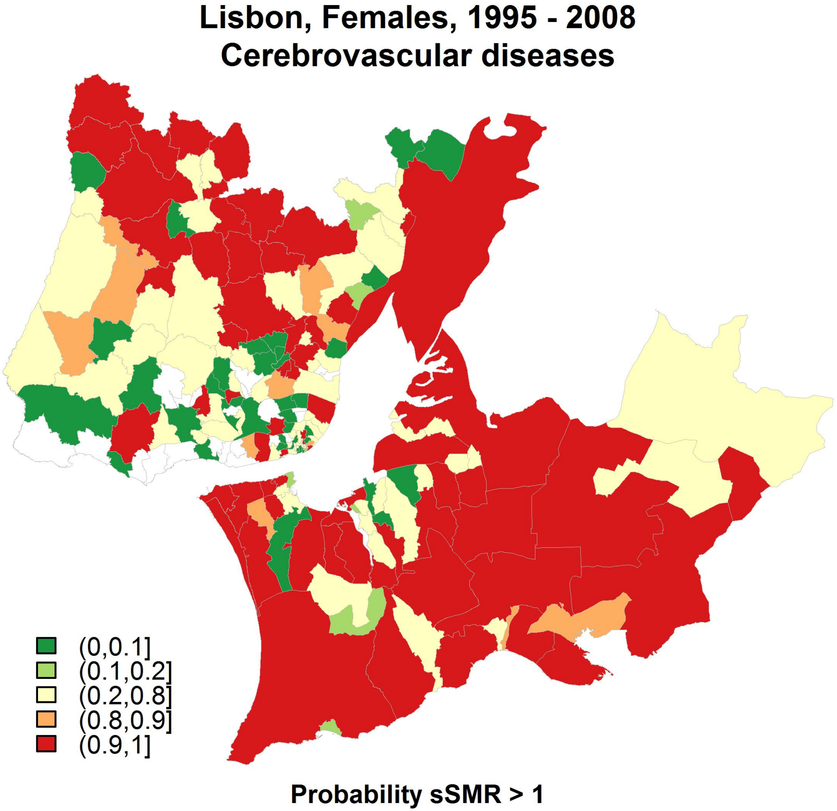

Lisbon, Females, 1995 - 2008
Cerebrovascular diseases
(0,0.1]
(0.1,0.2]
(0.2,0.8]
(0.8,0.9]
(0.9,1]
Probability sSMR > 1

Lisbon, Females, 1995 - 2008
Peptic ulcer

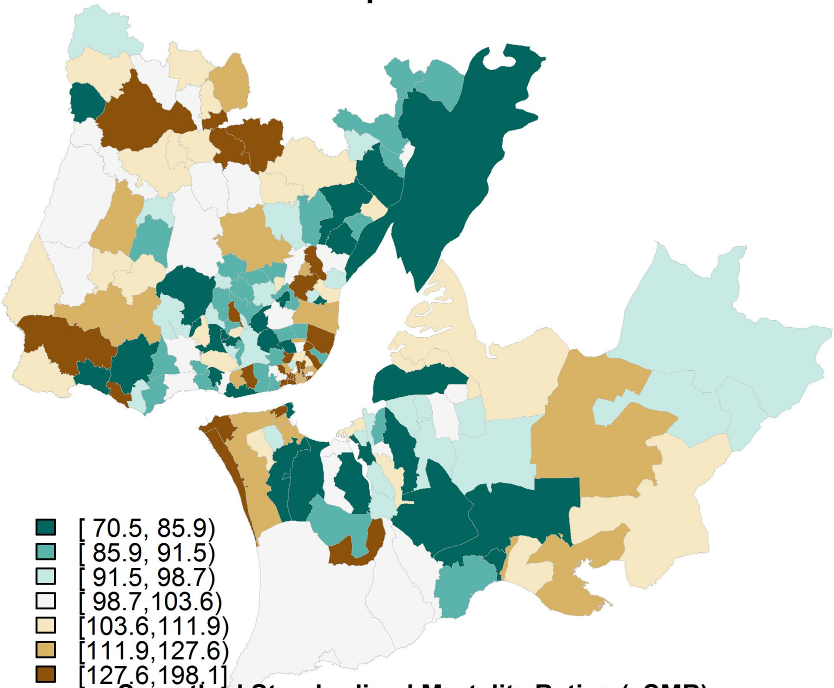

Smoothed Standardised Mortality Ratios (sSMR) with respect to EU

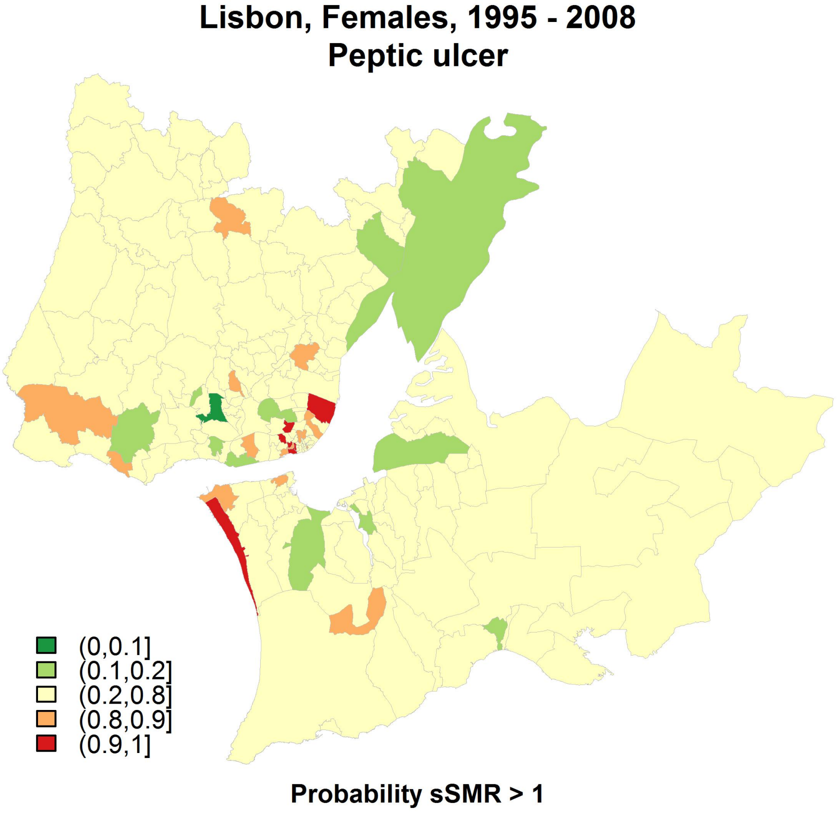
Lisbon, Females, 1995 - 2008
Peptic ulcer
(0,0.1]
(0.1,0.2]
(0.2,0.8]
(0.8,0.9]
(0.9,1]
Probability sSMR > 1

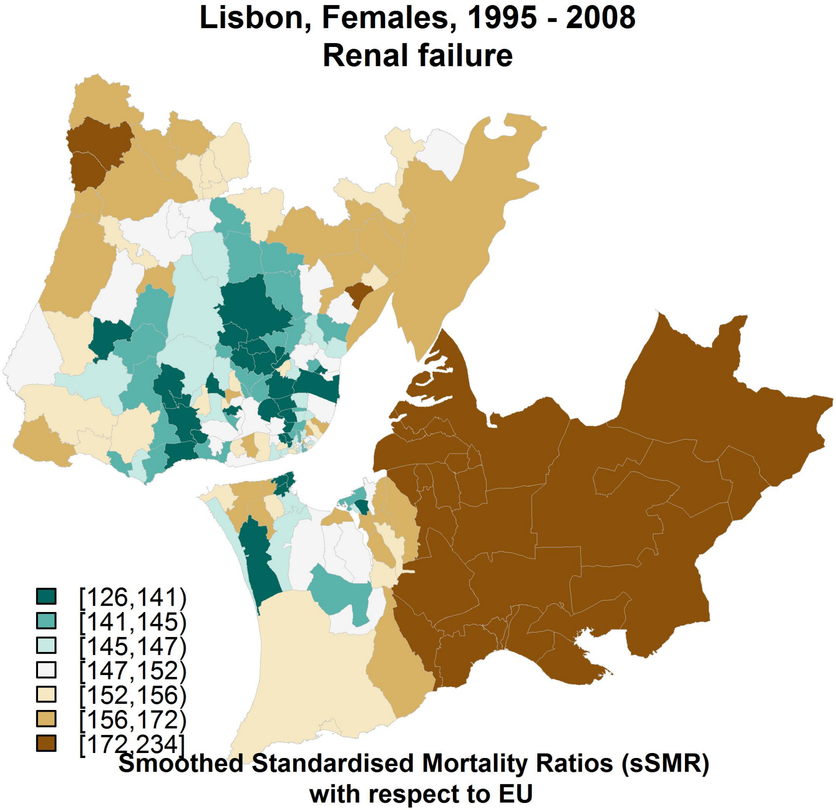
Lisbon, Females, 1995 - 2008
Renal failure
[126,141)
[141,145)
[145,147)
[147,152)
[152,156)
[156,172)
[172,234]
Smoothed Standardised Mortality Ratios (sSMR)
with respect to EU

Lisbon, Females, 1995 - 2008
Renal failure

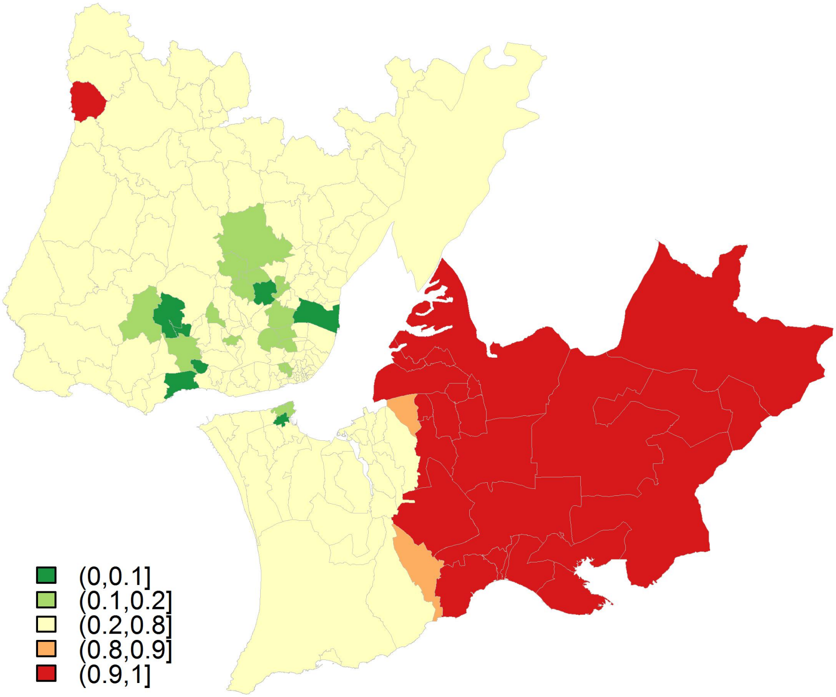

Probability sSMR > 1

Lisbon, Females, 1995 - 2008
Conditions originating in the perinatal period

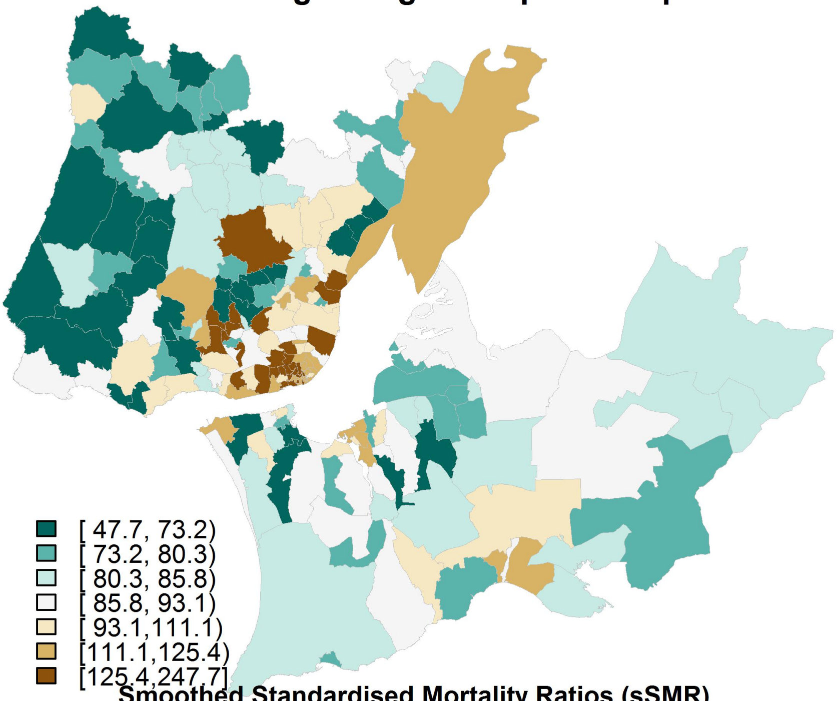

Smoothed Standardised Mortality Ratios (sSMR) with respect to EU

Lisbon, Females, 1995 - 2008
Conditions originating in the perinatal period

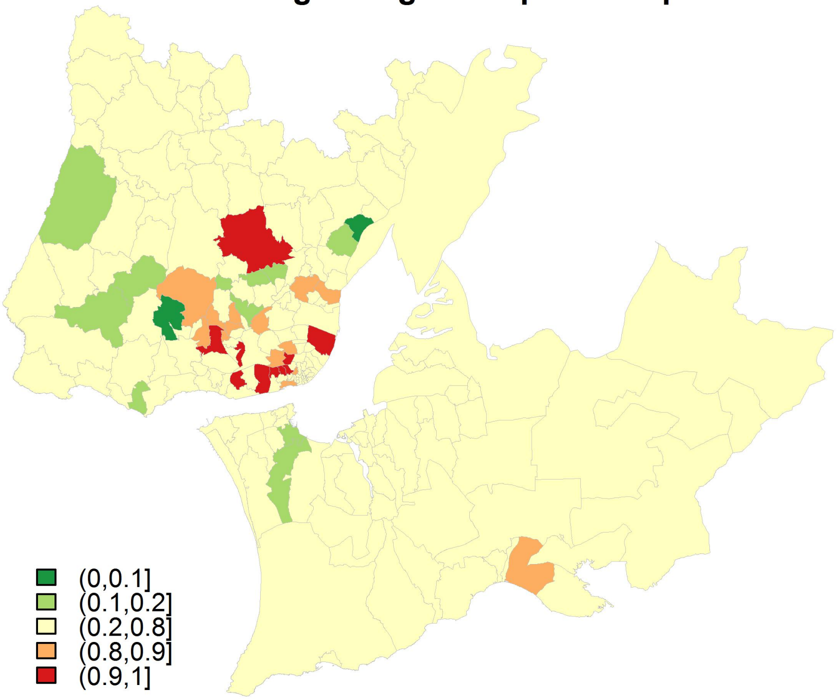

Probability sSMR > 1

Lisbon, Females, 1995 - 2008
Congenital heart disease

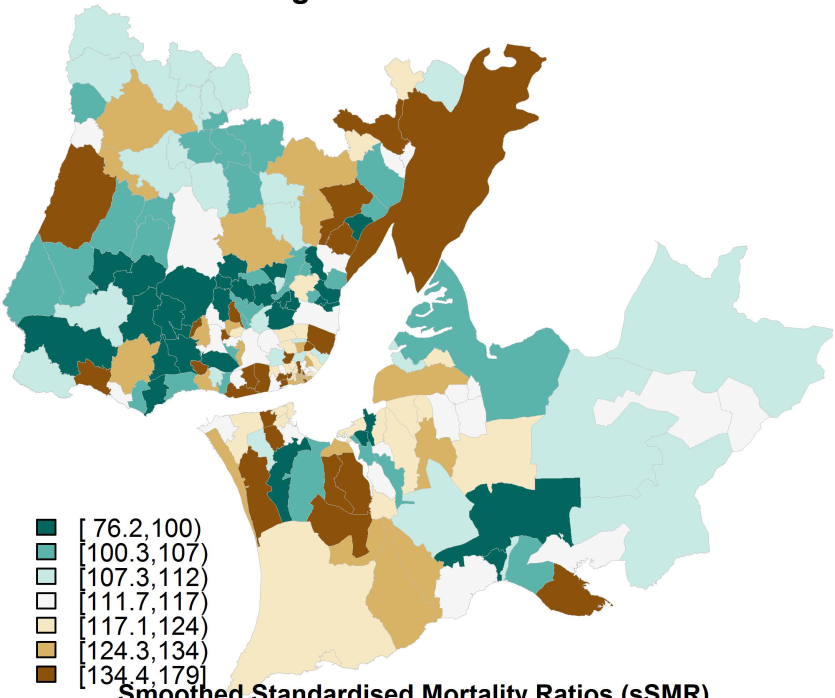

Smoothed Standardised Mortality Ratios (sSMR)
with respect to EU

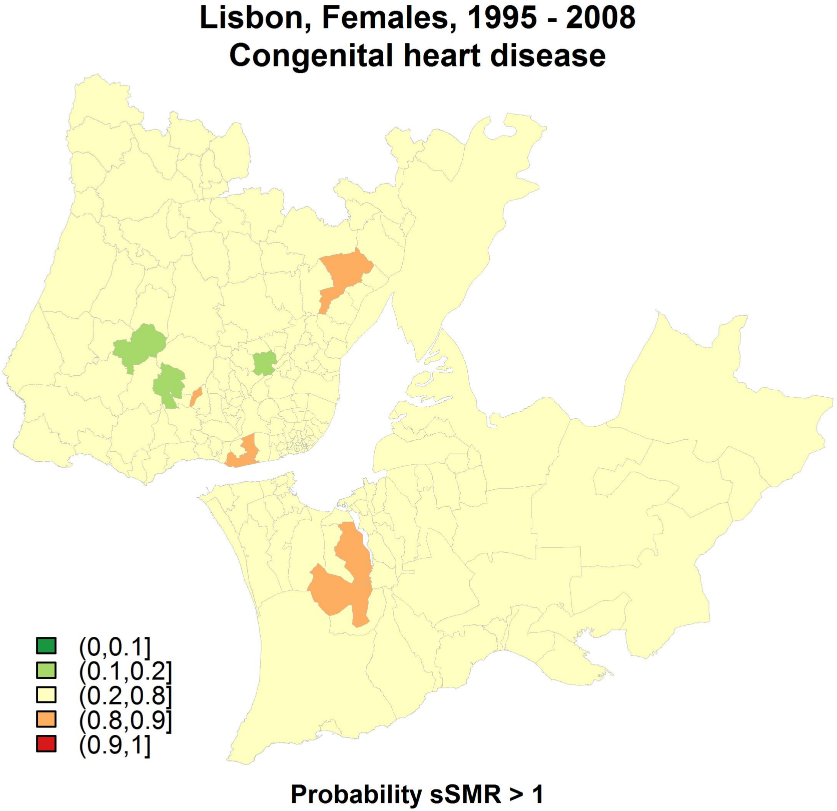
Lisbon, Females, 1995 - 2008
Congenital heart disease
(0,0.1]
(0.1,0.2]
(0.2,0.8]
(0.8,0.9]
(0.9,1]
Probability sSMR > 1
