# Supplementary material for: Social differences in avoidable mortality between small areas of 15 European cities: an ecological study
Source: Int J Health Geogr. 2014 Mar 12;13:8. doi: 10.1186/1476-072X-13-8 (PMC4007807; doi:10.1186/1476-072X-13-8)

**London, Males, 1995 - 2008**
**AIDS (HIV disease)**

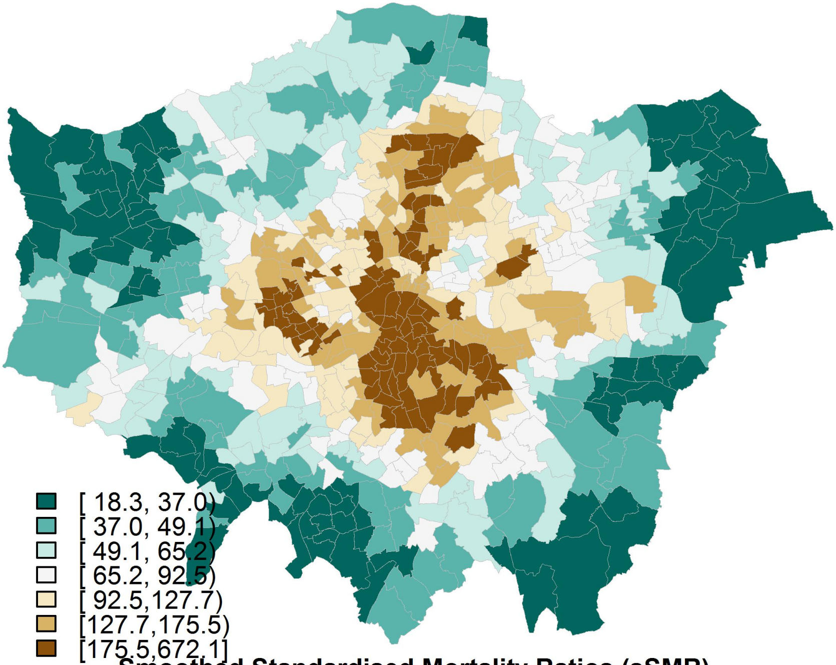


**Smoothed Standardised Mortality Ratios (sSMR)**
**with respect to EU**

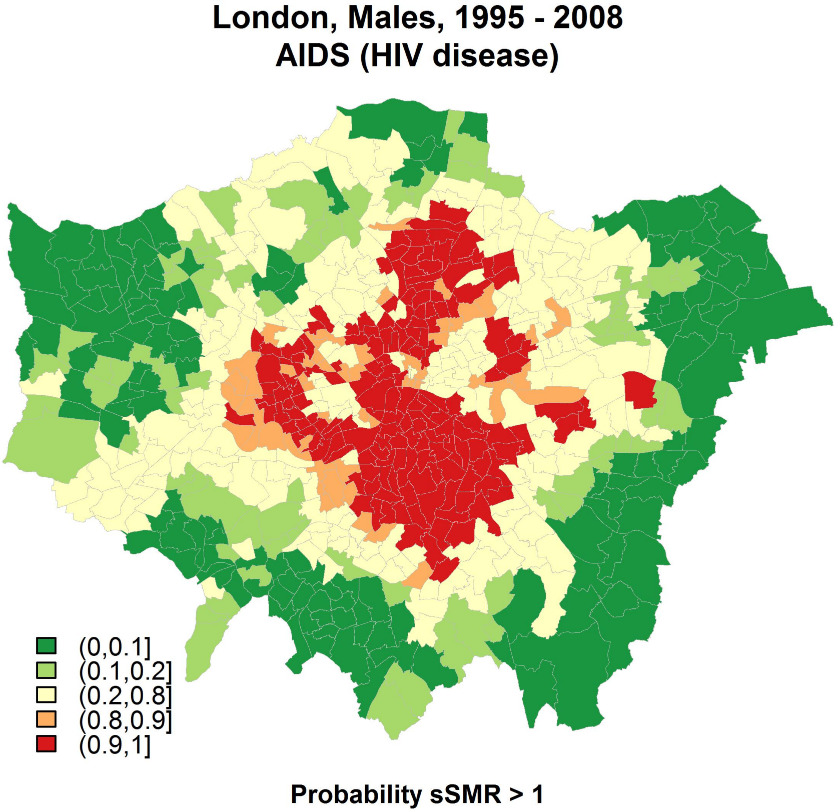
London, Males, 1995 - 2008
AIDS (HIV disease)
(0,0.1]
(0.1,0.2]
(0.2,0.8]
(0.8,0.9]
(0.9,1]
Probability sSMR > 1

**London, Males, 1995 - 2008**
**MN colon**

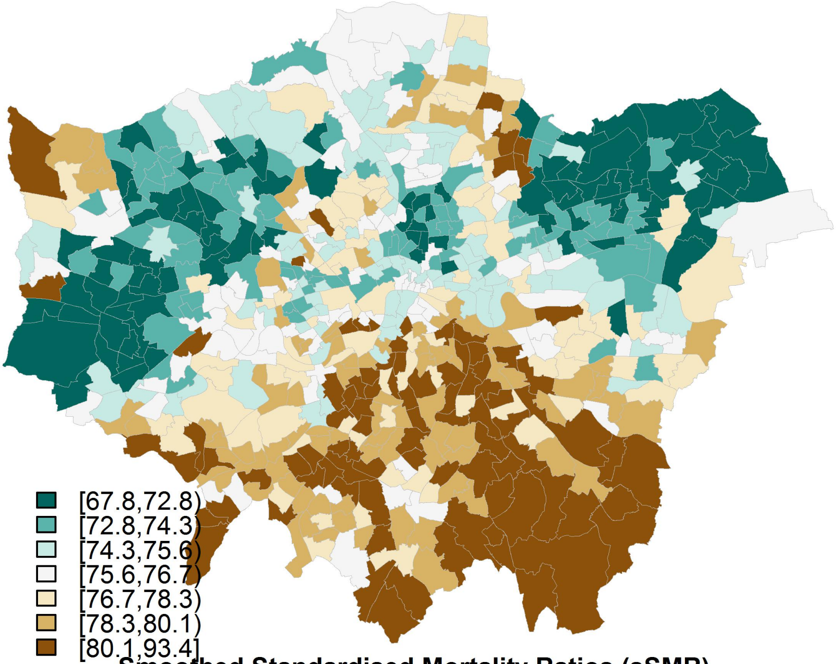


**Smoothed Standardised Mortality Ratios (sSMR)**
**with respect to EU**

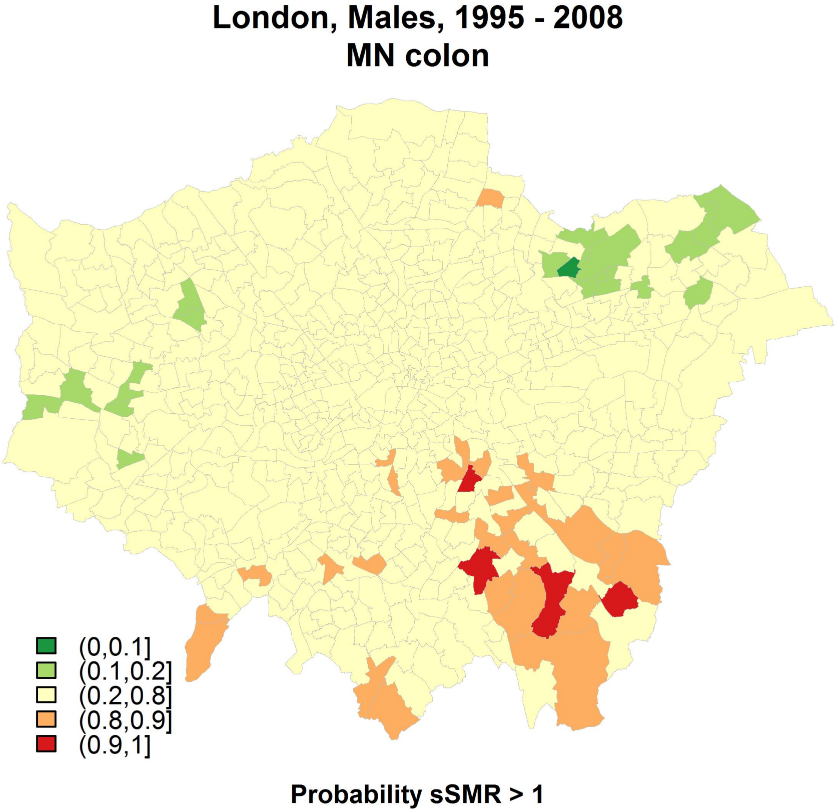
London, Males, 1995 - 2008
MN colon
(0,0.1]
(0.1,0.2]
(0.2,0.8]
(0.8,0.9]
(0.9,1]
Probability sSMR > 1

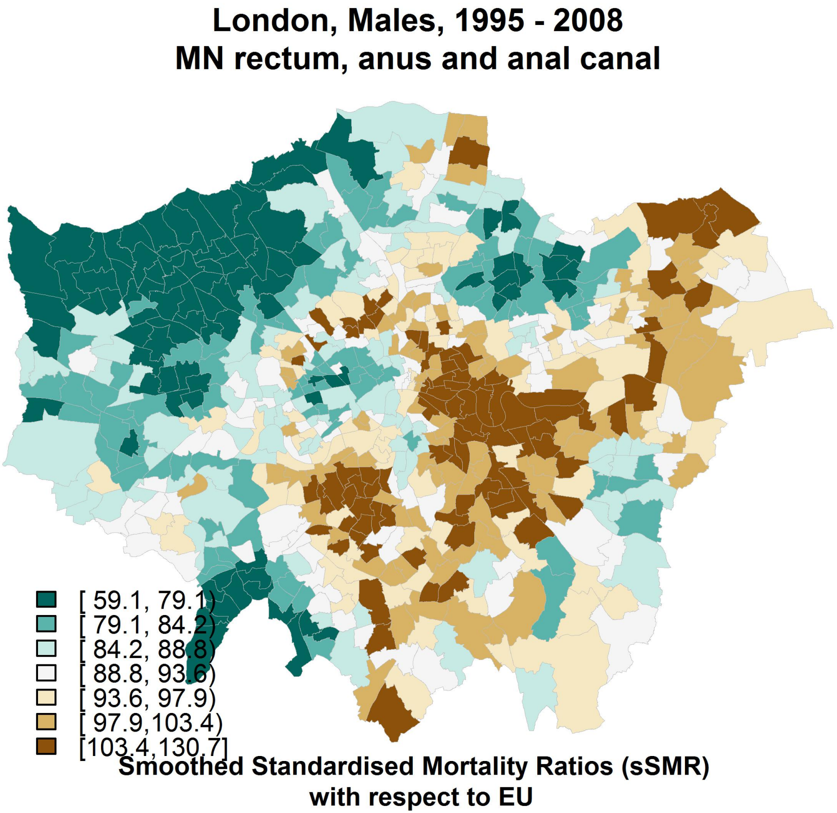
London, Males, 1995 - 2008
MN rectum, anus and anal canal
[ 59.1, 79.1)
[ 79.1, 84.2)
[ 84.2, 88.8)
[ 88.8, 93.6)
[ 93.6, 97.9)
[ 97.9,103.4)
[103.4,130.7]
Smoothed Standardised Mortality Ratios (sSMR)
with respect to EU

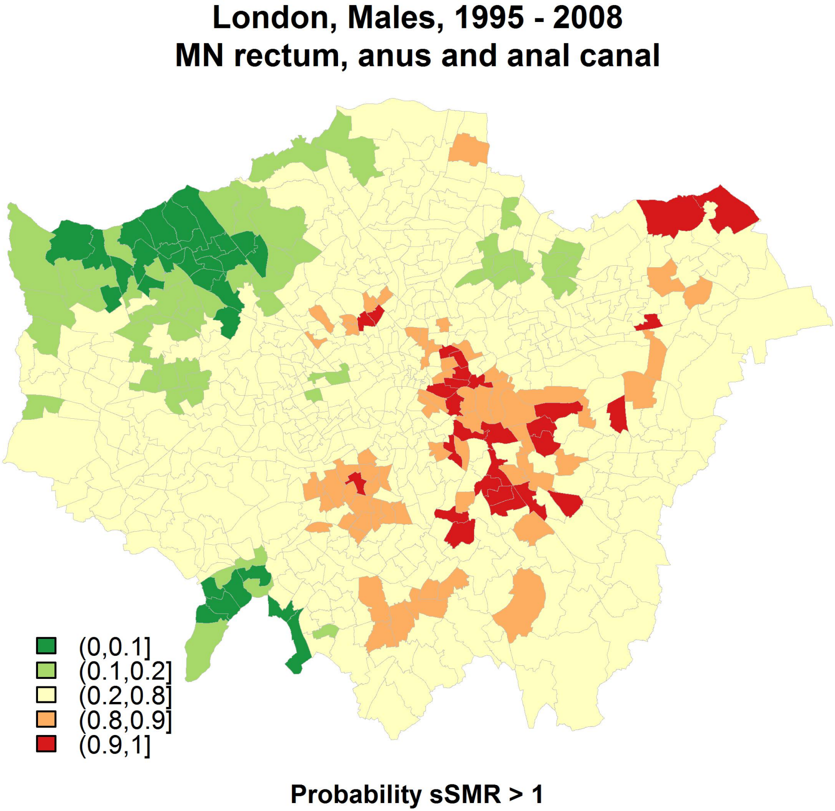
London, Males, 1995 - 2008
MN rectum, anus and anal canal
(0,0.1]
(0.1,0.2]
(0.2,0.8]
(0.8,0.9]
(0.9,1]
Probability sSMR > 1

# London, Males, 1995 - 2008
# Hodgkin's disease

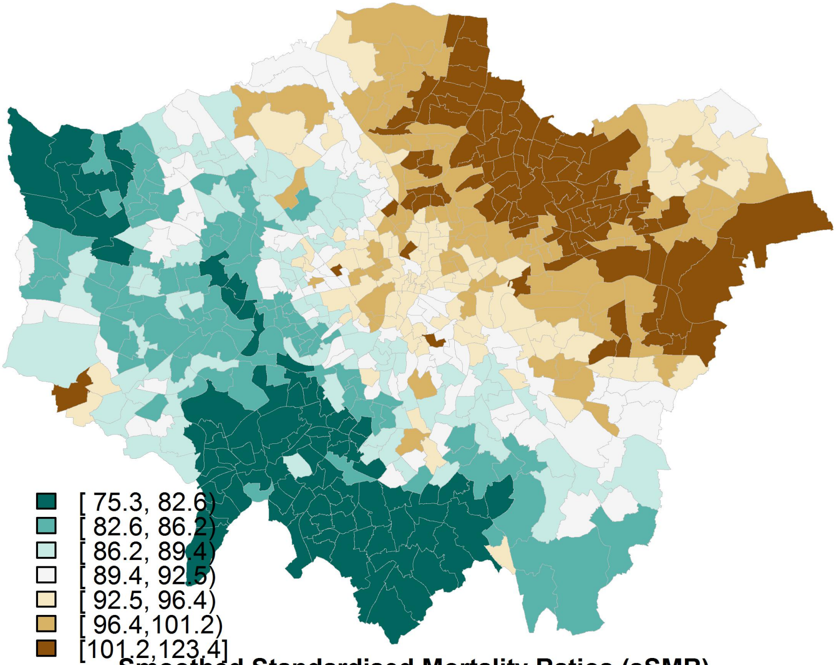


**Smoothed Standardised Mortality Ratios (sSMR) with respect to EU**

## London, Males, 1995 - 2008
## Hodgkin’s disease

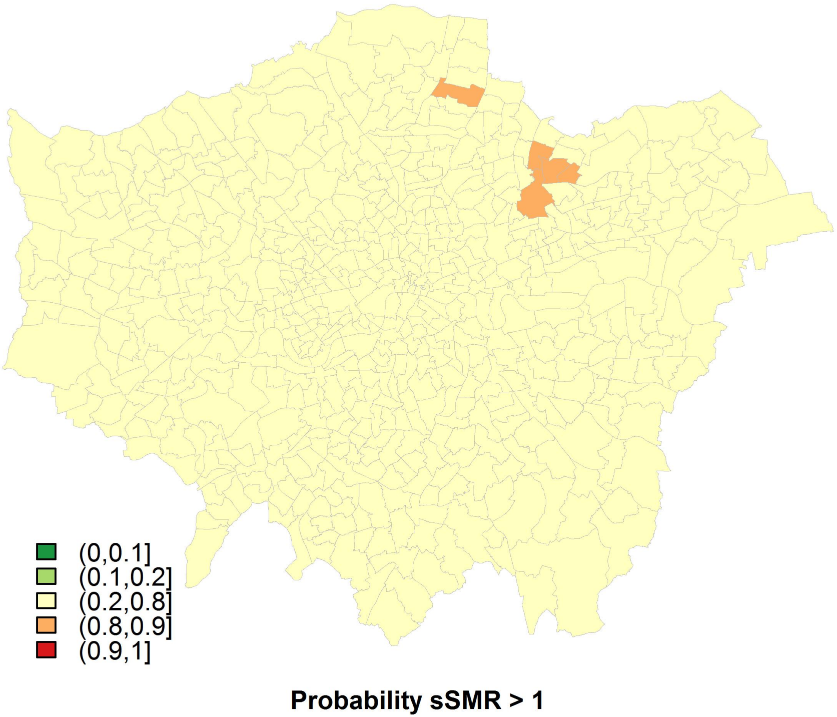

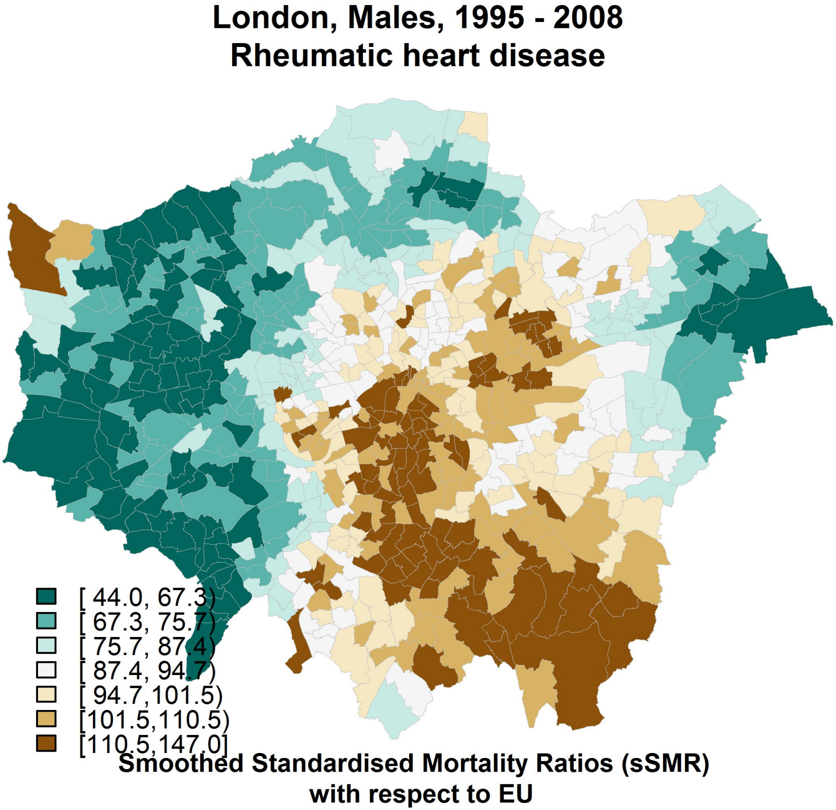
London, Males, 1995 - 2008
Rheumatic heart disease
[ 44.0, 67.3)
[ 67.3, 75.7)
[ 75.7, 87.4)
[ 87.4, 94.7)
[ 94.7,101.5)
[101.5,110.5)
[110.5,147.0]
Smoothed Standardised Mortality Ratios (sSMR)
with respect to EU

## London, Males, 1995 - 2008
## Rheumatic heart disease

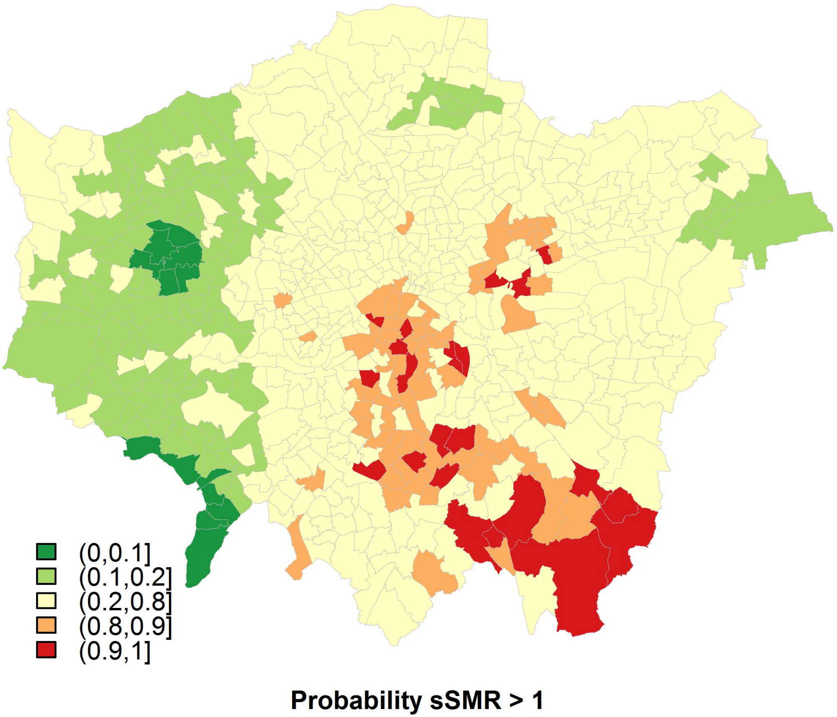

**London, Males, 1995 - 2008**
**Hypertension**

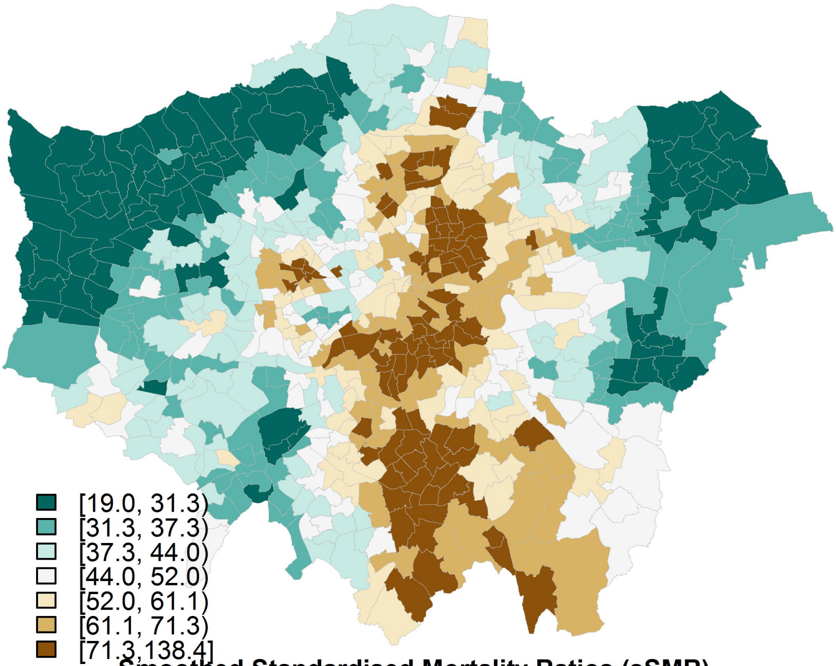


**Smoothed Standardised Mortality Ratios (sSMR)**
**with respect to EU**

## London, Males, 1995 - 2008
## Hypertension

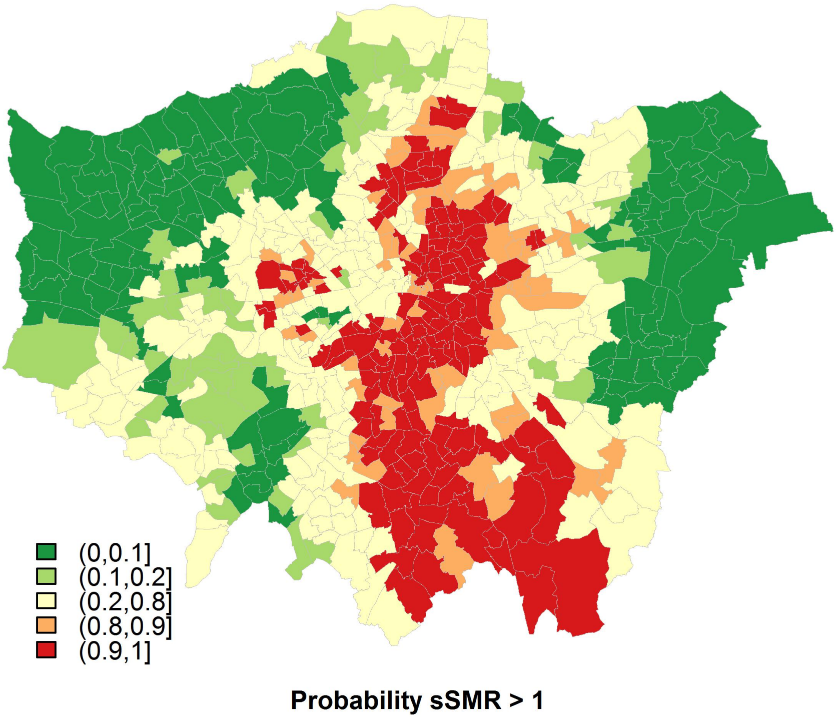

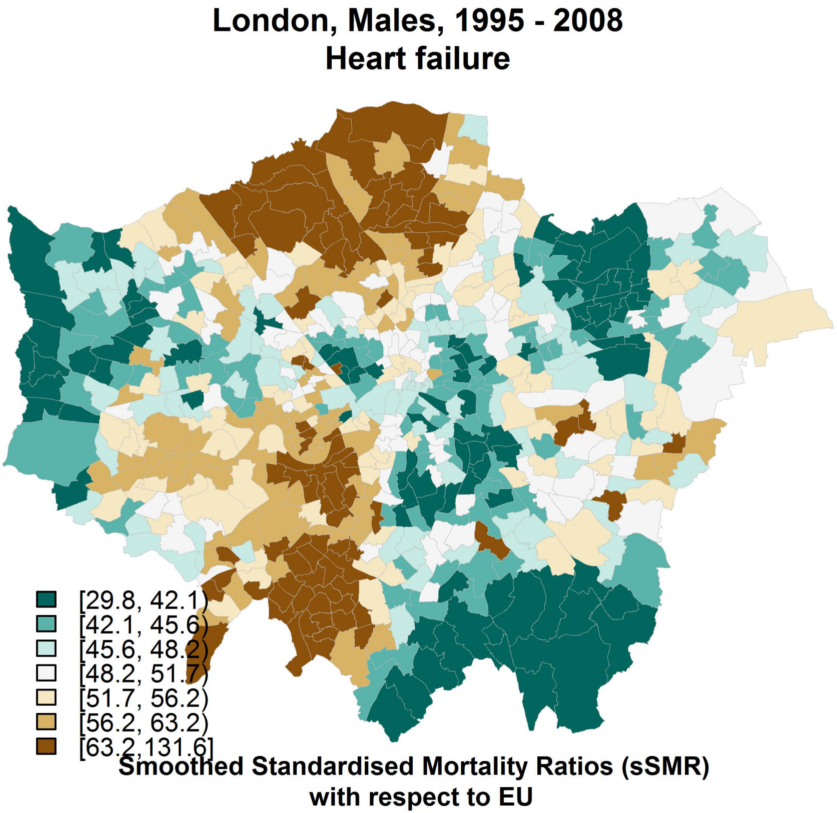
London, Males, 1995 - 2008
Heart failure
[29.8, 42.1)
[42.1, 45.6)
[45.6, 48.2)
[48.2, 51.7)
[51.7, 56.2)
[56.2, 63.2)
[63.2,131.6]
Smoothed Standardised Mortality Ratios (sSMR)
with respect to EU

# London, Males, 1995 - 2008
# Heart failure

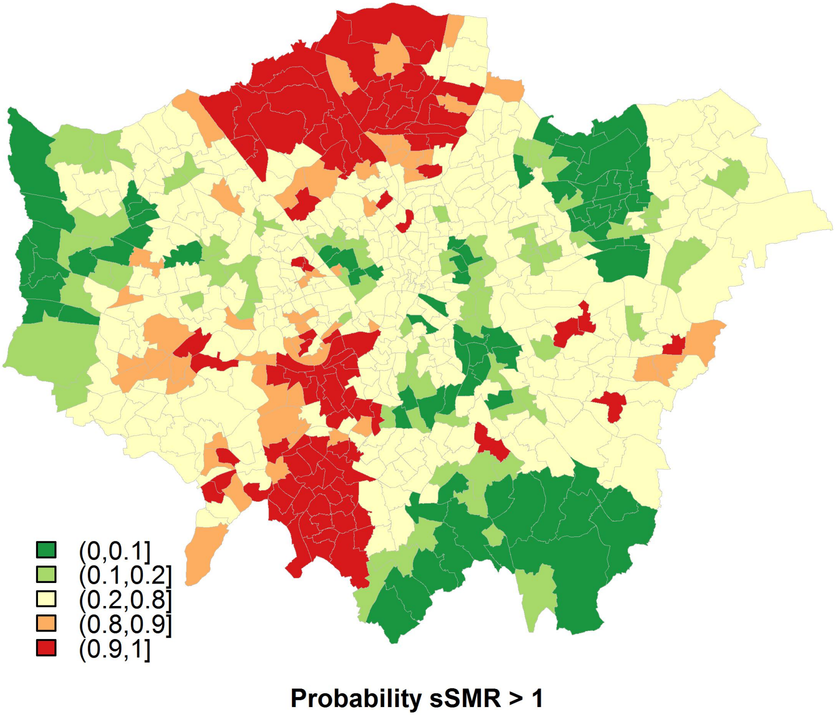

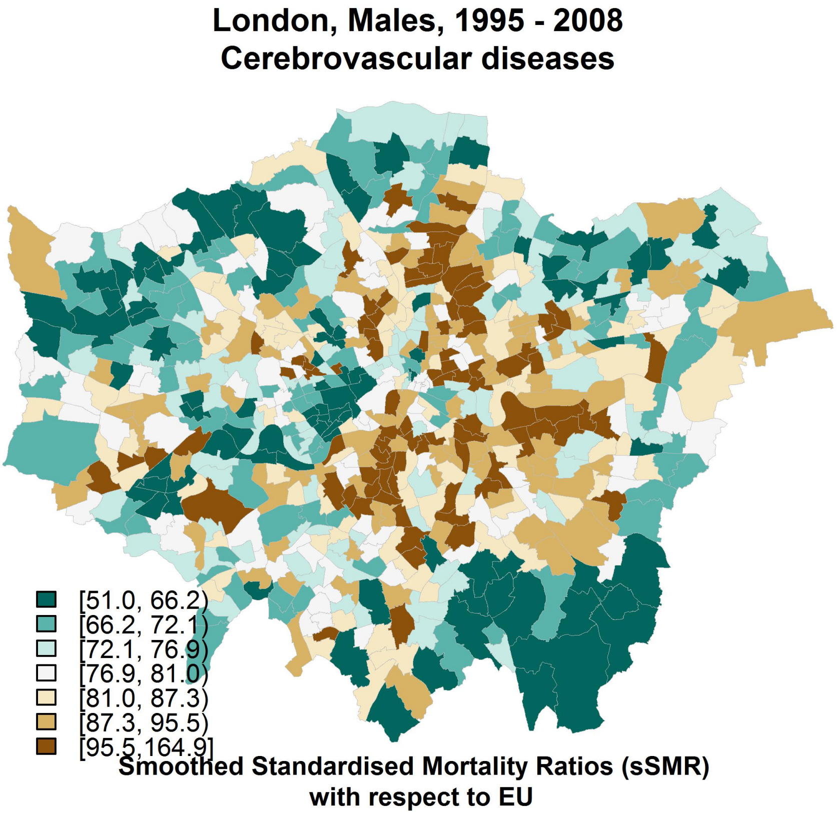
London, Males, 1995 - 2008
Cerebrovascular diseases
[51.0, 66.2)
[66.2, 72.1)
[72.1, 76.9)
[76.9, 81.0)
[81.0, 87.3)
[87.3, 95.5)
[95.5,164.9]
Smoothed Standardised Mortality Ratios (sSMR)
with respect to EU

## London, Males, 1995 - 2008
## Cerebrovascular diseases

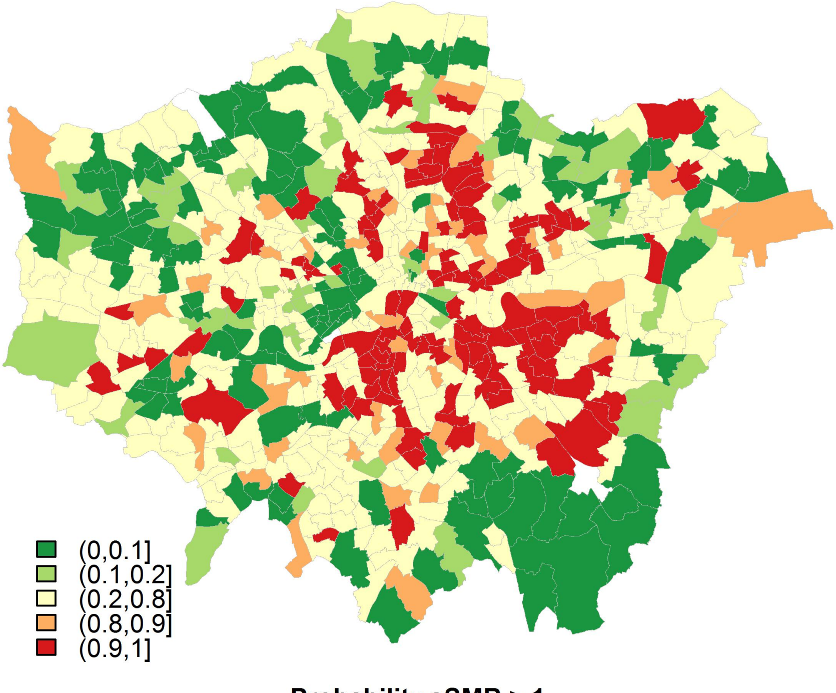

**London, Males, 1995 - 2008**
**Peptic ulcer**

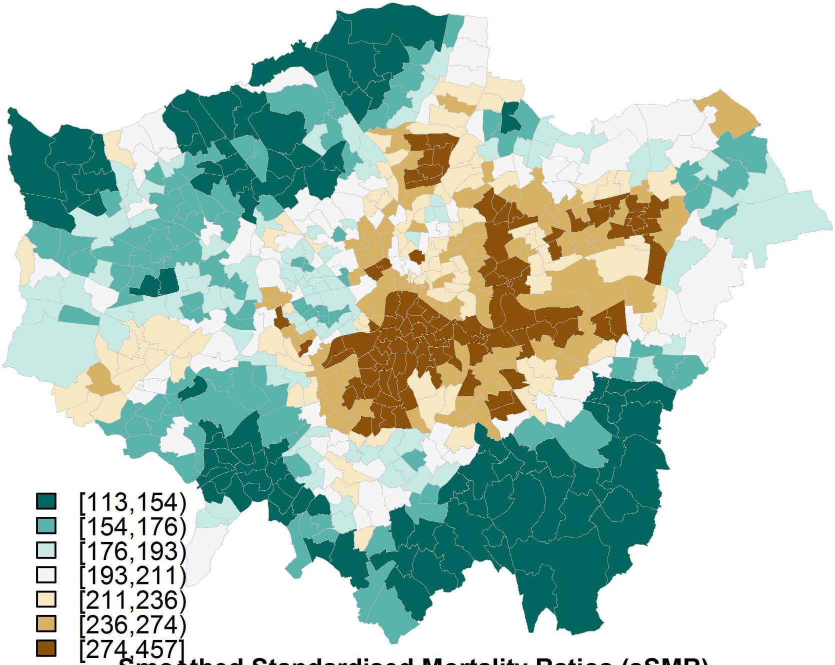


**Smoothed Standardised Mortality Ratios (sSMR)**
**with respect to EU**

## London, Males, 1995 - 2008
## Peptic ulcer

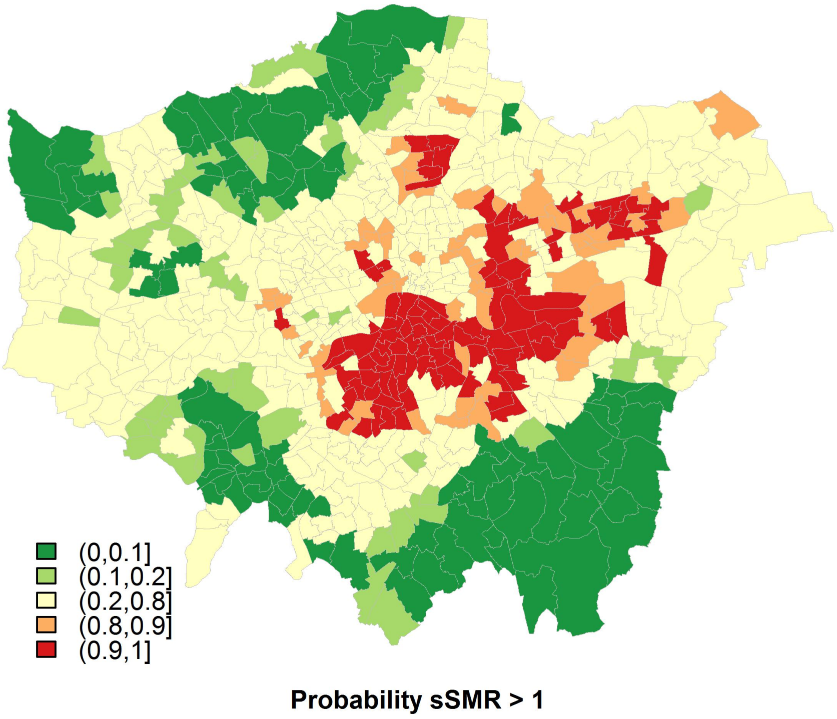

## London, Males, 1995 - 2008
## Renal failure

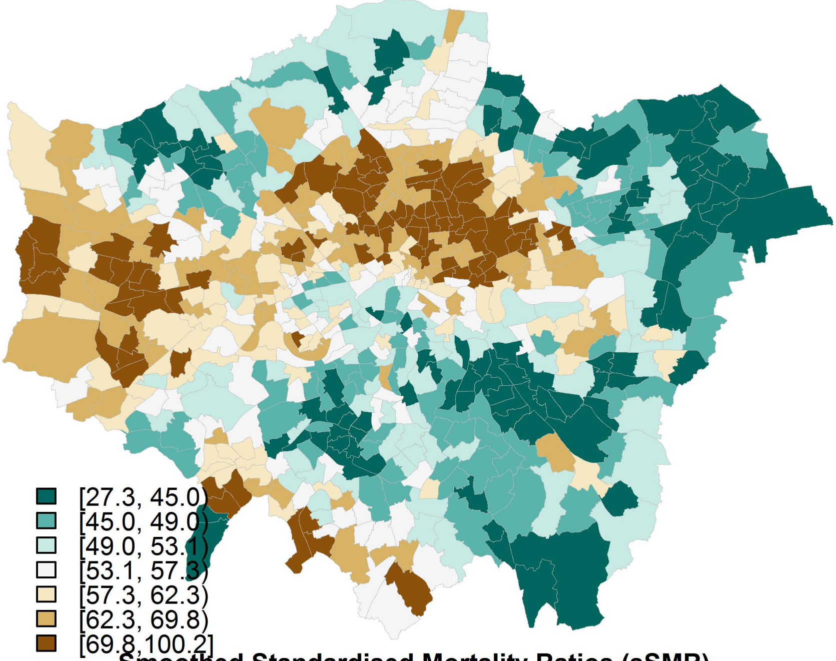


**Smoothed Standardised Mortality Ratios (sSMR)**
**with respect to EU**

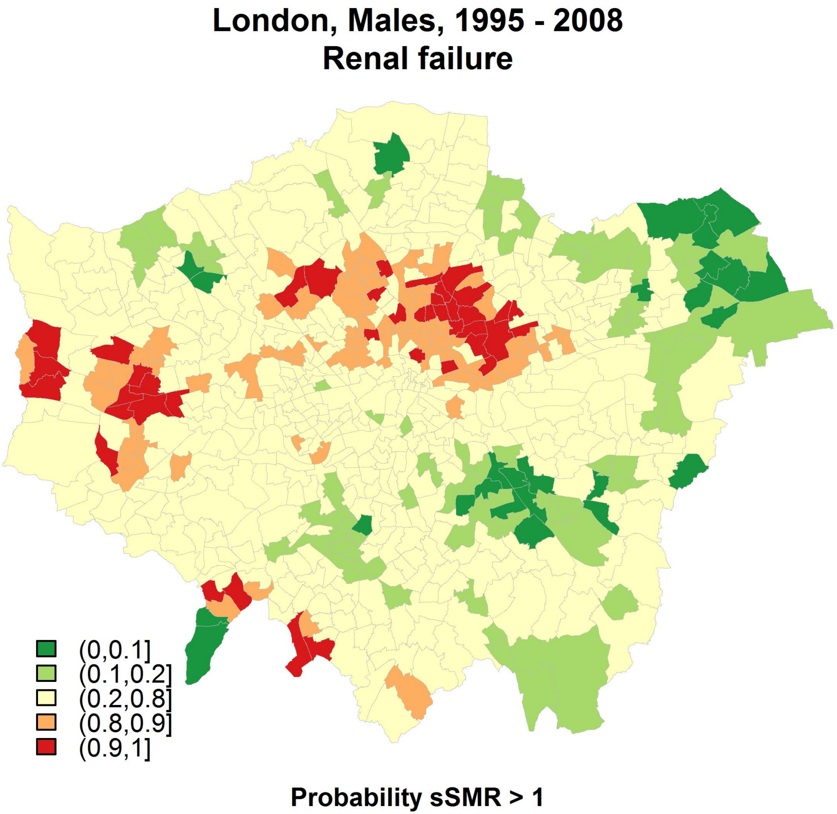
London, Males, 1995 - 2008
Renal failure
(0,0.1]
(0.1,0.2]
(0.2,0.8]
(0.8,0.9]
(0.9,1]
Probability sSMR > 1

## London, Females, 1995 - 2008
## AIDS (HIV disease)

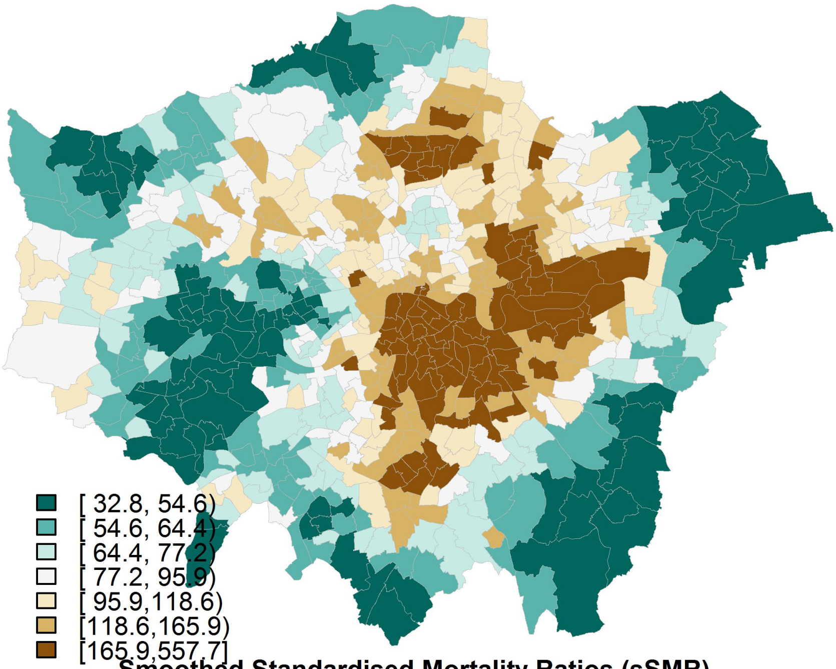


**Smoothed Standardised Mortality Ratios (sSMR)**
**with respect to EU**

**London, Females, 1995 - 2008**
**AIDS (HIV disease)**

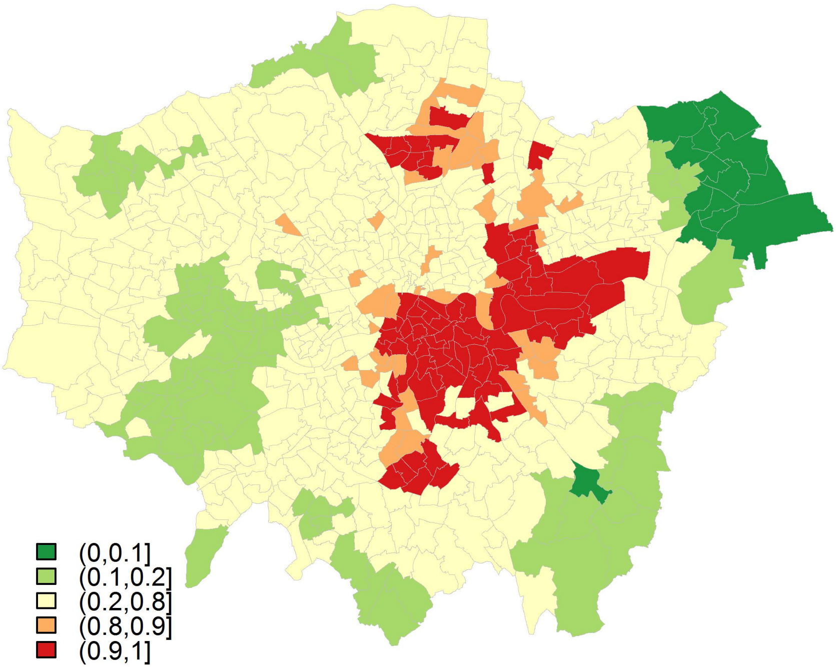


**Probability sSMR > 1**

## London, Females, 1995 - 2008
## MN colon

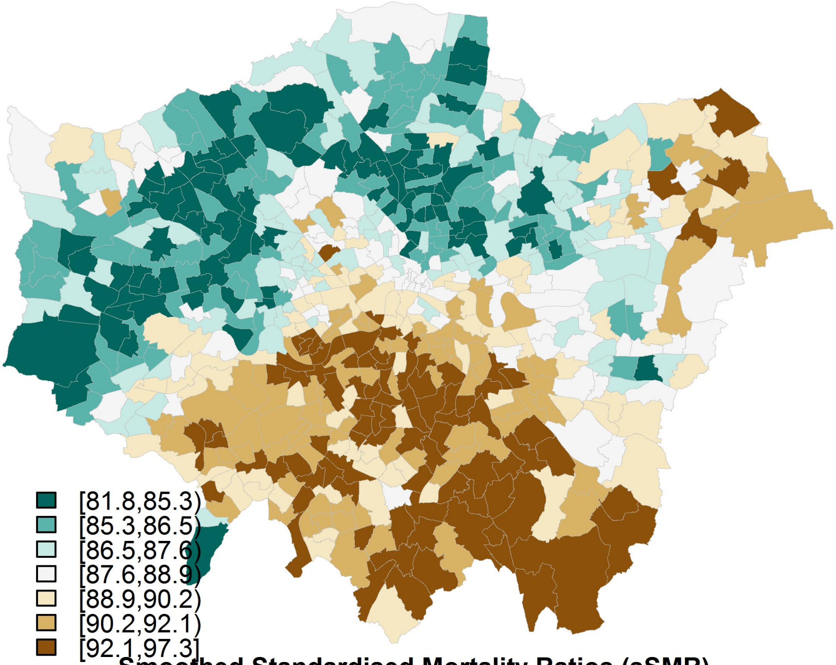


**Smoothed Standardised Mortality Ratios (sSMR)**
**with respect to EU**

## London, Females, 1995 - 2008
## MN colon

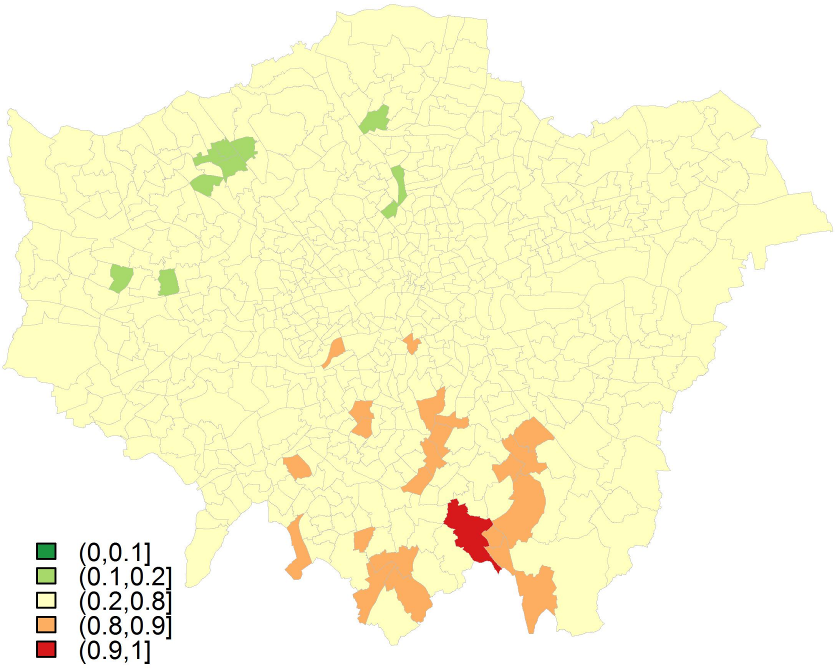

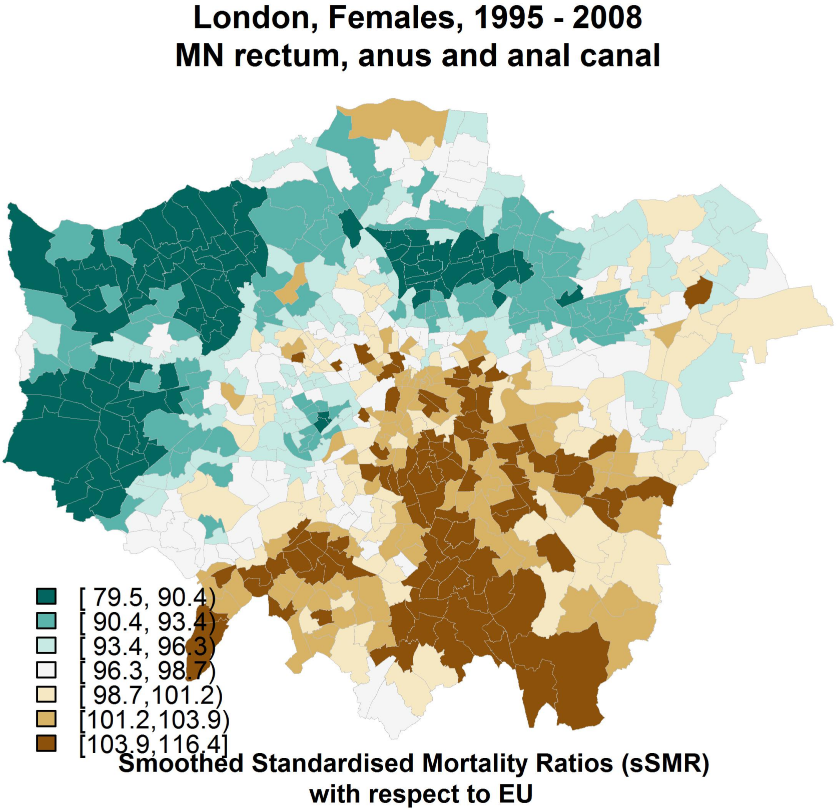
London, Females, 1995 - 2008
MN rectum, anus and anal canal
[ 79.5, 90.4)
[ 90.4, 93.4)
[ 93.4, 96.3)
[ 96.3, 98.7)
[ 98.7,101.2)
[101.2,103.9)
[103.9,116.4]
Smoothed Standardised Mortality Ratios (sSMR)
with respect to EU

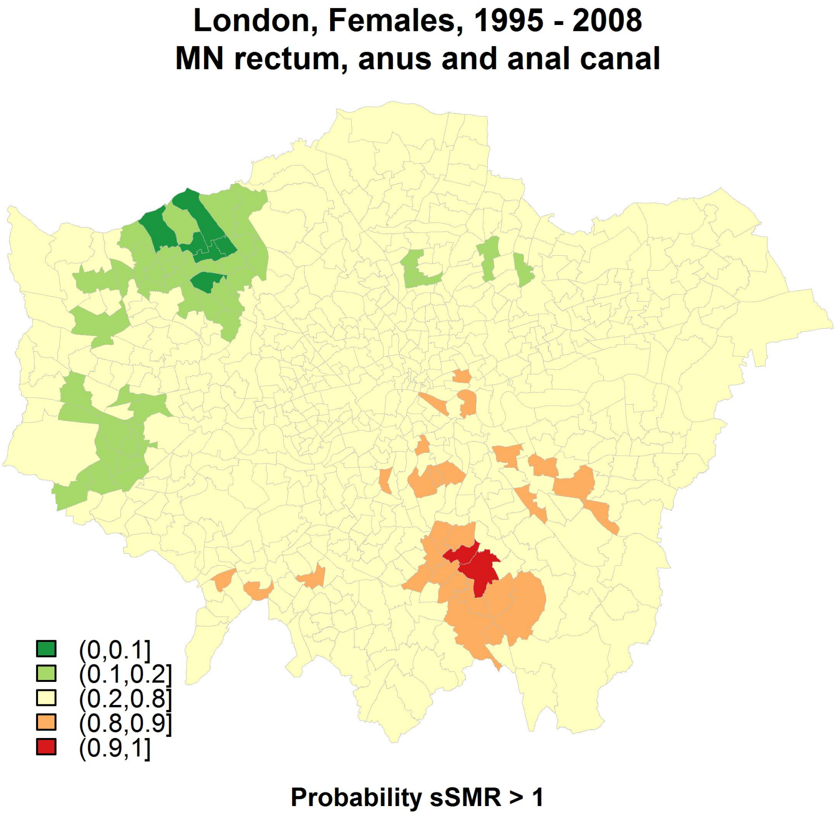
London, Females, 1995 - 2008
MN rectum, anus and anal canal
(0,0.1]
(0.1,0.2]
(0.2,0.8]
(0.8,0.9]
(0.9,1]
Probability sSMR > 1

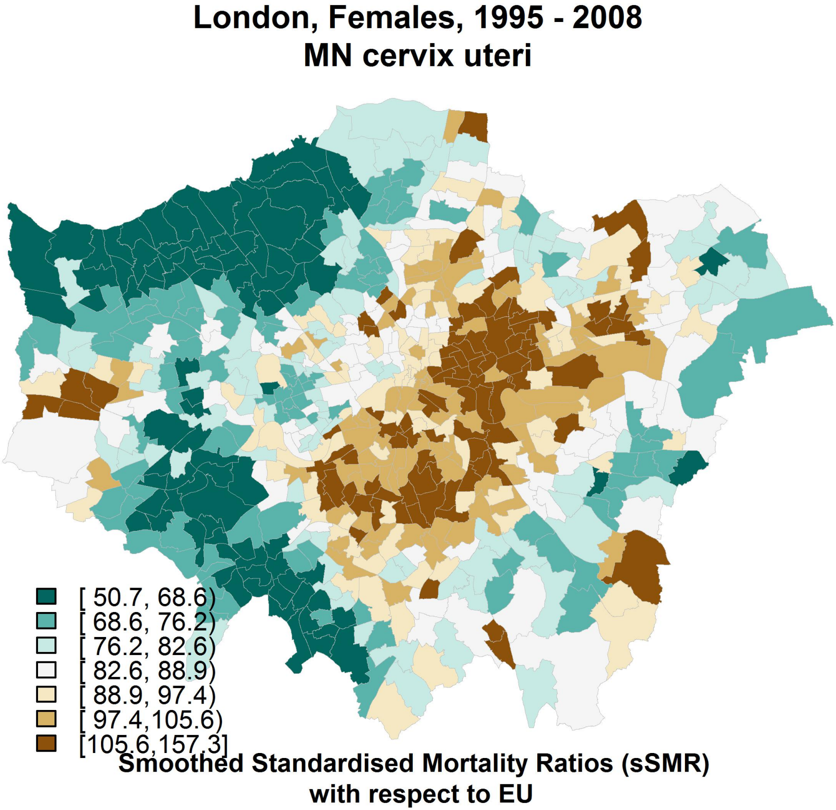
London, Females, 1995 - 2008
MN cervix uteri
[ 50.7, 68.6)
[ 68.6, 76.2)
[ 76.2, 82.6)
[ 82.6, 88.9)
[ 88.9, 97.4)
[ 97.4,105.6)
[105.6,157.3]
Smoothed Standardised Mortality Ratios (sSMR)
with respect to EU

## London, Females, 1995 - 2008
## MN cervix uteri

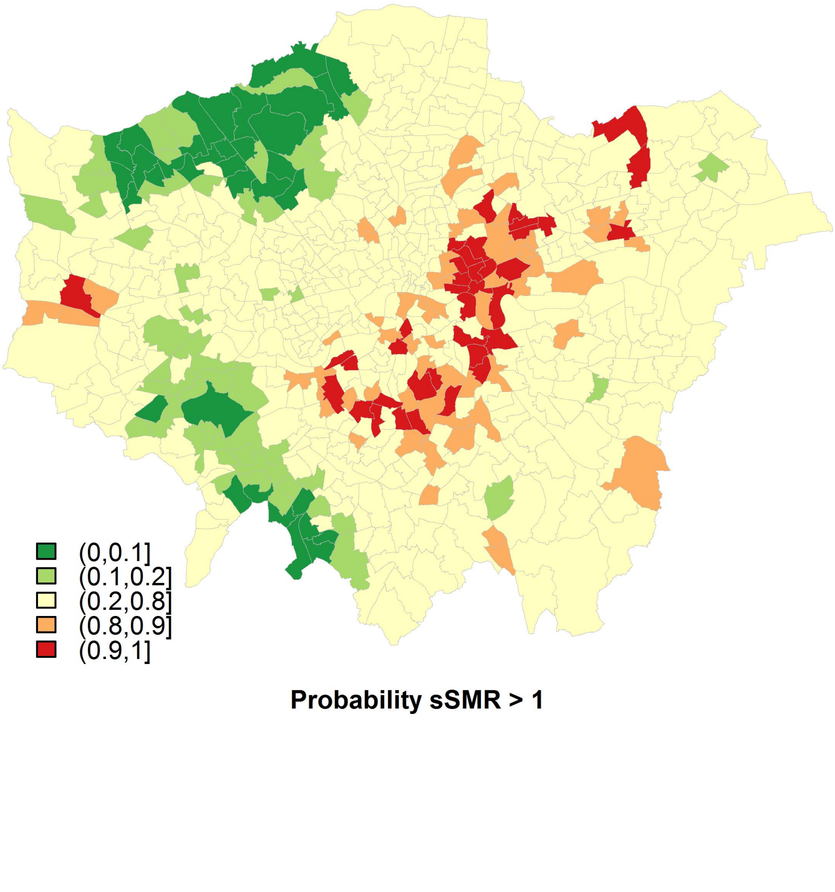

# London, Females, 1995 - 2008
## Rheumatic heart disease

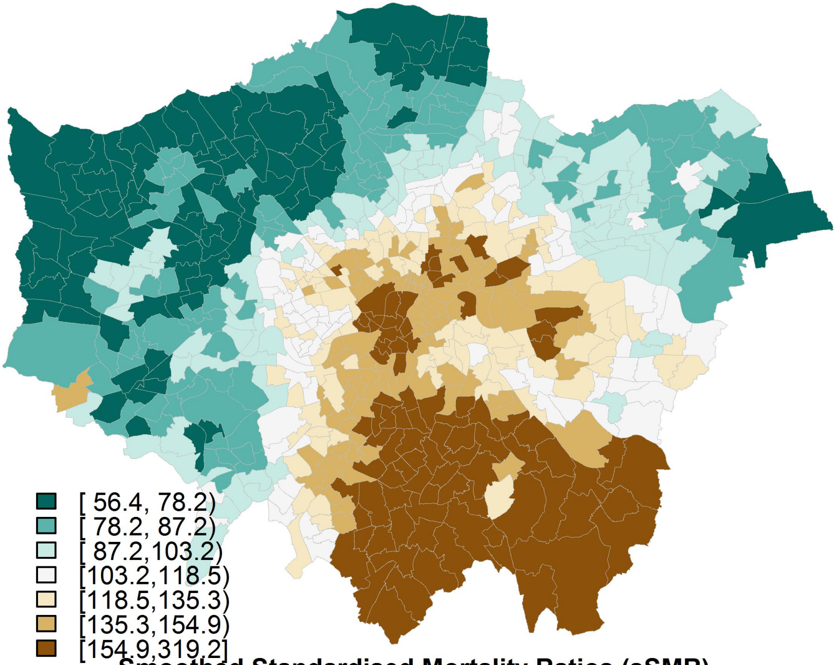


Smoothed Standardised Mortality Ratios (sSMR)
with respect to EU

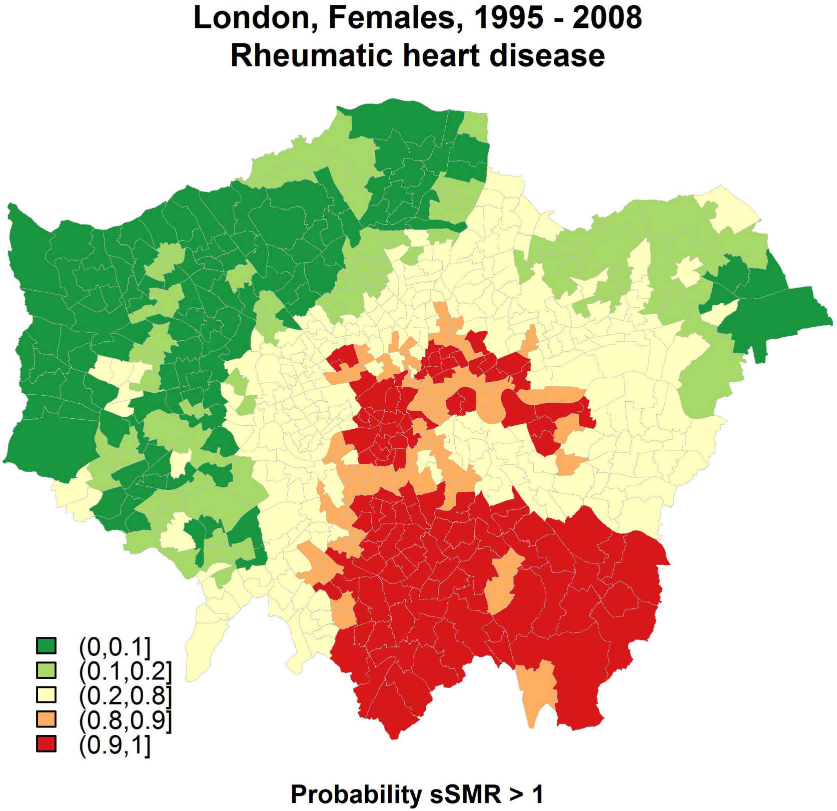
London, Females, 1995 - 2008
Rheumatic heart disease
(0,0.1]
(0.1,0.2]
(0.2,0.8]
(0.8,0.9]
(0.9,1]
Probability sSMR > 1

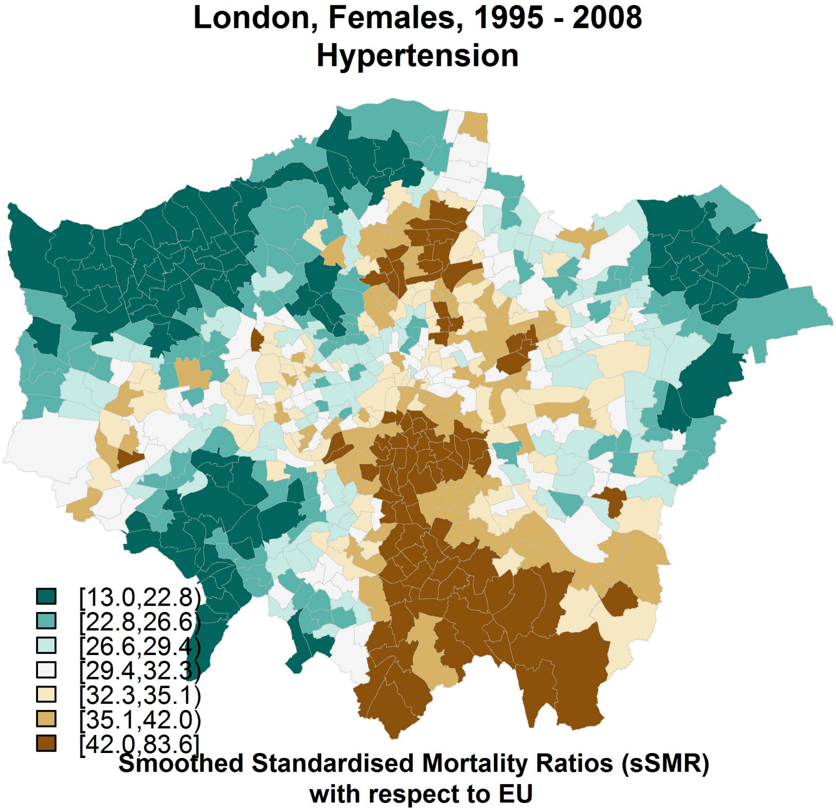
London, Females, 1995 - 2008
Hypertension
[13.0,22.8)
[22.8,26.6)
[26.6,29.4)
[29.4,32.3)
[32.3,35.1)
[35.1,42.0)
[42.0,83.6]
Smoothed Standardised Mortality Ratios (sSMR)
with respect to EU

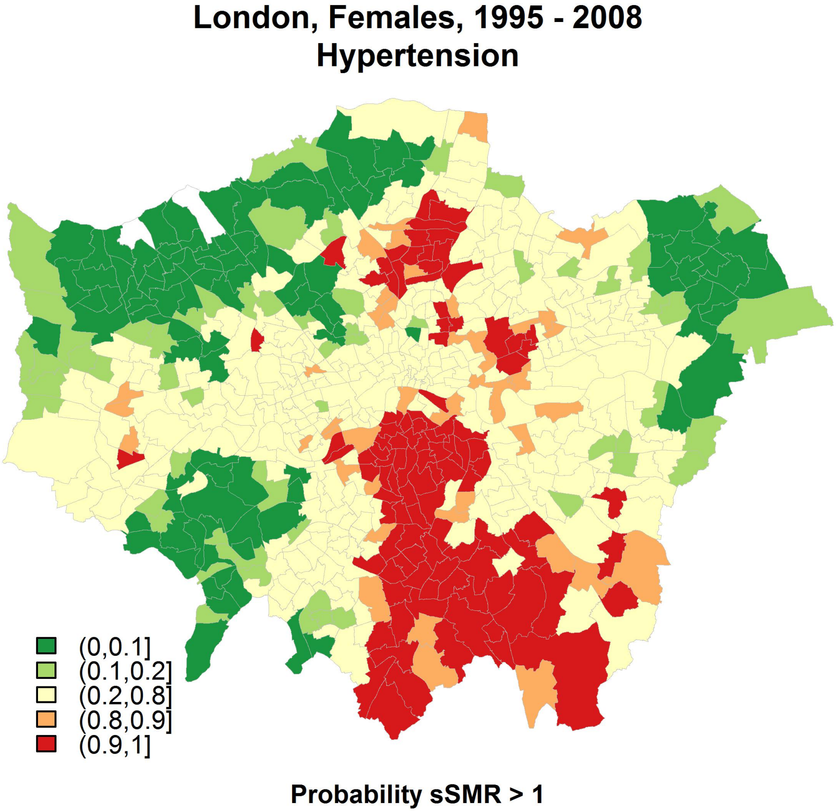
London, Females, 1995 - 2008
Hypertension
(0,0.1]
(0.1,0.2]
(0.2,0.8]
(0.8,0.9]
(0.9,1]
Probability sSMR > 1

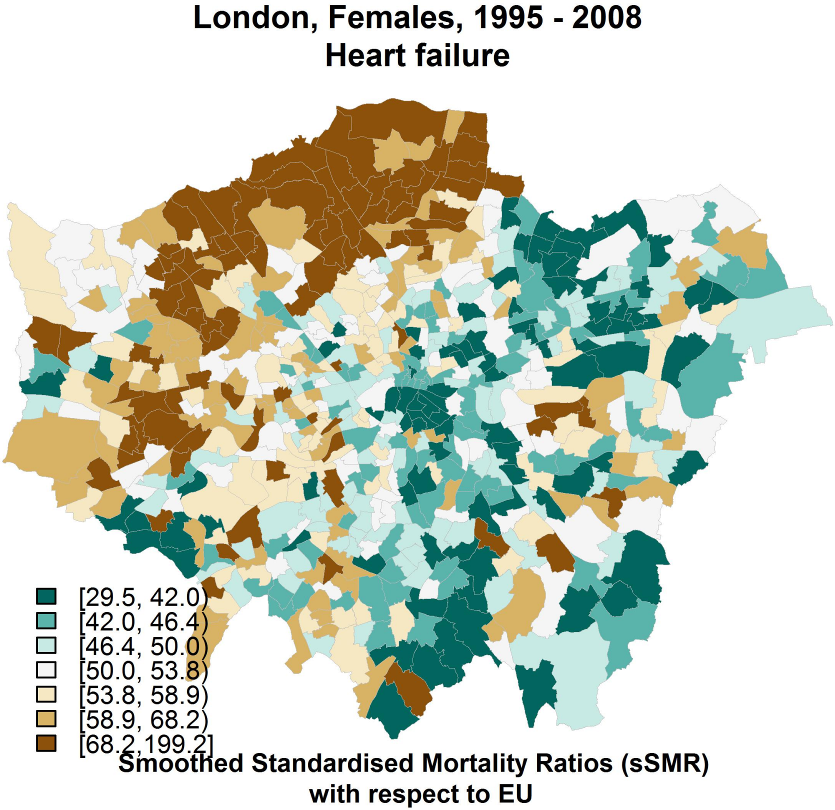
London, Females, 1995 - 2008
Heart failure
[29.5, 42.0)
[42.0, 46.4)
[46.4, 50.0)
[50.0, 53.8)
[53.8, 58.9)
[58.9, 68.2)
[68.2,199.2]
Smoothed Standardised Mortality Ratios (sSMR)
with respect to EU

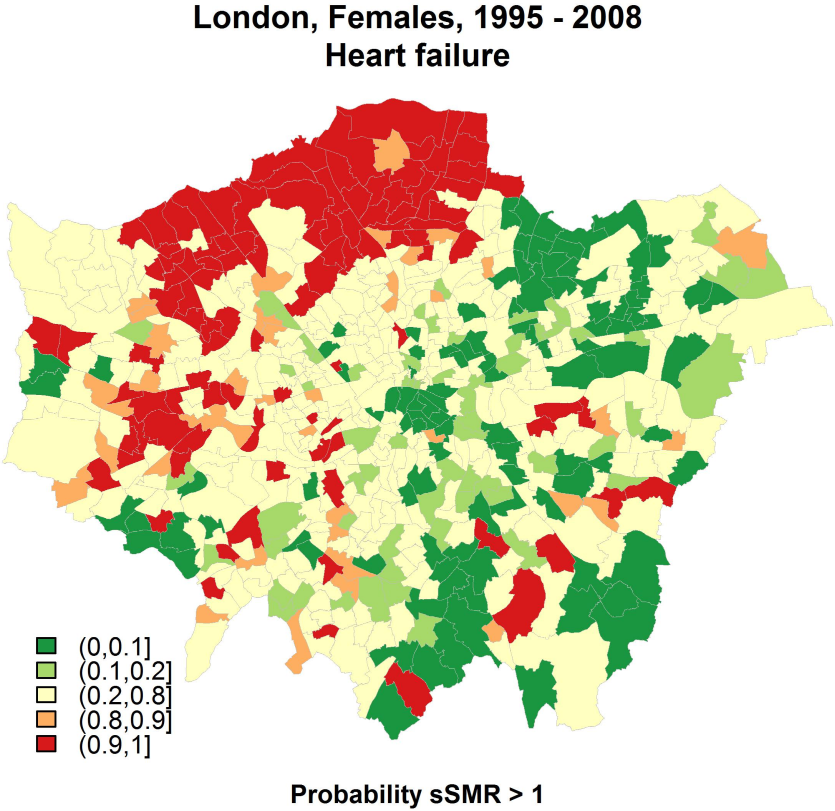
London, Females, 1995 - 2008
Heart failure
(0,0.1]
(0.1,0.2]
(0.2,0.8]
(0.8,0.9]
(0.9,1]
Probability sSMR > 1

**London, Females, 1995 - 2008**
**Cerebrovascular diseases**

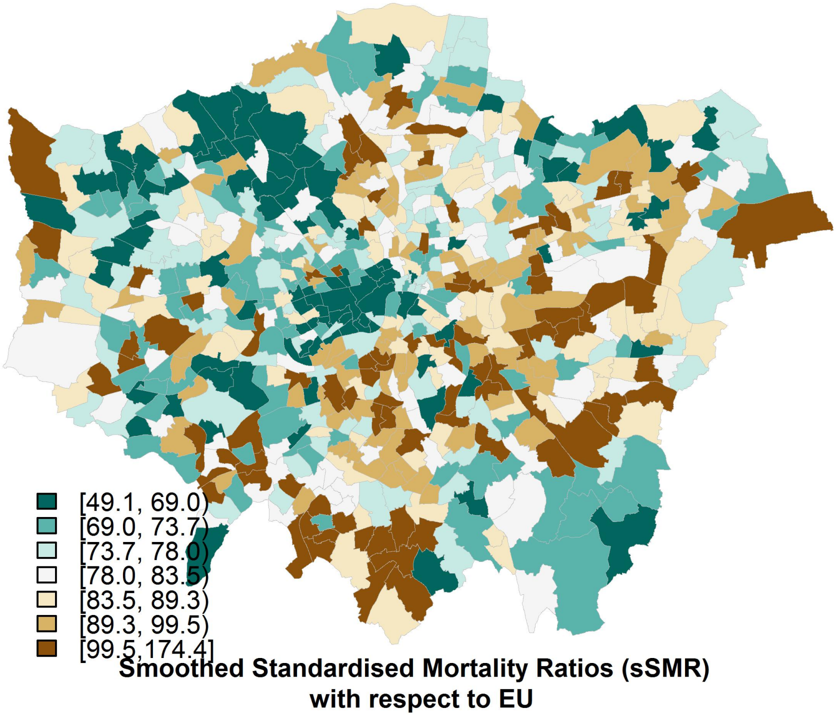


**Smoothed Standardised Mortality Ratios (sSMR)**
**with respect to EU**

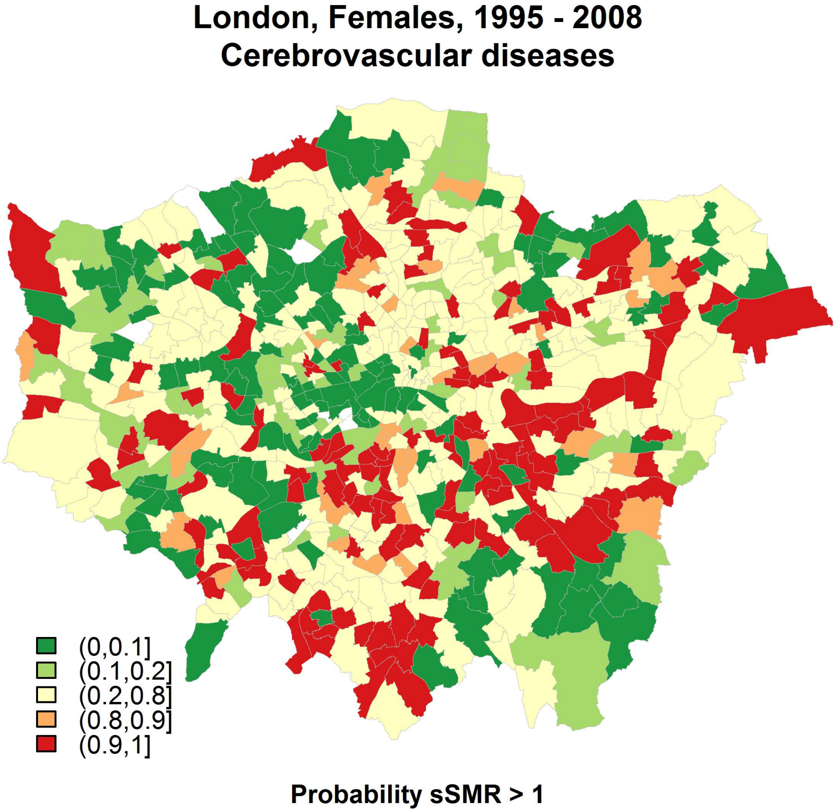
London, Females, 1995 - 2008
Cerebrovascular diseases
(0,0.1]
(0.1,0.2]
(0.2,0.8]
(0.8,0.9]
(0.9,1]
Probability sSMR > 1

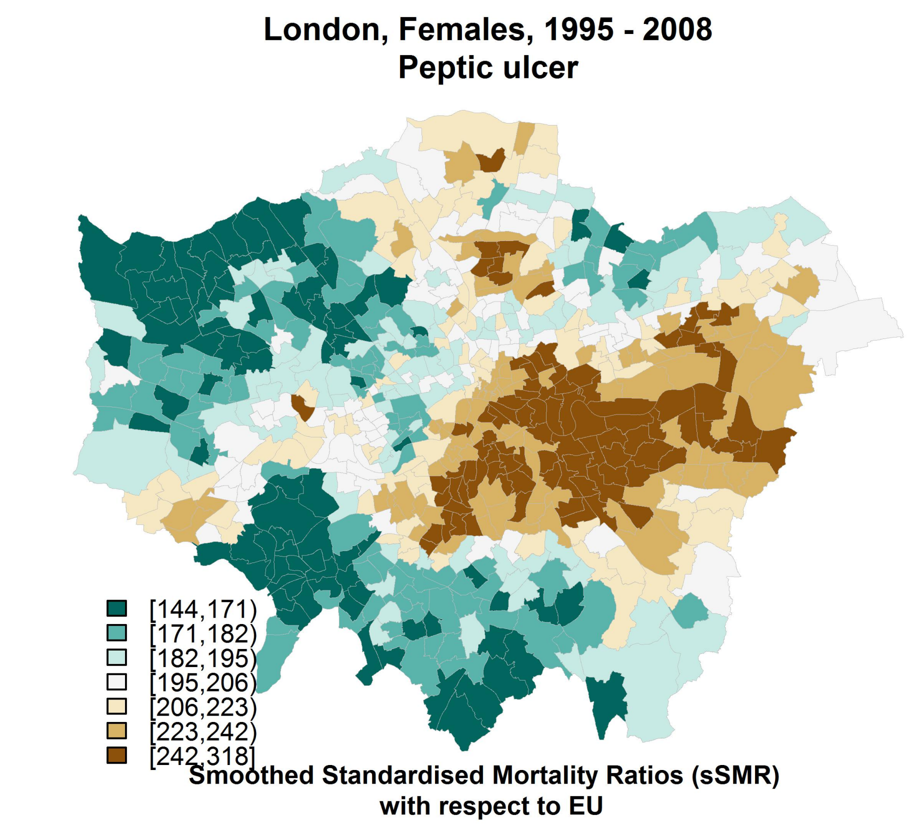
London, Females, 1995 - 2008
Peptic ulcer
[144,171)
[171,182)
[182,195)
[195,206)
[206,223)
[223,242)
[242,318]
Smoothed Standardised Mortality Ratios (sSMR)
with respect to EU

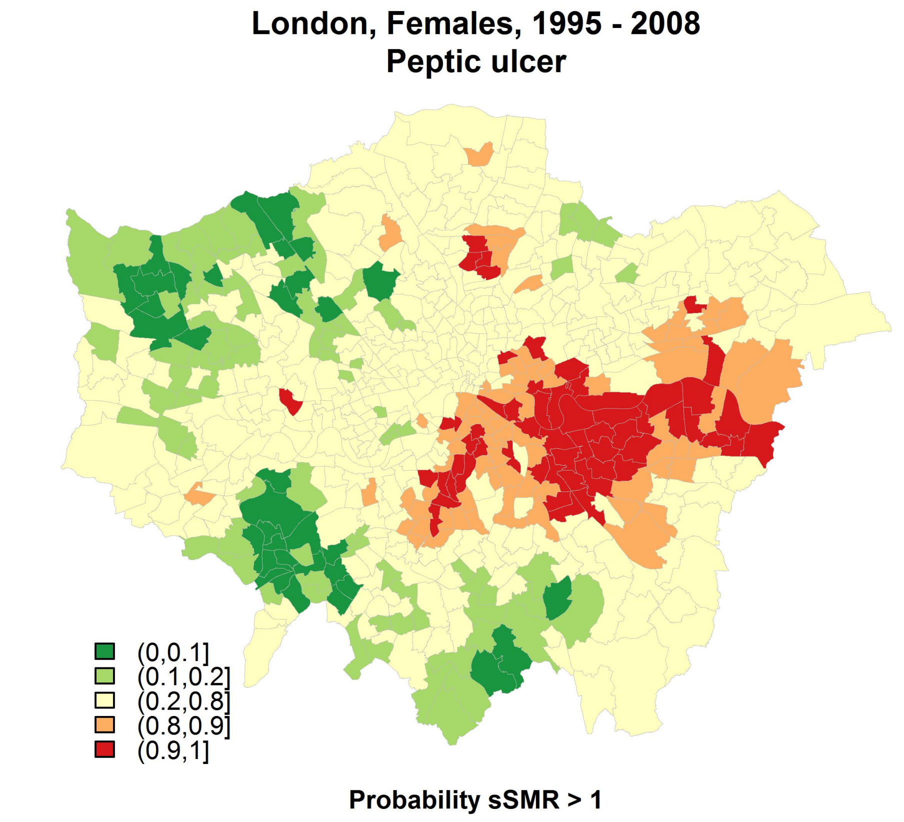
London, Females, 1995 - 2008
Peptic ulcer
(0,0.1]
(0.1,0.2]
(0.2,0.8]
(0.8,0.9]
(0.9,1]
Probability sSMR > 1

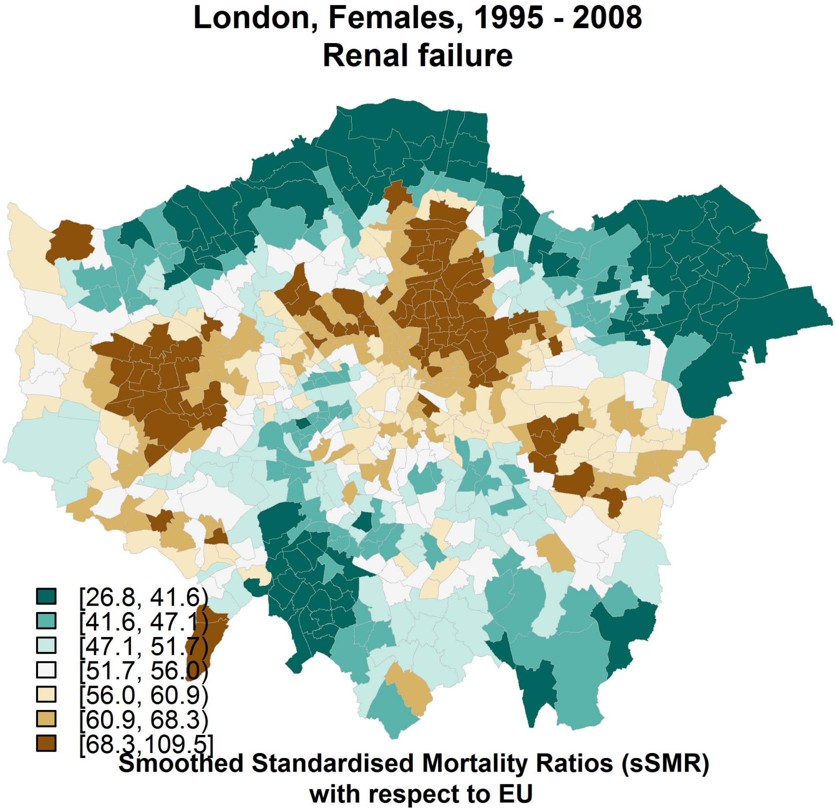
London, Females, 1995 - 2008
Renal failure
[26.8, 41.6)
[41.6, 47.1)
[47.1, 51.7)
[51.7, 56.0)
[56.0, 60.9)
[60.9, 68.3)
[68.3,109.5]
Smoothed Standardised Mortality Ratios (sSMR)
with respect to EU

# London, Females, 1995 - 2008
# Renal failure

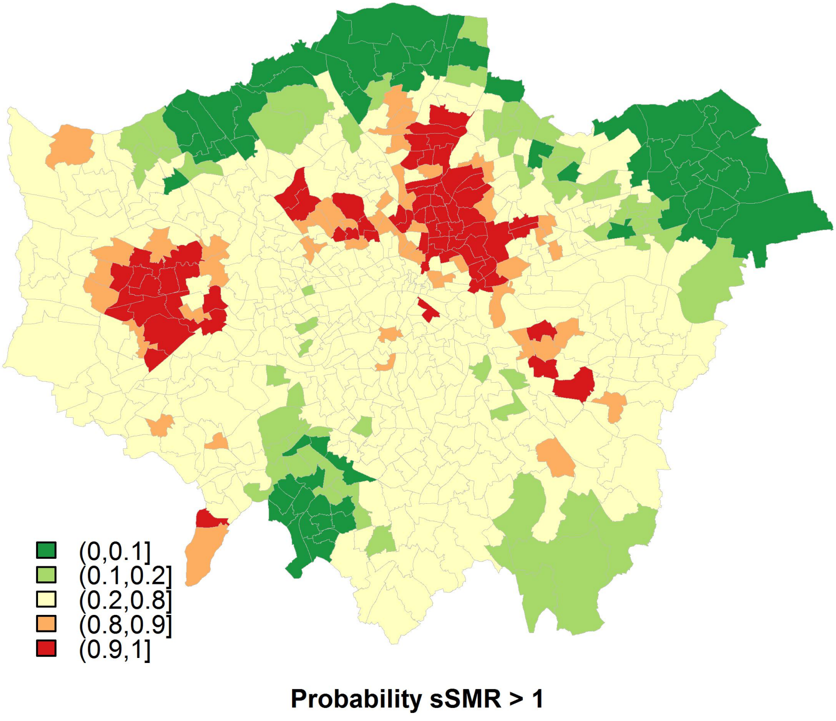

Supplement: Additional file 11 — Cause-specific mortality maps for London. [file 1476-072X-13-8-S11.pdf]
