# Supplementary material for: Social differences in avoidable mortality between small areas of 15 European cities: an ecological study
Source: Int J Health Geogr. 2014 Mar 12;13:8. doi: 10.1186/1476-072X-13-8 (PMC4007807; doi:10.1186/1476-072X-13-8)
Supplement: Additional file 12 — Cause-specific mortality maps for Madrid. [file 1476-072X-13-8-S12.pdf]

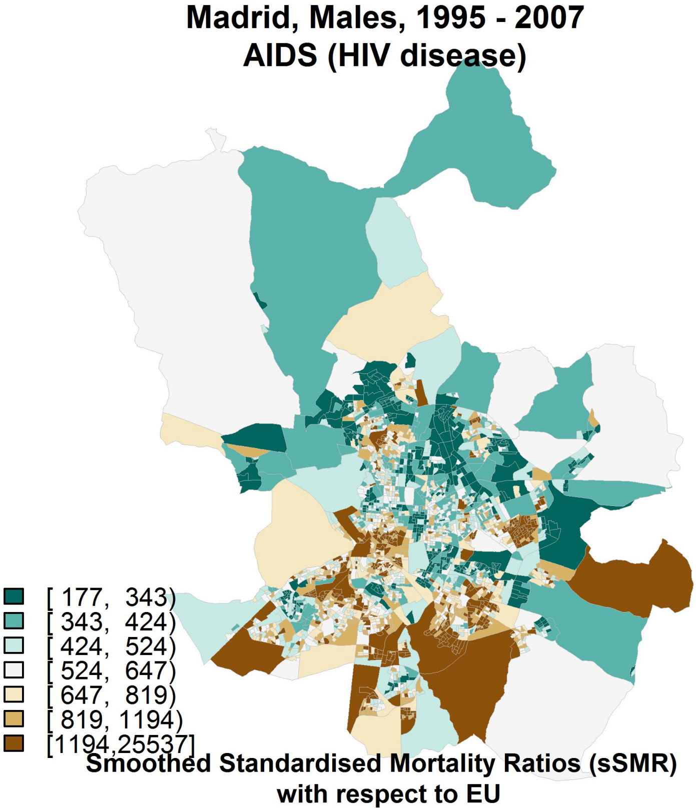

Madrid, Males, 1995 - 2007
AIDS (HIV disease)
[177, 343)
[343, 424)
[424, 524)
[524, 647)
[647, 819)
[819, 1194)
[1194,25537]
Smoothed Standardised Mortality Ratios (sSMR)
with respect to EU

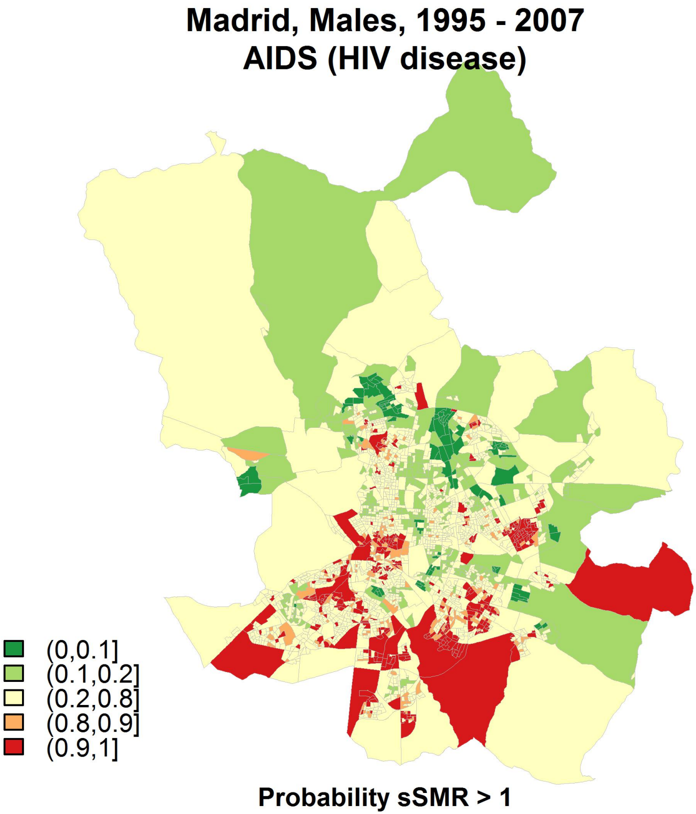
Madrid, Males, 1995 - 2007
AIDS (HIV disease)
(0,0.1]
(0.1,0.2]
(0.2,0.8]
(0.8,0.9]
(0.9,1]
Probability sSMR > 1

Madrid, Males, 1995 - 2007
MN colon

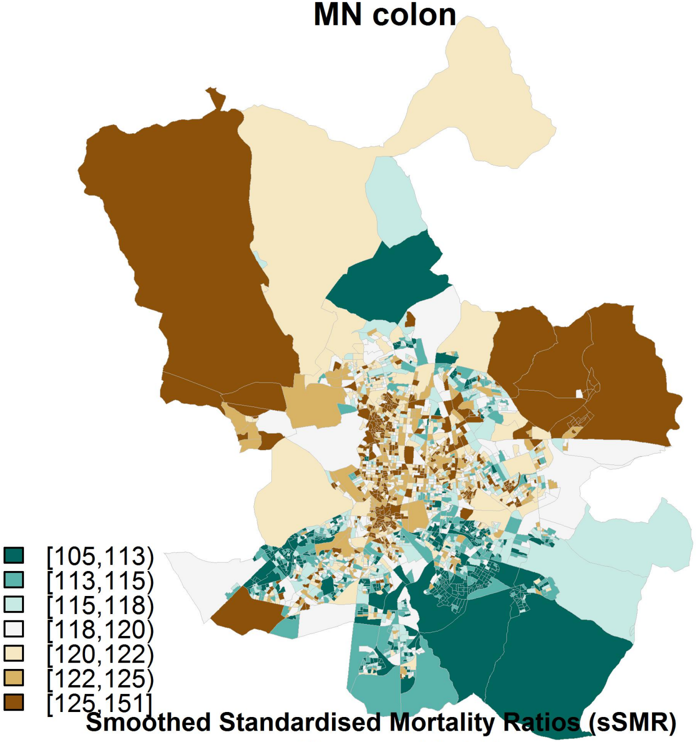

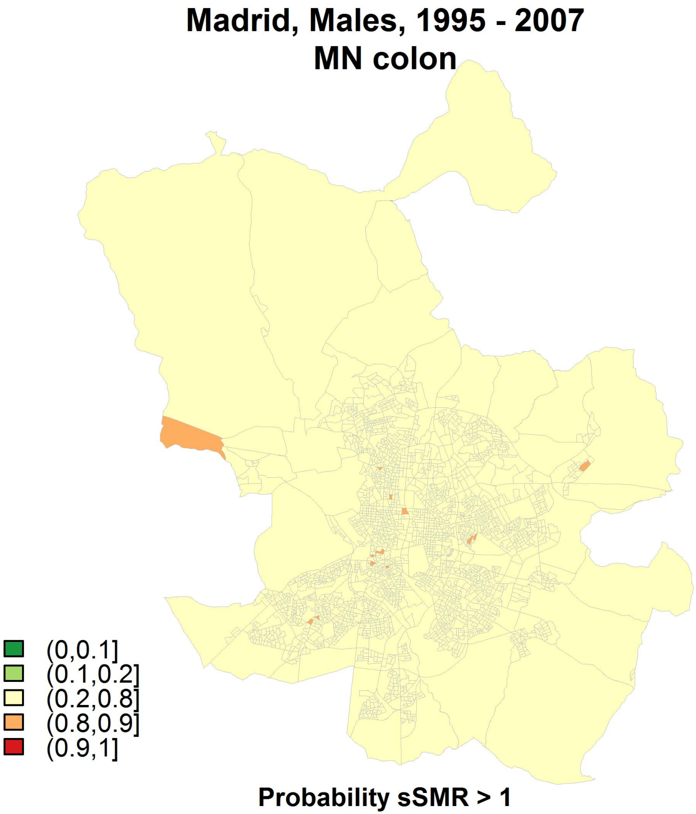
Madrid, Males, 1995 - 2007
MN colon
(0,0.1]
(0.1,0.2]
(0.2,0.8]
(0.8,0.9]
(0.9,1]
Probability sSMR > 1

Madrid, Males, 1995 - 2007
MN rectum, anus and anal canal

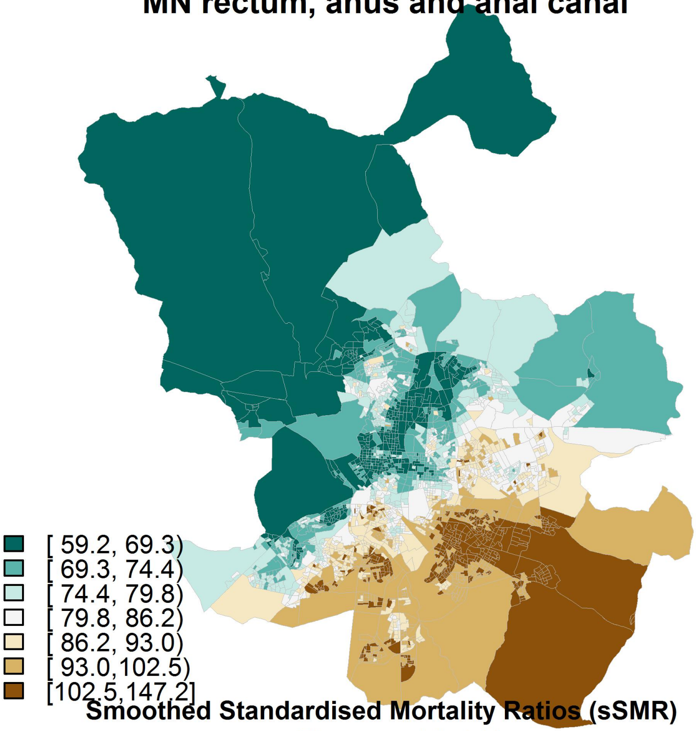

Smoothed Standardised Mortality Ratios (sSMR) with respect to EU

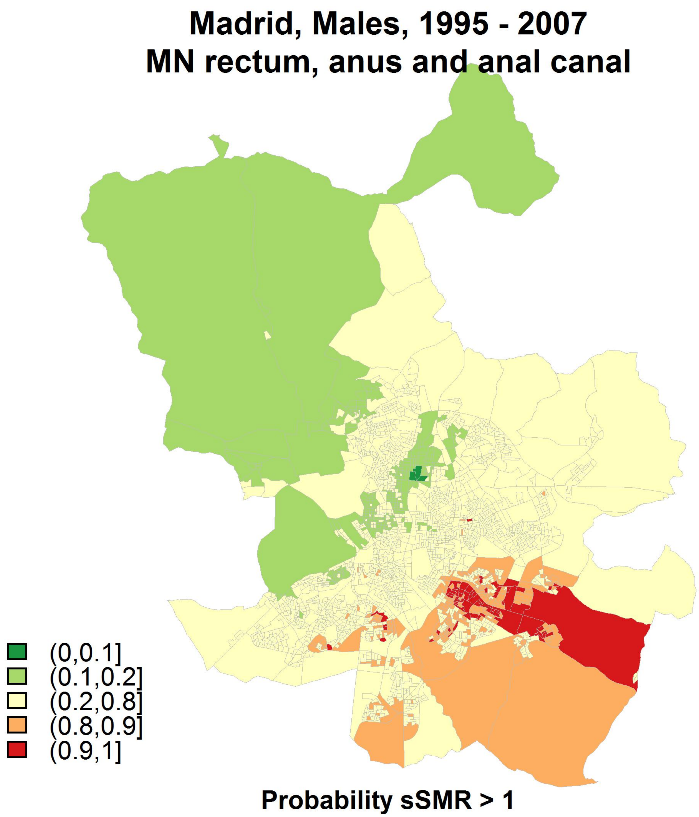
Madrid, Males, 1995 - 2007
MN rectum, anus and anal canal
(0,0.1]
(0.1,0.2]
(0.2,0.8]
(0.8,0.9]
(0.9,1]
Probability sSMR > 1

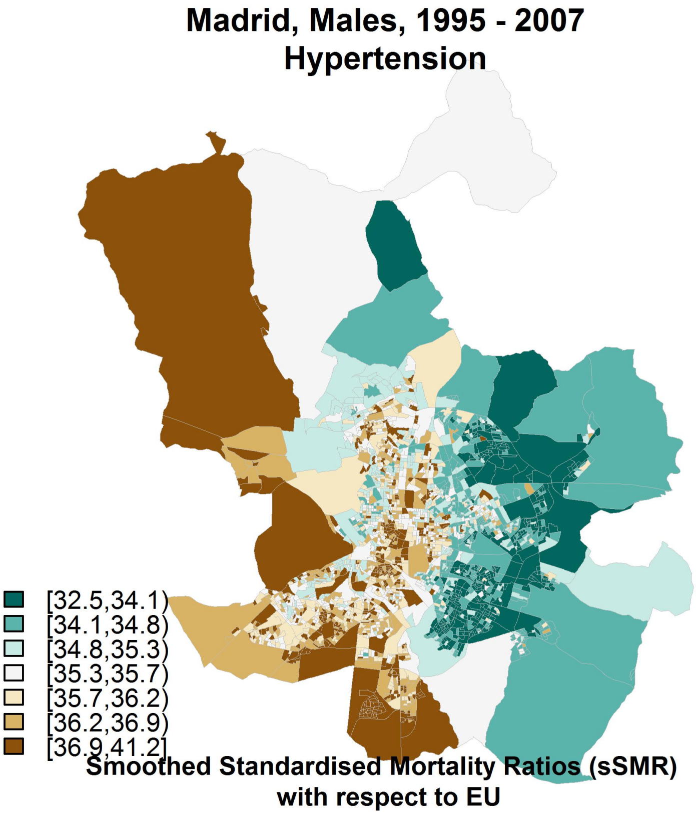
Madrid, Males, 1995 - 2007
Hypertension
[32.5,34.1)
[34.1,34.8)
[34.8,35.3)
[35.3,35.7)
[35.7,36.2)
[36.2,36.9)
[36.9,41.2]
Smoothed Standardised Mortality Ratios (sSMR)
with respect to EU

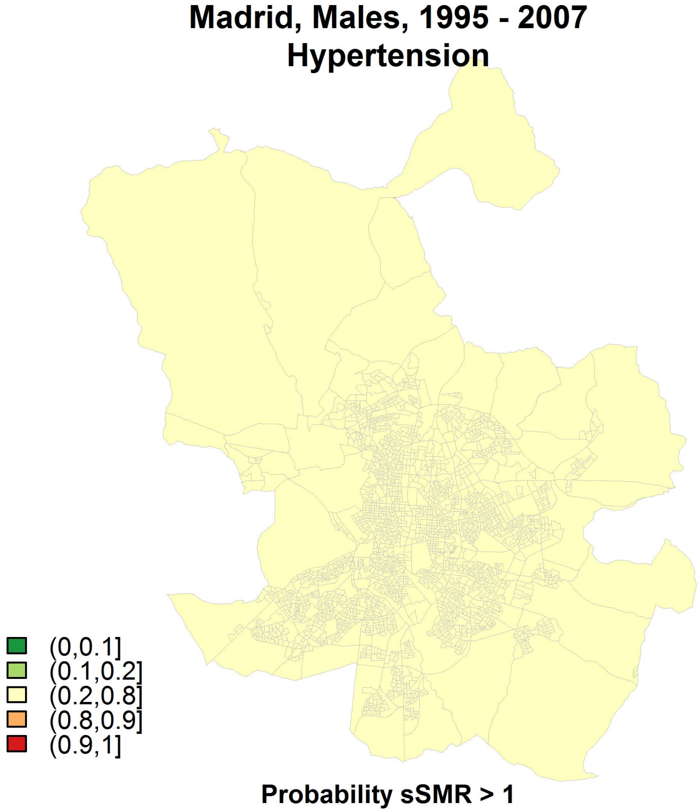
Madrid, Males, 1995 - 2007
Hypertension
(0,0.1]
(0.1,0.2]
(0.2,0.8]
(0.8,0.9]
(0.9,1]
Probability sSMR > 1

Madrid, Males, 1995 - 2007
Heart failure

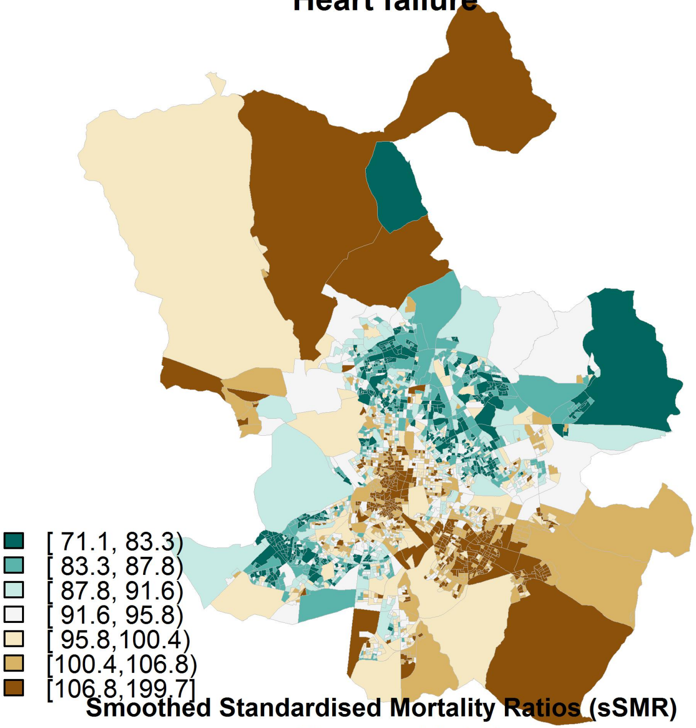

Smoothed Standardised Mortality Ratios (sSMR)
with respect to EU

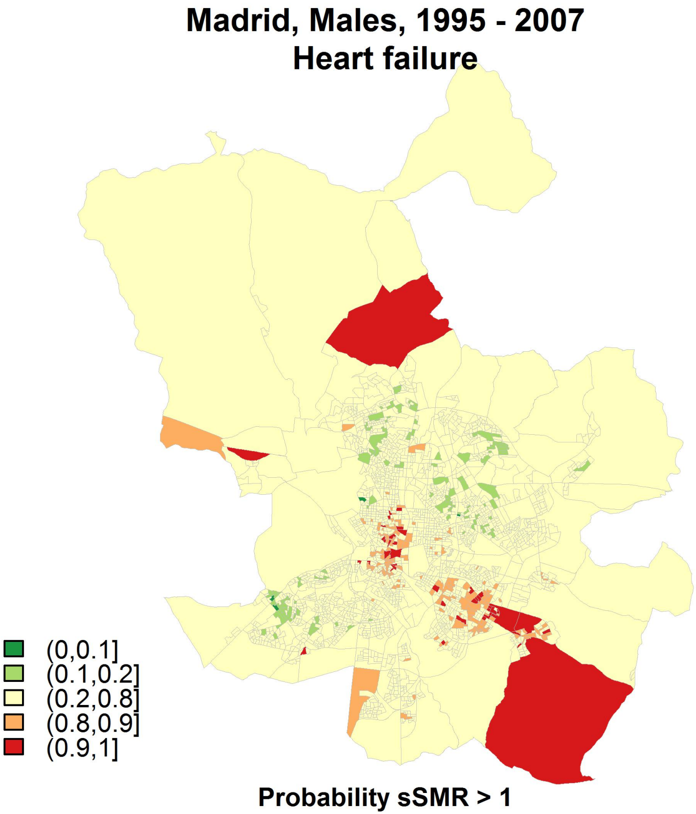
Madrid, Males, 1995 - 2007
Heart failure
(0,0.1]
(0.1,0.2]
(0.2,0.8]
(0.8,0.9]
(0.9,1]
Probability sSMR > 1

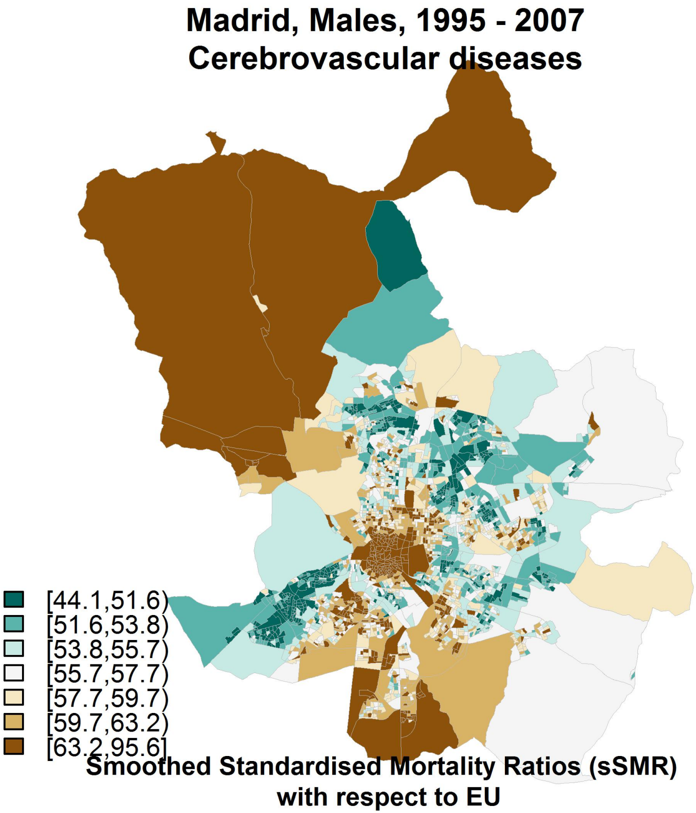
Madrid, Males, 1995 - 2007
Cerebrovascular diseases
[44.1,51.6)
[51.6,53.8)
[53.8,55.7)
[55.7,57.7)
[57.7,59.7)
[59.7,63.2)
[63.2,95.6]
Smoothed Standardised Mortality Ratios (sSMR)
with respect to EU

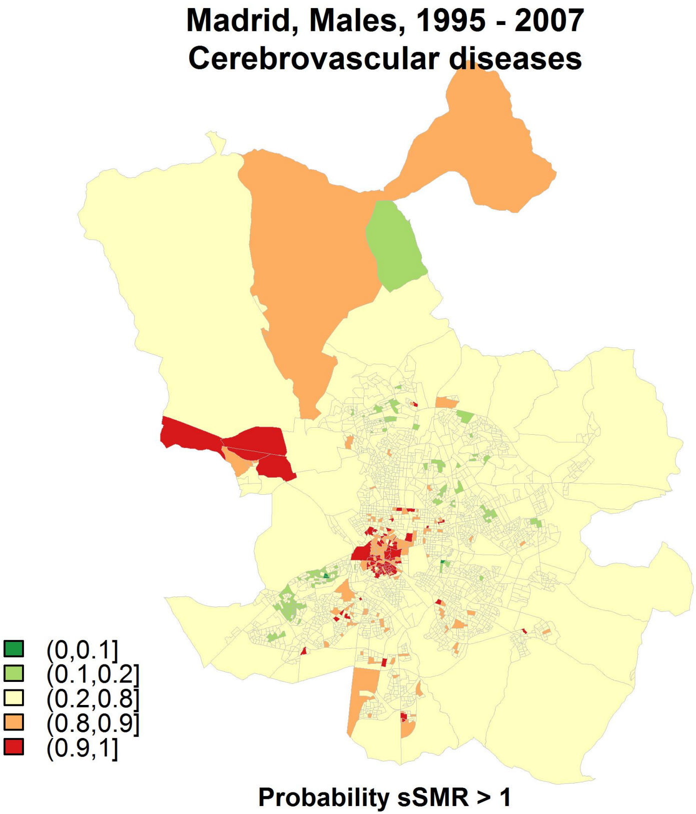
Madrid, Males, 1995 - 2007
Cerebrovascular diseases
(0,0.1]
(0.1,0.2]
(0.2,0.8]
(0.8,0.9]
(0.9,1]
Probability sSMR > 1

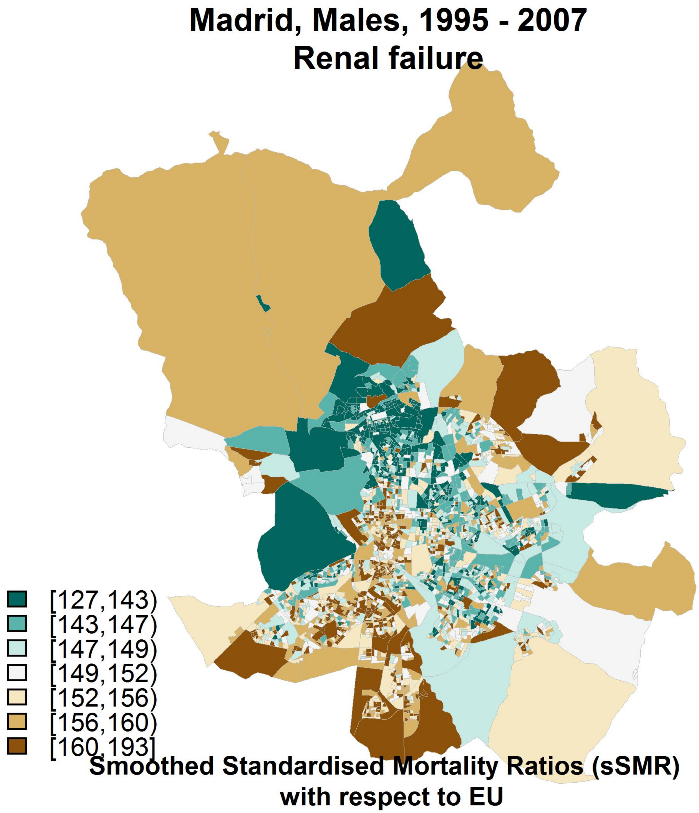
Madrid, Males, 1995 - 2007
Renal failure
[127,143)
[143,147)
[147,149)
[149,152)
[152,156)
[156,160)
[160,193]
Smoothed Standardised Mortality Ratios (sSMR)
with respect to EU

Madrid, Males, 1995 - 2007
Renal failure

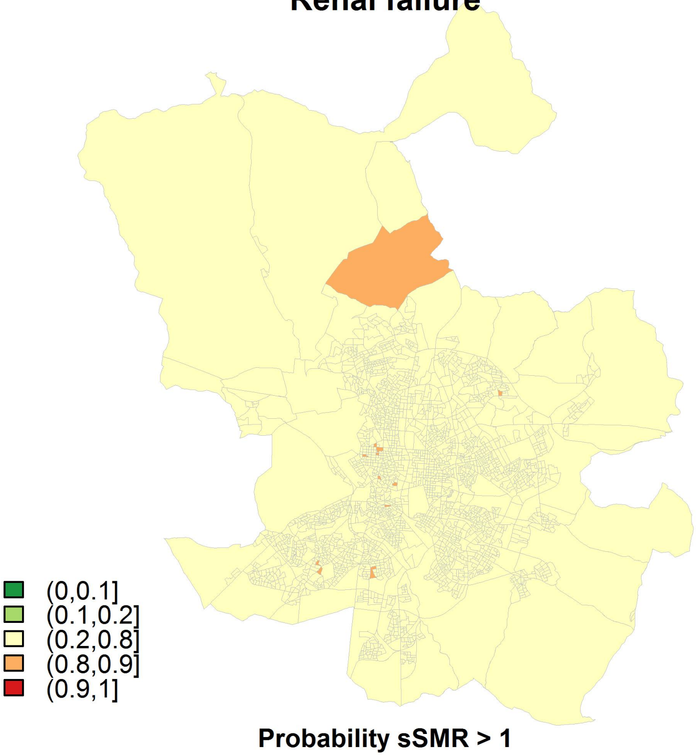

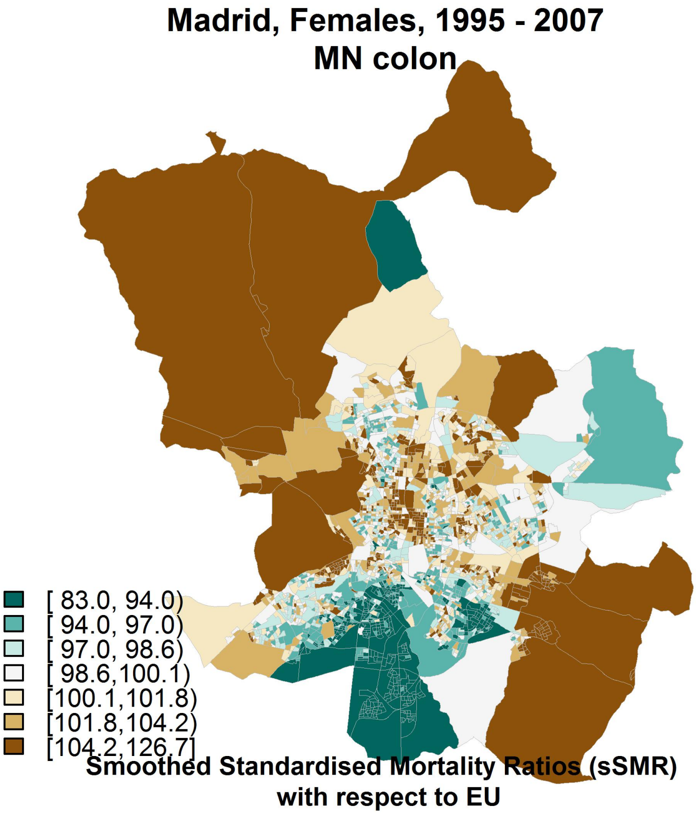
Madrid, Females, 1995 - 2007
MN colon
[83.0, 94.0)
[94.0, 97.0)
[97.0, 98.6)
[98.6,100.1)
[100.1,101.8)
[101.8,104.2)
[104.2,126.7]
Smoothed Standardised Mortality Ratios (sSMR)
with respect to EU

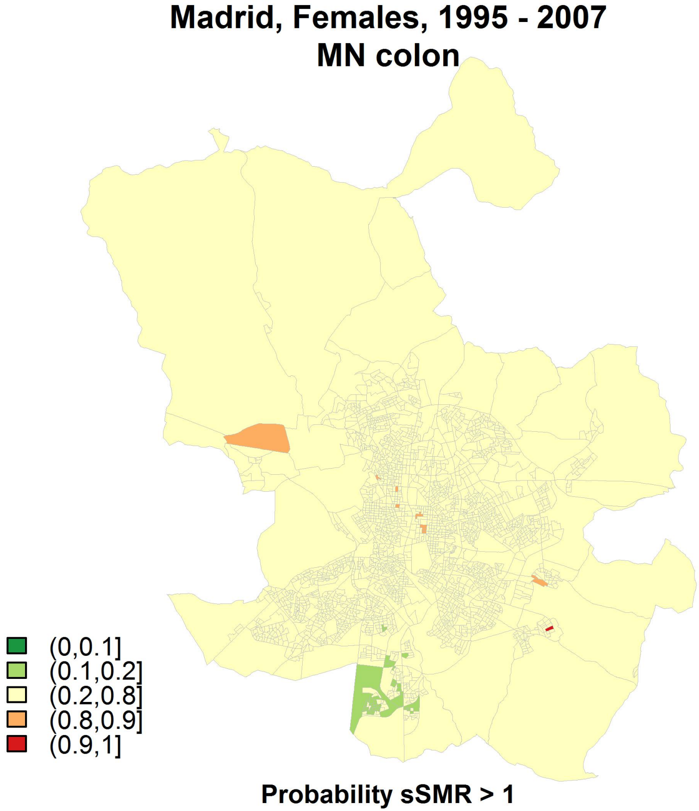
Madrid, Females, 1995 - 2007
MN colon
(0,0.1]
(0.1,0.2]
(0.2,0.8]
(0.8,0.9]
(0.9,1]
Probability sSMR > 1

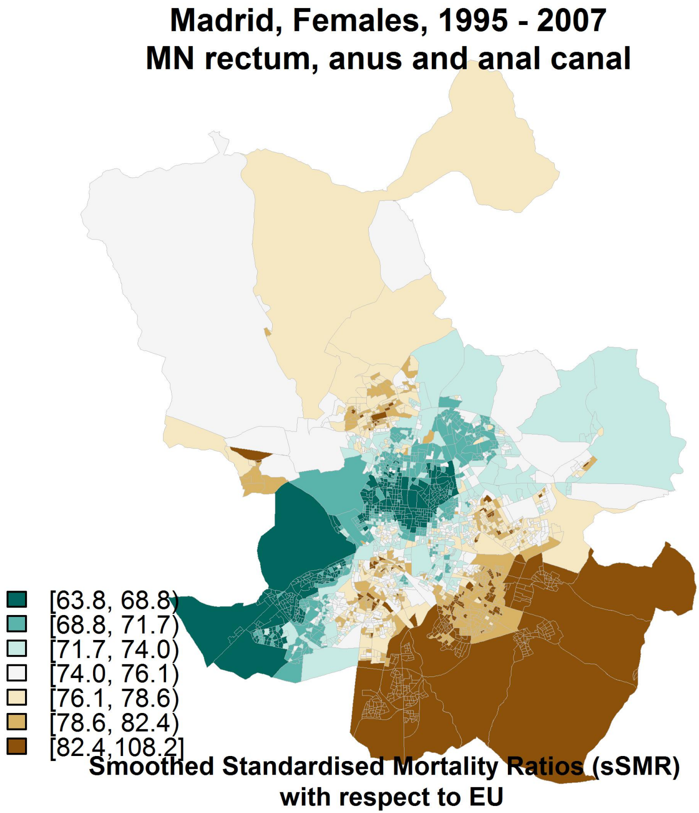
Madrid, Females, 1995 - 2007
MN rectum, anus and anal canal
[63.8, 68.8)
[68.8, 71.7)
[71.7, 74.0)
[74.0, 76.1)
[76.1, 78.6)
[78.6, 82.4)
[82.4,108.2]
Smoothed Standardised Mortality Ratios (sSMR)
with respect to EU

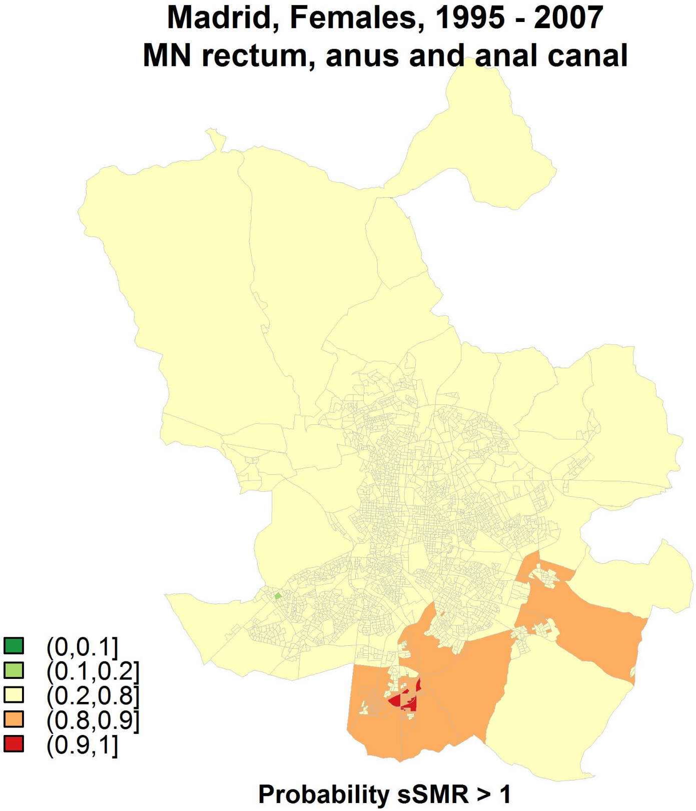
Madrid, Females, 1995 - 2007
MN rectum, anus and anal canal
(0,0.1]
(0.1,0.2]
(0.2,0.8]
(0.8,0.9]
(0.9,1]
Probability sSMR > 1

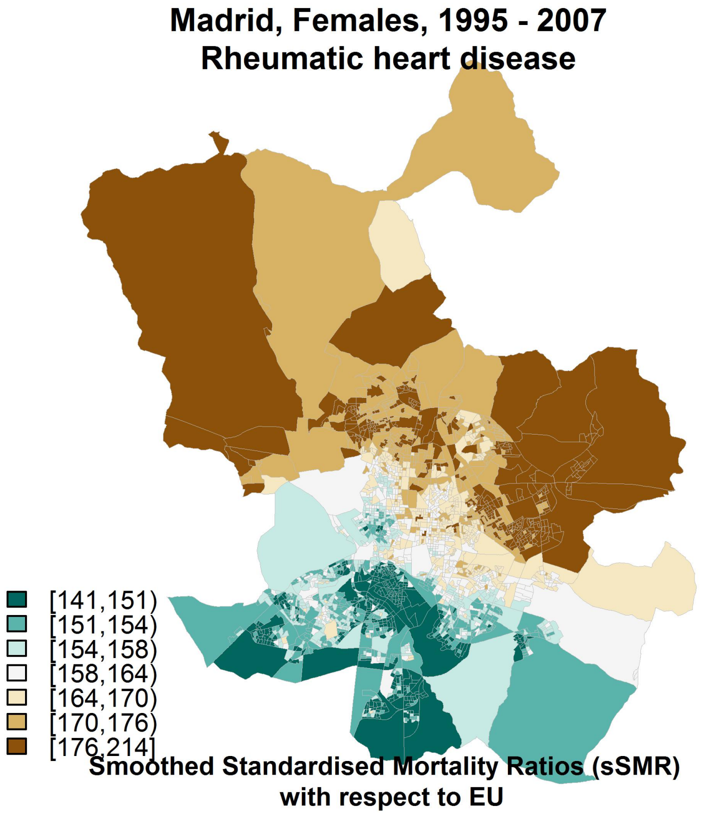
Madrid, Females, 1995 - 2007
Rheumatic heart disease
[141,151)
[151,154)
[154,158)
[158,164)
[164,170)
[170,176)
[176,214]
Smoothed Standardised Mortality Ratios (sSMR)
with respect to EU

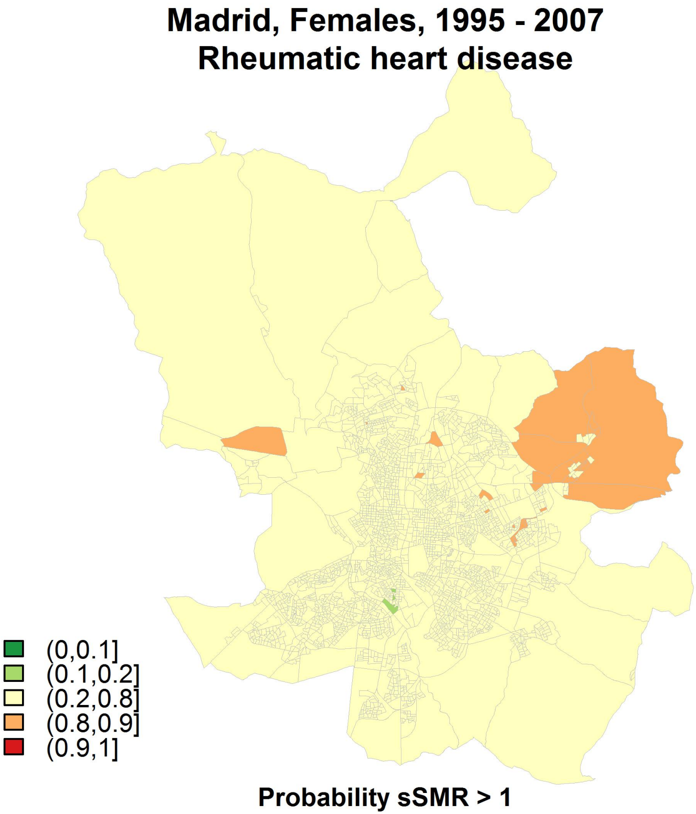
Madrid, Females, 1995 - 2007
Rheumatic heart disease
(0,0.1]
(0.1,0.2]
(0.2,0.8]
(0.8,0.9]
(0.9,1]
Probability sSMR > 1

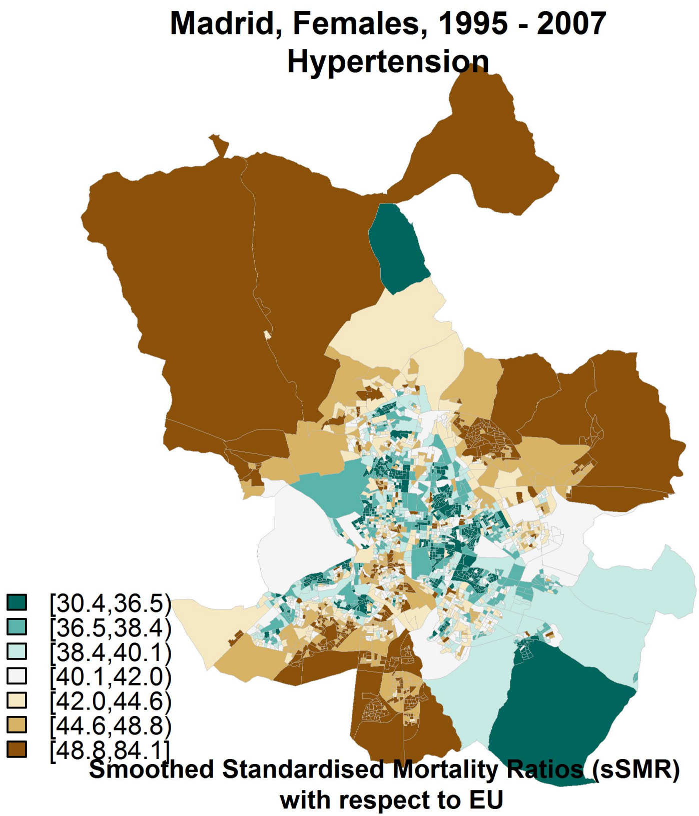
Madrid, Females, 1995 - 2007
Hypertension
[30.4,36.5)
[36.5,38.4)
[38.4,40.1)
[40.1,42.0)
[42.0,44.6)
[44.6,48.8)
[48.8,84.1]
Smoothed Standardised Mortality Ratios (sSMR)
with respect to EU

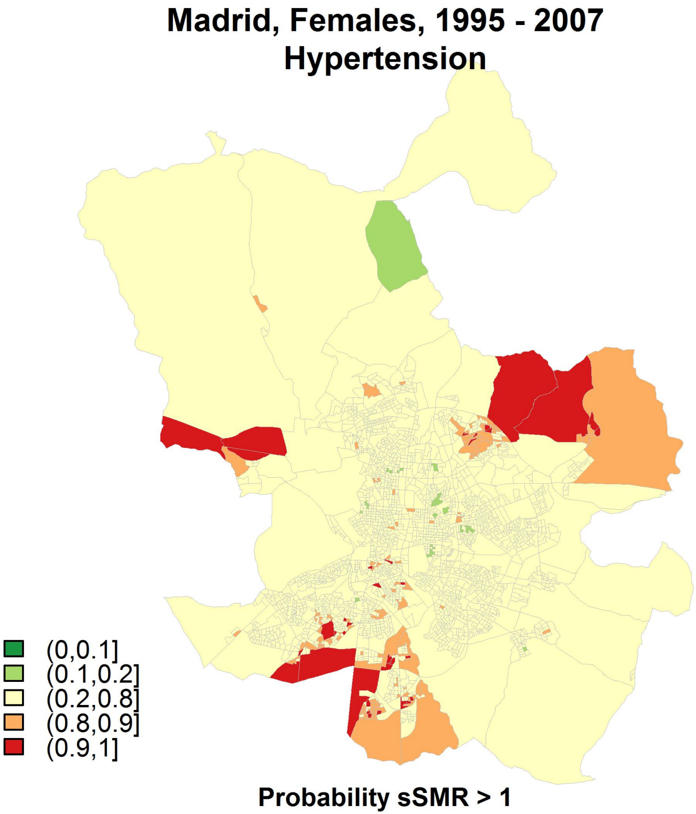
Madrid, Females, 1995 - 2007
Hypertension
(0,0.1]
(0.1,0.2]
(0.2,0.8]
(0.8,0.9]
(0.9,1]
Probability sSMR > 1

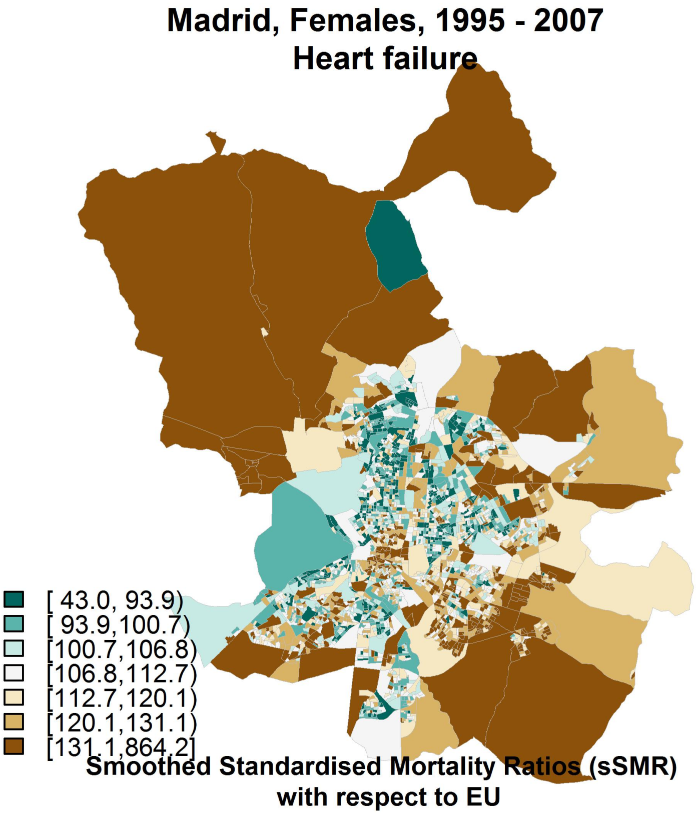
Madrid, Females, 1995 - 2007
Heart failure
[43.0, 93.9)
[93.9,100.7)
[100.7,106.8)
[106.8,112.7)
[112.7,120.1)
[120.1,131.1)
[131.1,864.2]
Smoothed Standardised Mortality Ratios (sSMR)
with respect to EU

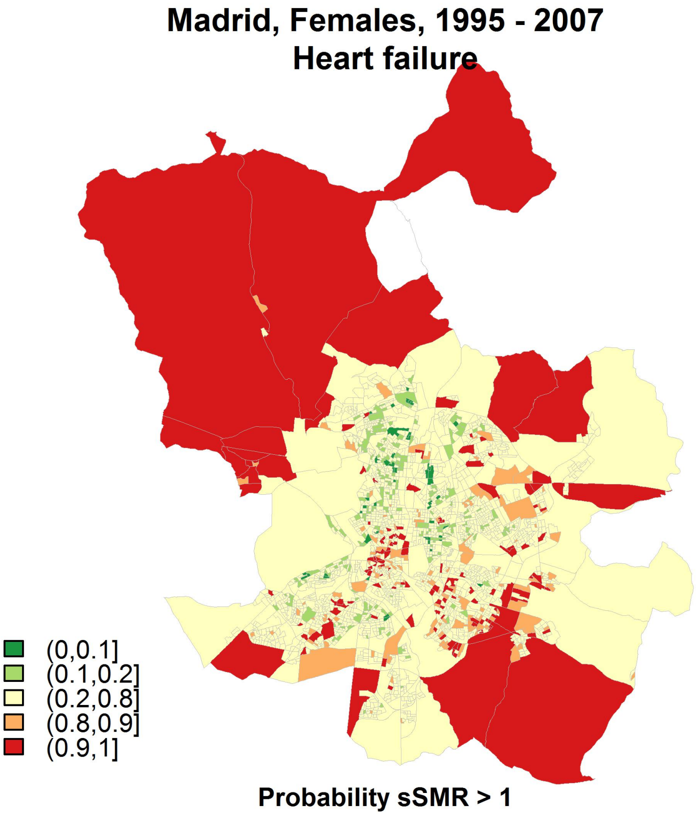
Madrid, Females, 1995 - 2007
Heart failure
(0,0.1]
(0.1,0.2]
(0.2,0.8]
(0.8,0.9]
(0.9,1]
Probability sSMR > 1

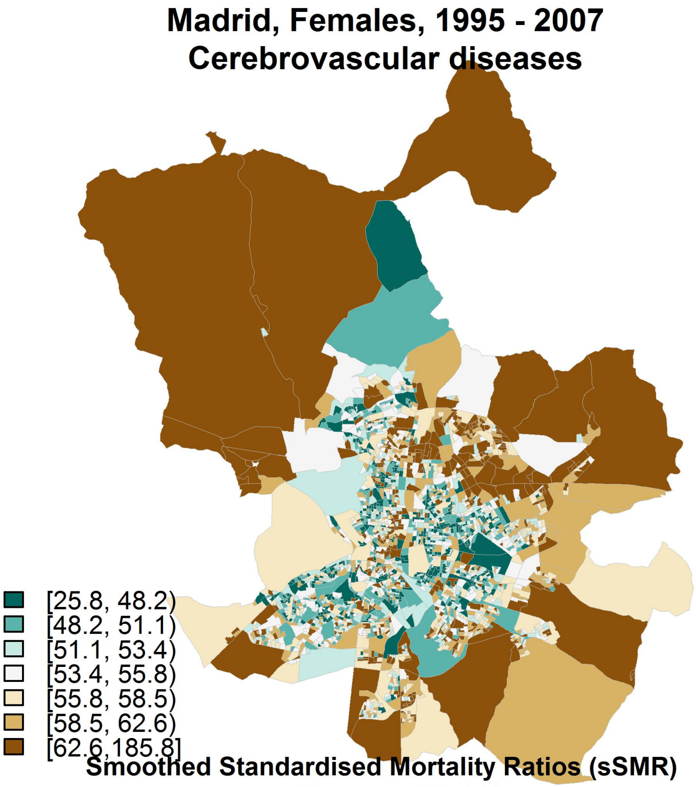

Smoothed Standardised Mortality Ratios (sSMR)
with respect to EU

Madrid, Females, 1995 - 2007
Cerebrovascular diseases

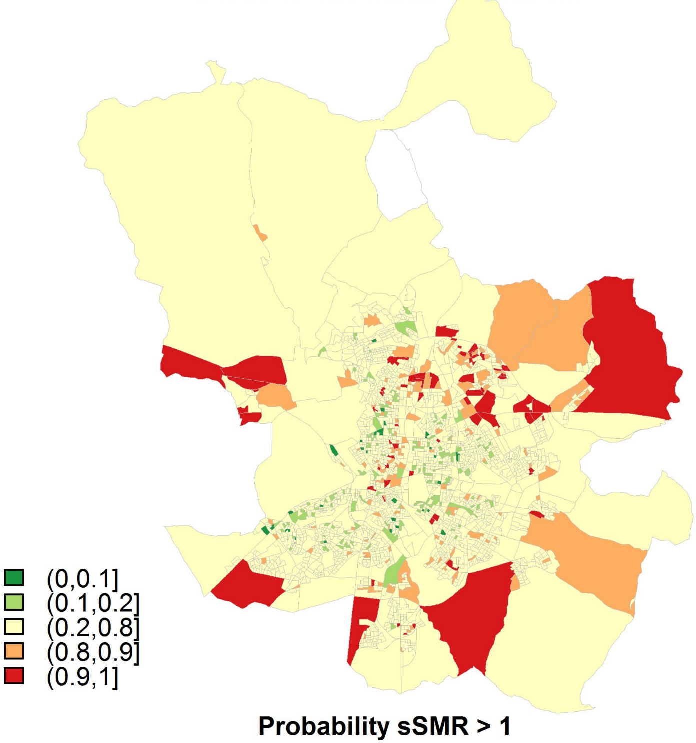

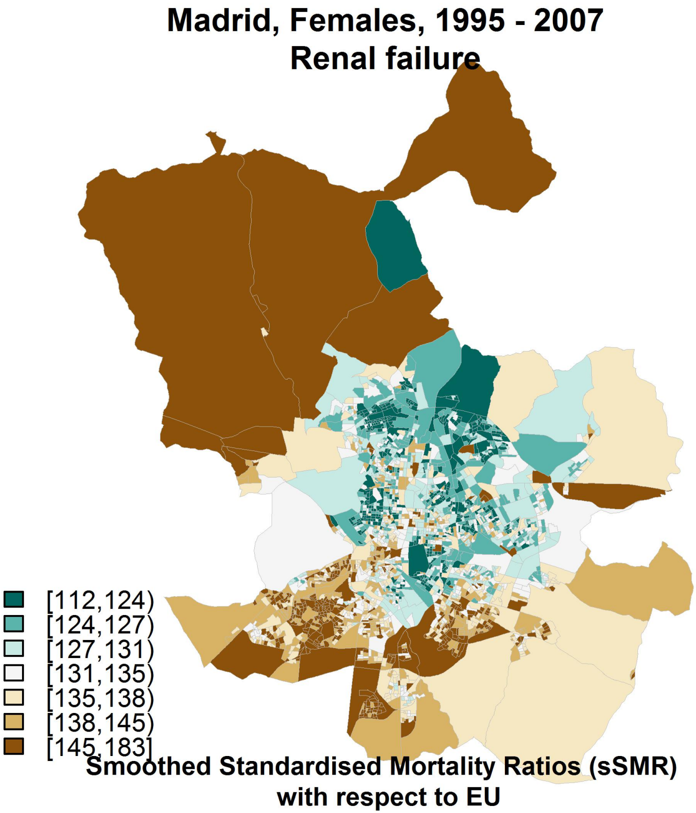
Madrid, Females, 1995 - 2007
Renal failure
[112,124)
[124,127)
[127,131)
[131,135)
[135,138)
[138,145)
[145,183]
Smoothed Standardised Mortality Ratios (sSMR)
with respect to EU

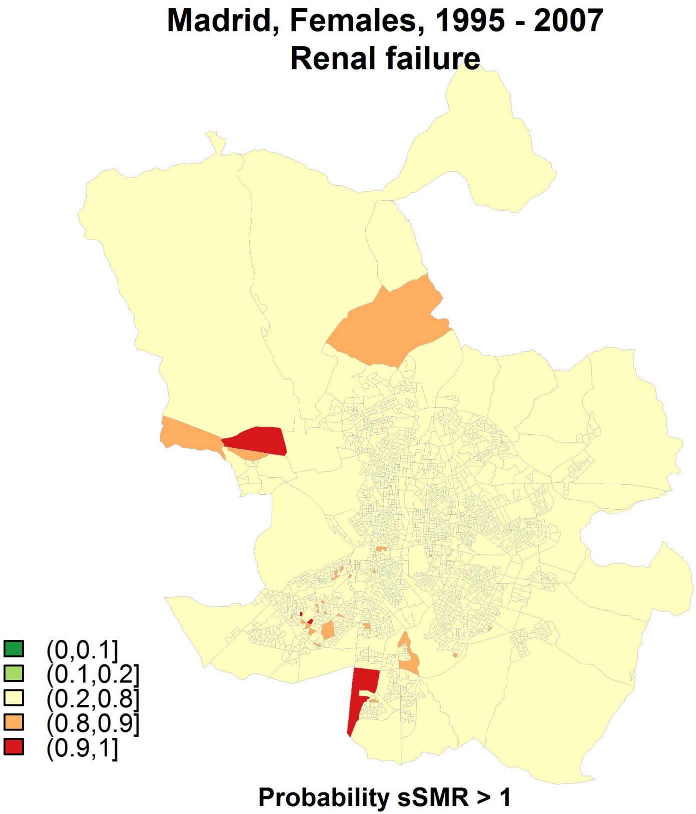
Madrid, Females, 1995 - 2007
Renal failure
(0,0.1]
(0.1,0.2]
(0.2,0.8]
(0.8,0.9]
(0.9,1]
Probability sSMR > 1
