# Supplementary material for: Social differences in avoidable mortality between small areas of 15 European cities: an ecological study
Source: Int J Health Geogr. 2014 Mar 12;13:8. doi: 10.1186/1476-072X-13-8 (PMC4007807; doi:10.1186/1476-072X-13-8)

Prague, Males, 2003 - 2007
MN colon

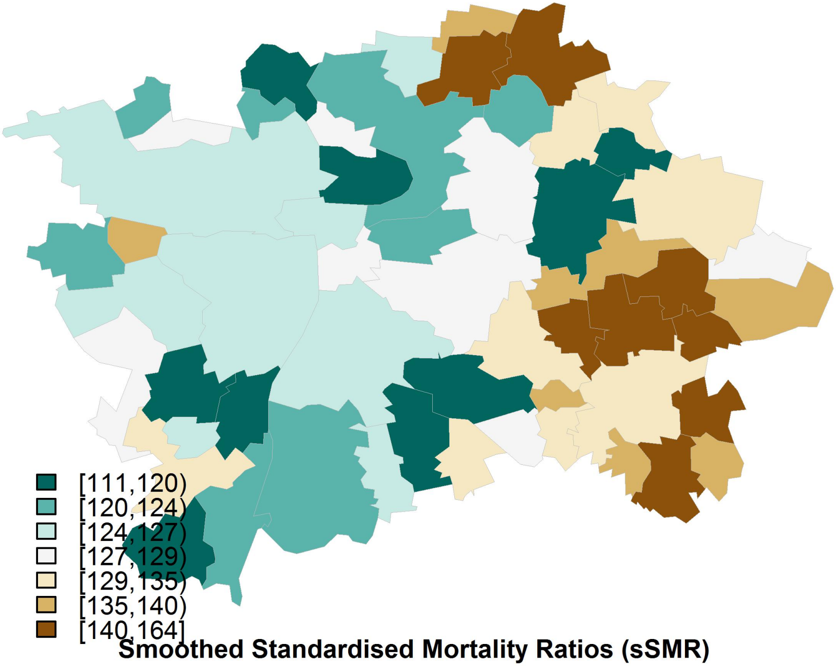

Smoothed Standardised Mortality Ratios (sSMR) with respect to EU

Prague, Males, 2003 - 2007
MN colon

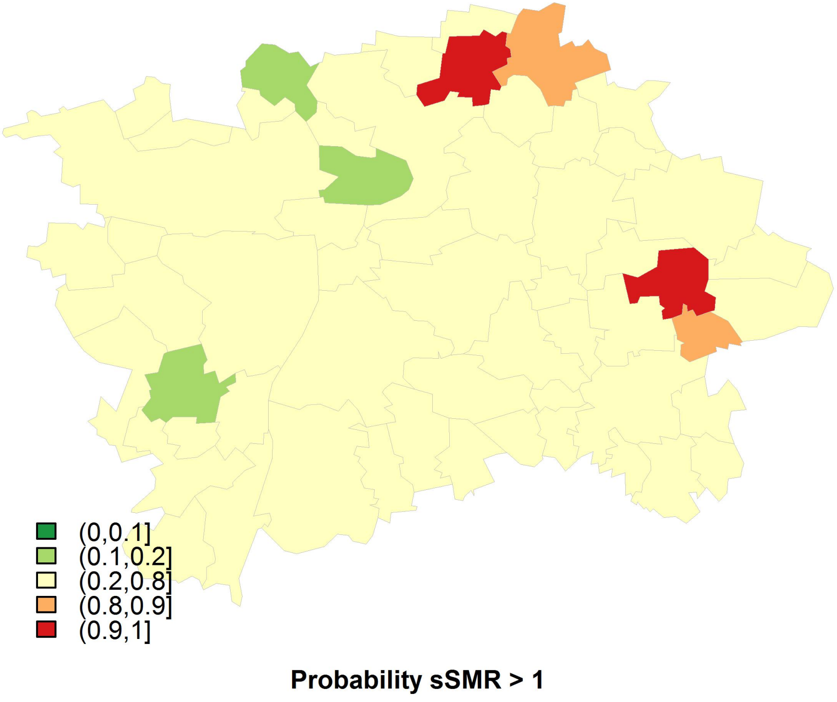

Prague, Males, 2003 - 2007
MN rectum, anus and anal canal

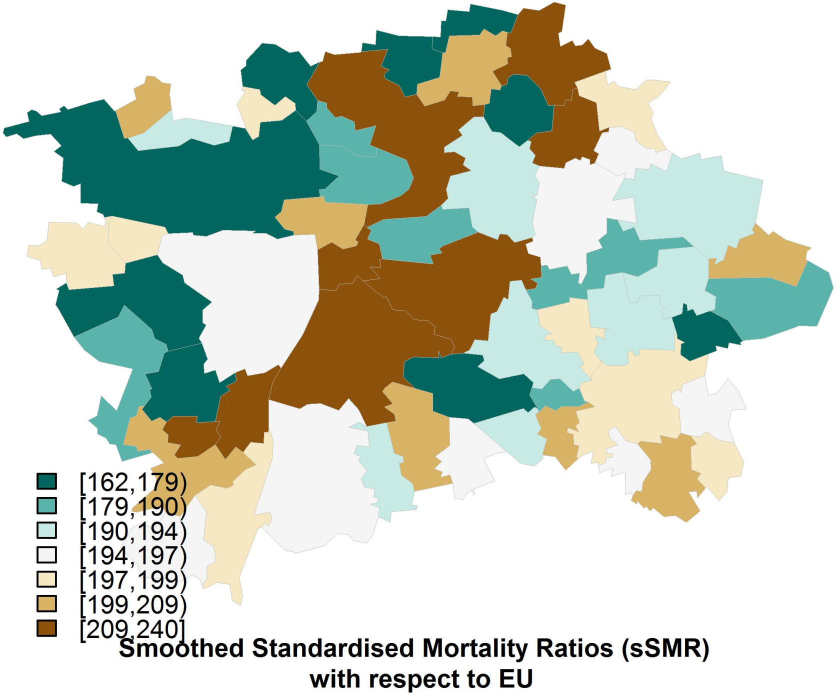

Smoothed Standardised Mortality Ratios (sSMR)
with respect to EU

Prague, Males, 2003 - 2007
MN rectum, anus and anal canal

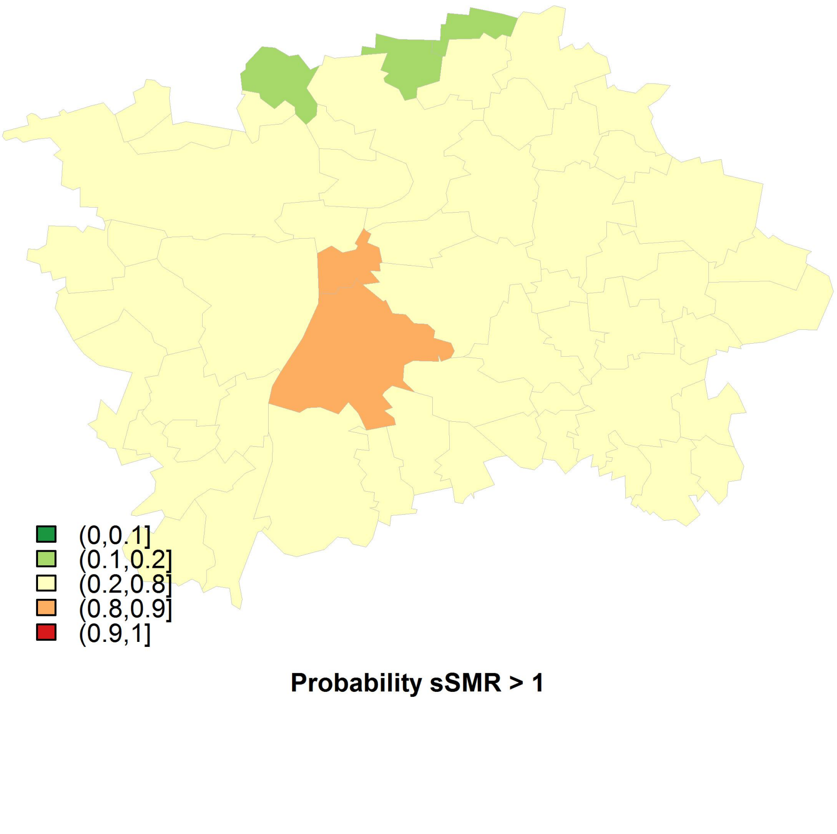

Probability sSMR > 1

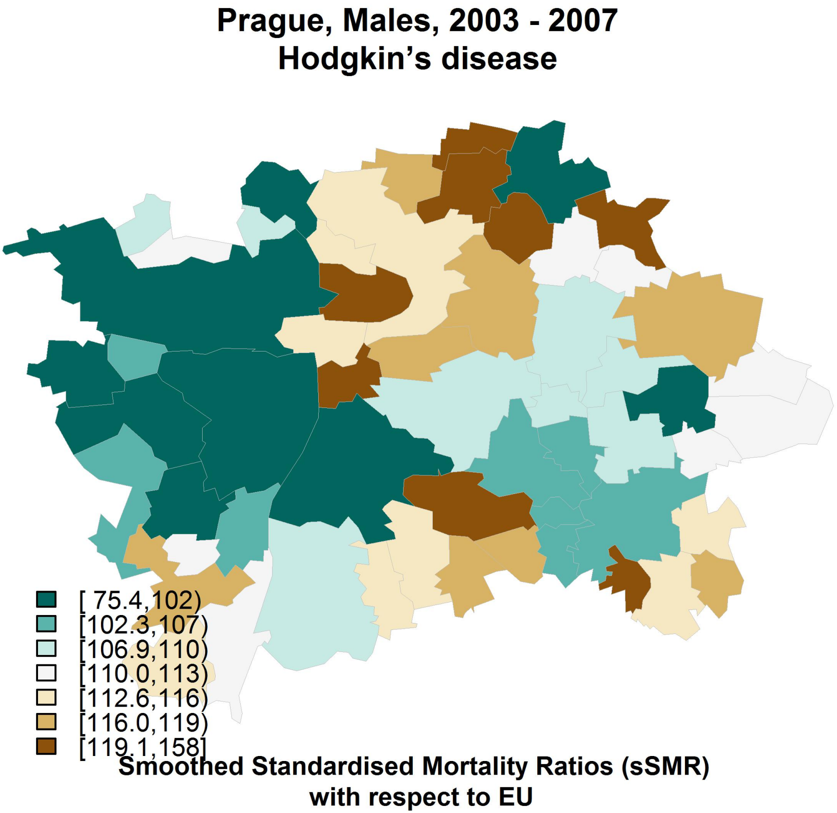
Prague, Males, 2003 - 2007
Hodgkin's disease
[75.4,102)
[102.3,107)
[106.9,110)
[110.0,113)
[112.6,116)
[116.0,119)
[119.1,158]
Smoothed Standardised Mortality Ratios (sSMR)
with respect to EU

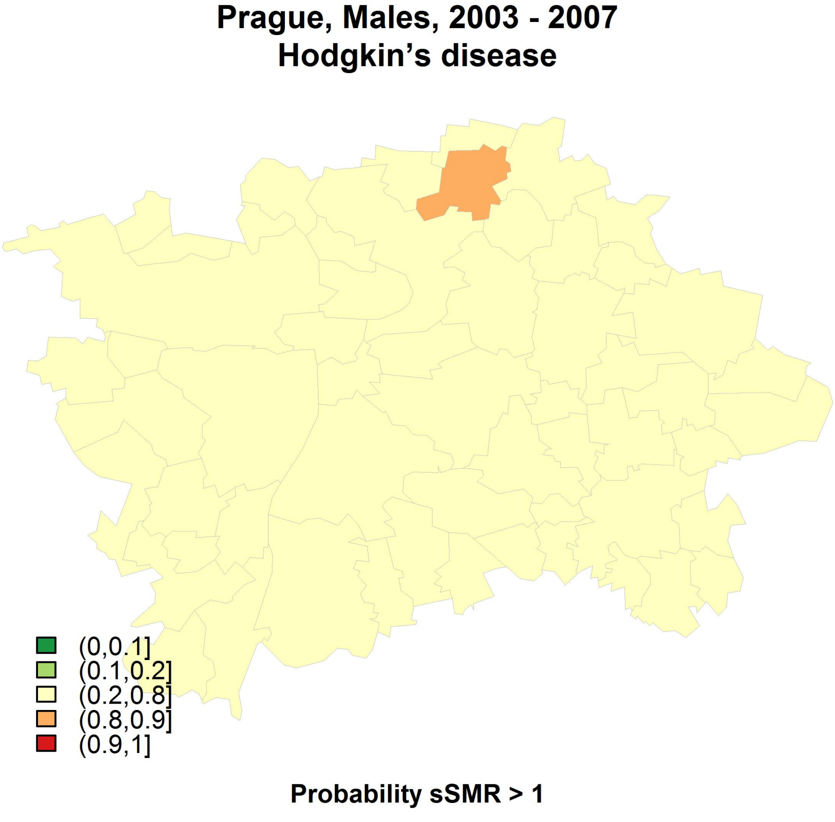

Prague, Males, 2003 - 2007
Hodgkin's disease
(0,0.1]
(0.1,0.2]
(0.2,0.8]
(0.8,0.9]
(0.9,1]
Probability sSMR > 1

Prague, Males, 2003 - 2007
Rheumatic heart disease

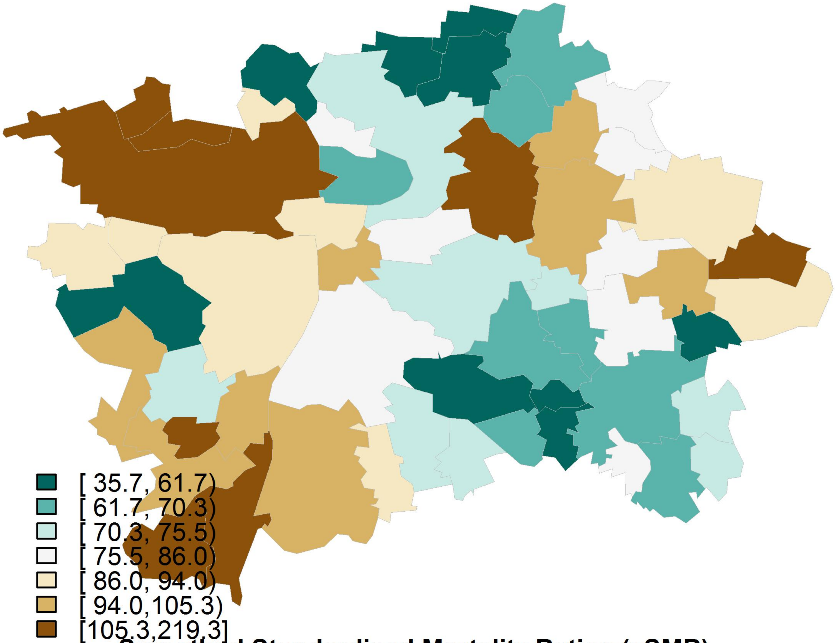

Smoothed Standardised Mortality Ratios (sSMR) with respect to EU

Prague, Males, 2003 - 2007
Rheumatic heart disease

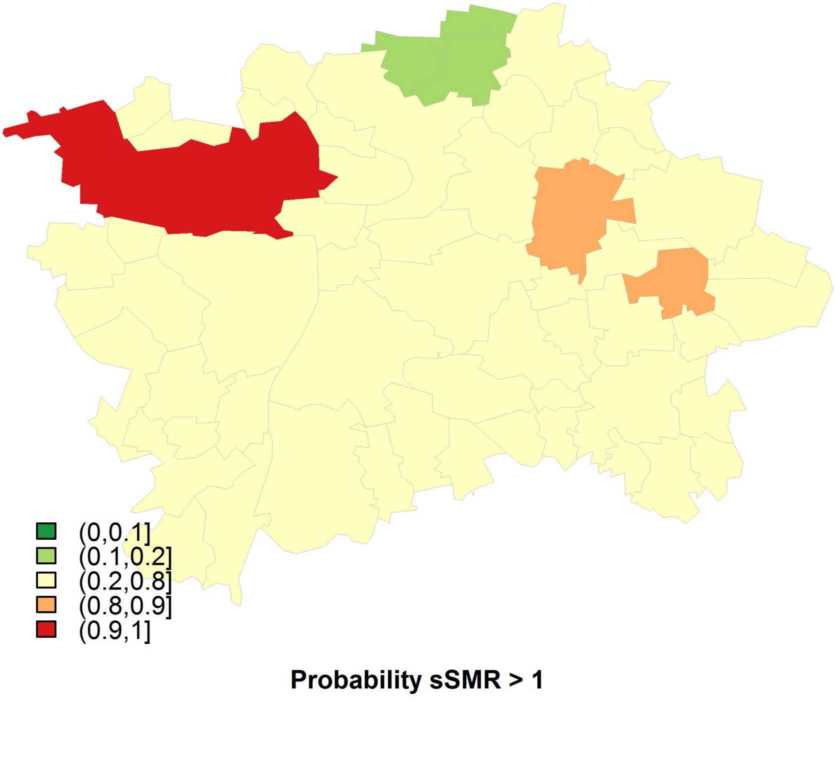

Probability sSMR > 1

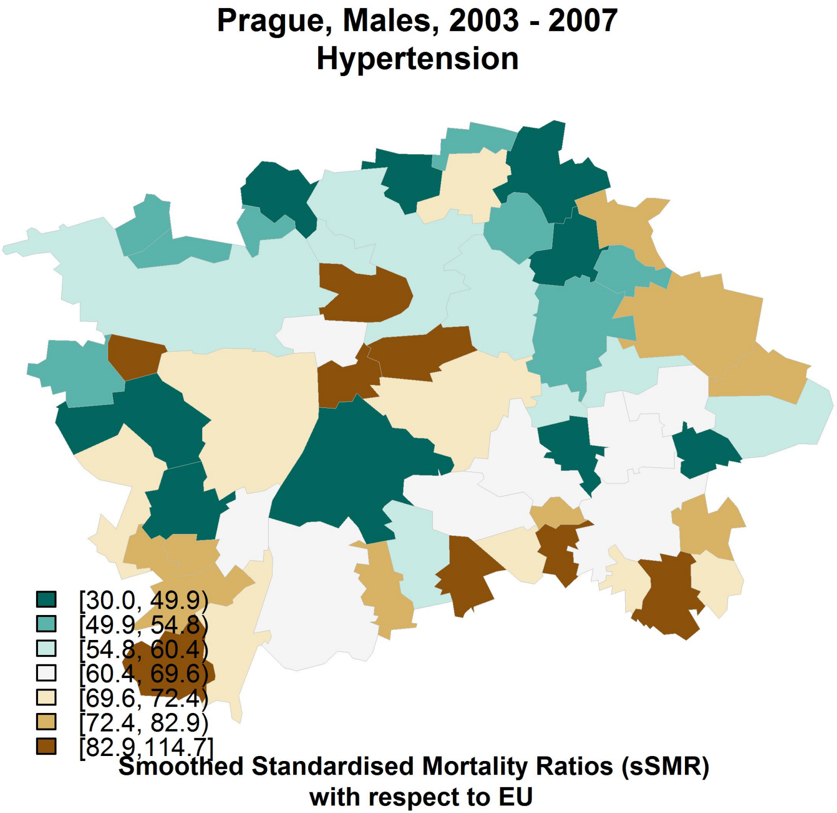
Prague, Males, 2003 - 2007
Hypertension
[30.0, 49.9)
[49.9, 54.8)
[54.8, 60.4)
[60.4, 69.6)
[69.6, 72.4)
[72.4, 82.9)
[82.9,114.7]
Smoothed Standardised Mortality Ratios (sSMR)
with respect to EU

Prague, Males, 2003 - 2007
Hypertension

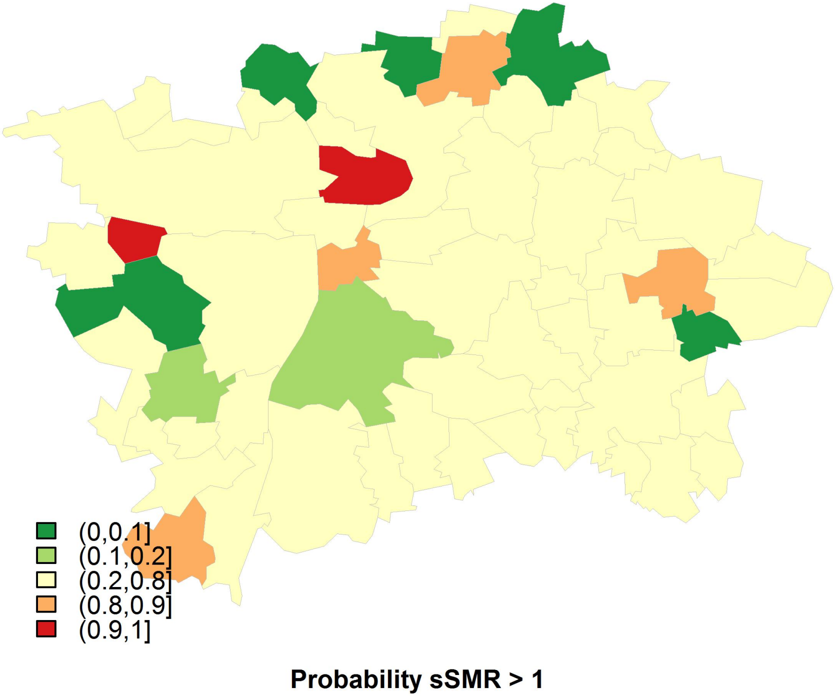

Prague, Males, 2003 - 2007
Heart failure

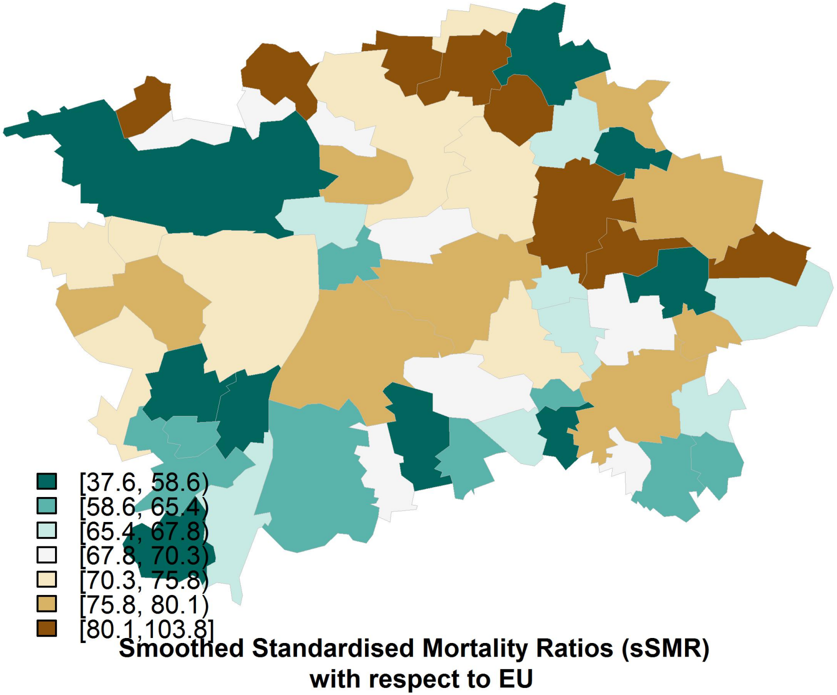

Smoothed Standardised Mortality Ratios (sSMR)
with respect to EU

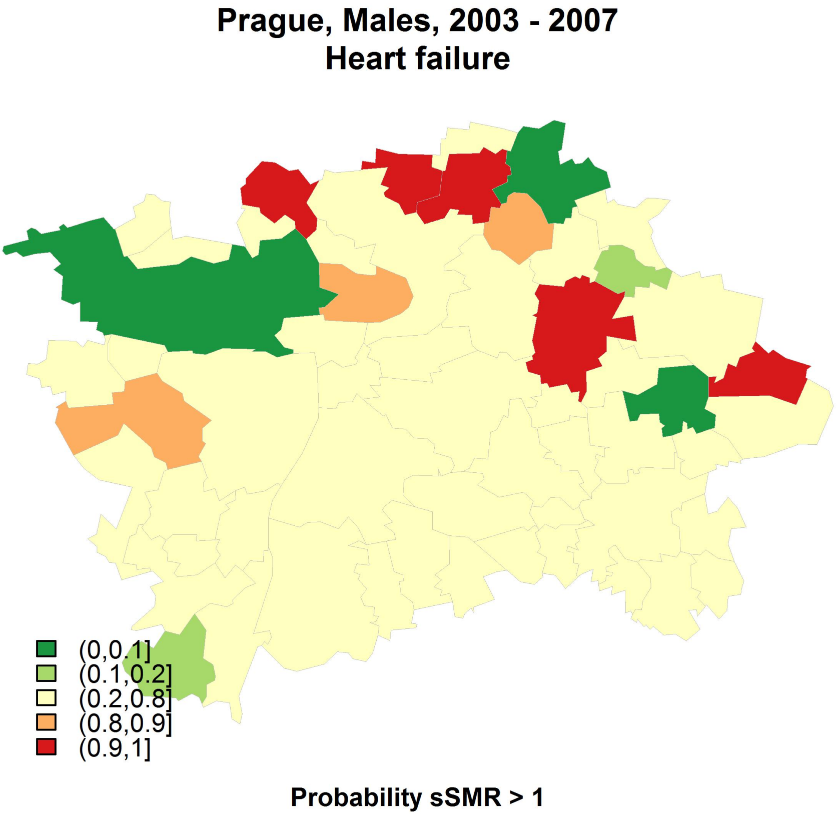
Prague, Males, 2003 - 2007
Heart failure
(0,0.1]
(0.1,0.2]
(0.2,0.8]
(0.8,0.9]
(0.9,1]
Probability sSMR > 1

Prague, Males, 2003 - 2007
Cerebrovascular diseases

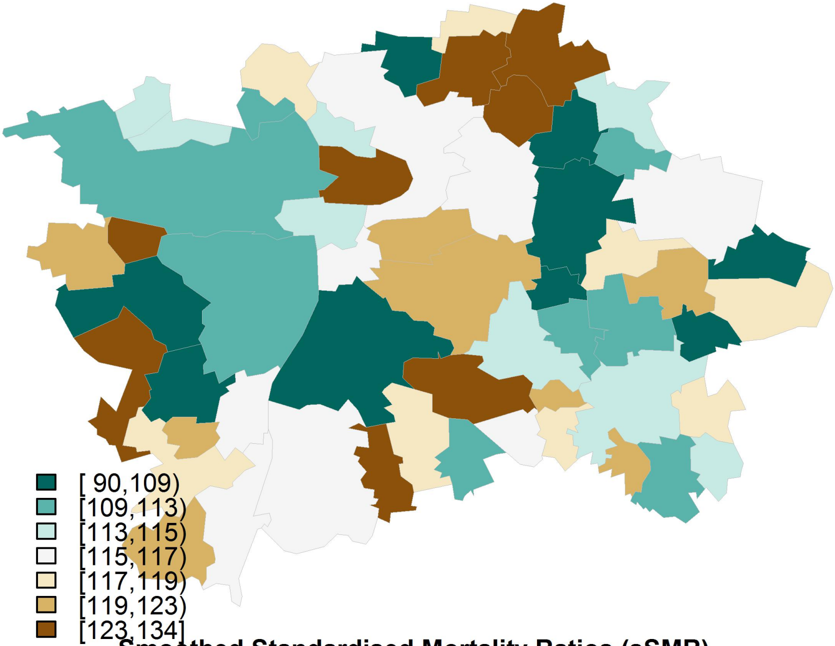

Smoothed Standardised Mortality Ratios (sSMR)
with respect to EU

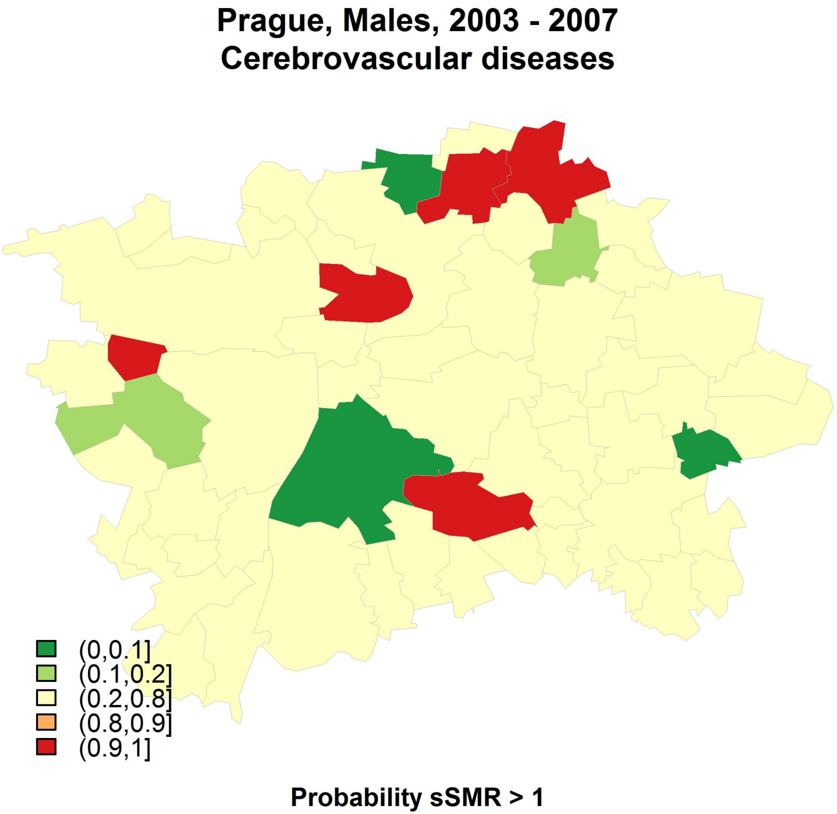

Prague, Males, 2003 - 2007
Cerebrovascular diseases
(0,0.1]
(0.1,0.2]
(0.2,0.8]
(0.8,0.9]
(0.9,1]
Probability sSMR > 1

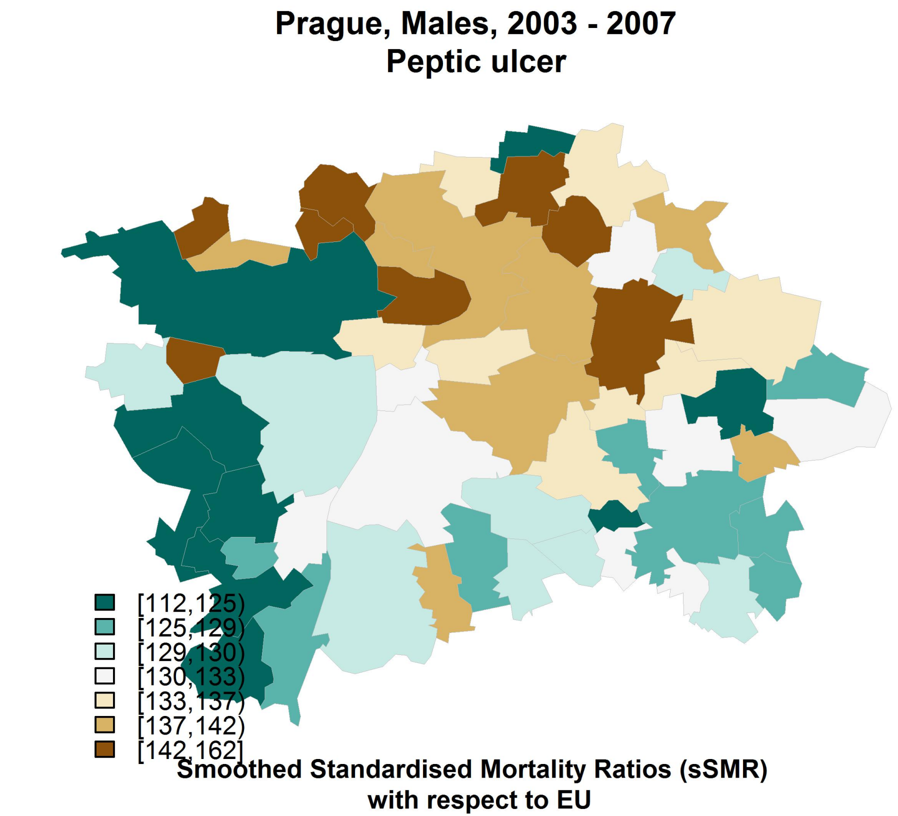
Prague, Males, 2003 - 2007
Peptic ulcer
[112,125)
[125,129)
[129,130)
[130,133)
[133,137)
[137,142)
[142,162]
Smoothed Standardised Mortality Ratios (sSMR)
with respect to EU

Prague, Males, 2003 - 2007
Peptic ulcer

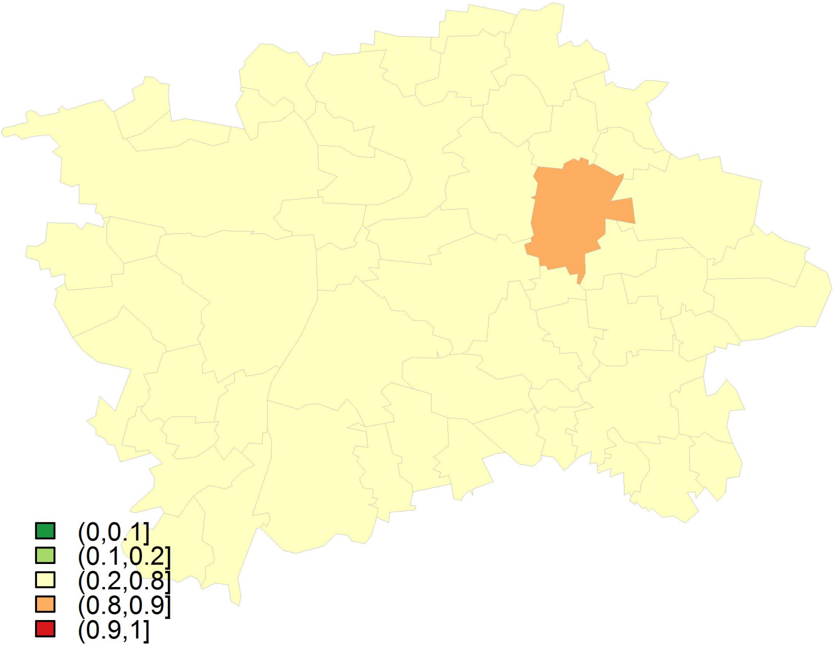

Probability sSMR > 1

Prague, Males, 2003 - 2007
Renal failure

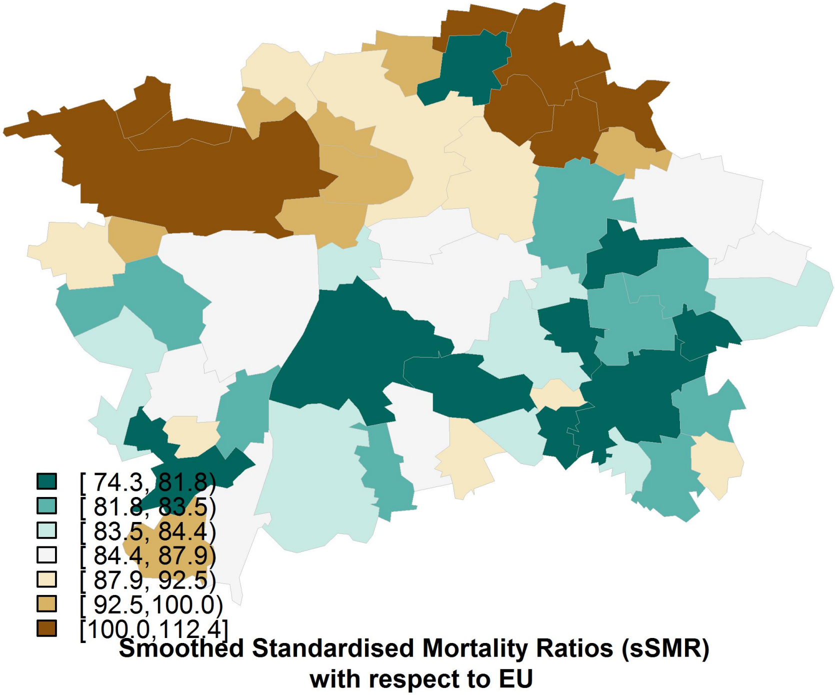

Smoothed Standardised Mortality Ratios (sSMR)
with respect to EU

Prague, Males, 2003 - 2007
Renal failure

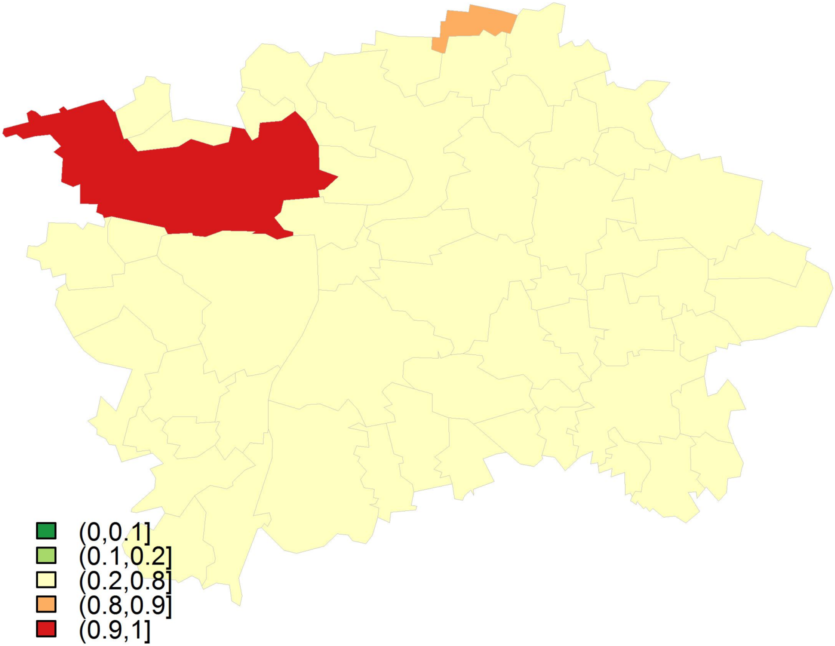

Probability sSMR > 1

Prague, Males, 2003 - 2007
Conditions originating in the perinatal period

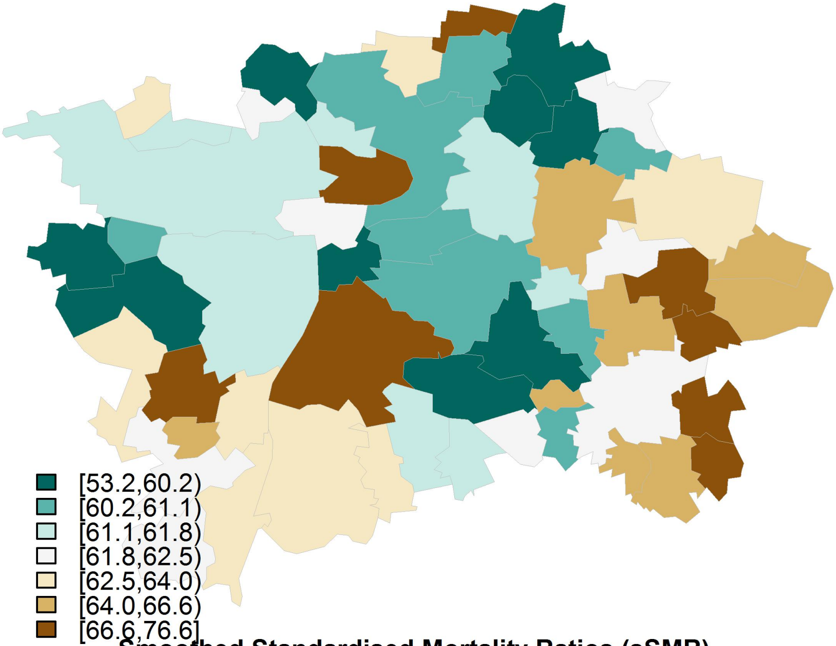

Smoothed Standardised Mortality Ratios (sSMR)
with respect to EU

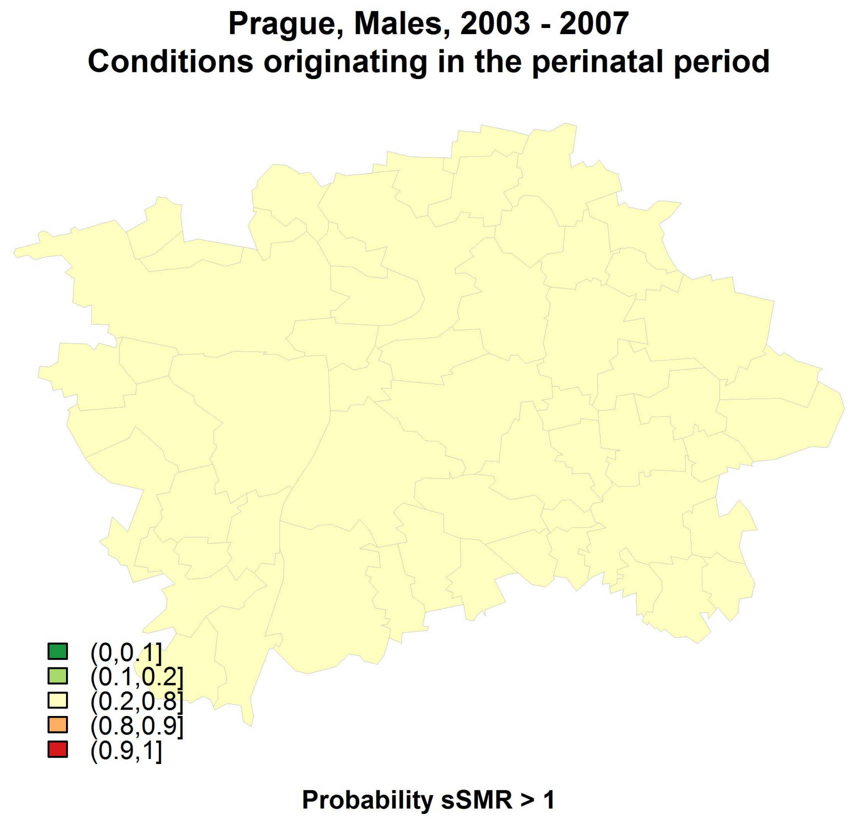
Prague, Males, 2003 - 2007
Conditions originating in the perinatal period
(0,0.1]
(0.1,0.2]
(0.2,0.8]
(0.8,0.9]
(0.9,1]
Probability sSMR > 1

Prague, Females, 2003 - 2007
MN colon

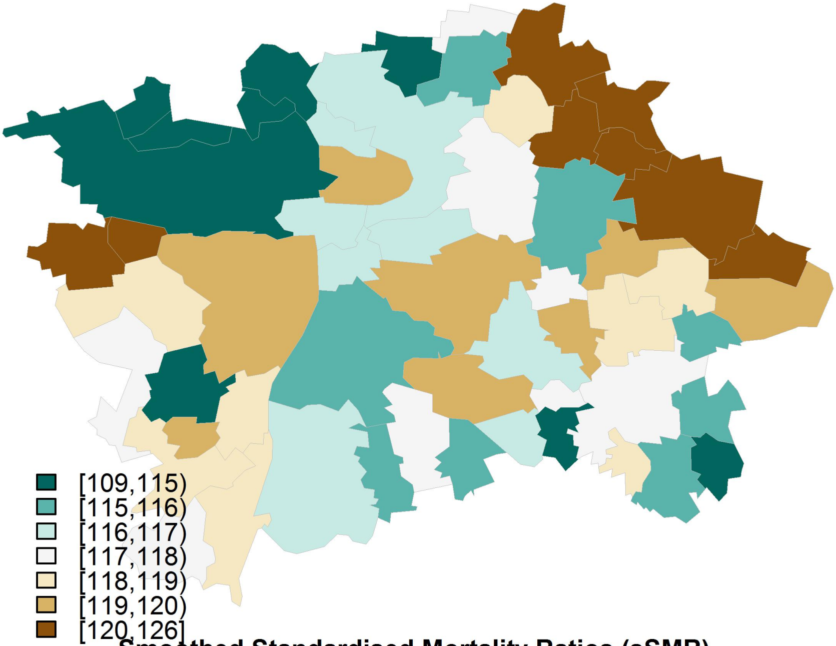

Smoothed Standardised Mortality Ratios (sSMR)
with respect to EU

Prague, Females, 2003 - 2007
MN colon

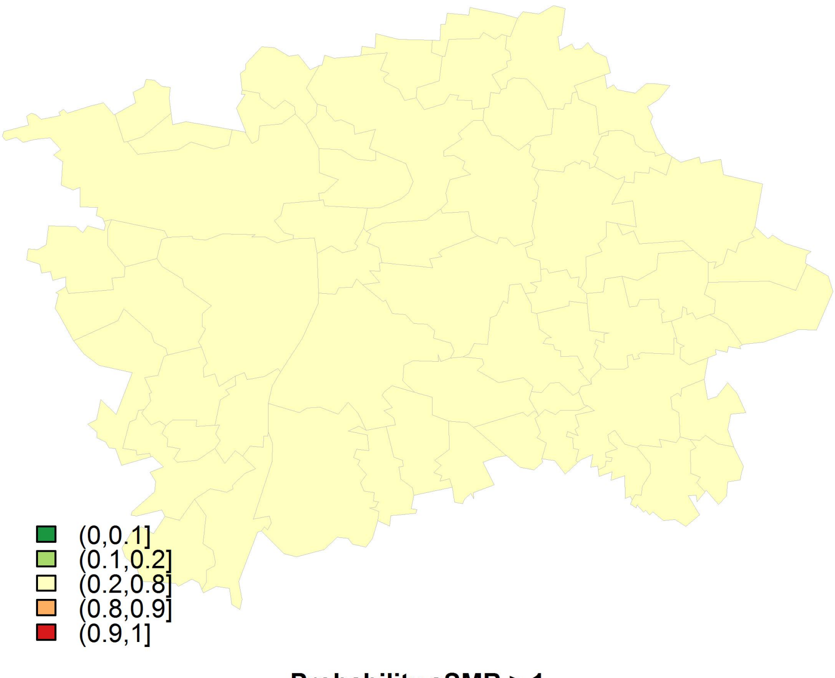

Probability sSMR > 1

Prague, Females, 2003 - 2007
MN rectum, anus and anal canal

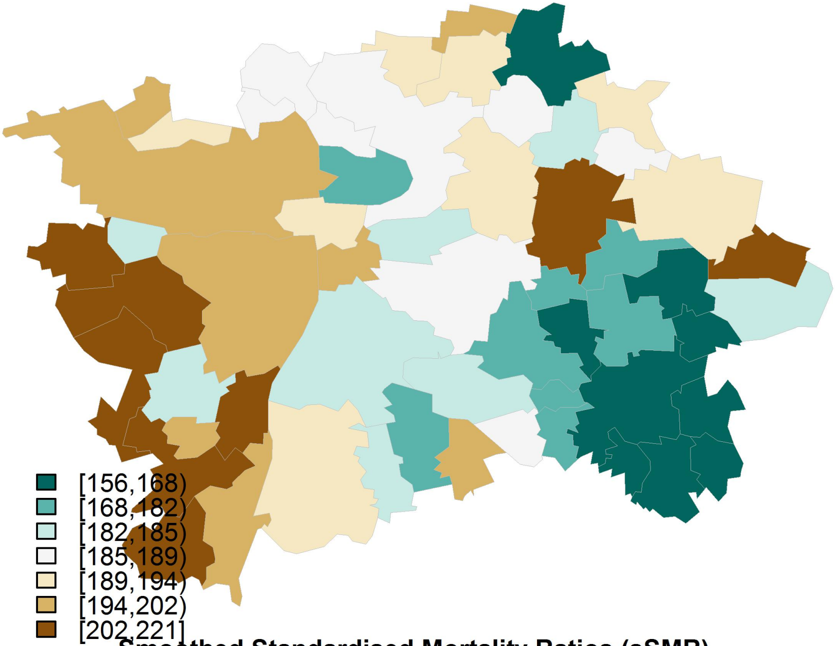

Smoothed Standardised Mortality Ratios (sSMR)
with respect to EU

Prague, Females, 2003 - 2007
MN rectum, anus and anal canal

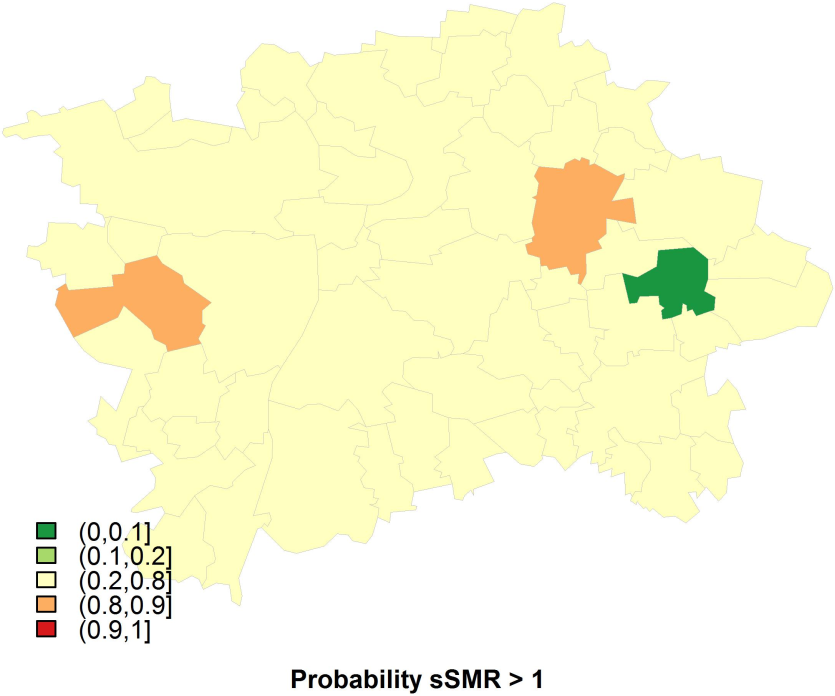

Prague, Females, 2003 - 2007
MN cervix uteri

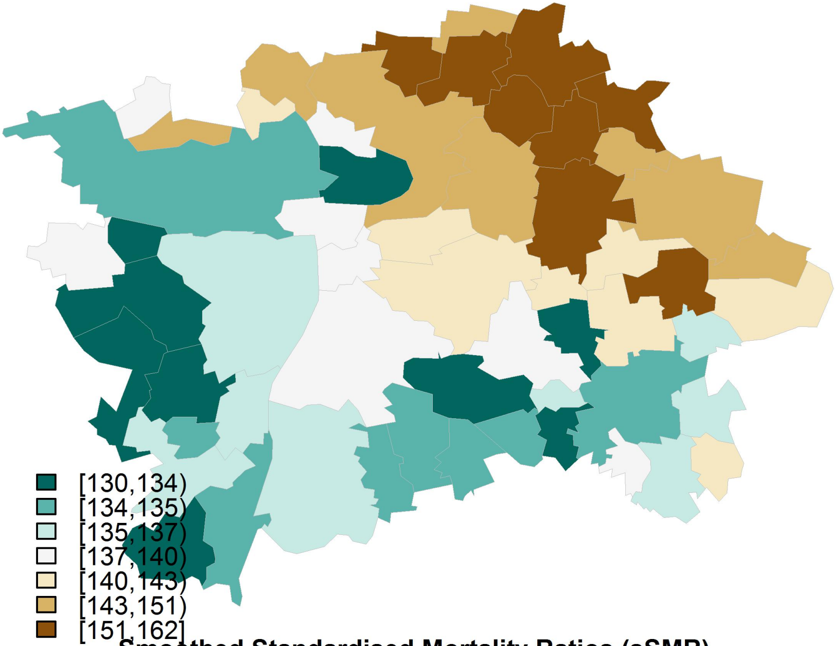

Smoothed Standardised Mortality Ratios (sSMR)
with respect to EU

Prague, Females, 2003 - 2007
MN cervix uteri

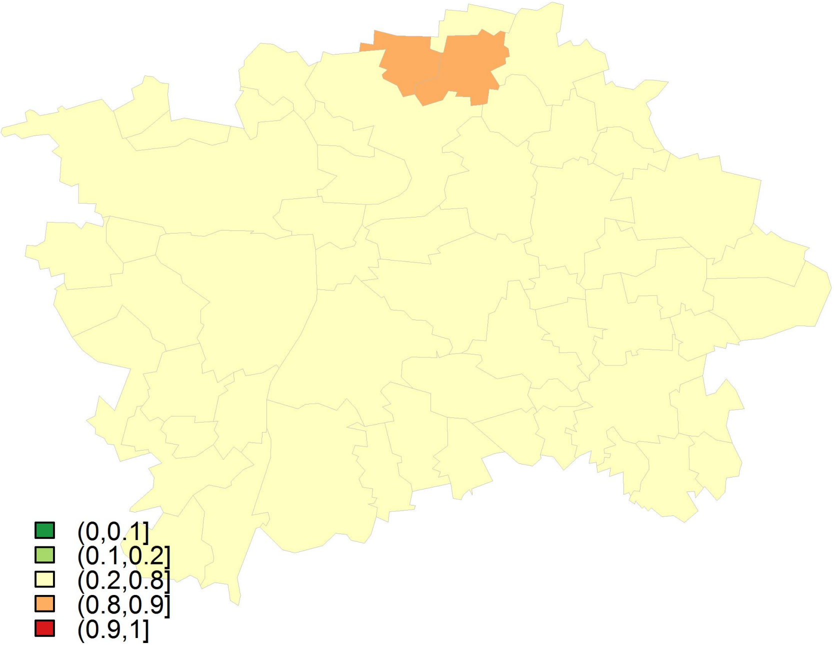

Probability sSMR > 1

Prague, Females, 2003 - 2007
Hodgkin's disease

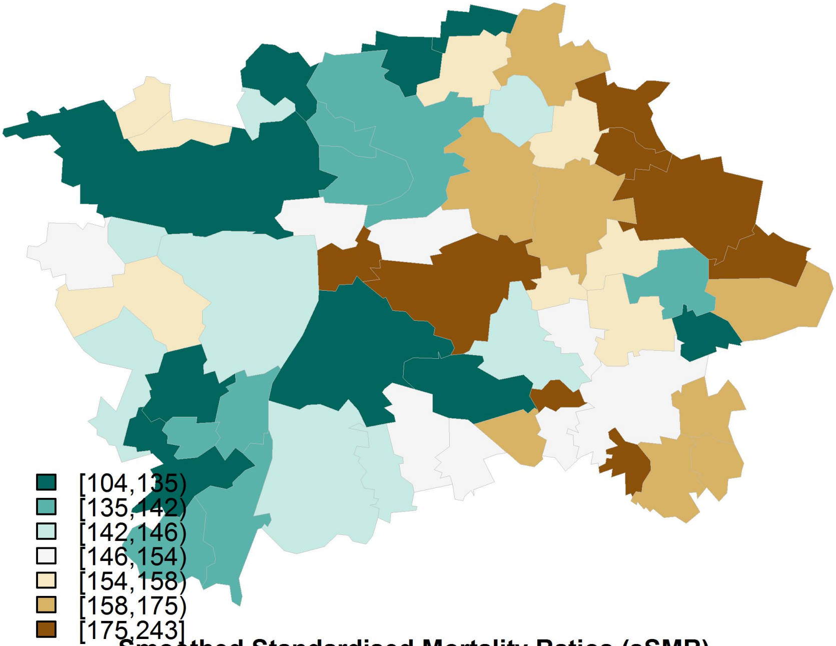

Smoothed Standardised Mortality Ratios (sSMR)
with respect to EU

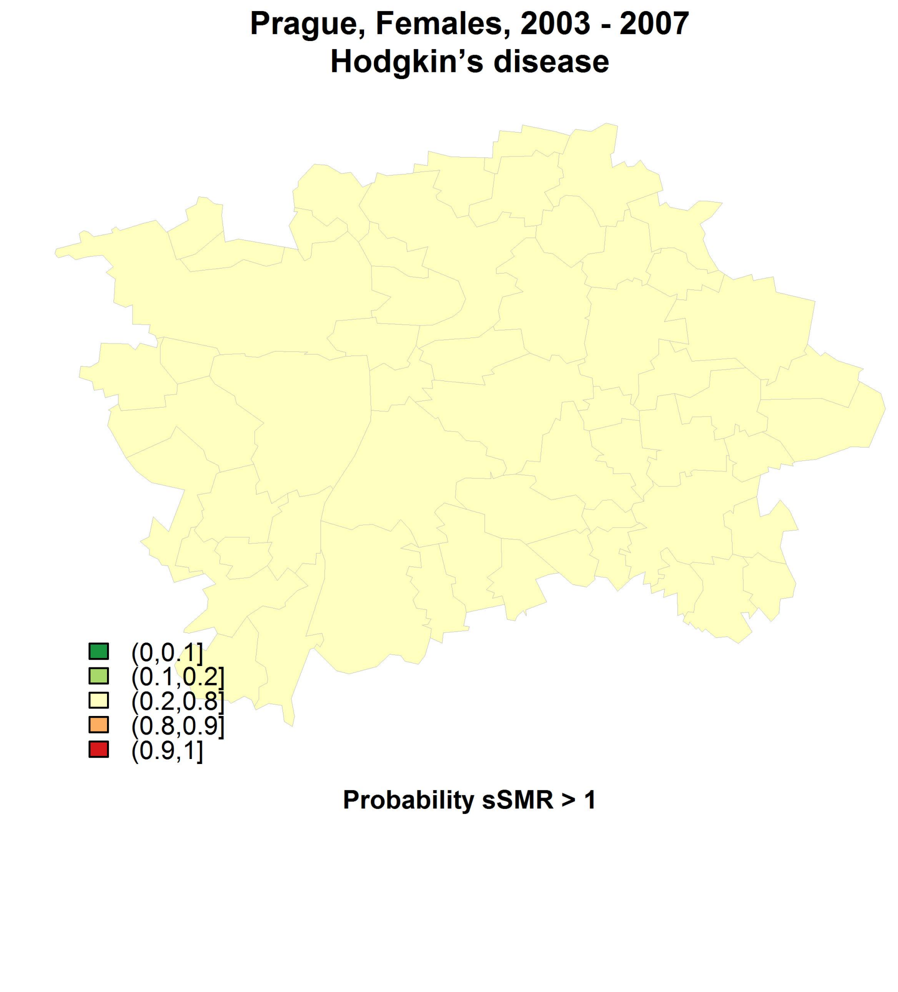
Prague, Females, 2003 - 2007
Hodgkin’s disease
(0,0.1]
(0.1,0.2]
(0.2,0.8]
(0.8,0.9]
(0.9,1]
Probability sSMR > 1

Prague, Females, 2003 - 2007
Rheumatic heart disease

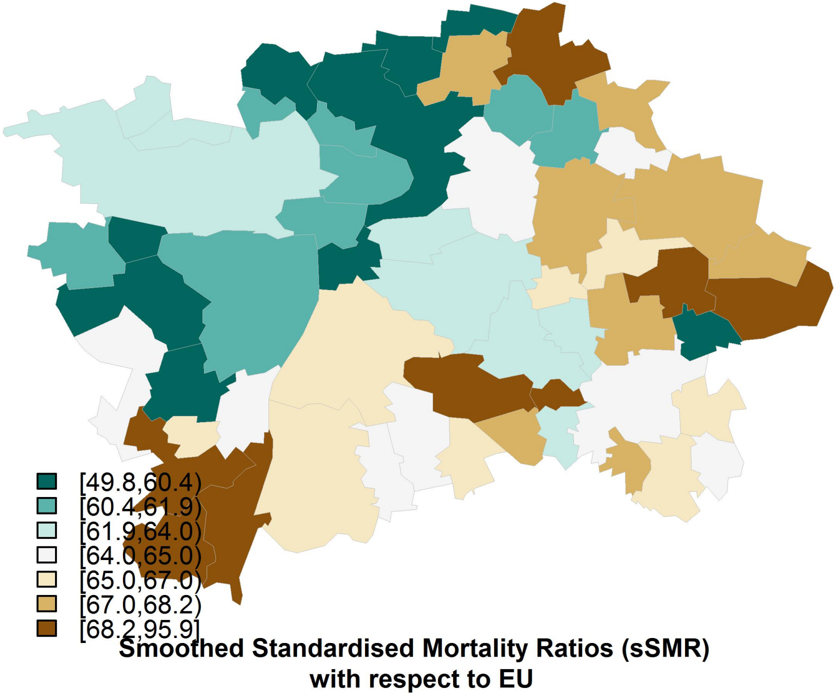

Smoothed Standardised Mortality Ratios (sSMR)
with respect to EU

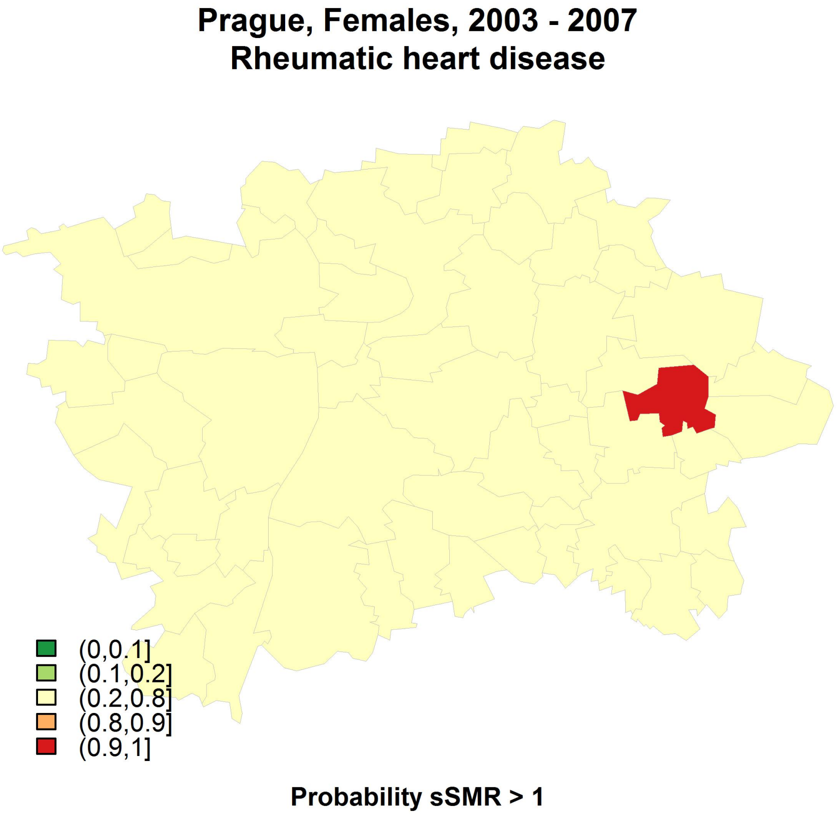

Prague, Females, 2003 - 2007
Rheumatic heart disease
(0,0.1]
(0.1,0.2]
(0.2,0.8]
(0.8,0.9]
(0.9,1]
Probability sSMR > 1

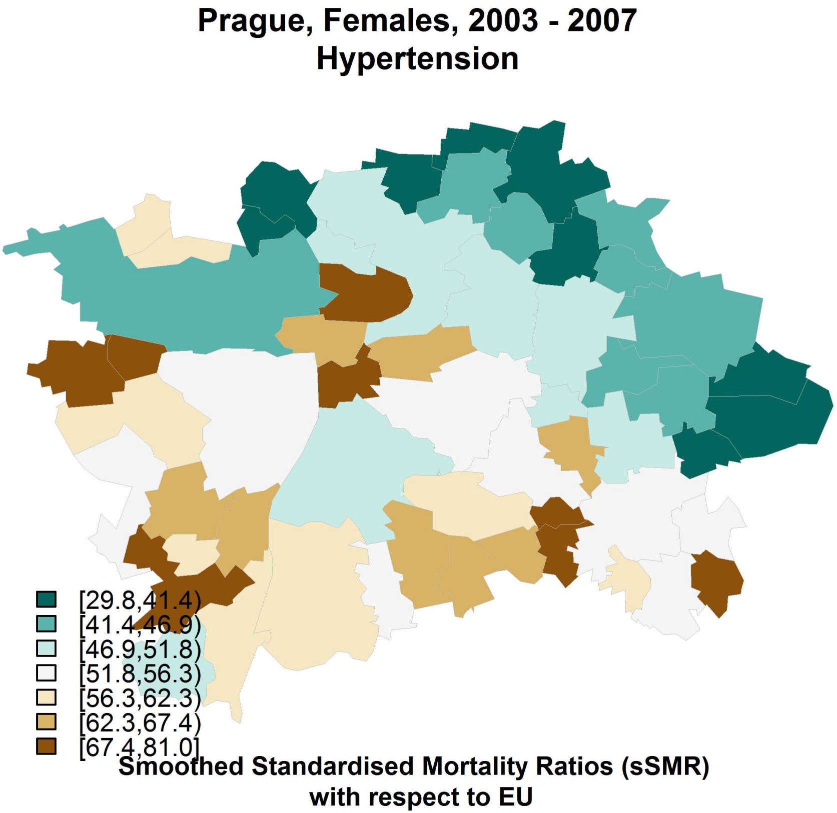
Prague, Females, 2003 - 2007
Hypertension
[29.8,41.4)
[41.4,46.9)
[46.9,51.8)
[51.8,56.3)
[56.3,62.3)
[62.3,67.4)
[67.4,81.0]
Smoothed Standardised Mortality Ratios (sSMR)
with respect to EU

Prague, Females, 2003 - 2007
Hypertension

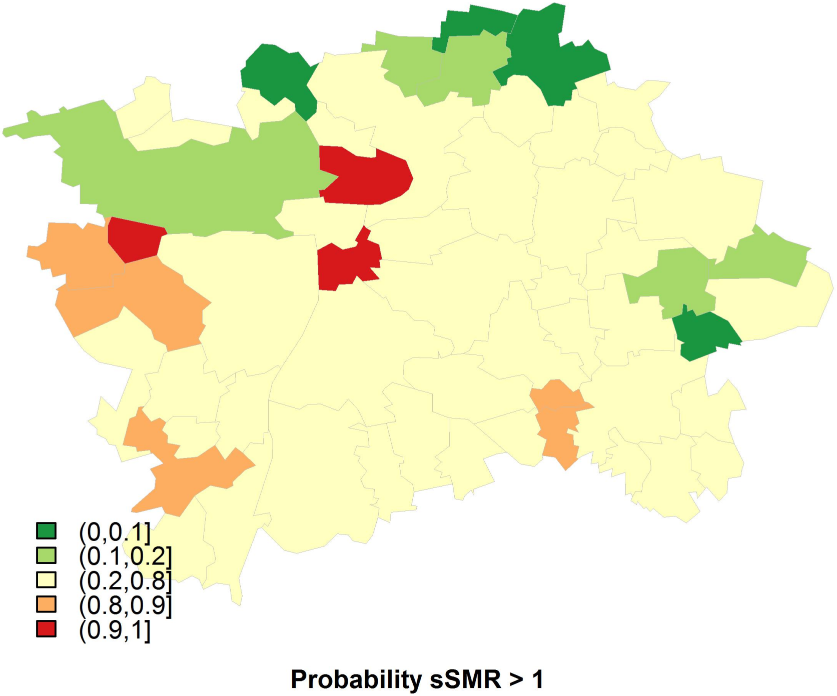

Prague, Females, 2003 - 2007
Heart failure

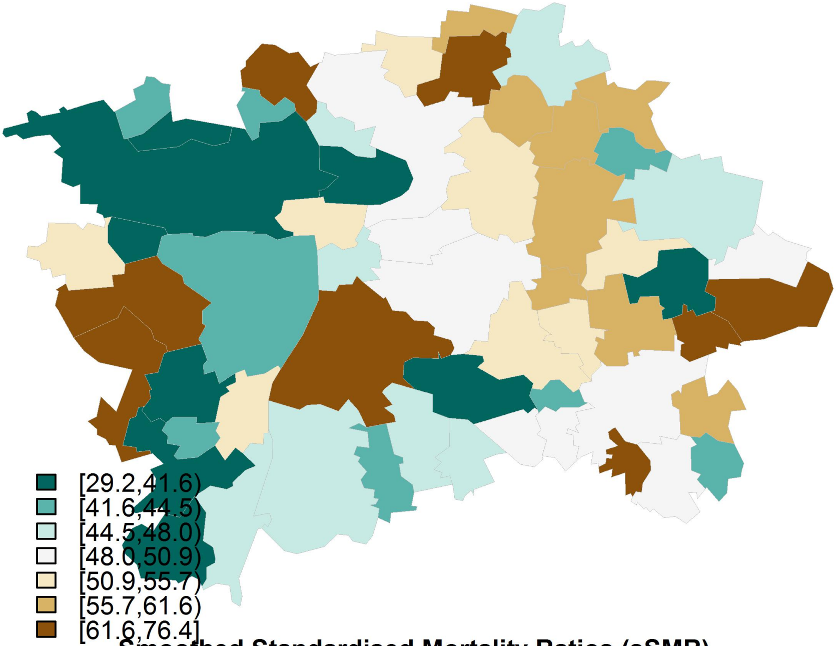

Smoothed Standardised Mortality Ratios (sSMR)
with respect to EU

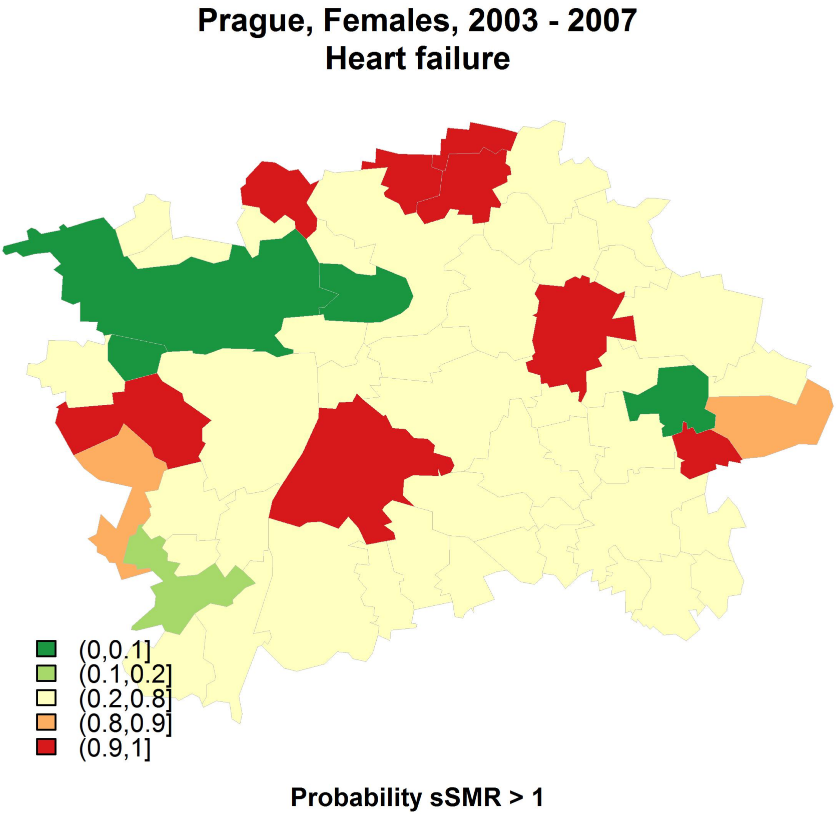
Prague, Females, 2003 - 2007
Heart failure
(0,0.1]
(0.1,0.2]
(0.2,0.8]
(0.8,0.9]
(0.9,1]
Probability sSMR > 1

Prague, Females, 2003 - 2007
Cerebrovascular diseases

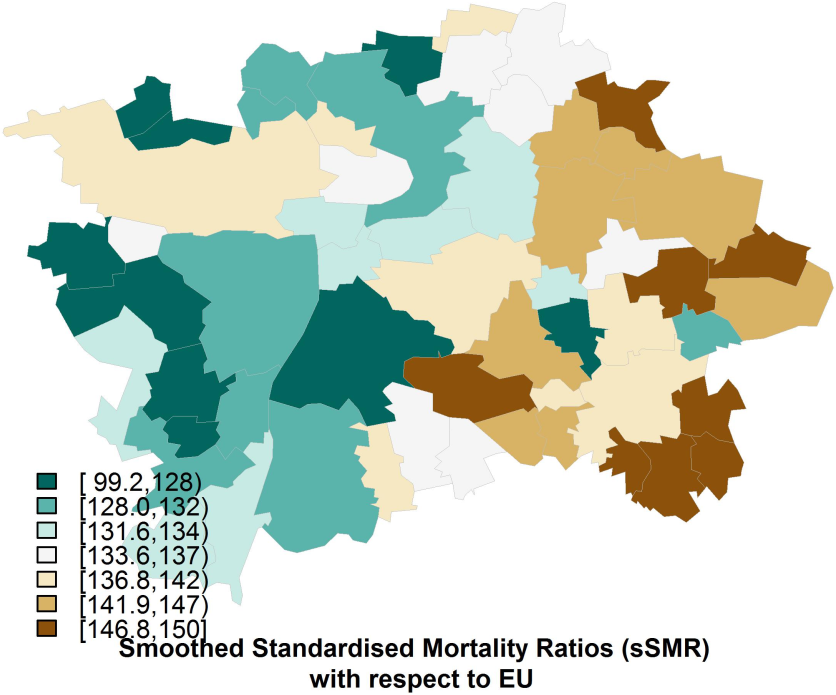

Smoothed Standardised Mortality Ratios (sSMR)
with respect to EU

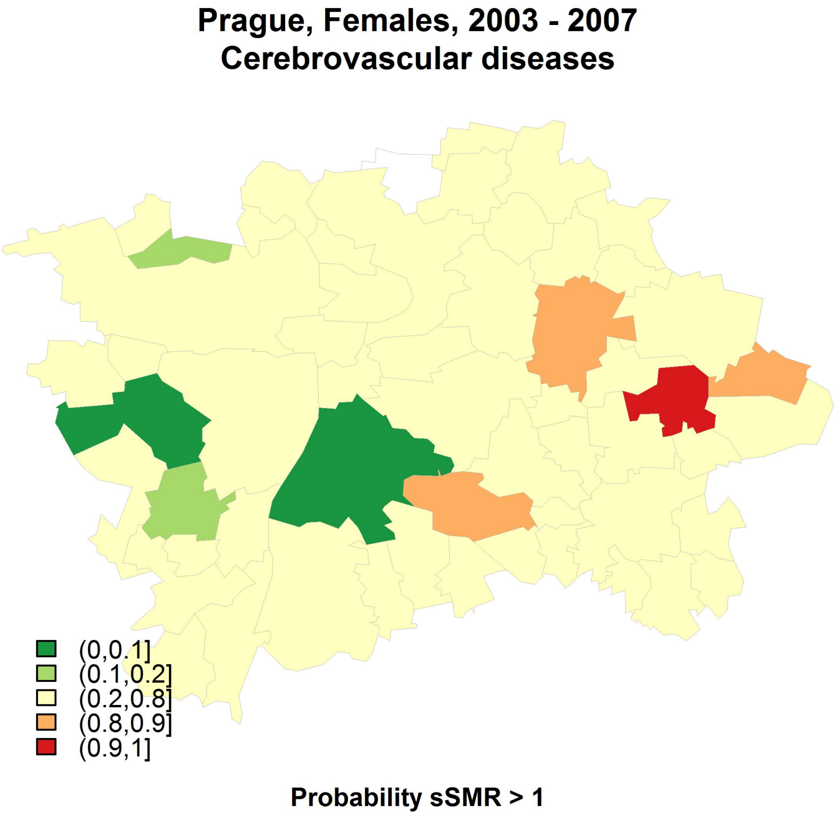
Prague, Females, 2003 - 2007
Cerebrovascular diseases
(0,0.1]
(0.1,0.2]
(0.2,0.8]
(0.8,0.9]
(0.9,1]
Probability sSMR > 1

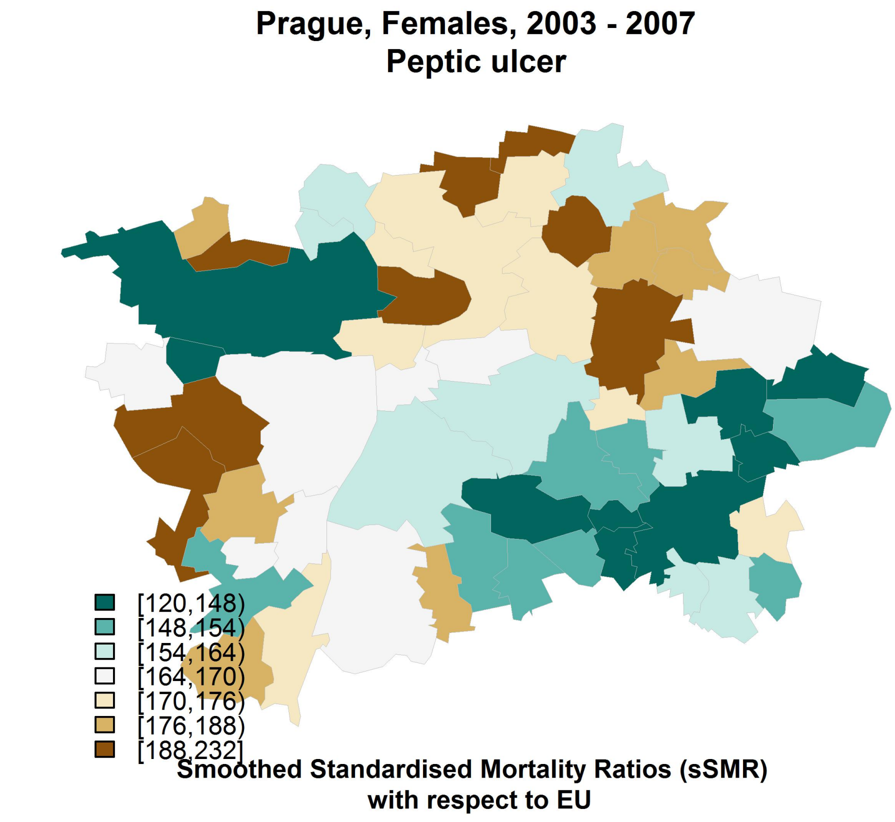
Prague, Females, 2003 - 2007
Peptic ulcer
[120,148)
[148,154)
[154,164)
[164,170)
[170,176)
[176,188)
[188,232]
Smoothed Standardised Mortality Ratios (sSMR)
with respect to EU

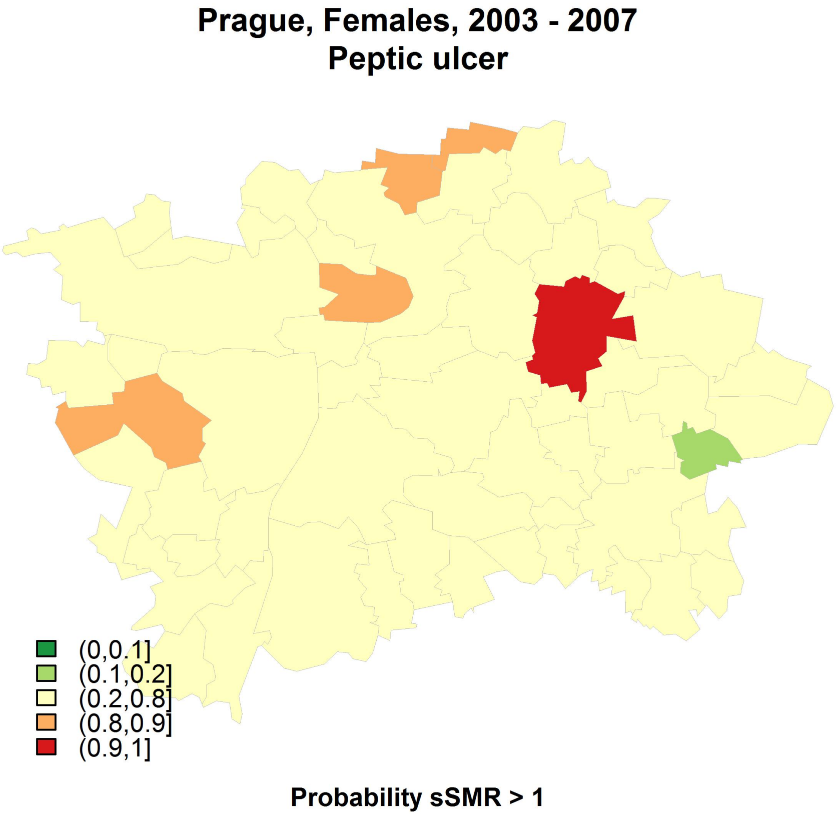
Prague, Females, 2003 - 2007
Peptic ulcer
(0,0.1]
(0.1,0.2]
(0.2,0.8]
(0.8,0.9]
(0.9,1]
Probability sSMR > 1

Prague, Females, 2003 - 2007
Renal failure

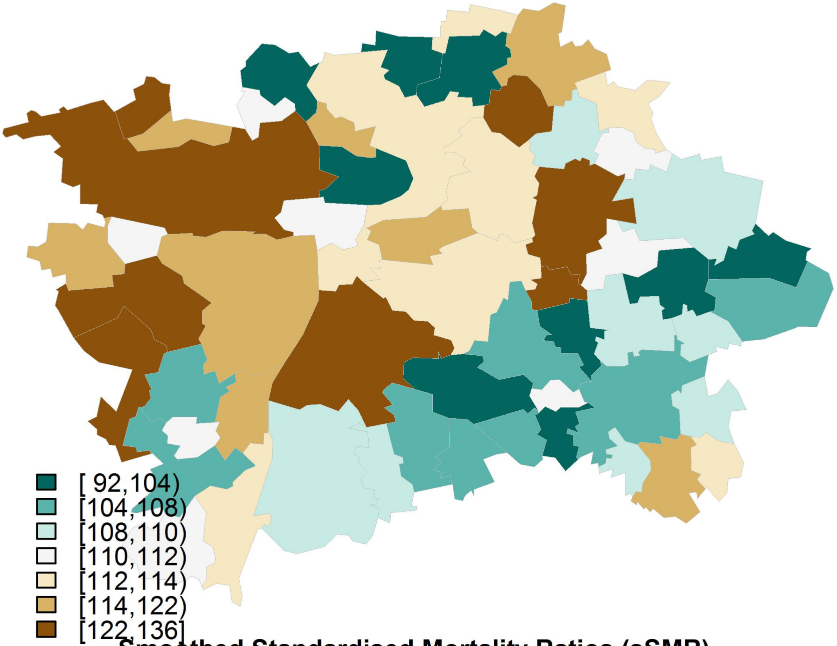

Smoothed Standardised Mortality Ratios (sSMR)
with respect to EU

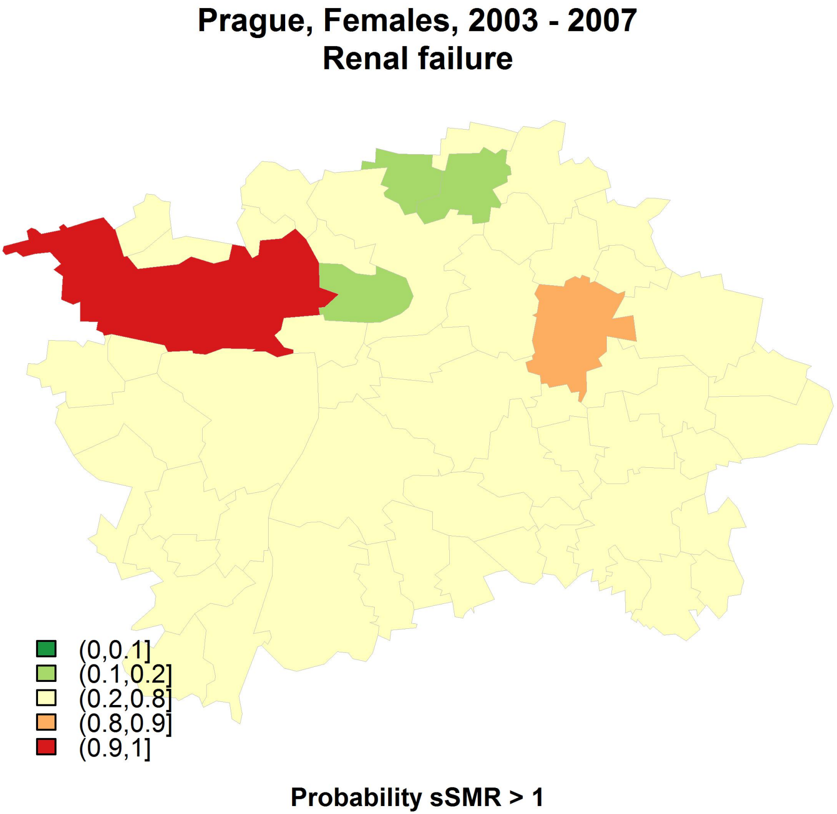
Prague, Females, 2003 - 2007
Renal failure
(0,0.1]
(0.1,0.2]
(0.2,0.8]
(0.8,0.9]
(0.9,1]
Probability sSMR > 1

Prague, Females, 2003 - 2007
Conditions originating in the perinatal period

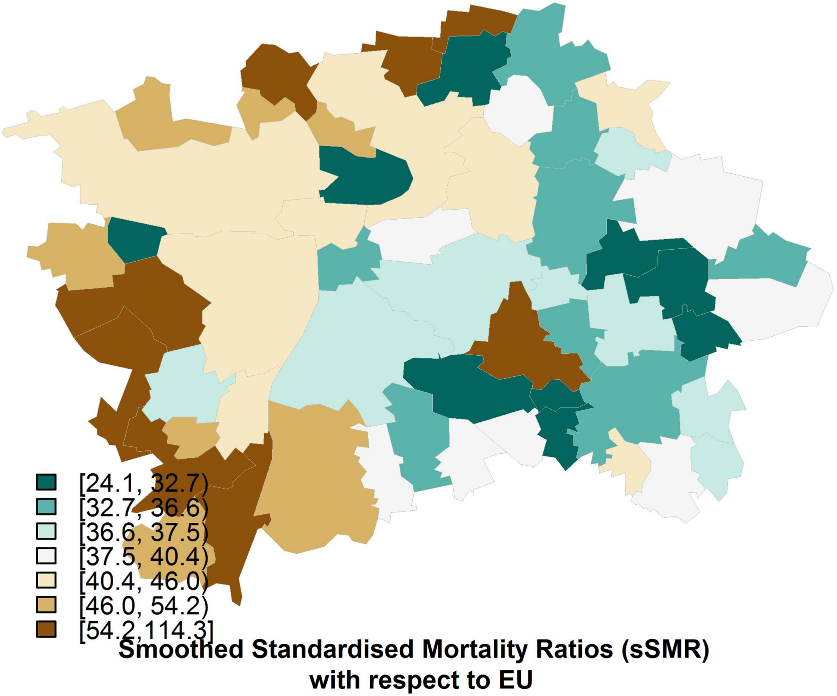

Prague, Females, 2003 - 2007
Conditions originating in the perinatal period

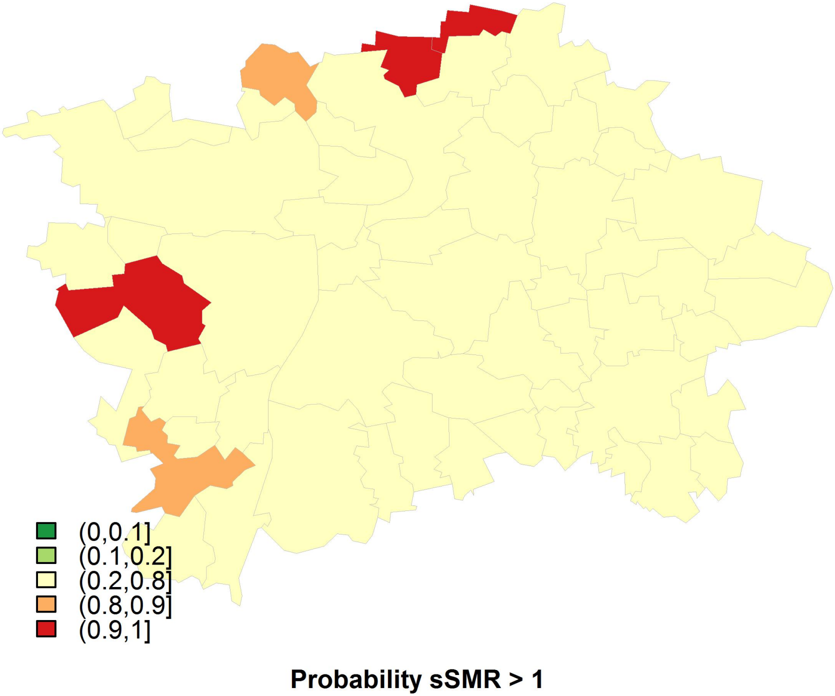

Supplement: Additional file 13 — Cause-specific mortality maps for Prague. [file 1476-072X-13-8-S13.pdf]
