# Supplementary material for: Social differences in avoidable mortality between small areas of 15 European cities: an ecological study
Source: Int J Health Geogr. 2014 Mar 12;13:8. doi: 10.1186/1476-072X-13-8 (PMC4007807; doi:10.1186/1476-072X-13-8)

Rotterdam, Males, 1996 - 2008
AIDS (HIV disease)

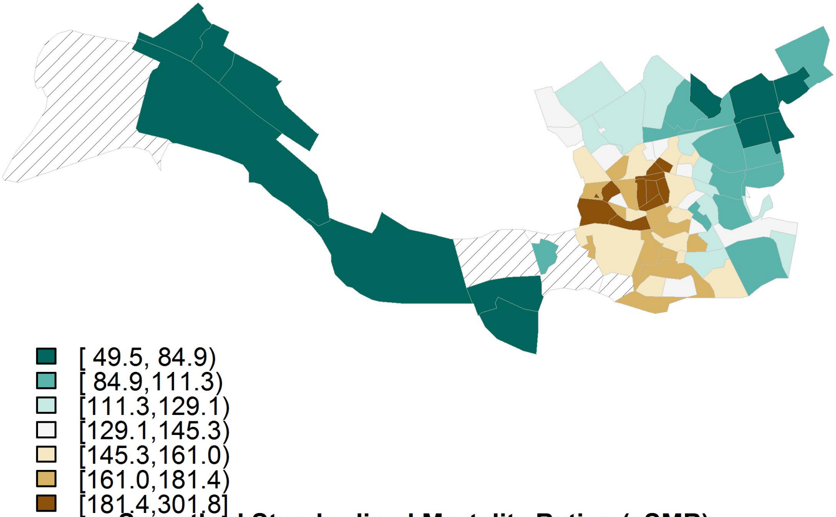

Smoothed Standardised Mortality Ratios (sSMR)
with respect to EU

Rotterdam, Males, 1996 - 2008
AIDS (HIV disease)

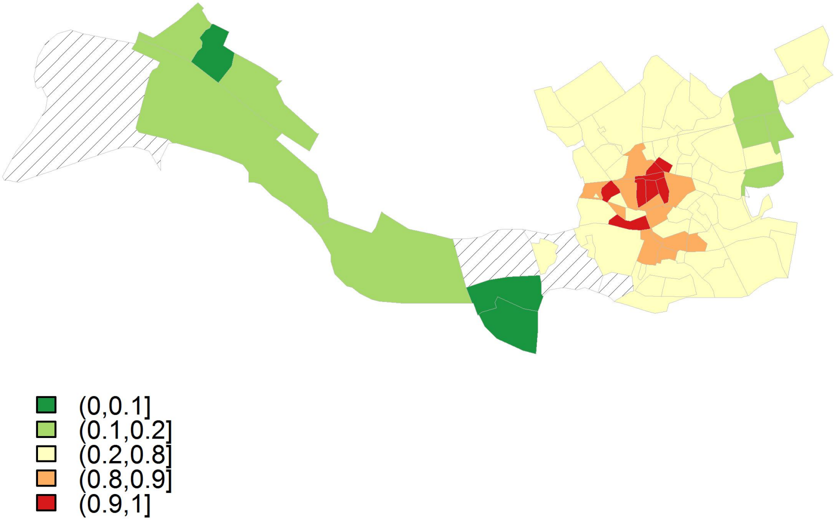

Probability sSMR > 1

Rotterdam, Males, 1996 - 2008
MN colon

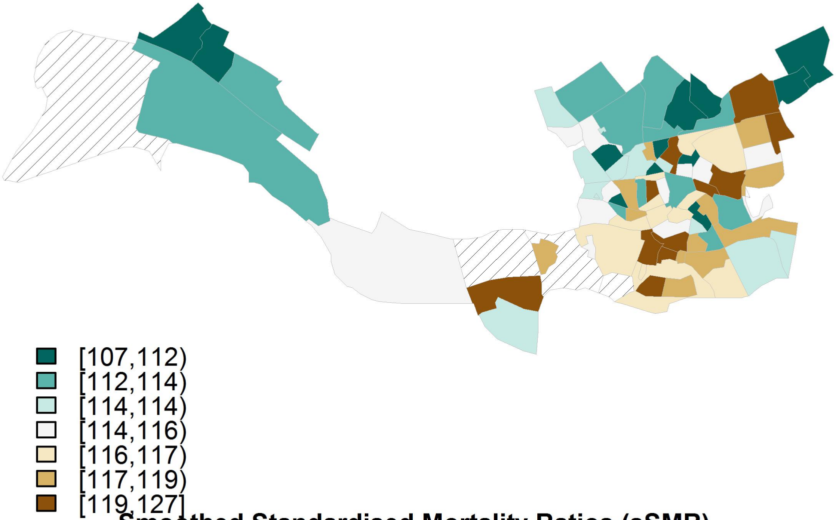

Smoothed Standardised Mortality Ratios (sSMR)
with respect to EU

Rotterdam, Males, 1996 - 2008
MN colon

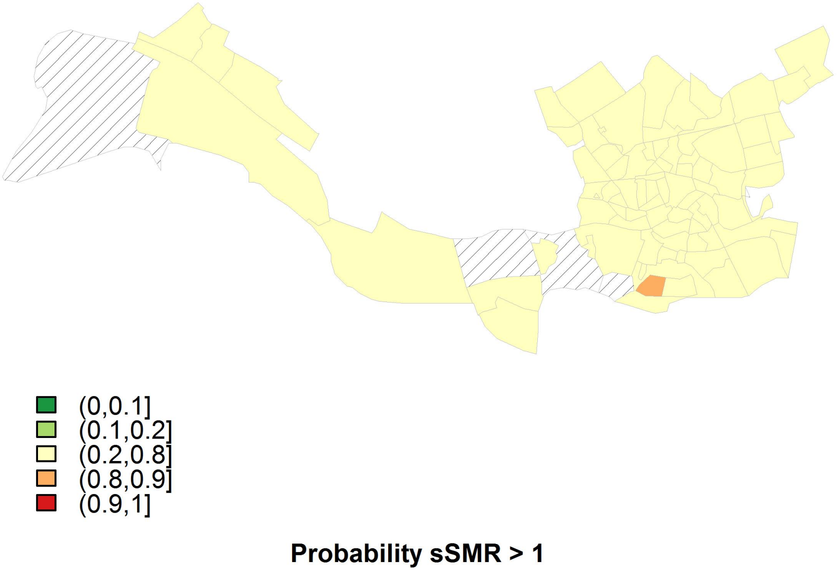

Rotterdam, Males, 1996 - 2008
MN rectum, anus and anal canal

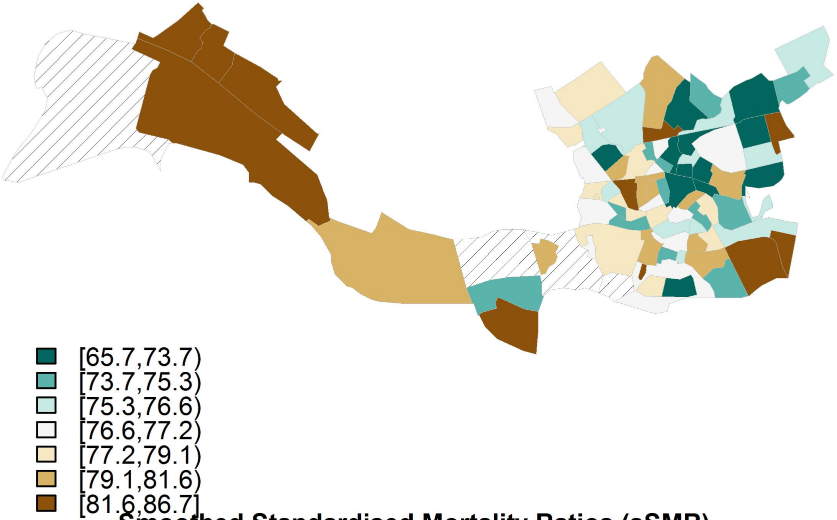

Smoothed Standardised Mortality Ratios (sSMR) with respect to EU

Rotterdam, Males, 1996 - 2008
MN rectum, anus and anal canal

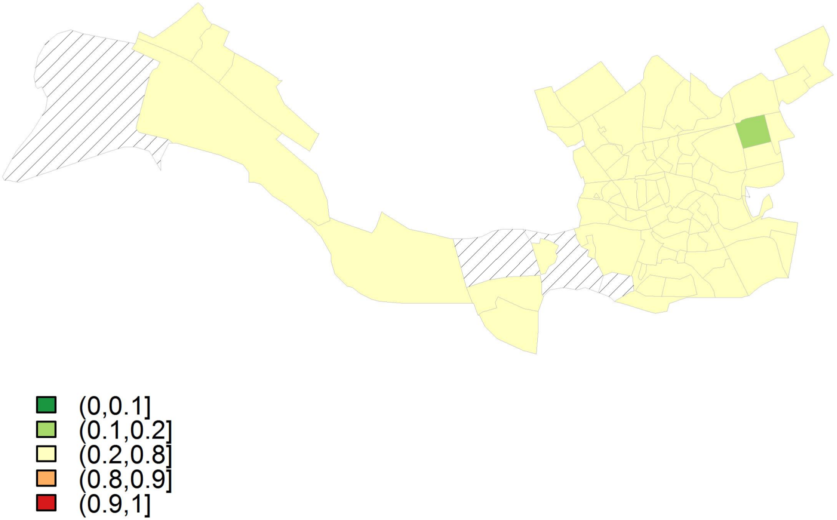

Probability sSMR > 1

Rotterdam, Males, 1996 - 2008
Hypertension

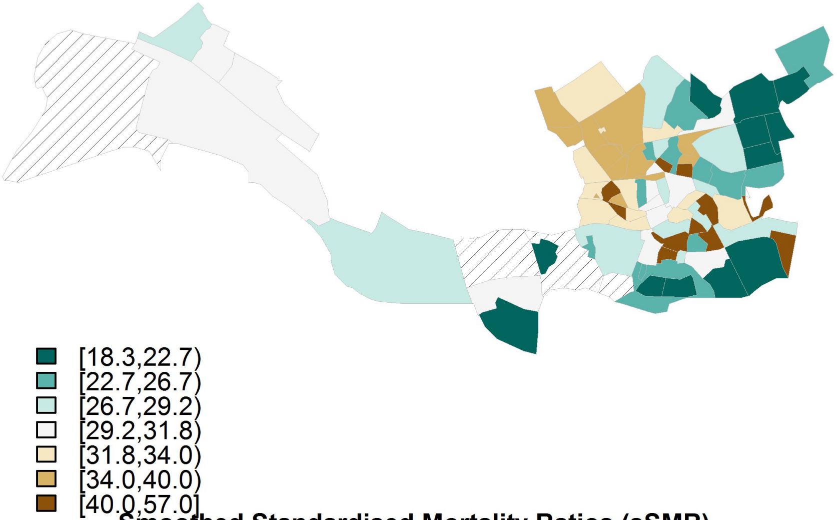

Smoothed Standardised Mortality Ratios (sSMR)
with respect to EU

Rotterdam, Males, 1996 - 2008
Hypertension

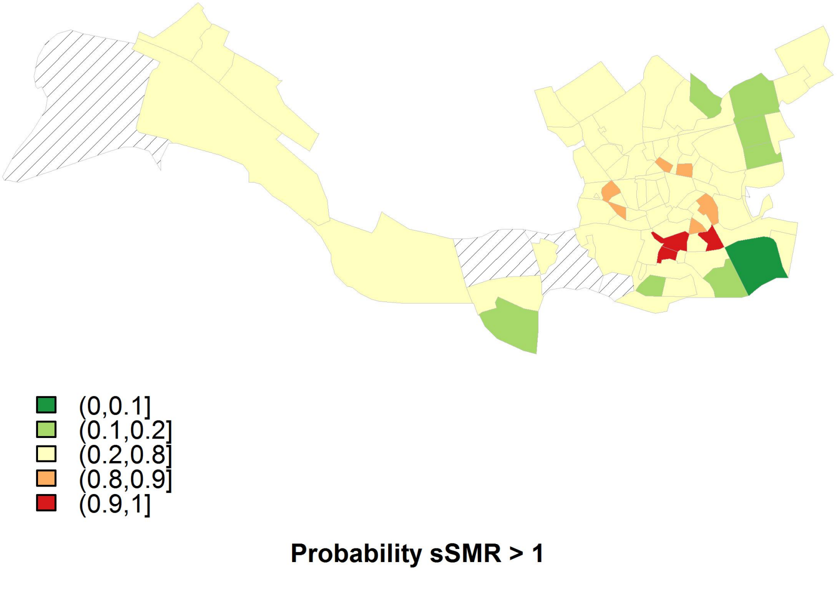

Rotterdam, Males, 1996 - 2008
Heart failure

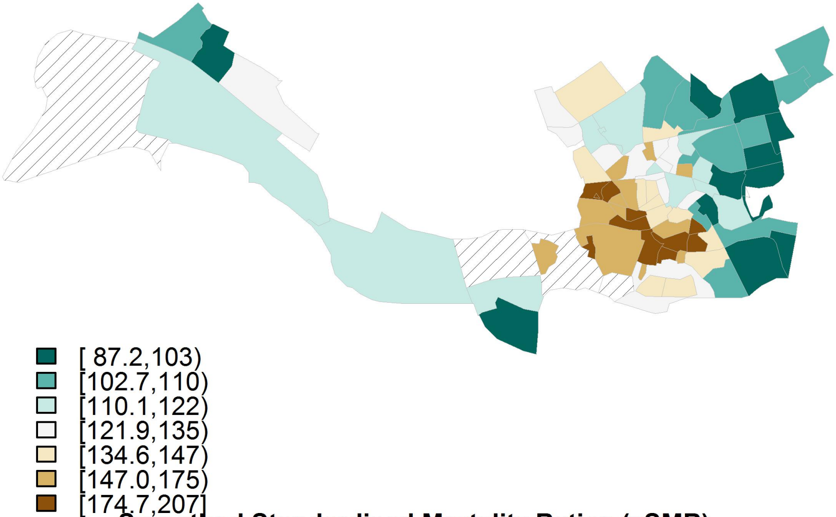

Smoothed Standardised Mortality Ratios (sSMR)
with respect to EU

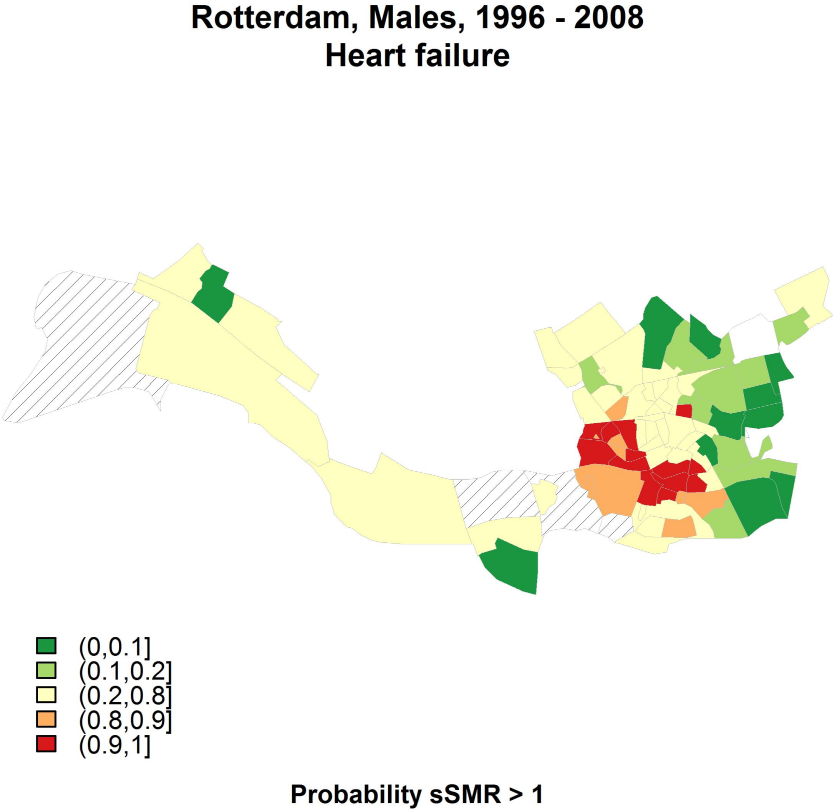
Rotterdam, Males, 1996 - 2008
Heart failure
(0,0.1]
(0.1,0.2]
(0.2,0.8]
(0.8,0.9]
(0.9,1]
Probability sSMR > 1

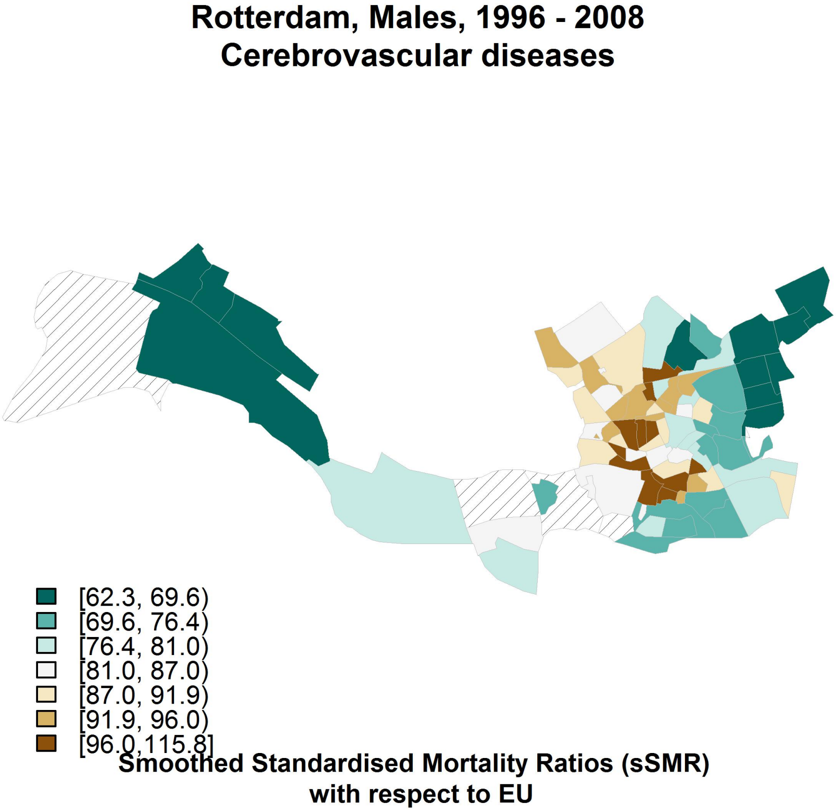
Rotterdam, Males, 1996 - 2008
Cerebrovascular diseases
[62.3, 69.6)
[69.6, 76.4)
[76.4, 81.0)
[81.0, 87.0)
[87.0, 91.9)
[91.9, 96.0)
[96.0,115.8]
Smoothed Standardised Mortality Ratios (sSMR)
with respect to EU

Rotterdam, Males, 1996 - 2008
Cerebrovascular diseases

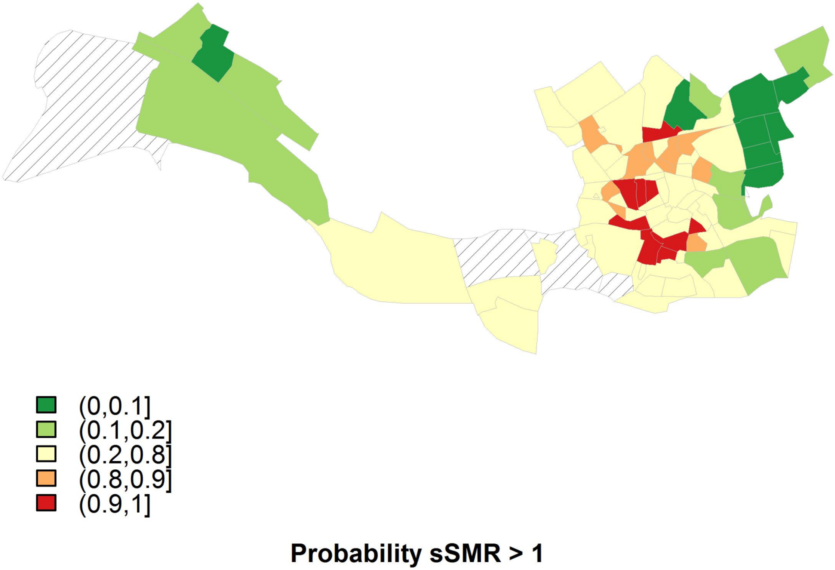

Rotterdam, Males, 1996 - 2008
Peptic ulcer

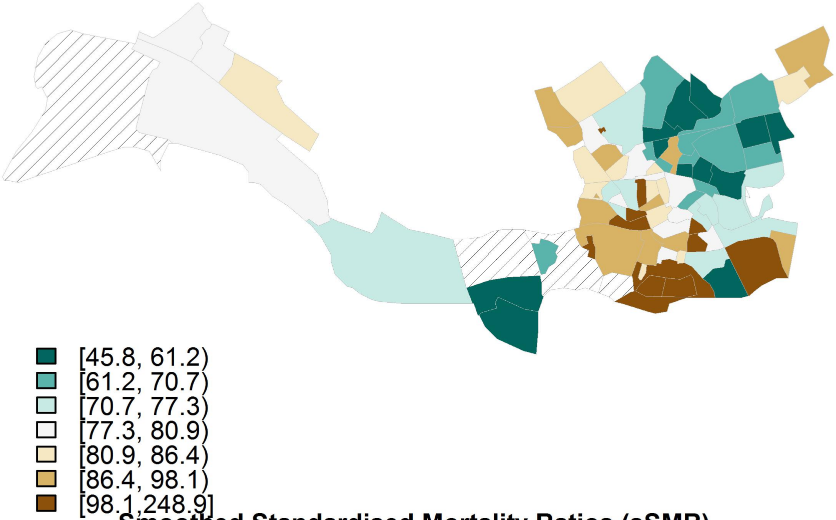

Smoothed Standardised Mortality Ratios (sSMR) with respect to EU

Rotterdam, Males, 1996 - 2008
Peptic ulcer

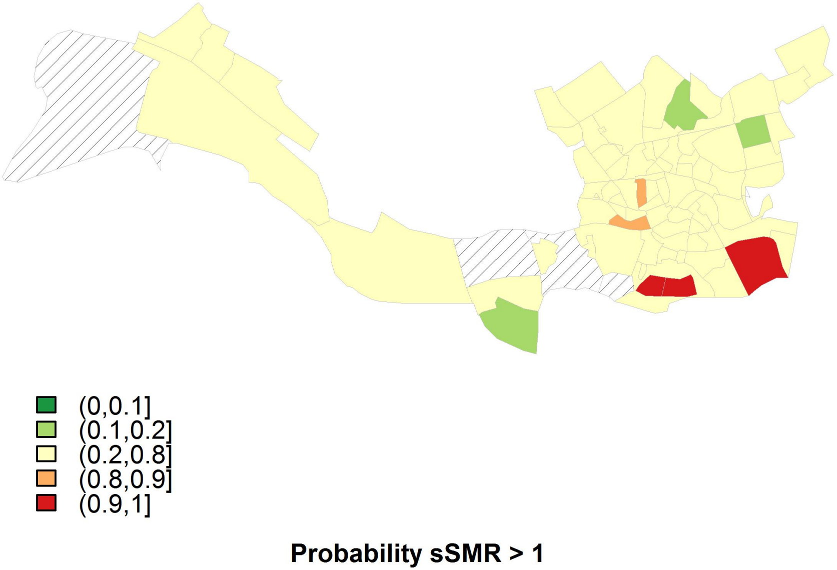

Rotterdam, Males, 1996 - 2008
Renal failure

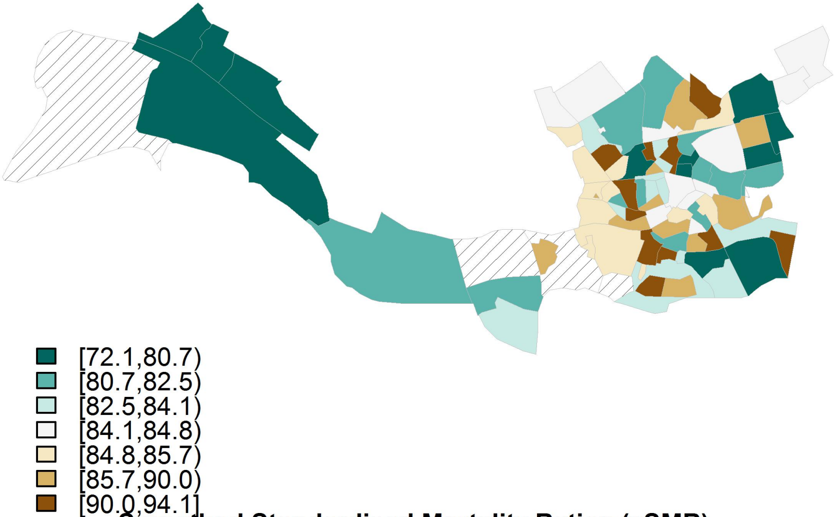

Smoothed Standardised Mortality Ratios (sSMR) with respect to EU

Rotterdam, Males, 1996 - 2008
Renal failure

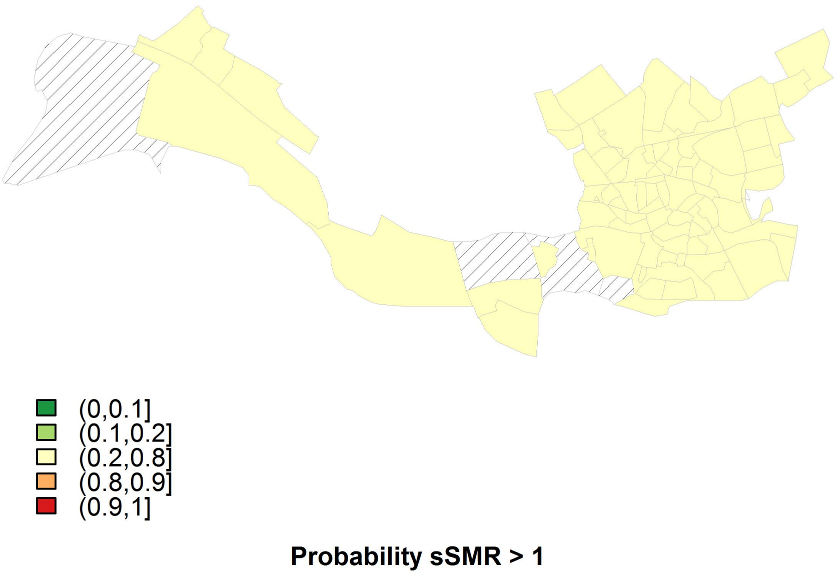

Rotterdam, Males, 1996 - 2008
Conditions originating in the perinatal period

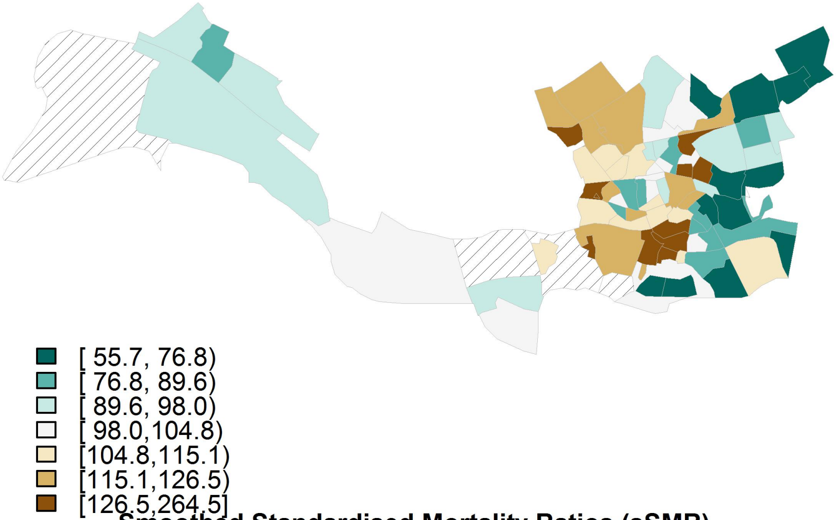

**Smoothed Standardised Mortality Ratios (sSMR)
with respect to EU**

Rotterdam, Males, 1996 - 2008
Conditions originating in the perinatal period

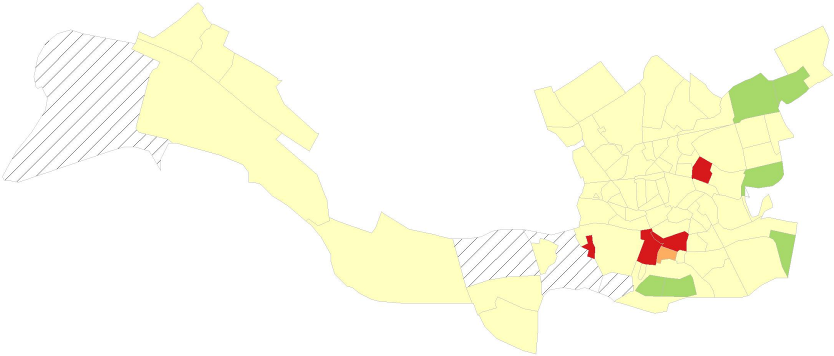

(0,0.1]
(0.1,0.2]
(0.2,0.8]
(0.8,0.9]
(0.9,1]

Probability sSMR > 1

Rotterdam, Males, 1996 - 2008
Congenital heart disease

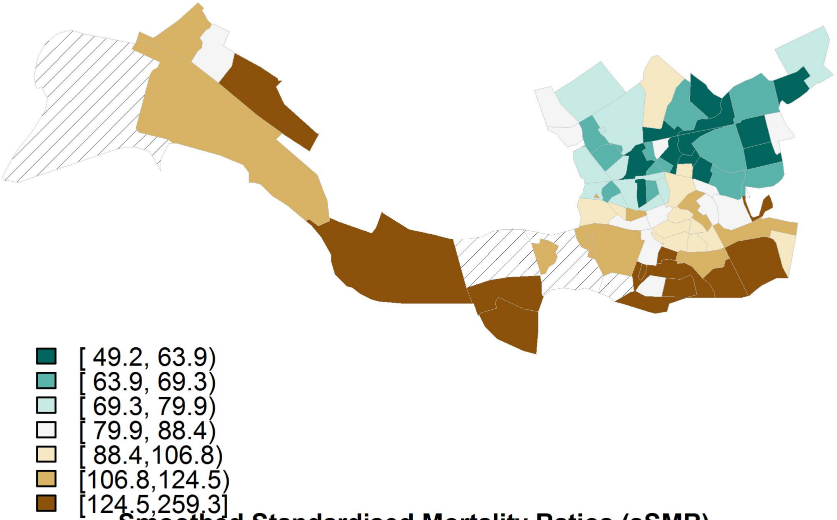

Smoothed Standardised Mortality Ratios (sSMR) with respect to EU

Rotterdam, Males, 1996 - 2008
Congenital heart disease

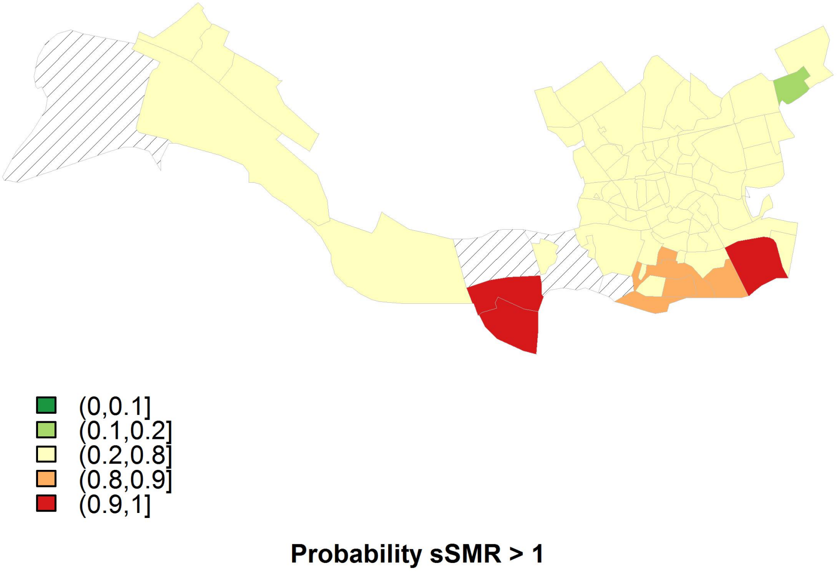

Rotterdam, Females, 1996 - 2008
AIDS (HIV disease)

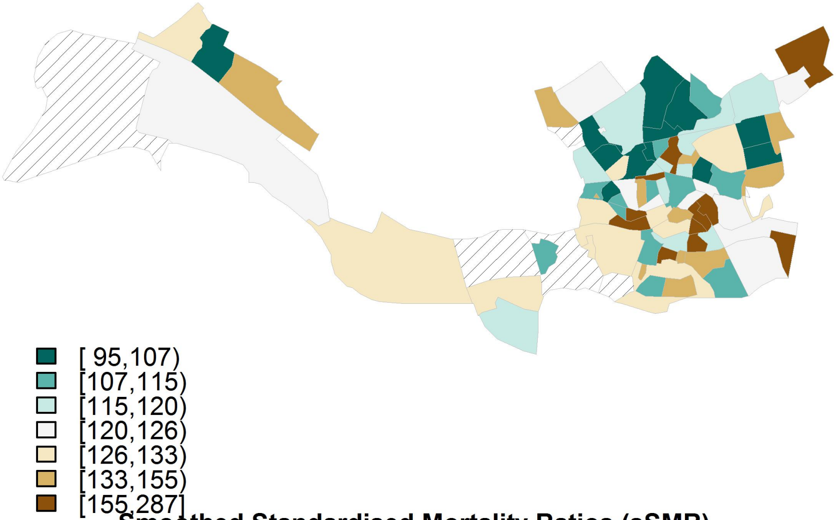

**Smoothed Standardised Mortality Ratios (sSMR)
with respect to EU**

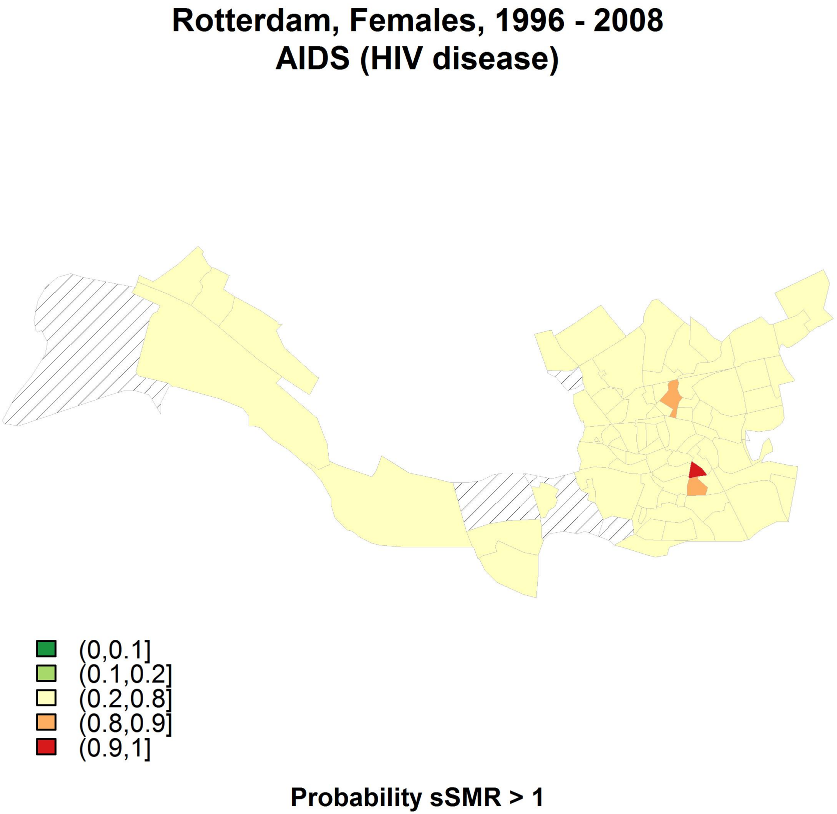

Rotterdam, Females, 1996 - 2008
AIDS (HIV disease)
(0,0.1]
(0.1,0.2]
(0.2,0.8]
(0.8,0.9]
(0.9,1]
Probability sSMR > 1

Rotterdam, Females, 1996 - 2008
MN colon

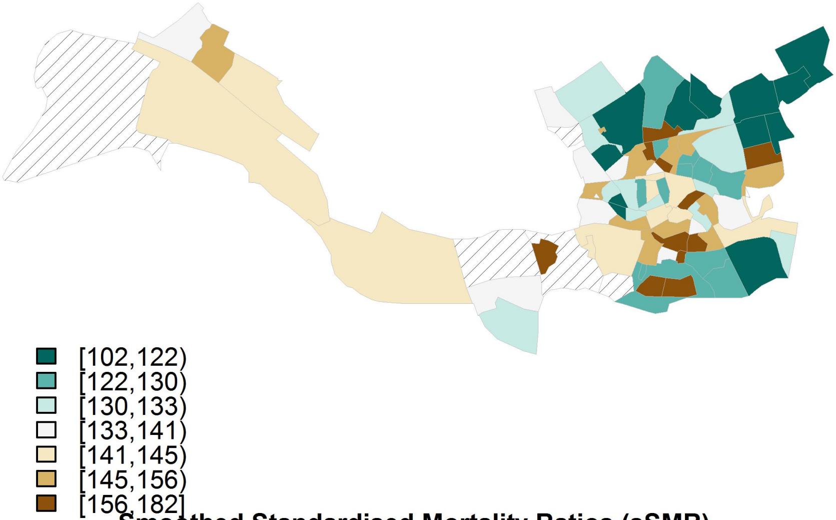

Smoothed Standardised Mortality Ratios (sSMR)
with respect to EU

Rotterdam, Females, 1996 - 2008
MN colon

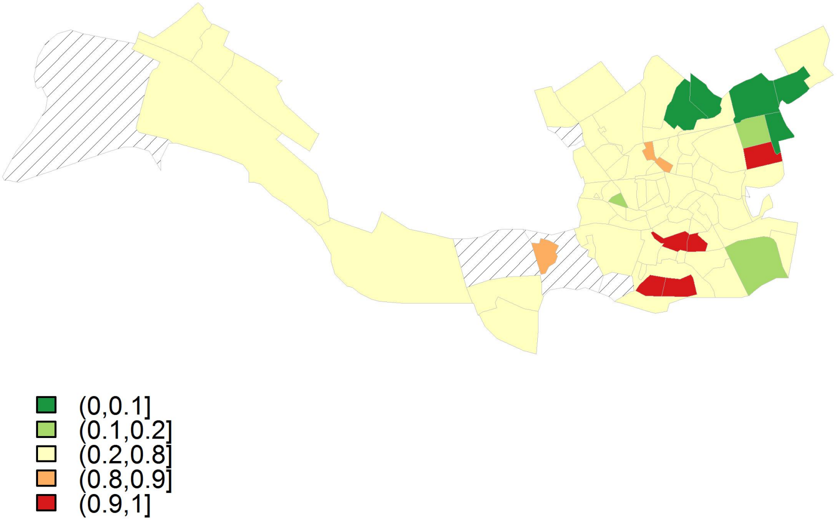

Probability sSMR > 1

Rotterdam, Females, 1996 - 2008
MN rectum, anus and anal canal

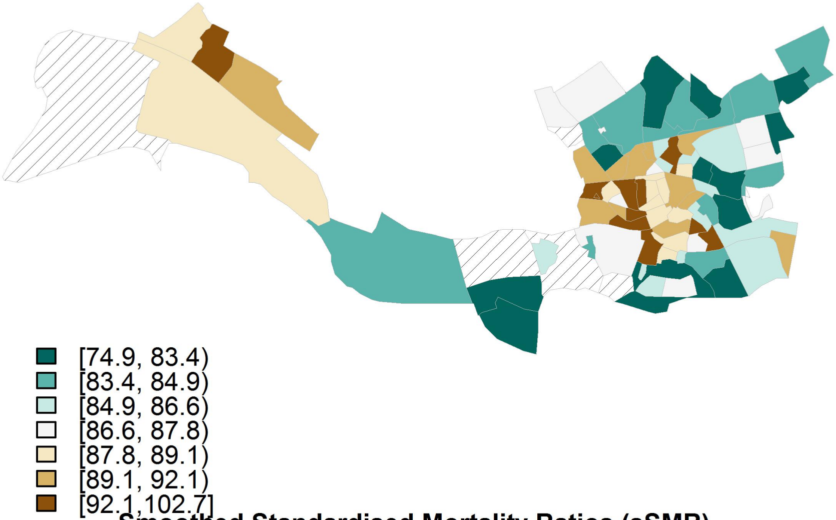

Smoothed Standardised Mortality Ratios (sSMR)
with respect to EU

Rotterdam, Females, 1996 - 2008
MN rectum, anus and anal canal

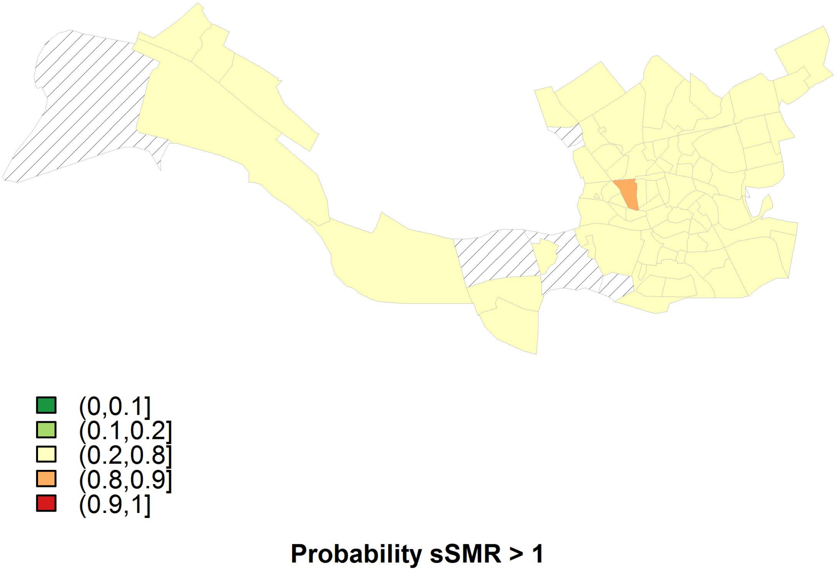

Rotterdam, Females, 1996 - 2008
MN cervix uteri

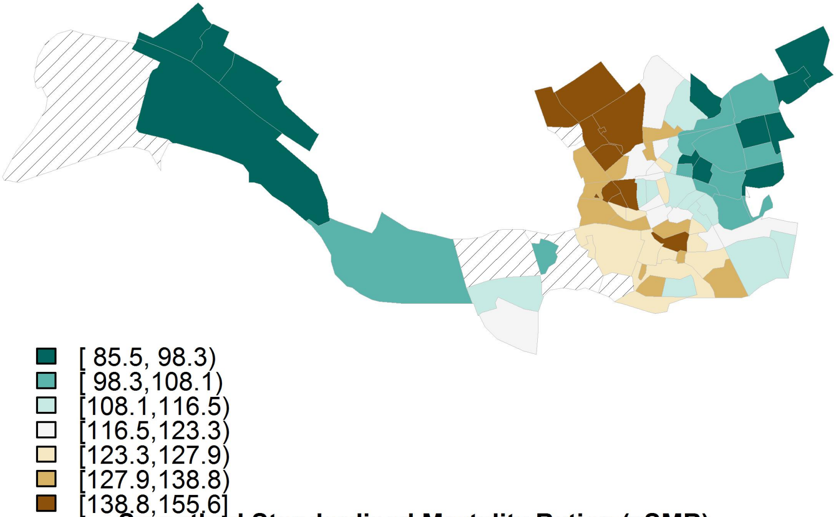

Smoothed Standardised Mortality Ratios (sSMR)
with respect to EU

Rotterdam, Females, 1996 - 2008
MN cervix uteri

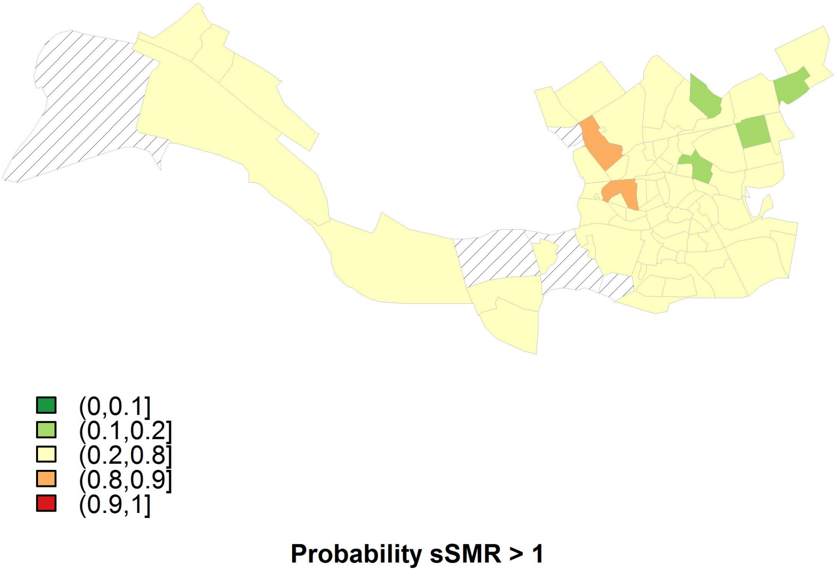

Rotterdam, Females, 1996 - 2008
Hypertension

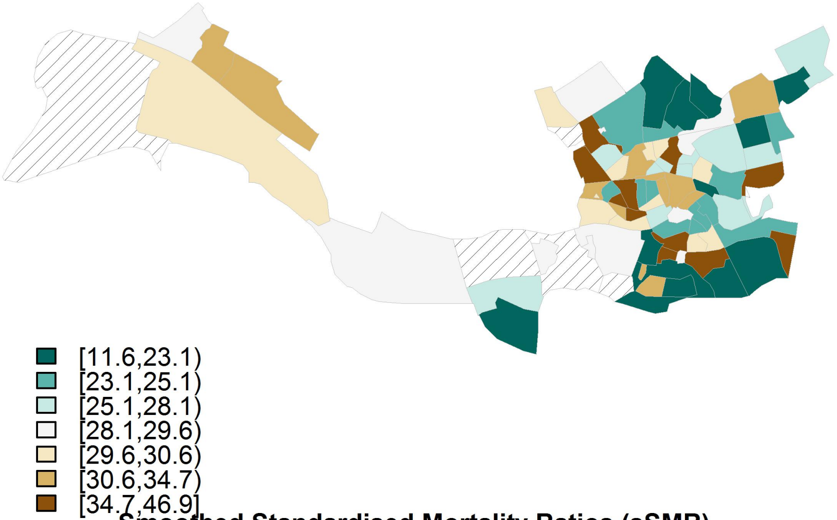

Smoothed Standardised Mortality Ratios (sSMR)
with respect to EU

Rotterdam, Females, 1996 - 2008
Hypertension

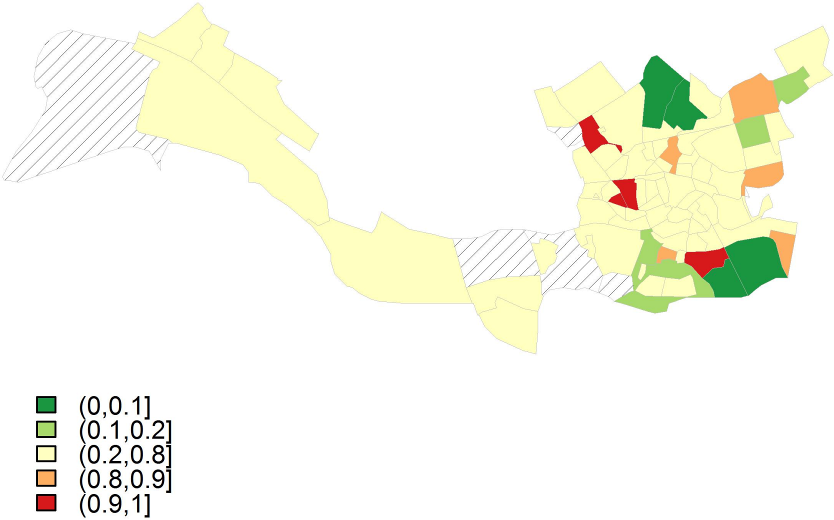

Probability sSMR > 1

Rotterdam, Females, 1996 - 2008
Heart failure

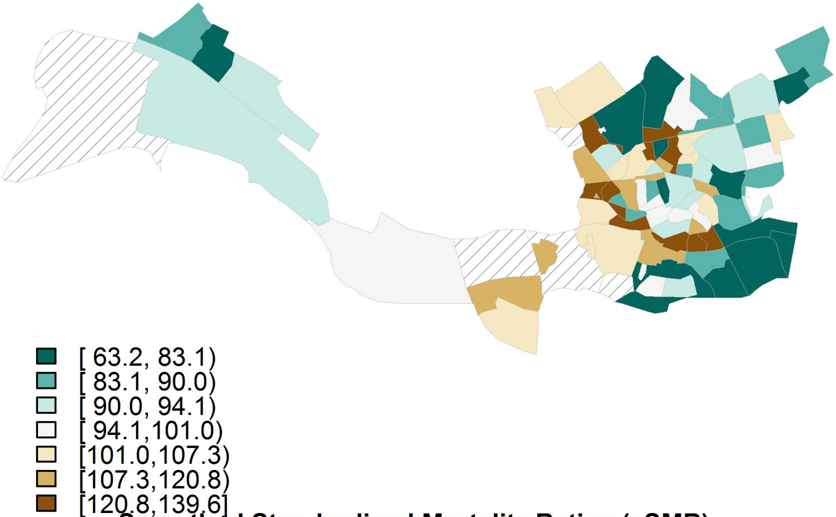

Smoothed Standardised Mortality Ratios (sSMR)
with respect to EU

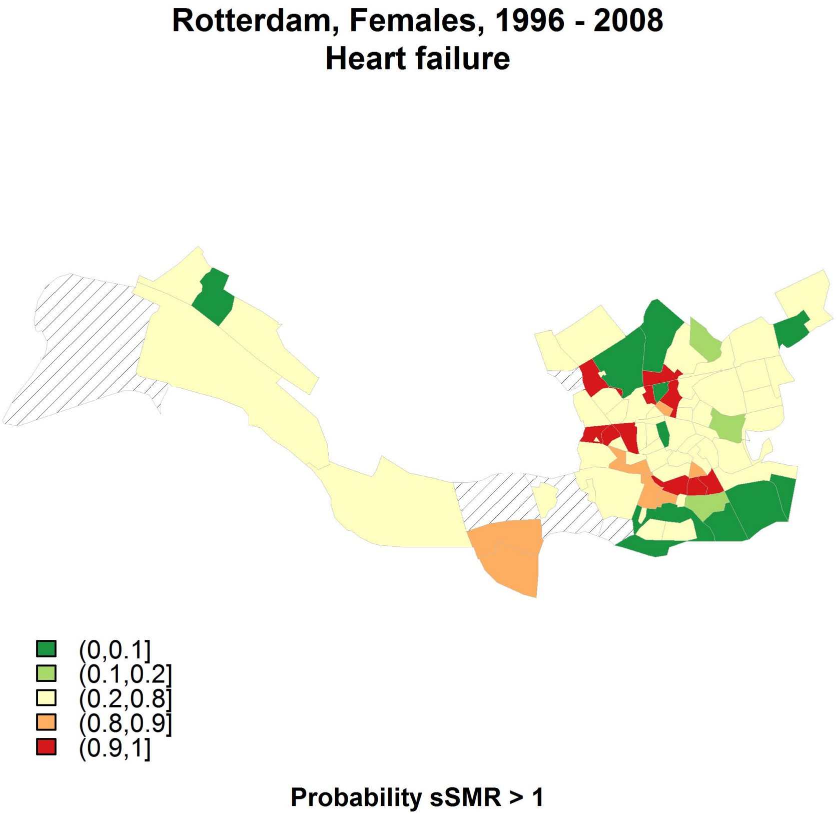
Rotterdam, Females, 1996 - 2008
Heart failure
(0,0.1]
(0.1,0.2]
(0.2,0.8]
(0.8,0.9]
(0.9,1]
Probability sSMR > 1

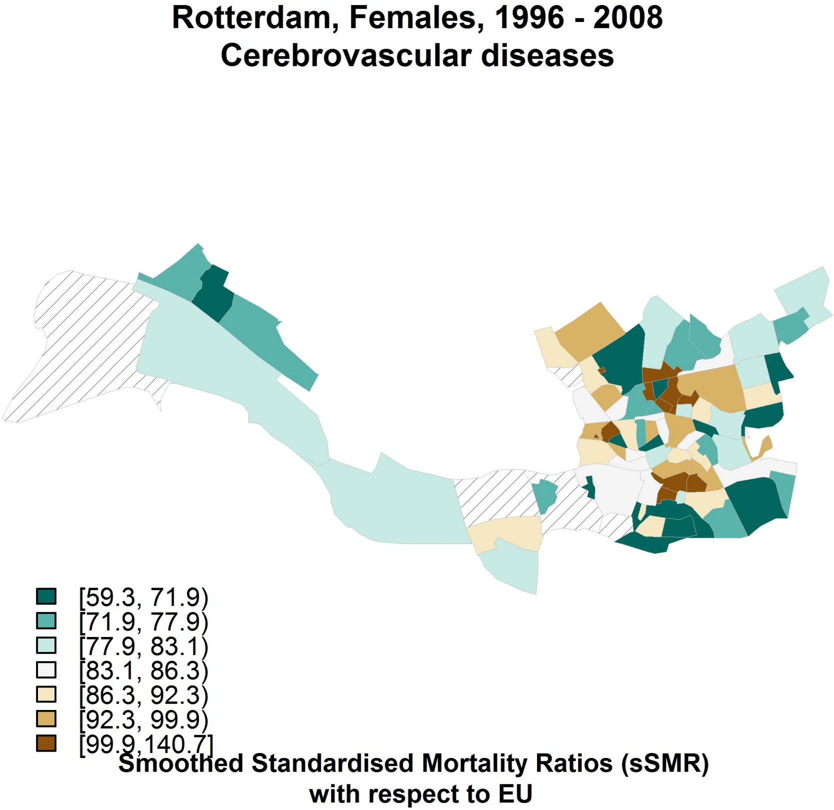
Rotterdam, Females, 1996 - 2008
Cerebrovascular diseases
[59.3, 71.9)
[71.9, 77.9)
[77.9, 83.1)
[83.1, 86.3)
[86.3, 92.3)
[92.3, 99.9)
[99.9,140.7]
Smoothed Standardised Mortality Ratios (sSMR)
with respect to EU

Rotterdam, Females, 1996 - 2008
Cerebrovascular diseases

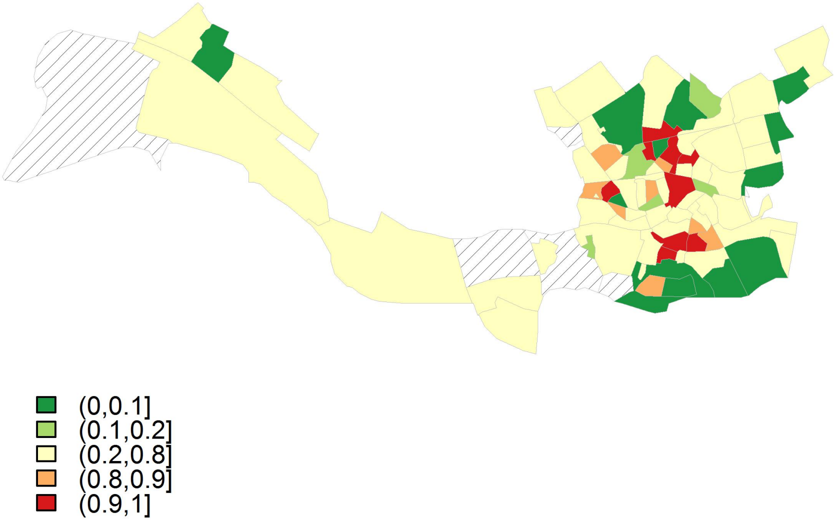

Probability sSMR > 1

Rotterdam, Females, 1996 - 2008
Peptic ulcer

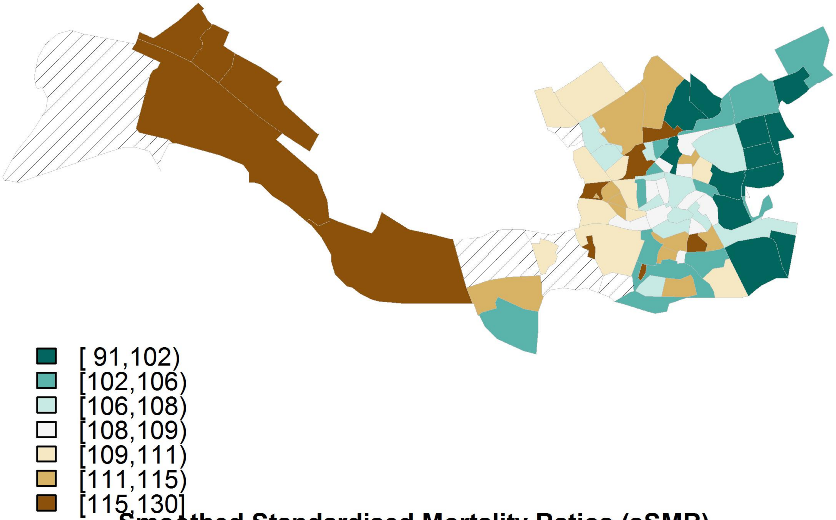

**Smoothed Standardised Mortality Ratios (sSMR)
with respect to EU**

Rotterdam, Females, 1996 - 2008
Peptic ulcer

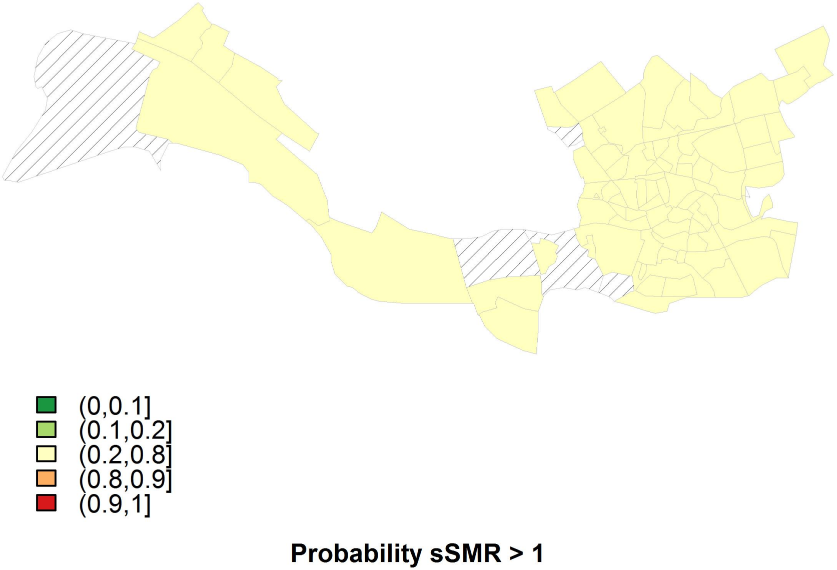

Rotterdam, Females, 1996 - 2008
Renal failure

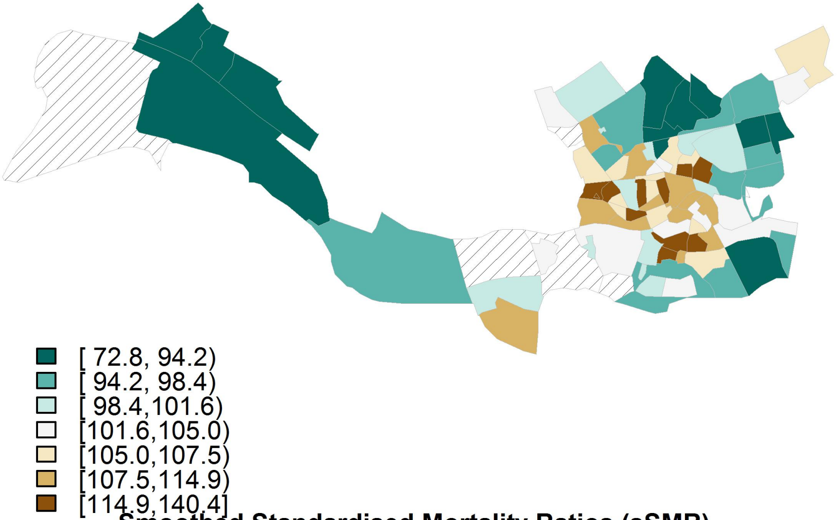

Smoothed Standardised Mortality Ratios (sSMR)
with respect to EU

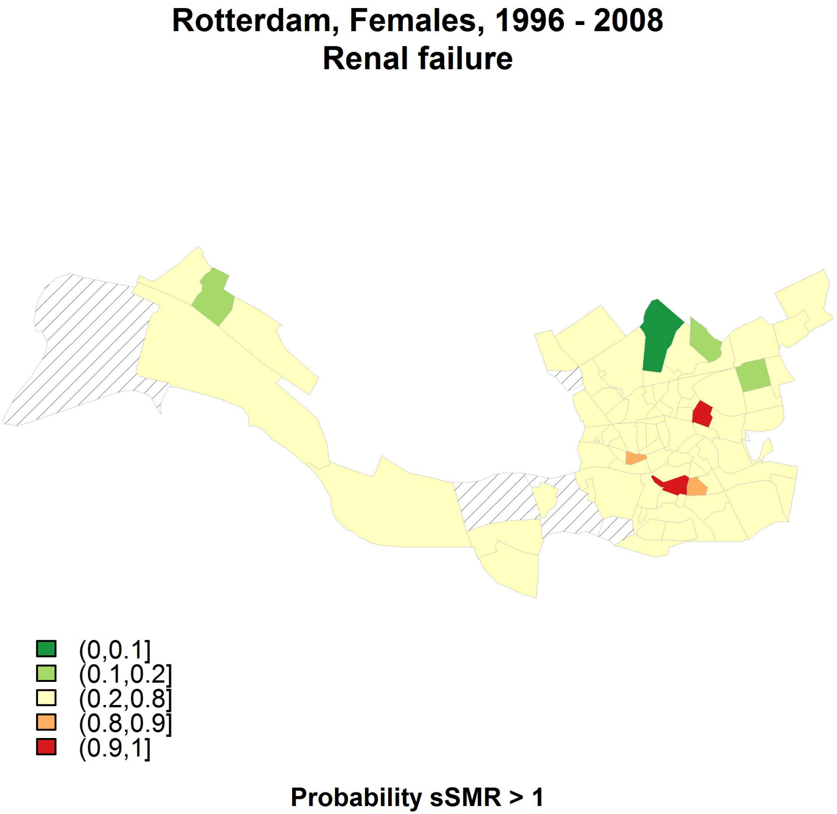
Rotterdam, Females, 1996 - 2008
Renal failure
(0,0.1]
(0.1,0.2]
(0.2,0.8]
(0.8,0.9]
(0.9,1]
Probability sSMR > 1

Rotterdam, Females, 1996 - 2008
Conditions originating in the perinatal period

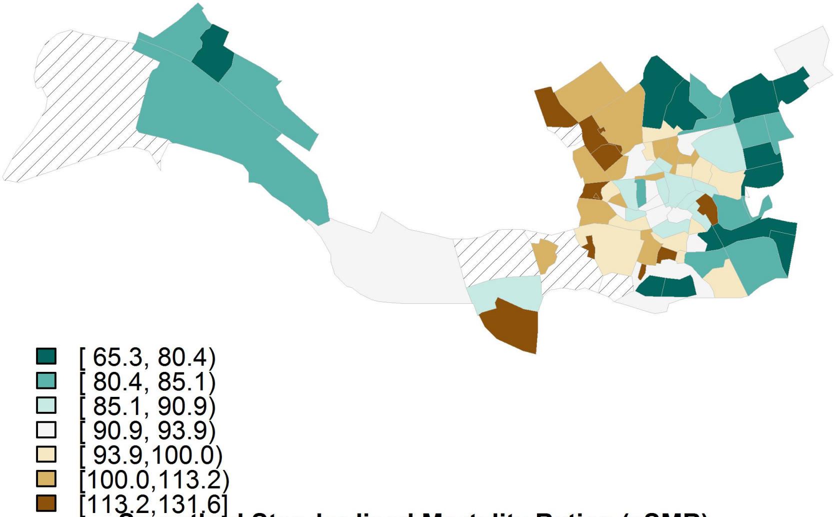

Smoothed Standardised Mortality Ratios (sSMR)
with respect to EU

Rotterdam, Females, 1996 - 2008
Conditions originating in the perinatal period

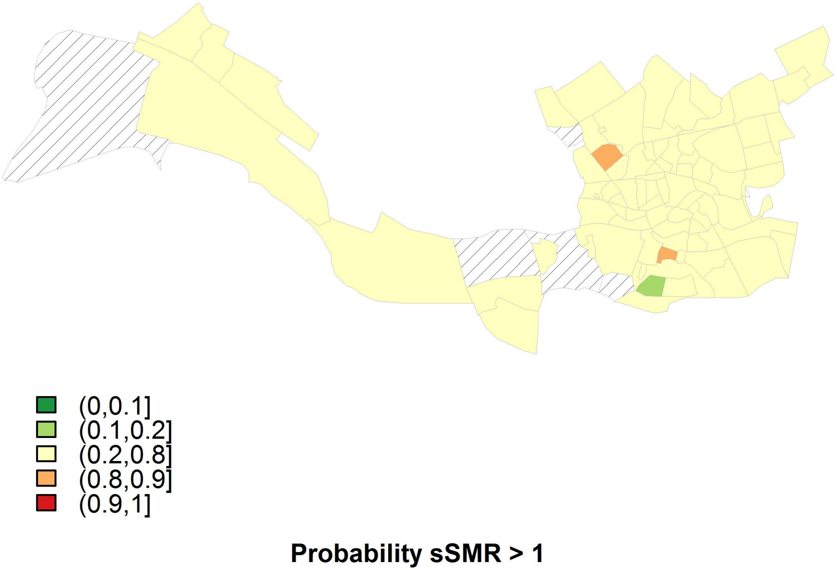

Supplement: Additional file 14 — Cause-specific mortality maps for Rotterdam. [file 1476-072X-13-8-S14.pdf]
