# Supplementary material for: Social differences in avoidable mortality between small areas of 15 European cities: an ecological study
Source: Int J Health Geogr. 2014 Mar 12;13:8. doi: 10.1186/1476-072X-13-8 (PMC4007807; doi:10.1186/1476-072X-13-8)

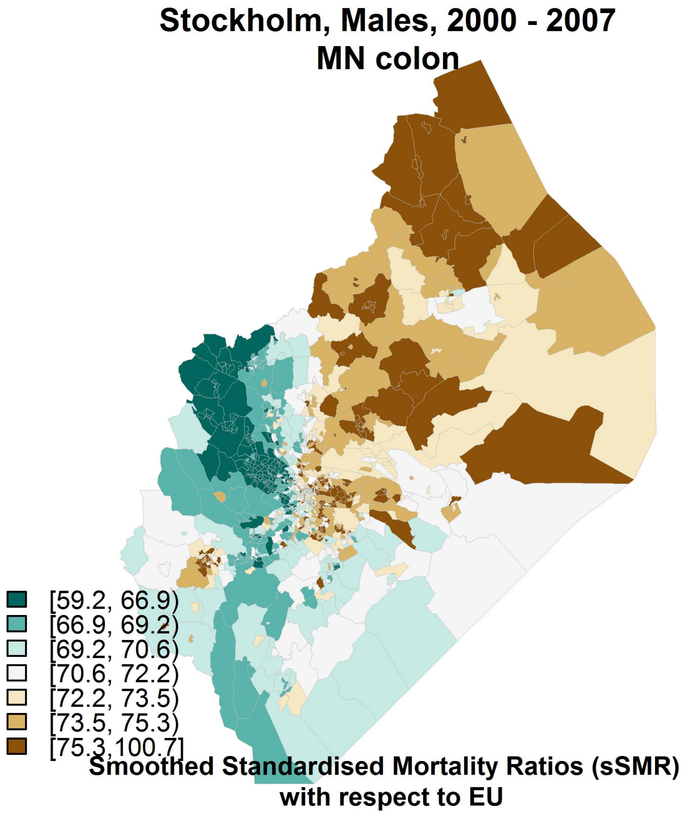
Stockholm, Males, 2000 - 2007
MN colon
[59.2, 66.9)
[66.9, 69.2)
[69.2, 70.6)
[70.6, 72.2)
[72.2, 73.5)
[73.5, 75.3)
[75.3,100.7]
Smoothed Standardised Mortality Ratios (sSMR)
with respect to EU

Stockholm, Males, 2000 - 2007
MN colon

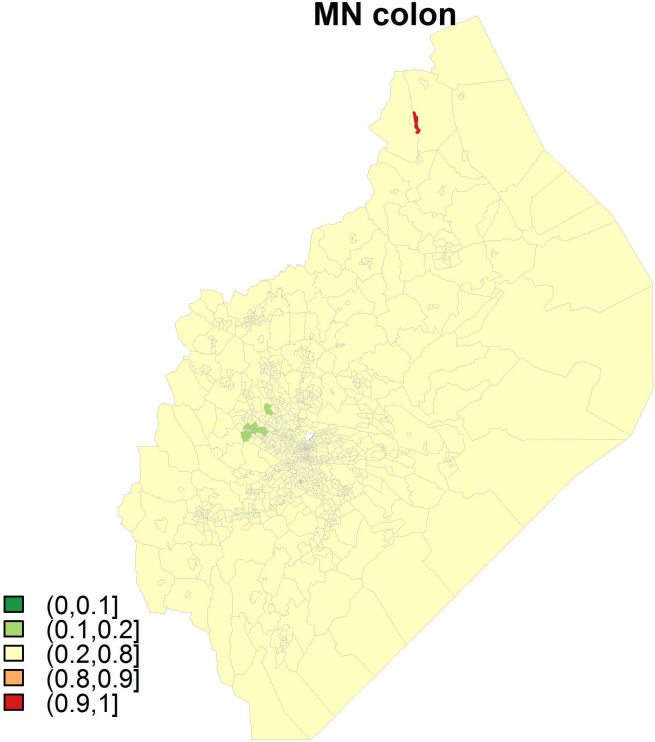

Probability sSMR > 1

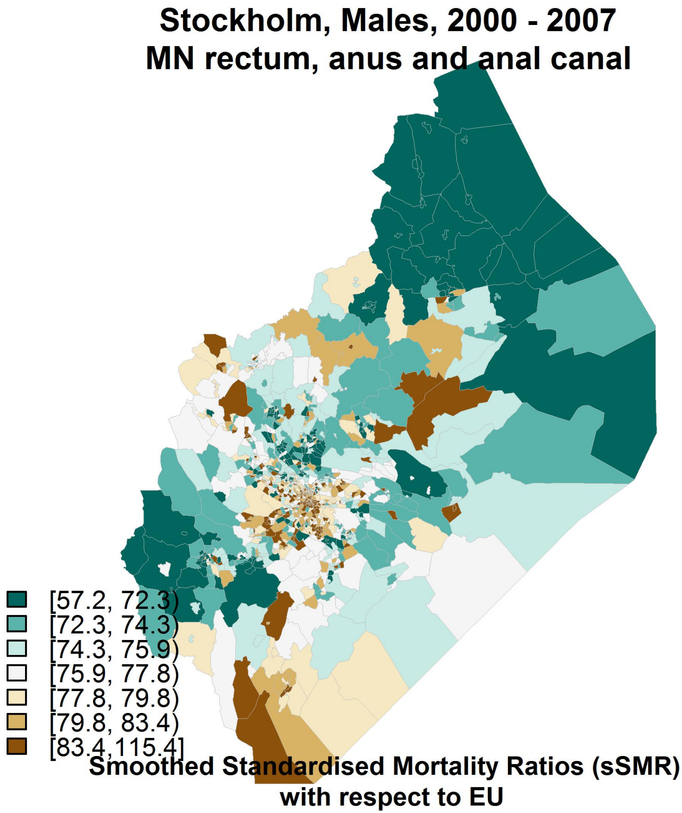
Stockholm, Males, 2000 - 2007
MN rectum, anus and anal canal
[57.2, 72.3)
[72.3, 74.3)
[74.3, 75.9)
[75.9, 77.8)
[77.8, 79.8)
[79.8, 83.4)
[83.4,115.4]
Smoothed Standardised Mortality Ratios (sSMR)
with respect to EU

Stockholm, Males, 2000 - 2007
MN rectum, anus and anal canal

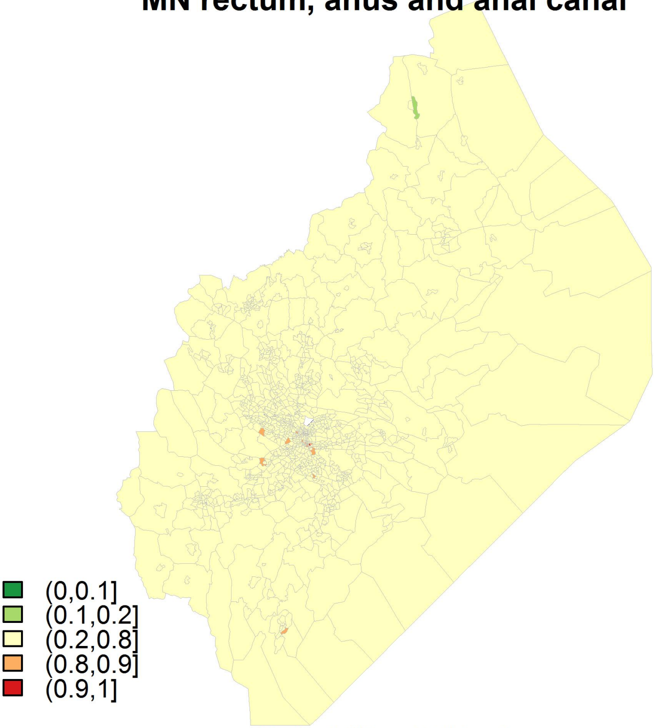

Probability sSMR > 1

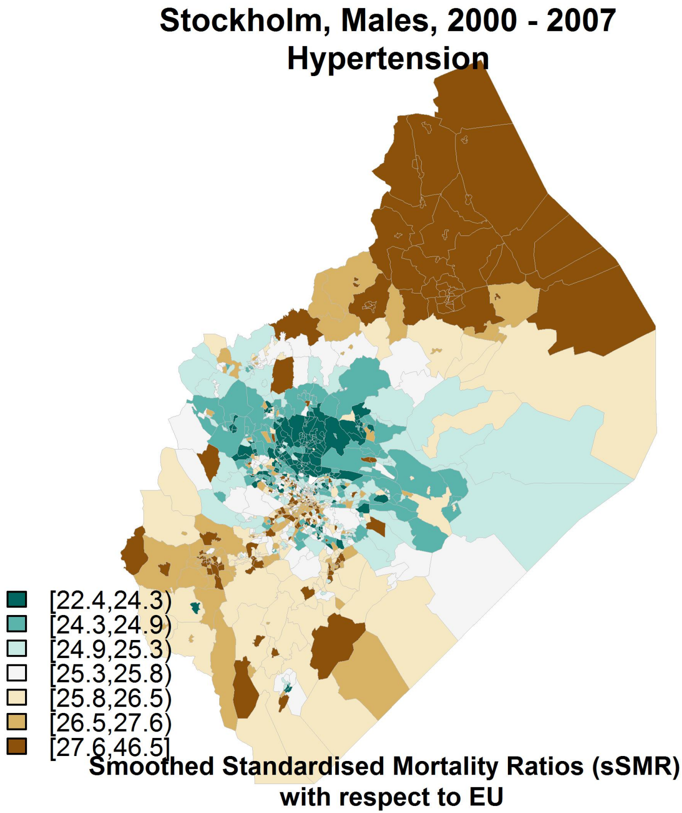
Stockholm, Males, 2000 - 2007
Hypertension
[22.4,24.3)
[24.3,24.9)
[24.9,25.3)
[25.3,25.8)
[25.8,26.5)
[26.5,27.6)
[27.6,46.5]
Smoothed Standardised Mortality Ratios (sSMR)
with respect to EU

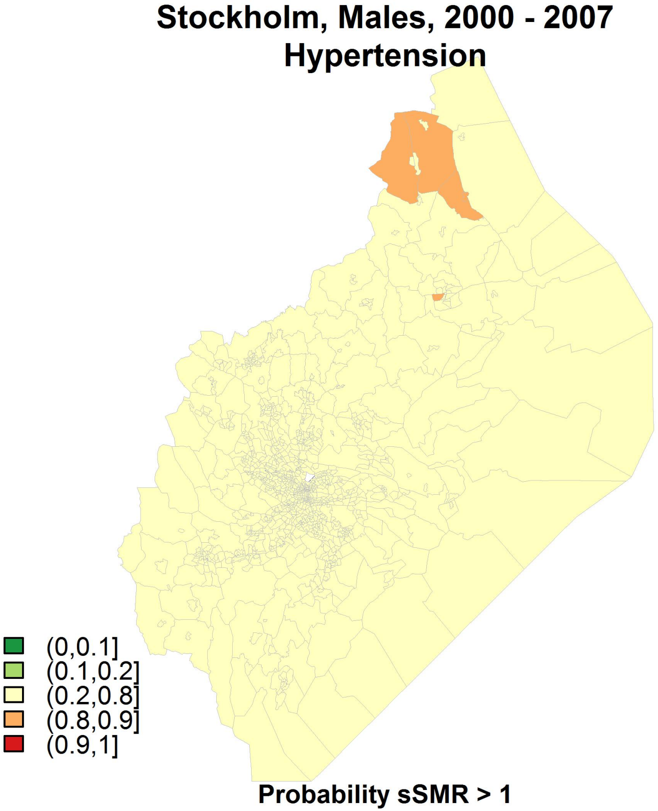
Stockholm, Males, 2000 - 2007
Hypertension
(0,0.1]
(0.1,0.2]
(0.2,0.8]
(0.8,0.9]
(0.9,1]
Probability sSMR > 1

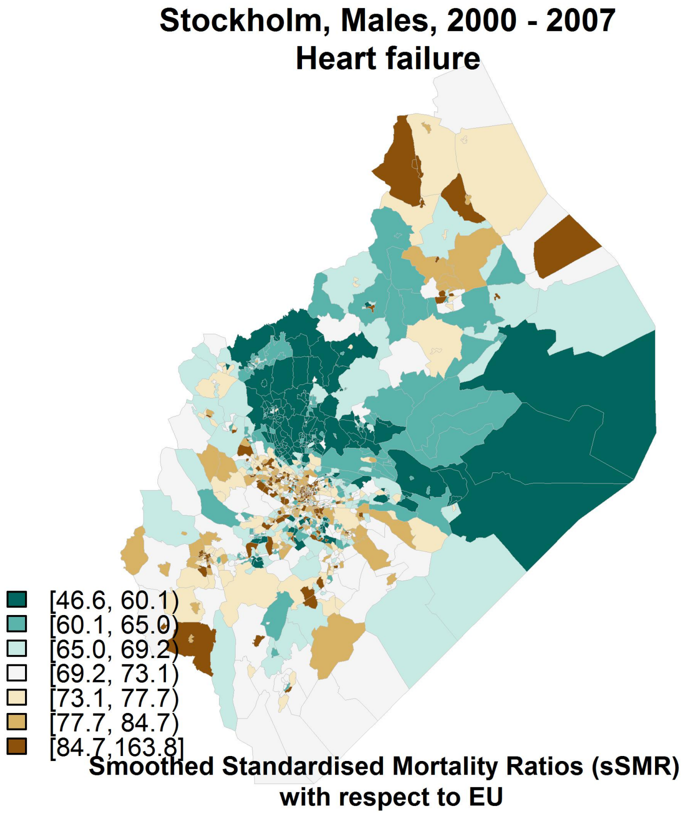
Stockholm, Males, 2000 - 2007
Heart failure
[46.6, 60.1)
[60.1, 65.0)
[65.0, 69.2)
[69.2, 73.1)
[73.1, 77.7)
[77.7, 84.7)
[84.7,163.8]
Smoothed Standardised Mortality Ratios (sSMR)
with respect to EU

Stockholm, Males, 2000 - 2007
Heart failure

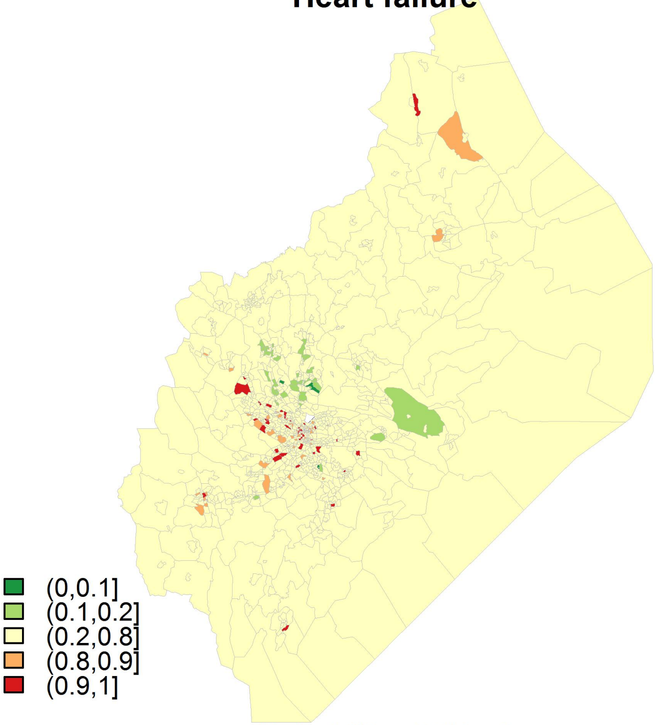

Probability sSMR > 1

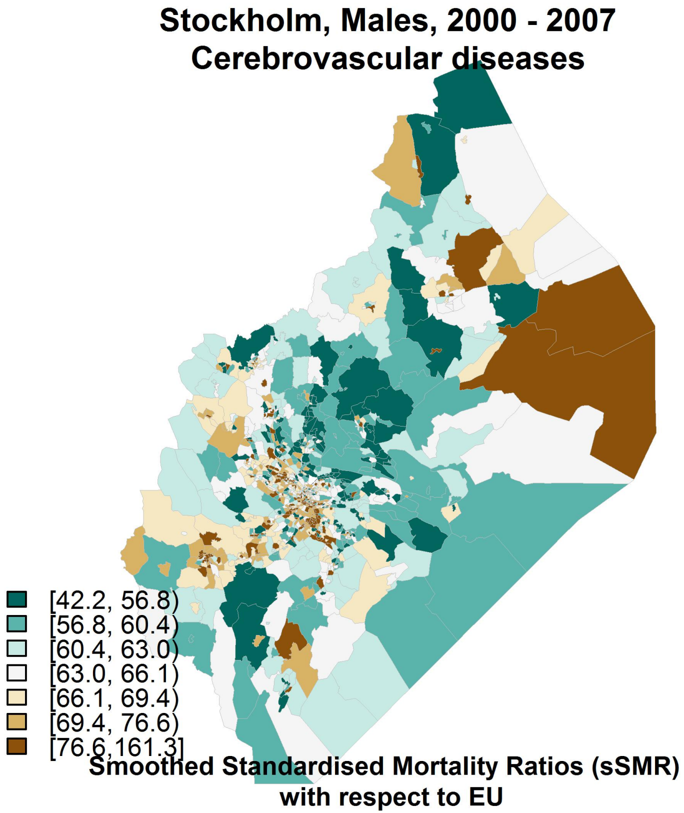
Stockholm, Males, 2000 - 2007
Cerebrovascular diseases
[42.2, 56.8)
[56.8, 60.4)
[60.4, 63.0)
[63.0, 66.1)
[66.1, 69.4)
[69.4, 76.6)
[76.6,161.3]
Smoothed Standardised Mortality Ratios (sSMR)
with respect to EU

Stockholm, Males, 2000 - 2007
Cerebrovascular diseases

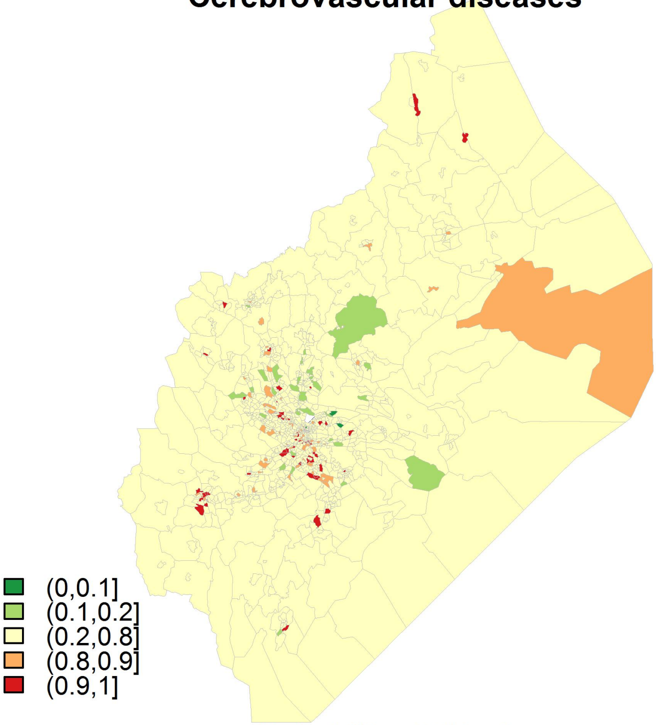

Probability sSMR > 1

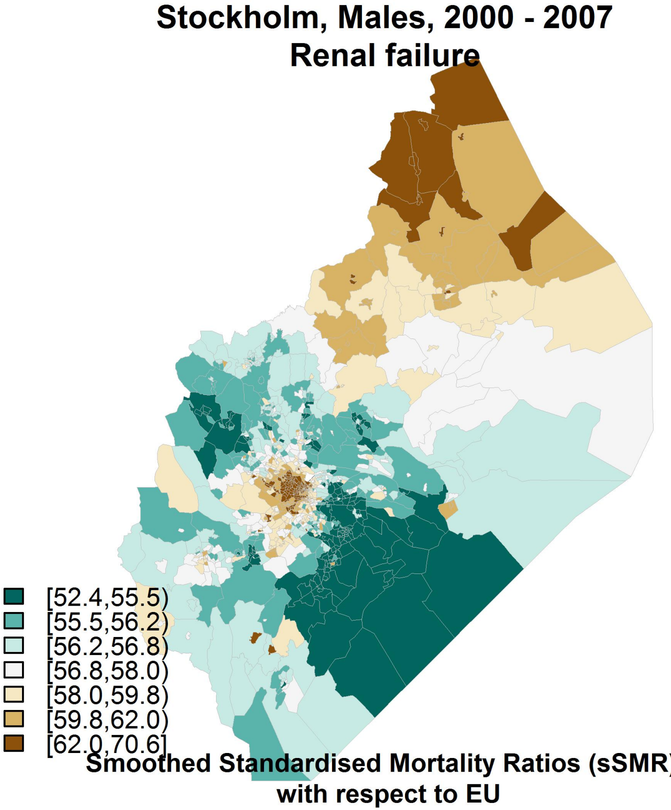
Stockholm, Males, 2000 - 2007
Renal failure
[52.4,55.5)
[55.5,56.2)
[56.2,56.8)
[56.8,58.0)
[58.0,59.8)
[59.8,62.0)
[62.0,70.6]
Smoothed Standardised Mortality Ratios (sSMR)
with respect to EU

Stockholm, Males, 2000 - 2007
Renal failure

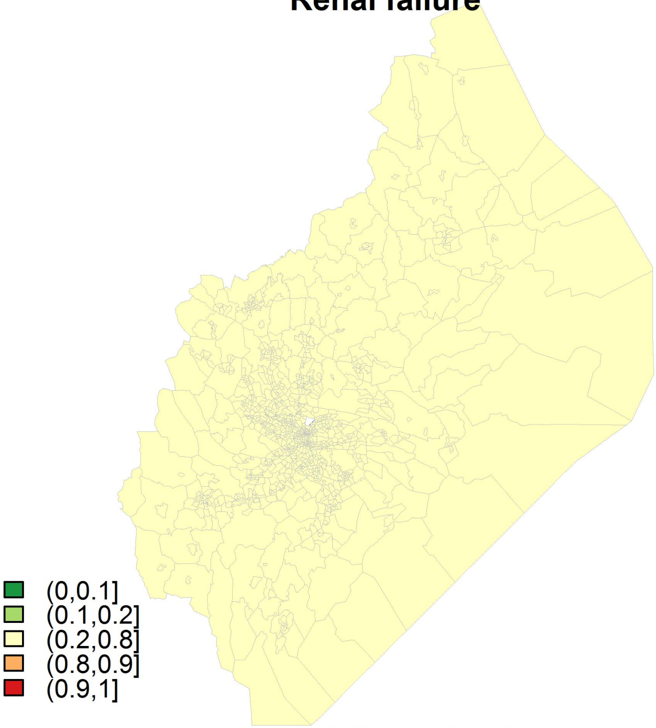

Probability sSMR > 1

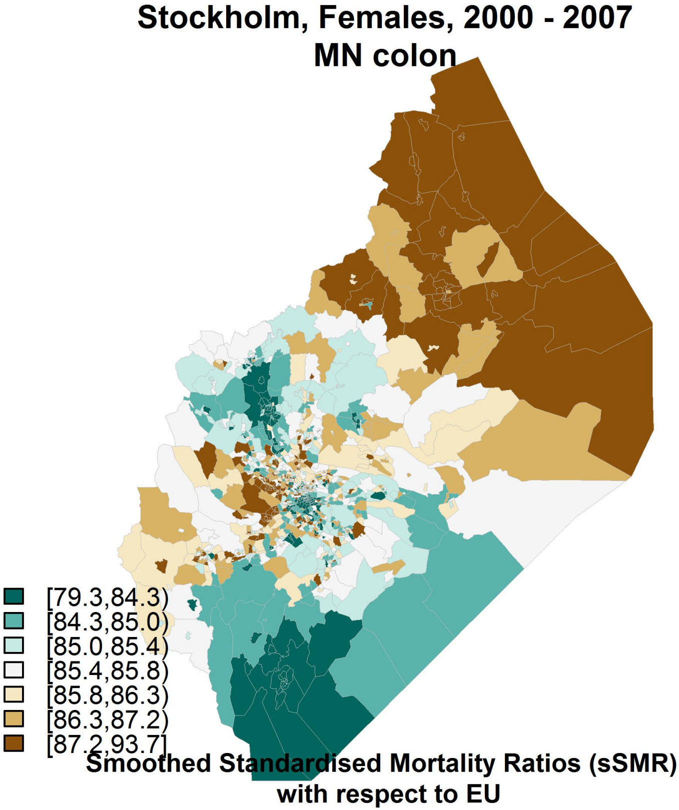
Stockholm, Females, 2000 - 2007
MN colon
[79.3,84.3)
[84.3,85.0)
[85.0,85.4)
[85.4,85.8)
[85.8,86.3)
[86.3,87.2)
[87.2,93.7]
Smoothed Standardised Mortality Ratios (sSMR)
with respect to EU

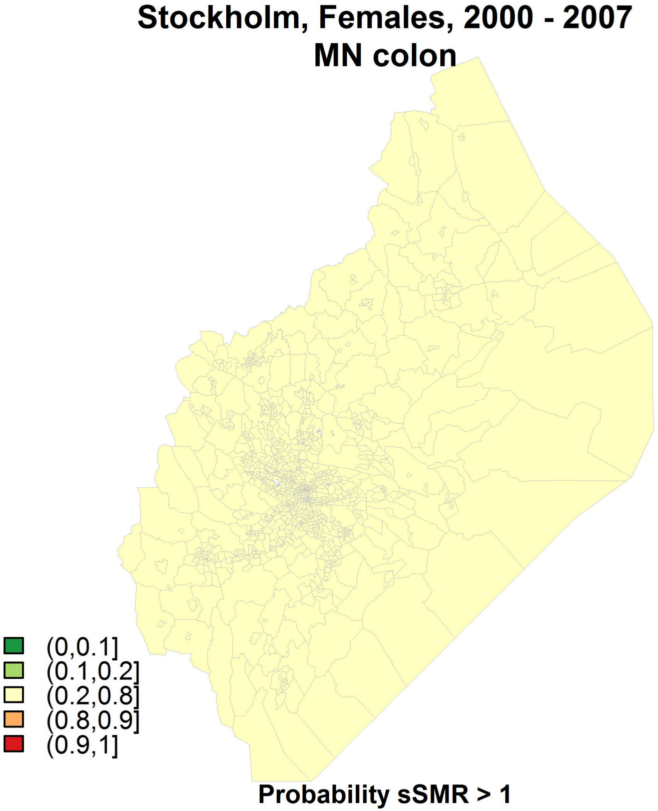
Stockholm, Females, 2000 - 2007
MN colon
(0,0.1]
(0.1,0.2]
(0.2,0.8]
(0.8,0.9]
(0.9,1]
Probability sSMR > 1

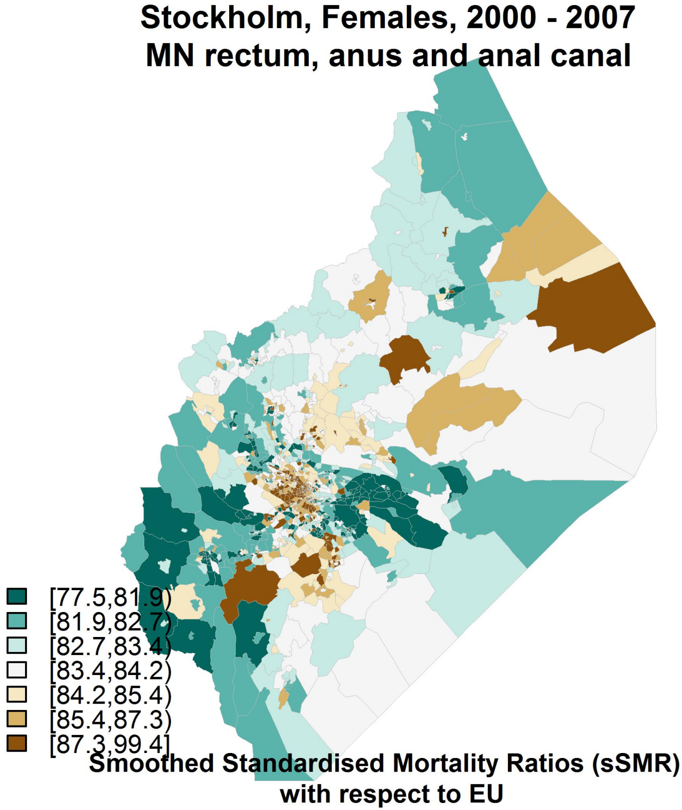

Stockholm, Females, 2000 - 2007
MN rectum, anus and anal canal
[77.5,81.9)
[81.9,82.7)
[82.7,83.4)
[83.4,84.2)
[84.2,85.4)
[85.4,87.3)
[87.3,99.4]
Smoothed Standardised Mortality Ratios (sSMR)
with respect to EU

Stockholm, Females, 2000 - 2007
MN rectum, anus and anal canal

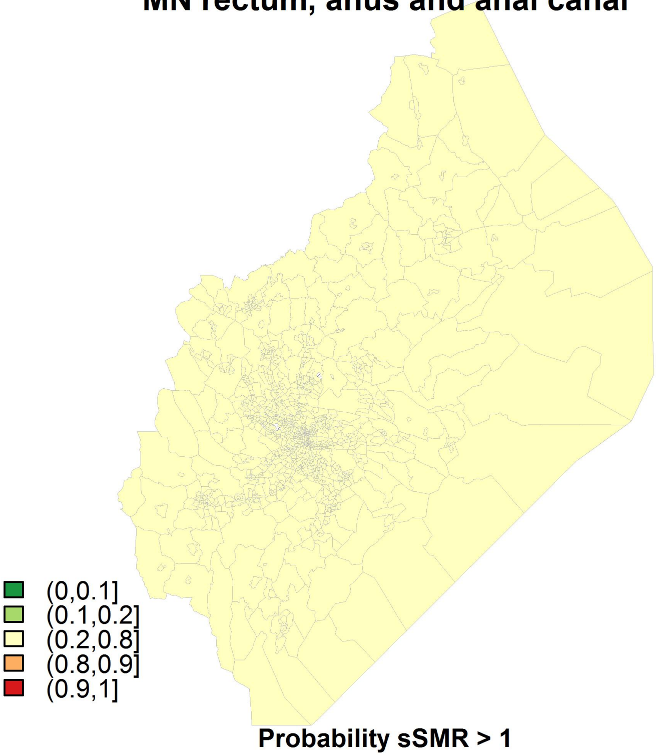

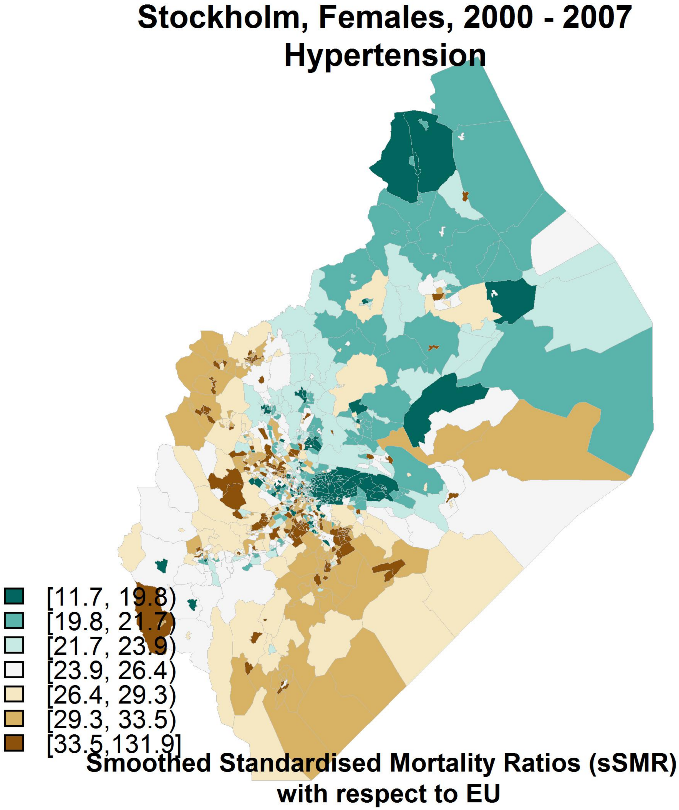
Stockholm, Females, 2000 - 2007
Hypertension
[11.7, 19.8)
[19.8, 21.7)
[21.7, 23.9)
[23.9, 26.4)
[26.4, 29.3)
[29.3, 33.5)
[33.5,131.9]
Smoothed Standardised Mortality Ratios (sSMR)
with respect to EU

Stockholm, Females, 2000 - 2007
Hypertension

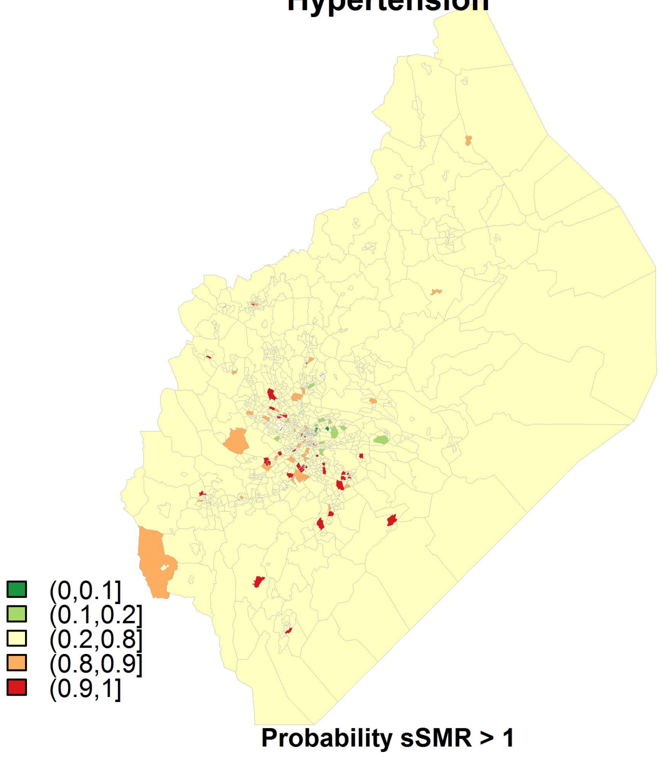

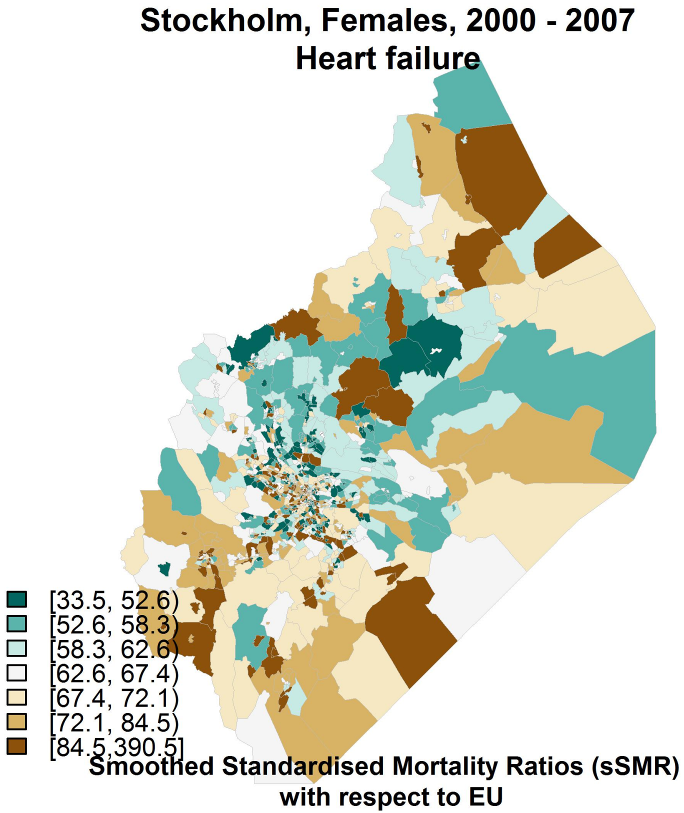
Stockholm, Females, 2000 - 2007
Heart failure
[33.5, 52.6)
[52.6, 58.3)
[58.3, 62.6)
[62.6, 67.4)
[67.4, 72.1)
[72.1, 84.5)
[84.5,390.5]
Smoothed Standardised Mortality Ratios (sSMR)
with respect to EU

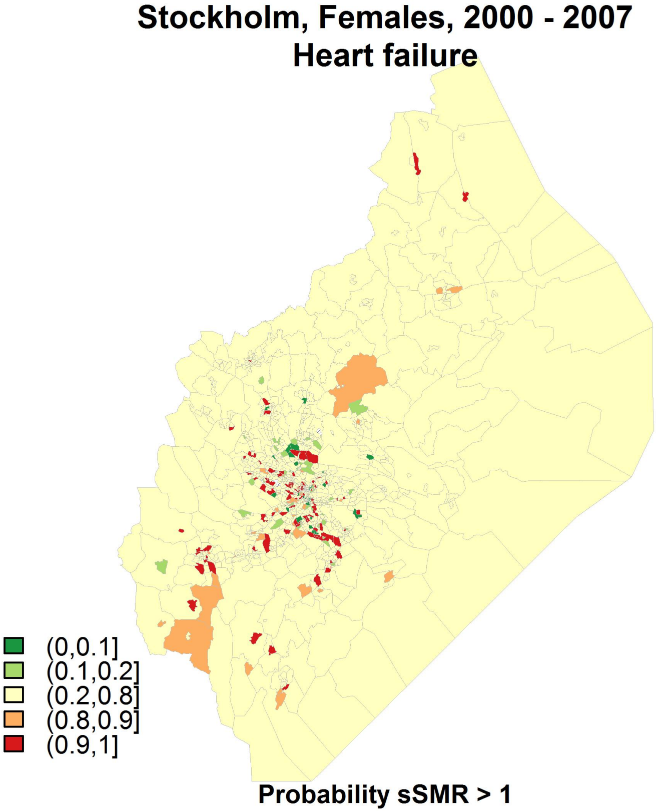
Stockholm, Females, 2000 - 2007
Heart failure
(0,0.1]
(0.1,0.2]
(0.2,0.8]
(0.8,0.9]
(0.9,1]
Probability sSMR > 1

Stockholm, Females, 2000 - 2007
Cerebrovascular diseases

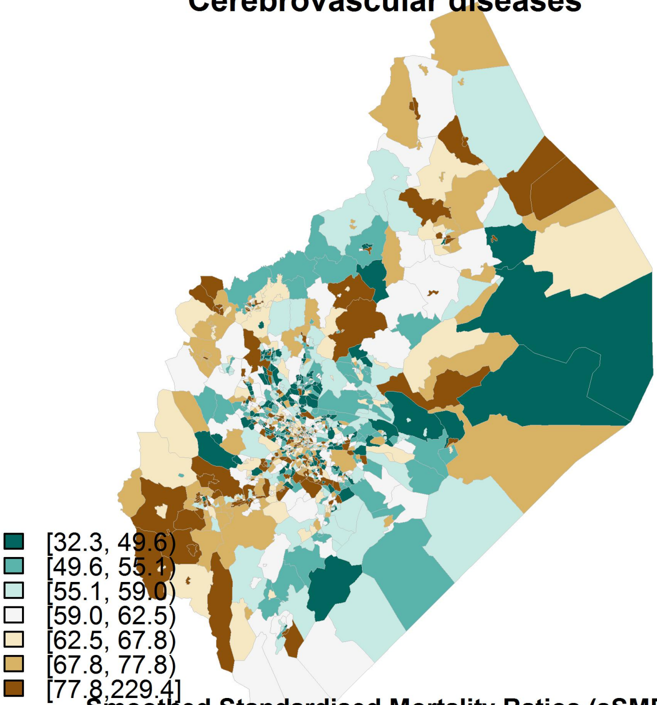

Smoothed Standardised Mortality Ratios (sSMR)
with respect to EU

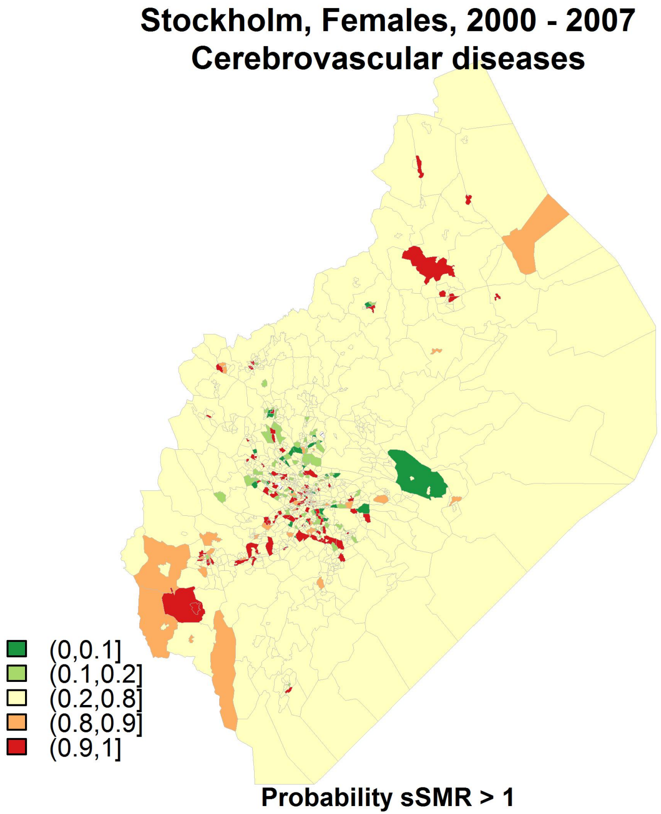
Stockholm, Females, 2000 - 2007
Cerebrovascular diseases
(0,0.1]
(0.1,0.2]
(0.2,0.8]
(0.8,0.9]
(0.9,1]
Probability sSMR > 1

Stockholm, Females, 2000 - 2007
Renal failure

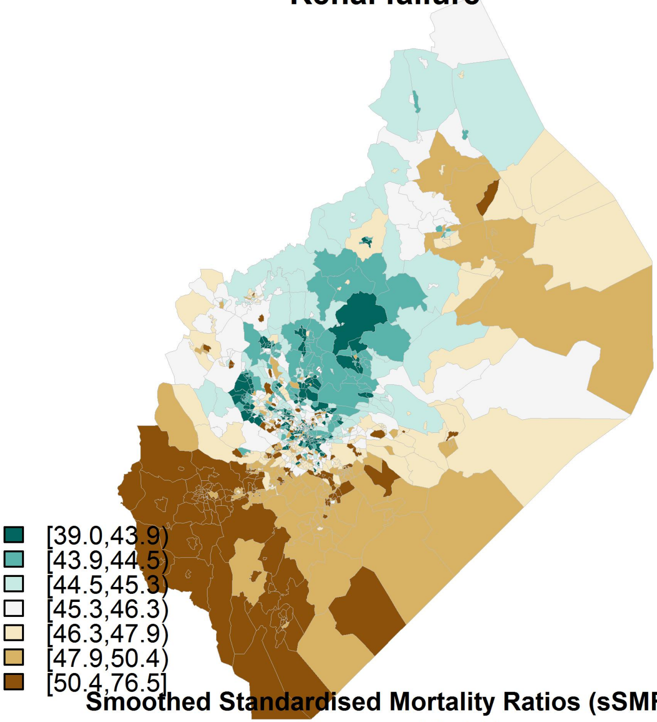

Smoothed Standardised Mortality Ratios (sSMR)
with respect to EU

Stockholm, Females, 2000 - 2007
Renal failure

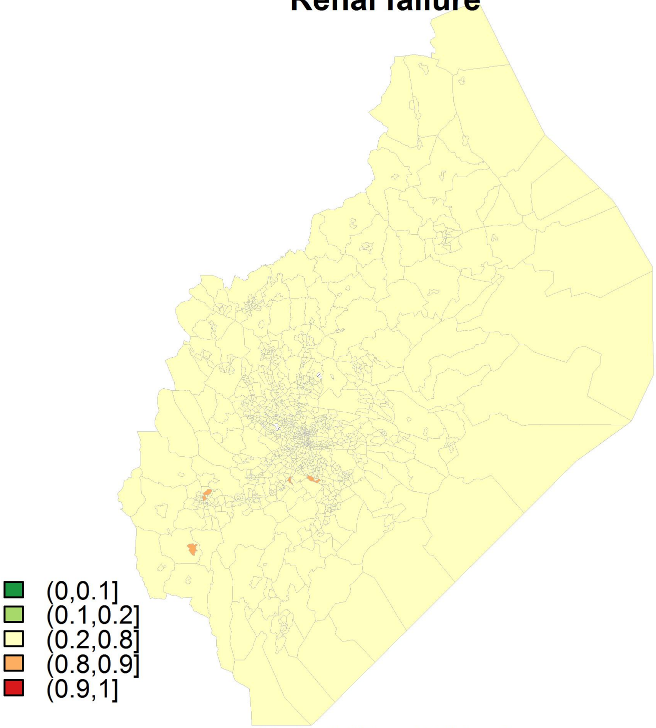

Supplement: Additional file 15 — Cause-specific mortality maps for Stockholm. [file 1476-072X-13-8-S15.pdf]
