# Supplementary material for: Social differences in avoidable mortality between small areas of 15 European cities: an ecological study
Source: Int J Health Geogr. 2014 Mar 12;13:8. doi: 10.1186/1476-072X-13-8 (PMC4007807; doi:10.1186/1476-072X-13-8)
Supplement: Additional file 16 — Cause-specific mortality maps for Turin. [file 1476-072X-13-8-S16.pdf]

Turin, Males, 1995 - 2008
AIDS (HIV disease)

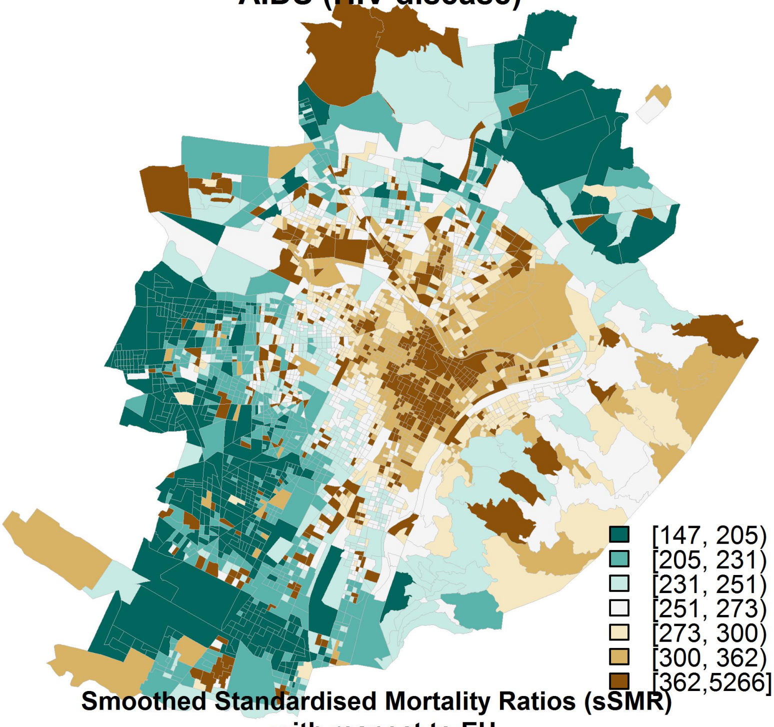

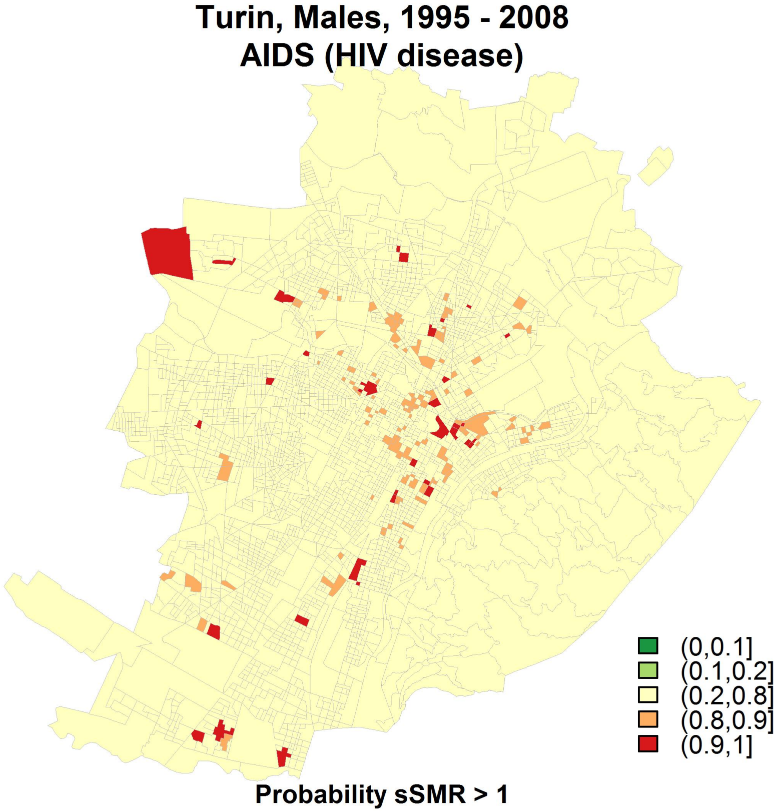
Turin, Males, 1995 - 2008
AIDS (HIV disease)
(0,0.1]
(0.1,0.2]
(0.2,0.8]
(0.8,0.9]
(0.9,1]
Probability sSMR > 1

Turin, Males, 1995 - 2008
MN colon

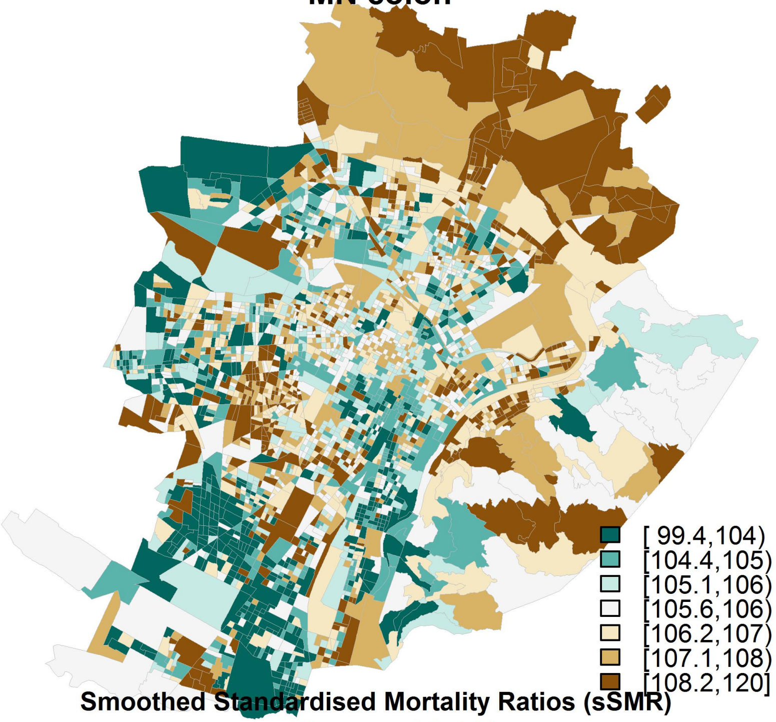

Smoothed Standardised Mortality Ratios (sSMR)
with respect to EU

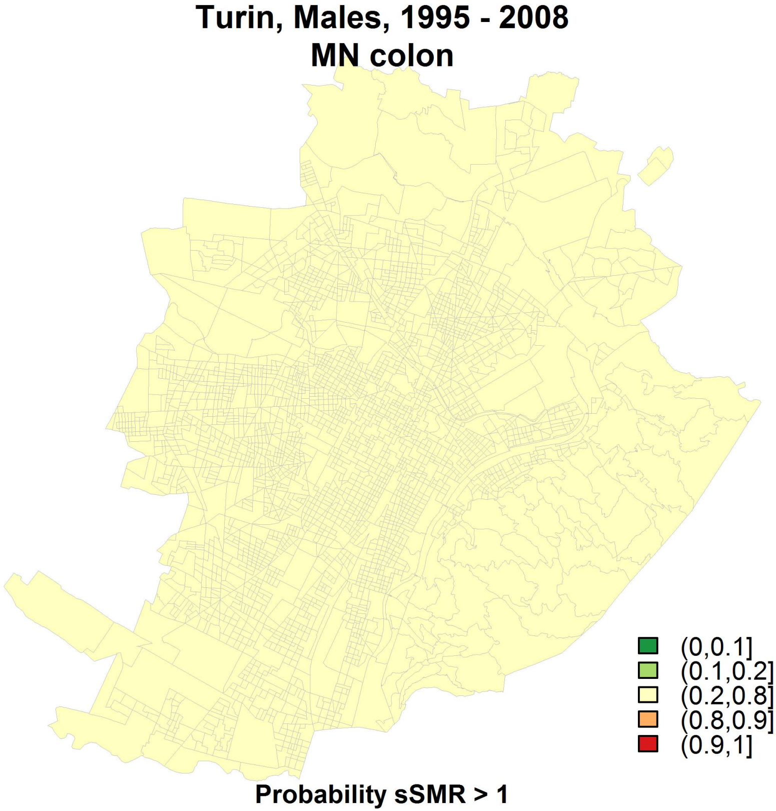
Turin, Males, 1995 - 2008
MN colon
(0,0.1]
(0.1,0.2]
(0.2,0.8]
(0.8,0.9]
(0.9,1]
Probability sSMR > 1

Turin, Males, 1995 - 2008
MN rectum, anus and anal canal

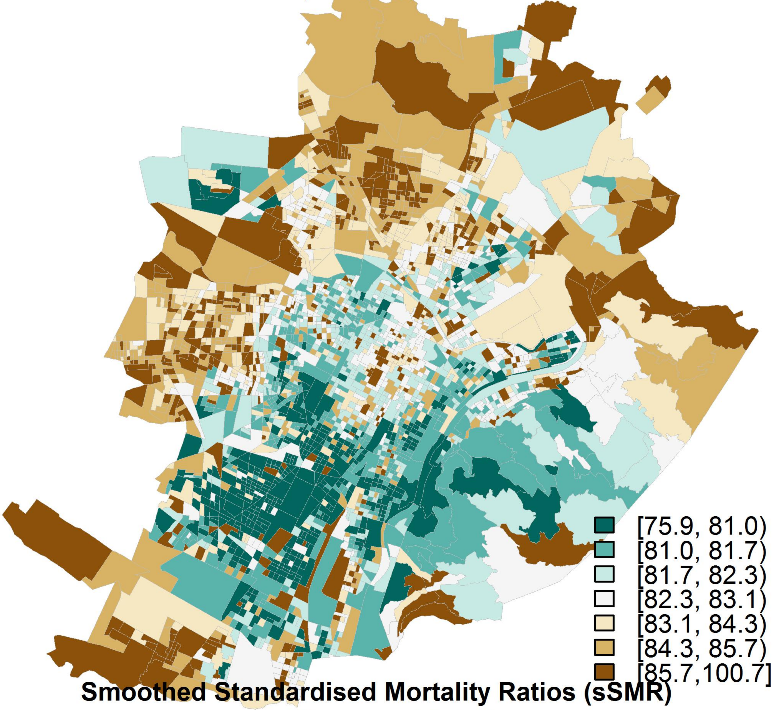

Smoothed Standardised Mortality Ratios (sSMR)
with respect to EU

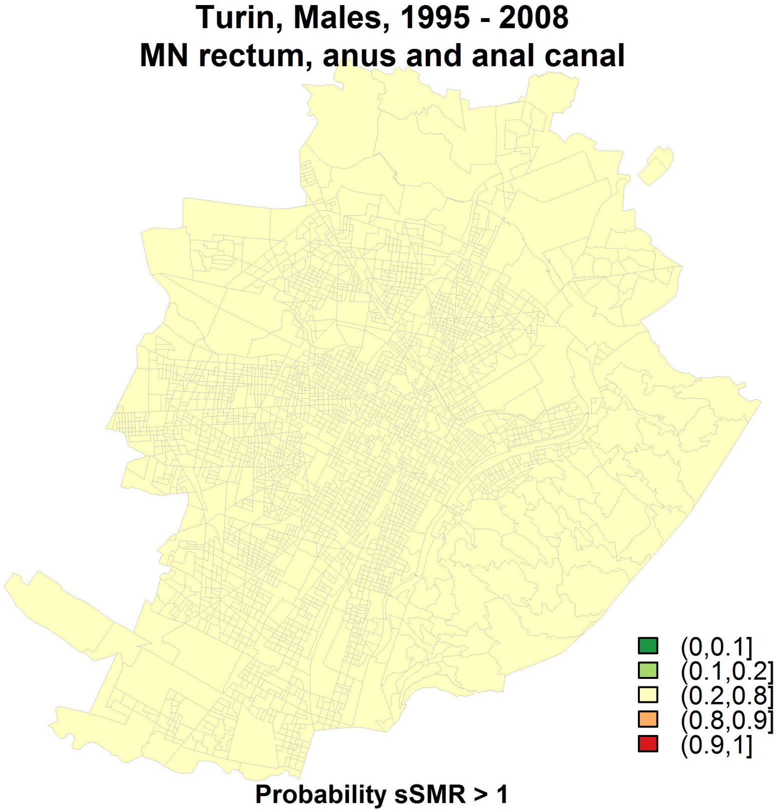

Turin, Males, 1995 - 2008
MN rectum, anus and anal canal
(0,0.1]
(0.1,0.2]
(0.2,0.8]
(0.8,0.9]
(0.9,1]
Probability sSMR > 1

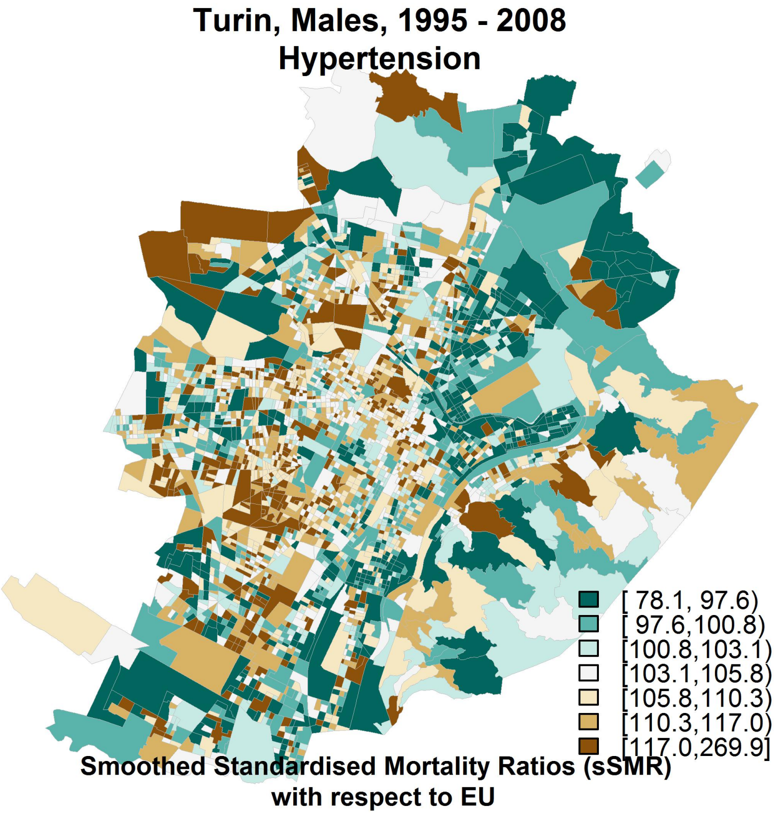
Turin, Males, 1995 - 2008
Hypertension
[78.1, 97.6)
[97.6,100.8)
[100.8,103.1)
[103.1,105.8)
[105.8,110.3)
[110.3,117.0)
[117.0,269.9]
Smoothed Standardised Mortality Ratios (sSMR)
with respect to EU

Turin, Males, 1995 - 2008
Hypertension

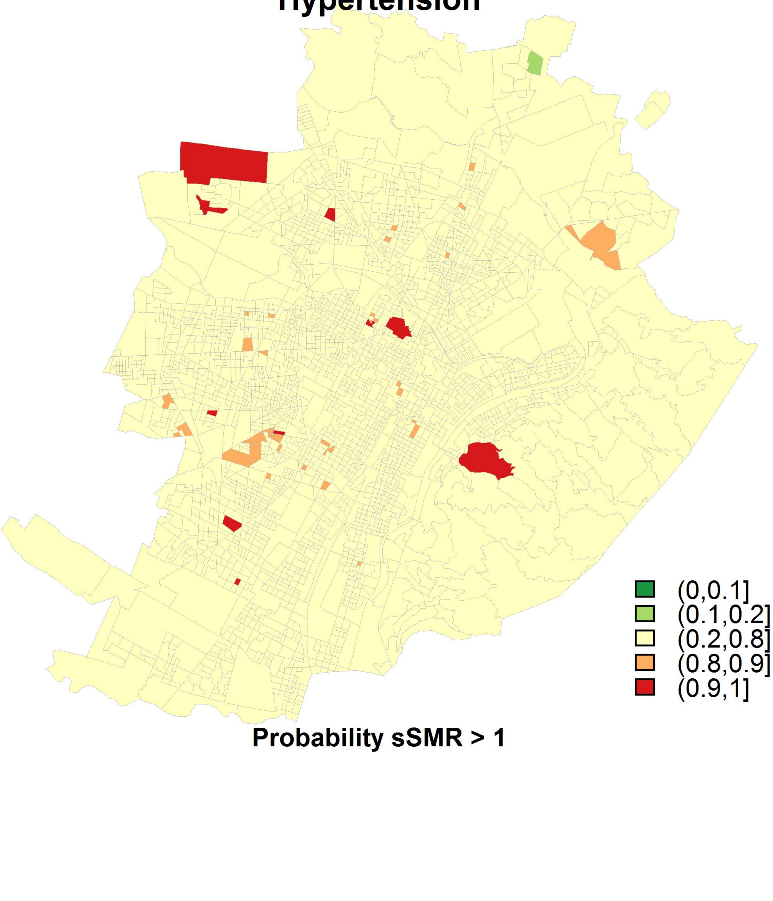

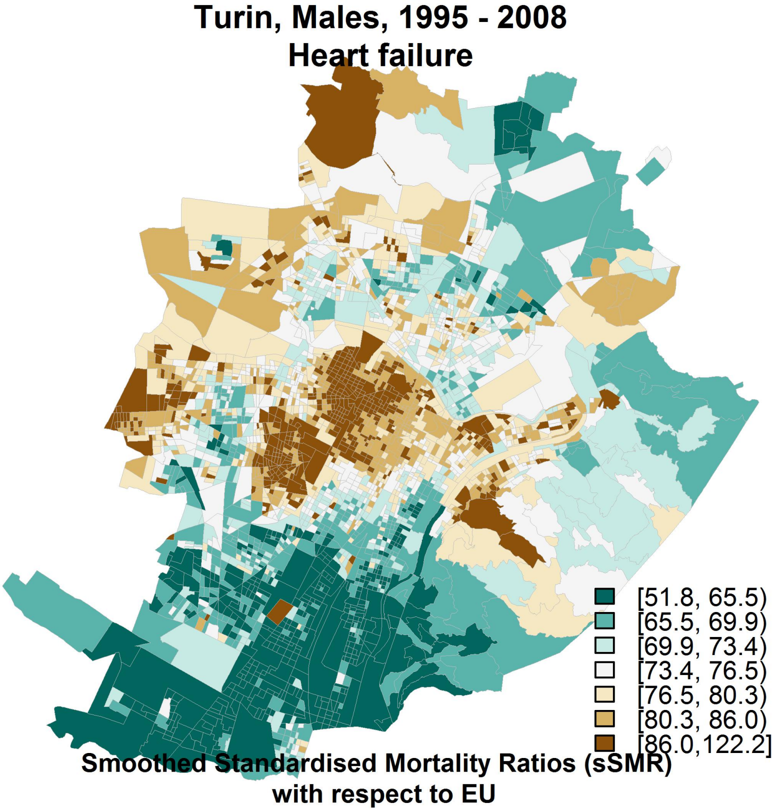
Turin, Males, 1995 - 2008
Heart failure
[51.8, 65.5)
[65.5, 69.9)
[69.9, 73.4)
[73.4, 76.5)
[76.5, 80.3)
[80.3, 86.0)
[86.0,122.2]
Smoothed Standardised Mortality Ratios (sSMR)
with respect to EU

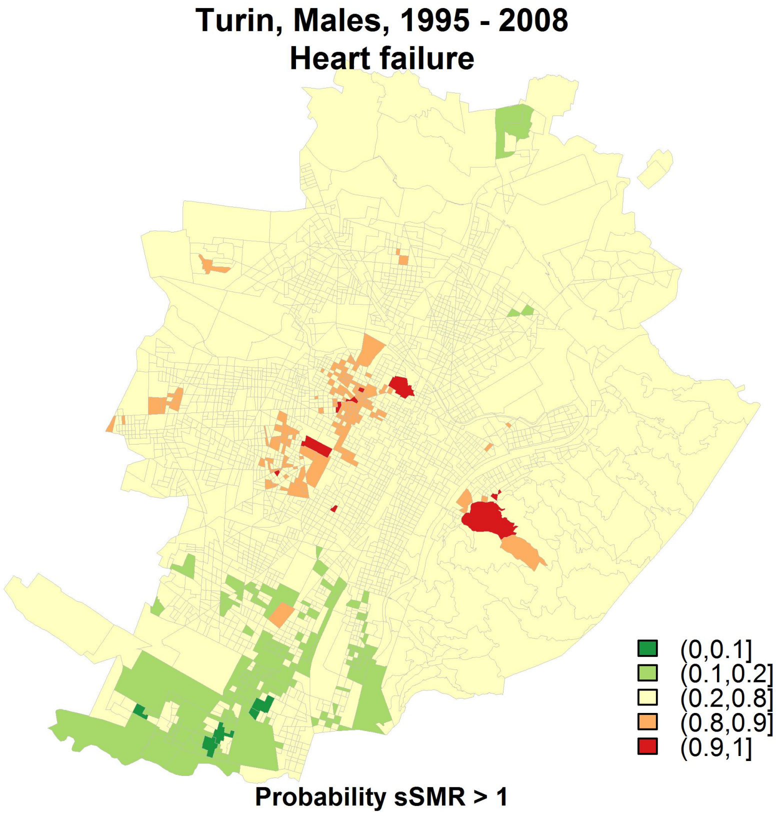
Turin, Males, 1995 - 2008
Heart failure
(0,0.1]
(0.1,0.2]
(0.2,0.8]
(0.8,0.9]
(0.9,1]
Probability sSMR > 1

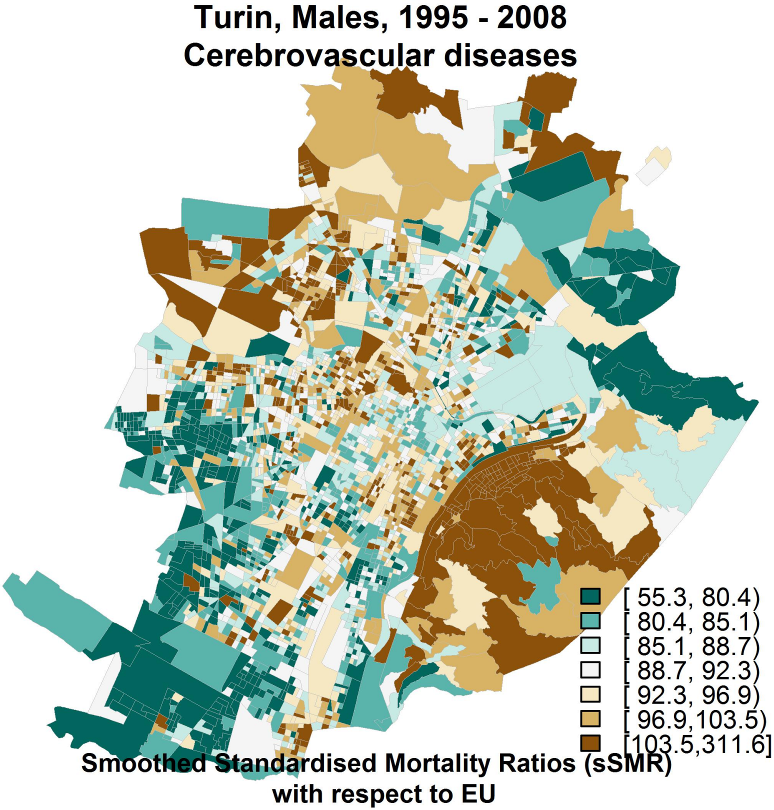
Turin, Males, 1995 - 2008
Cerebrovascular diseases
[55.3, 80.4)
[80.4, 85.1)
[85.1, 88.7)
[88.7, 92.3)
[92.3, 96.9)
[96.9,103.5)
[103.5,311.6]
Smoothed Standardised Mortality Ratios (sSMR)
with respect to EU

Turin, Males, 1995 - 2008
Cerebrovascular diseases

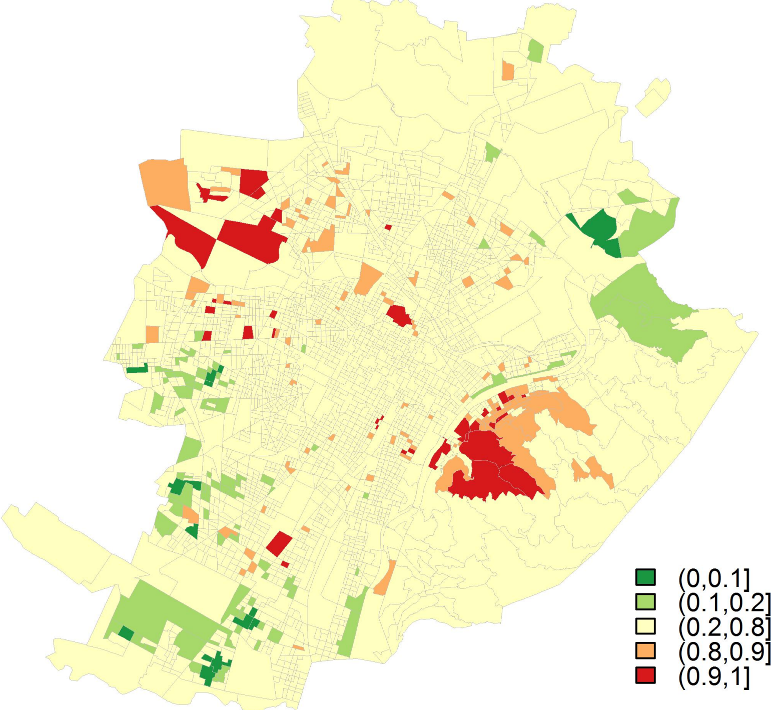

Turin, Males, 1995 - 2008
Renal failure

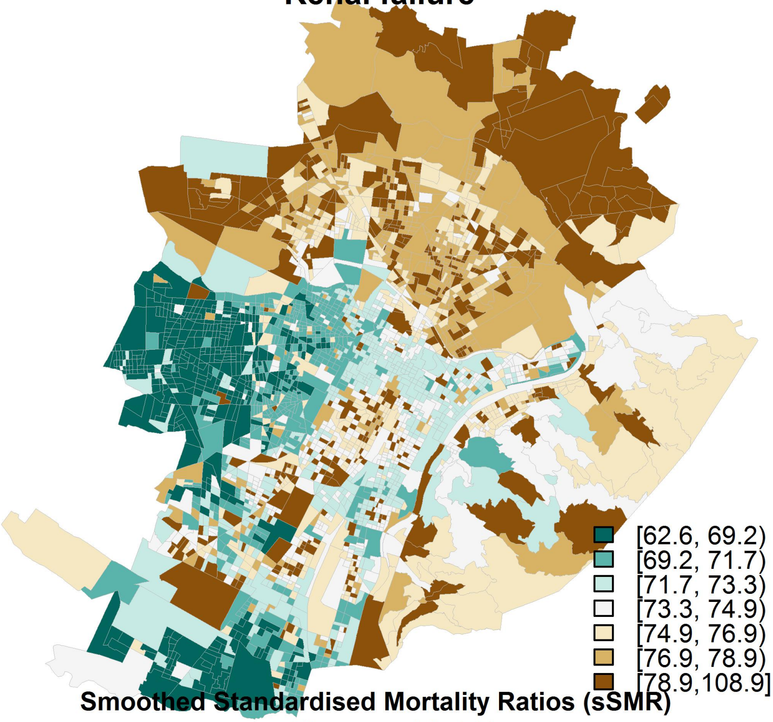

Smoothed Standardised Mortality Ratios (sSMR)
with respect to EU

Turin, Males, 1995 - 2008
Renal failure

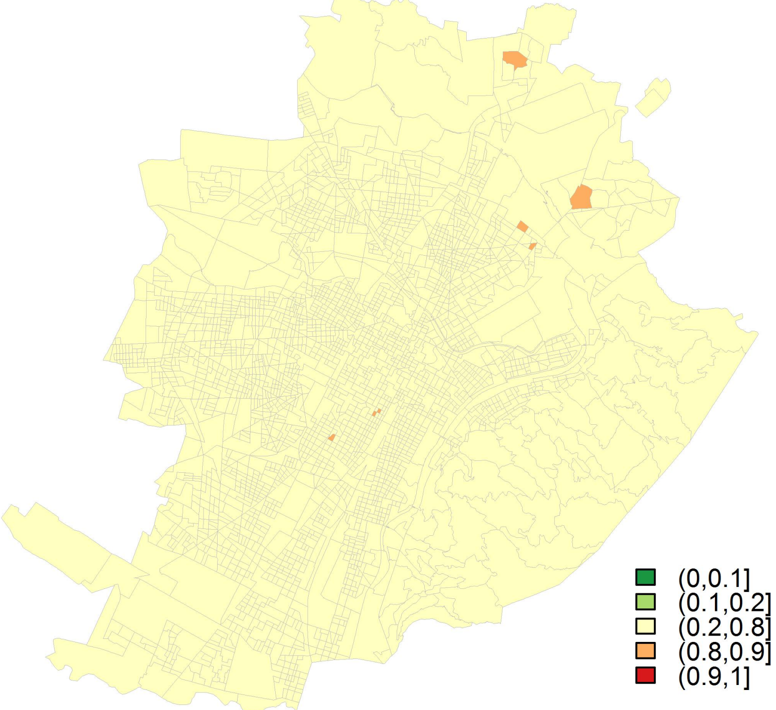

Turin, Females, 1995 - 2008
MN colon

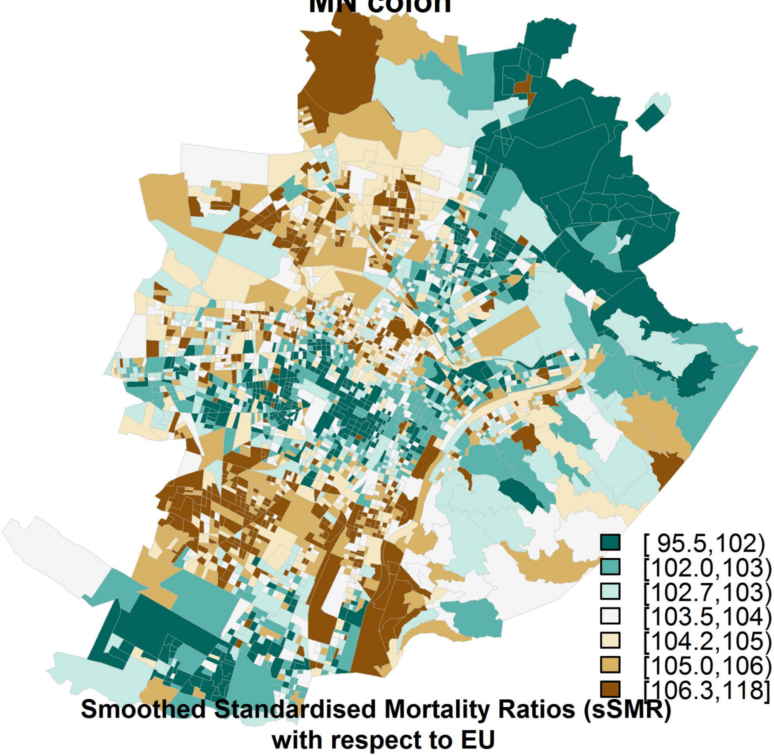

Smoothed Standardised Mortality Ratios (sSMR) with respect to EU

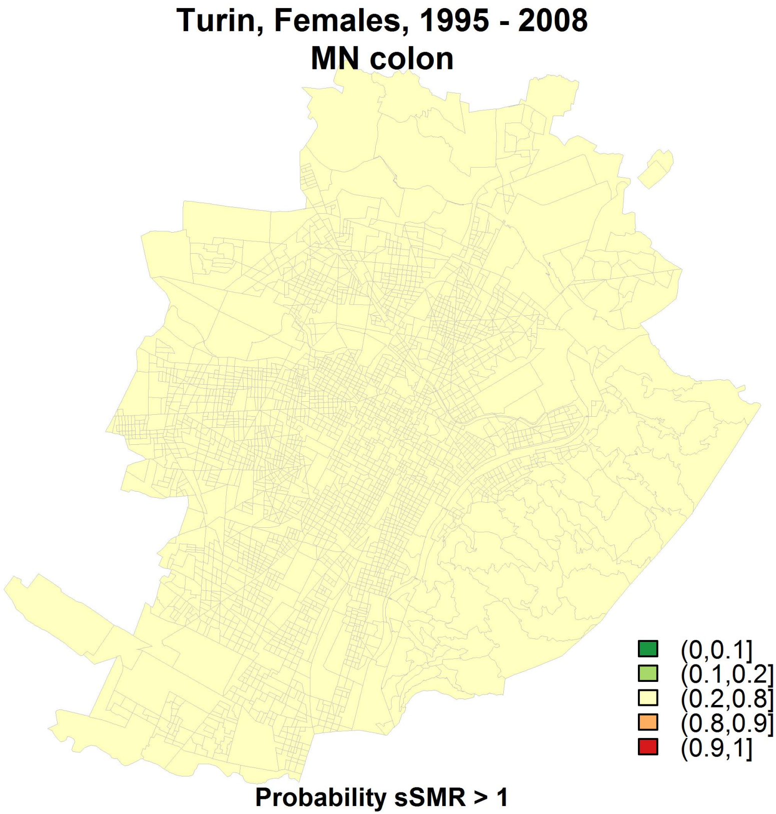
Turin, Females, 1995 - 2008
MN colon
(0,0.1]
(0.1,0.2]
(0.2,0.8]
(0.8,0.9]
(0.9,1]
Probability sSMR > 1

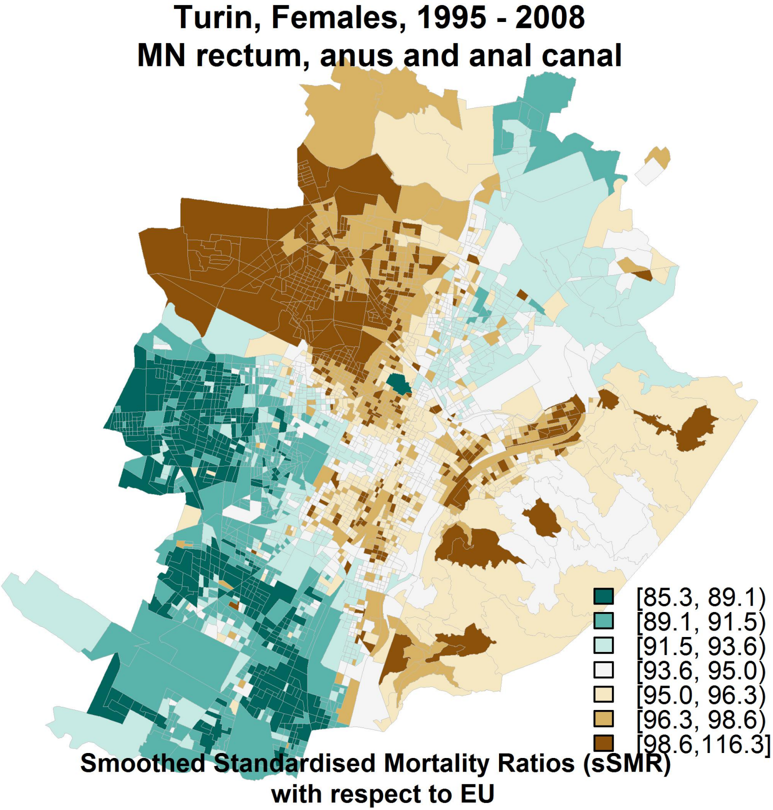

Turin, Females, 1995 - 2008
MN rectum, anus and anal canal
[85.3, 89.1)
[89.1, 91.5)
[91.5, 93.6)
[93.6, 95.0)
[95.0, 96.3)
[96.3, 98.6)
[98.6,116.3]
Smoothed Standardised Mortality Ratios (sSMR)
with respect to EU

Turin, Females, 1995 - 2008
MN rectum, anus and anal canal

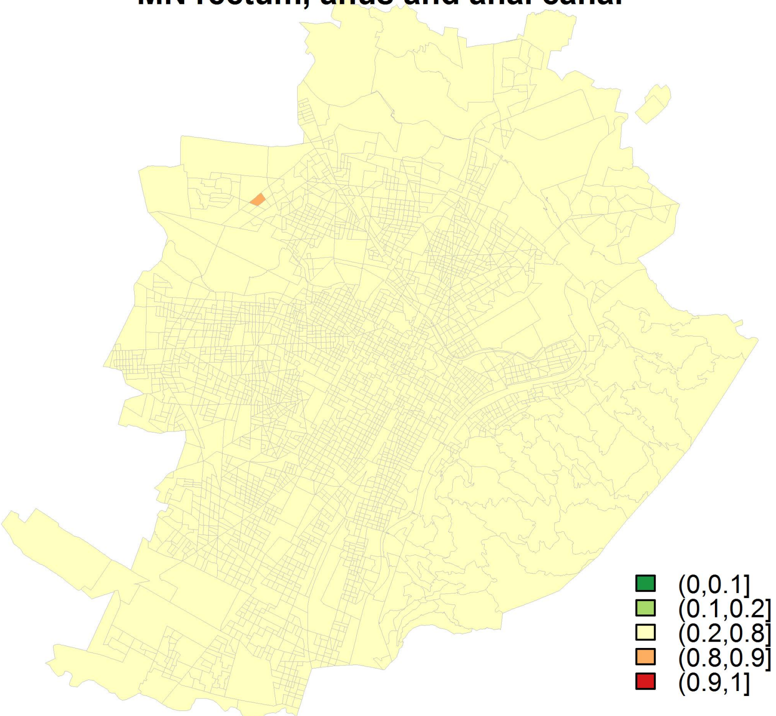

Probability sSMR > 1

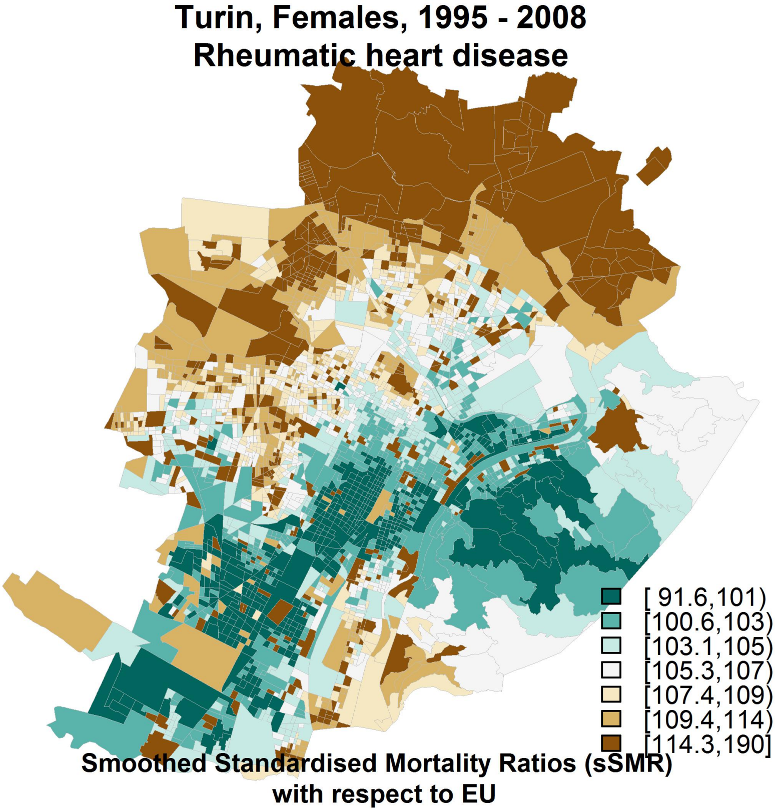
Turin, Females, 1995 - 2008
Rheumatic heart disease
[91.6,101)
[100.6,103)
[103.1,105)
[105.3,107)
[107.4,109)
[109.4,114)
[114.3,190]
Smoothed Standardised Mortality Ratios (sSMR)
with respect to EU

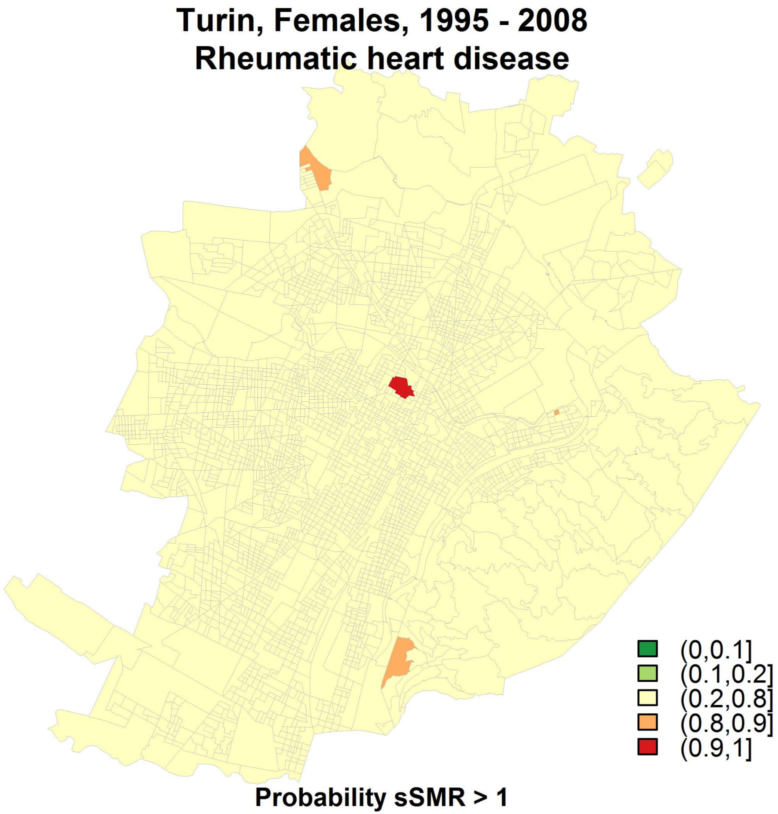
Turin, Females, 1995 - 2008
Rheumatic heart disease
(0,0.1]
(0.1,0.2]
(0.2,0.8]
(0.8,0.9]
(0.9,1]
Probability sSMR > 1

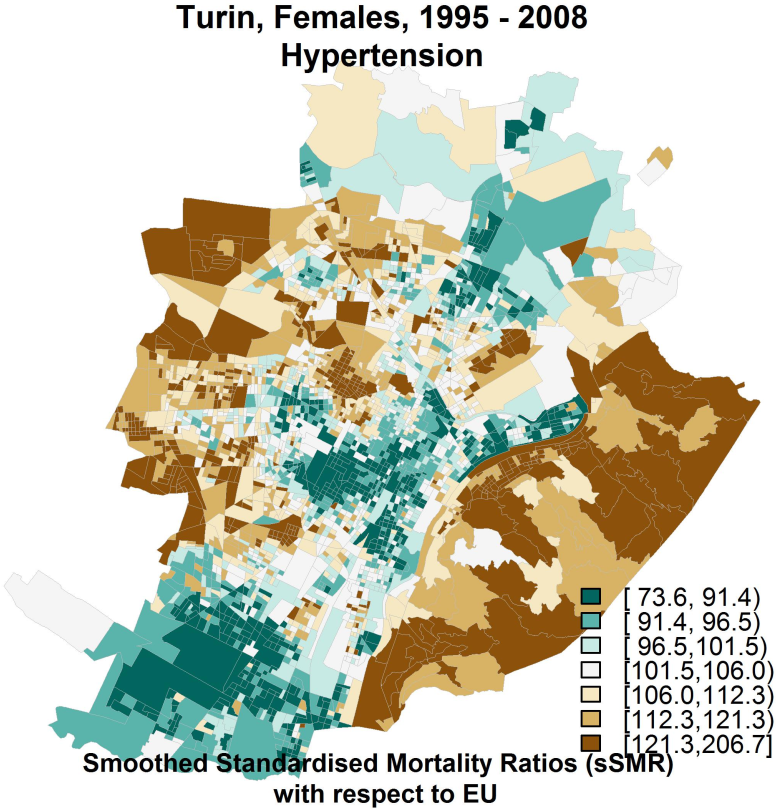
Turin, Females, 1995 - 2008
Hypertension
[73.6, 91.4)
[91.4, 96.5)
[96.5,101.5)
[101.5,106.0)
[106.0,112.3)
[112.3,121.3)
[121.3,206.7]
Smoothed Standardised Mortality Ratios (sSMR)
with respect to EU

Turin, Females, 1995 - 2008
Hypertension

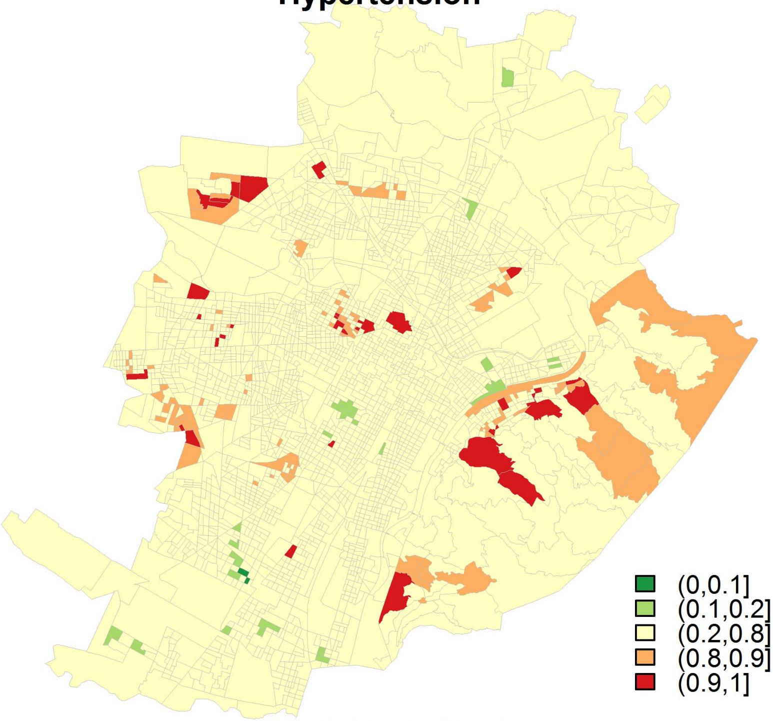

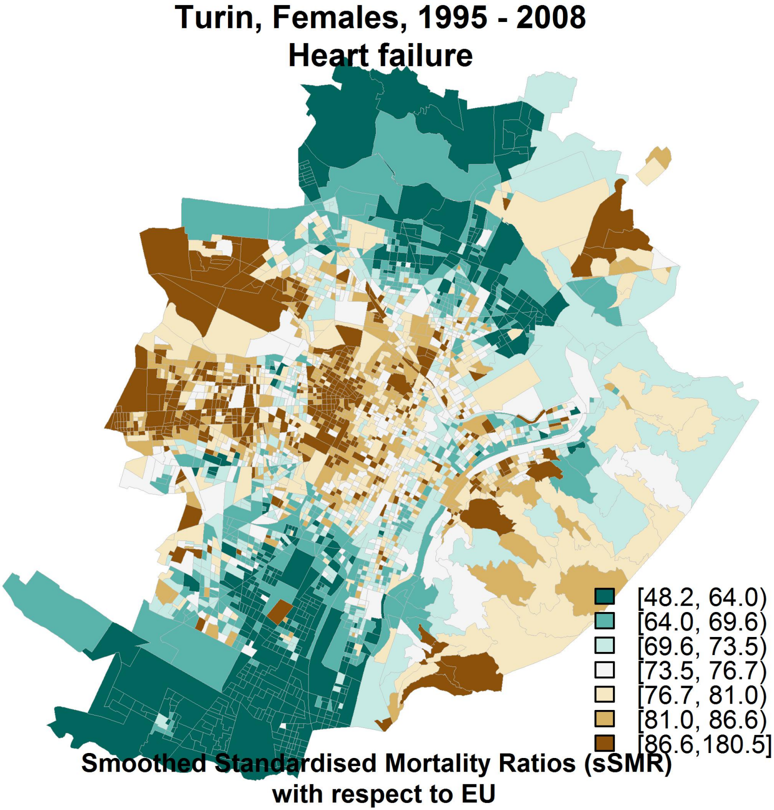
Turin, Females, 1995 - 2008
Heart failure
[48.2, 64.0)
[64.0, 69.6)
[69.6, 73.5)
[73.5, 76.7)
[76.7, 81.0)
[81.0, 86.6)
[86.6,180.5]
Smoothed Standardised Mortality Ratios (sSMR)
with respect to EU

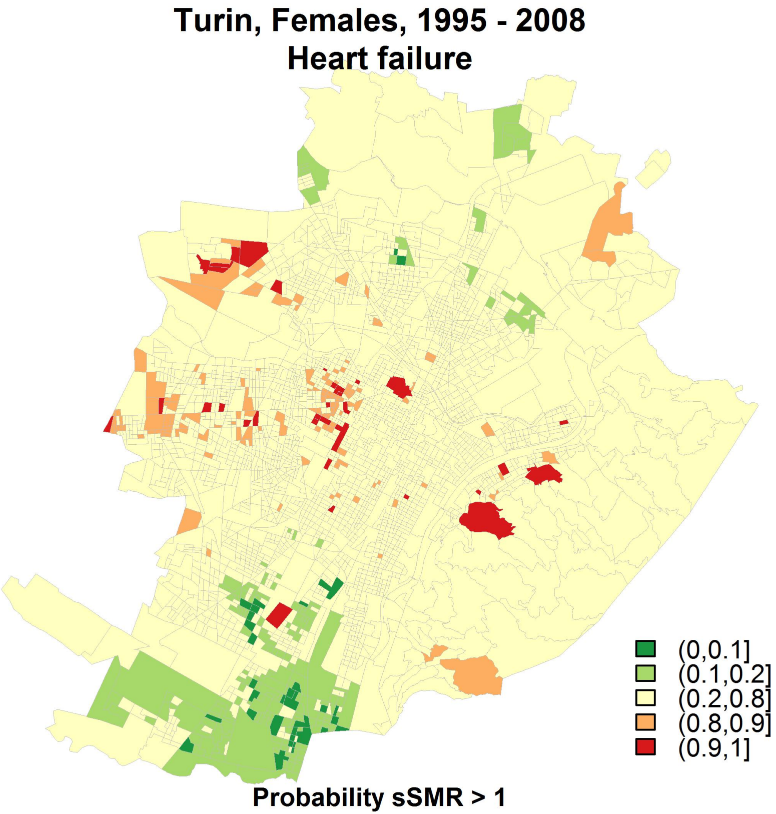
Turin, Females, 1995 - 2008
Heart failure
(0,0.1]
(0.1,0.2]
(0.2,0.8]
(0.8,0.9]
(0.9,1]
Probability sSMR > 1

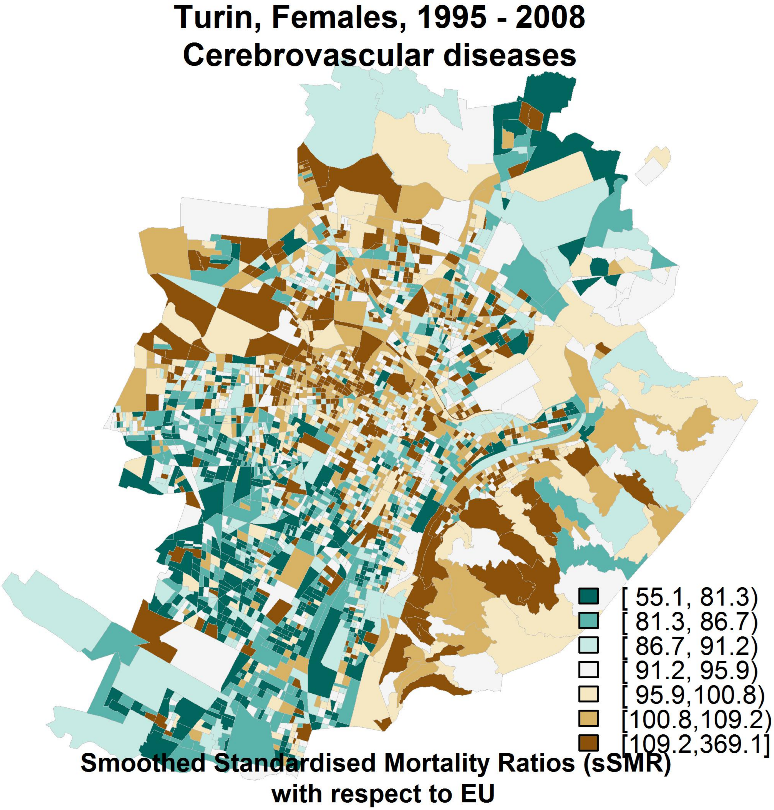
Turin, Females, 1995 - 2008
Cerebrovascular diseases
[55.1, 81.3)
[81.3, 86.7)
[86.7, 91.2)
[91.2, 95.9)
[95.9,100.8)
[100.8,109.2)
[109.2,369.1]
Smoothed Standardised Mortality Ratios (sSMR)
with respect to EU

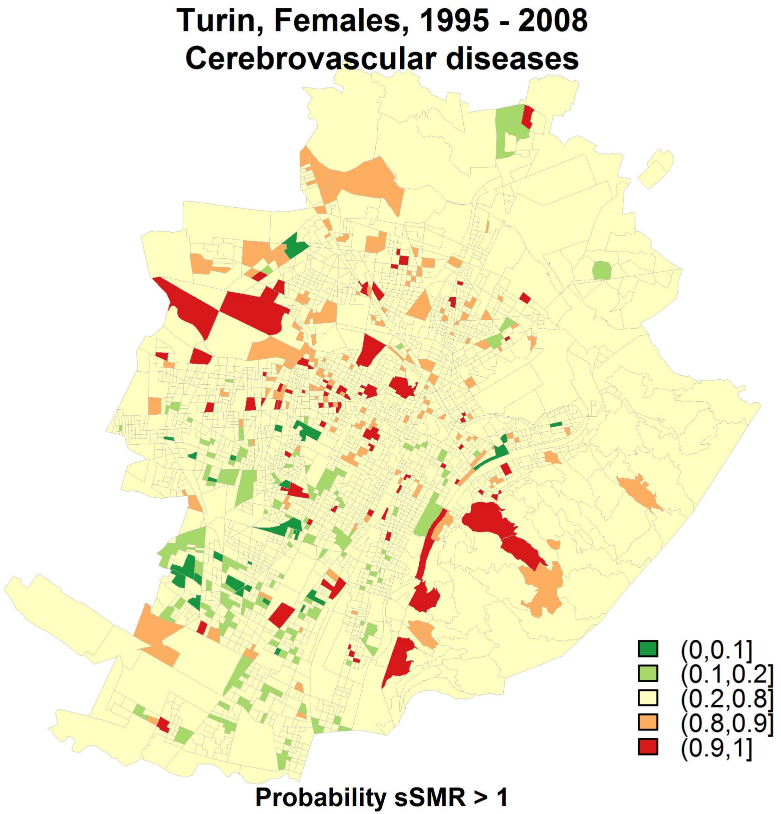
Turin, Females, 1995 - 2008
Cerebrovascular diseases
(0,0.1]
(0.1,0.2]
(0.2,0.8]
(0.8,0.9]
(0.9,1]
Probability sSMR > 1

Turin, Females, 1995 - 2008
Renal failure

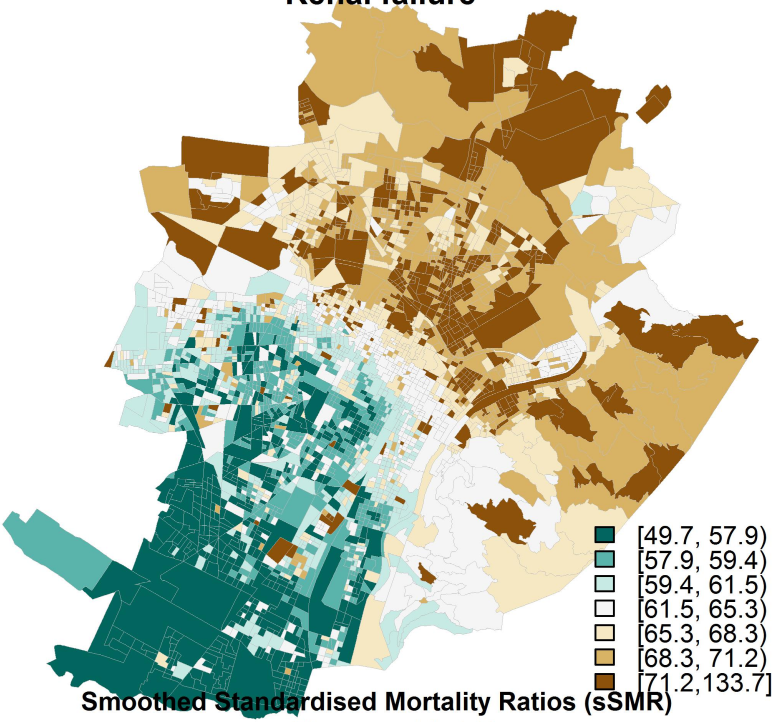

Smoothed Standardised Mortality Ratios (sSMR) with respect to EU

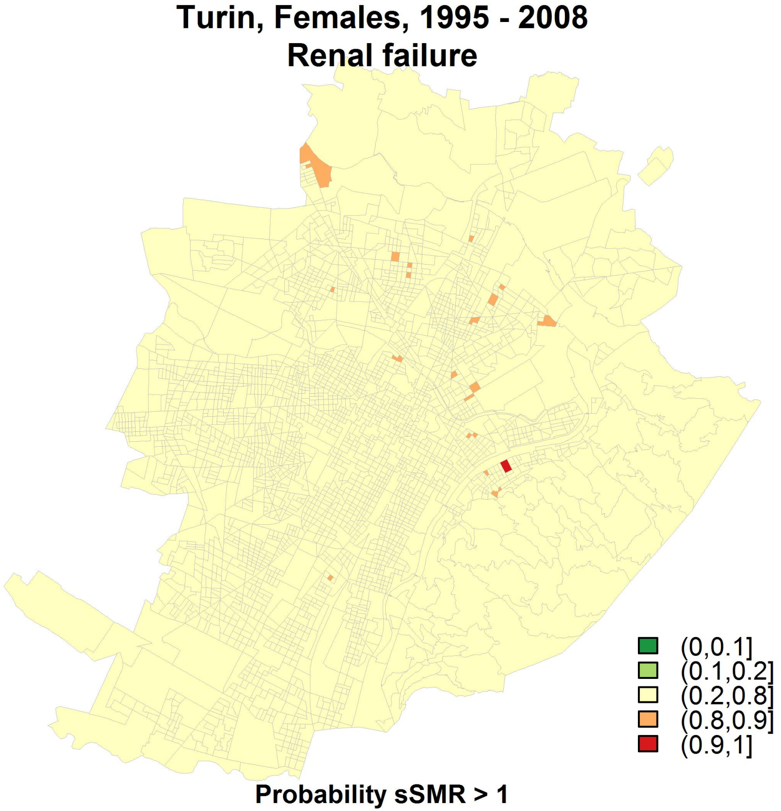
Turin, Females, 1995 - 2008
Renal failure
(0,0.1]
(0.1,0.2]
(0.2,0.8]
(0.8,0.9]
(0.9,1]
Probability sSMR > 1
