# Supplementary material for: Social differences in avoidable mortality between small areas of 15 European cities: an ecological study
Source: Int J Health Geogr. 2014 Mar 12;13:8. doi: 10.1186/1476-072X-13-8 (PMC4007807; doi:10.1186/1476-072X-13-8)

Zurich, Males, 1995 - 2008
AIDS (HIV disease)

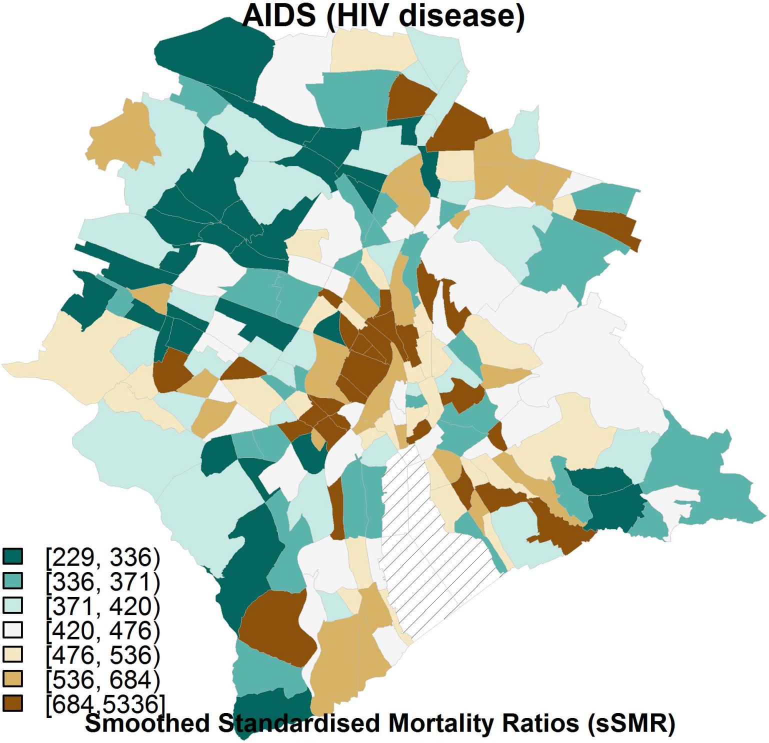

Smoothed Standardised Mortality Ratios (sSMR)
with respect to EU

Zurich, Males, 1995 - 2008
AIDS (HIV disease)

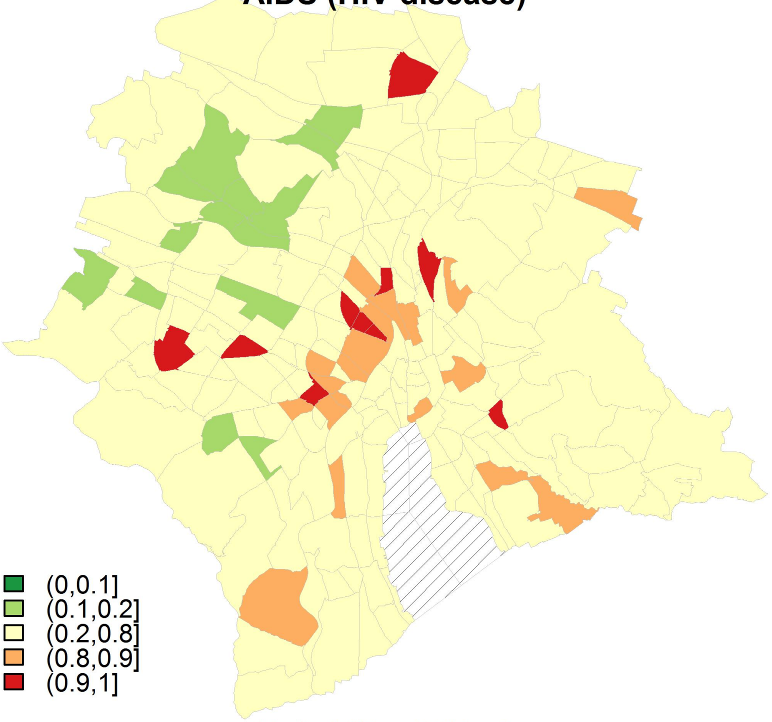

Probability sSMR > 1

Zurich, Males, 1995 - 2008
MN colon

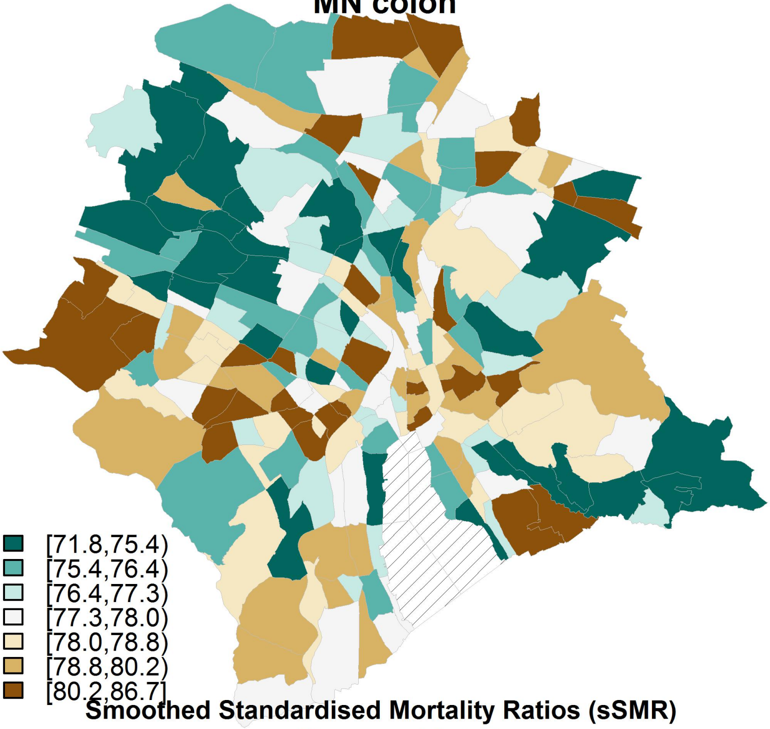

Smoothed Standardised Mortality Ratios (sSMR)
with respect to EU

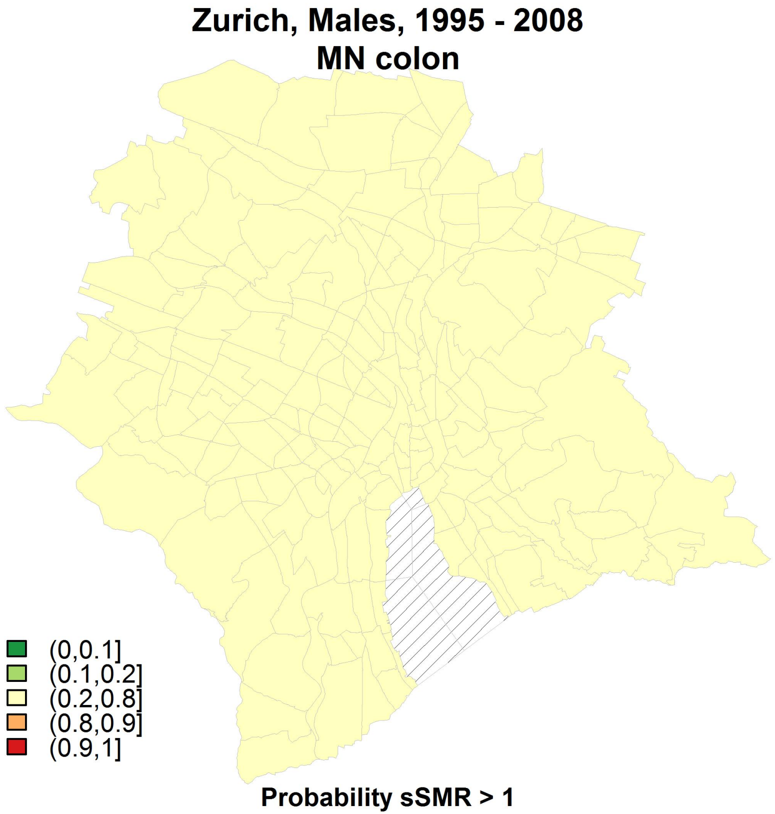
Zurich, Males, 1995 - 2008
MN colon
(0,0.1]
(0.1,0.2]
(0.2,0.8]
(0.8,0.9]
(0.9,1]
Probability sSMR > 1

Zurich, Males, 1995 - 2008
MN rectum, anus and anal canal

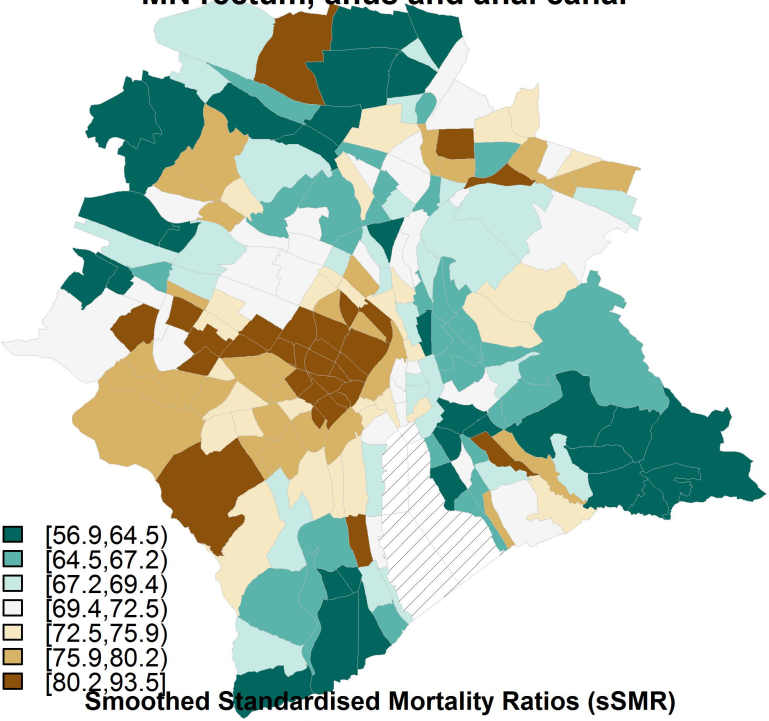

Smoothed Standardised Mortality Ratios (sSMR)
with respect to EU

Zurich, Males, 1995 - 2008
MN rectum, anus and anal canal

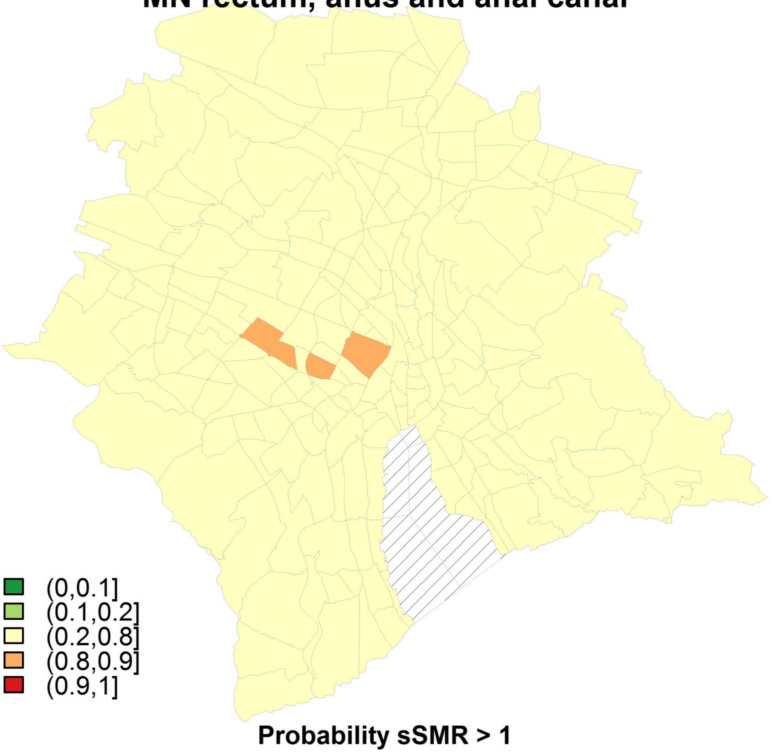

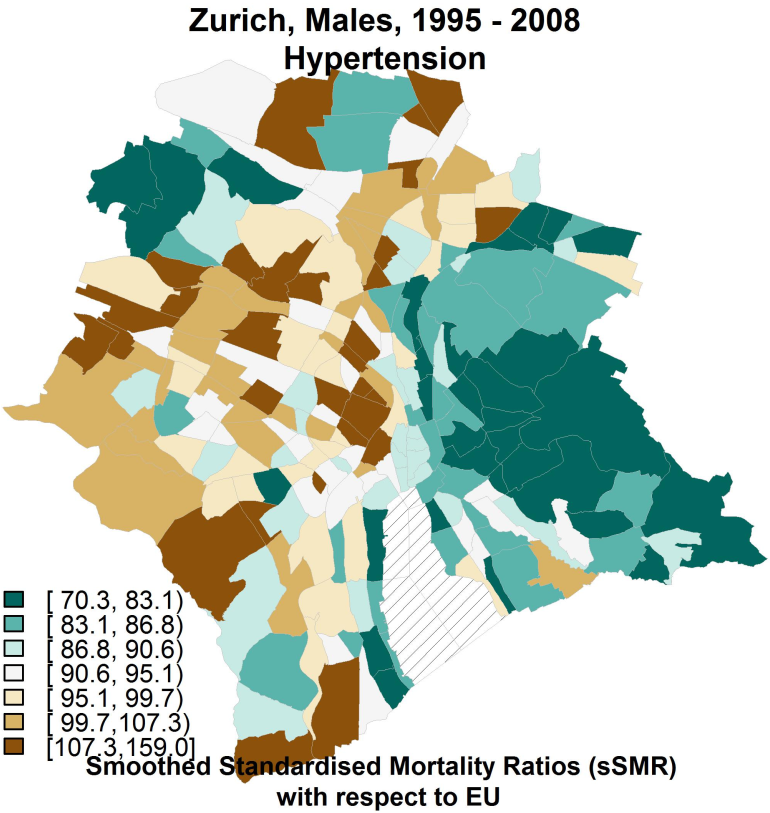
Zurich, Males, 1995 - 2008
Hypertension
[70.3, 83.1)
[83.1, 86.8)
[86.8, 90.6)
[90.6, 95.1)
[95.1, 99.7)
[99.7,107.3)
[107.3,159.0]
Smoothed Standardised Mortality Ratios (sSMR)
with respect to EU

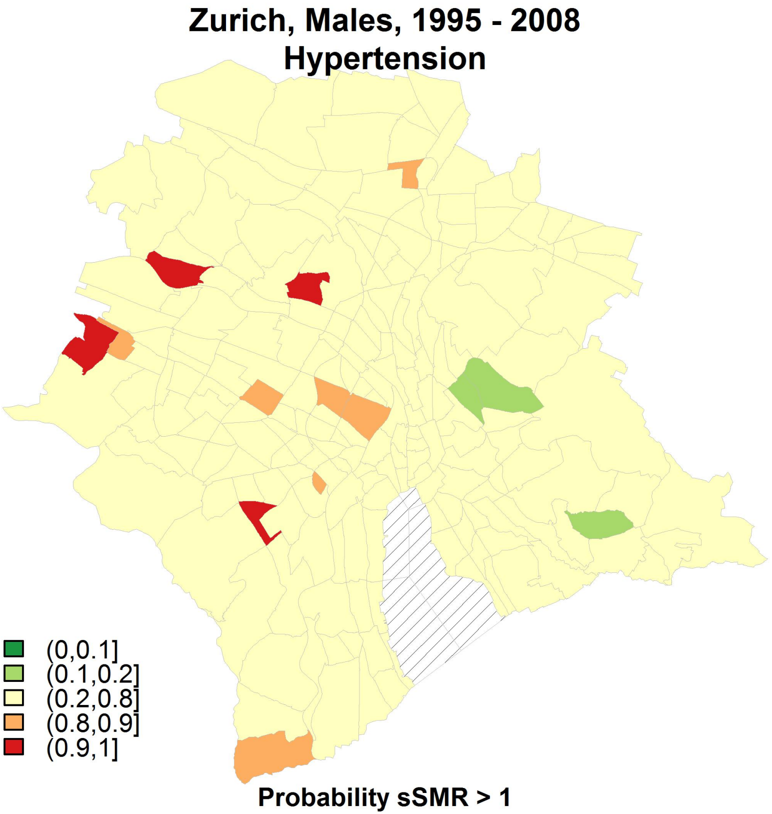
Zurich, Males, 1995 - 2008
Hypertension
(0,0.1]
(0.1,0.2]
(0.2,0.8]
(0.8,0.9]
(0.9,1]
Probability sSMR > 1

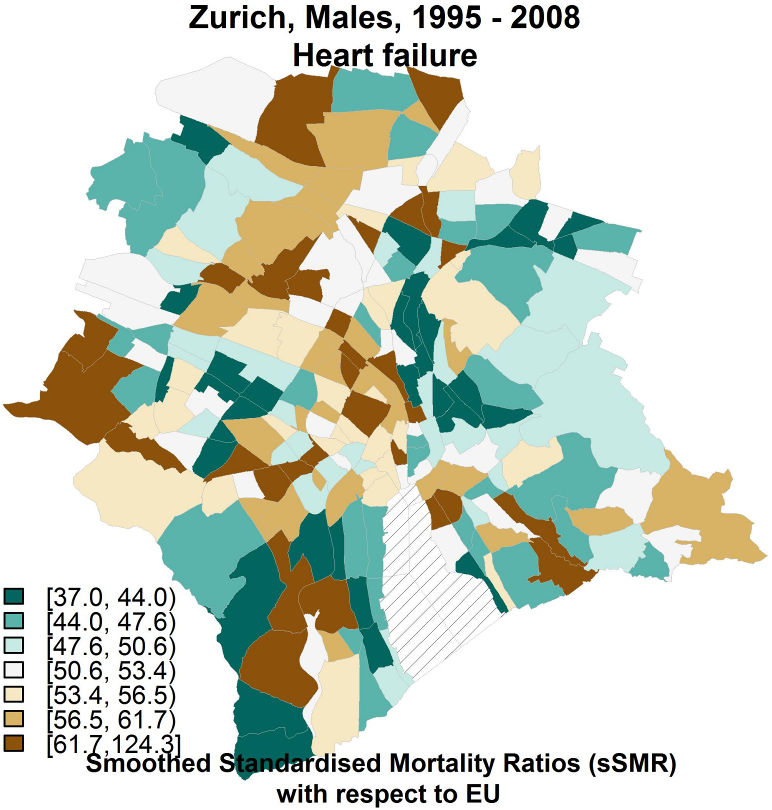
Zurich, Males, 1995 - 2008
Heart failure
[37.0, 44.0)
[44.0, 47.6)
[47.6, 50.6)
[50.6, 53.4)
[53.4, 56.5)
[56.5, 61.7)
[61.7,124.3]
Smoothed Standardised Mortality Ratios (sSMR)
with respect to EU

Zurich, Males, 1995 - 2008
Heart failure

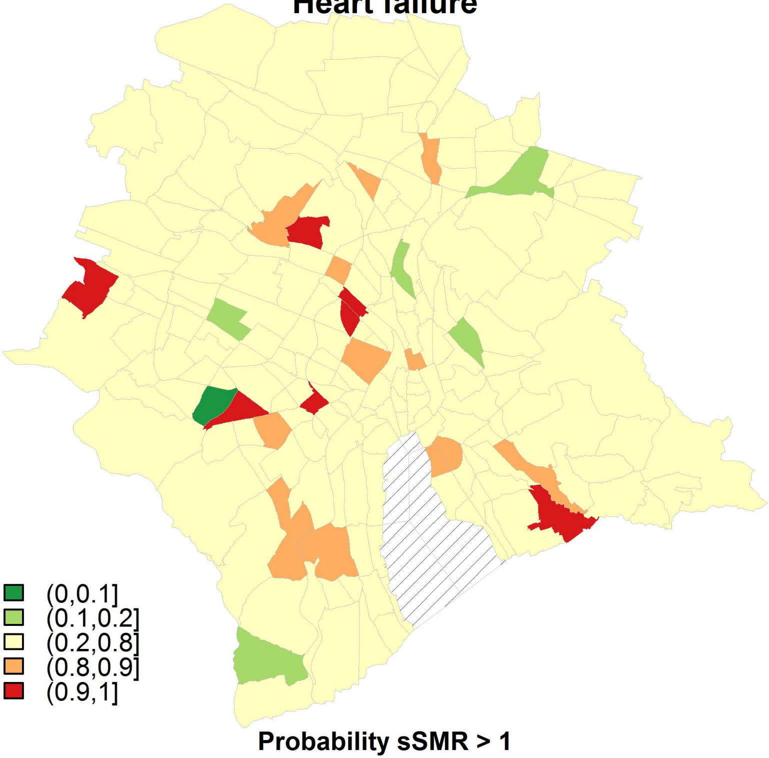

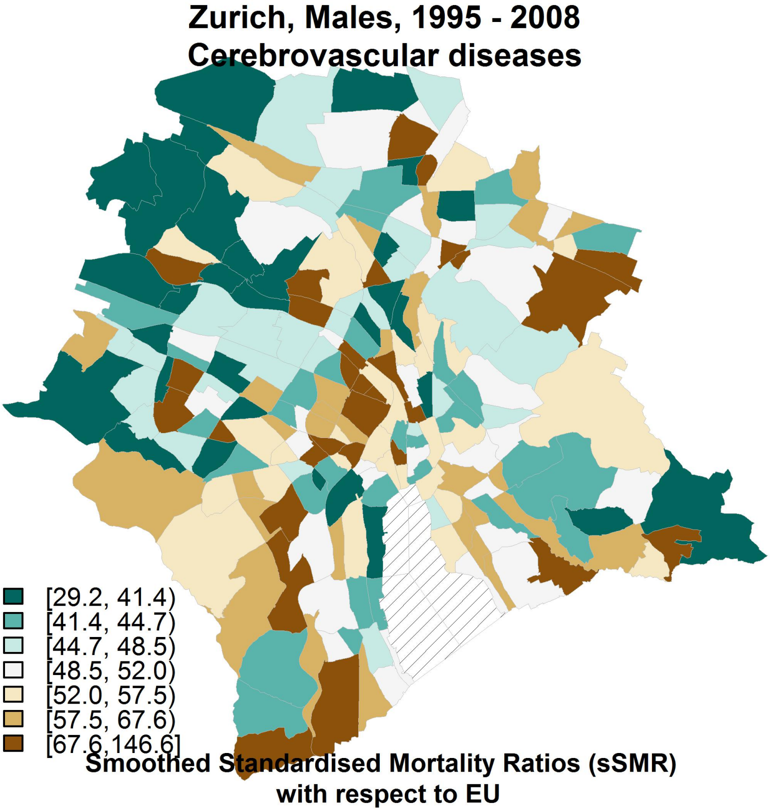
Zurich, Males, 1995 - 2008
Cerebrovascular diseases
[29.2, 41.4)
[41.4, 44.7)
[44.7, 48.5)
[48.5, 52.0)
[52.0, 57.5)
[57.5, 67.6)
[67.6,146.6]
Smoothed Standardised Mortality Ratios (sSMR)
with respect to EU

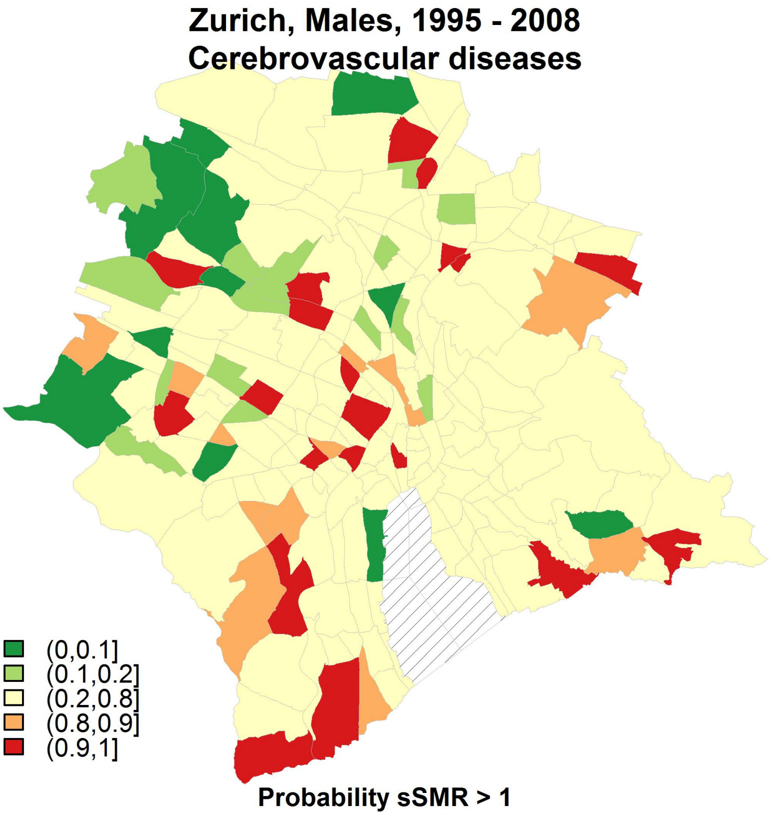

Zurich, Males, 1995 - 2008
Cerebrovascular diseases
(0,0.1]
(0.1,0.2]
(0.2,0.8]
(0.8,0.9]
(0.9,1]
Probability sSMR > 1

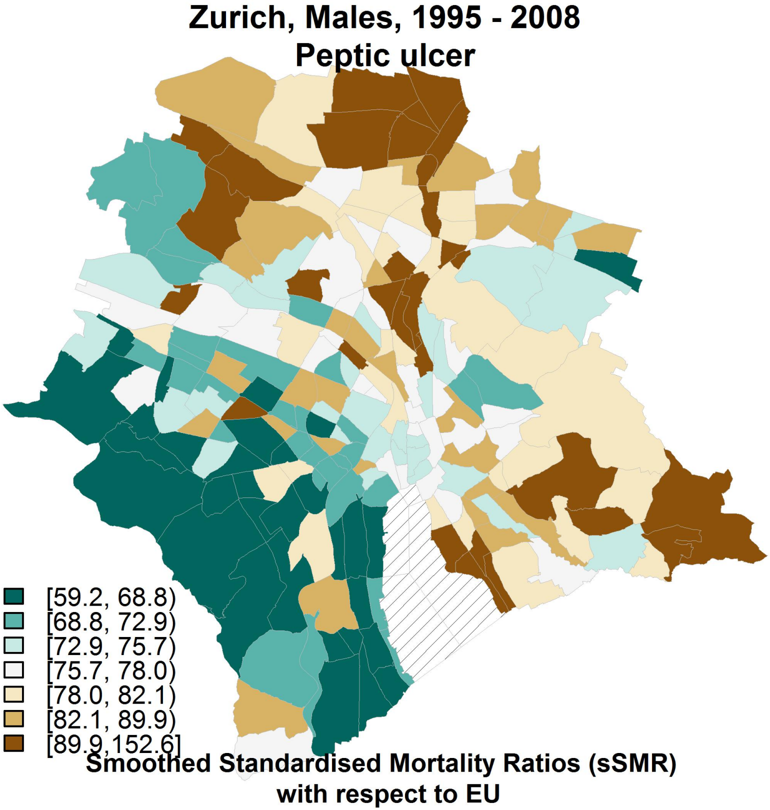
Zurich, Males, 1995 - 2008
Peptic ulcer
[59.2, 68.8)
[68.8, 72.9)
[72.9, 75.7)
[75.7, 78.0)
[78.0, 82.1)
[82.1, 89.9)
[89.9,152.6]
Smoothed Standardised Mortality Ratios (sSMR)
with respect to EU

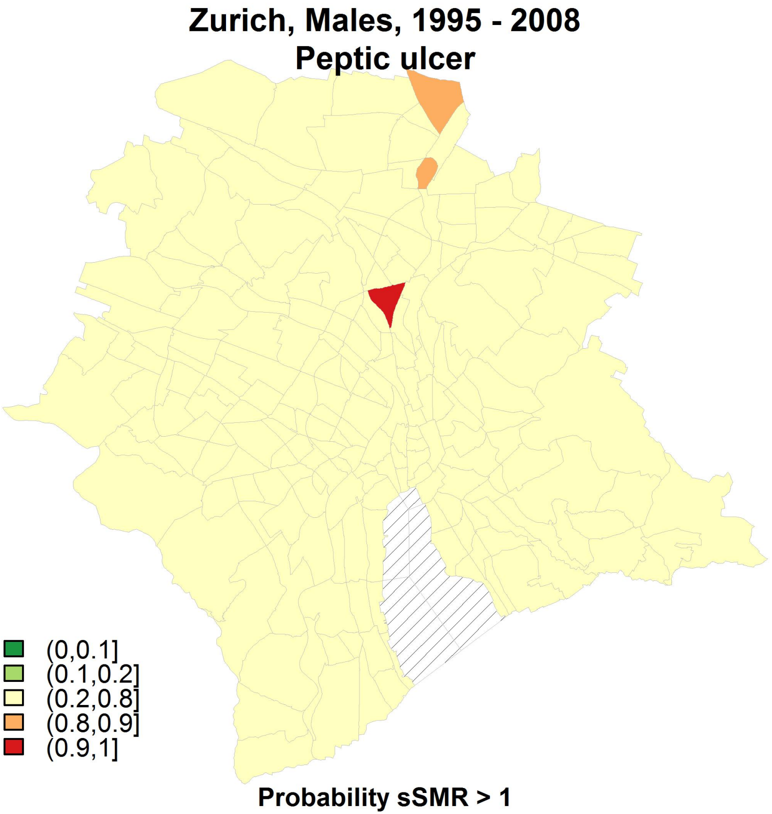
Zurich, Males, 1995 - 2008
Peptic ulcer
(0,0.1]
(0.1,0.2]
(0.2,0.8]
(0.8,0.9]
(0.9,1]
Probability sSMR > 1

Zurich, Males, 1995 - 2008
Renal failure

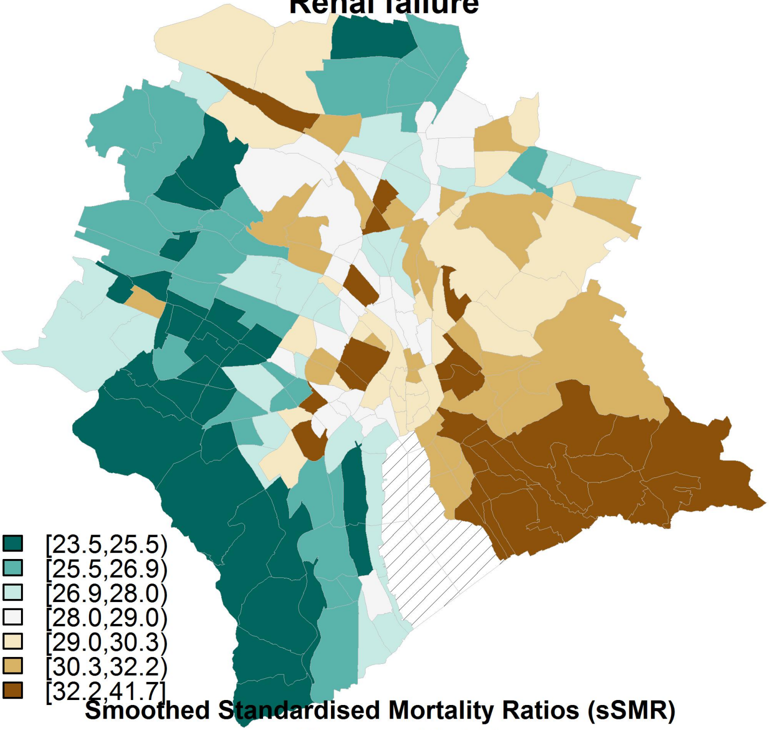

Smoothed Standardised Mortality Ratios (sSMR)
with respect to EU

Zurich, Males, 1995 - 2008
Renal failure

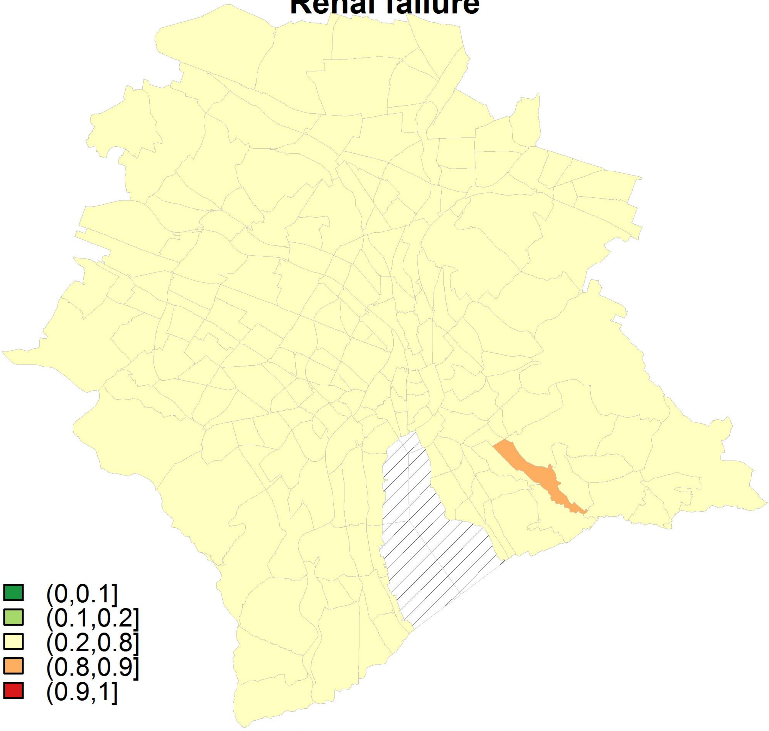

Probability sSMR > 1

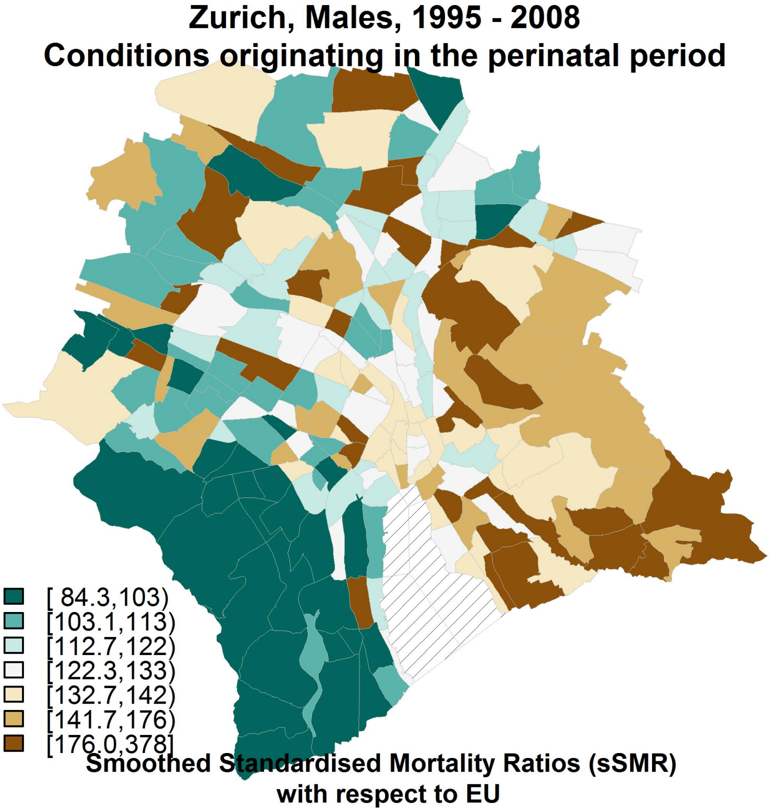
Zurich, Males, 1995 - 2008
Conditions originating in the perinatal period
[84.3,103)
[103.1,113)
[112.7,122)
[122.3,133)
[132.7,142)
[141.7,176)
[176.0,378]
Smoothed Standardised Mortality Ratios (sSMR)
with respect to EU

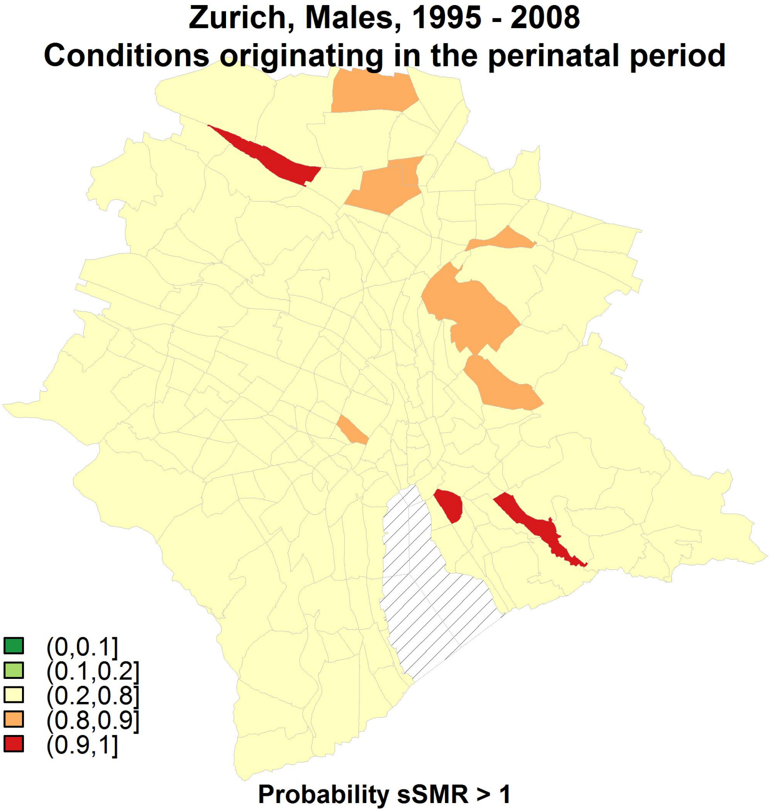
Zurich, Males, 1995 - 2008
Conditions originating in the perinatal period
(0,0.1]
(0.1,0.2]
(0.2,0.8]
(0.8,0.9]
(0.9,1]
Probability sSMR > 1

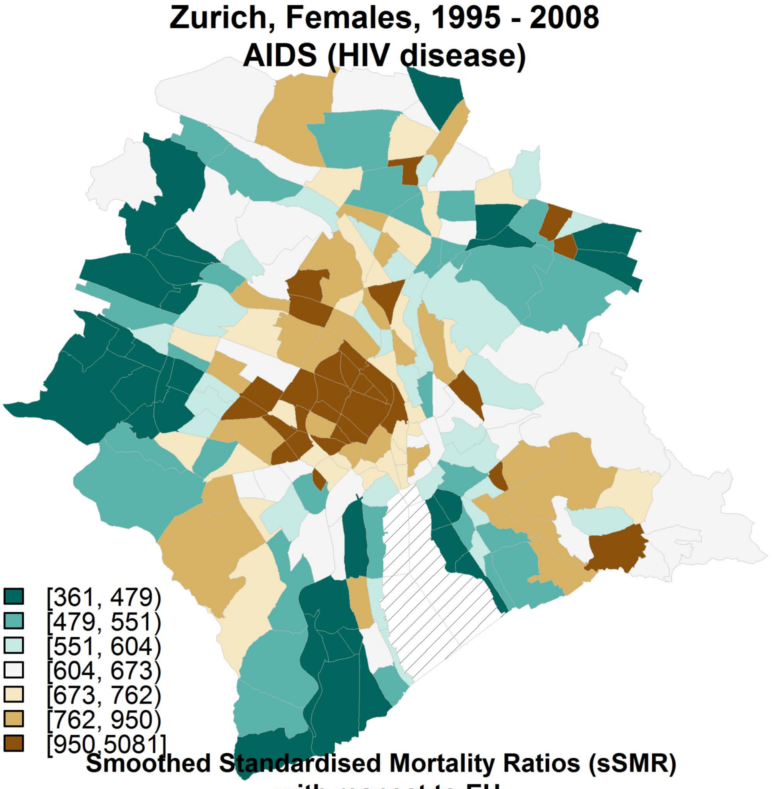
Zurich, Females, 1995 - 2008
AIDS (HIV disease)
[361, 479)
[479, 551)
[551, 604)
[604, 673)
[673, 762)
[762, 950)
[950,5081]
Smoothed Standardised Mortality Ratios (sSMR)
with respect to EU

Zurich, Females, 1995 - 2008
AIDS (HIV disease)

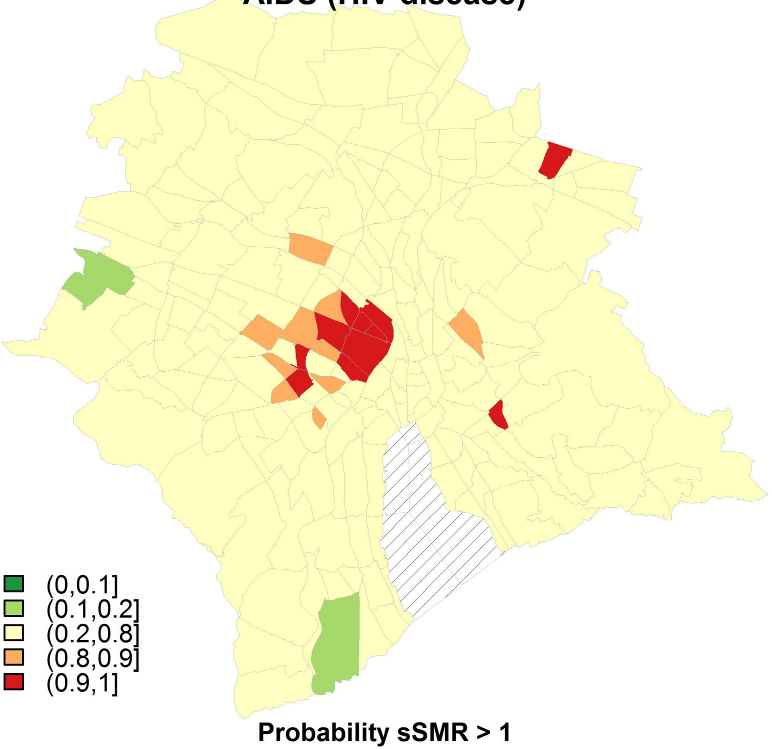

Zurich, Females, 1995 - 2008
MN colon

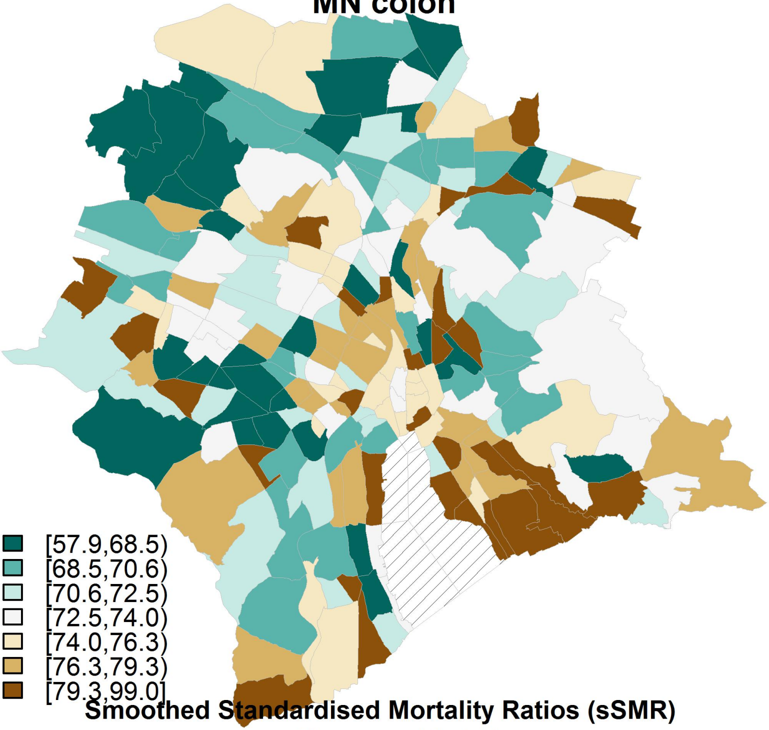

Smoothed Standardised Mortality Ratios (sSMR)
with respect to EU

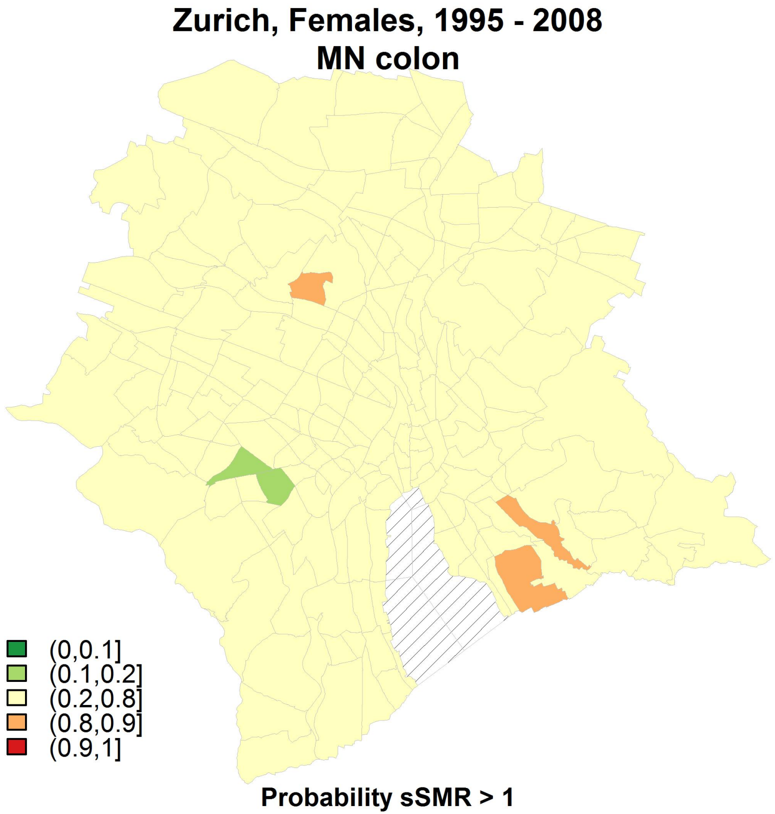
Zurich, Females, 1995 - 2008
MN colon
(0,0.1]
(0.1,0.2]
(0.2,0.8]
(0.8,0.9]
(0.9,1]
Probability sSMR > 1

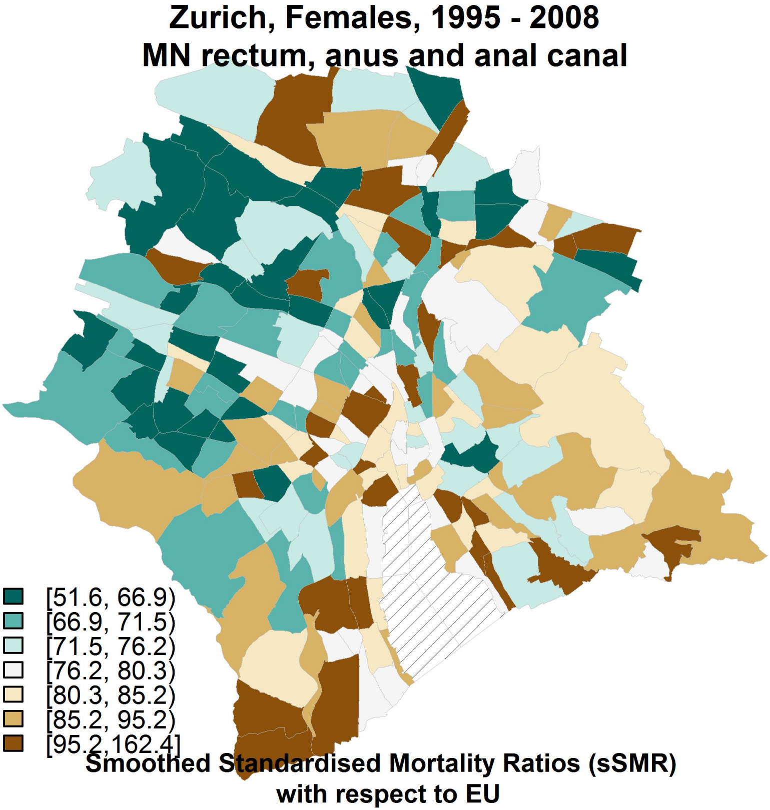
Zurich, Females, 1995 - 2008
MN rectum, anus and anal canal
[51.6, 66.9)
[66.9, 71.5)
[71.5, 76.2)
[76.2, 80.3)
[80.3, 85.2)
[85.2, 95.2)
[95.2,162.4]
Smoothed Standardised Mortality Ratios (sSMR)
with respect to EU

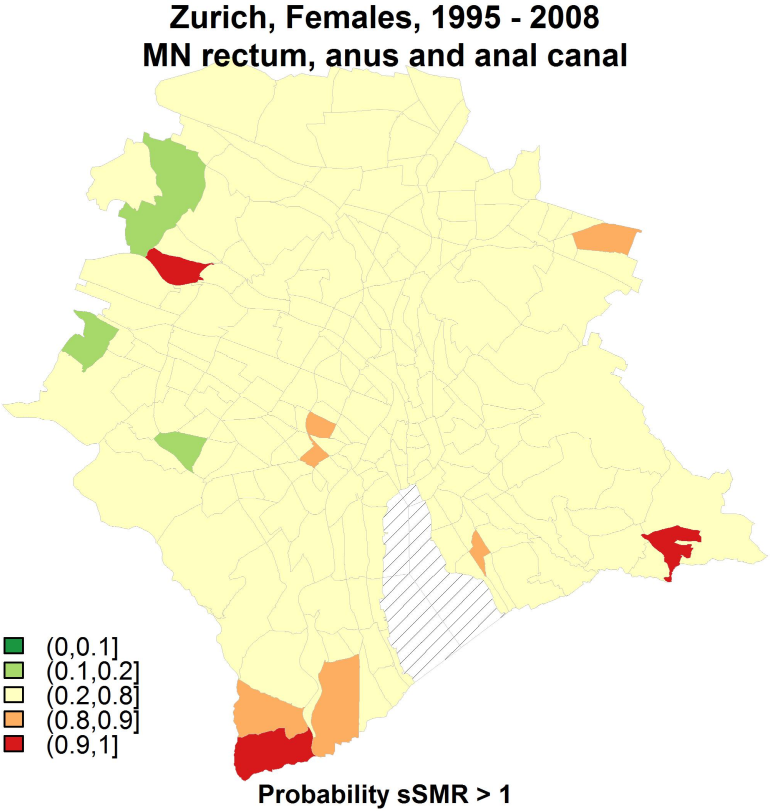
Zurich, Females, 1995 - 2008
MN rectum, anus and anal canal
(0,0.1]
(0.1,0.2]
(0.2,0.8]
(0.8,0.9]
(0.9,1]
Probability sSMR > 1

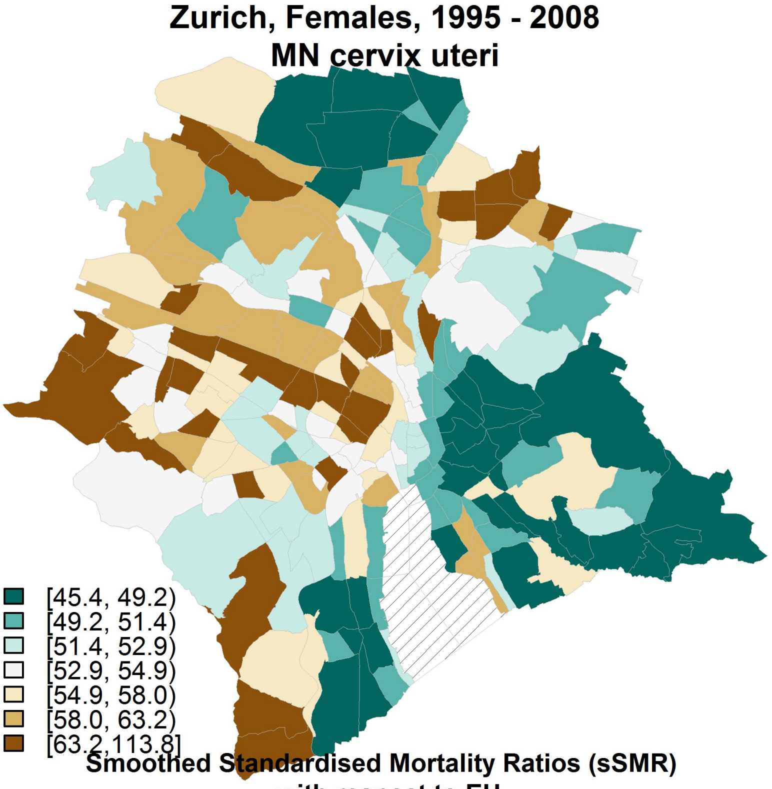
Zurich, Females, 1995 - 2008
MN cervix uteri
[45.4, 49.2)
[49.2, 51.4)
[51.4, 52.9)
[52.9, 54.9)
[54.9, 58.0)
[58.0, 63.2)
[63.2,113.8]
Smoothed Standardised Mortality Ratios (sSMR)
with respect to EU

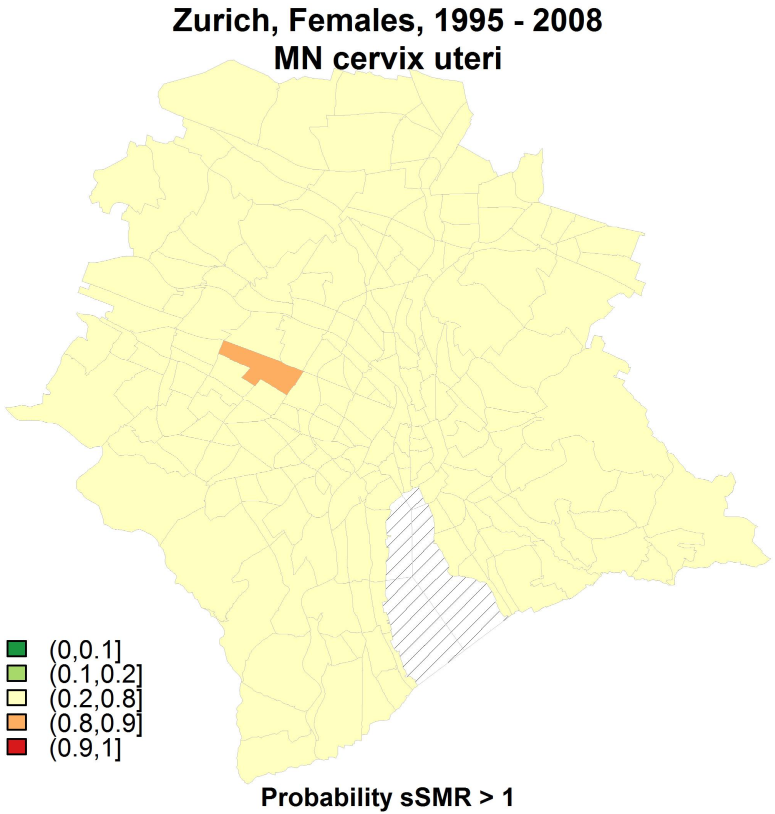
Zurich, Females, 1995 - 2008
MN cervix uteri
(0,0.1]
(0.1,0.2]
(0.2,0.8]
(0.8,0.9]
(0.9,1]
Probability sSMR > 1

Zurich, Females, 1995 - 2008
Rheumatic heart disease

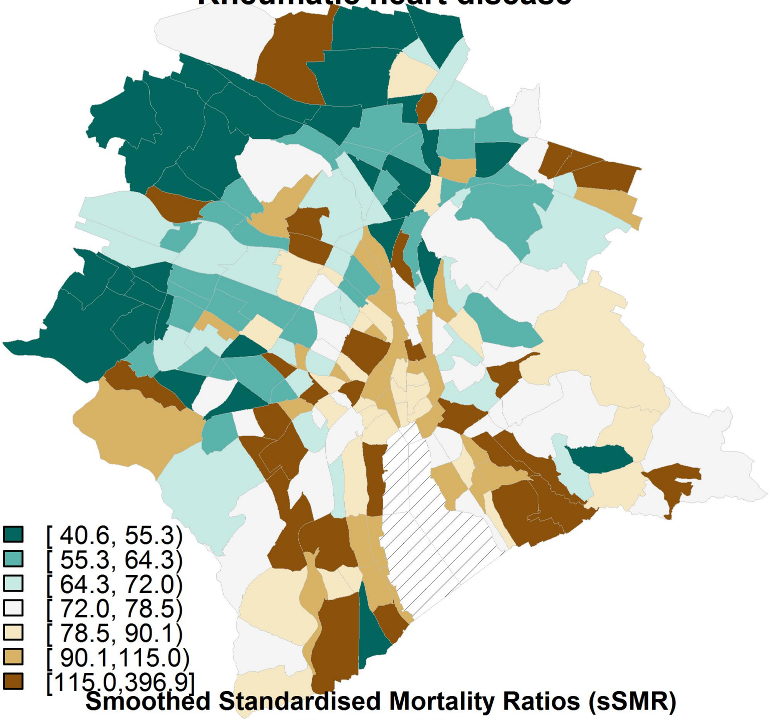

Smoothed Standardised Mortality Ratios (sSMR)
with respect to EU

Zurich, Females, 1995 - 2008
Rheumatic heart disease

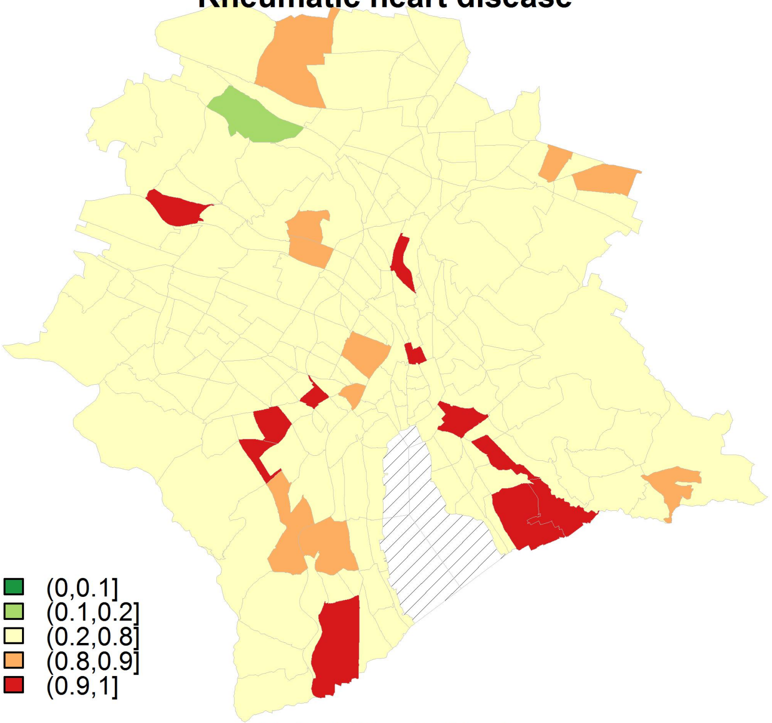

Probability sSMR > 1

Zurich, Females, 1995 - 2008
Hypertension

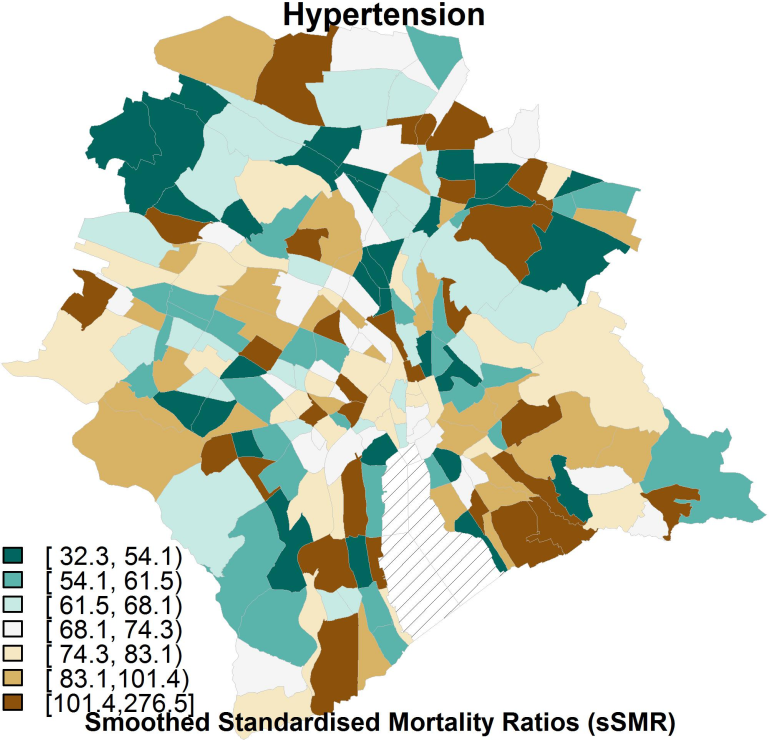

Smoothed Standardised Mortality Ratios (sSMR)
with respect to EU

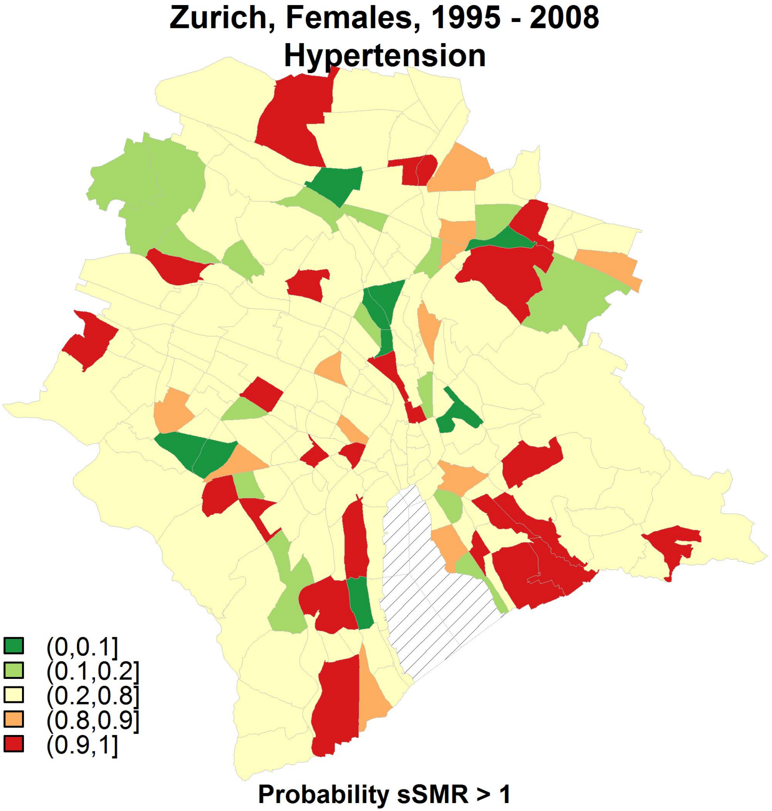
Zurich, Females, 1995 - 2008
Hypertension
(0,0.1]
(0.1,0.2]
(0.2,0.8]
(0.8,0.9]
(0.9,1]
Probability sSMR > 1

Zurich, Females, 1995 - 2008
Heart failure

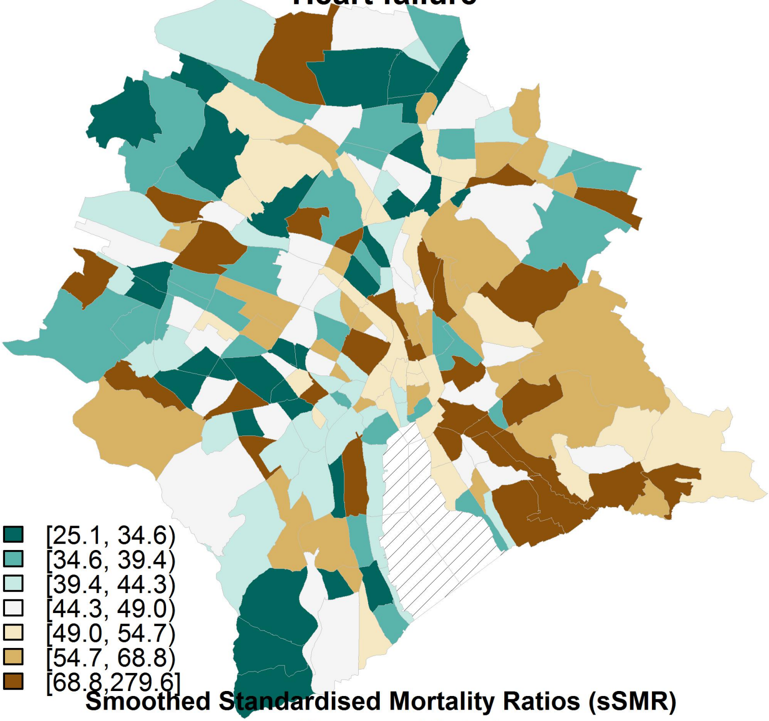

Smoothed Standardised Mortality Ratios (sSMR)
with respect to EU

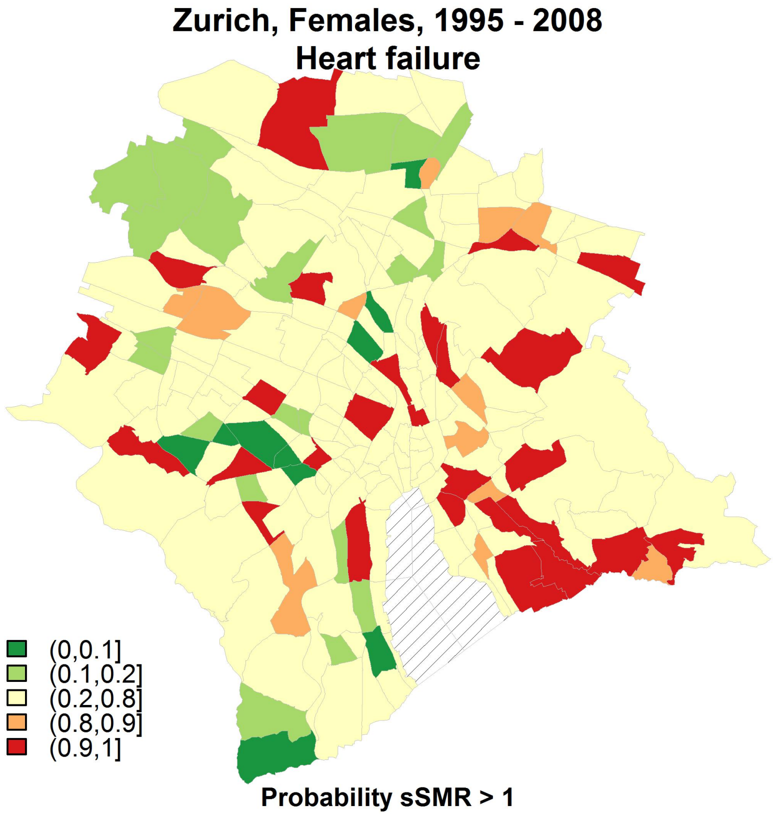
Zurich, Females, 1995 - 2008
Heart failure
(0,0.1]
(0.1,0.2]
(0.2,0.8]
(0.8,0.9]
(0.9,1]
Probability sSMR > 1

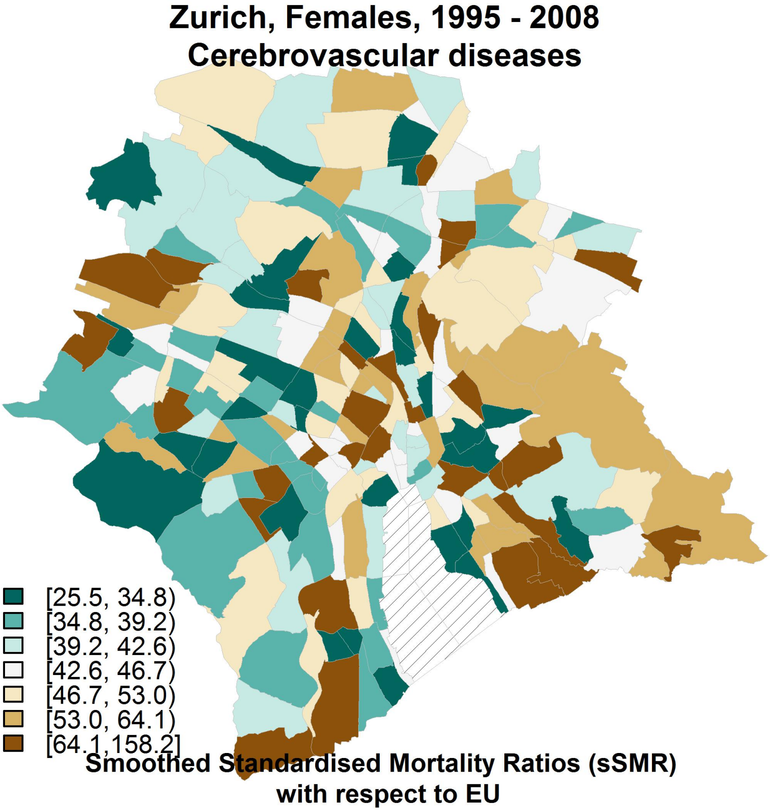

Zurich, Females, 1995 - 2008
Cerebrovascular diseases
[25.5, 34.8)
[34.8, 39.2)
[39.2, 42.6)
[42.6, 46.7)
[46.7, 53.0)
[53.0, 64.1)
[64.1,158.2]
Smoothed Standardised Mortality Ratios (sSMR)
with respect to EU

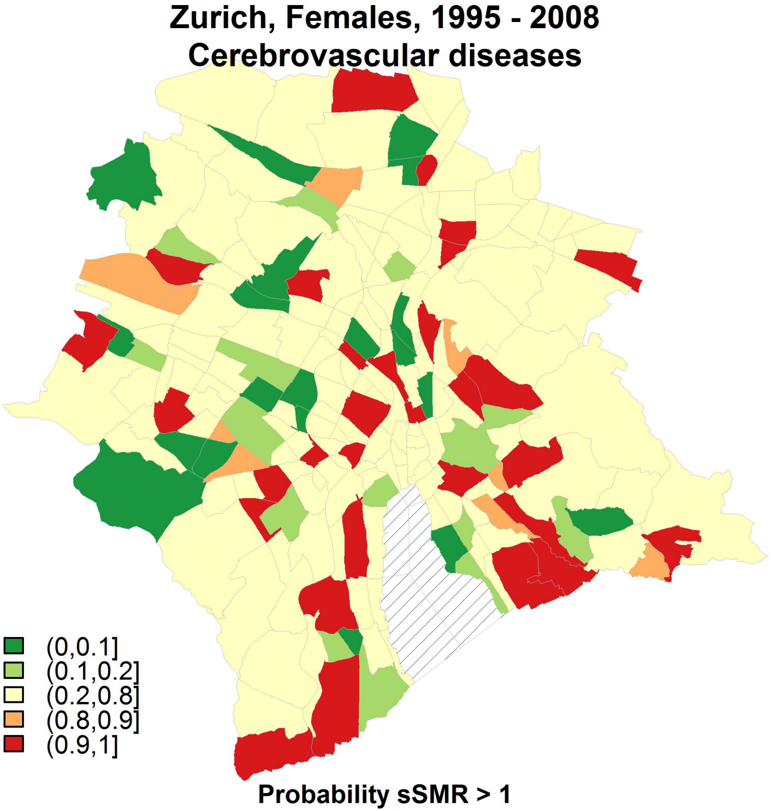
Zurich, Females, 1995 - 2008
Cerebrovascular diseases
(0,0.1]
(0.1,0.2]
(0.2,0.8]
(0.8,0.9]
(0.9,1]
Probability sSMR > 1

Zurich, Females, 1995 - 2008
Peptic ulcer

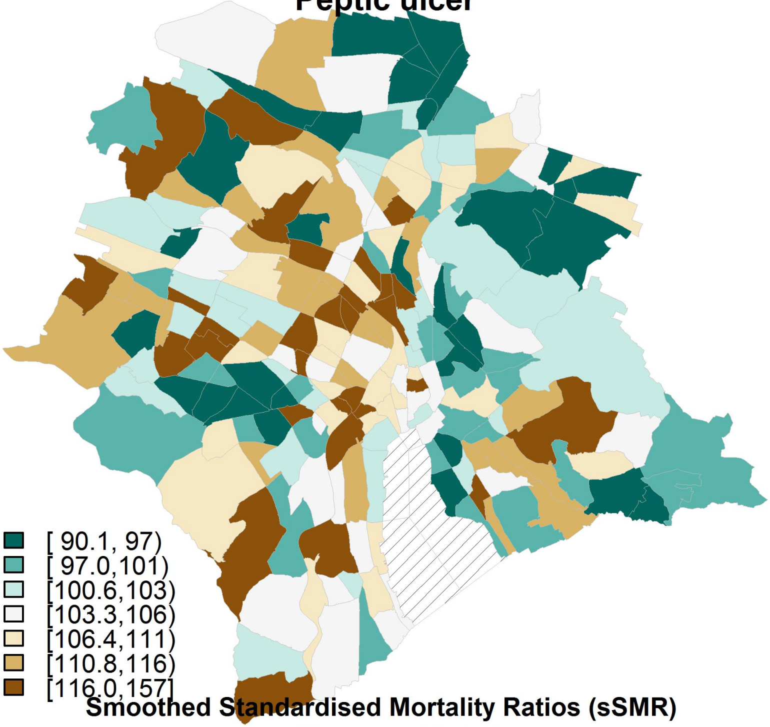

Smoothed Standardised Mortality Ratios (sSMR)
with respect to EU

Zurich, Females, 1995 - 2008
Peptic ulcer

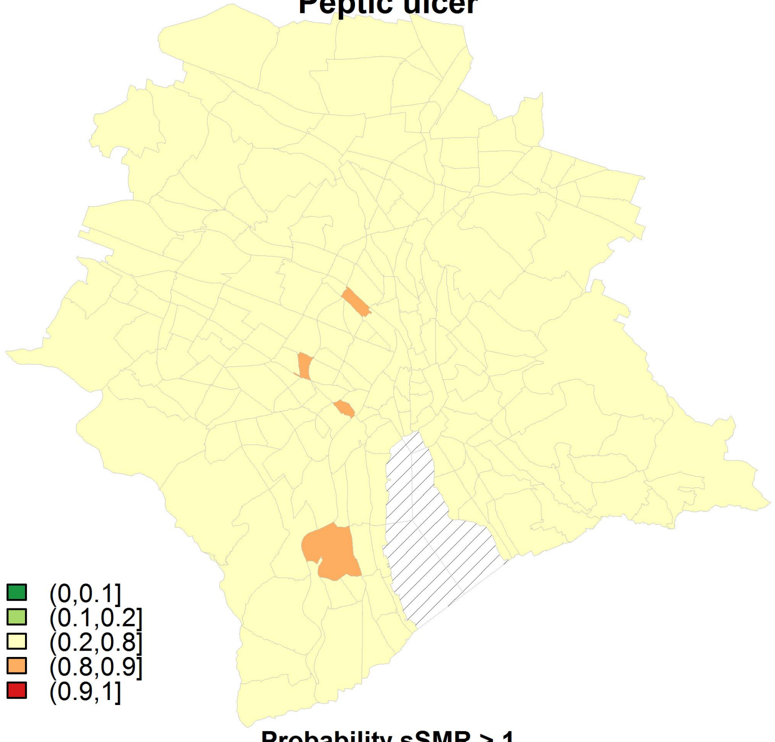

Probability sSMR > 1

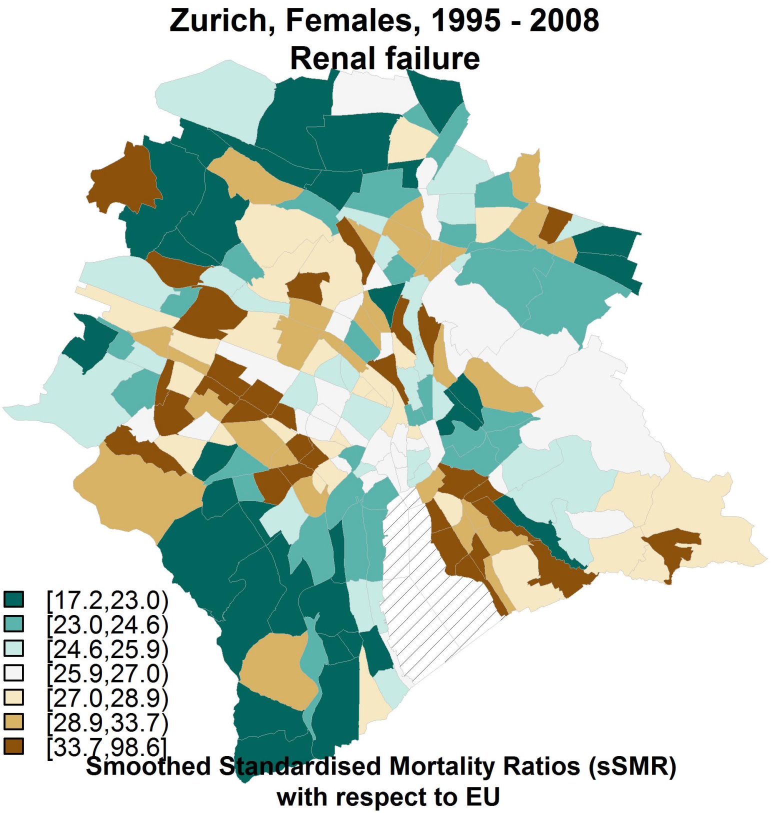
Zurich, Females, 1995 - 2008
Renal failure
[17.2,23.0)
[23.0,24.6)
[24.6,25.9)
[25.9,27.0)
[27.0,28.9)
[28.9,33.7)
[33.7,98.6]
Smoothed Standardised Mortality Ratios (sSMR)
with respect to EU

Zurich, Females, 1995 - 2008
Renal failure

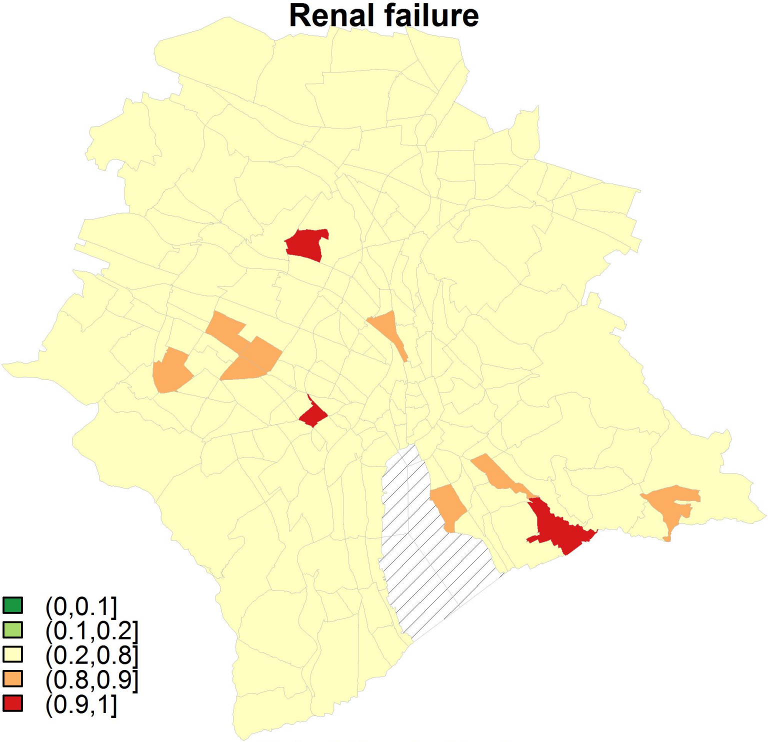

Probability sSMR > 1

Zurich, Females, 1995 - 2008
Conditions originating in the perinatal period

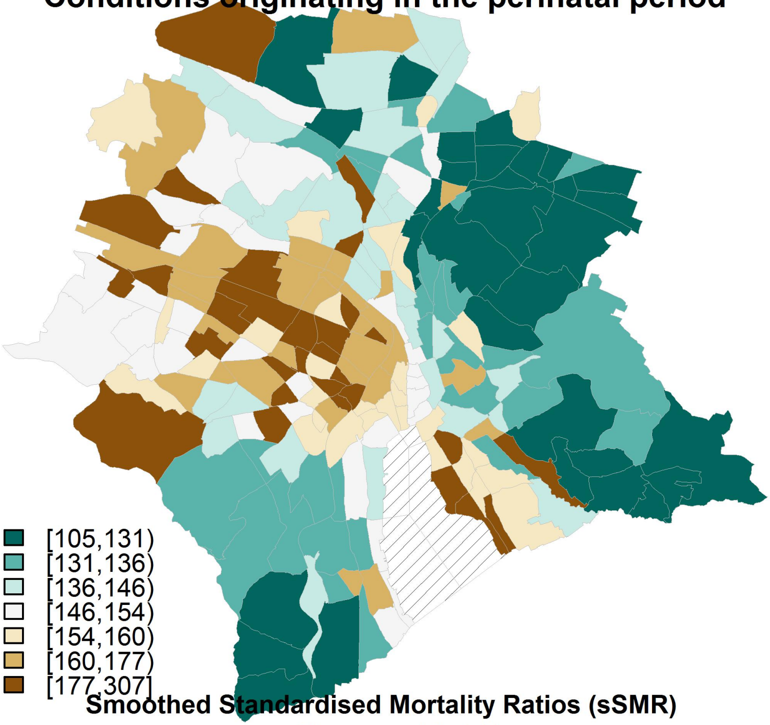

Smoothed Standardised Mortality Ratios (sSMR)
with respect to EU

Zurich, Females, 1995 - 2008
Conditions originating in the perinatal period

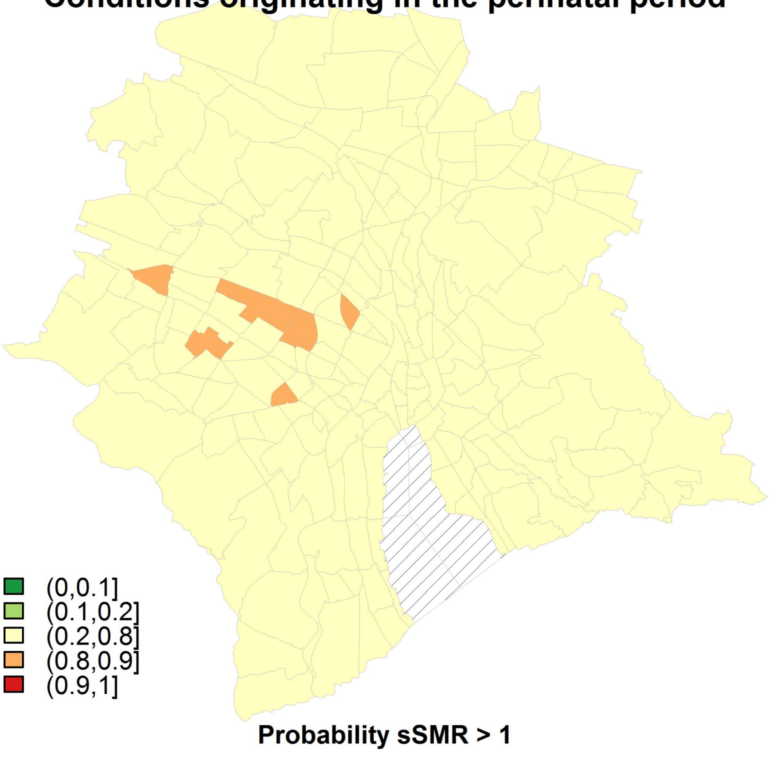

Supplement: Additional file 17 — Cause-specific mortality maps for Zurich. [file 1476-072X-13-8-S17.pdf]
