# Supplementary material for: Social differences in avoidable mortality between small areas of 15 European cities: an ecological study
Source: Int J Health Geogr. 2014 Mar 12;13:8. doi: 10.1186/1476-072X-13-8 (PMC4007807; doi:10.1186/1476-072X-13-8)
Supplement: Additional file 18 — Cause-specific box-plots graphs. [file 1476-072X-13-8-S18.pdf]

AIDS (HIV disease)

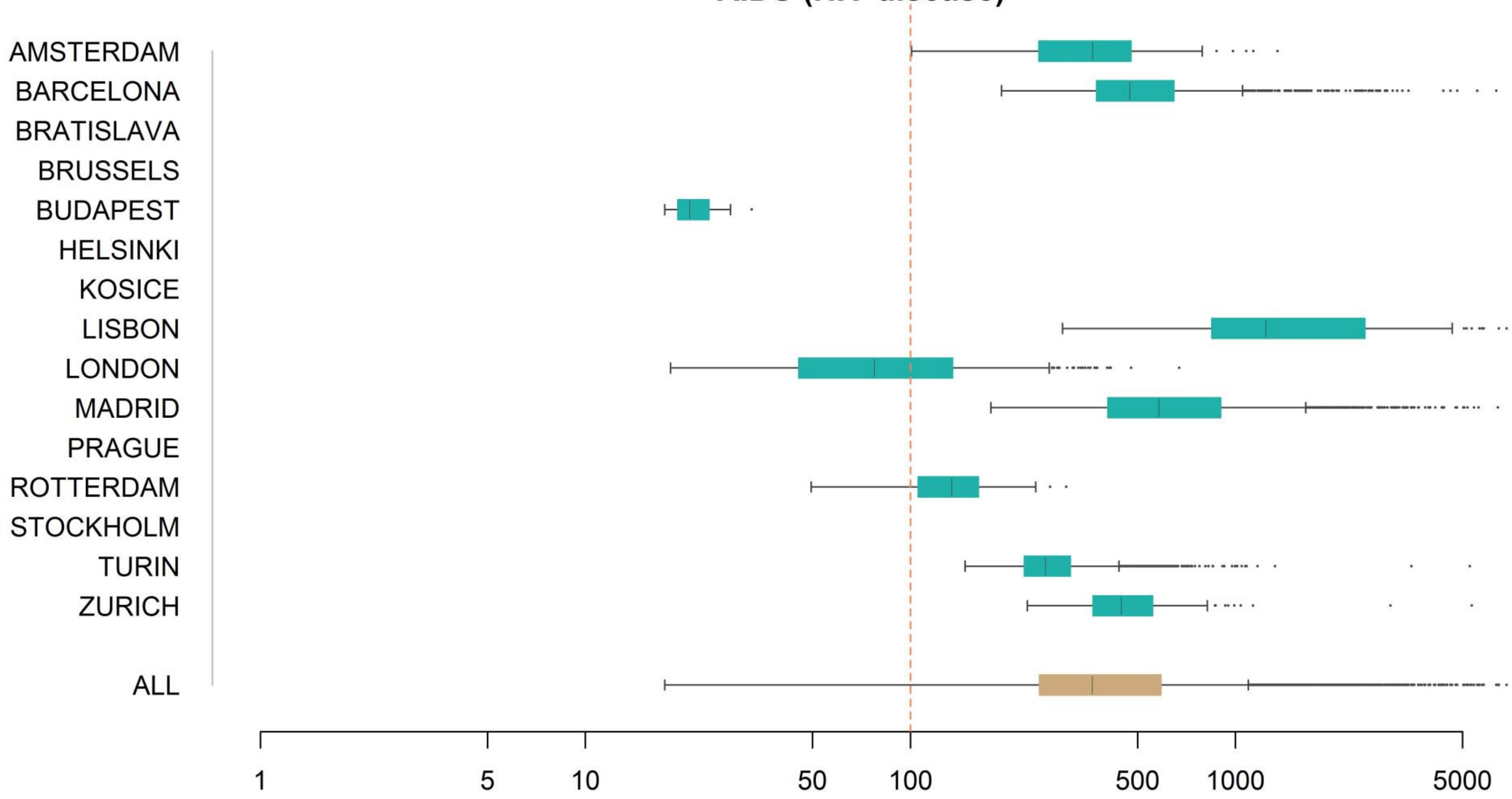

Smoothed Standardised Mortality Ratios (sSMR) with respect to EU, Males

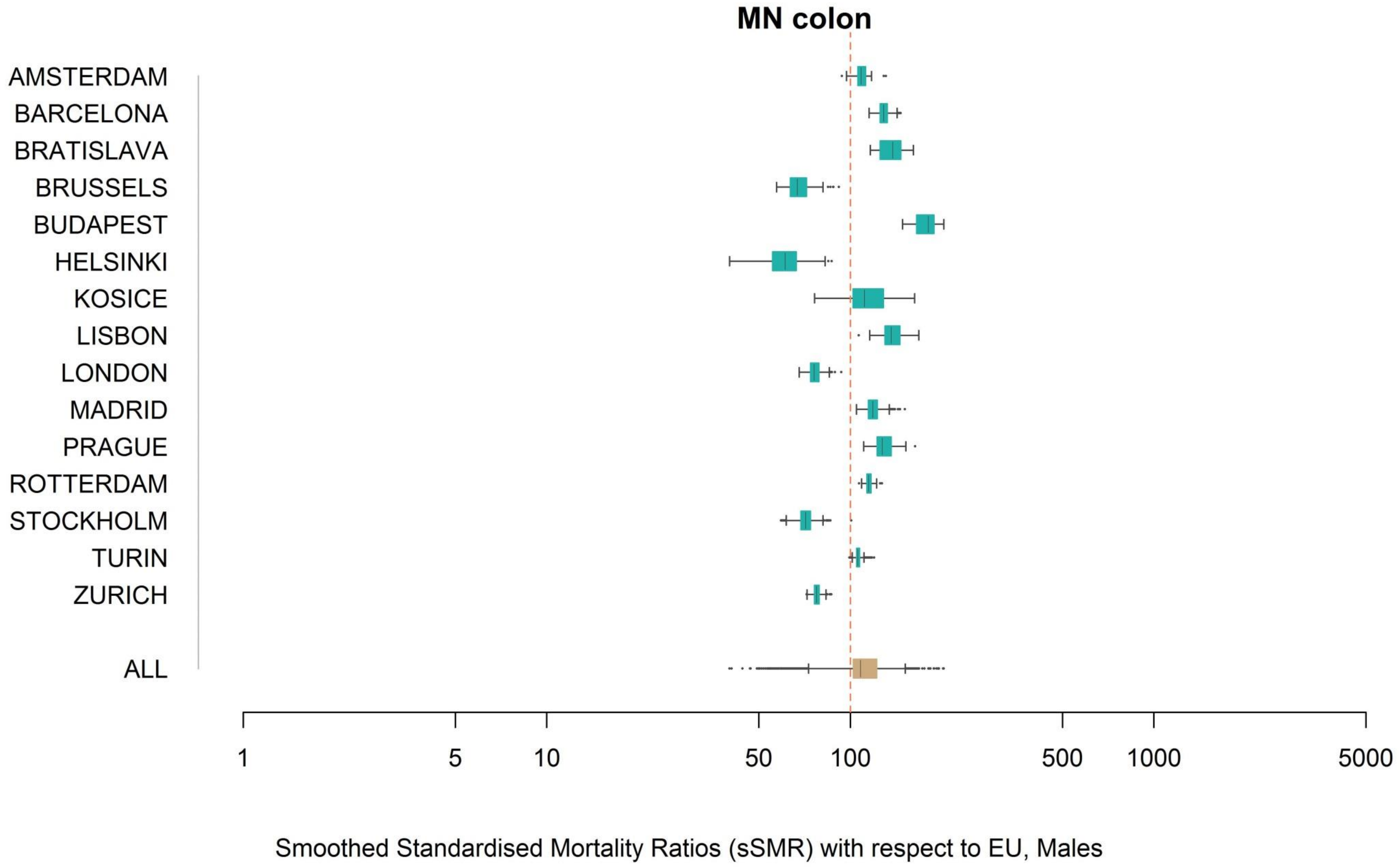

MN colon
AMSTERDAM
BARCELONA
BRATISLAVA
BRUSSELS
BUDAPEST
HELSINKI
KOSICE
LISBON
LONDON
MADRID
PRAGUE
ROTTERDAM
STOCKHOLM
TURIN
ZURICH
ALL
1
5
10
50
100
500
1000
5000
Smoothed Standardised Mortality Ratios (sSMR) with respect to EU, Males

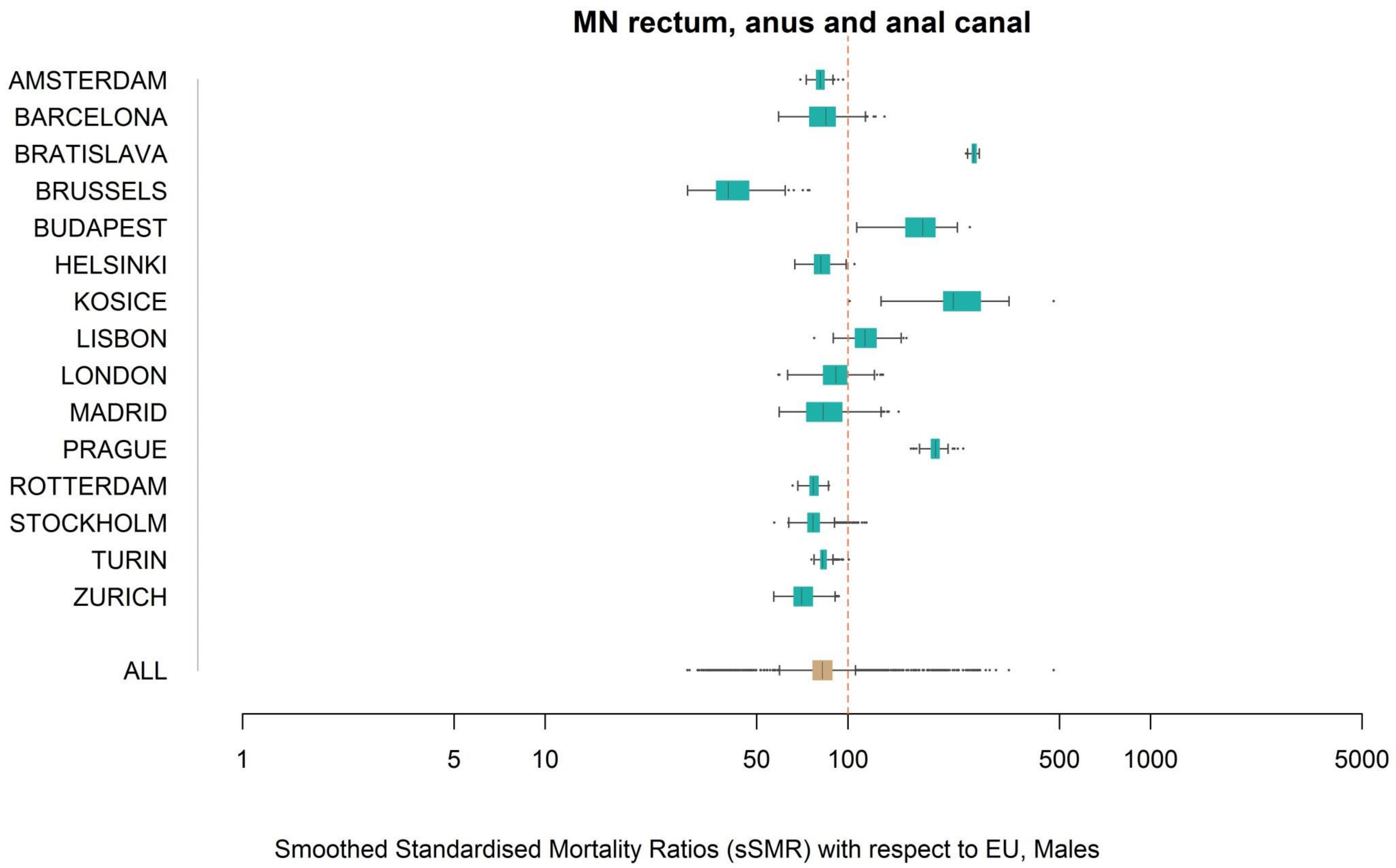

MN rectum, anus and anal canal
AMSTERDAM
BARCELONA
BRATISLAVA
BRUSSELS
BUDAPEST
HELSINKI
KOSICE
LISBON
LONDON
MADRID
PRAGUE
ROTTERDAM
STOCKHOLM
TURIN
ZURICH
ALL
1
5
10
50
100
500
1000
5000
Smoothed Standardised Mortality Ratios (sSMR) with respect to EU, Males

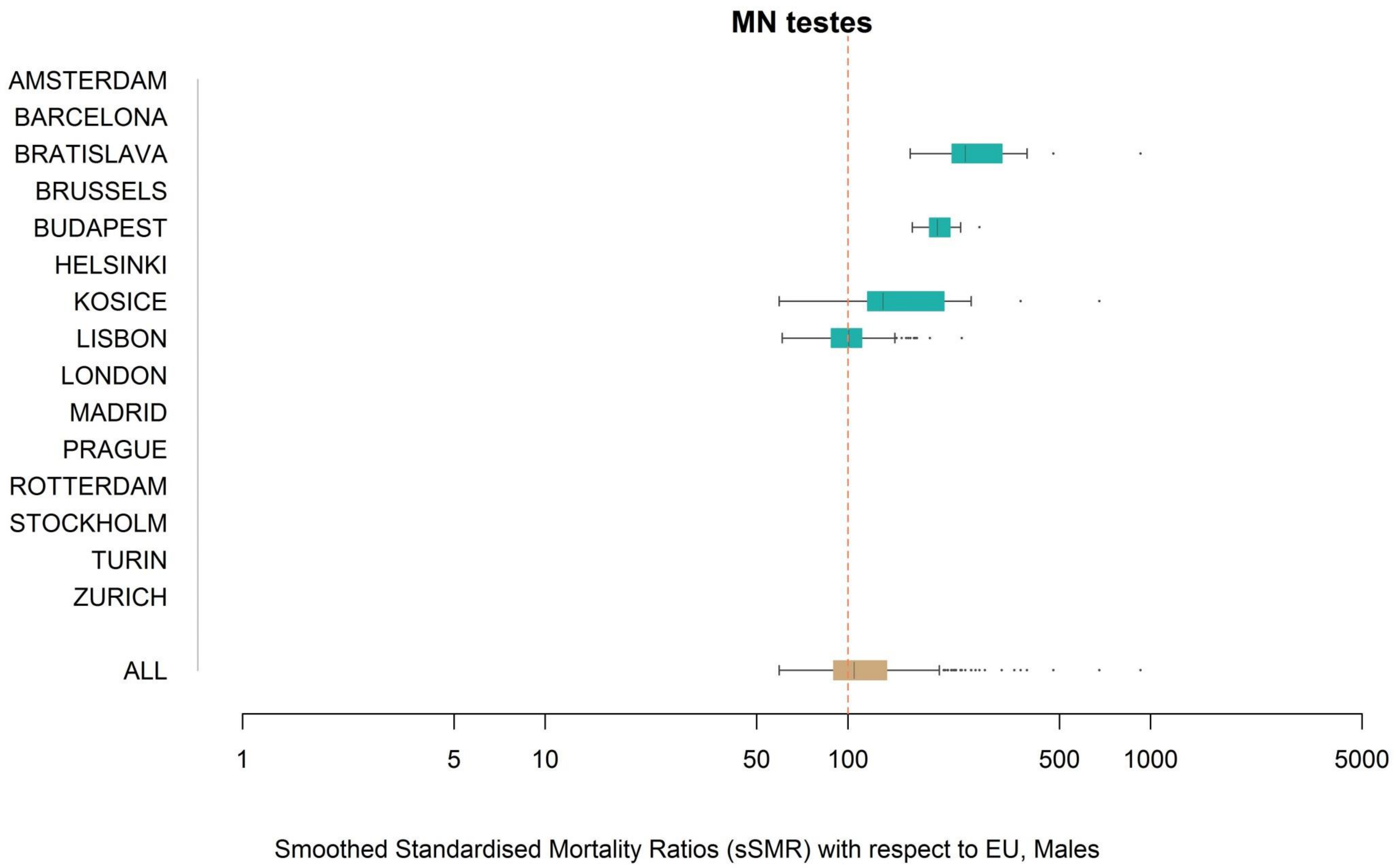

MN testes
AMSTERDAM
BARCELONA
BRATISLAVA
BRUSSELS
BUDAPEST
HELSINKI
KOSICE
LISBON
LONDON
MADRID
PRAGUE
ROTTERDAM
STOCKHOLM
TURIN
ZURICH
ALL
1
5
10
50
100
500
1000
5000
Smoothed Standardised Mortality Ratios (sSMR) with respect to EU, Males

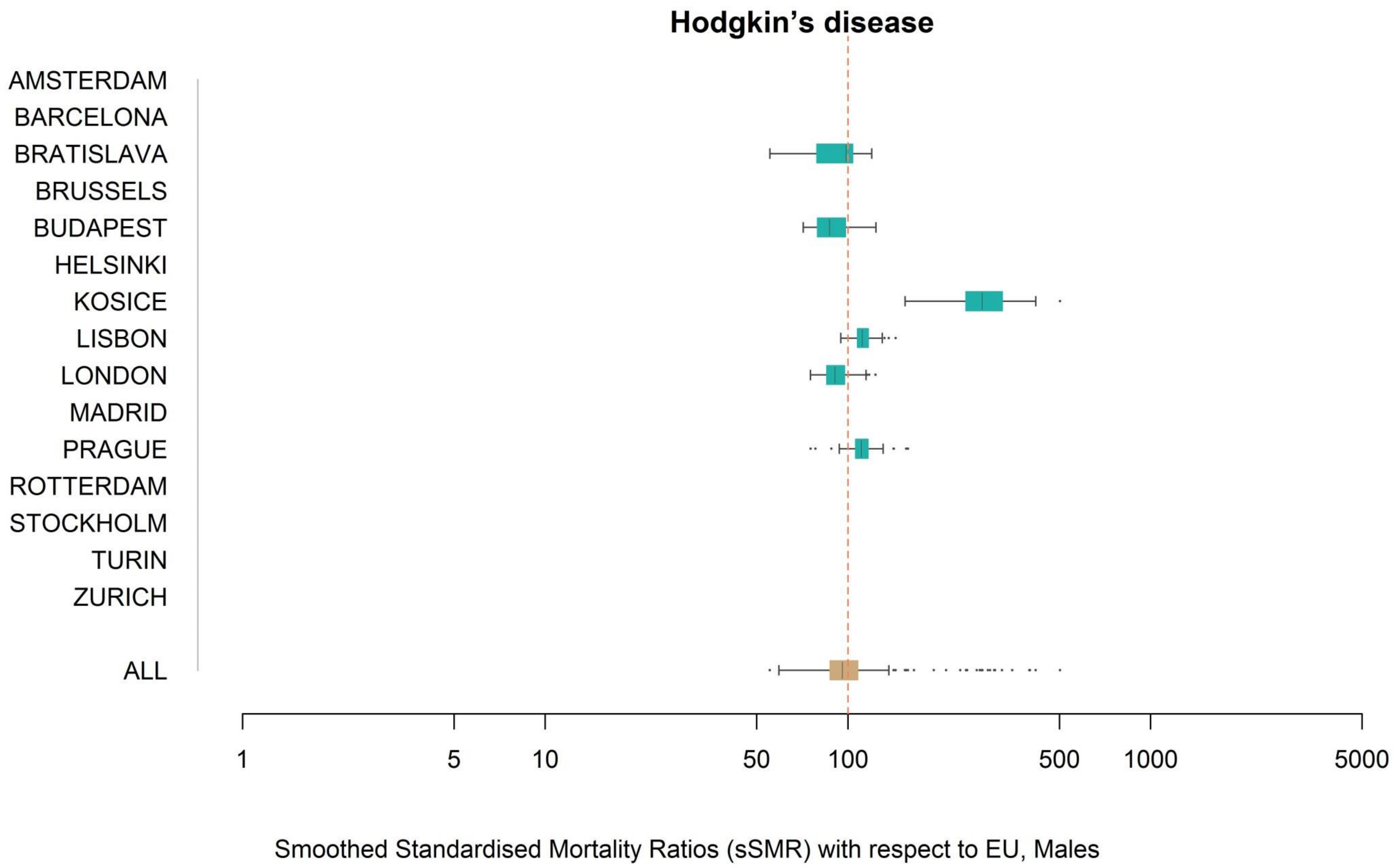
Hodgkin's disease
AMSTERDAM
BARCELONA
BRATISLAVA
BRUSSELS
BUDAPEST
HELSINKI
KOSICE
LISBON
LONDON
MADRID
PRAGUE
ROTTERDAM
STOCKHOLM
TURIN
ZURICH
ALL
1
5
10
50
100
500
1000
5000
Smoothed Standardised Mortality Ratios (sSMR) with respect to EU, Males

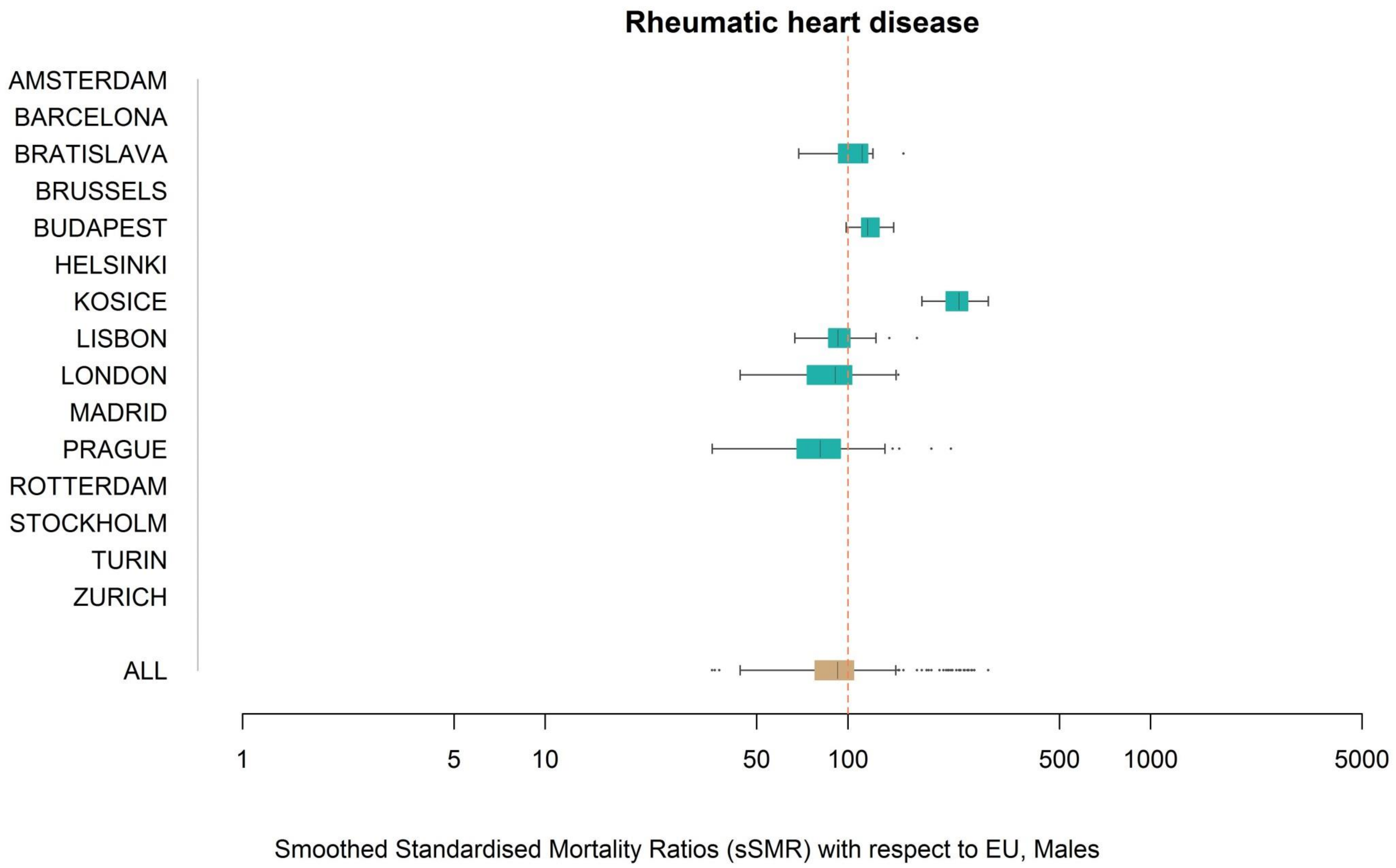
Rheumatic heart disease
AMSTERDAM
BARCELONA
BRATISLAVA
BRUSSELS
BUDAPEST
HELSINKI
KOSICE
LISBON
LONDON
MADRID
PRAGUE
ROTTERDAM
STOCKHOLM
TURIN
ZURICH
ALL
1
5
10
50
100
500
1000
5000
Smoothed Standardised Mortality Ratios (sSMR) with respect to EU, Males

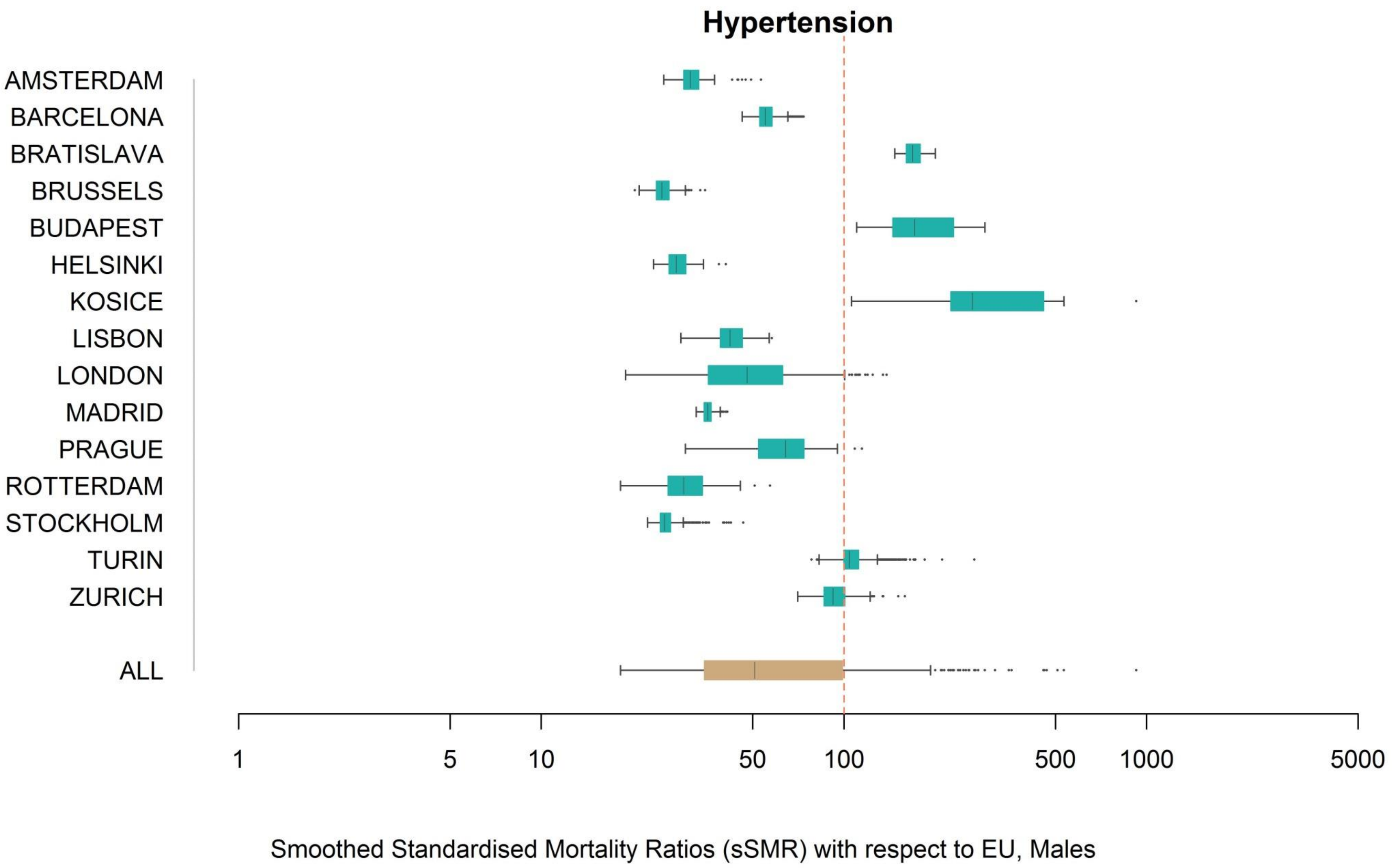

Hypertension
AMSTERDAM
BARCELONA
BRATISLAVA
BRUSSELS
BUDAPEST
HELSINKI
KOSICE
LISBON
LONDON
MADRID
PRAGUE
ROTTERDAM
STOCKHOLM
TURIN
ZURICH
ALL
1
5
10
50
100
500
1000
5000
Smoothed Standardised Mortality Ratios (sSMR) with respect to EU, Males

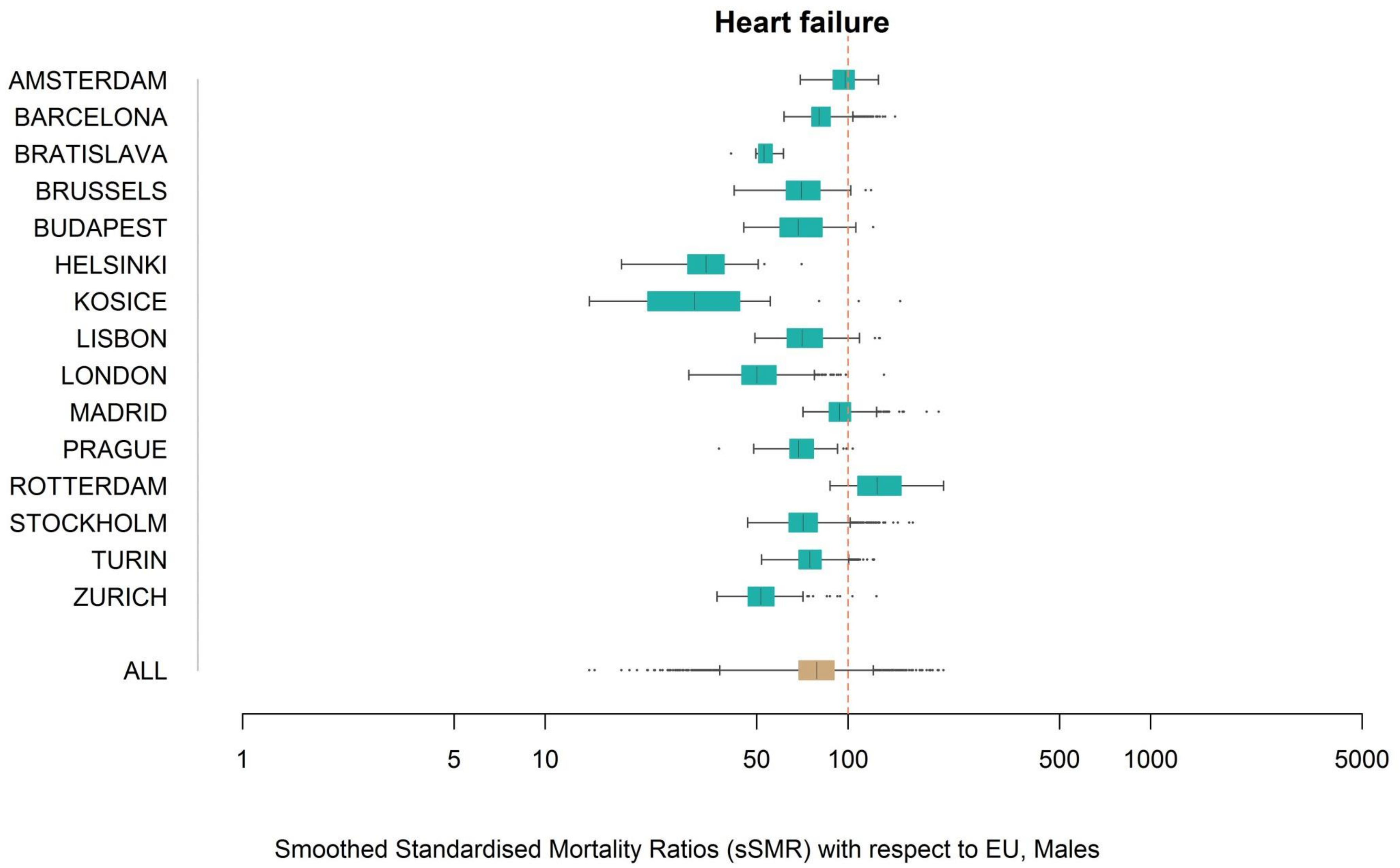

Heart failure
AMSTERDAM
BARCELONA
BRATISLAVA
BRUSSELS
BUDAPEST
HELSINKI
KOSICE
LISBON
LONDON
MADRID
PRAGUE
ROTTERDAM
STOCKHOLM
TURIN
ZURICH
ALL
1
5
10
50
100
500
1000
5000
Smoothed Standardised Mortality Ratios (sSMR) with respect to EU, Males

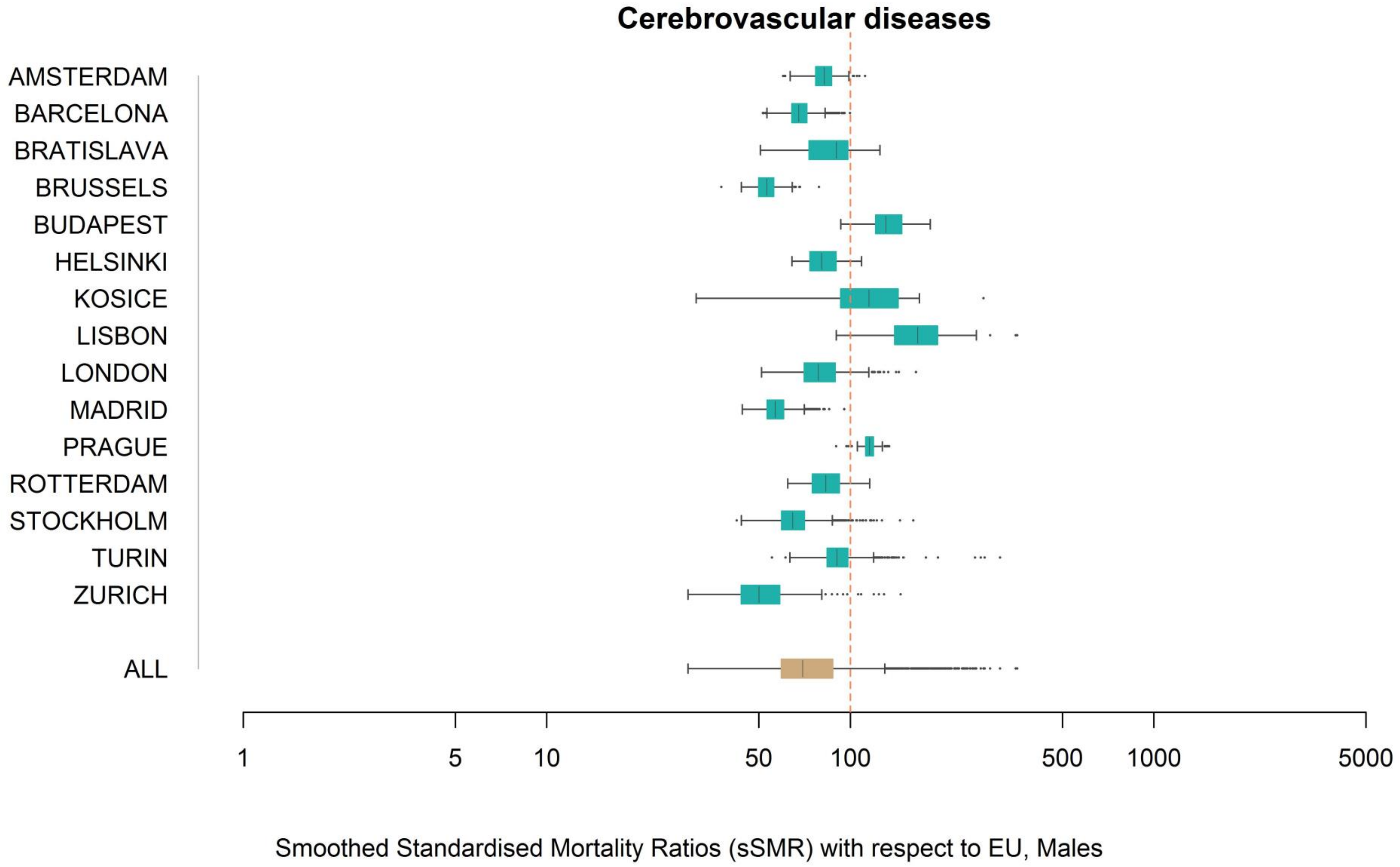
Cerebrovascular diseases
AMSTERDAM
BARCELONA
BRATISLAVA
BRUSSELS
BUDAPEST
HELSINKI
KOSICE
LISBON
LONDON
MADRID
PRAGUE
ROTTERDAM
STOCKHOLM
TURIN
ZURICH
ALL
1
5
10
50
100
500
1000
5000
Smoothed Standardised Mortality Ratios (sSMR) with respect to EU, Males

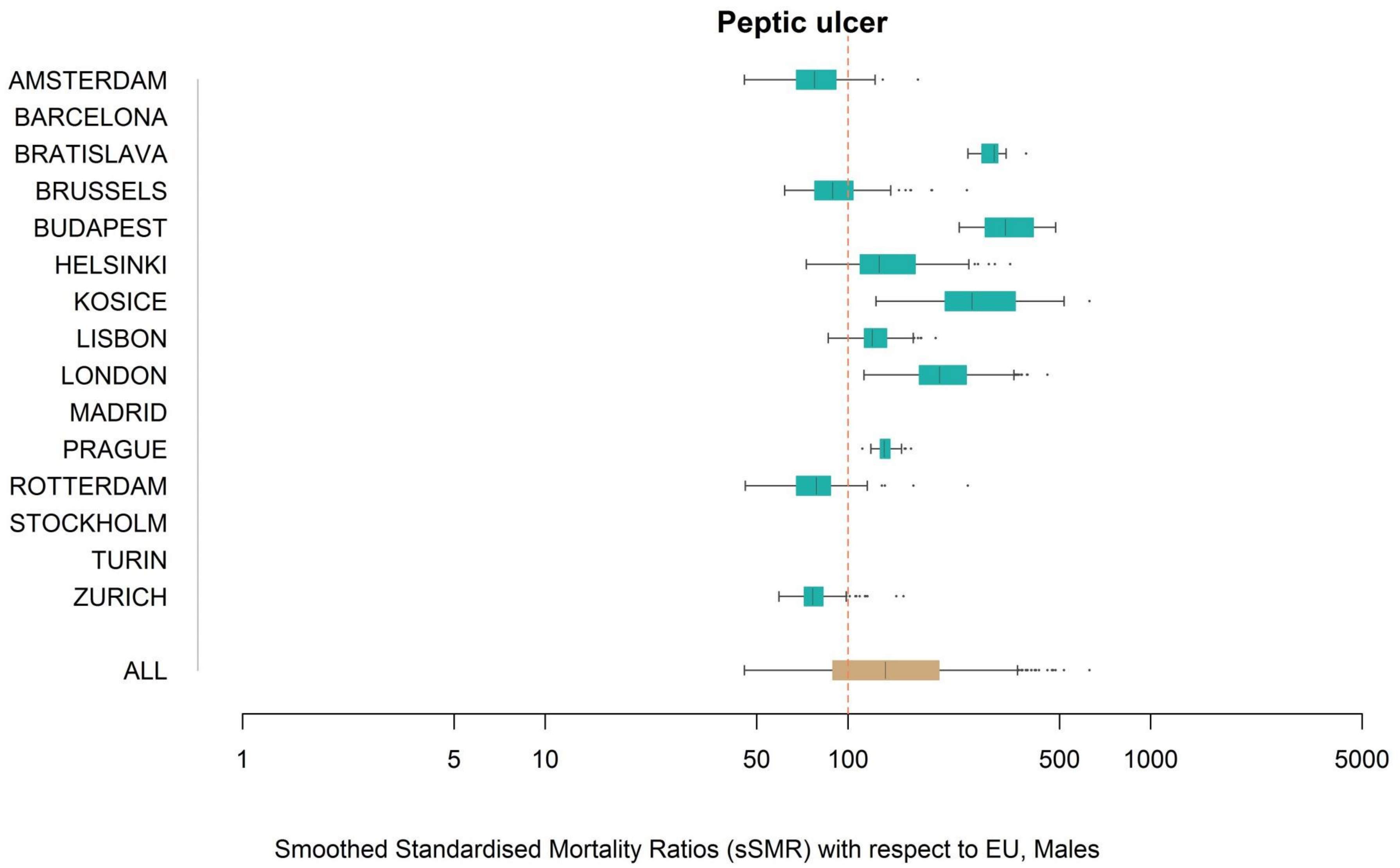
Peptic ulcer
AMSTERDAM
BARCELONA
BRATISLAVA
BRUSSELS
BUDAPEST
HELSINKI
KOSICE
LISBON
LONDON
MADRID
PRAGUE
ROTTERDAM
STOCKHOLM
TURIN
ZURICH
ALL
1
5
10
50
100
500
1000
5000
Smoothed Standardised Mortality Ratios (sSMR) with respect to EU, Males

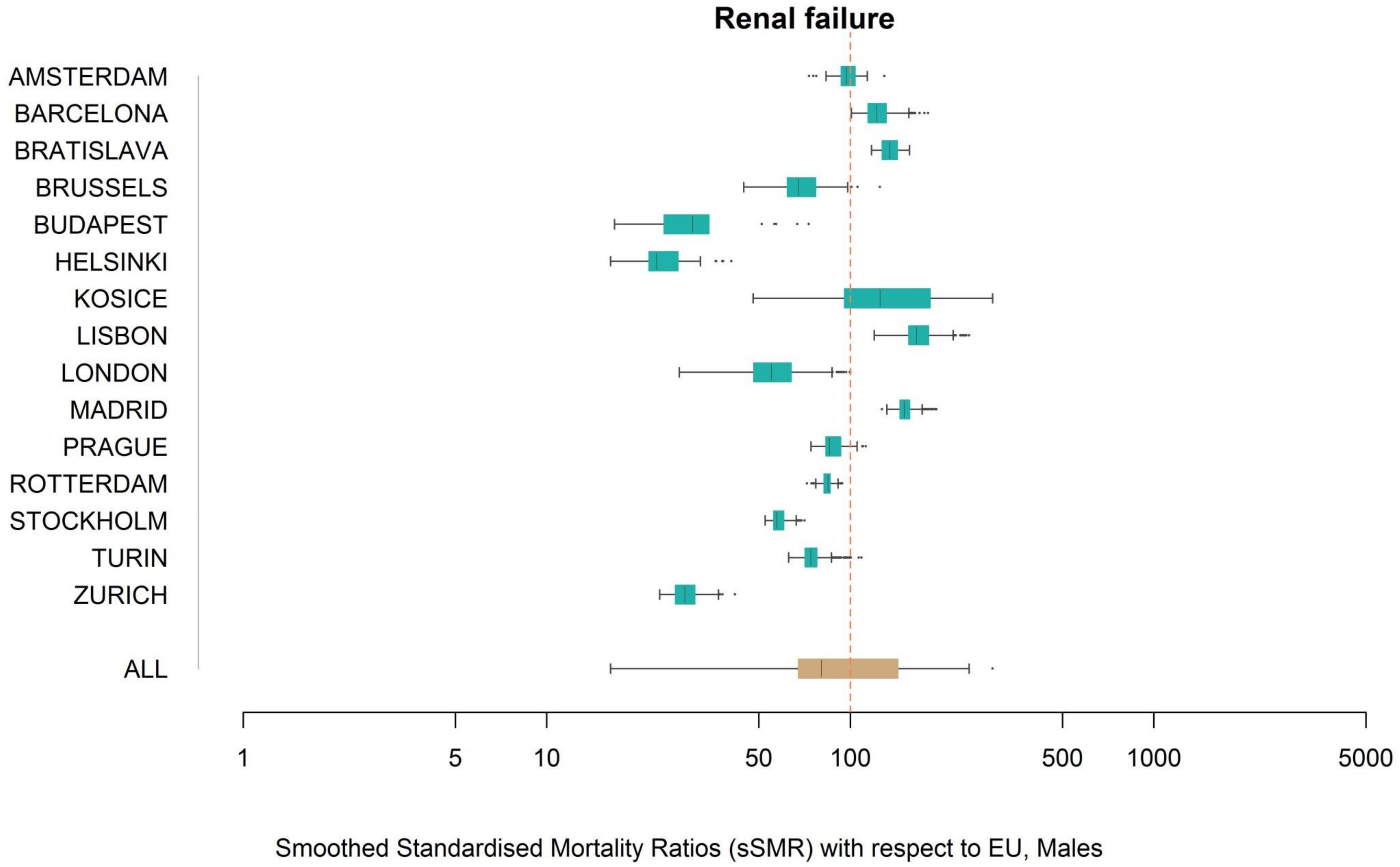
Renal failure
AMSTERDAM
BARCELONA
BRATISLAVA
BRUSSELS
BUDAPEST
HELSINKI
KOSICE
LISBON
LONDON
MADRID
PRAGUE
ROTTERDAM
STOCKHOLM
TURIN
ZURICH
ALL
1
5
10
50
100
500
1000
5000
Smoothed Standardised Mortality Ratios (sSMR) with respect to EU, Males

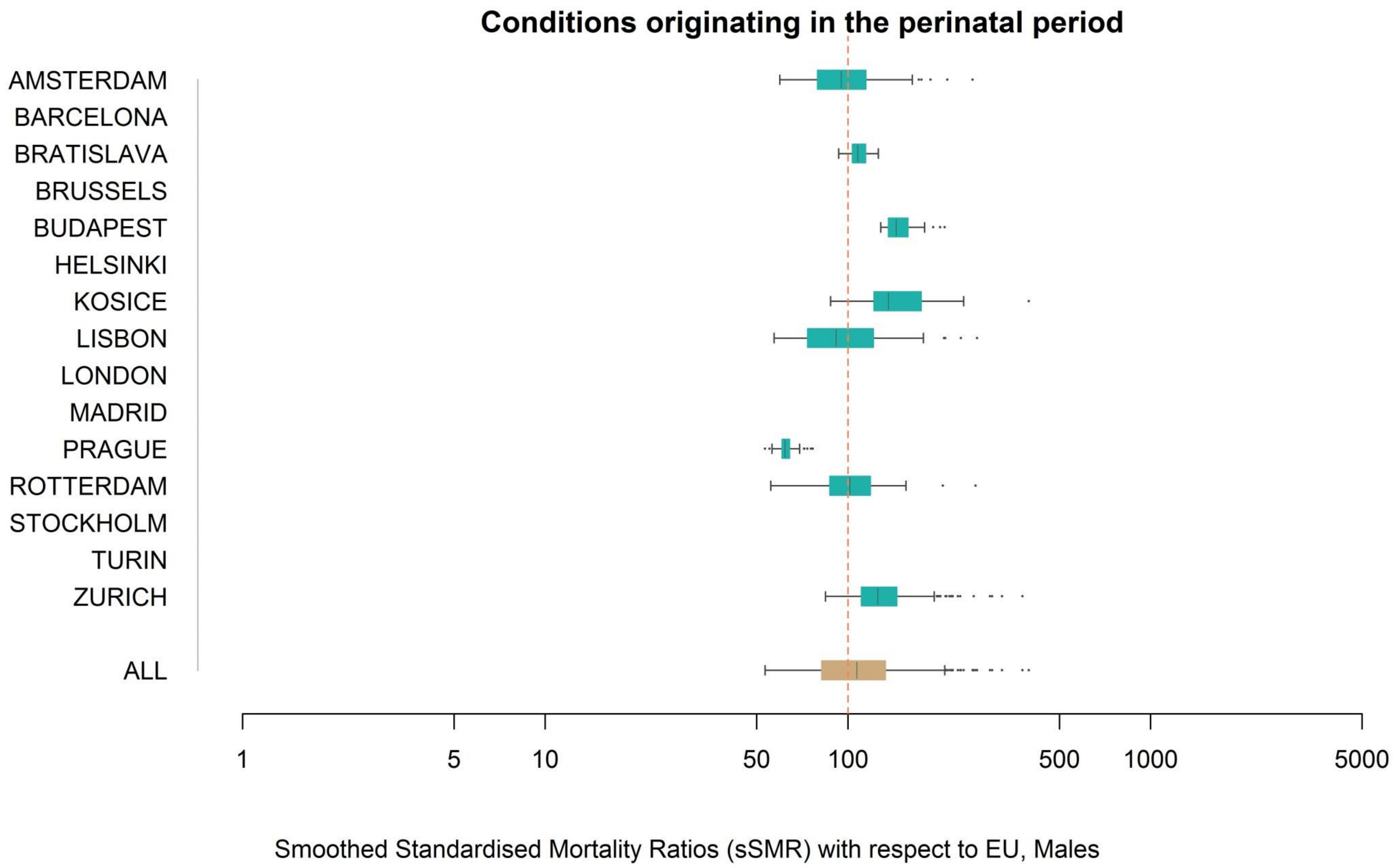
Conditions originating in the perinatal period
AMSTERDAM
BARCELONA
BRATISLAVA
BRUSSELS
BUDAPEST
HELSINKI
KOSICE
LISBON
LONDON
MADRID
PRAGUE
ROTTERDAM
STOCKHOLM
TURIN
ZURICH
ALL
1
5
10
50
100
500
1000
5000
Smoothed Standardised Mortality Ratios (sSMR) with respect to EU, Males

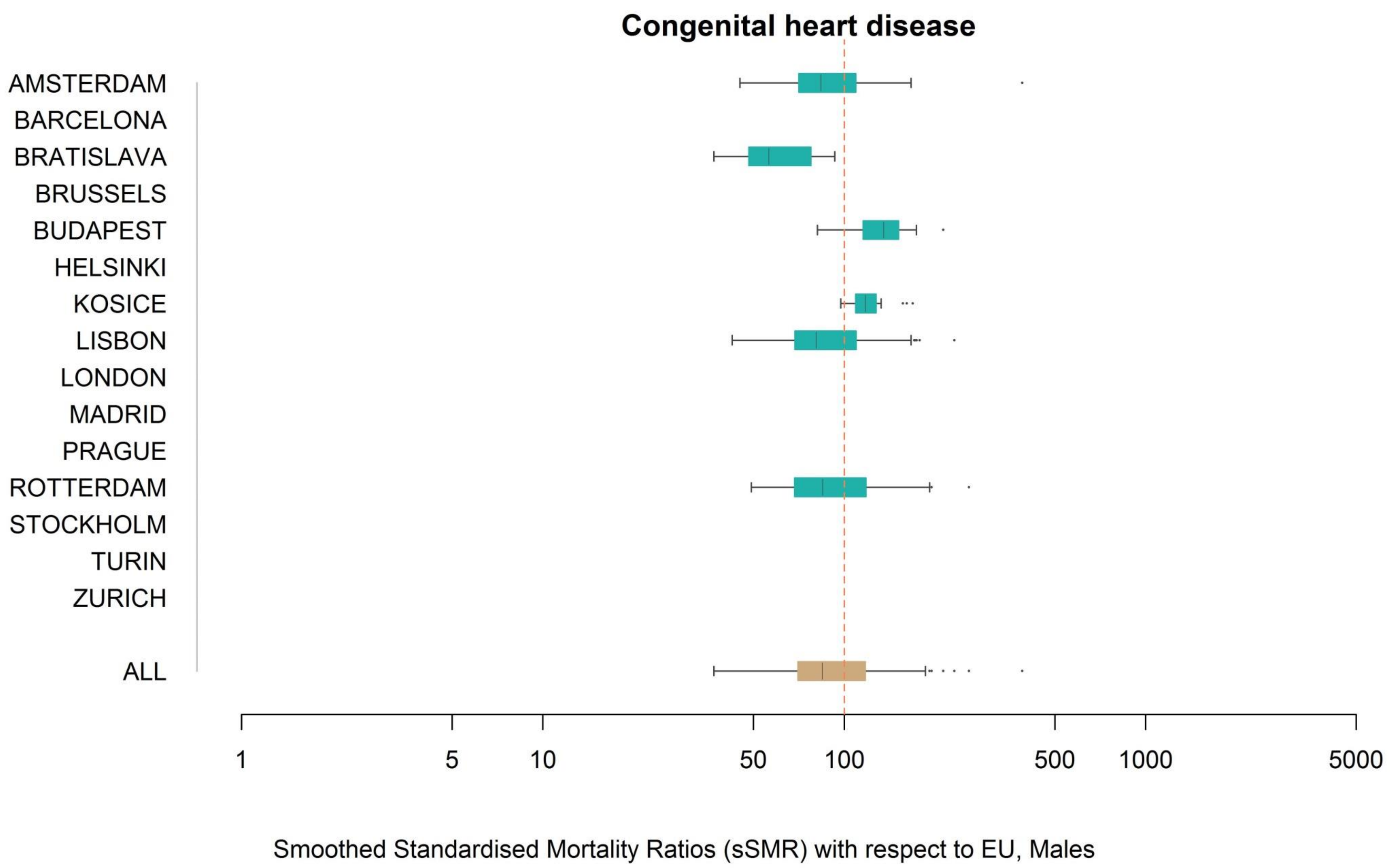

Congenital heart disease
AMSTERDAM
BARCELONA
BRATISLAVA
BRUSSELS
BUDAPEST
HELSINKI
KOSICE
LISBON
LONDON
MADRID
PRAGUE
ROTTERDAM
STOCKHOLM
TURIN
ZURICH
ALL
1
5
10
50
100
500
1000
5000
Smoothed Standardised Mortality Ratios (sSMR) with respect to EU, Males

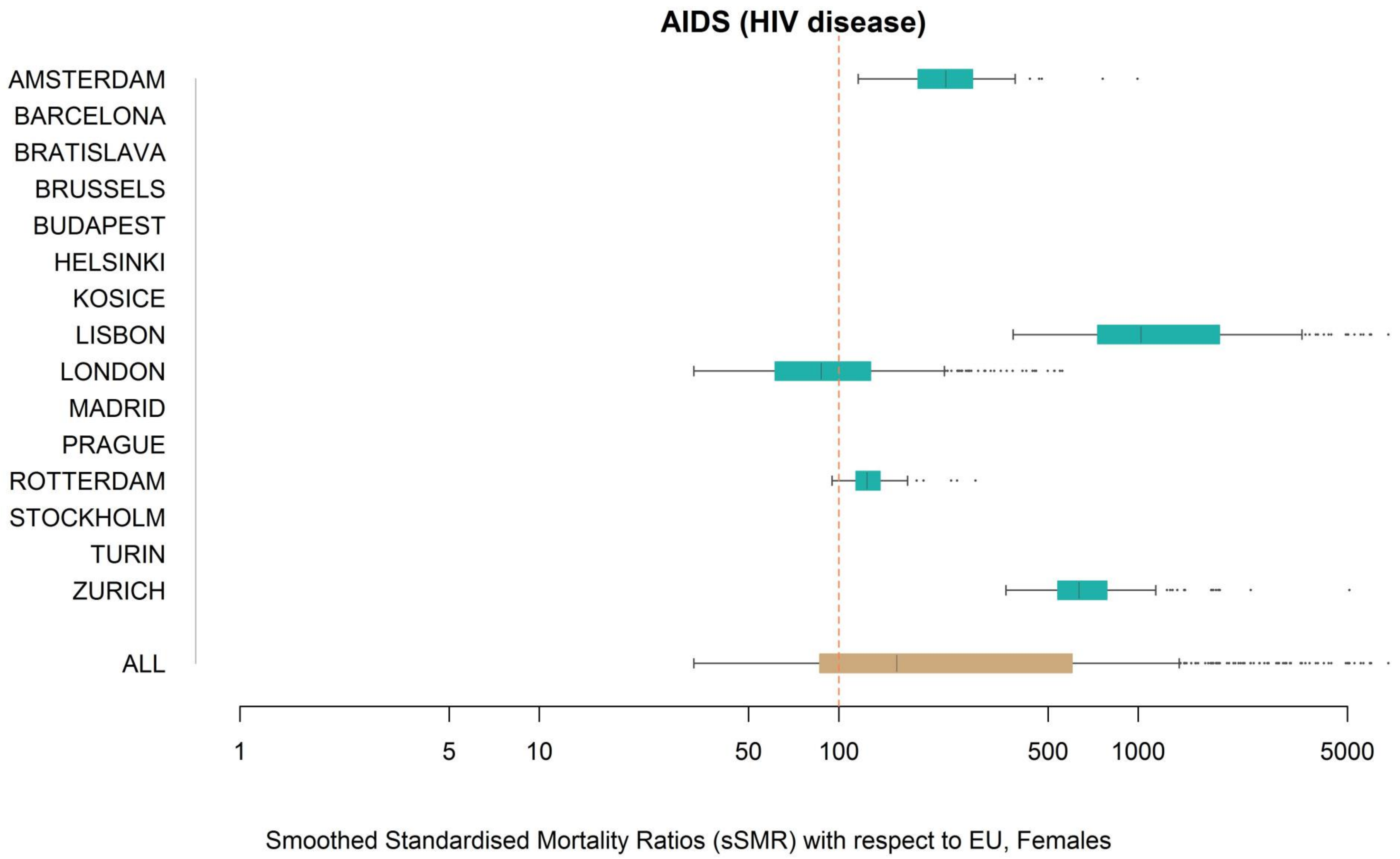
AIDS (HIV disease)
AMSTERDAM
BARCELONA
BRATISLAVA
BRUSSELS
BUDAPEST
HELSINKI
KOSICE
LISBON
LONDON
MADRID
PRAGUE
ROTTERDAM
STOCKHOLM
TURIN
ZURICH
ALL
1
5
10
50
100
500
1000
5000
Smoothed Standardised Mortality Ratios (sSMR) with respect to EU, Females

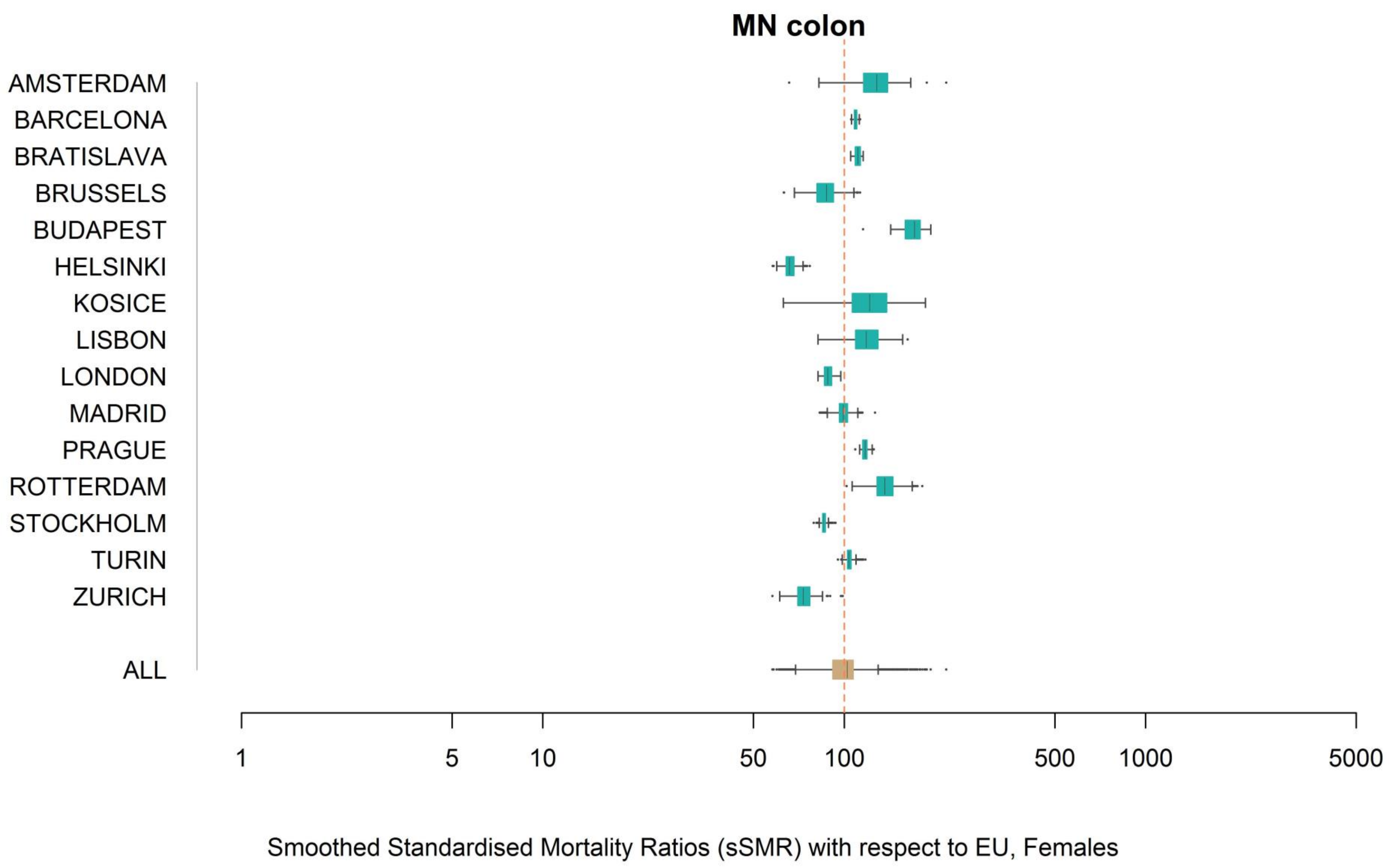
MN colon
AMSTERDAM
BARCELONA
BRATISLAVA
BRUSSELS
BUDAPEST
HELSINKI
KOSICE
LISBON
LONDON
MADRID
PRAGUE
ROTTERDAM
STOCKHOLM
TURIN
ZURICH
ALL
1
5
10
50
100
500
1000
5000
Smoothed Standardised Mortality Ratios (sSMR) with respect to EU, Females

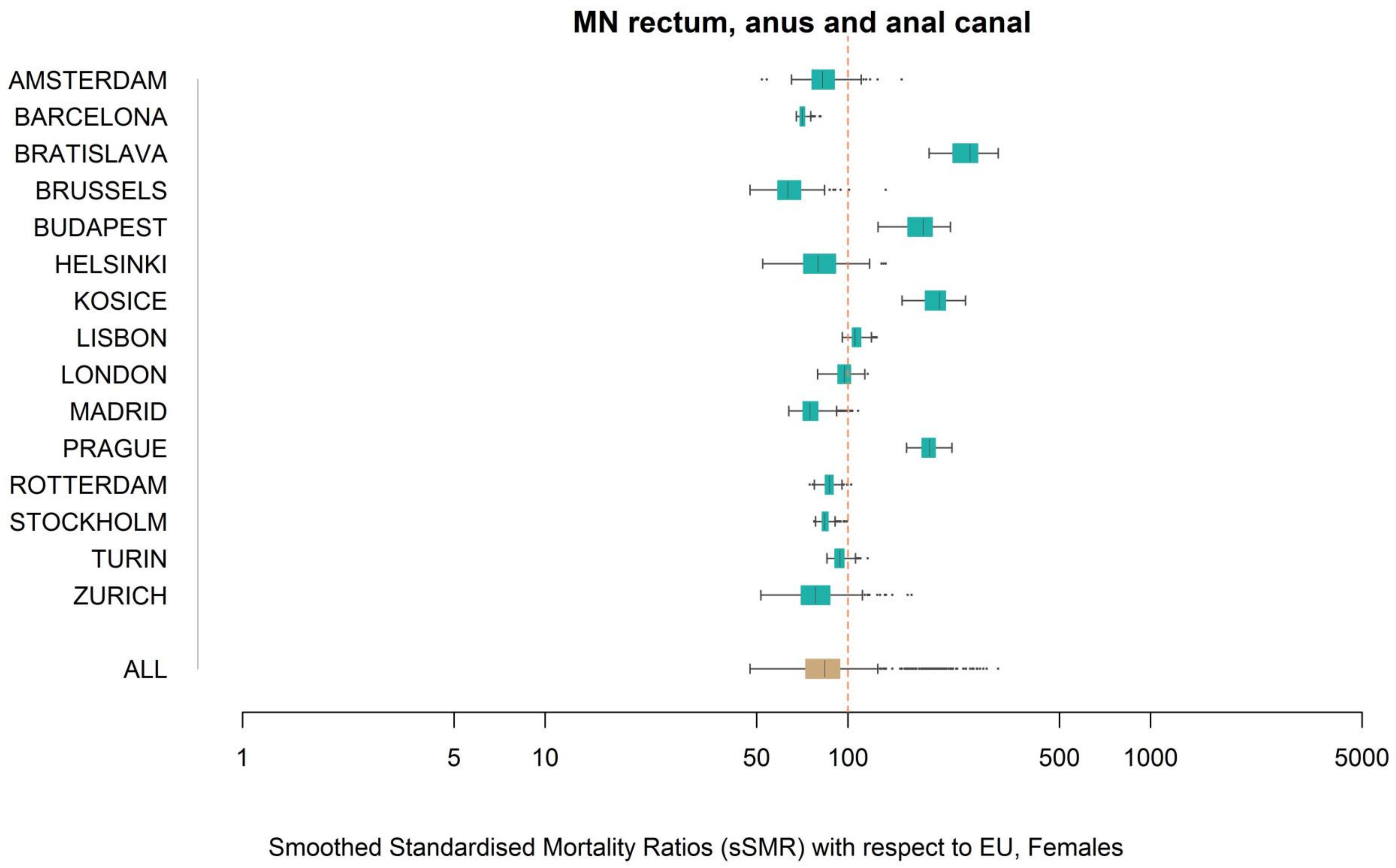
MN rectum, anus and anal canal
AMSTERDAM
BARCELONA
BRATISLAVA
BRUSSELS
BUDAPEST
HELSINKI
KOSICE
LISBON
LONDON
MADRID
PRAGUE
ROTTERDAM
STOCKHOLM
TURIN
ZURICH
ALL
1
5
10
50
100
500
1000
5000
Smoothed Standardised Mortality Ratios (sSMR) with respect to EU, Females

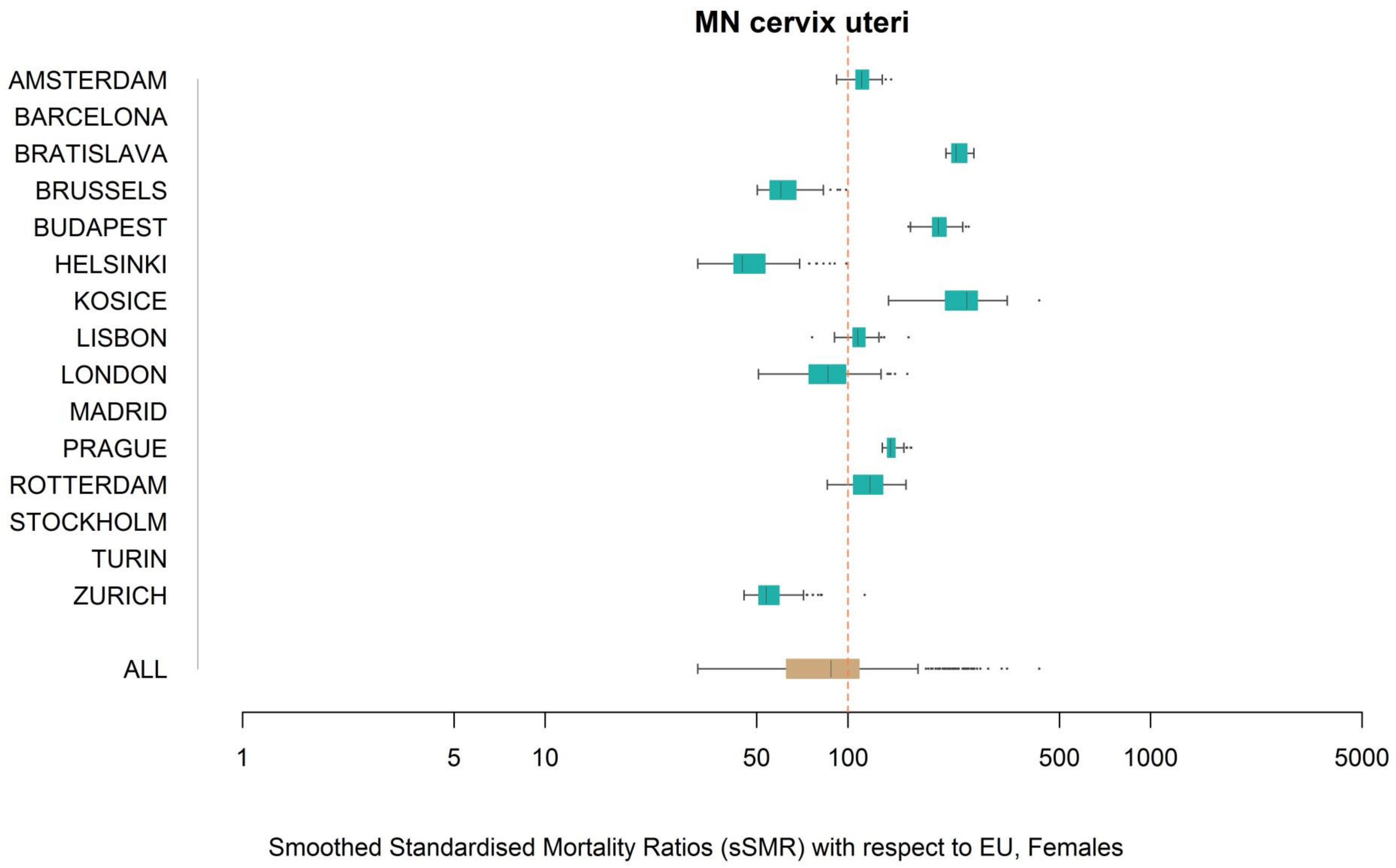
MN cervix uteri
AMSTERDAM
BARCELONA
BRATISLAVA
BRUSSELS
BUDAPEST
HELSINKI
KOSICE
LISBON
LONDON
MADRID
PRAGUE
ROTTERDAM
STOCKHOLM
TURIN
ZURICH
ALL
1
5
10
50
100
500
1000
5000
Smoothed Standardised Mortality Ratios (sSMR) with respect to EU, Females

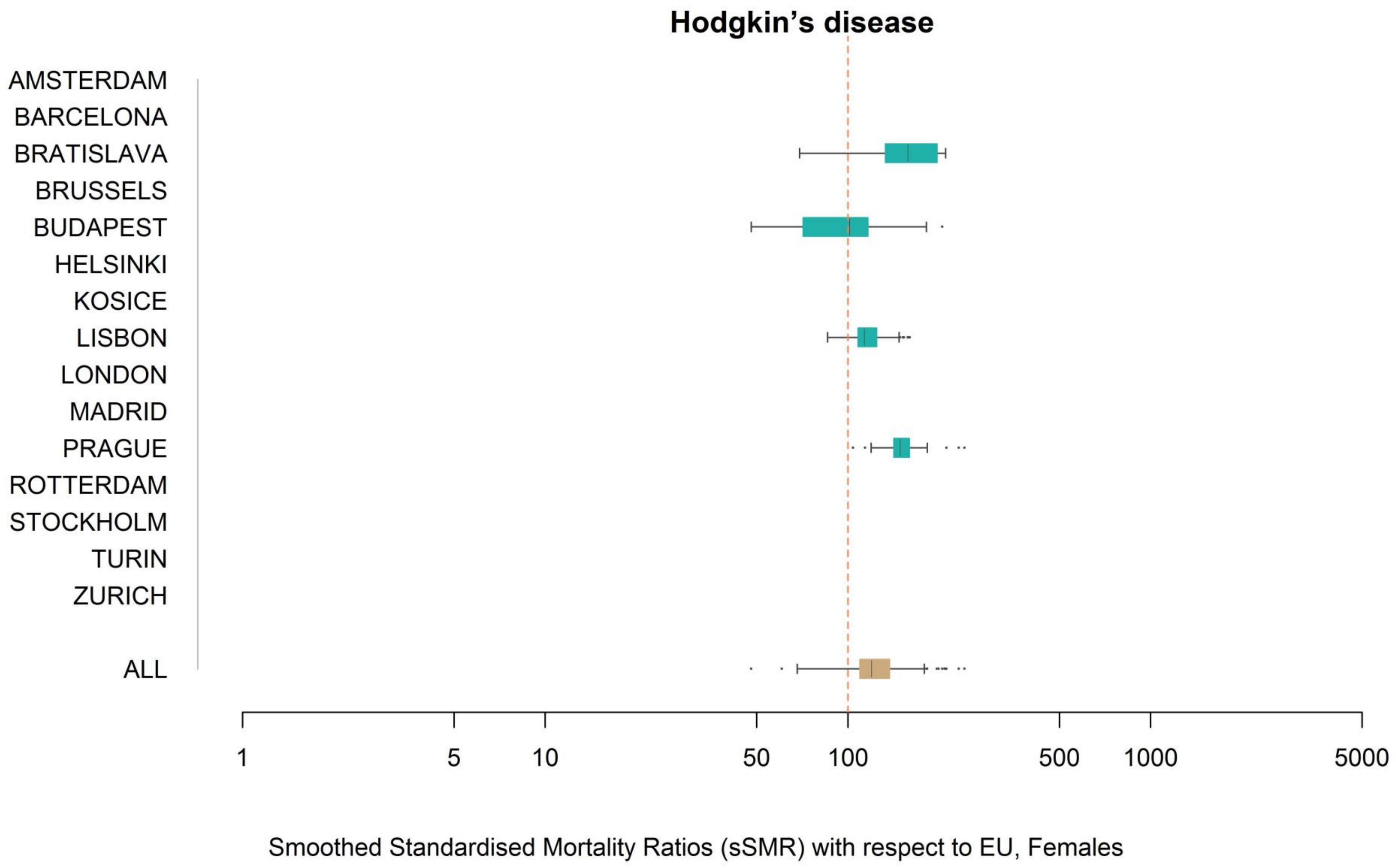
Hodgkin's disease
AMSTERDAM
BARCELONA
BRATISLAVA
BRUSSELS
BUDAPEST
HELSINKI
KOSICE
LISBON
LONDON
MADRID
PRAGUE
ROTTERDAM
STOCKHOLM
TURIN
ZURICH
ALL
1
5
10
50
100
500
1000
5000
Smoothed Standardised Mortality Ratios (sSMR) with respect to EU, Females

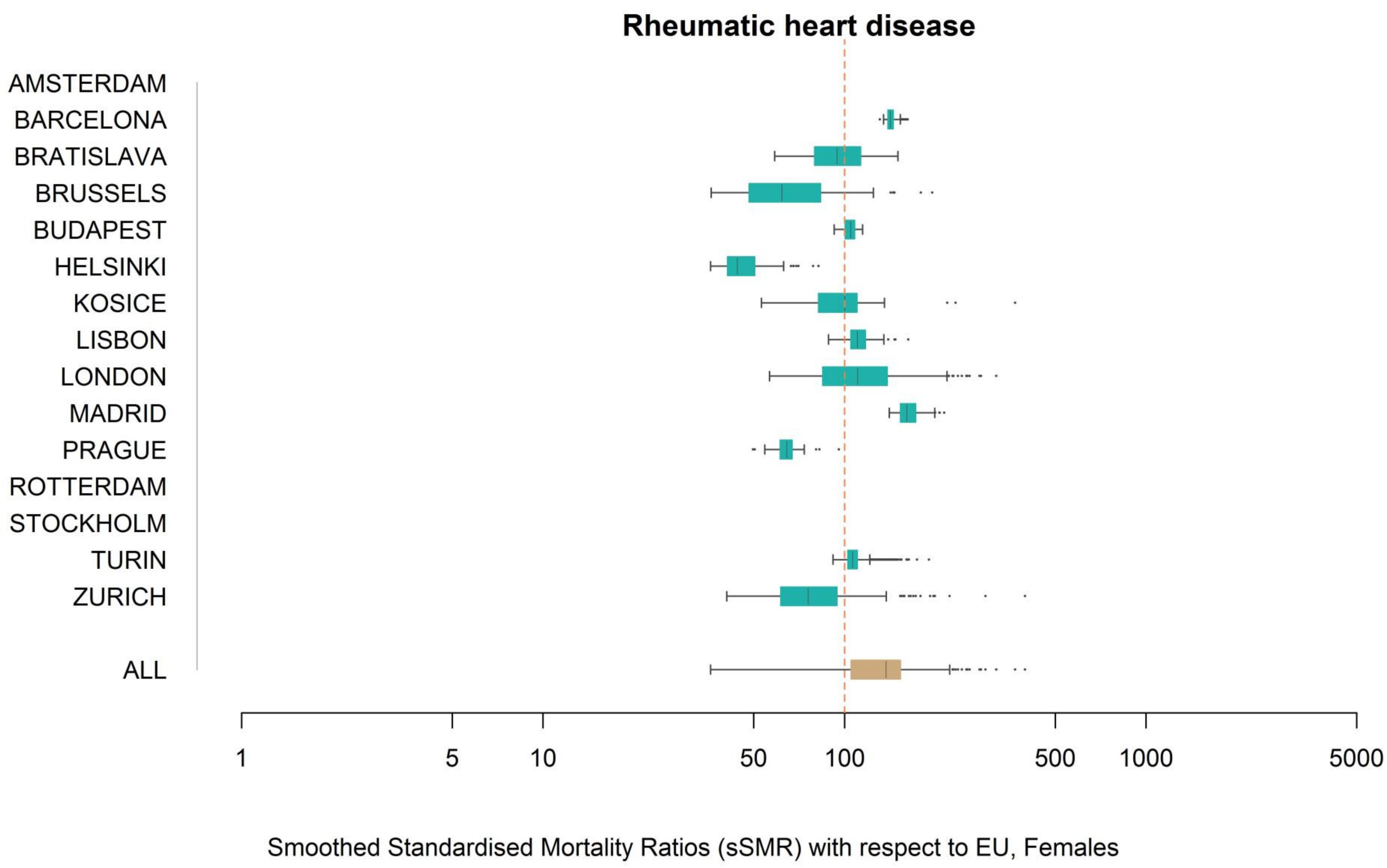
Rheumatic heart disease
AMSTERDAM
BARCELONA
BRATISLAVA
BRUSSELS
BUDAPEST
HELSINKI
KOSICE
LISBON
LONDON
MADRID
PRAGUE
ROTTERDAM
STOCKHOLM
TURIN
ZURICH
ALL
1
5
10
50
100
500
1000
5000
Smoothed Standardised Mortality Ratios (sSMR) with respect to EU, Females

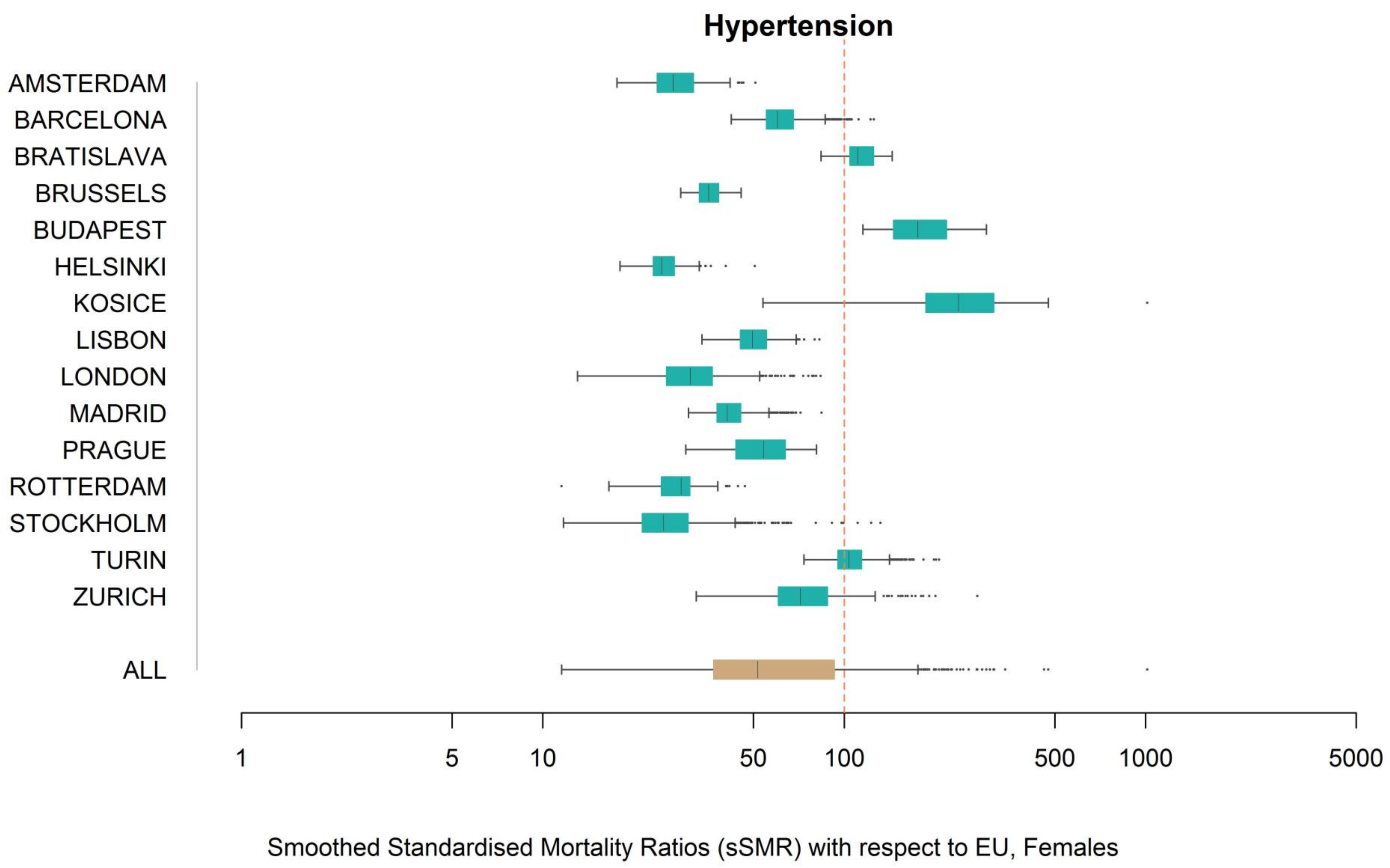
Hypertension
AMSTERDAM
BARCELONA
BRATISLAVA
BRUSSELS
BUDAPEST
HELSINKI
KOSICE
LISBON
LONDON
MADRID
PRAGUE
ROTTERDAM
STOCKHOLM
TURIN
ZURICH
ALL
1
5
10
50
100
500
1000
5000
Smoothed Standardised Mortality Ratios (sSMR) with respect to EU, Females

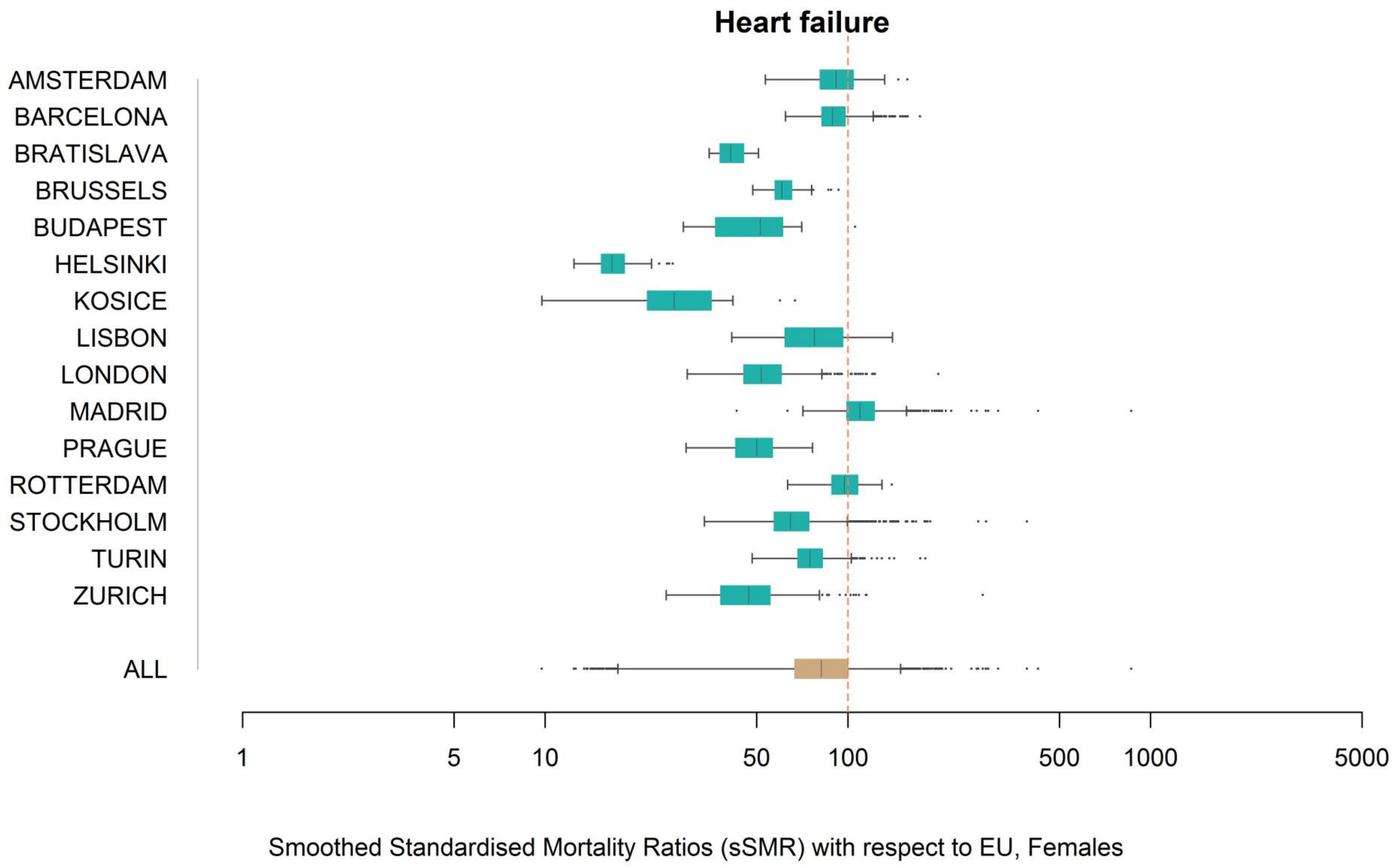
Heart failure
AMSTERDAM
BARCELONA
BRATISLAVA
BRUSSELS
BUDAPEST
HELSINKI
KOSICE
LISBON
LONDON
MADRID
PRAGUE
ROTTERDAM
STOCKHOLM
TURIN
ZURICH
ALL
1
5
10
50
100
500
1000
5000
Smoothed Standardised Mortality Ratios (sSMR) with respect to EU, Females

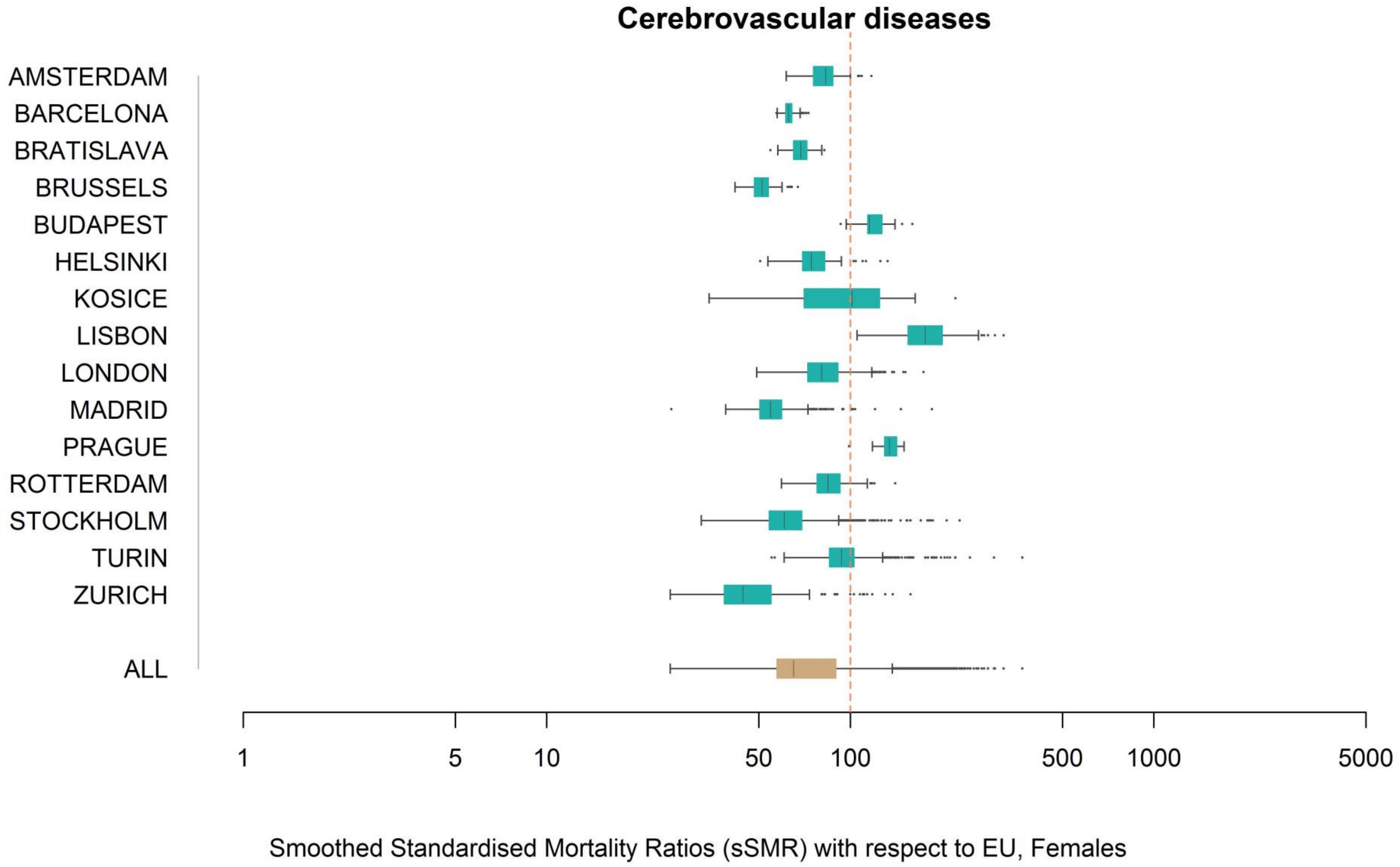
Cerebrovascular diseases
AMSTERDAM
BARCELONA
BRATISLAVA
BRUSSELS
BUDAPEST
HELSINKI
KOSICE
LISBON
LONDON
MADRID
PRAGUE
ROTTERDAM
STOCKHOLM
TURIN
ZURICH
ALL
1
5
10
50
100
500
1000
5000
Smoothed Standardised Mortality Ratios (sSMR) with respect to EU, Females

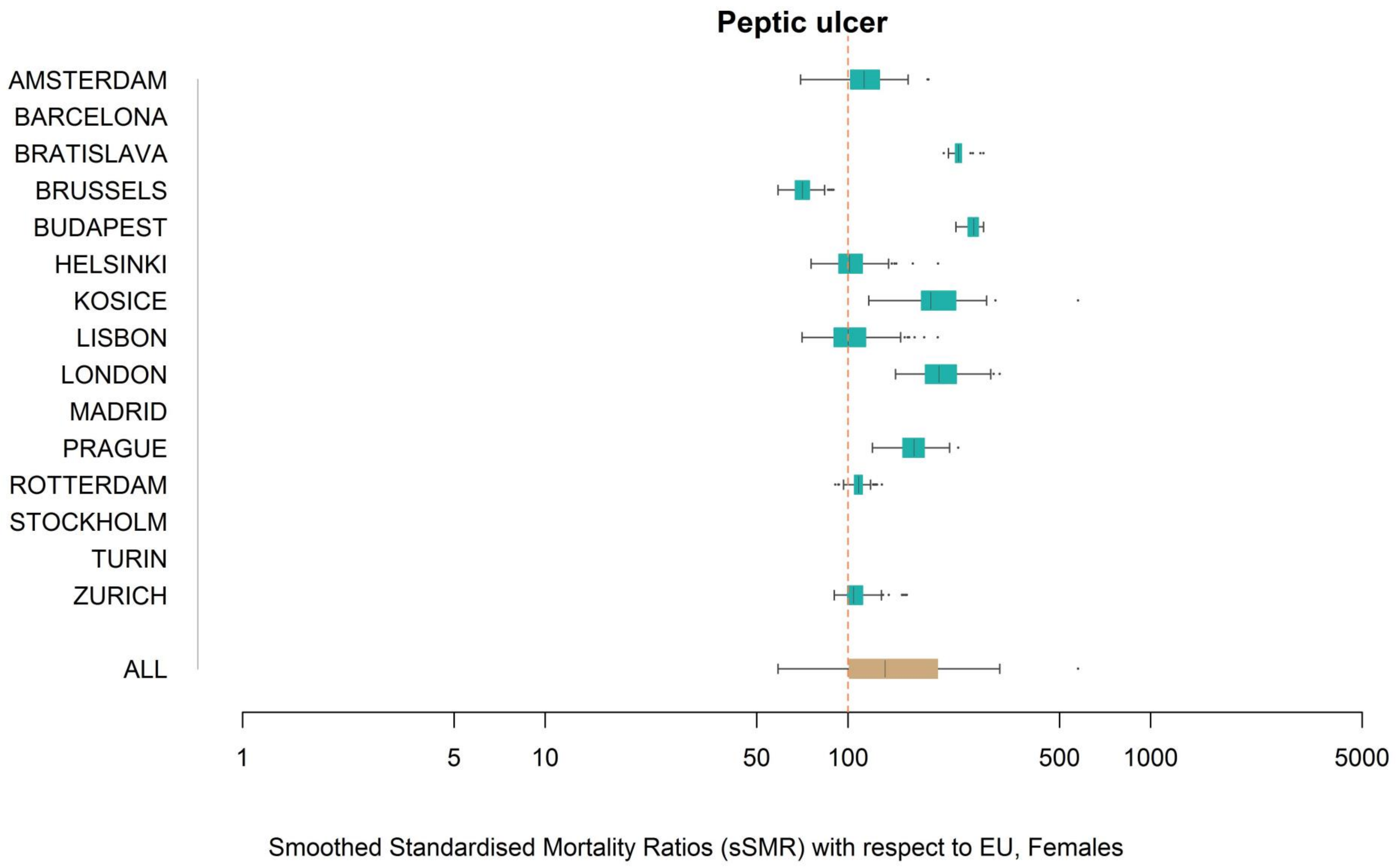
Peptic ulcer
AMSTERDAM
BARCELONA
BRATISLAVA
BRUSSELS
BUDAPEST
HELSINKI
KOSICE
LISBON
LONDON
MADRID
PRAGUE
ROTTERDAM
STOCKHOLM
TURIN
ZURICH
ALL
1
5
10
50
100
500
1000
5000
Smoothed Standardised Mortality Ratios (sSMR) with respect to EU, Females

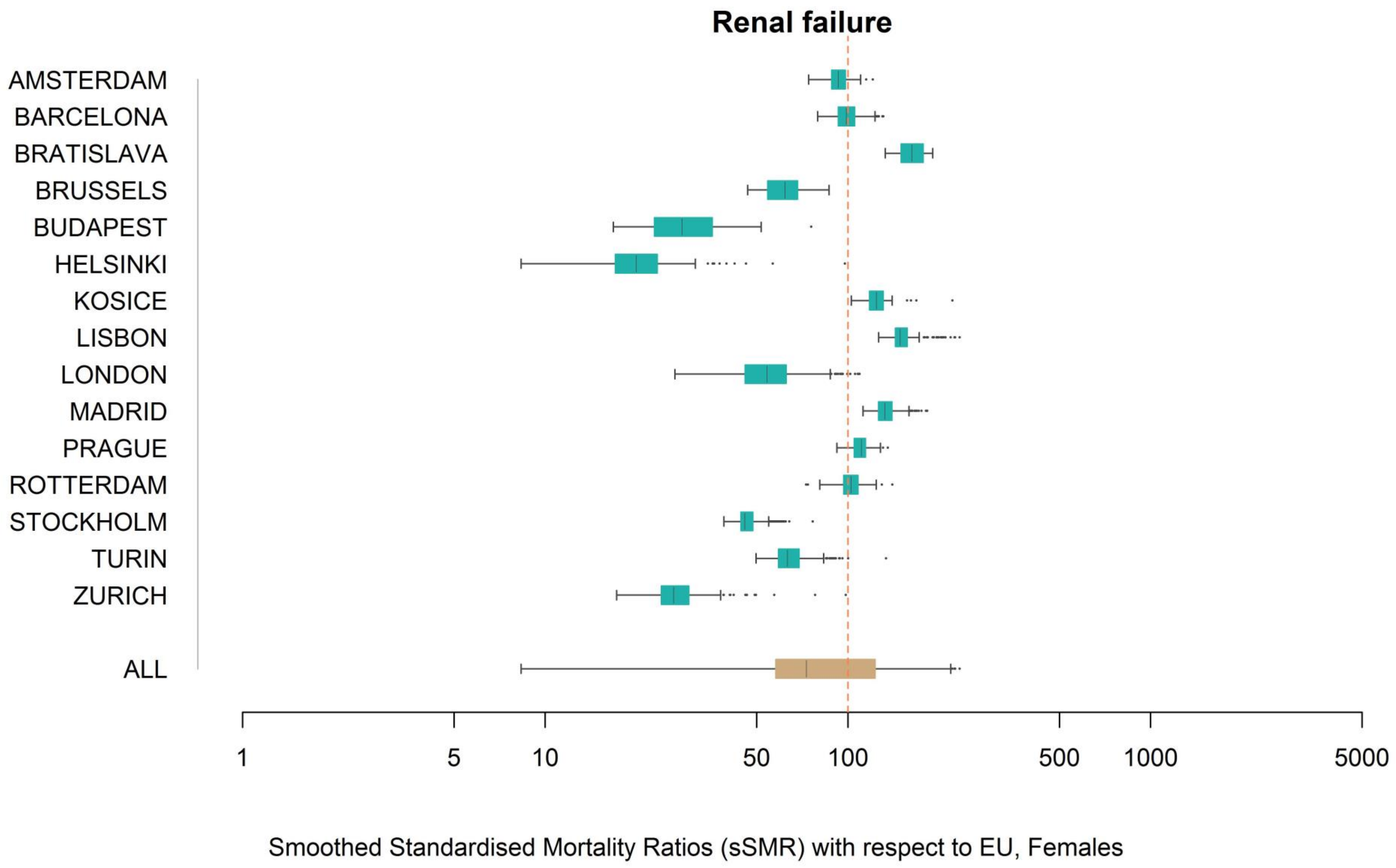

Renal failure
AMSTERDAM
BARCELONA
BRATISLAVA
BRUSSELS
BUDAPEST
HELSINKI
KOSICE
LISBON
LONDON
MADRID
PRAGUE
ROTTERDAM
STOCKHOLM
TURIN
ZURICH
ALL
1
5
10
50
100
500
1000
5000
Smoothed Standardised Mortality Ratios (sSMR) with respect to EU, Females

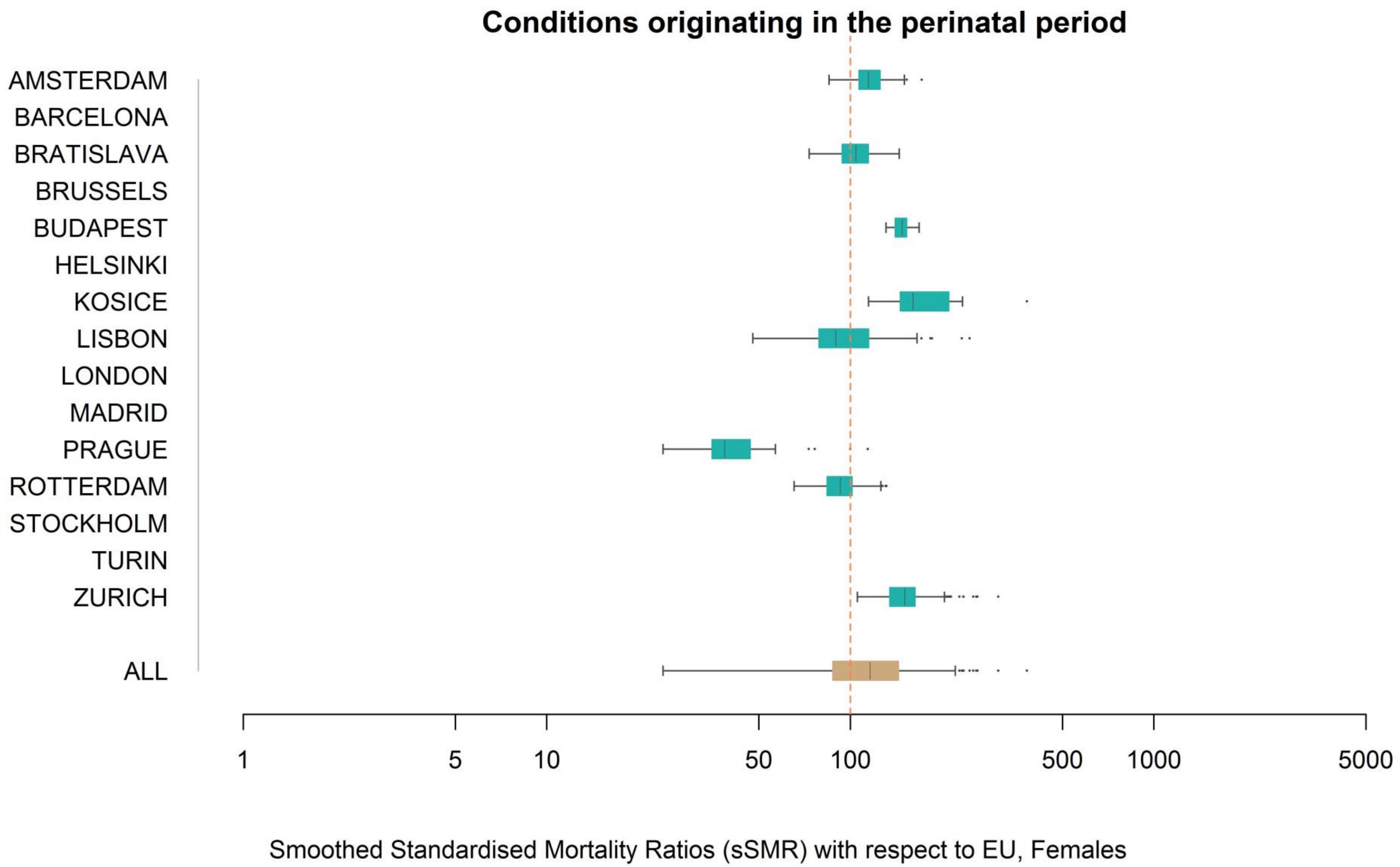

Conditions originating in the perinatal period
AMSTERDAM
BARCELONA
BRATISLAVA
BRUSSELS
BUDAPEST
HELSINKI
KOSICE
LISBON
LONDON
MADRID
PRAGUE
ROTTERDAM
STOCKHOLM
TURIN
ZURICH
ALL
1
5
10
50
100
500
1000
5000
Smoothed Standardised Mortality Ratios (sSMR) with respect to EU, Females

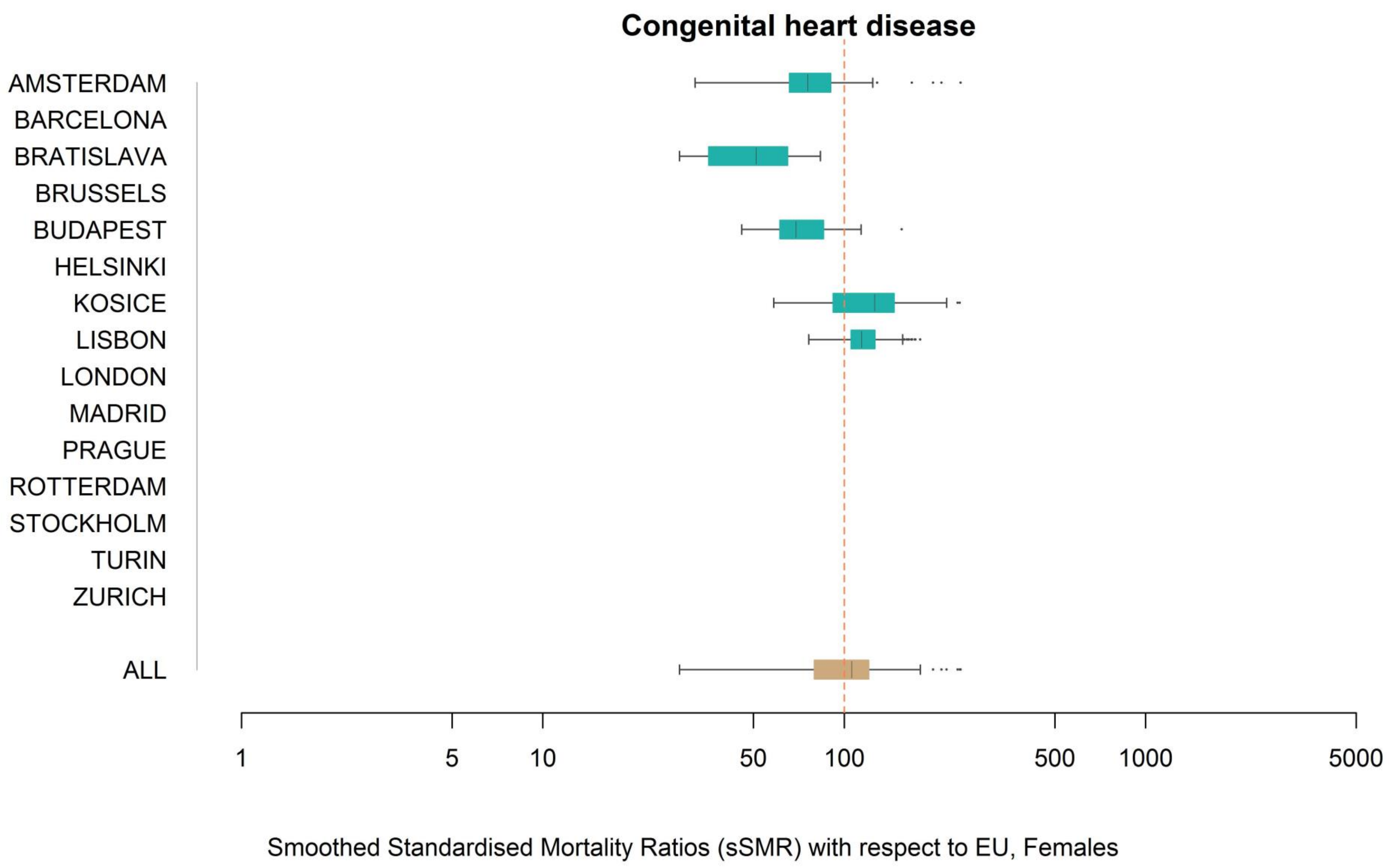
Congenital heart disease
AMSTERDAM
BARCELONA
BRATISLAVA
BRUSSELS
BUDAPEST
HELSINKI
KOSICE
LISBON
LONDON
MADRID
PRAGUE
ROTTERDAM
STOCKHOLM
TURIN
ZURICH
ALL
1
5
10
50
100
500
1000
5000
Smoothed Standardised Mortality Ratios (sSMR) with respect to EU, Females
