# Supplementary material for: Social differences in avoidable mortality between small areas of 15 European cities: an ecological study
Source: Int J Health Geogr. 2014 Mar 12;13:8. doi: 10.1186/1476-072X-13-8 (PMC4007807; doi:10.1186/1476-072X-13-8)
Supplement: Additional file 19 — Cause-specific mortality rate ratios (Table). [file 1476-072X-13-8-S19.pdf]

# Appendix Table 2: Mortality rate ratios between most deprived and least deprived quartile, by city, cause of death and gender[a]

| Cause of Death | AIDS (HIV disease) | | MN colon | | MN rectum, anus, anal canal | | MN cervix uteri | MN testes | Hodgkin's disease | | Rheumatic heart disease | | Hypertension | | Heart failure | | Cerebro-vascular diseases | | Peptic ulcer | | Renal failure | | Cond. origin. in perinatal period | | Congenital heart disease | |
|---|---|---|---|---|---|---|---|---|---|---|---|---|---|---|---|---|---|---|---|---|---|---|---|---|---|---|
| Gender / City | m | f | m | f | m | f | f | m | m | f | m | f | m | f | m | f | m | f | m | f | m | f | m | f | m | f |
| Amsterdam | 1.09 | **4.95** | **0.70** | 0.90 | **0.65** | 1.18 | 0.99 | [b] | 0.42 | [b] | [b] | [b] | 1.06 | 0.93 | 0.89 | 1.26 | 0.91 | 1.26 | 2.07 | 1.73 | 1.15 | 0.98 | **6.24** | **2.49** | 2.30 | 2.55 |
| Barcelona | **2.86** | **2.70** | 0.93 | 0.91 | **1.28** | 1.02 | 1.34 | 1.38 | 1.62 | **2.14** | 0.98 | 1.12 | 0.93 | 0.99 | 1.02 | **0.82** | 1.01 | 0.82 | 1.31 | 1.54 | 0.97 | 1.02 | 0.98 | **2.02** | 0.77 | 1.26 |
| Bratislava | [b] | [b] | 0.69 | 0.76 | 1.45 | 1.05 | 0.71 | 5.42 | [b] | 2.42 | 1.45 | 0.70 | 0.97 | 1.16 | 1.27 | 1.82 | **10.3** | 1.15 | 1.73 | 1.27 | 1.22 | 3.83 | 0.96 | 3.90 | [b] | [b] |
| Brussels | 8.66 | 2.55 | **0.55** | 0.86 | 0.60 | 0.98 | 1.43 | [b] | [b] | [b] | 0.57 | 1.78 | **0.45** | 1.11 | 0.89 | **0.68** | 0.99 | 0.68 | 1.25 | 0.84 | 0.88 | 1.05 | [b] | [b] | [b] | [b] |
| Budapest | 2.06 | [b] | 0.99 | 0.87 | 1.29 | 1.11 | 1.32 | 1.83 | 1.51 | 2.55 | 0.75 | **0.67** | 1.21 | 1.14 | 1.11 | 1.07 | 1.00 | 0.82 | 1.54 | 0.90 | **0.43** | 0.69 | **2.11** | 1.38 | 1.57 | 0.93 |
| Helsinki | [b] | [b] | 1.00 | 0.78 | 1.36 | 0.97 | 1.56 | [b] | 1.93 | [b] | 3.09 | 0.85 | 0.67 | 0.73 | **1.85** | 1.07 | 1.08 | 0.98 | 1.70 | 0.87 | 1.02 | 1.91 | [b] | [b] | [b] | 5.73 |
| Košice | [b] | [b] | 0.64 | | 2.75 | 1.23 | 1.66 | [b] | [b] | [b] | 2.25 | 2.79 | 1.01 | 2.02 | 1.66 | 1.30 | 1.51 | 0.97 | [b] | [b] | 2.56 | 3.46 | **8.94** | **6.32** | [b] | [b] |
| Lisbon | **2.01** | **2.72** | **0.85** | **0.83** | 1.03 | 0.86 | **1.44** | 1.01 | 1.52 | 0.53 | 1.26 | 1.04 | 0.83 | 1.14 | 0.94 | 0.89 | **1.27** | **1.18** | 1.12 | 1.36 | 1.10 | 1.17 | 1.23 | 1.25 | 1.42 | **1.79** |
| London | **1.70** | **2.75** | 0.94 | **0.82** | 1.07 | **0.86** | **1.42** | 1.39 | 1.38 | 1.16 | 1.10 | 1.12 | **1.31** | 1.08 | **0.86** | **0.81** | 1.00 | **0.83** | **1.25** | 0.88 | **0.83** | 0.96 | [b] | [b] | [b] | [b] |
| Madrid | **3.76** | **3.78** | 0.97 | **0.87** | 1.15 | 1.13 | **1.33** | 0.93 | 1.62 | 0.68 | 0.96 | 0.83 | 1.11 | 1.13 | **1.13** | **1.15** | **1.14** | **1.15** | 1.29 | 0.96 | **1.22** | 1.11 | 0.77 | 1.25 | 0.69 | **0.64** |
| Prague | [b] | [b] | 1.02 | 1.26 | 0.83 | **2.88** | 0.86 | [b] | [b] | 0.92 | 0.37 | 1.19 | 2.27 | 0.94 | **2.38** | 1.05 | 0.95 | 1.05 | 1.52 | 0.66 | **0.50** | 0.67 | 2.67 | [b] | 0.61 | [b] |
| Rotterdam | **5.95** | 2.51 | 0.80 | **0.70** | **0.48** | 1.15 | 1.33 | [b] | [b] | 0.42 | [b] | [b] | 1.51 | **0.50** | 0.85 | 0.92 | 1.01 | 1.08 | 2.12 | 0.21 | 0.83 | 1.10 | 2.08 | 2.09 | [b] | 0.99 |
| Stockholm | **4.39** | 4.14 | 1.12 | 0.92 | 1.21 | 0.82 | **1.70** | 1.21 | 0.36 | 3.17 | 0.99 | 1.06 | 1.26 | 1.41 | **1.25** | 1.01 | **1.31** | 1.09 | **1.95** | 1.20 | 0.95 | 1.02 | 0.98 | 1.57 | 1.45 | 0.86 |
| Turin | **3.06** | **3.79** | 0.89 | **0.81** | 0.98 | 0.98 | **1.87** | 3.51 | 1.45 | 0.85 | 0.73 | 1.05 | **0.84** | 0.90 | 1.11 | **0.82** | 0.94 | **0.82** | 1.15 | 0.64 | 1.01 | 0.98 | **2.02** | **2.44** | 1.30 | [b] |
| Zurich | 1.04 | 1.36 | 0.89 | **0.64** | 1.09 | 0.83 | 1.94 | [b] | 4.18 | 0.84 | 1.44 | 0.58 | 1.14 | **0.61** | 0.72 | **0.43** | 0.85 | **0.43** | 1.01 | [b] | 0.88 | 0.60 | 0.89 | **2.49** | 0.56 | 1.21 |

[a] Statistically significant results at 95% level in bold

[b] These rate ratios could not be calculated because of an insufficient number of deaths and too many areas with zero deaths
