# Supplementary material for: Social differences in avoidable mortality between small areas of 15 European cities: an ecological study
Source: Int J Health Geogr. 2014 Mar 12;13:8. doi: 10.1186/1476-072X-13-8 (PMC4007807; doi:10.1186/1476-072X-13-8)
Supplement: Additional file 20 — Cause-specific mortality rate ratios (Graphs). [file 1476-072X-13-8-S20.pdf]

AIDS (HIV disease)

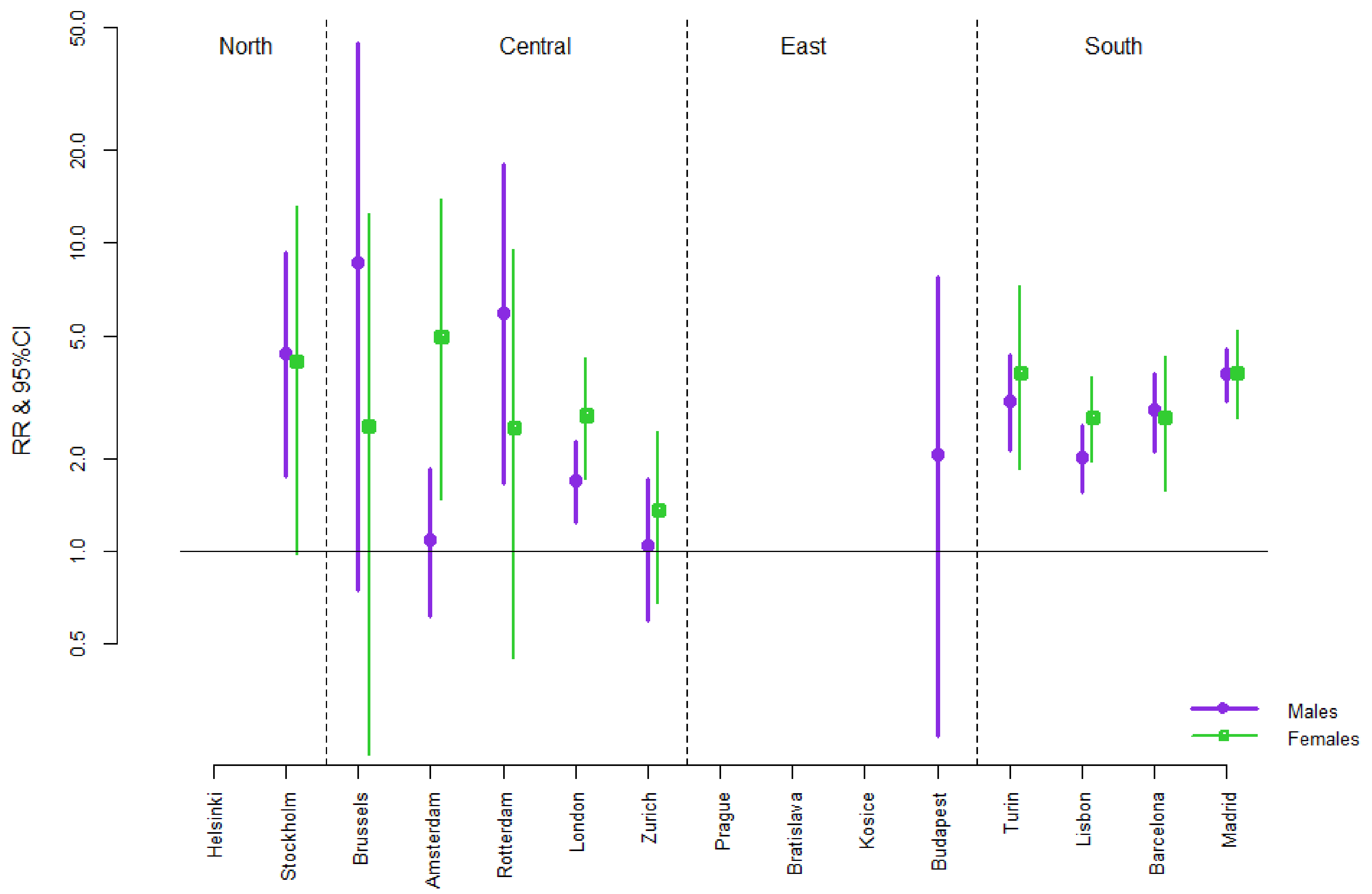

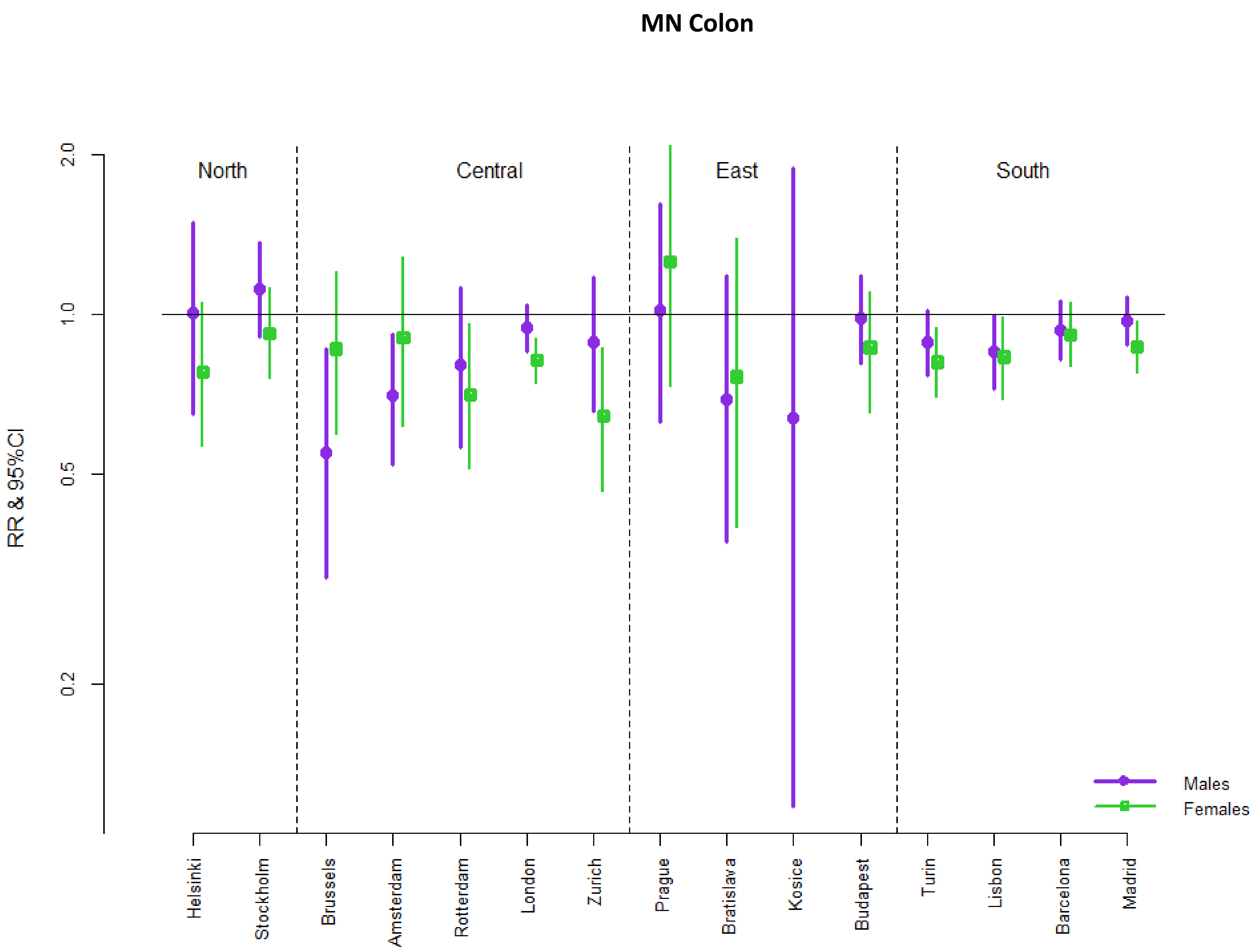

MN Colon
RR & 95%CI
2.0
1.0
0.5
0.2
North
Central
East
South
Males
Females
Helsinki
Stockholm
Brussels
Amsterdam
Rotterdam
London
Zurich
Prague
Bratislava
Kosice
Budapest
Turin
Lisbon
Barcelona
Madrid

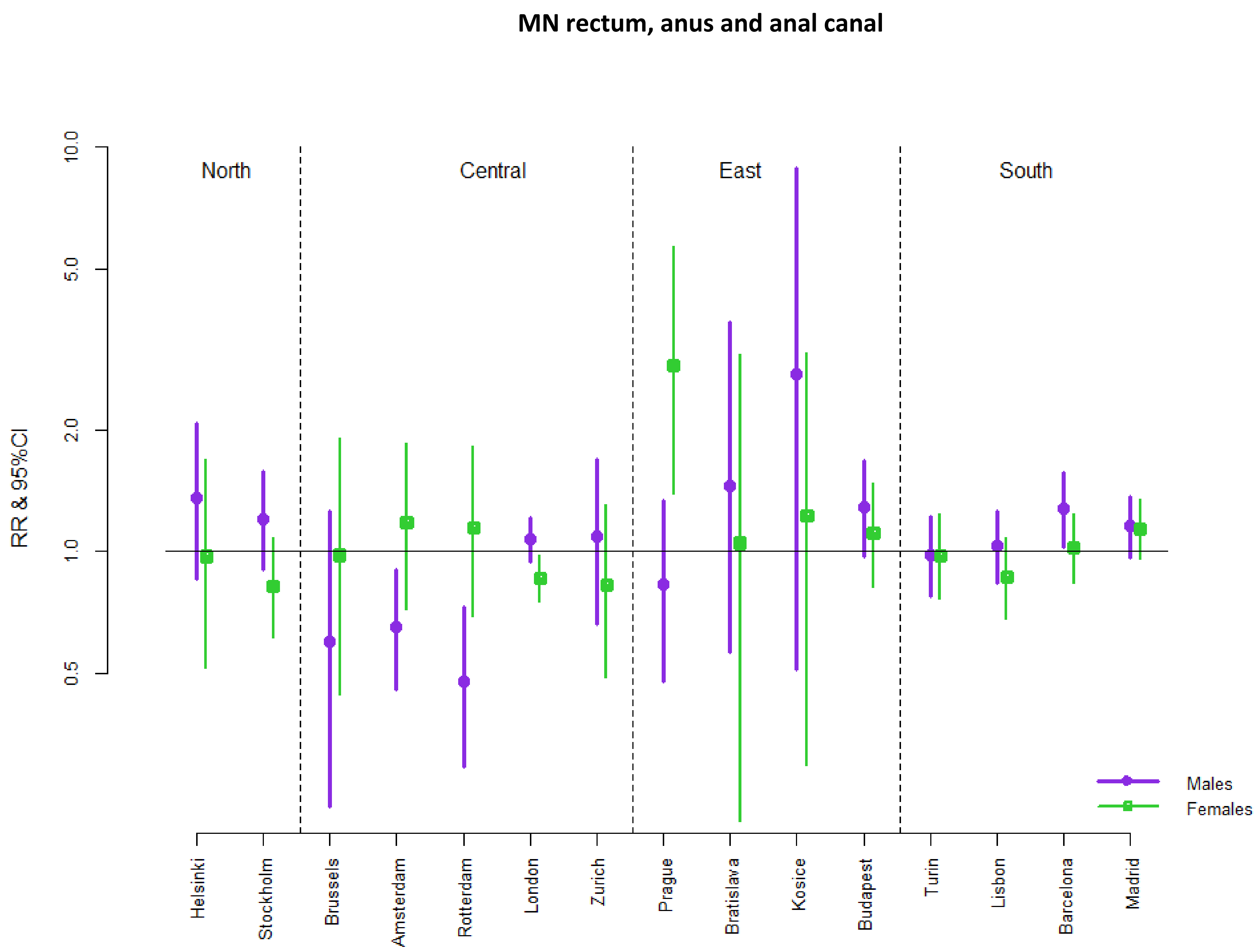

MN rectum, anus and anal canal
RR & 95%CI
10.0
5.0
2.0
1.0
0.5
North
Central
East
South
Males
Females
Helsinki
Stockholm
Brussels
Amsterdam
Rotterdam
London
Zurich
Prague
Bratislava
Kosice
Budapest
Turin
Lisbon
Barcelona
Madrid

MN Uterine Cervix

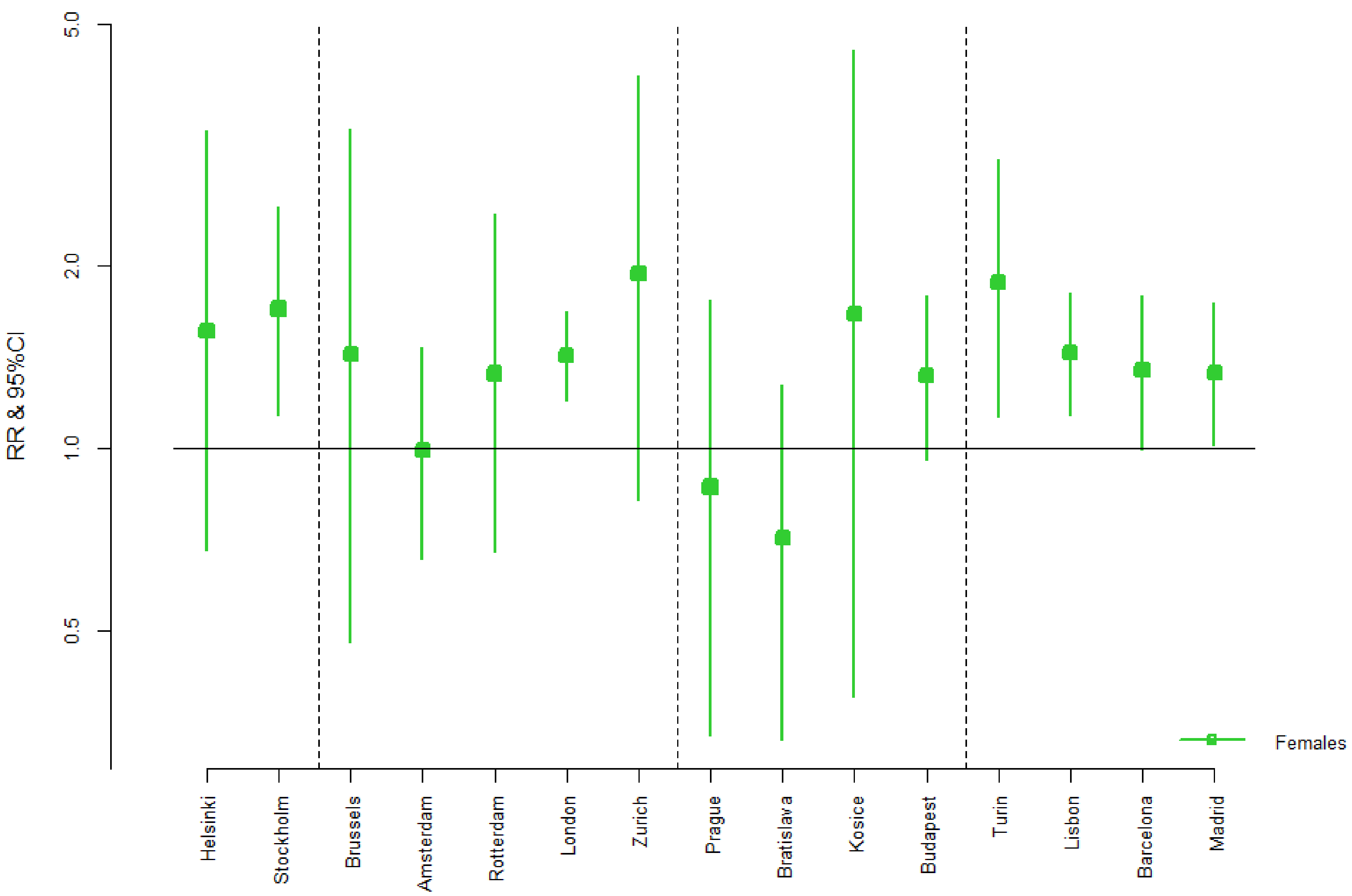

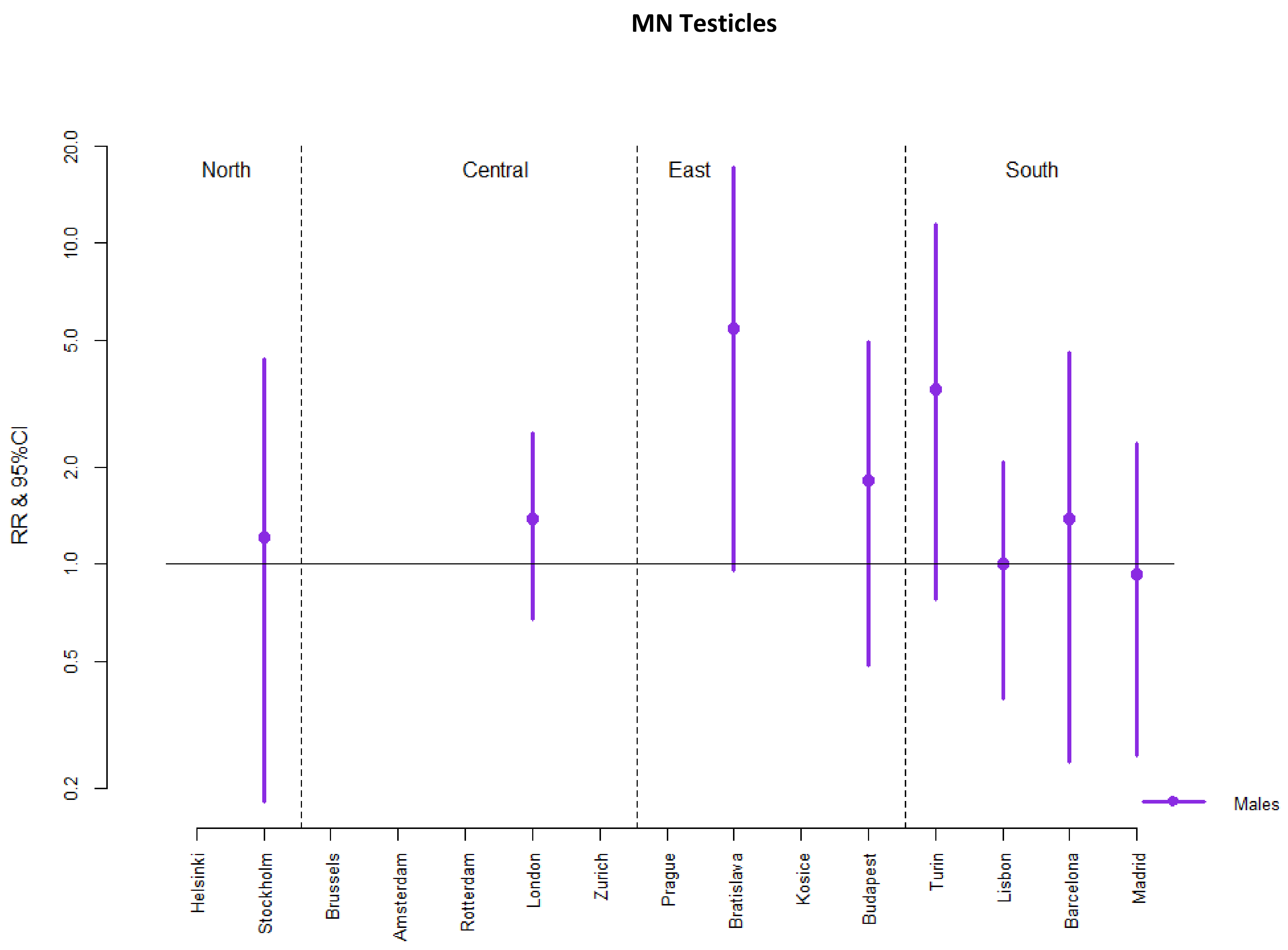
MN Testicles
RR & 95%CI
20.0
10.0
5.0
2.0
1.0
0.5
0.2
North
Central
East
South
Males
Helsinki
Stockholm
Brussels
Amsterdam
Rotterdam
London
Zurich
Prague
Bratislava
Kosice
Budapest
Turin
Lisbon
Barcelona
Madrid

Hodgkin's disease

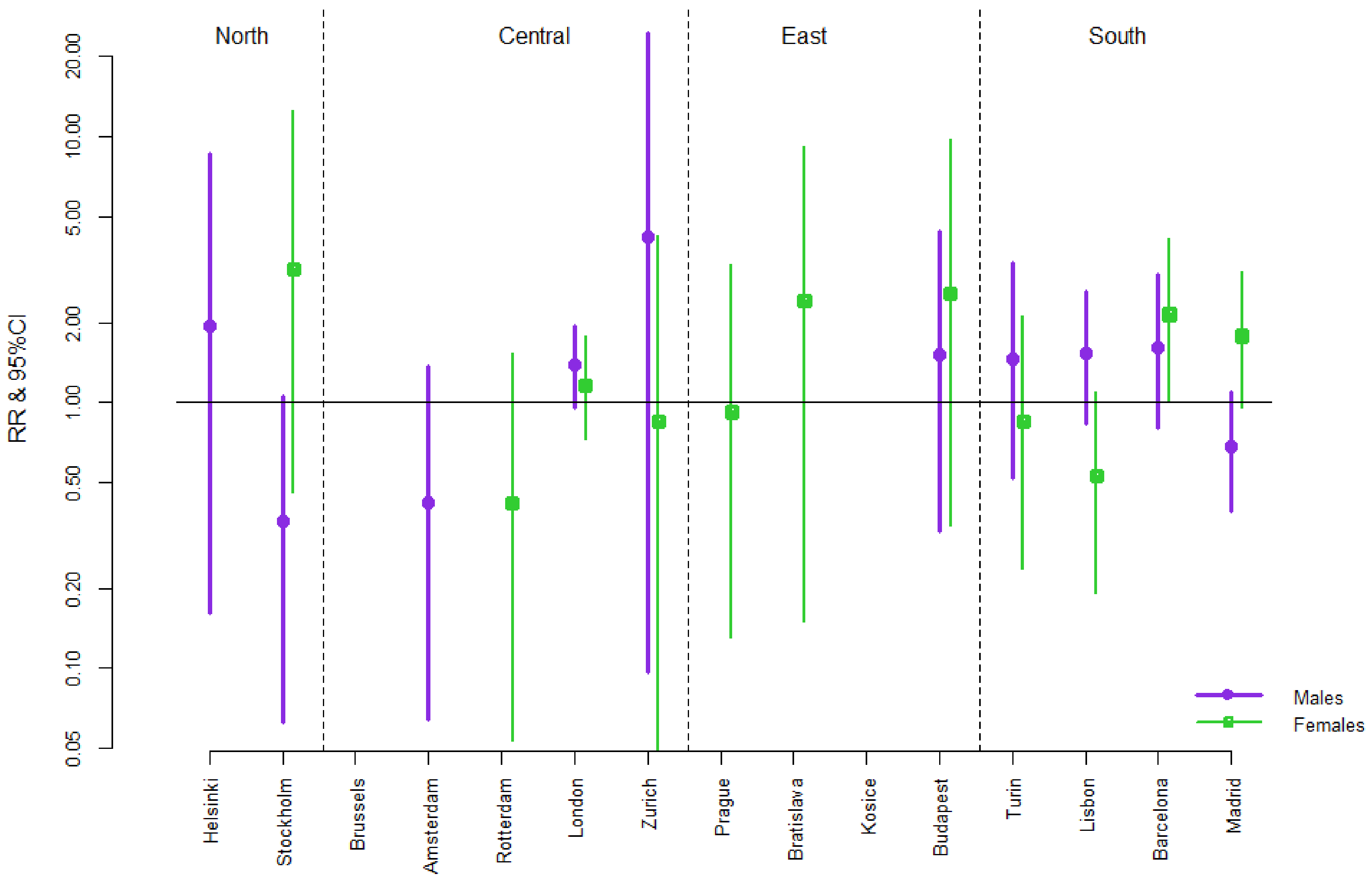

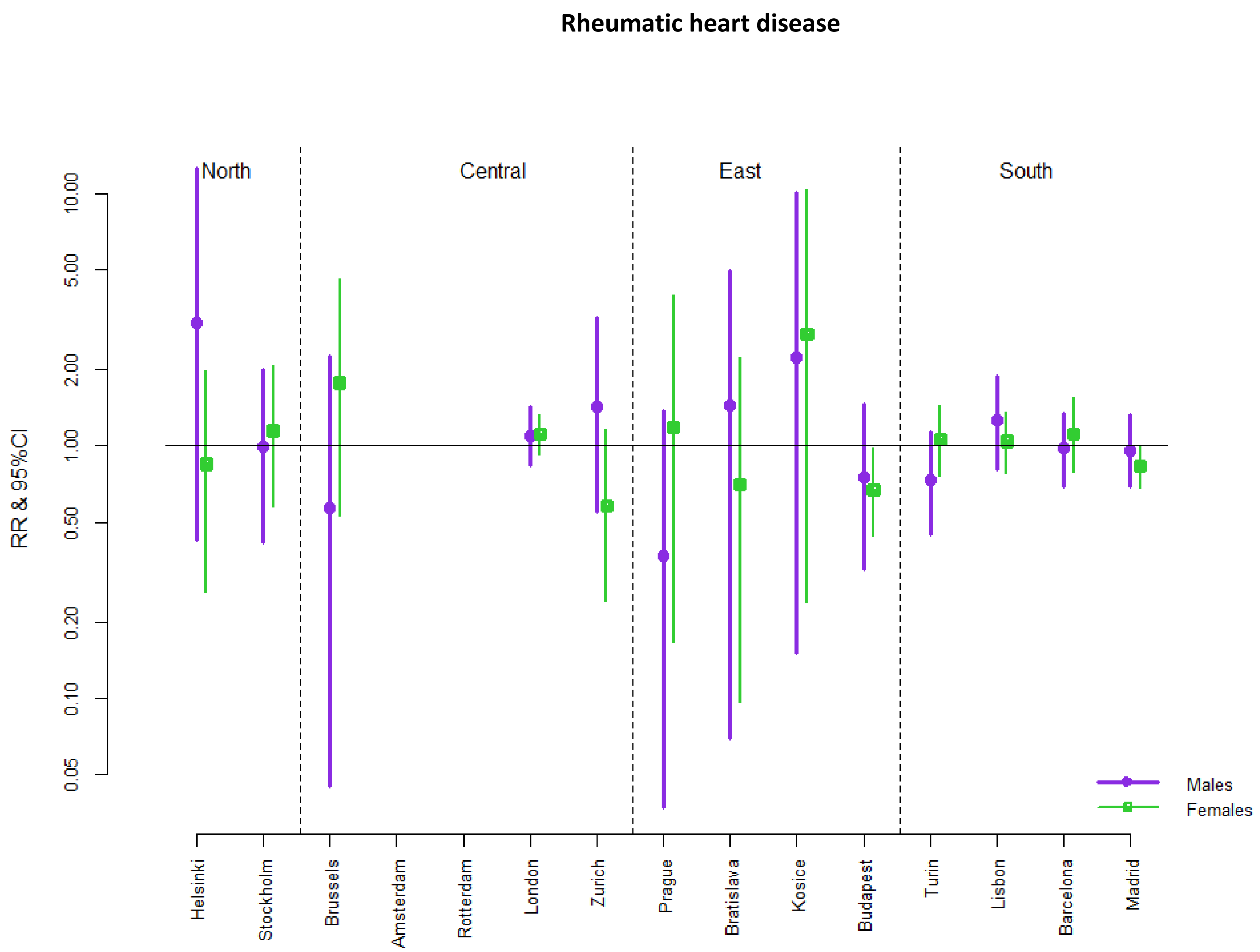
Rheumatic heart disease
RR & 95%CI
10.00
5.00
2.00
1.00
0.50
0.20
0.10
0.05
North
Central
East
South
Helsinki
Stockholm
Brussels
Amsterdam
Rotterdam
London
Zurich
Prague
Bratislava
Kosice
Budapest
Turin
Lisbon
Barcelona
Madrid
Males
Females

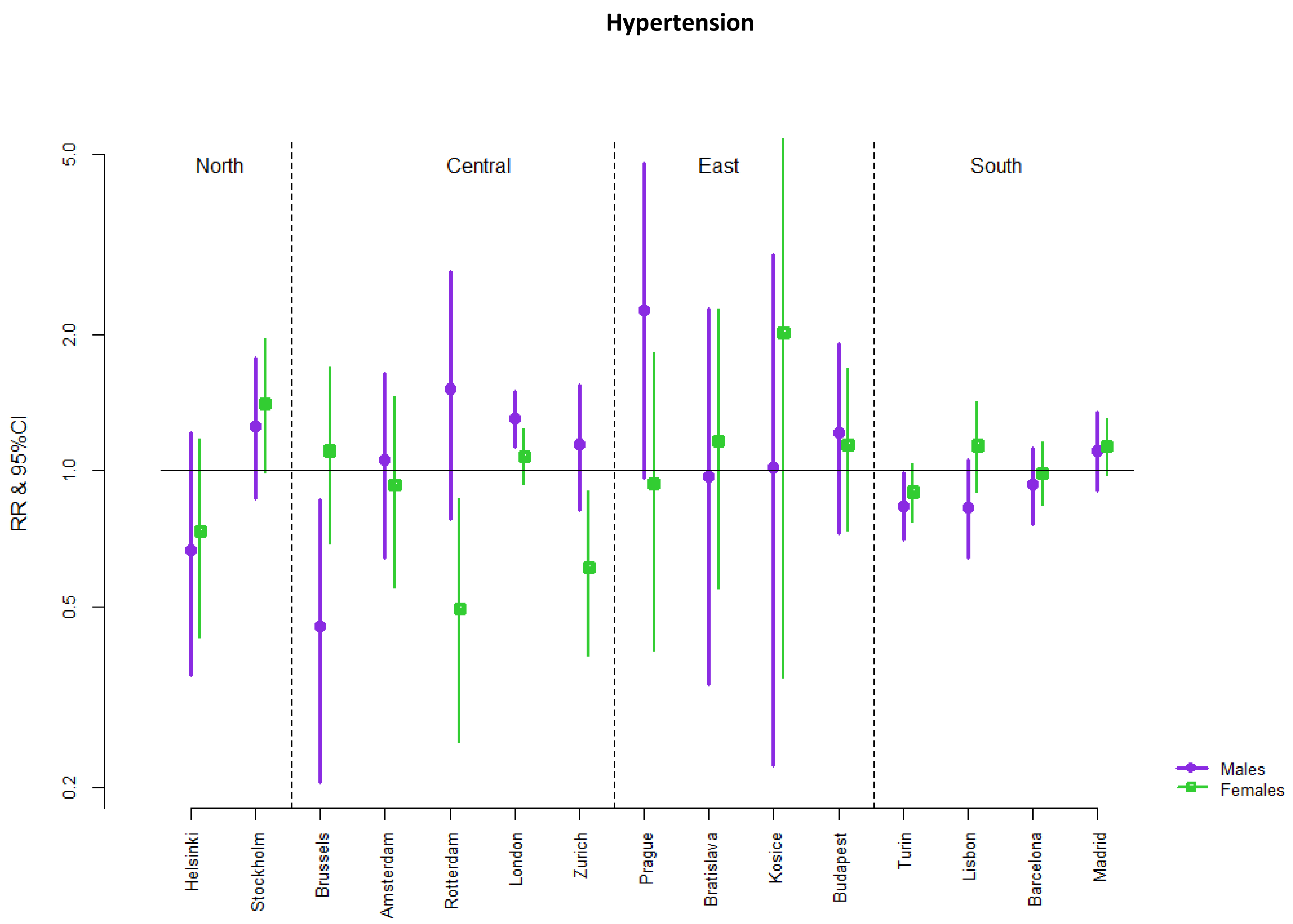
Hypertension
RR & 95%CI
5.0
2.0
1.0
0.5
0.2
North
Central
East
South
Helsinki
Stockholm
Brussels
Amsterdam
Rotterdam
London
Zurich
Prague
Bratislava
Kosice
Budapest
Turin
Lisbon
Barcelona
Madrid
Males
Females

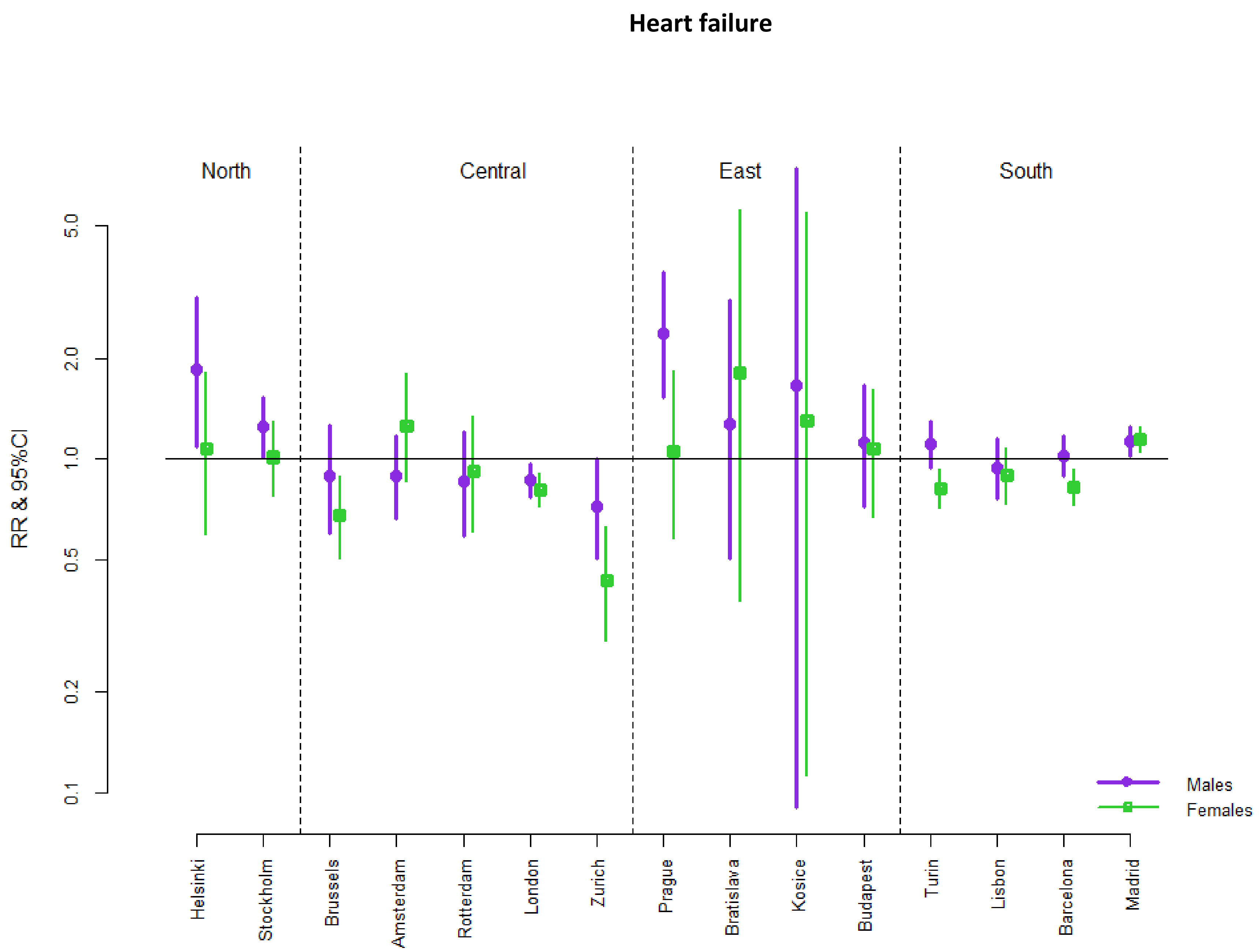

Heart failure
North
Central
East
South
RR & 95%CI
5.0
2.0
1.0
0.5
0.2
0.1
Males
Females
Helsinki
Stockholm
Brussels
Amsterdam
Rotterdam
London
Zurich
Prague
Bratislava
Kosice
Budapest
Turin
Lisbon
Barcelona
Madrid

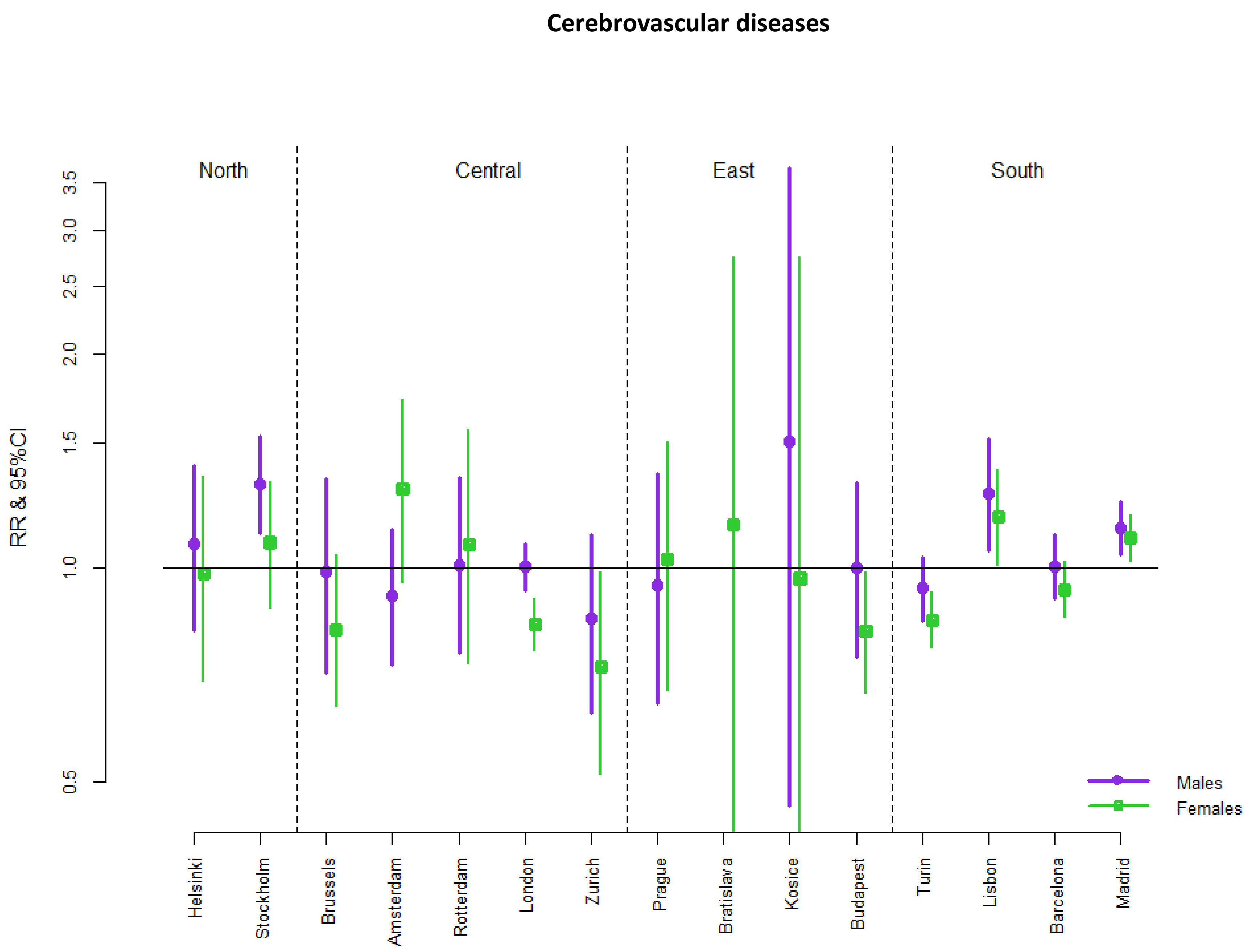

Cerebrovascular diseases
North
Central
East
South
RR & 95%CI
3.5
3.0
2.5
2.0
1.5
1.0
0.5
Males
Females
Helsinki
Stockholm
Brussels
Amsterdam
Rotterdam
London
Zurich
Prague
Bratislava
Kosice
Budapest
Turin
Lisbon
Barcelona
Madrid

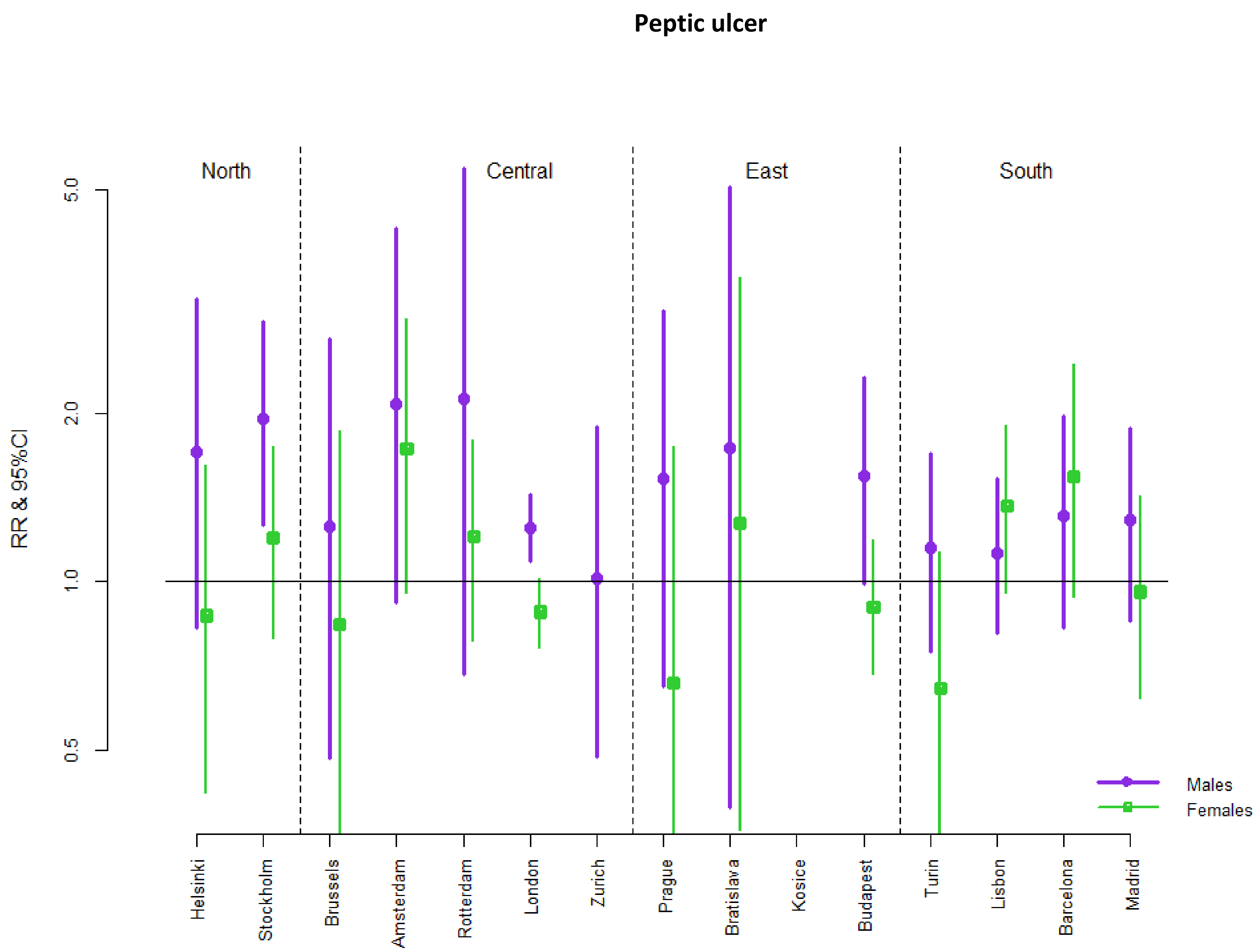
Peptic ulcer
North
Central
East
South
RR & 95%CI
5.0
2.0
1.0
0.5
Helsinki
Stockholm
Brussels
Amsterdam
Rotterdam
London
Zurich
Prague
Bratislava
Kosice
Budapest
Turin
Lisbon
Barcelona
Madrid
Males
Females

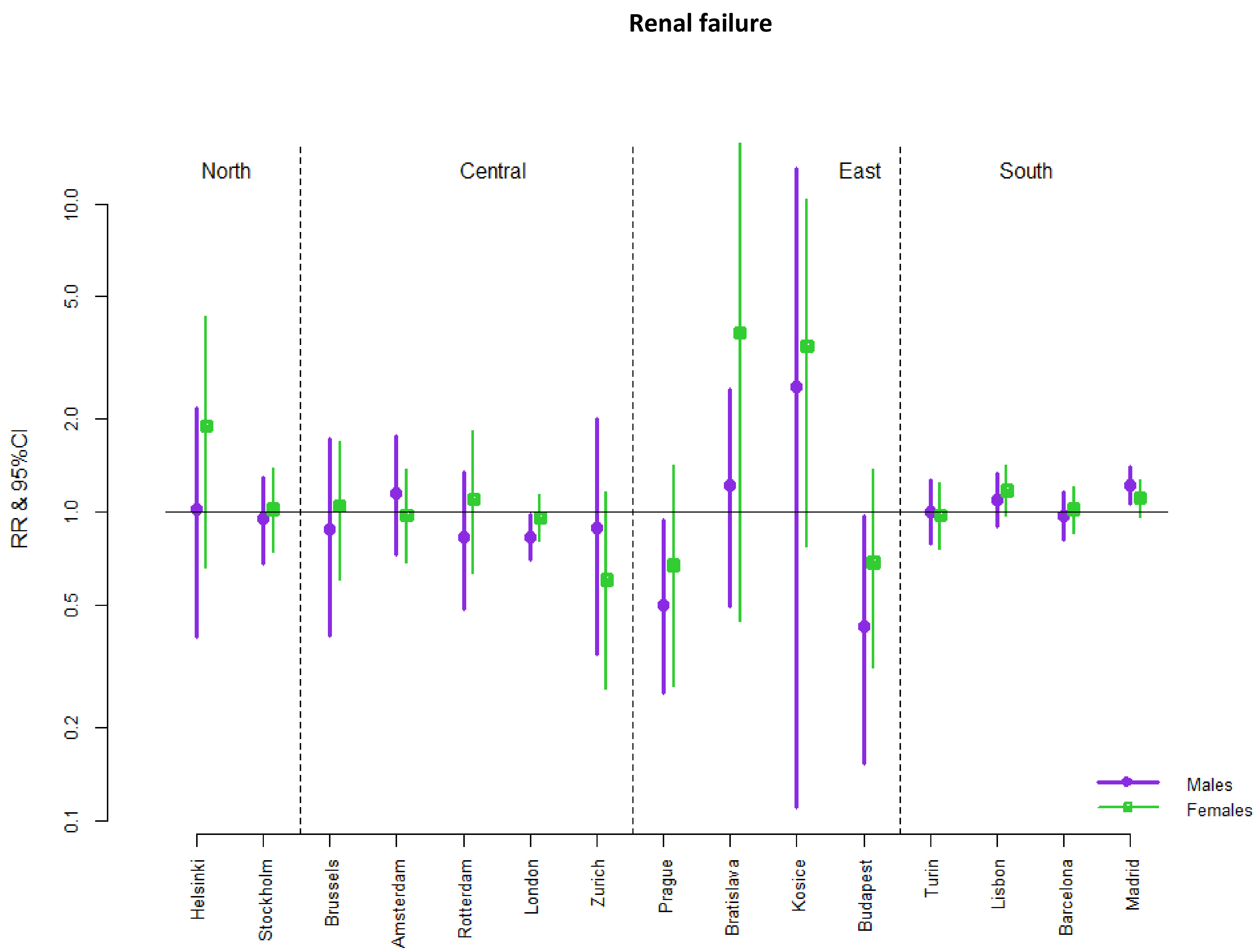
Renal failure
North
Central
East
South
RR & 95%CI
10.0
5.0
2.0
1.0
0.5
0.2
0.1
Helsinki
Stockholm
Brussels
Amsterdam
Rotterdam
London
Zurich
Prague
Bratislava
Kosice
Budapest
Turin
Lisbon
Barcelona
Madrid
Males
Females

Conditions originating in the perinatal period

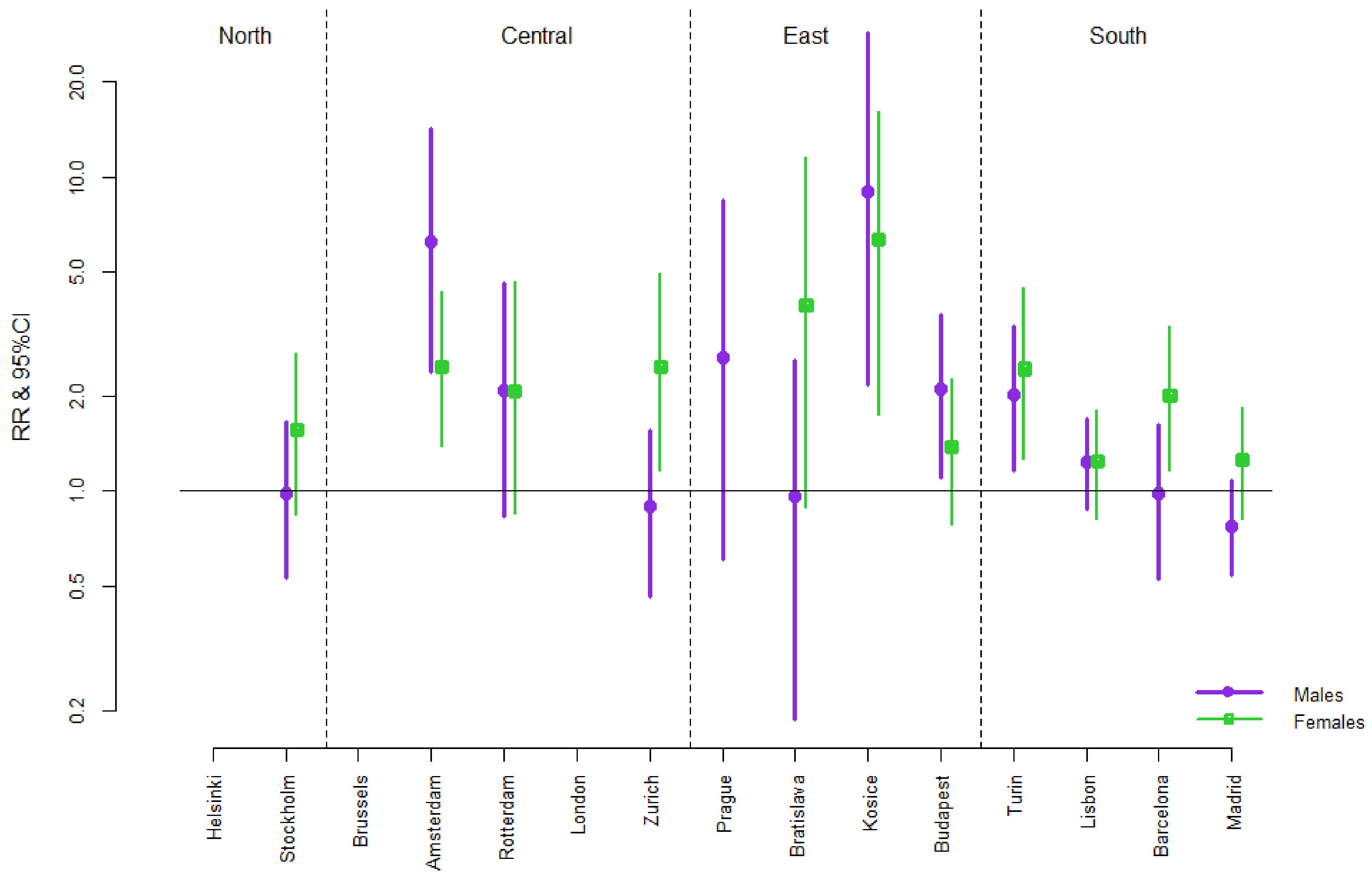

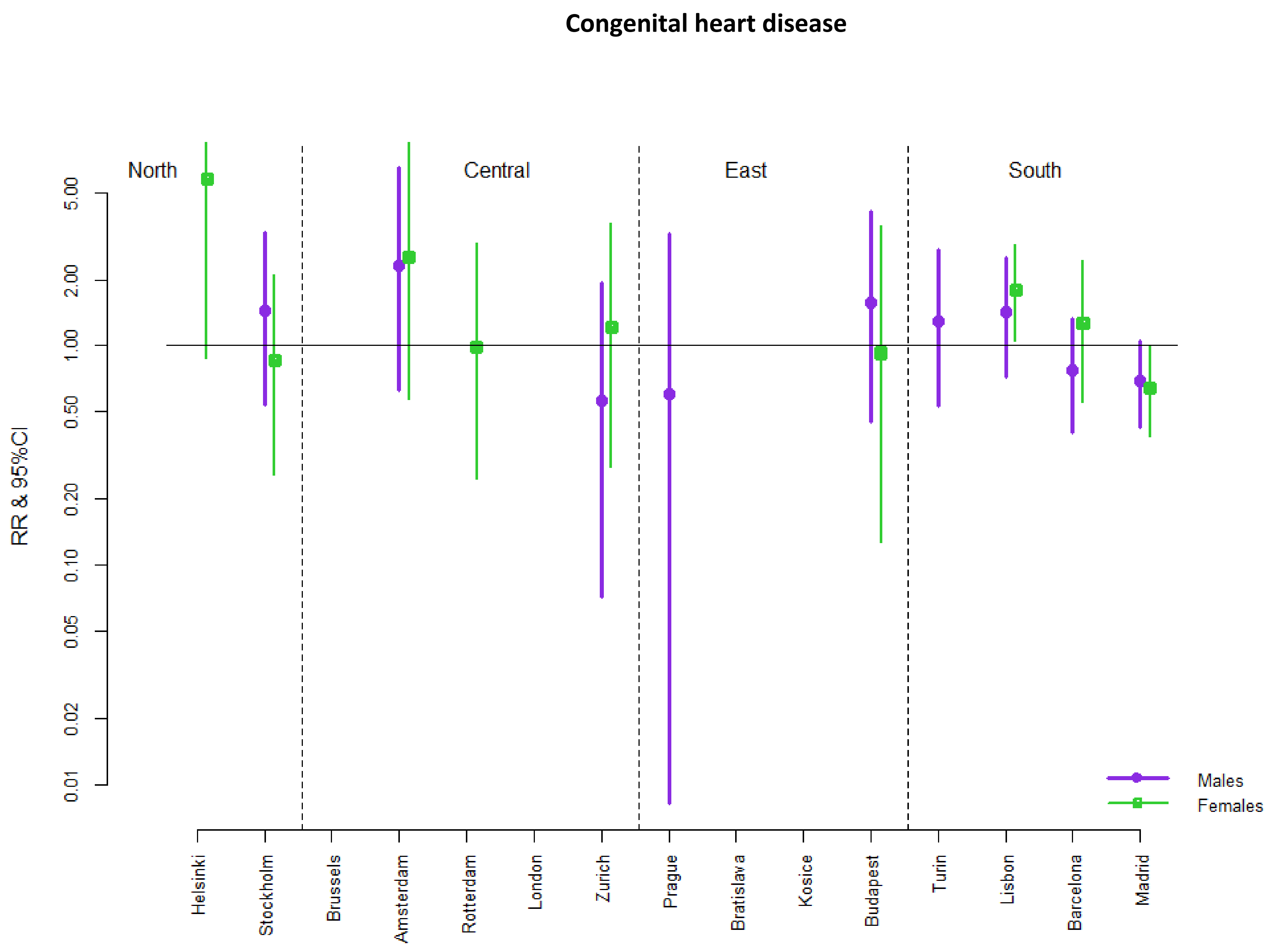
Congenital heart disease
North
Central
East
South
RR & 95%CI
5.00
2.00
1.00
0.50
0.20
0.10
0.05
0.02
0.01
Males
Females
Helsinki
Stockholm
Brussels
Amsterdam
Rotterdam
London
Zurich
Prague
Bratislava
Kosice
Budapest
Turin
Lisbon
Barcelona
Madrid
